# Macro-micromorphological, anatomical, and phytochemical characterization of *Cucumis melo* var. *agrestis* Naudin: a potential source of natural antioxidants

**DOI:** 10.1038/s41598-026-47246-7

**Published:** 2026-04-17

**Authors:** Faiza A. Shehata, Rim Hamdy, Ibrahim El Garf, Esraa M. Megahed

**Affiliations:** 1 Botany and Microbiology Department, Faculty of Science, Menoufia University, Shibin El Kom, Egypt; 2 Botany and Microbiology Department, Faculty of Science, Cairo University, Giza, 12613 Egypt; 3 Department of Agricultural Chemistry, Faculty of Agriculture, Minia University, El-Minia, Egypt; 4 Department of Biological Sciences, Faculty of Science, Galala University, New Galala City, Suez,43511, Egypt

**Keywords:** Anatomy, Antioxidant, *Cucumis melo* var. *agrestis*, DCPIP, HPLC, Pollen, Seed, Biochemistry, Plant sciences

## Abstract

A comprehensive study of macro-micro-morphological and anatomical seed and pollen characteristics of *Cucumis melo* var. *agrestis* (Cucurbitaceae) using plant materials gathered during field visits, the results showed that the plant is annual, pubescent, prostrate with a single hairy unbranched tendril, andro-monoecious; yellow perfect flowers and large male flowers on separate branches. Fruit yellowish green to yellow, fleshy, globular to ellipsoid, berry-like, indehiscent, pubescent when young, turns glabrous at maturity, many-seeded. Pollen grains are monads, sub-triangular, polar-shaped with tri-zonoporate aperture, reticulate texture, and foveolate ornamentation. Seed pale cream, obovoid with apical hilum, reticulate sculpture with hexagonal cells. The stem is circular with ridges; vascular bundles are arranged in two alternate rings.  The petiole has an oval outline with a groove along its the narrow side. The leaf has a U-shaped midrib region, and the mesophyll is differentiated into palisade and spongy. The leaf and fruit phenolic profiles, antioxidant actavity, and free radical scavenging activity were studied. A qualitative phytochemical screening procedure was performed on the 70% ethanolic extract. The Phytochemical study revealed the presence of tannins, flavonoids, alkaloids, saponins, steroids, fatty acids, coumarins, terpenoids, and glycosides. Total phenolic and flavonoid content in the leaves and fruit was determined. The data showed that the ethanolic extract of leaves records the highest concentration of 55.71 mg GAE/g and 9.013 mg QE/g. Antioxidant activity was evaluated using different assays, including KMnO₄, methylene blue, DCPIP, and DPPH. *In vitro* methods using leaf and fruit extracts, the leaf extract showed significant action towards free radicals in all methods. The ethanolic extract was analyzed by using high-performance liquid chromatography (HPLC) to identify its phenolic constituents. HPLC analysis of the ethanolic extract of Cucumis melo var. agrestis revealed 12 phenolic compounds in both leaf and fruit extracts . These findings highlight the potential of Cucumis melo var. agrestis as a promising natural source of antioxidant compounds and provide a basis for further pharmacological investigations.

**Supplementary Information:**

The online version contains supplementary material available at 10.1038/s41598-026-47246-7.

## Introduction

The Cucurbitaceae (cucurbits) or gourd family of plants includes around 965 species in 95 genera^1^. The family is found in temperate and tropical regions. Cucurbits are economically significant; many species’ fruits, like cucumbers and squash, are consumed by humans. According to Lucian and Teodosiu^2^, several Cucurbitaceae plant species have significant chemical compounds with potential medical uses.

Gourds or cucurbits are typically climbers or trailers and include significant economic plants such as watermelons, cucumbers, squash, and luffas^3^. Cucurbitaceae members are climbing or prostrate herbs with hairy, pentangular stems, leaves that are alternating and petiolate, with palmate veins. Flowers can be unisexual, monoecious, or dioecious, and fruits can be pepo, berry, or capsule^4–6^.

*Cucumis melon* was divided into two subspecies: *C. melo* subsp. *melo* and *C. melo* subsp. *agrestis*^7,8^ and it was recently categorized into sixteen subgroups, often known as “types” or “tribes”^9^.

The wild melon, *Cucumis melo* var. *agrestis*, is different from the cultivated variety in terms of fruit size, sweetness, and other traits. According to taxonomy, cultivated cultivars belong to *C. melo melo*, although wild forms are frequently categorized under the subspecies *C. melo agrestis*. The wild Cucumis species are of great commercial importance since they are the source of several beneficial gene sets for a range of yield-related agronomic traits, such as tolerance to biotic stress and disease^10^. Fruit is rich in nutrients, has a somewhat pleasant taste, and has significant health benefits like anti-inflammatory, analgesic, antioxidant, and hypoglycemic properties, according to Hui et al.^11^**.**

*Cucumis melo*var. *agrestis*, a member of the Cucurbitaceae family, is a wild herbaceous plant widely distributed across inland and coastal regions^12^. Commonly known as wild musk melon, kachari, or small guard, this species has garnered attention for its ecological adaptability and traditional medicinal uses. Phytochemical analyses reveal a rich composition of bioactive compounds, including tannins, flavonoids, alkaloids, phenolic components, steroids, resins, terpenoids, glycosides, and saponins, which collectively contribute to its therapeutic properties^13,14^**.**

### Economic importance

Many wild species are prized for their medicinal and commercial use. Among them is *Citrullus colocynthis*, a severely bitter herb used as a purgative since the Assyrian era^15^. In India and Malaysia, the fruit, seeds, and tender shoots of *Coccinia grandis* are edible. The root’s juice is used to treat diabetes, while the leaf juice is used to relieve earaches^16^. *Ecballium elaterium* fruit juice is used in traditional medicine as a purgative,  an antitumor, and an anti-inflammatory in traditional medicine^17,18^.

*Bryonia* is a medicinal and ornamental plant^15^^19^,.  The immature fruit and leaves of *Momordica balsamina* red pulp and seeds are edible, while the leaves and stems are utilized as camel fodder^20^. *Momordica charantia* immature fruit and leaves are edible in China and India, and the fruit extract possesses insulin-like properties^20,21^.

### Macro-morphology

For the Cucurbitaceae in Egypt, 11 species belonging to 6 genera^22^ and 7 genera^23^ were reported  . *Zehneria anomala* and *Kedrostis gijef* were added to Egypt’s flora^24^. (El-Hadidi and Fayed^25^ and Boulos and^26^increased the number of genera and species to 8 and 14, respectively; however, Boulos^27^ decreased the number of species to 13 by removing *Cucumis ficifolius* and adding *Cucumis figarei* var. *ficifolius* as a synonym for *Cucumis pustulatus*. *Momordica balsamina* and *Cucumis melo* subsp. *agrestis* were identified by El-Khanagry^28^ as new to Egypt’s flora.

(Rizk^29^,**)** studied the morphology and cytotaxonomy of 27 Egyptian taxa of landraces and cultivated cultivars from three genera (*Cucumis*,* Cucurbita*, and *Luffa*), six species, and five subspecies. Fruit characteristics , along with other vegetative traits characteristics at the varietal level provide an effective taxonomic tool.

Mabberley^30^ reported that *agrestis* is the wild form of *C. melo*with inedible fruit, exported from Sudan as oilseed, which escapes from cultivation and naturalizes in the Nile valley. El-Khanagry^28^ reported *Momordica balsamina* and *Cucumis melo* subsp. *agrestis along the canal bank*, in the * Giza region*,  as new records for the Egyptian flora.additionally,  an undated specimen lacking collector information from Qena is present in (CAI).

A taxonomic revision of Cucurbitaceae revealed 13 species belonged to nine genera, one subfamily, three tribes, and four subtribes, including *Ecballium elaterium* and *Momordica charantia*, which have been reintroduced to Egypt’s flora, and *Cucumis ficifolius* and *Momordica balsamina* have been recorded^31^.

According to Rabei et al.^32^, 27 taxa of cultivated and landrace Cucurbitaceae are found in Egypt,  belonging to three genera, six species, and five subspecies. These characters are grouped as follows based on their usefulness for identification: trichome type, fruit characters, and seed characters. Using 36 morphological features, including vegetative, floral, fruit, and seed characters, two keys to 27 cultivated varieties of Cucurbitaceae (*Cucumis*,* Cucurbita*, and *Luffa*) were created using the DELTA software system.

The floral morphology of seven species and two cultivars belonging to five genera of Cucurbitaceae, *Citrullus lanatus (*Thunb.*)* Matsum. & Naka, *Citrullus lanatus cv. Colocynthis*,* Cucurbita moschata* Duchesne, *Cucurbita pepo* L., *Cucumis sativus* L., *Cucumis melo* L., *Cucumis melo cv. flexuosus* (L.) Naudin and *Lagenaria siceraria*(Molina) Stand carried out by Rezk et al.^5^ the study focused on the morphological, micromorphological, and anatomical characteristics; the findings revealed that the flowers, anther forms, pollen grains, epidermal cell walls in sepals and petals, hairs, the anatomical flower, and the number of vascular bundles in the petiole play important taxonomic role in discrimination amongst taxa.

Hasson et al.^33^, studied several species in Iraq, including *Citrullus lanatus* (Thumb.) Mastum, *Cucumis melo* L., *Cucumis melo var. flexuosus* Naudia, *Cucumis sativus* L., *Cucurbita maxima* Duchesne, *Cucurbita moschata* (Duchesne) Poir, and *Luffa cylindrica* L. The findings revealed some unique morphological, anatomical, and palynological traits.

A total of 39  melon accessions accessions of melons, *Cucumis* melo subsp. *agrestis* var. *agrestis* (local names-‘choti kachri’, ‘badi kachri’, ‘sukkangai’), *C. melo* subsp. *agrestis* var. *momordica*, *C. melo* subsp. *agrestis* var. *conomon*, *C. melo* subsp. *melo* var. *flexuosus* and *C. melo* subsp. *agrestis* var. *alwarensis* (‘arya’) were examined for fruit morphological characteristics to determine their genetic resource value. This helped combine snake melon and “arya” for nutritional benefits and made pre-breeding programs easier by identifying distinct forms based on anther characteristics^34^.

In 2022, Masungsong et al. Classified  57 *Cucumis* (Cucurbitaceae) accessions into six species based on leaf characteristics: *C. melo* subsp. *agrestis*, *C. melo* var. *texanus*,* C. melo* var. *flexuosus*,* C. zambianus*,* C. sativus*, and *C. sativus* var. *hardwickii*. These species were characterized by a macrophyllous leaf blade, an odd-lobed structure with an acute apex angle, and tertiary veins diverging at obtuse angles.

Biology, phenology, and floral morphology of wild melon, namely *Cucumis melo* L. ssp. *agrestis* (Naudin) and *Pangalo* var. *agrestis*,  were studied by Chakravarthi et al., 2023. The ratio of staminate to pistillate flowers, the duration of the male flowers, the anther dehiscence time, pollen germination, and stigma receptivity were among the differentiation features.Fruits of *Cucumis melo* var. *agrestis* are solids of revolution. This study provides insights into the evolutionary aspects of fruit geometry in plants with egg-shaped fruits, introducing a practical tool for non-destructively calculating fruit volume and surface area from photographed 2D fruit profiles. (Ke^35^).

### Pollen

Cucurbitaceae is a eurypalynous family with distinct palynological characteristics. Previously, the family’s palynology was researched by Erdtman^36^, Marticorena^37^, Jeffrey^38,39^, Saad^40^, Moore and Webb^41^, Stafford and Sutton^42^, Khunwasi^43^, Pruesapan & Van der Ham^44^, Jeffery & Wilde (2006), and Perveen and Qaiser^45^**.**

SEM analysis of the pollen morphology of *Cucumis melo* subsp. *agrestis* var. *agrestis*showed tricolporate pollen, sunken interapertural area, and a reticulate type of exine sculpturing. Akhtar et al.^46^.

Palynological characteristics of 24 species belonging to the family Cucurbitaceae, including *Cucumis melo* var. *agrestis* L using scanning electron microscopy and light microscopy investigated by^47^. This study’s quantitative and qualitative pollen data provide important insights into the systematics of the Cucurbitaceae family’s taxonomy.

### Seed

Seed morphology provides several traits that are potentially useful for species identification, phylogenetic inference, and character-state evolution^48–52^.

Seed morphology has been traditionally used in taxonomy with variable degrees of effectiveness across plant families of plants^53–56^. Seed morphology distinguishes various species, confirms the tribe and subtribe classifications as suggested by Schaefer and Renner^57^, Jeffrey^58^, and Achigan-Dako^59^.

Using stereomicroscopy and scanning electron microscopy, the seed coat morphology of 16 taxa from 11 genera of the Cucurbitaceae was investigated. A taxonomic key for some Cucurbitaceae species was developed using several seed characteristics, including size, shape, and surface details of the seed coat^60^.

Diverse *Cucumis* L. species, as well as *Echinocystis lobata* (Michx.) Torr. & A. Gray and *Lagenaria sphaerica* (Sond.) Naudin, The results support a relationship between seed shape and species ecology^61^.

Eleven species in nine genera of the family Cucurbitaceae in Nigeria, *Citrullus lanatus*,* Citrullus mucosospermus*,* Cucumis sativus*,* Cucurbita maxima*,* Lagenaria siceraria*,* Lagenaria sphaerica*,* Luffa aegyptiaca*,* Momordica charantia*,* Siraitia africana*,* Telfairia occidentalis*, and *Trichosanthes cucumerina*, were investigated by Umoh and Bassey^62^. The results show variation in morphology across the stem, leaves, petals, fruit, and seeds .

Martín-Gómez^63^ analyzed seeds from seven genera of the Cucurbitaceae: *Bryonia*,* Citrullus*,* Coccinia*,* Cucumis*,* Cucurbita*,* Ecballium*,* Momordica*, and *Sicana* using three quantitative morphological methods. The results provide valuable information for taxonomy.

### Anatomy

Comparative anatomy of the fruit stalk and tendril of nine species representing 8 genera of Cucurbits from Nigeria, *Zehneria*,* Luffa*,* Momordica*,* Coccinnia*,* Telfairia*,* Cucurbita*,* Lagenaria*, and *Cucumis* has been carried out to complement the existing taxonomic data on the family^64^. The study’s findings revealed that similarities and differences in shape, number, and size of vascular bundles, nature of epidermis, layers, and nature of sclerenchymatous, collenchymatous, and chlorenchymatous cells in the fruit stalk and tendril could be used to distinguish between taxa.

Anatomical features of five genera in the family Cucurbitaceae, *Colocynthis*,* Cucumis*,* Cucurbita*,* Citrullus*, and *Luffa*, were investigated by Mohammed and Guma^65^; root, stem, and leaf anatomy have played a major role in the identification, characterization, and delimitation of taxa.

Trichomes on the lower surface of the leaves imply considerable taxonomic value and are widely recognized in the Cucurbitaceae^66^.

Flower stalk anatomy, stem, petiole, tendrils, and median vein shape of three representative Cucurbitaceae members: *Citrullus lanatus*,* Cucumeropsis mannii*, and *Citrullus colocynthis*^67^. These anatomical features suggest taxonomic affinity among the species, improve species delimitation, and reinforce the view that the species are maintained as separate species; anatomical characteristics are an important line of evidence in the classification of these species.

The morphological and stem anatomical characteristics of *Coccinia grandis* (L.) Voigt, *Luffa acutangula* (L.) Roxb., *Lagenaria siceraria* (Molina) Standl. and *Cucurbita pepo*L., belonging to Cucurbitaceae, were studied by Kumar et al.^68^; characteristics aid in the plant identification.

Thirteen genera, 23 species of the family Cucurbitaceae, including *Cucumis melo* var. *agrestis*Naudin) were studied for trichome micromorphology using a scanning electron microscope (SEM).The trichomes in the family Cucurbitaceae range from unicellular to multicellular, conical to elongated, glabrous to ridged, with or without a flattened disk at the base and cyctolithic appendages, thin to thick-walled, curved at the apices to blunt. (Ali and Al-Hemaid^69^) showed that the micromorphology of trichomes in the Cucurbitaceae family was noteworthy from a taxonomical standpoint.

The tendril anatomy of 17 taxa of Cucurbitaceae including *Cucumis* (five species), *Cucurbita* and *Luffa* (three species each), *Citrullus* and *Momordica* (two species each) while *Lagenaria* and *Praecitrullus* (one species each) carried out by^70^; the anatomical morphometry of tendril micromorphological features in Cucurbitaceous taxa exhibited variations, and provides trustworthy traits that help identify different species.

Phytochemical analyses.

Antioxidants are essential because they scavenge free radicals, which are the primary cause of most illnesses. These antioxidants prevent the cells from experiencing oxidative stress. Endogenous antioxidants such as glutathione, catalase, and superoxidase dismutase are produced by human systems^71^; Valko et al.,, 2004).

## Materials and methods

### Plant material and authentication

Plant materials of Cucumis melo var. agrestis.

Naudin were collected from Kom Hamada, Beheira Governorate, Egypt. The collected specimens were examined and authenticated by Prof. Rim S. Hamdy, Professor of Plant Taxonomy and Flora, Botany and Microbiology Department, Faculty of Science, Cairo University. A voucher specimen was deposited in the Herbarium of the Botany Department, Faculty of Science, Cairo University (CAI), Egypt, under voucher number CAI-72.308.05.396.

### Statement of plant collection permission

The collection of plant materials was carried out in accordance with local and national regulations, and no specific permission or license was required for this plant species.

### Sampling & macro-morphological investigations

Whole plant samples were collected from their natural habitat at Kom Hamada, Beheira Governorate, Egypt. The identification was conducted by Prof.Ibrahim El Garf , a member of the Cairo University Herbarium and Professor of Taxonomy and Flora in the Botany and Microbiology, Faculty of Science, Cairo University. The specimen was identified as *Cucumis melo* var. *agrestis* (Family: Cucurbitaceae) through the use of using floral keys and comparative analysis with verified specimens housed in the Cairo University Herbarium (CAI), specifically specimen number CAI.

Macro-morphological characteristics were recorded directly from approximately 15–20 fresh specimens before preservation as voucher specimens, and examination was performed using a binocular stereo light microscope (Leica Wild M3C, Heerbrugg, Switzerland). All images were taken using a digital camera (Samsung Mobile A50, Note 10 Lite, and realme Mobile RMX3085).

### Pollen and seed micro-morphological investigations

The macro-morphological characteristics of pollen and seeds of approximately 40 specimens, acquired from fresh material, were investigated using a light microscope. Pollen and seed from SEM-dried samples were mounted on brass stubs and coated with a thin layer of gold using a JEOL JSM-IT200 Scanning Electron Microscope (with an accelerating voltage of 20 KV) at Alexandria University in Egypt.

The terminology used to describe the micromorphological properties of pollen is compatible with previous publications^36,72–74^^,^^75^.

### Anatomical investigations

Sections of the vegetative organs (stem, petiole, and leaf) were taken from fresh material. All assessments were performed on all plants at fruiting developmental stages. Samples were collected from the fourth internode from the apex, about 2–3 cm long, and then fixed in FAA (Formalin-glacial acetic acid-70% ethyl alcohol, 5:5:90 V/V). After 24 h of fixing, the specimens were immersed in ethyl alcohol and embedded in paraffin wax.

The specimens were sectioned at 10–15 μm using a rotary microtome and dehydrated in an alcohol-xylol series. Sections were dyed with Safranin and light green according to the method of ^76^. The anatomical features were studied with a Zeiss light stereomicroscope and photographed with a digital camera (OPTIKA). A planimeter was used to estimate the proportion of each tissue to the total section area. Terminology according to Abd El-Rahman et al.^77^, Pandey^78^,and Abd El-Gawad et al.^79^.

### Chemicals, reagents, and instruments

The chemicals used in the present study are of high grade and high purity. These include: ethanol 70%,concentrated H_2_SO_4_ , Chloroform, Lead acetate, 33% Ammonia, FeCl_3_., NH_4_OH, Mayer’s reagent, Sodium hydroxide NaOH, Vanillin, HCl, Folin Ciocalteu reagent, Gallic acid, Potassium acetate (CH_3_-COOK), 2,2-diphenyl-1-picrylhydrazyl (DPPH), methylene blue, Potassium permanganate (KMnO_4_) and 2,6-Dichlorophenolindophenol (DCPIP), quercetin, ascorbic acid.

### Preparation of samples for analysis

The selected plant parts (fruits and leaves) were thoroughly cleaned with distilled water to remove dust, then shade-dried for seven days. The samples were then ground into a powder using a Bajaj GX1 (500) W grinder. An airtight container was filled with 250 g of coarse powder and 1000 mL of 70% ethanol. The sample was sealed and left to macerate for seven days, with occasional shaking. The maceration material was filtered through a Whatman filter (Paper No. 1) after being run through muslin cloth on the eighth day. At 25 °C, the filtrate was concentrated using a Büchi Rotavapor R-200 under reduced pressure. Finally, dark brown color semisolid mass (% yield 27.85 g cured extract from leaves and 36.8 g cured extract from fruits^80^.

### Phytochemical screening for each extract

Phytochemical screening was conducted using standard methods. Steroids were detected by adding concentrated H₂SO₄ to a chloroform mixture, resulting in a red upper layer and a yellowish-green fluorescence method^81^**.** Terpenoids were identified by forming a blue-green ring using the acetic anhydride and H₂SO₄ method^82^**.** Tannins were confirmed by a reddish precipitate with lead acetate^83^**.** Saponins were detected by foam production after shaking the extract with water^84^**.** Anthocyanins were indicated by a color change from pink-red to blue-violet with HCl and ammonia^85^**.** Glycosides were confirmed by a brown ring using FeCl₃ and H₂SO₄^86^**.** Emodins were detected by red color with benzene and NH₄OH. Alkaloids were confirmed by a cream-colored precipitate with Mayer’s reagent method^81^, and phenolics and flavonoids were detected by distinctive color changes with FeCl₃ and sodium hydroxide, respectively^81,86^**.**

### Determination of phytochemical compounds

#### Total phenolic compounds

The total amount of phenolic compounds in extracts was determined with the Folin–Ciocalteu reagent. All tests were carried out in triplicate, and the total phenol was expressed as mg of gallic acid equivalents (GAEs) per g of extract. For this reason, the calibration curve of gallic acid was developed. Standard solutions of gallic acid at concentrations of 0.01, 0.02, 0.03, 0.04, and 0.05 mg/mL were prepared in methanol, with 1 mL of each used to construct the calibration curve. A concentration of 1 mg/mL of extract in methanol was also made , and 0.5 mL of the previous solution was transferred into test tubes and combined with 2.5 mL of 10-fold diluted Folin reagent and two mL of 7.5% sodium carbonate solution. The tubes were covered with parafilm. The tubes were covered with parafilm and allowed to stand for 30 min at room temperature, and the absorbance was read at 750 nm^87^**.**

### Total flavonoids

The flavonoid content of each extract was measured based on the methods described by Ebrahimzadeh et al.^88^. Briefly, 1.5 mL of methanol was mixed with the sample (1 mg/mL), followed by the addition of 2.8 mL of distilled water, 0.1 mL of 10% AlCl₃, and 0.1 mL of 1 M potassium acetate. For half an hour, the mixture was incubated at room temperature. A spectrophotometer was used to measure the absorbance at 415 nm. Milligrams of quercetin equivalents (QE) per gram of extract (mg QE/g extract) were used to express the results. Quercetin was used to create the standard curve at different concentrations (5–50 mg/L).

### Antioxidant activity

#### Measurement of the antioxidant power of vitamin C in comparison samples

The procedure involved pipetting 1000 ppm of Vitamin C solution along varying volumes of the sample (1000, 500, 400, 200, 100, 50, and 25 µg/mL). Subsequently, 200 µL of each reagent DPPH, methylene blue, KMnO₄, and DCPIP was added to the mixture. Following this, 800 µL of distilled water was added, and the mixture was incubated at room temperature in the dark . The absorbance of the substance was recorded at four different wavelengths: 517 nm, 660 nm, 514 nm, and 600 nm^89^.

#### Potassium permanganate method

The scavenging activity of the crude extract were characterized as follows: Potassium permanganate solution was made by dissolving 0.04 g of KMnO4 in 100 ml of distilled water. Various samples (1000, 500, 400, 200,100, 50, and 25 µL) were placed in a test tube with 200 µL of KMnO_4_ and 800 µL of distilled water. After vortexing the mixture for 1 min, it was incubated at room temperature for 30 min in the dark. The sample solutions and ascorbic acid, used as a natural antioxidant reference, were evaluated for absorbance at a wavelength of 514 nm^90^.

The percentage (%) of scavenging activity was calculated as the follows: % antioxidant activity =(control−sample)/control ×100.

#### Methylene blue method

The scavenging activity of the crude extract were as follows. A solution of methylene blue was made by dissolving 0.04 g of methylene blue in 100 ml of distilled water. Various samples (1000, 500, 400, 200,100, 50, and 25 µL) were prepared and combined with a test tube containing 200 µL methylene blue and 800 µL distilled water. After vortexing the mixture for 1 min, it was left at room temperature for 30 min in the dark. The sample solutions and ascorbic acid, used as a natural antioxidant reference, were tested for their absorbances at a wavelength of 660 nm^90^. The percentage (%) of scavenging activity was calculated as follows: % antioxidant activity =(control−sample)/control ×100, where the control is methylene blue solution(0.04%).

#### DCPIP scavenging assay

The scavenging activity of cured extracts were assessed using a novel approach as follows: A 1.0 ml aliquot of the sample, with varying concentrations of 1000, 500, 400, 200,100, 50, and 25 µg/mL, was added to a test tube. Then, 1.0 mL of a 0.04 g/100 ml DCPIP solution, which is soluble in an aqueous ethanol solution with a concentration of 70%, was added to the same test tube. The mixture was agitated using a vortex mixer for 1 min and maintained at room temperature for varying intervals, 5 min, all while being shielded from light. The sample solutions were analyzed using a Genway spectrophotometer to measure their absorbance at a wavelength of 600 nm^91^.

% antioxidant activity =(control−sample)/control ×100, where the control is DCPIP solution (0.04%) .

#### Evaluation of antioxidant activity by the DPPH radical scavenging method

The antioxidant activity of various leaf extracts was assessed via the 1,1-diphenyl-2-picrylhydrazyl (DPPH) free radical scavenging method. A stock solution of 0.1 mM DPPH in ethanol was prepared, and serial concentrations of the test extracts (3.9–1000 µg/mL) were diluted ethanol as the solvent. For the assay, 1 mL of the DPPH stock solution was combined with the extract solutions at a 1:3 (v/v) ratio. The mixtures were vigorously shaken at room temperature for 20–30 min, after which their absorbance was measured at 517 nm. All experiments were carried out in triplicate, employing ascorbic acid as the reference standard under identical experimental conditions^92,93^. The percentage of DPPH radical scavenging activity was calculated using the following formula: DPPH scavenging effect (%).

Percent inhibition = A0 - A1/A0 × 100, where A0 was the absorbance of the control (without sample) reaction, and A1 was the absorbance in the presence of the sample solution.

#### HPLC conditions

HPLC analysis was performed using an Agilent 1260 series system. Separation was achieved on a Zorbax Eclipse Plus C8 column (4.6 mm × 250 mm i.d., 5 μm particle size). The mobile phase was composed of water (A) and 0.05% trifluoroacetic acid in acetonitrile (B) at a flow rate of 0.9 ml/min. The mobile phase was programmed sequentially in a linear gradient, as follows: 0 min (82% A); 0–1 min (82% A); 1–11 min (75% A); 11–18 min (60% A); 18–22 min (82% A); and 22–24 min (82% A). The multi-wavelength detector was examined at 280 nm. The injection volume was 5 µl per sample solution. The column temperature was maintained at 40 °C.

#### Statistical analysis

All experiments were performed in triplicate (*n* = 3), and the results are expressed as mean ± standard deviation (SD). Statistical analysis was carried out using one-way analysis of variance (ANOVA) with GraphPad Prism^®^ software (GraphPad Software, San Diego, CA, USA) according to the method described by Motulsky (1999). Differences were considered statistically significant at *p* < 0.05.

## Results and discussion

### Macromorphological results

**Fig. 1:**
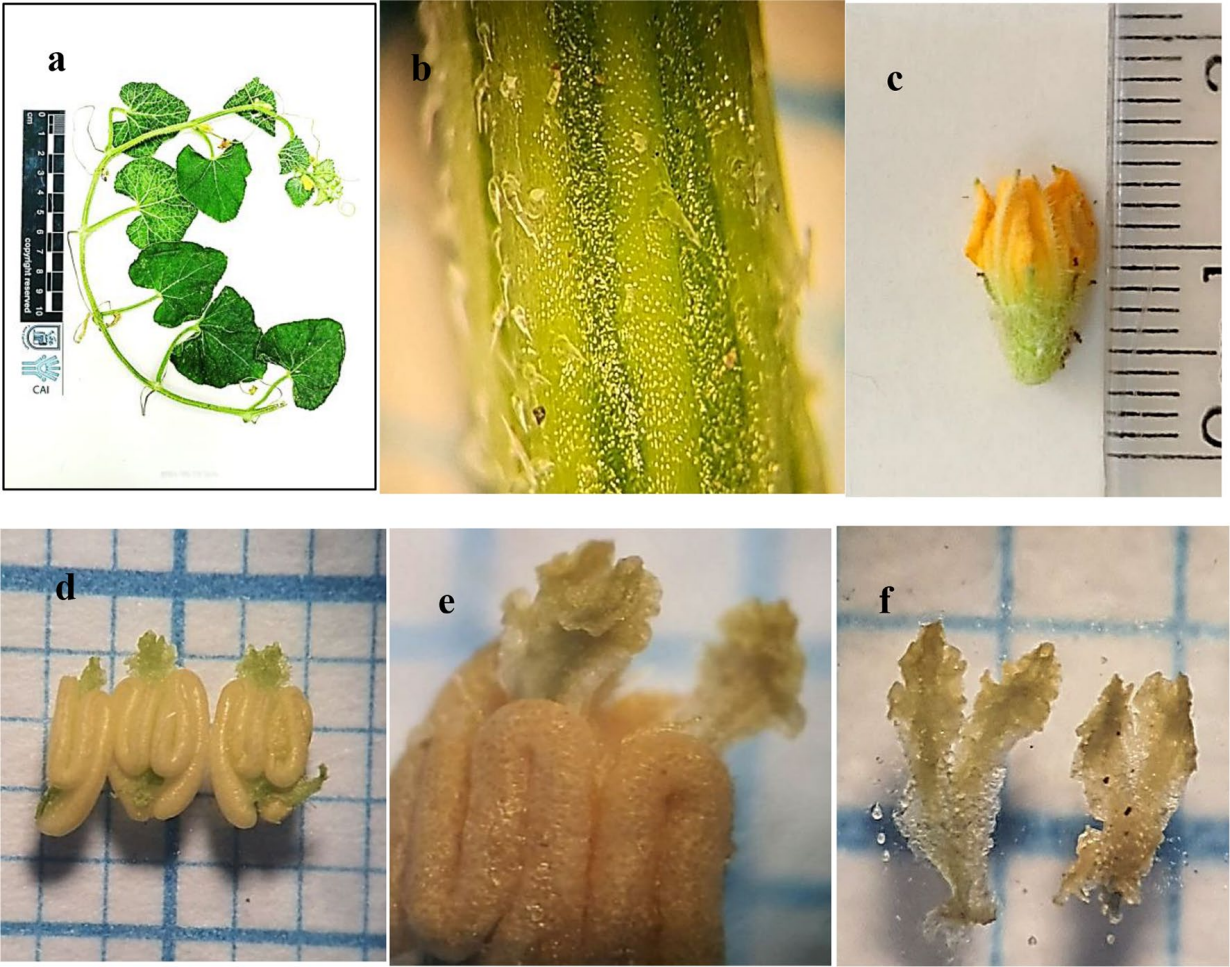
Morphological characters of *Cucumis melo var agrestis*; (**a**); twigs with leaves and tendrils, (**b-c**); Adaxial & abaxial surface of a leaf, (**d**); stem, (**e**); male flower, (**f**); androecium.

**Fig. 2:**
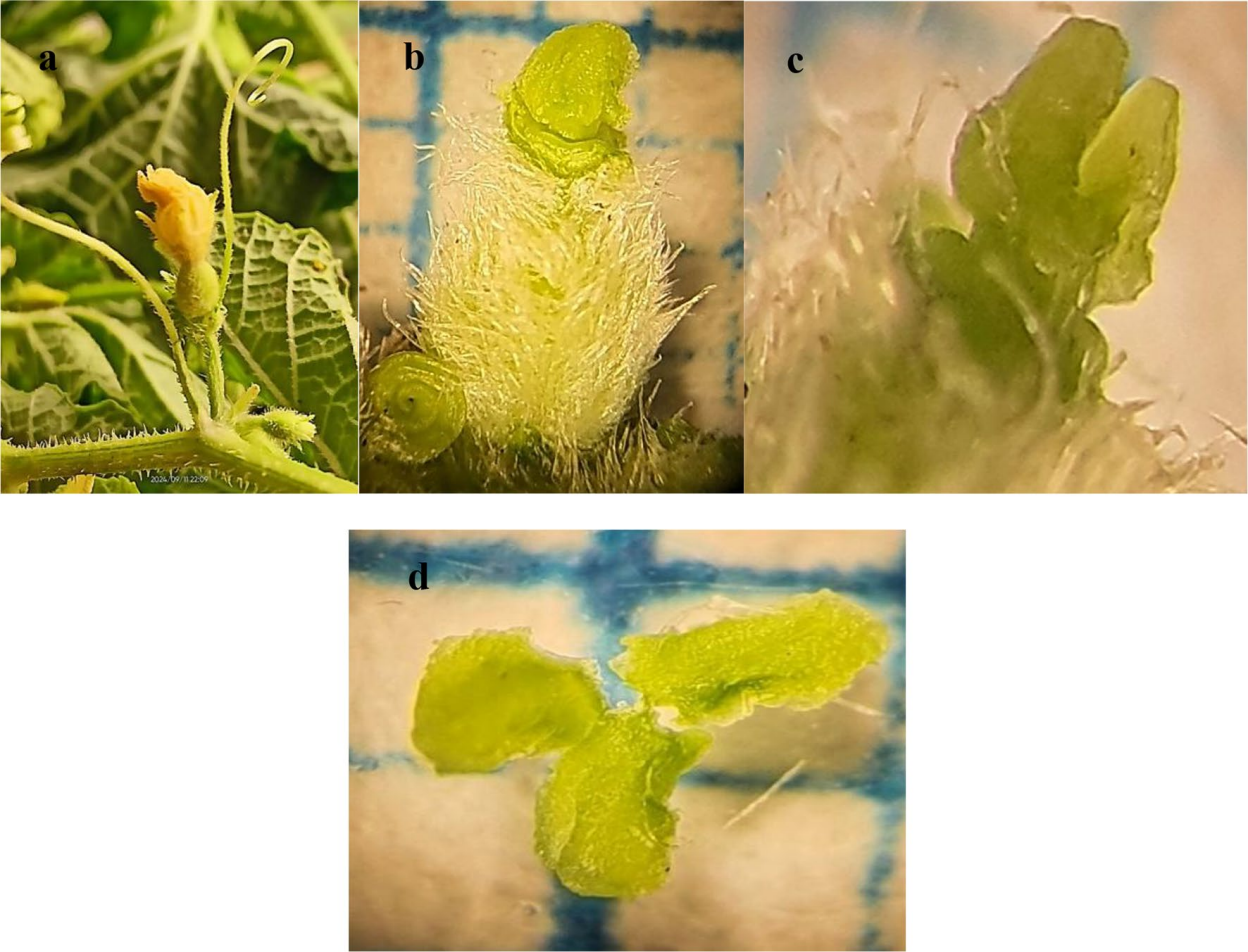
Morphological characters of *Cucumis melo var agrestis*; (**a-b**); connective, (**c**); bisexual flower, (**d**); gynoecium, (**e-f**); stigma.

Annual herb, prostrate with tap root, a single unbranched tendril as an anchor system with hairy surface; (4)6–14 cm long, stem pubescent, circular with ridges, internodes (3)5–7(9.5) cm long, leaves simple, palmate, obcordate- ovate, with long petiole; 2–4(6) cm long( Fig.1 a-b), leaf pubescent adaxially, scabrid abaxially with symmetric base; comparatively dense at midrib and veins **(**^69^**)**, with dentate margins(Fig.1 c). Plant andro-monoecious; bears yellow perfect flowers and male flowers on separate branches. Inflorescence axillary, pedunculate, consists of 1–3 flowers. The male inflorescences are larger in both number and size compared to the female inflorescences. Male flower pedicellate; pedicel (1.8)2.5–3 mm long, orangish yellow colour, calyx and corolla are joined forming a basal bell-shaped part, free-forming lobes at the mouth of sepals and petals(Fig. 1e). Calyx 3.5–5 mm long, densely hairy with five-pointed sepal lobes, corolla (4.5)5–8 mm long, densely hairy, gamopetalous, campanulate, contorted clockwise or anticlockwise with very short lobes; 0.5–1 mm long. Androecium (1-)2–2.2.2(3.2) mm long, 3 stamens, epipetalous, anthers yellow, curved with bright hairy interior margin. The connective extends beyond the polliniferous part, forming three pale green curly parts, each part divided into two extended segments 0.2–0.4 mm long (Figs. 1 e-f and Figs.2 a-b).

Yellow-orange bisexual flower pedicellate; pedicel 3–10 mm long and hairy, flower (2.5)4–4.5.5(5.5) mm long, ovary inferior, (1)1.5–3.5 mm long, woolly. At the apex of the ovary, calyx 2.4–3 mm long, with finger-shaped sepals that end with an obtuse apex, densely hairy. Corolla 4–4.5.5 (5.5) mm long, gamopetalous, contorted clockwise or anticlockwise, lobe slightly shorter than tube; tube 2–2.7 mm long, lobe 1.8–2.5 mm long, Androecium as in male flower. Style 0.2–0.4 mm long, smooth with circular outline, stigma green, three parts; each 0.8–1.8 × 0.6–0.8 mm long, oblong with wavy margins (Fig. 2, c-f).

**Fig. 3:**
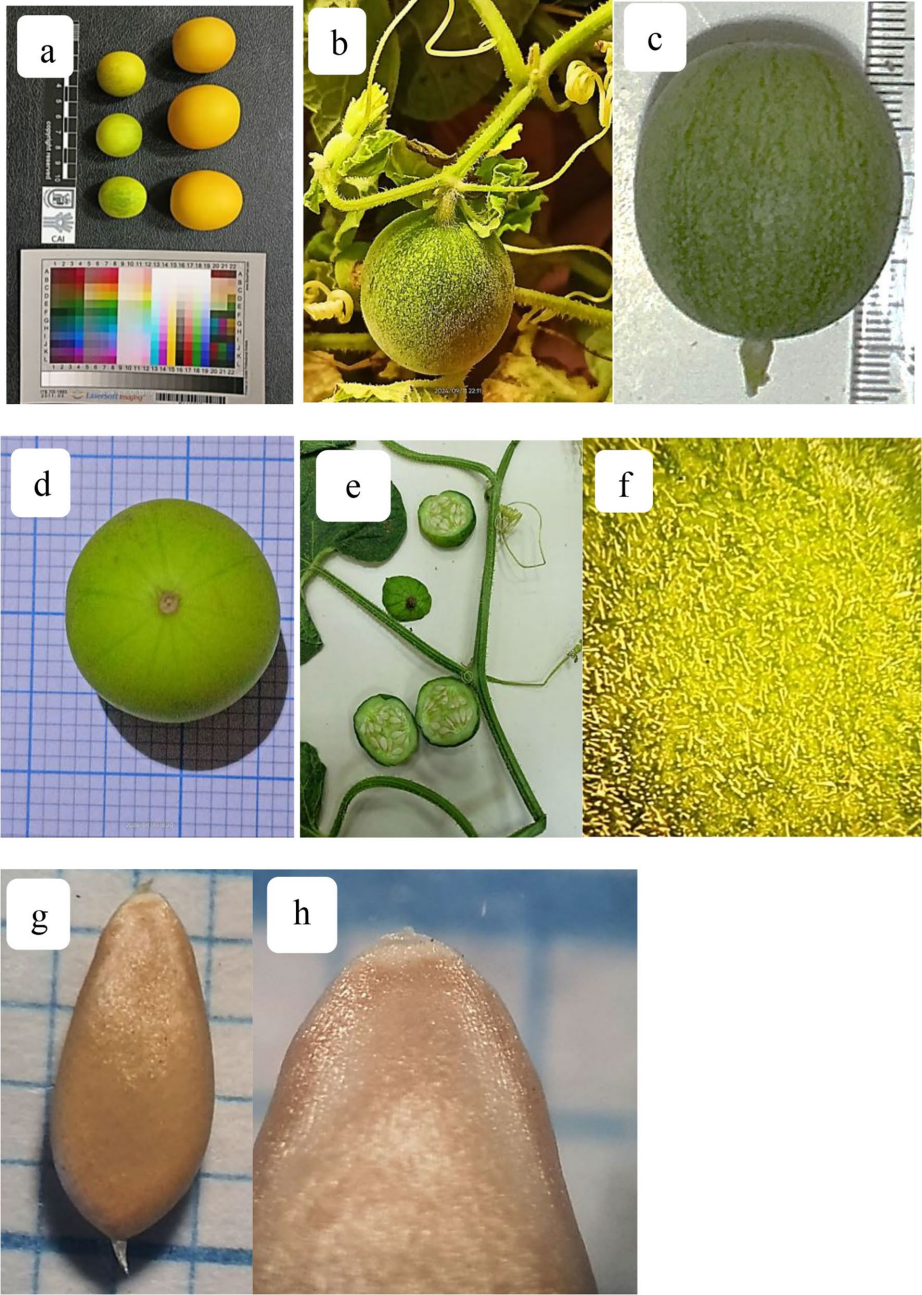
Morphological characters of *Cucumis melo var agrestis*; (**a-e**); fruit, (**a,d**); top view, (**b,c**) lateral view, (**e**); hairy surface of unripen fruit, (**f-g**); seed.

Fruit 2.2–3.2 × 2–2.5 cm, yellowish green- yellow, fleshy, globular to ellipsoid, berry-like, indehiscent, pubescent when young, turns glabrous at maturity, many-seeded. Seeds 4.2–4.5 × 1.8–2 mm, length/width ratio 2.1–2.3, which agrees with Cervantes &Martin Gomez (2018). Seed obovoid, pale cream, smooth, covered with a gelatinous sheath (an arillode jacket), hilum apical, at the opposite broad pole a transparent triangle (Fig. 3, a-g).

The current findings are supported by numerous earlier studies on *Cucumis melo* in across various fields; flowers may be unisexual, or dioecious, which agrees with Pandey and Mirsa^4^, Simpson^6^,and Rezk et al.^5^. Fruits pepo or berry in Cucurbitaceae (agree with^4,94^^,^^5^. Simple (unbranched) tendrils, in *Cucumis melo*^33^. Trichomes are distributed all over the leaf surface and are comparatively dense at the midrib, which agrees with Ali and Al-Hemaid^69^.

### Pollen

**Fig. 4:**
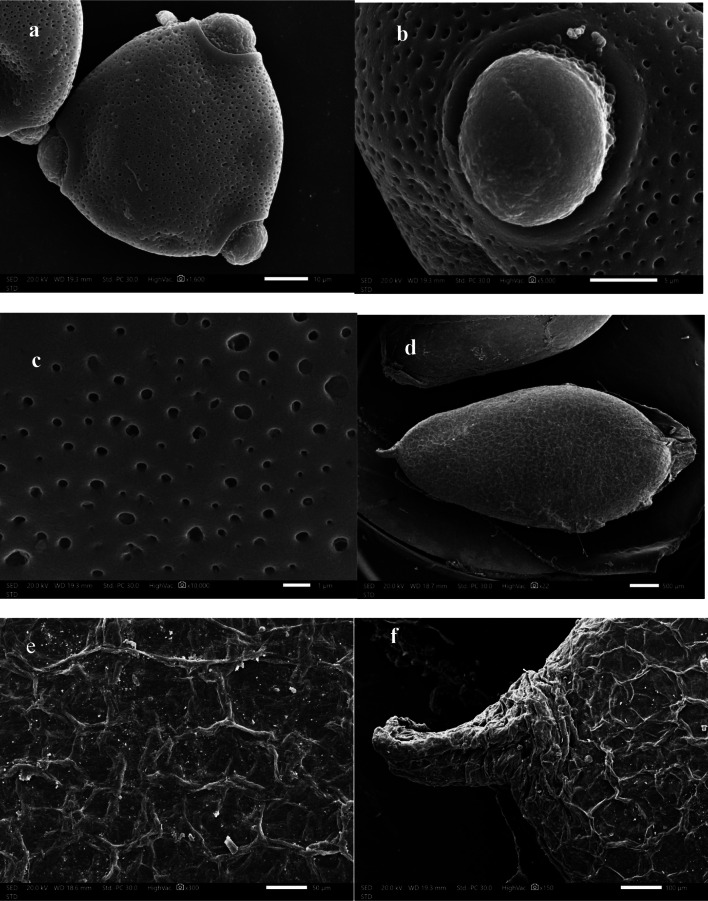
SEM photomicrographs of pollen grains and seed of *Cucumis melo var agrestis *; (**a-c**) pollen; (**a**); Equatorial view, (**b**); Aperture, c; exine ornamentation. (**d-f**); seed, (**d**); seed shape, (**e**); Magnified spermoderm surface, f; hilum.

Pollen grains monads, sub-triangular polar shape; 664–667 × 628–630 μm. Elliptical elliptic/oblate equatorial outline; 628–629 × 11–12 μm. Aperture is tri-zonoporate; pores circular with a distinct annulus. Ora is circular, convex, granulated ecoaperture and papillate base. Pollen grain texture is reticulate, foveolate ornamentation, which is in harmony with Teppner^95^, Perveen and Qaiser^45^, Abd–El Maksoud & Rania (2013), and Srivastava and Sharma^96^. (Fig.4,a-c).

Pollen grains of some species of the Cucurbitaceae family are eurypalynous, isopolar, radially symmetrical, and have a smooth surface in harmony with^96^. Pollen grains were 3 zonoporate in *Cucumis melo*. These results are consistent with the study of Lakshmi^97^and Hasson et al.^33^.

The size of the pollen grain was used depending on the length of the polar axis of the grain in the separation species. It was medium in species *cucumis melo*, Erdtman^98^,and Lakshmi^97^.

### Seed

Seed color is highly diagnostic and of systematic interest among taxa; seed, 58,500–58,515 × 25,500–25,510 μm, pale cream, obovoid with apical hilum, reticulate seed sculpture with hexagonal cells. The anticlinal wall is straight to slightly sinuous, raised, and smooth. The periclinal wall is flat-slightly concave (Fig. 4, d-f).

Seed morphology provides several characteristics that are potentially useful for species identification, phylogenetic inference, and character-state evolution^48–51^^,^^52^.

### Anatomy

#### Stem

**Fig. 5:**
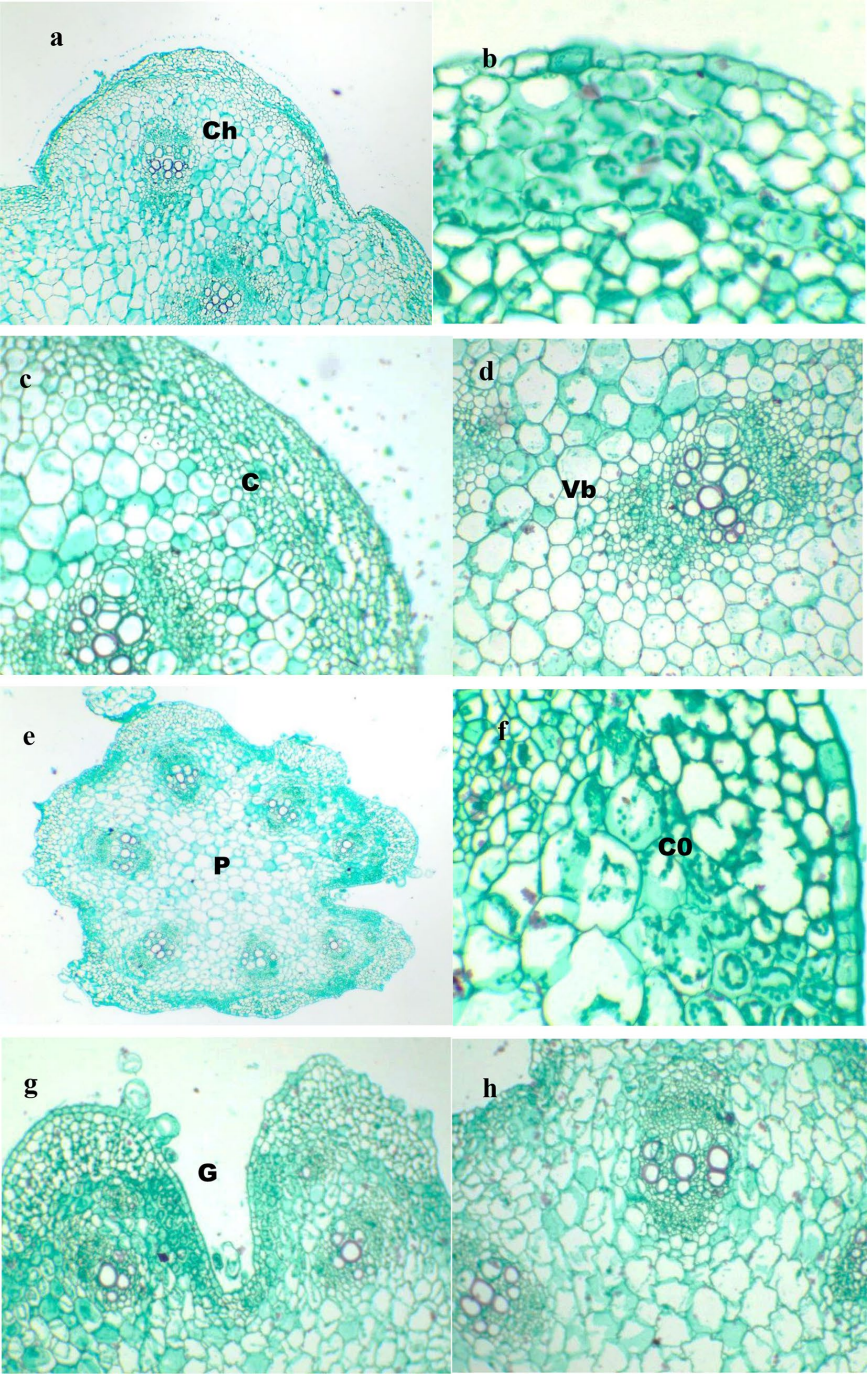
Transverse section of stem and petiole of *Cucumis melo var agrestis* .; (**a -d**) stem; (**e-h**) petiole. (ax300, bx3000, c-d x750, ex300, fx300, g-h x 750). Abbreviations, C; Cortex, Ch; Chlorenchyma, Co; collenchyma, G; groove, Vb; Vascular bundles.

Stem outline circular with ridges, epidermis is regular,  with tangentially elongated cells, with a thin cuticle layer except at ridges,  and isodiametric-tetragonal cells. Trichomes are glandular and nonglandular multicellular hairs; glandular trichomes are unicellular or multicellular, while nonglandular multicellular trichomes may have uniseriate or multiseriate trichomes (Fig. 5, a-c). 4–6 layers of tangentially-isodiametric elongated, irregular chlorenchyma cells at ridges below epidermal tissue, alternatingwith 7–14 tangentially elongated-isodiametric parenchyma cells. Cortical cells progressively enlarge and exhibit a reduction in plastid intensity with increasing proximity to the vascular bundles. Nine vascular bundles are arranged in two alternate rings: 5 outside and 4 inside. The outer ring has smaller bundles along its ridges. The vascular bundles bicollateral consist of 5–9(−11) layers of outer phloem; 1–2 layers of cambium, followed by xylem with 1–5 arches each with 3–4 vessels; vessels 7–8-gonals and inner phloem 5–9(11) layers. (Fig.5,d)The pith is composed of isodiametric to irregularly shaped parenchyma cells. Sand crystals are scattered in the cortex and pith cells  .

#### Petiole

The outline is oval with a groove along the narrow side. Epidermal tissue consists of one tangentially elongated, tubular cell coated with a thin cuticle. Glandular and multicellular trichomes are present on the epidermis; the multicellular trichomes occur in two forms: uniseriate and multiseriate. Beneath the epidermal layer, collenchyma and parenchyma tissues are arranged in an alternating pattern. The number of collenchyma cushions is consistent with the number of protrusions. The cortex consists of 4–7 layers of collenchyma composed of radially elongated cells, followed by 4–6 layers of parenchyma made up of isodiametric cells. The vascular system comprises nine bicollateral vascular bundles, seven larger and two smaller ones arranged within the ear zone. Each vascular bundle consists of 8–10 layers of outer phloem, 1–2 cambium layers, xylem 2–4 xylem arches, each arch has 1–3 vessels,  with vessels mainly 7-gonal cells, followed by 5–7 layers of inner phloem. Pith isodiametric-irregular thin-walled parenchymatous cells, 12–18 layers (Fig. 5, e-h).

The occurrence of vascular bundles in the petiole of *Cucumis melo* is almost a closed crescent or circle^99^. The importance of epidermal characters of leaves in angiosperms has been reviewed by several authors^100–107^; two basic hair types (glandular and non-glandular) and many variations among cucurbits species^108,109^. The presence of trichomes on the underside of leaves,which is widely recognized as having major taxonomic relevance, is widely recognized in the Cucurbitaceae^66^.

Among various plant species, including cucurbits, anatomical characteristics of the petiole, leaf lamina, and the number of vascular bundles in different plant parts have proven valuable for distinguishing and delimiting species within the same genus or family^64,110–115^.

#### Leaf

**Fig. 6:**
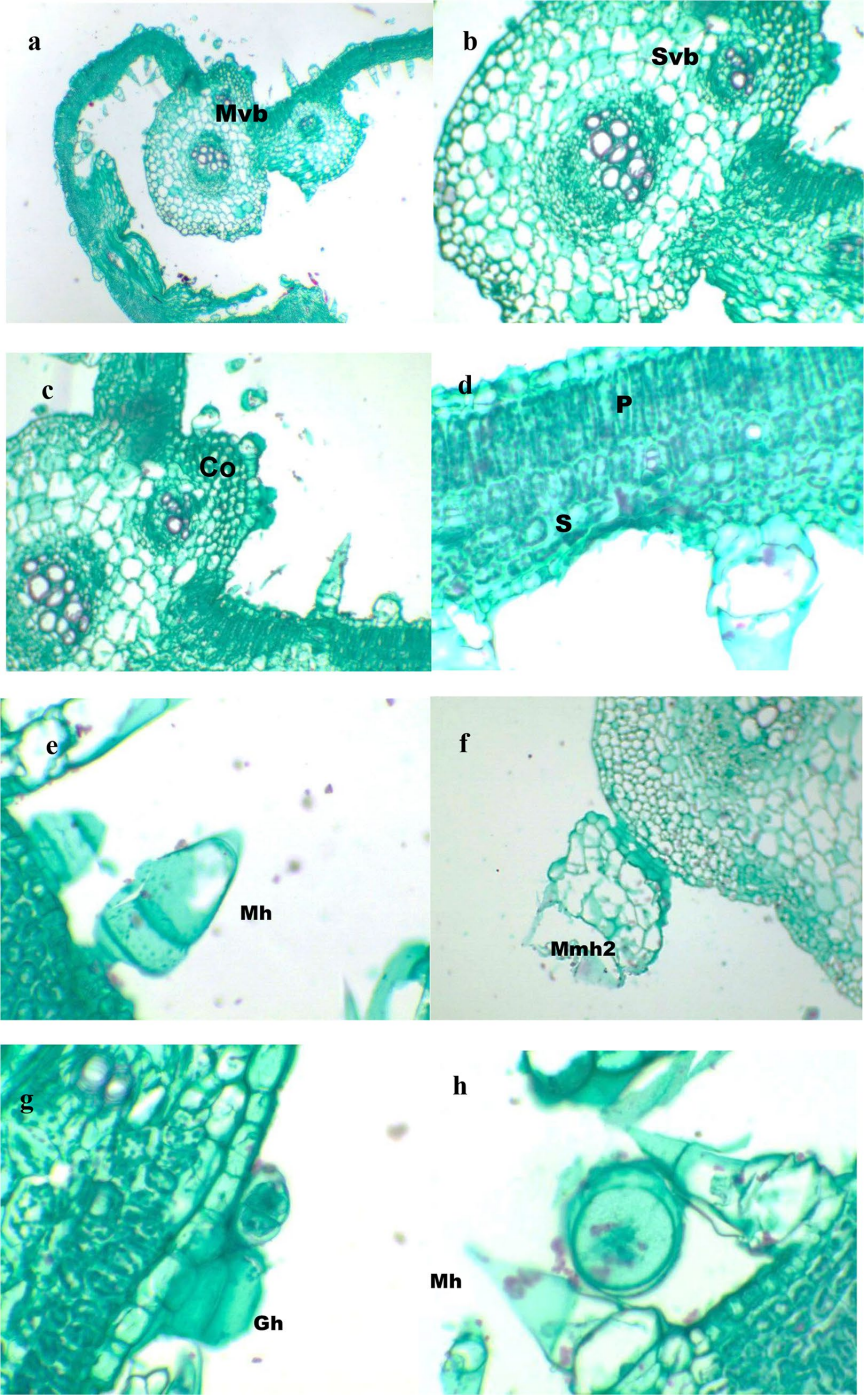
Transverse section of leaf of *Cucumis melo var agrestis* .; (**a -d**) leaf; (**e-h**) trichomes. (ax300, b-d x750, e-h x 300). Abbreviations: Co; Collenchyma, Gh; Glandular hair, Mh; Multicellular hair, Mmh; multicellular multiseriate, Mvb; Main vascular bundle, P; Palisade tissue, S; Spongy tissue, Svb; small vascular bundles.

 The U-shaped midrib region has a single layer of tangentially elongated, 4–5-gonal upper epidermal cells with a thin cuticle layer. Hairs on both surfaces are glandular and multicellular (Fig. 6, e-h). 5–6 layers of collenchyma tissue present in the median line of the upper surface of the leaf midrib are isodiametric-radially elongated cells. Followed by 1–2 layers of tangentially elongated parenchyma cells. 2–3 bundles are present in the midrib region; the uppermost bundle is smaller, while the lowest is the largest. The outer one has 4–5 outer phloem followed by 1–2 cambium layers, xylem with 3–4 arches each with 1–2 vessels, and inner phloem has 4–5 layers. The biggest with 8–10 outer phloem, 1–2 cambium layers, xylem 4–5 arches each with 1–3 vessels, inner phloem with 9–10(−11) layers. 8–9 layers of isodiametric-radially elongated parenchyma cells followed by 2–3 layers of isodiametric-radially collenchyma cells. Lower epidermis isodiametric-radially elongated cells coated with a cuticle (Fig. 6, a-c).

Wing region: upper epidermis of isodiametric-radially elongated cells.  The mesophyll consists of 2–3 palisade layers and 3–4 spongy layers. Spongy cells are irregularly isodiametric,  with a lower epidermis of tangentially-radially elongated cells (Fig. 6, d).

The number of vascular bundles in the petiole of cucurbits members varied from 7 to 9^67^. Bicollateral bundles, which are composed of both internal and external phloem^116,117^, have been observed in various plant families, such as Apocynaceae, Convolvulaceae, Cucurbitaceae, Solanaceae, and Asteraceae^118,119^. Stem vascular bundles in most species of *Colocynthis*,* Cucumis*,* Cucurbita*,* Citrullus*, and *Luffa* are arranged in two rings^108^, which agrees with the present results.

The genus *Cucumis* has three bundles in the midrib region of the leaf, arranged in a straight line from above downwards; the uppermost bundle is the smallest, while the lowest is the largest (agree with^108^. The thickness of the palisade parenchyma varies across species. The spongy parenchyma has two to six layers of cells. This observation is consistent with the findings of Ismail et al.^108^, and Okoli^120^**)**, Edeoga and Okoli^121^,and Edeoga and Okoli^122^.

#### Phytochemical screening

The preliminary qualitative phytochemical screening of 70% ethanol extracts from the leaves and fruits of *Cucumis melo* var. *agrestis*(Table 1) revealed the presence of tannins, alkaloids, saponins, glycosides, phenolics, flavonoids, terpenoids, steroids, fatty acids, and coumarins. Conversely, anthocyanins and emodins were absent. The fruits possess digestive, stomachic, vermifuge, febrifuge properties, analgesic, antioxidant, antibacterial, and anti-inflammatory properties . Sharma^123^ reported the presence of various phytoconstituents in the fruit extract of (*Cucumis melo* var. *agrestis*). Consistent with their findings, our results also demonstrated that the fruit extract contains multiple phytochemicals, including glycosides, alkaloids, phenols, flavonoids, saponins, tannins, proteins, amino acids, and carbohydrates.

**Table 1:** Phytochemical screening of 70% ethanol extracts of leaves and fruits of *Cucumis melo*var.*agrestis.*

Chemical constituents	*Cucumis melo*var.*agrestis.*	
	Ethanol 70%	
	Leaves	Fruits
Tannins	++	+++
Alkaloids	+	++
Saponins	+++	+
Glycosides	+++	++
Anthocyanins	-	-
Emodins	-	-
Phenolics	+++	+
Flavonoids	+++	+
Terpenoids	+++	++
Steroids	+++	++
Fatty acids	+	++
Coumarins	+++	++

(+++) high presence, (++) moderate presence, (+) low presence, and (−) absence of the tested phytochemicals.

The results agree with the findings of Yadav et al.^80^, who reported the presence of alkaloids, flavonoids, phenolic compounds, saponins, steroids, carbohydrates, cardiac glycosides, and tannins in the 70% ethanolic extracts of *Cucumis melo* var. *agrestis* leaves and seeds, as determined through phytochemical screening. Various researchers^124^ showed that polyphenols, including flavonoids and their derivatives, exhibit outstanding antioxidant properties because of their optimal structural chemistry for scavenging free radicals^125^. Due to their involvement as electron or hydrogen donors to free radicals, they can also serve as reducing agents and free radical quenchers.

Preliminary phytochemical analysis conducted by Gopalasatheeskumar and Kalaichelvan^126^ revealed the presence of alkaloids, flavonoids, tannins, carbohydrates, saponins, glycosides, proteins, and amino acids in most extracts. However, the seed, fruit, flower, and root extracts of *Cucumis melo* var. *agrestis* were found to lack tannins, and the flower extract was also devoid of saponins. Phytochemical screening plays a critical role in evaluating antioxidant activity, as various phytoconstituents contribute differently to the antioxidant potential of plant extracts.

#### Total phenolic and flavonoid content

Determination of total phenolic and flavonoid contents in the ethanol extract revealed that the leaves contained higher levels than the fruits. The leaves extract showed 55.71 ± 2.816 mg GAE/g of total phenolics and 9.013 ± 0.421 mg QE/g of total flavonoids, whereas the fruits extract contained 35.15 ± 2.48 mg GAE/g and 6.447 ± 0.255 mg QE/g, respectively (Table 2).

**Table 2:** Total phenolic compounds and total flavonoids content of leaves and fruits extracted by Ethanol 70% from Cucumis melo var. agrestis.

Ethanol 70% extract			
Leaves		Fruits	
**Total phenolic (mg GAE/g)** ^*^	**Total flavonoid (mg QE/g)** ^**^	**Total phenolic (mg GAE/g)** ^*^	**Total flavonoid (mg QE/g)** ^**^
55.71 ± 2.816	9.013 ± 0.42	35.15 ± 2.48	6.447 ± 0.26

*:(mg GAE/g of extracts) gallic acid equivalents per g **: (mg QE/g of extracts) quercetin equivalent per g.Each value is expressed as the mean ± SD(*n* = 3).

The results are in agreement with the findings of Gopalasatheeskumar and Kalaichelvan^126^, who reported that the leaf and fruit extracts of *Cucumis melo* var. *agrestis* contain high levels of total phenolic content—approximately 17.82 mg/g and 19.82 mg/g, respectively (expressed as gallic acid equivalents). They also demonstrated notable levels of total flavonoid content 12.93 mg/g and 18.39 mg/g, respectively (expressed as quercetin equivalents).

Similarly, these findings are in agreement with Gopalasatheeskumar et al.^12^, who identified various phytochemicals in *C. melo* var. *agrestis* extracts, including alkaloids, flavonoids, tannins, carbohydrates, saponins, glycosides, proteins, and amino acids. Their quantitative analysis confirmed a total phenolic content of 77.82 mg/g (GAE) and a total flavonoid content of 30.06 mg/g, also calculated as gallic acid equivalents.

#### Antioxidant activity

To obtain a comprehensive evaluation of the antioxidant potential of the extracts, multiple antioxidant assays were employed, including DPPH, KMnO₄, methylene blue, and DCPIP methods. Each assay is based on a different reaction mechanism, such as radical scavenging ability or reducing power, which provides a broader assessment of antioxidant activity. Therefore, the use of several assays allows a more reliable and complete evaluation of the antioxidant properties of the plant extracts.

In the present study, four different antioxidant assays were employed to assess the antioxidant activity of successive ethanol extracts from the leaves and fruits of *Cucumis melo* var. *agrestis*. These included one radical-based method (DPPH radical assay) and three non-radical-based methods: potassium permanganate (KMnO₄) assay, methylene blue assay, and the DCPIP (2,6-dichlorophenol-indophenol) reduction assay.

#### KMnO₄ method

The reaction with potassium permanganate (KMnO₄) involves redox processes, in which antioxidant compounds undergo oxidation, typically at unsaturated bonds, leading to their conversion into diol molecules^127^. The antioxidant activity in this assay is evaluated by the ability of phytochemicals present in the extracts to reduce KMnO₄. A higher degree of KMnO₄ reduction indicates greater antioxidant potential of the tested extracts^127^.

In the present study, the leaf and fruit extracts of *Cucumis melo* var. *agrestis* demonstrated reducing activity in the KMnO₄ assay, confirming the presence of antioxidant constituents (Table 3).

**Table 3:** Antioxidant activity of 70% ethanol extracts of *Cucumis melo* var. *agrestis* using the KMnO₄ assay.

KMnO₄ assay (70% ethanol)			
Concentration (µg/mL)	Leaves	Fruits	Ascorbic acid
1000	75.75 ± 0.59 ^*^	66.04 ± 0.62 ^*^	55.11 ± 0.23
500	73.27 ± 0.74 ^*^	61.7 ± 0.25 ^*^	52.42 ± 0.46
400	63.39 ± 2.6 ^*^	60.39 ± 0.16 ^*^	51.70 ± 0.58
200	57.93 ± 0.63 ^*^	51.29 ± 0.12 ^*^	47.93 ± 1.3
100	42.02 ± 1.07 ^*^	32.83 ± 1.13 ^*^	19.87 ± 0.35
50	33.25 ± 0.7 ^*^	28.81 ± 0.56 ^*^	18.91 ± 0.3
25	30.28 ± 0.58 ^*^	26.21 ± 0.76 ^*^	16.97 ± 0.47
IC_50 (µg/mL)_	145.90 ± 0.02	169.89 ± 0.34	205.3 ± 0.31

Values are expressed as mean ± SD (*n* = 3). * indicates a statistically significant difference compared with the ascorbic acid control (*p* < 0.05) using one-way ANOVA.

#### Methylene blue (MB) method

The methylene blue (MB) assay is a well-established method for evaluating the antioxidant capacity of various samples. This assay is based on the reduction of methylene blue by antioxidants present in the sample, which leads to a decrease in absorbance measured spectrophotometrically. As shown in (Table 4), the ethanol extracts from the leaves and fruits of *Cucumis melo* var. *agrestis* and ascorbic acid were tested for their antioxidant activity against methylene blue. The results indicate that the leaf extract exhibited the highest inhibition compared to the fruit extract and ascorbic acid, with ascorbic acid showing approximately 80.51 ± 0.11% inhibition at 1000 µg/ml. Moreover, the leaf extract demonstrated the lowest IC₅₀ value, approximately 102.2%, indicating stronger antioxidant activity than the fruit extract and ascorbic acid.

**Table 4:** Antioxidant activity of 70% ethanol extracts of *Cucumis melo* var. *agrestis* using the methylene blue assay.

Methylene blue assay (70% ethanol)			
Concentration (µg/mL)	Leaves	Fruits	Ascorbic acid
1000	80.51 ± 0.11^*^	77.41 ± 0.16 ^*^	75.01 ± 0.59
500	79.55 ± 0.3^*^	72.67 ± 0.26 ^*^	70.44 ± 0.43
400	75.44 ± 0.4^*^	73.61 ± 0.43 ^*^	69.26 ± 0.16
200	72.64 ± 0.28 ^*^	70.50 ± 0.27 ^*^	68 ± 0.39
100	69.30 ± 0.11 ^*^	67.53 ± 0.29	66.21 ± 0.035
50	68.40 ± 0.14 ^*^	66.51 ± 0.33^*^	63.53 ± 0.25
25	44.00 ± 0.44 ^*^	33.41 ± 0.19^*^	29.22 ± 0.13
IC_50_ (µg/mL)	102.2 ± 0.05	106.2 ± 0.03	110.49 ± 0.26

Values are expressed as mean ± SD (*n* = 3). * indicates a statistically significant difference compared with the ascorbic acid control at *p* < 0.05 according to one-way ANOVA.

Although the MB and KMnO₄ methods have not been previously applied to *C. melo* var. *agrestis* extracts, the plant’s high phenolic and flavonoid content strongly suggests significant antioxidant potential comparable to ascorbic acid. High levels of phenolics and flavonoids in *C. melo* var. *agrestis*extracts are likely responsible for the notable methylene blue reduction observed, supporting findings reported by Arora et al.^128^.

#### DCPIP scavenging assay

The DCPIP titrimetric method has been widely employed as an indicator of vitamin C content, particularly for the quantification of ascorbic acid. Although it is commonly believed that the DCPIP titrimetric approach is effective primarily for ascorbic acid and is limited to colorful fruits^129^, 2,6-dichlorophenolindophenol (DCPIP) itself is a well-known redox indicator dye. It functions as an oxidizing agent through a rapid electron transfer process with antioxidants^130,131^.

The present study’s results, summarized in Table 5, demonstrate the antioxidant activity of ethanol extracts from the leaves and fruits of *Cucumis melo* var. *agrestis*, alongside ascorbic acid, against DCPIP. The leaf extract exhibited the highest inhibition compared to the fruit extract and ascorbic acid, with ascorbic acid showing approximately 82.94 ± 0.58% inhibition at 1000 µg/ml. Furthermore, the leaf extract displayed the lowest IC₅₀ value, approximately 104.6%, indicating stronger antioxidant potential when compared with the fruit extract and natural ascorbic acid.

**Table 5:** Antioxidant activity of 70% ethanol extracts of *Cucumis melo* var. *agrestis* using the DCPIP assay.

DCPIP assay (70% ethanol)			
Concentration (µg/mL)	Leaves	Fruits	Ascorbic acid
1000	82.94 ± 0.58 ^*^	76.40 ± 0.13 ^*^	66.44 ± 0.29
500	75.98 ± 0.69 ^*^	72.34 ± 0.31 ^*^	66.11 ± 0.1
400	73.33 ± 0.1 ^*^	70.35 ± 0.19 ^*^	65.04 ± 0.39
200	70.69 ± 0.66 ^*^	69.51 ± 0.3 ^*^	63.65 ± 0.69
100	68.51 ± 0.28 ^*^	64.37 ± 0.12 ^*^	62.09 ± 0.72
50	63.17 ± 1.5 ^*^	61.14 ± 0.22 ^*^	55.97 ± 2.6
25	49.30 ± 0.05 ^*^	47.69 ± 0.45 ^*^	33.24 ± 0.23
IC_50_ (µg/mL)	104.6 ± 0.62	108.3 ± 0.28	117.7 ± 0.06

Values are expressed as mean ± SD (*n* = 3). * indicates a statistically significant difference compared with the ascorbic acid control at *p* < 0.05 according to one-way ANOVA.

#### DPPH assay

The superiority of phenolic content and antioxidant activity in leaves compared to fruits can be attributed to the direct exposure of leaves to light and UV radiation, which induces the generation of reactive oxygen species (ROS). This activates the shikimate/phenylpropanoid pathway and enhances the accumulation of phenolics in vacuoles as a defensive mechanism. Moreover, differences in plant organs (leaf vs. pulp/peel) and the wild nature of certain genotypes explain the variation in results reported among different studies^132^^,^^133,134^.

The data presented in Table 6 illustrate the antioxidant activity of ethanol extracts from the leaves and fruits of *Cucumis melo* var. *agrestis*, along with ascorbic acid, as assessed by the DPPH radical scavenging assay. The results revealed the highest percentage of inhibition in the leaf extract and ascorbic acid when compared to the fruit extract, with values of 89.2 ± 0.003% and 92.3 ± 0.003%, respectively, at a concentration of 1000 µg/ml.

**Table 6:** Antioxidant activity of 70% ethanol extracts of *Cucumis melo* var. *agrestis* using the DPPH assay.

DPPH assay (70% ethanol)			
Concentration (µg/mL)	Leaves	Fruits	Ascorbic acid
1000	89.2 ± 0.003^*^	84.6 ± 0.005^*^	92.3 ± 0.003
500	80.7 ± 0.003^*^	78.8 ± 0.004^*^	88.5 ± 0.003
400	71.2 ± 0.002^*^	70.0 ± 0.002^*^	80.6 ± 0.002
200	62.4 ± 0.004^*^	60.9 ± 0.005^*^	73.6 ± 0.003
100	53.5 ± 0.003^*^	52.0 ± 0.003^*^	65.6 ± 0.003
50	44.8 ± 0.005^*^	43.3 ± 0.003^*^	56.9 ± 0.002
25	36.1 ± 0.005^*^	34.7 ± 0.003^*^	51.6 ± 0.003
IC_50_ (µg/mL)	126.6 ± 0.03	130.14 ± 0.23	104.51

Values are expressed as mean ± SD (*n* = 3). * indicates a statistically significant difference compared with the ascorbic acid control at *p* < 0.05 according to one-way ANOVA.

These findings are supported by Vidya and Kalaivani^135^ and Gopalasatheeskumar and Kalaichelvan and^126^, who reported that the ethanolic fruit extract of *C. melo* var. *agrestis* exhibited 64.46% DPPH radical scavenging activity at tested concentrations, demonstrating a dose-dependent response. Interestingly, their study indicated that the IC₅₀ value of the fruit extract was lower than that of other plant parts (e.g., leaves, stem, seeds), suggesting comparatively higher antioxidant potential in fruits under their experimental conditions.

The present findings provide valuable information regarding the antioxidant potential of *Cucumis melo* var. *agrestis* extracts. The observed antioxidant activity may be associated with the high phenolic and flavonoid contents detected in the extracts. These results contribute to the growing evidence that plant-derived phenolic compounds play an important role in scavenging free radicals and may have potential applications in pharmaceutical and nutraceutical fields.

#### High-performance liquid chromatography (HPLC)

The bioactive phenolic components in the methanolic extract of *Cucumis melo* var. *agrestis*leaves were identified and quantified using High-Performance Liquid Chromatography (HPLC). The identification was performed by comparing the HPLC peaks of the sample with those of known standards based on peak area, retention time, and spectral characteristics, following the method described by Mehmood et al.^136^.

#### Phenolic compounds profile of leaves *Cucumis melo var. agrestis* ethanolic extract

**Fig. 7:**
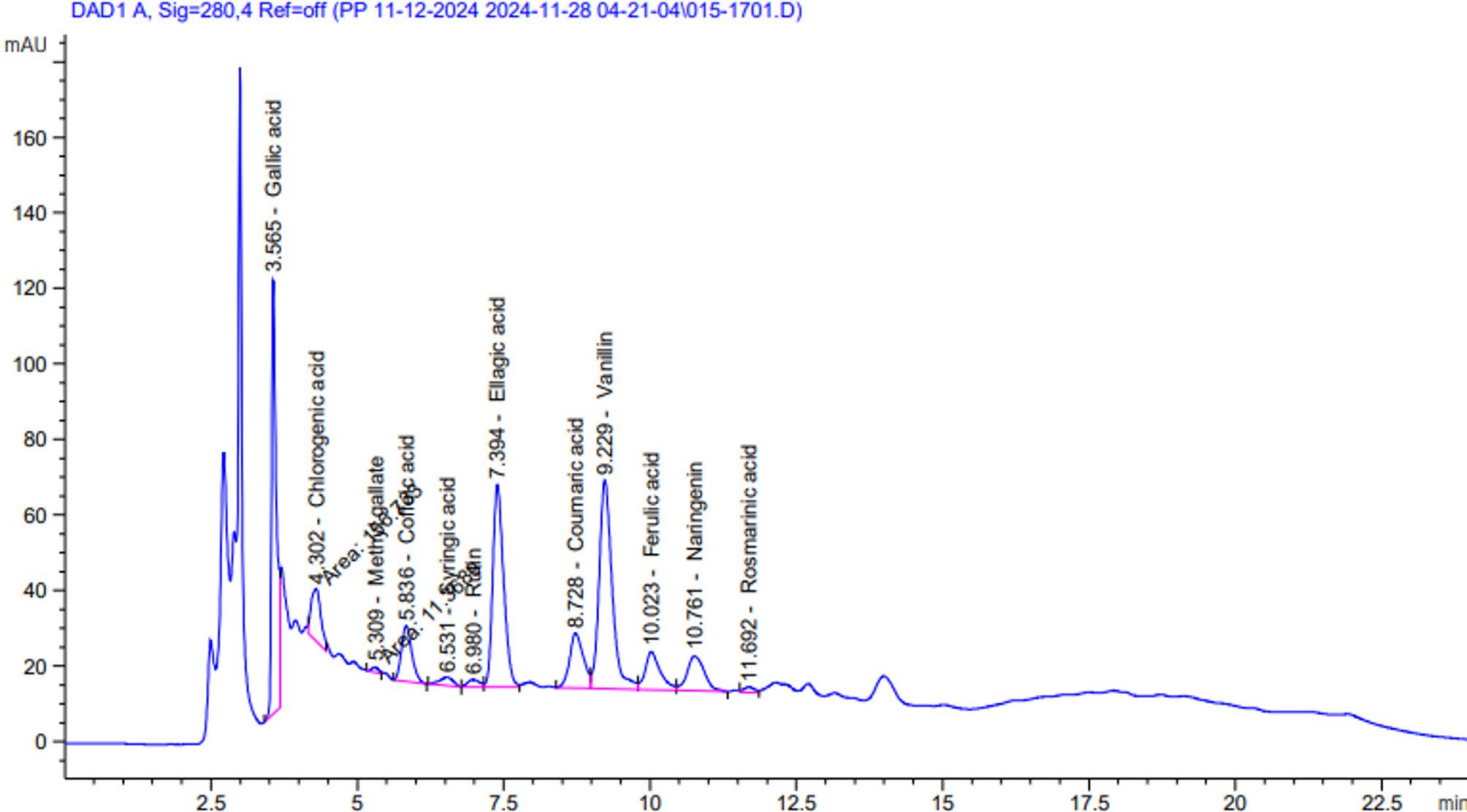
HPLC chromatogram of leaves *Cucumis melo var. agrestis* ethanolic extract.

HPLC analysis of the ethanolic leaf extract revealed the presence of multiple phenolic compounds. As shown in Table 7; Fig. 7, twelve phenolic compounds were identified and classified in Table 7 according to their peak area. Gallic acid was found in the highest concentration, measuring 2380.60 µg/g of extract.

**Table 7:** Phenolic compounds profile of leaves *Cucumis melo* var. *agrestis* ethanolic extract.

Leaves				
Compounds	Area	Chemical structure	Conc. (µg/g)	Biological activity
Gallic acid	650.28		2380.60	Antioxidant, Antimicrobial, Anti-inflammatory, Anticancer Activity^137^
Chlorogenic acid	156.73		1092.01	Antioxidant, Anti inflammatory, Antimicrobial, Antihypertensive, Antidiabetic Activity^138^.
Methyl gallate	11.37		31.81	Antioxidant, Antimicrobial, Anti-inflammatory, Neuroprotective, Anticancer, Cardioprotective Activity^139^
Coffeic acid	182.41		467.91	Antioxidant, Anti-inflammatory, Neuroprotective, Anticancer, Cardioprotective Activity^140^
Syringic acid	35.03		103.01	Antioxidant, Anti-inflammatory, Anticancer, Cardioprotectie Activity^141^
Rutin	26.83		200.82	Antioxidant, hepatoprotective, nephroprotective, neuroprotective, anti-inflammatory, antimicrobial, antidiabetic, antitumor properties^142^
Ellagic acid	689.49		3502.15	Antioxidant, Anti-inflammatory, Neuroprotective, Anticancer, Cardioprotectie,, Antimicrobial Activity^143^
Coumaric acid	235.18		422.61	Antioxidant, anti-inflammatory, analgesic, and antimicrobia^144^
Vanillin	850.74		1542.80	Antimicrobial Activity, Anti-inflammatory, Anticancer Activity^145^
Ferulic acid	180.82		524.82	Antioxidant, anti-inflammatory, antiviral, antiallergic, antimicrobial, antithrombotic, anticarcinogenic^146^
Naringenin	189.08		872.55	Antidiabetic, anticancer, antimicrobial ^147^.
Rosmarinic acid	20.19		98.06	Antioxidant, Anti-inflammatory, Antimicrobial, Antiviral. Neuroprotective, Anticancer Activity^148^

#### Phenolic compounds profile of fruits *Cucumis melo var. agrestis* ethanolic extract

**Fig. 8:**
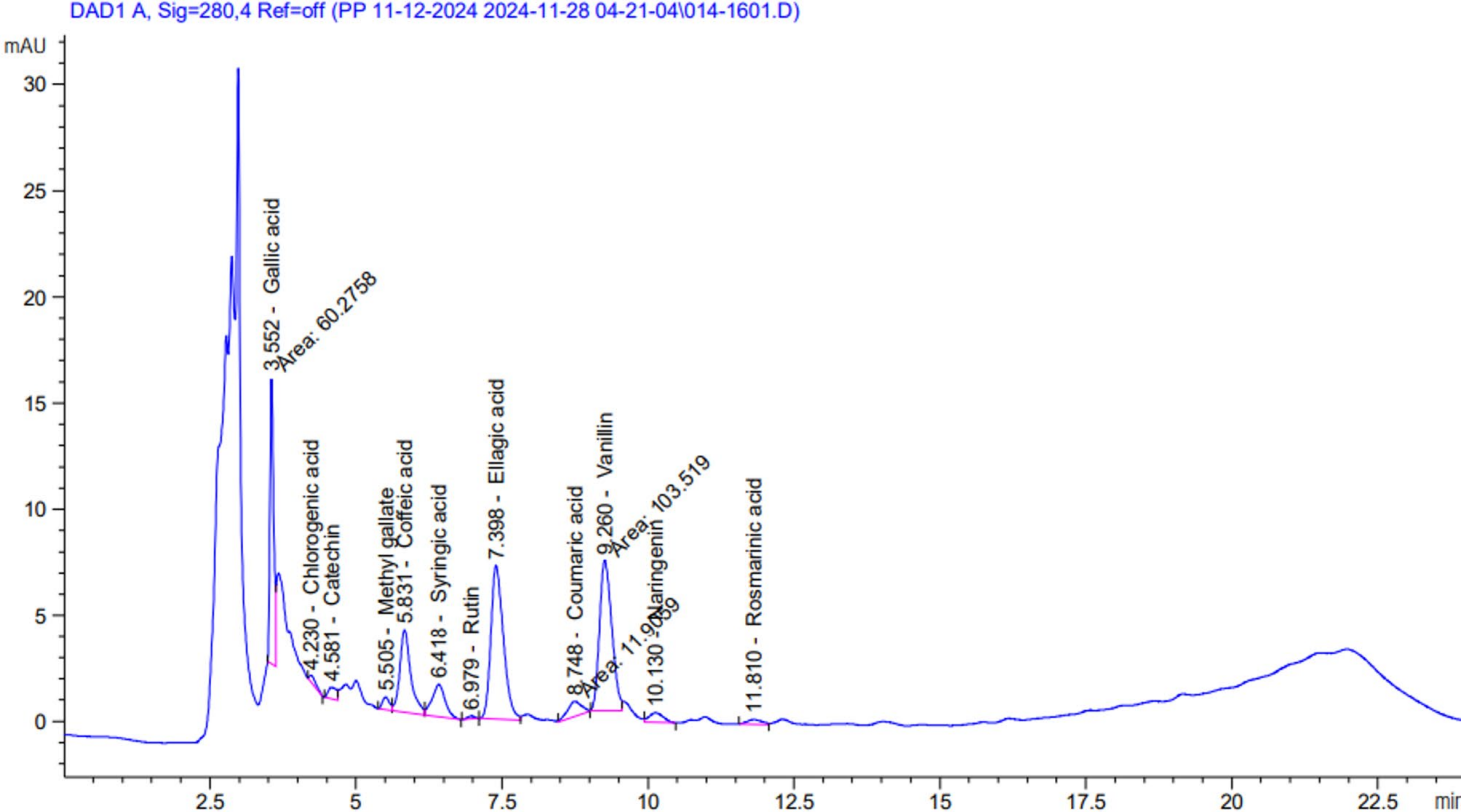
HPLC chromatogram of fruits *Cucumis melo var. agrestis* ethanolic extract.

The HPLC analysis of the ethanolic extract revealed the presence of various phenolic compounds. As presented in Table 8; Fig. 8, twelve phenolic compounds were identified in the fruit extract. Among these, ellagic acid was found at the highest concentration, measuring 551.15 µg/g.

**Table 8:** Phenolic compounds profile of fruits *Cucumis melo var. agrestis* ethanolic extract.

Fruits				
Compounds	Area	Chemical structure	Conc. (µg/g)	Biological activity
Gallic acid	60.28		220.66	Antioxidant, Antimicrobial, Anti-inflammatory, Anticancer Activity^137^
Chlorogenic acid	2.51		17.51	Antioxidant, Anti inflammatory, Antimicrobial, Antihypertensive, Antidiabetic Activity^138^
Catechin	5.52		59.27	Antioxidant, Anti-inflammatory, Antimicrobial, Antihypertensive, Antidiabetic Activity^149^
Methyl gallate	5.04		14.10	Antioxidant, Antimicrobial, Anti-inflammatory, Neuroprotective, Anticancer, Cardioprotective Activity^139^
Coffeic acid	50.71		130.09	Antioxidant, Anti-inflammatory, Neuroprotective, Anticancer, Cardioprotective Activity^140^
Syringic acid	23.44		68.94	Antioxidant, Anti-inflammatory, Anticancer, Cardioprotectie Activity^141^
Rutin	1.17		8.74	Antioxidant, hepatoprotective, nephroprotective, neuroprotective, anti-inflammatory, antimicrobial, antidiabetic, antitumor properties^142^
Ellagic acid	108.51		551.15	Antioxidant, Anti-inflammatory, Neuroprotective, Anticancer, Cardioprotectie,, Antimicrobial Activity^143^
Coumaric acid	11.91		21.39	Antioxidant, anti-inflammatory, analgesic, and antimicrobia^144^
Vanillin	103.52		187.73	Antimicrobial Activity, Anti-inflammatory, Anticancer Activity^145^
Naringenin	7.62		35.18	Antidiabetic, anticancer, antimicrobial ^147^
Rosmarinic acid	3.73		18.14	Antioxidant, Anti-inflammatory, Antimicrobial, Antiviral. Neuroprotective, Anticancer Activity^148^

A clear difference was observed between the phenolic profiles of leaves and fruits. Leaves of *Cucumis melo var. agrestis* showed higher concentrations of gallic acid, consistent with their need for enhanced antioxidant protection during photosynthesis and exposure to UV-induced ROS. In contrast, fruits accumulate phenolics such as chlorogenic and ellagic acids, which are biologically significant in protecting reproductive tissues against pathogens and oxidative damage. These tissue-specific accumulations suggest that different plant organs prioritize distinct phenolic compounds as part of their adaptive defense strategies^132–134^.

These findings are consistent with those of Akhter et al.^150^, who reported the presence of several phenolic compounds in the fruit extract of *C. melo* var. *agrestis*through HPLC analysis, including gallic acid (10.50 µg/g), chlorogenic acid (36.43 µg/g), 4-hydroxybenzoic acid (37.85 µg/g), kaempferol (7.99 µg/g), caffeic acid (9.85 µg/g), and quercetin (12.45 µg/g). Similarly, Zulfiqar et al.^151^, confirmed the presence of eleven phenolic compounds in the fruit extract, with chlorogenic acid being the most abundant, followed by gallic acid and vanillic acid^2,34,44,63,152–160^.

## Conclusion

In the present study, the morphological and anatomical characteristics of the vegetative parts—including the stem, petiole, and leaf—as well as pollen and seed features of *Cucumis melo* var. *agrestis* were examined using light microscopy and scanning electron microscopy (SEM). These results provide robust morphological and anatomical markers that support the identification and taxonomic clarification of *Cucumis melo* var. *agrestis*. Additionally, the ethanolic leaf extract of *C. melo* var. *agrestis* exhibited the highest total phenolic and flavonoid content ,which , correlated with strong antioxidant activity. Phytochemical screening confirmed the presence of diverse classes of bioactive compounds, highlighting the pharmacological potential of *Cucumis melo* var. *agrestis*. These findings suggest that the plant may be a valuable source of natural antioxidants and other therapeutically significant phytochemicals.

## Supplementary Information

Below is the link to the electronic supplementary material.

Supplementary Material 1

Supplementary Material 2

## Data Availability

All data generated or analysed during this study are included in this published article and its supplementary information files.
