# Supplementary figures and images for: Macro-micromorphological, anatomical, and phytochemical characterization of Cucumis melo var. agrestis Naudin: a potential source of natural antioxidants
Source: Sci Rep. 2026 Apr 17;16:12711. doi: 10.1038/s41598-026-47246-7 (PMC13090356; doi:10.1038/s41598-026-47246-7)

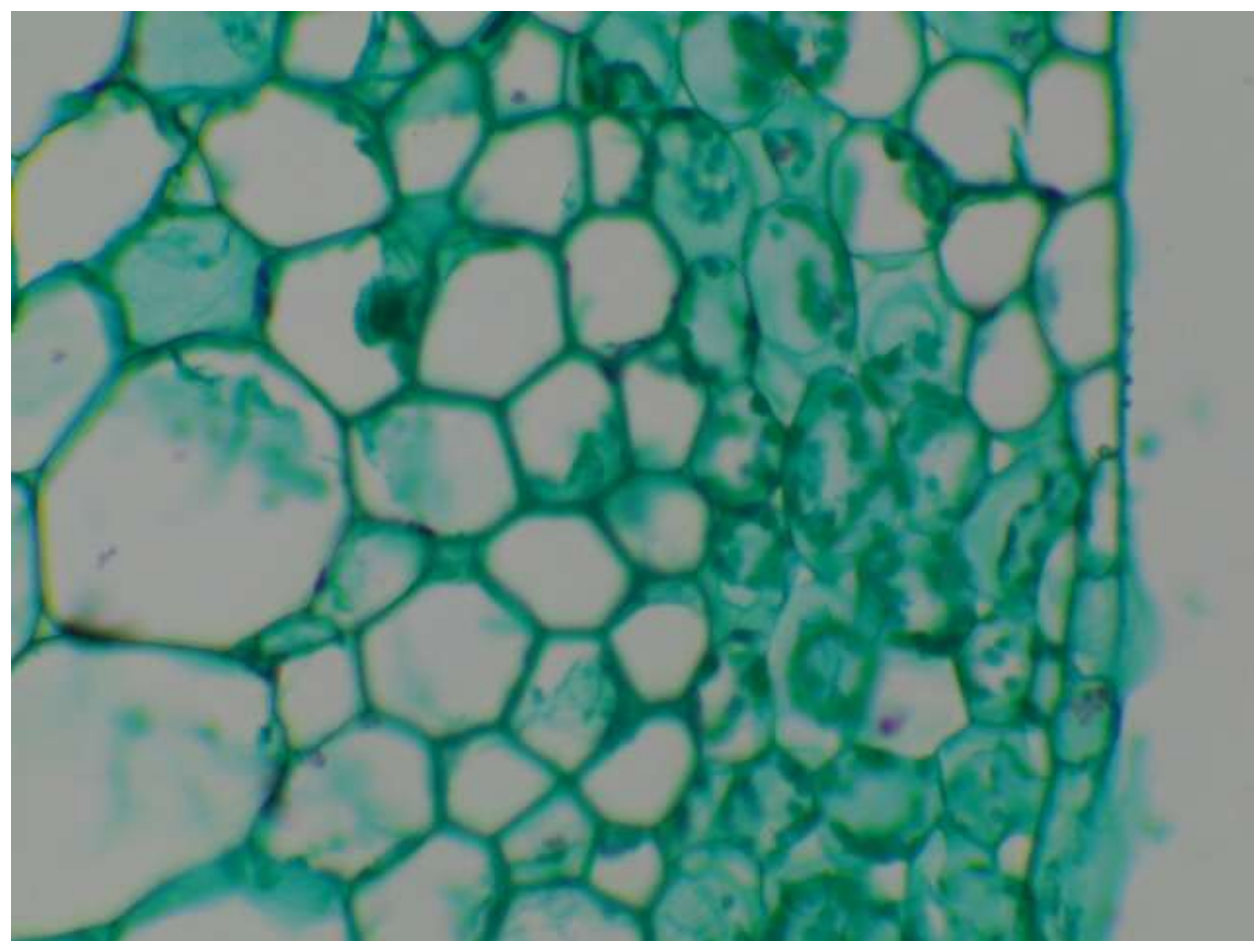

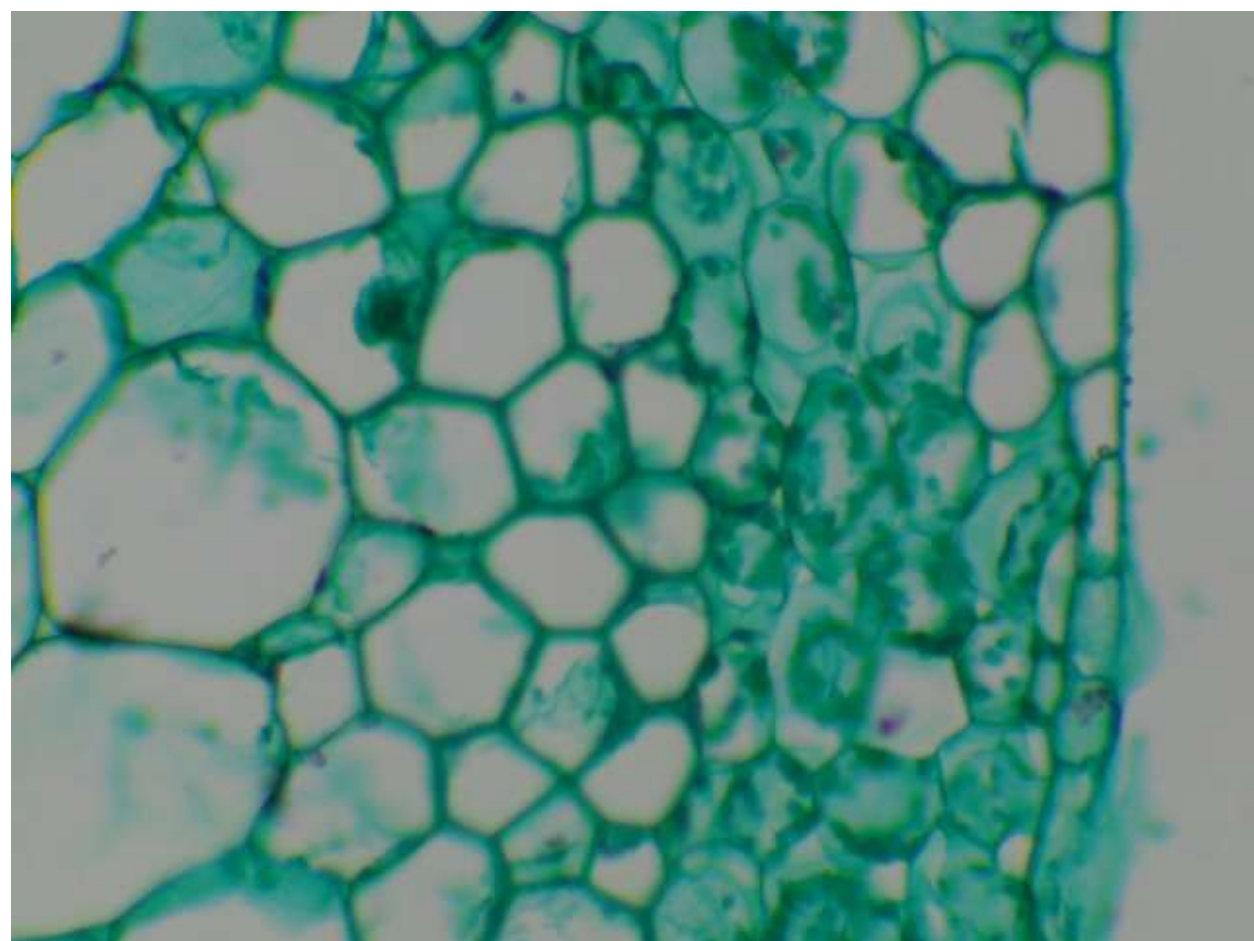

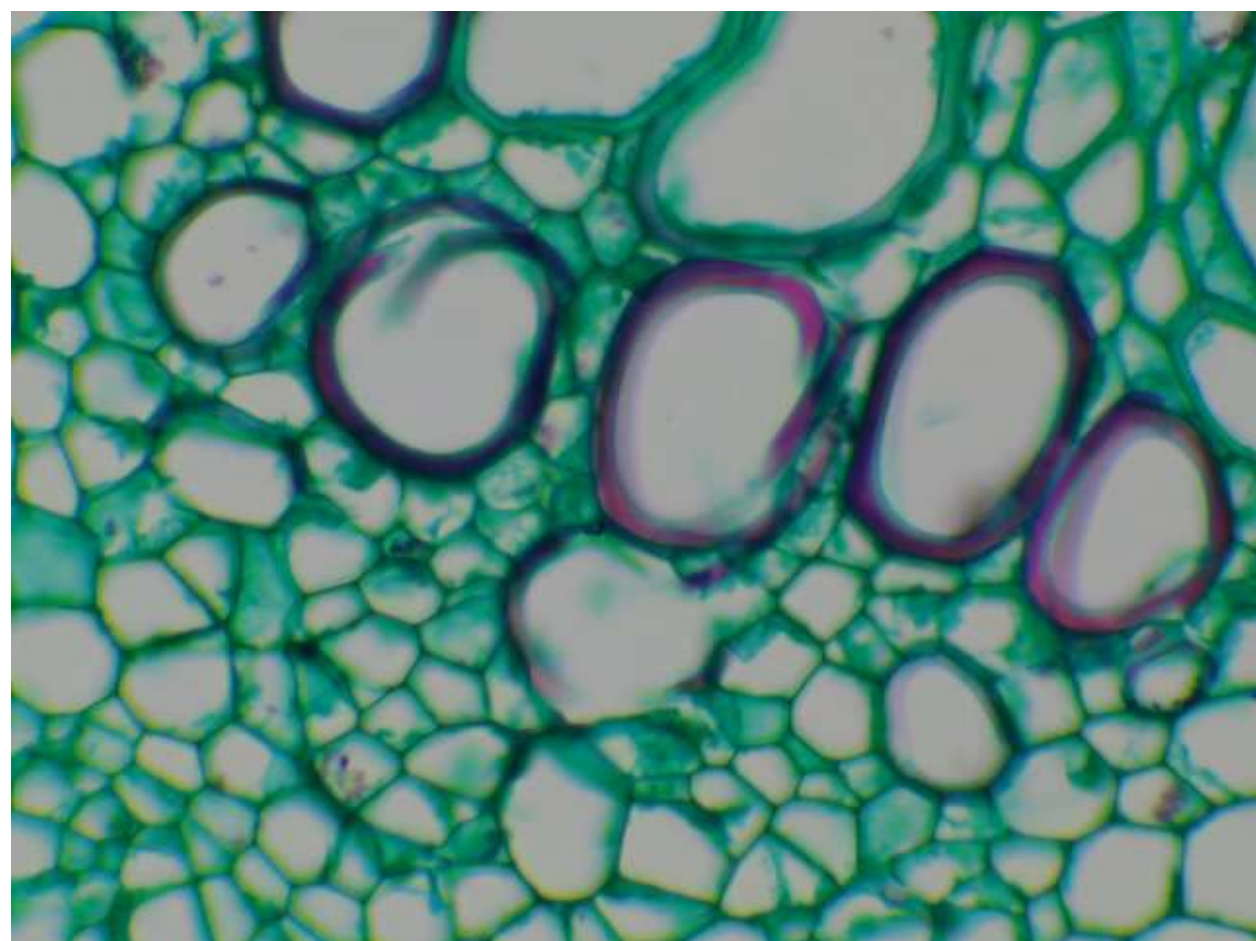

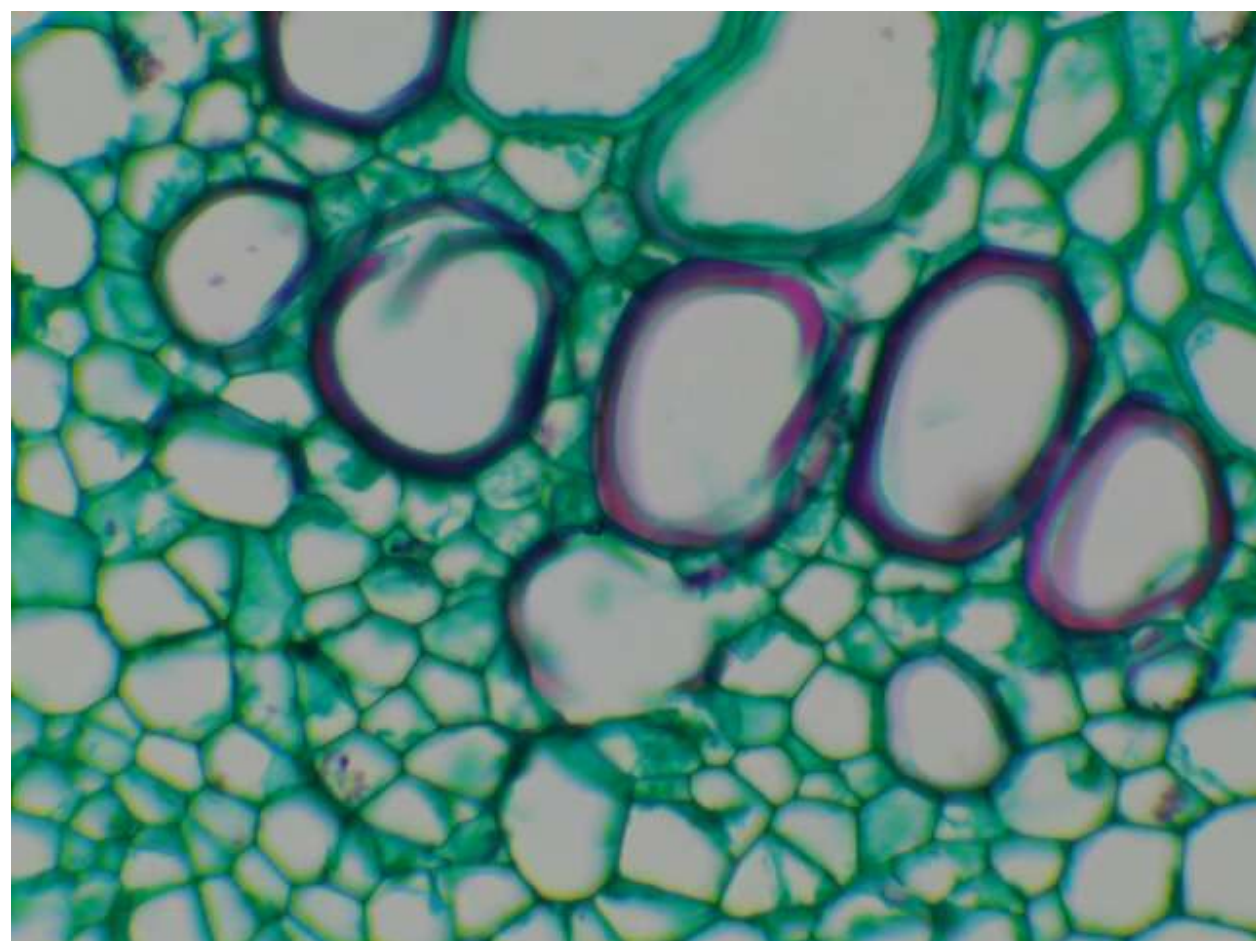

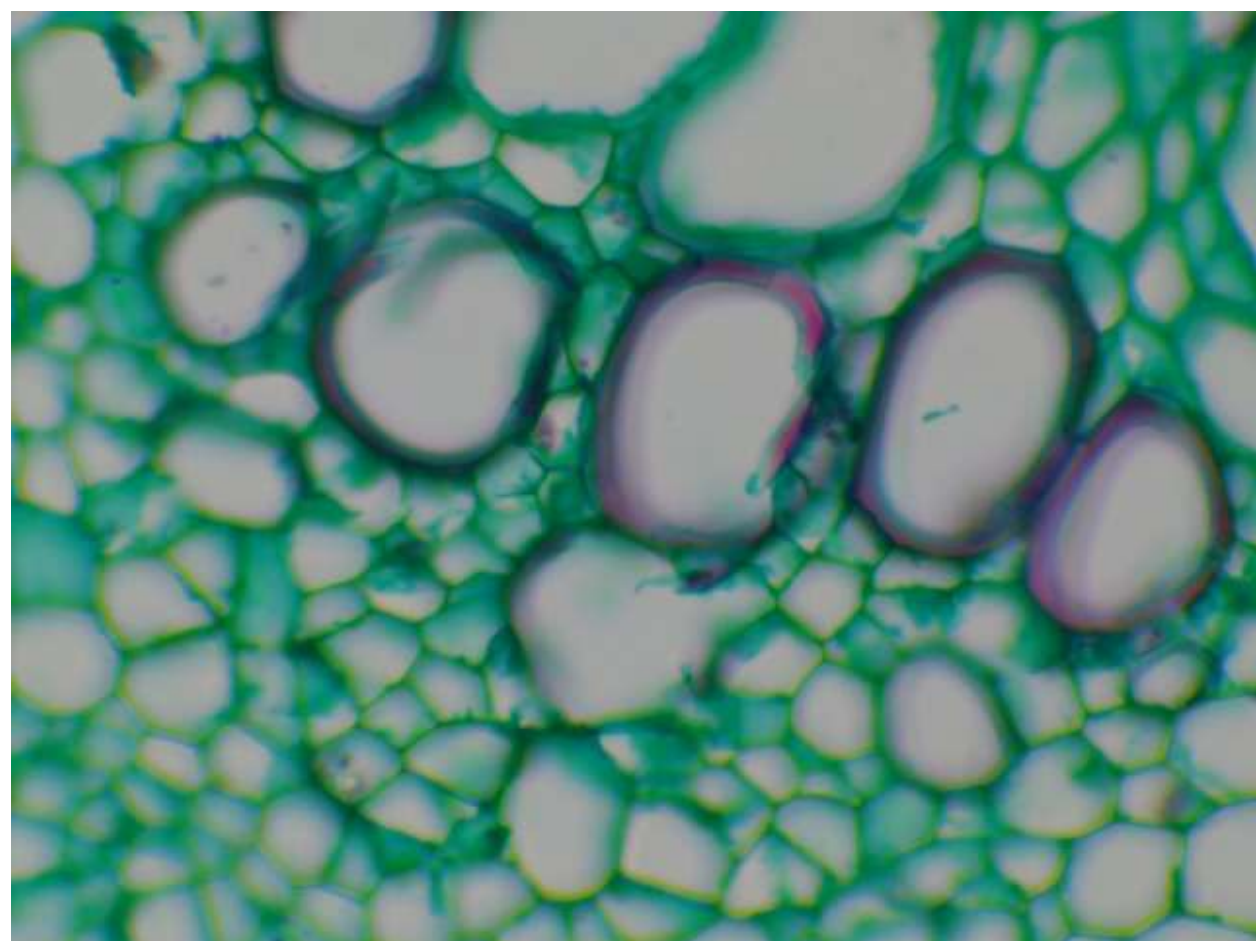

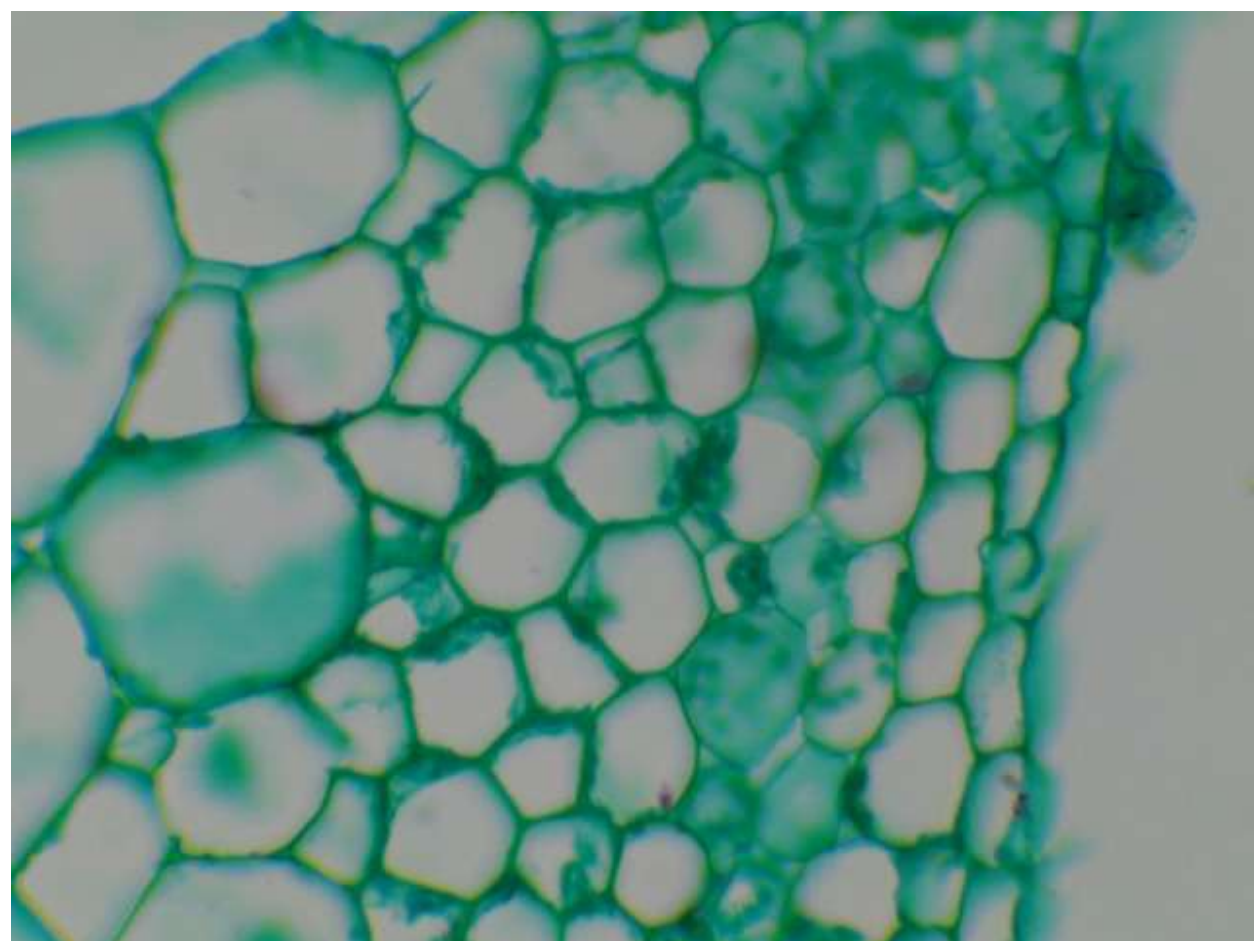

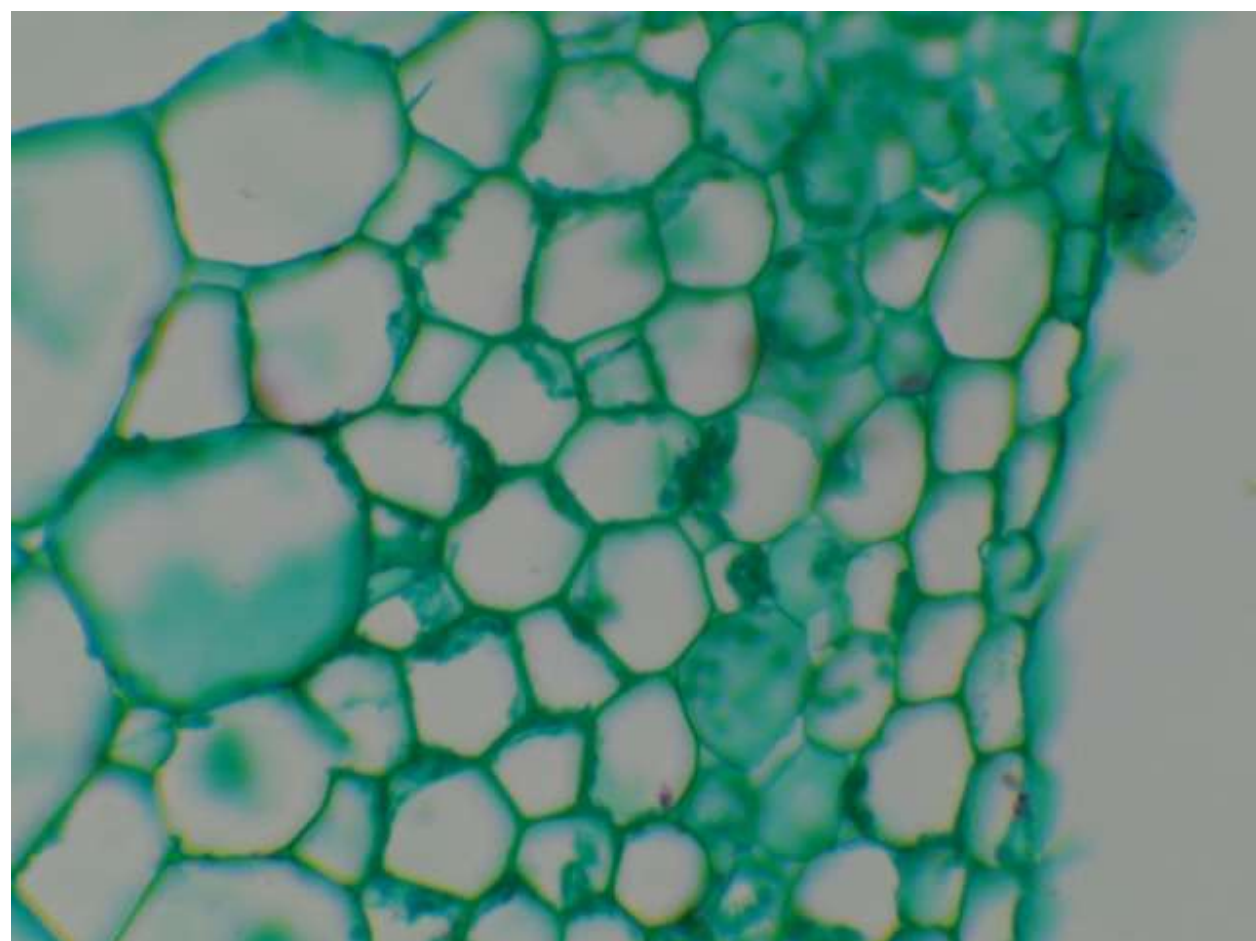

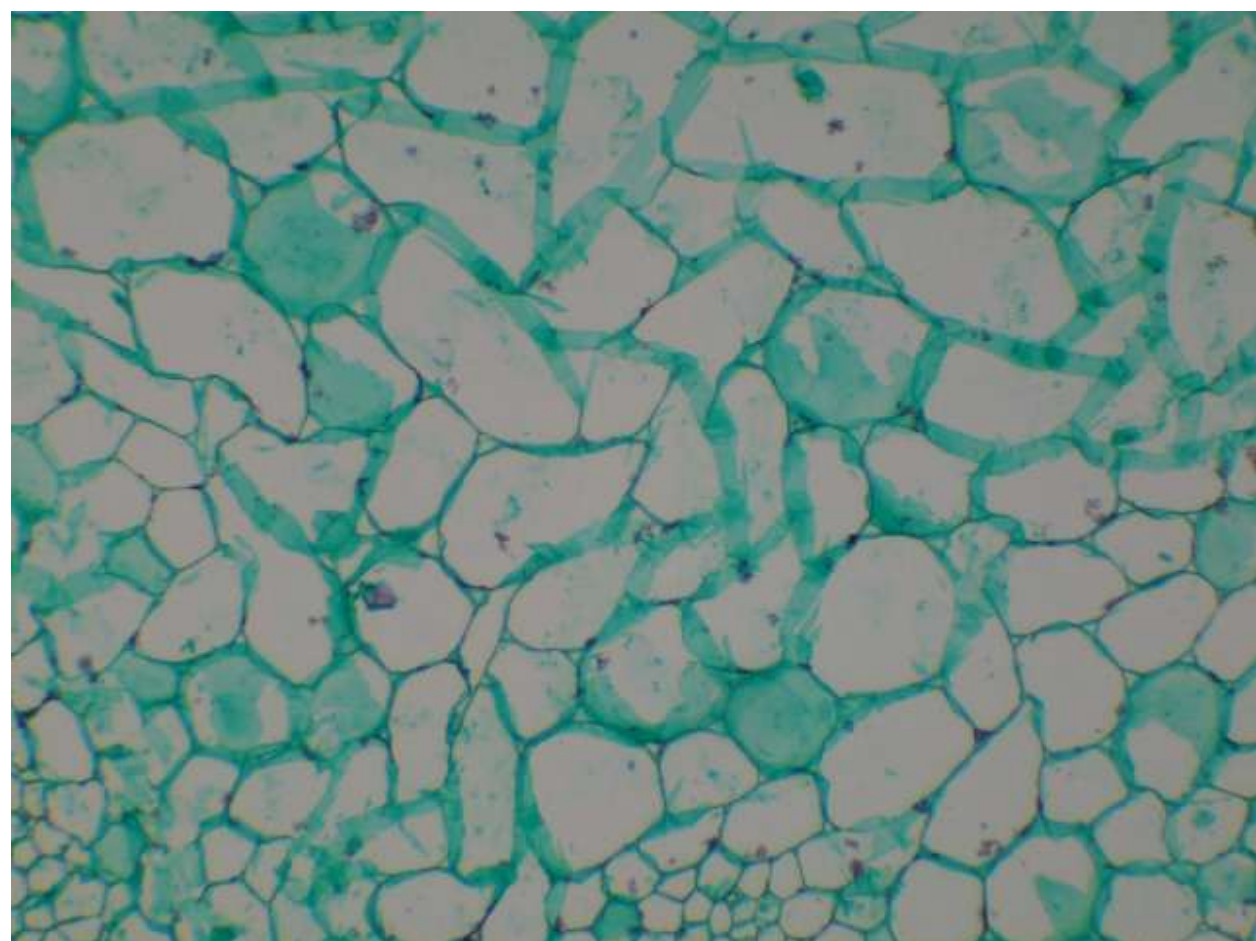

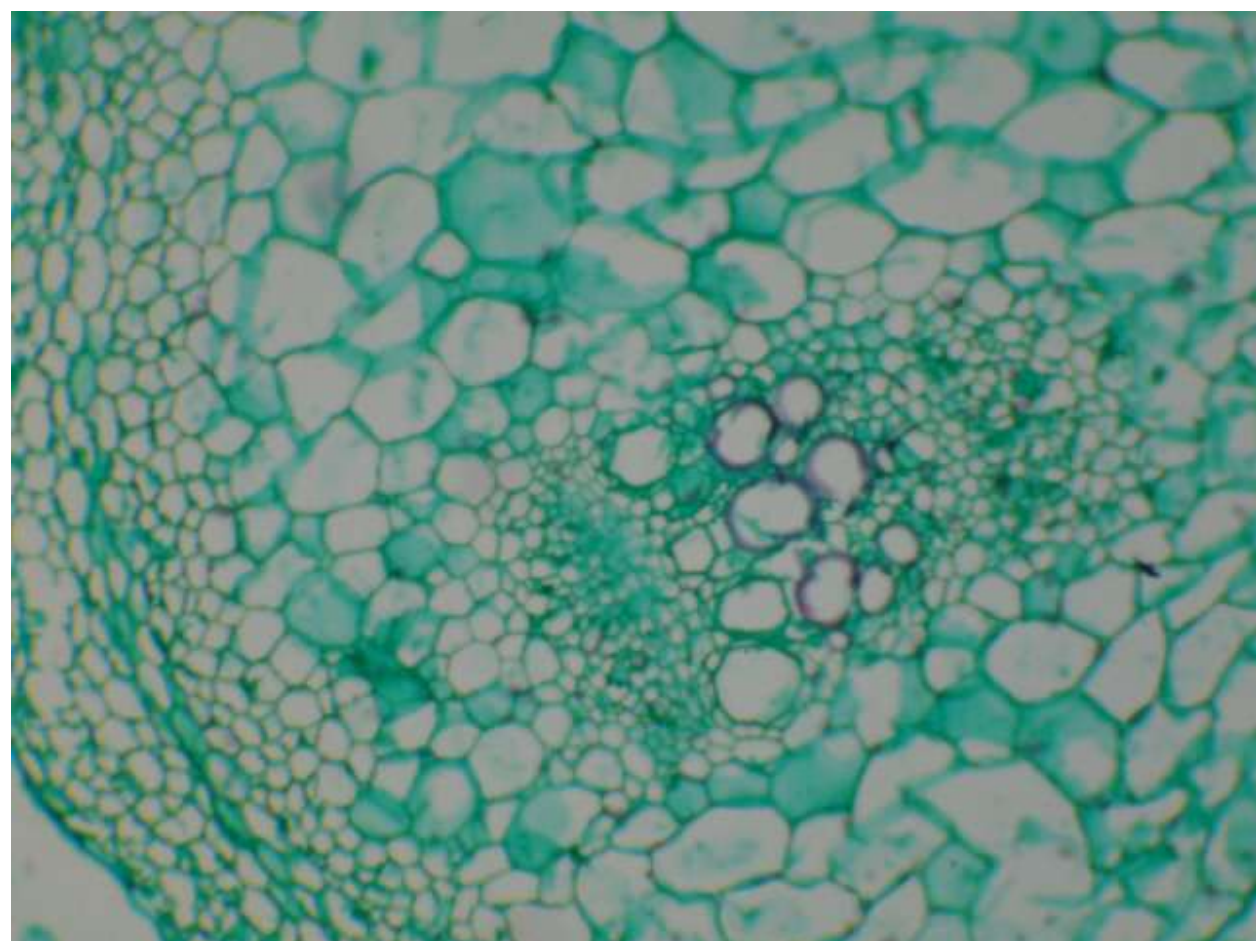

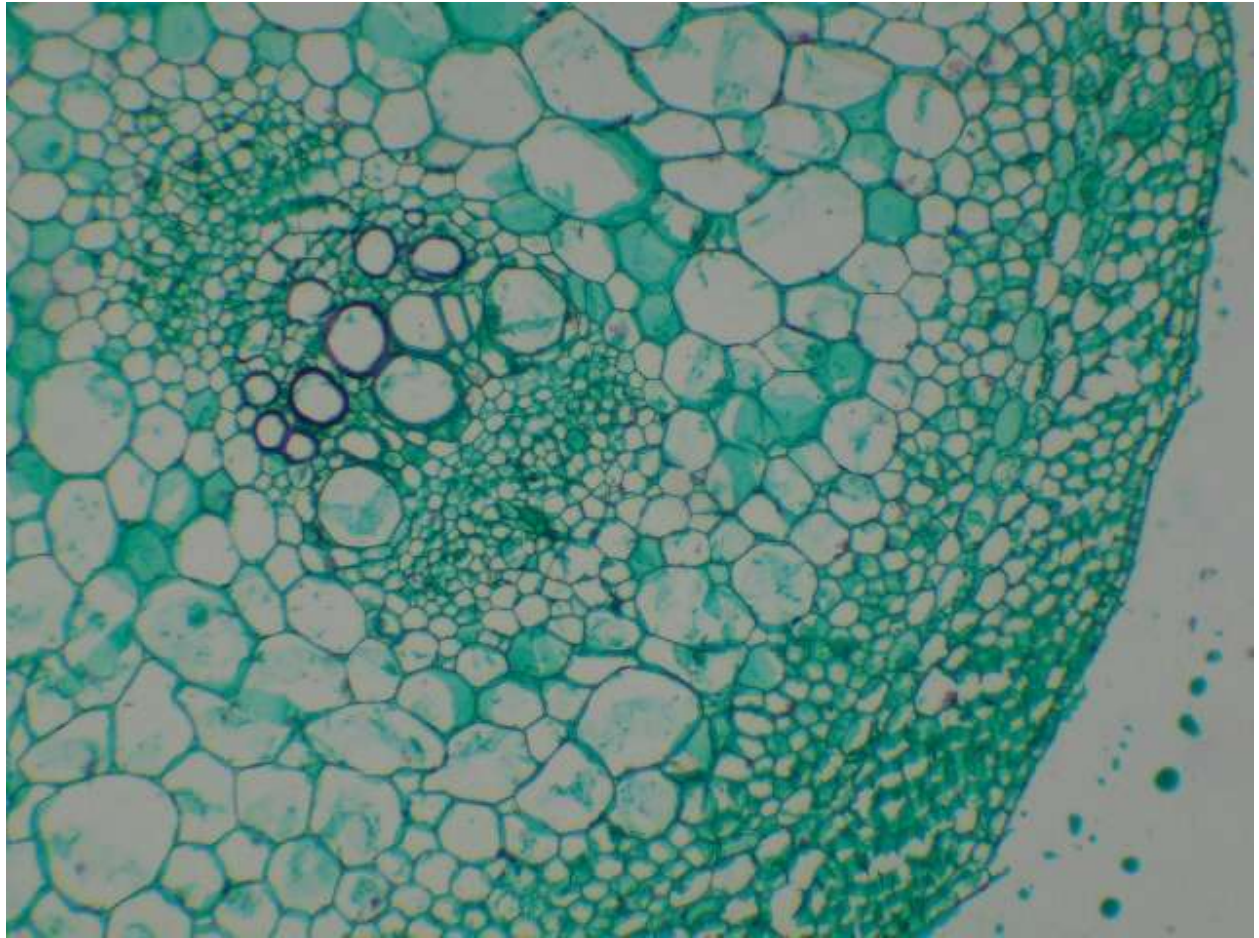

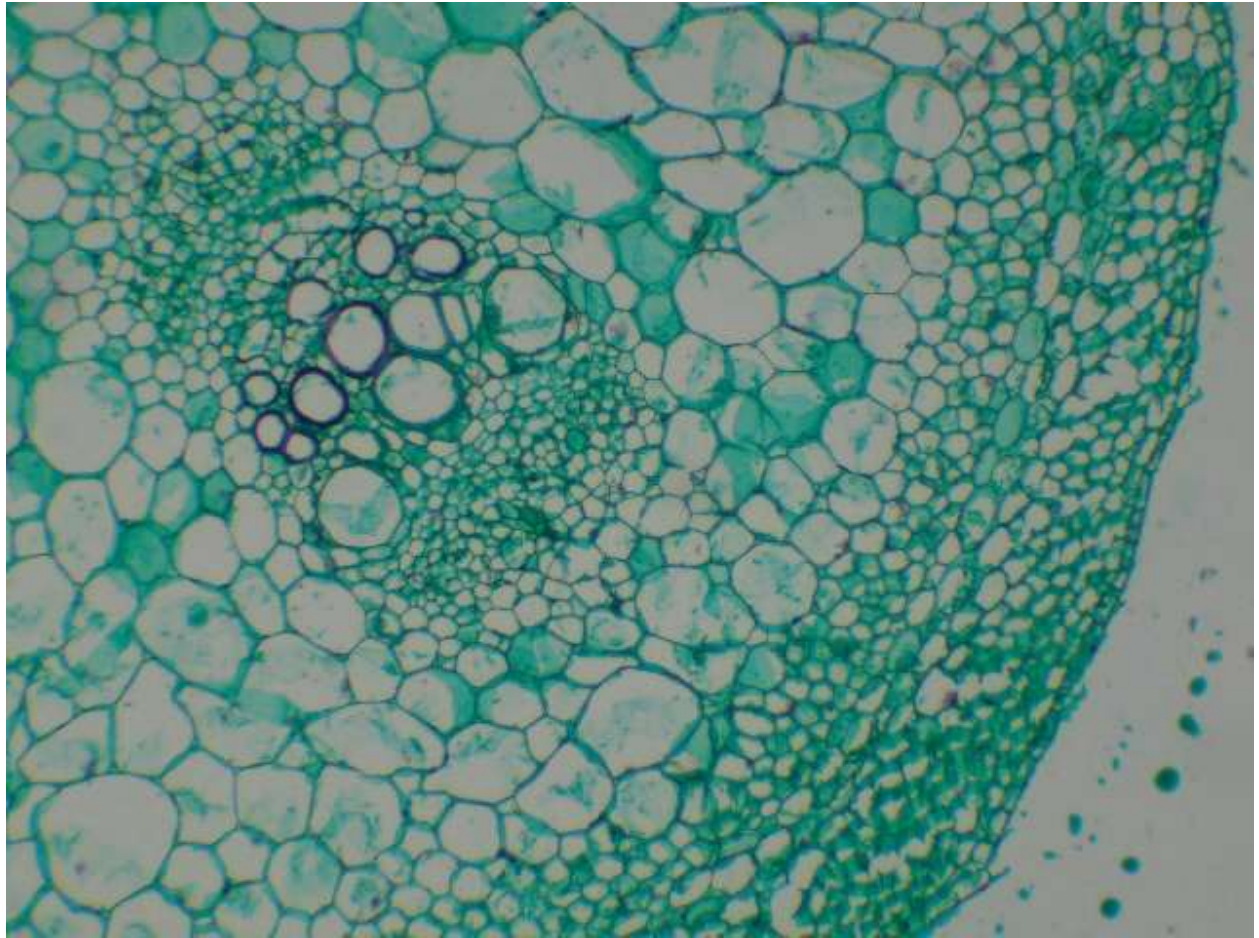

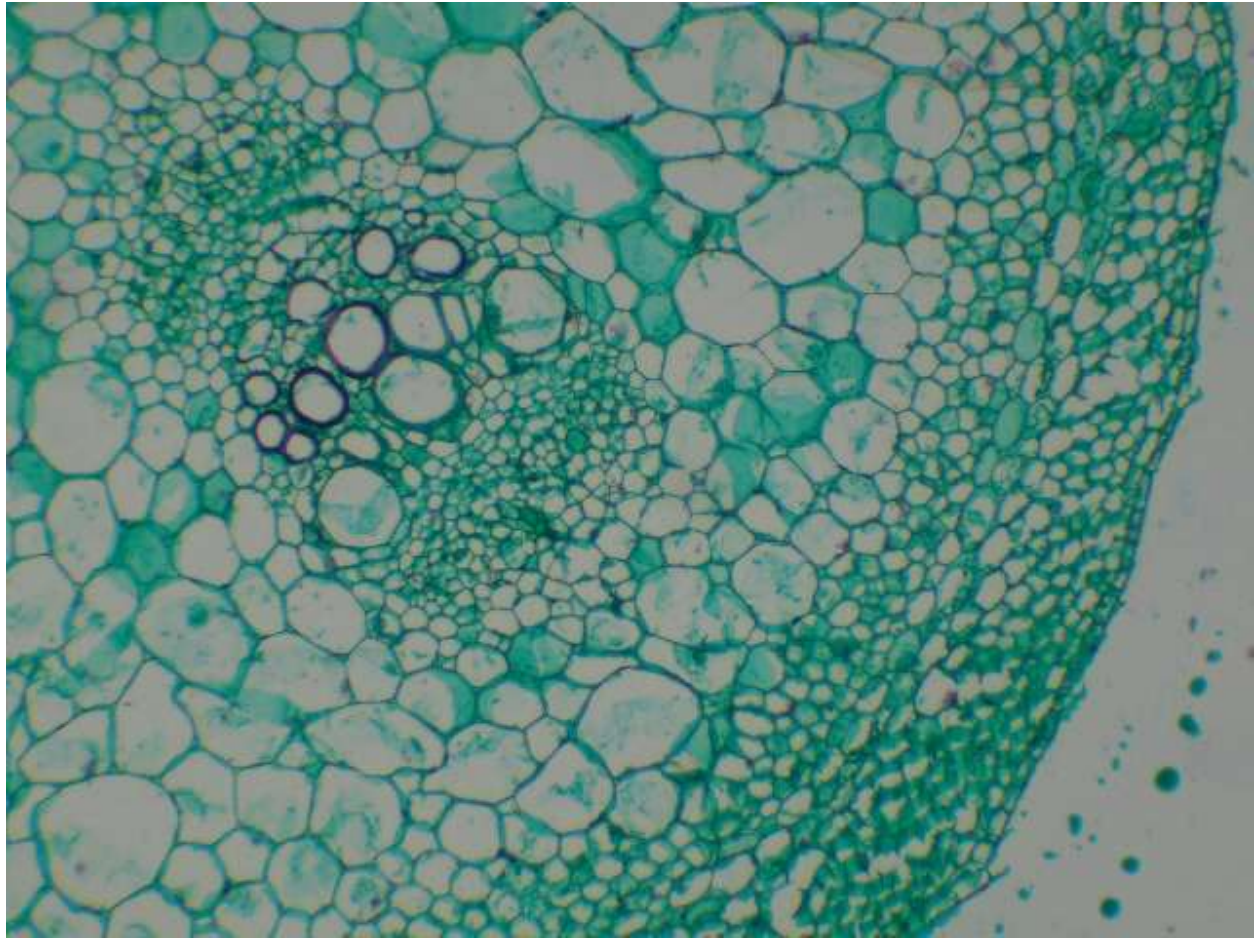

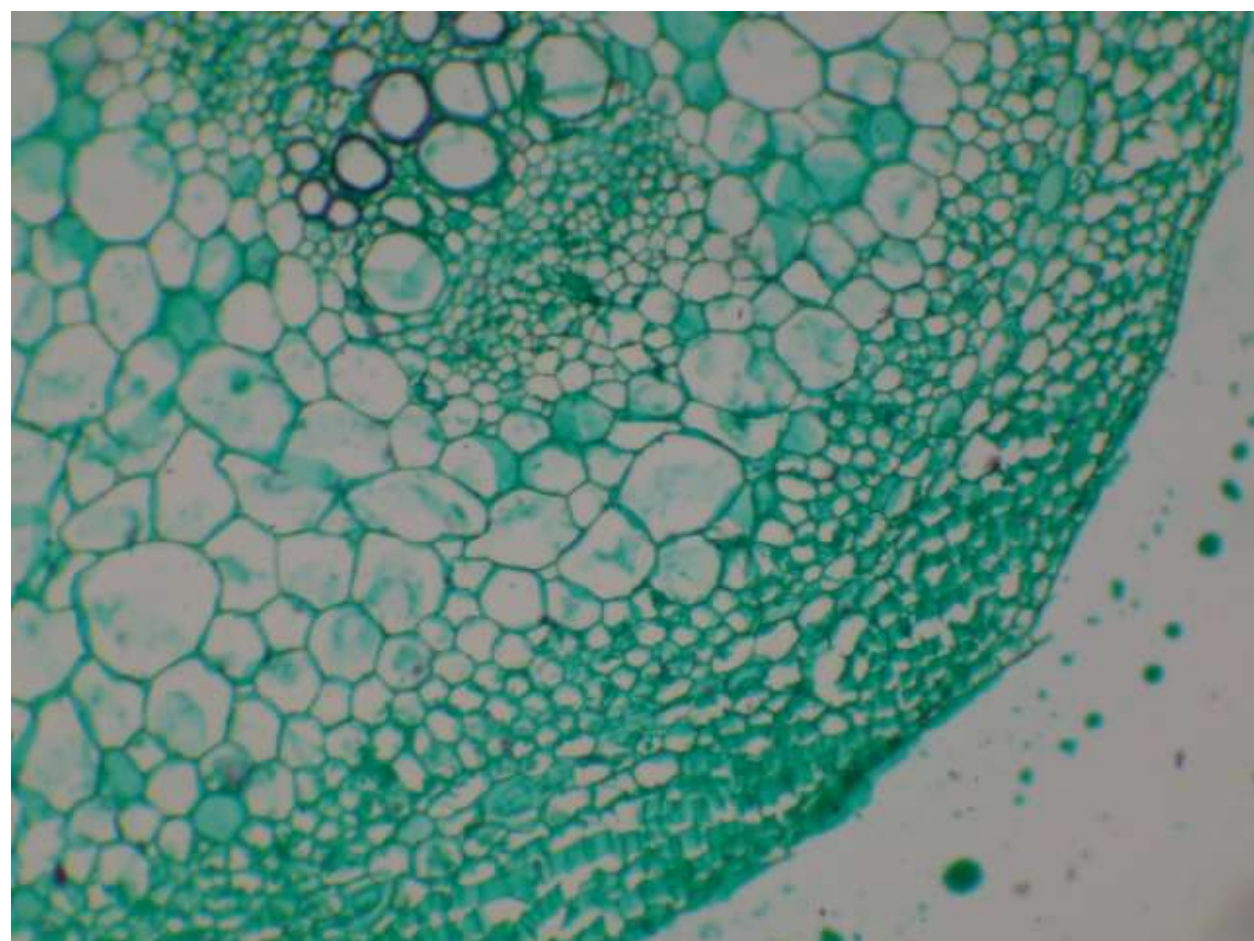

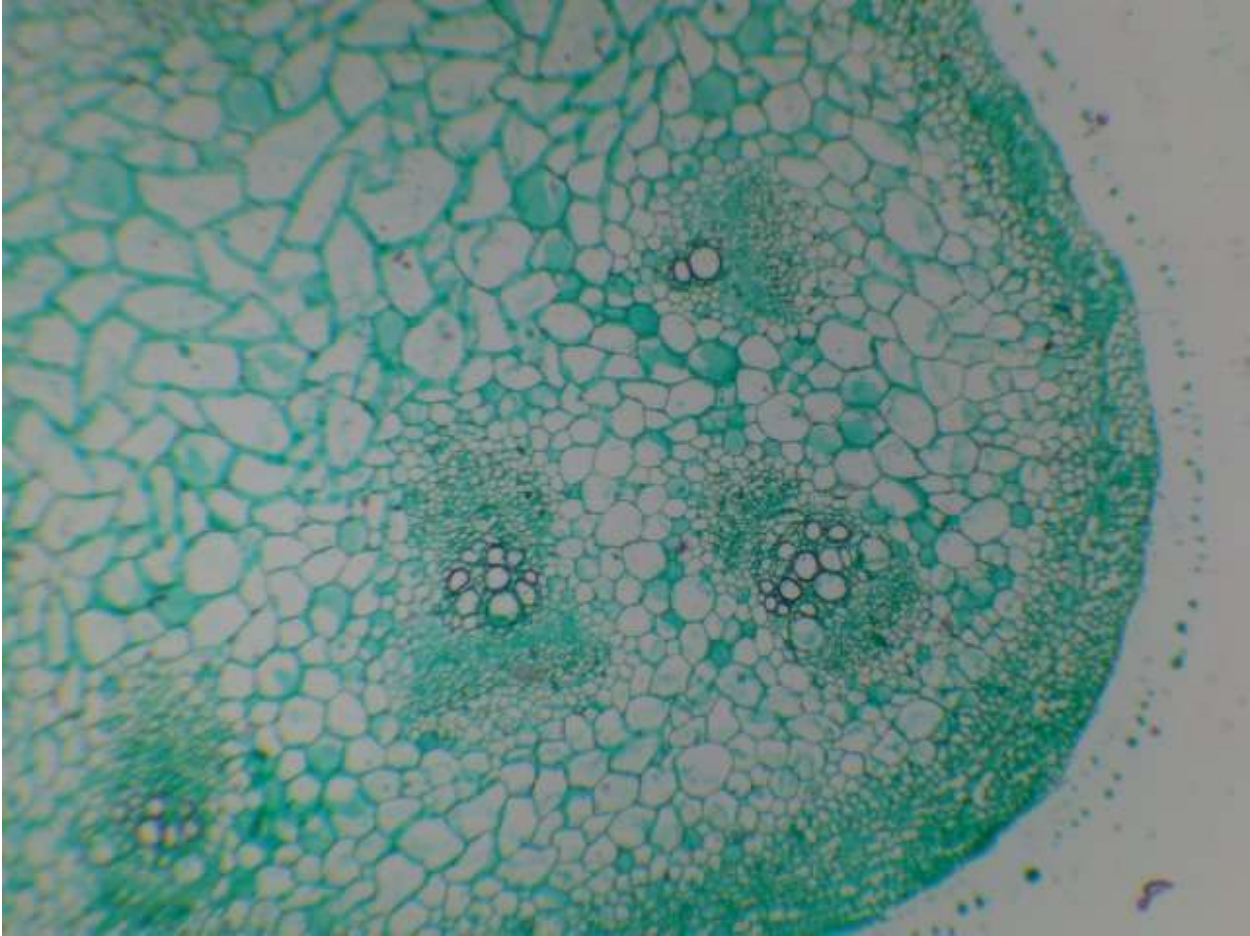

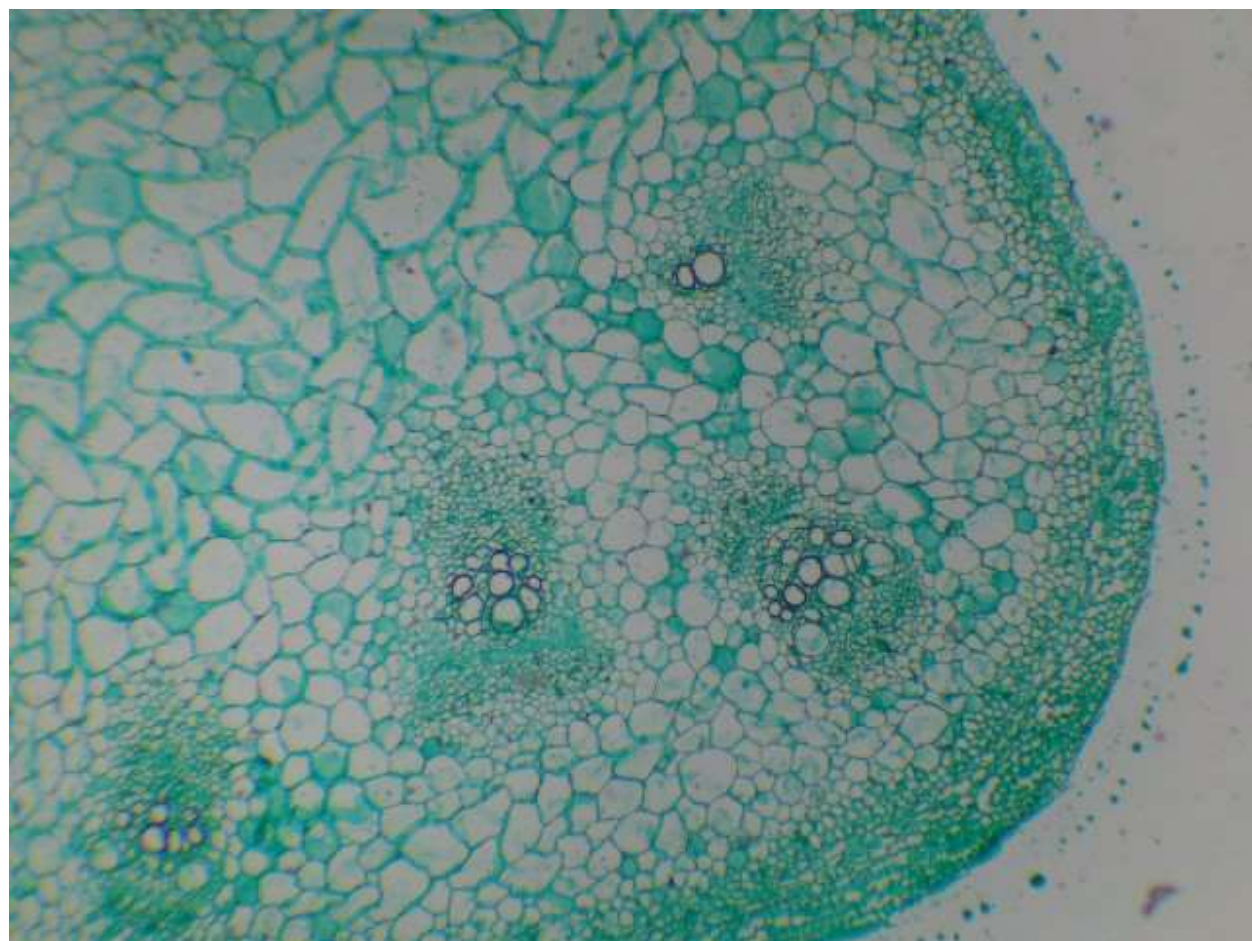

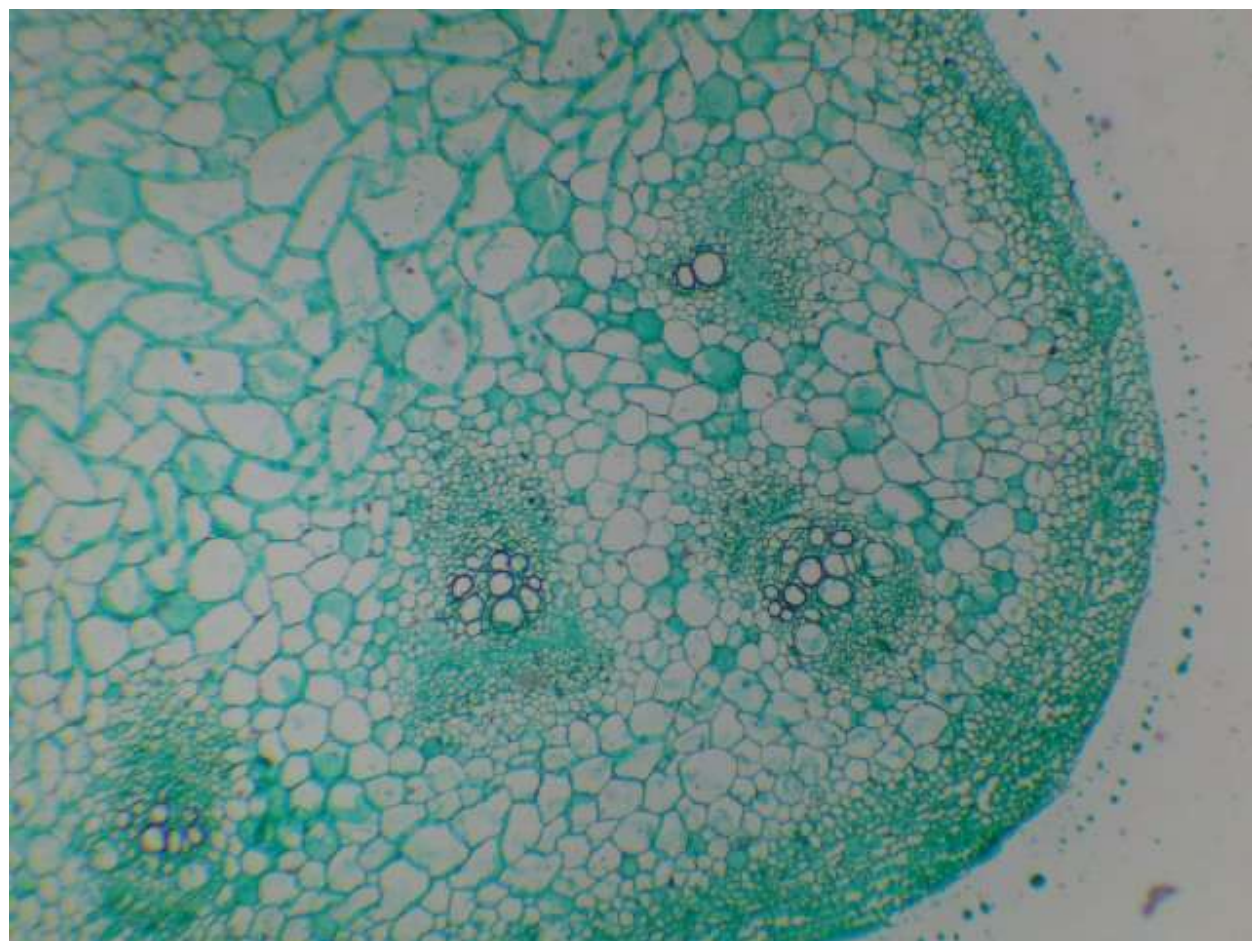

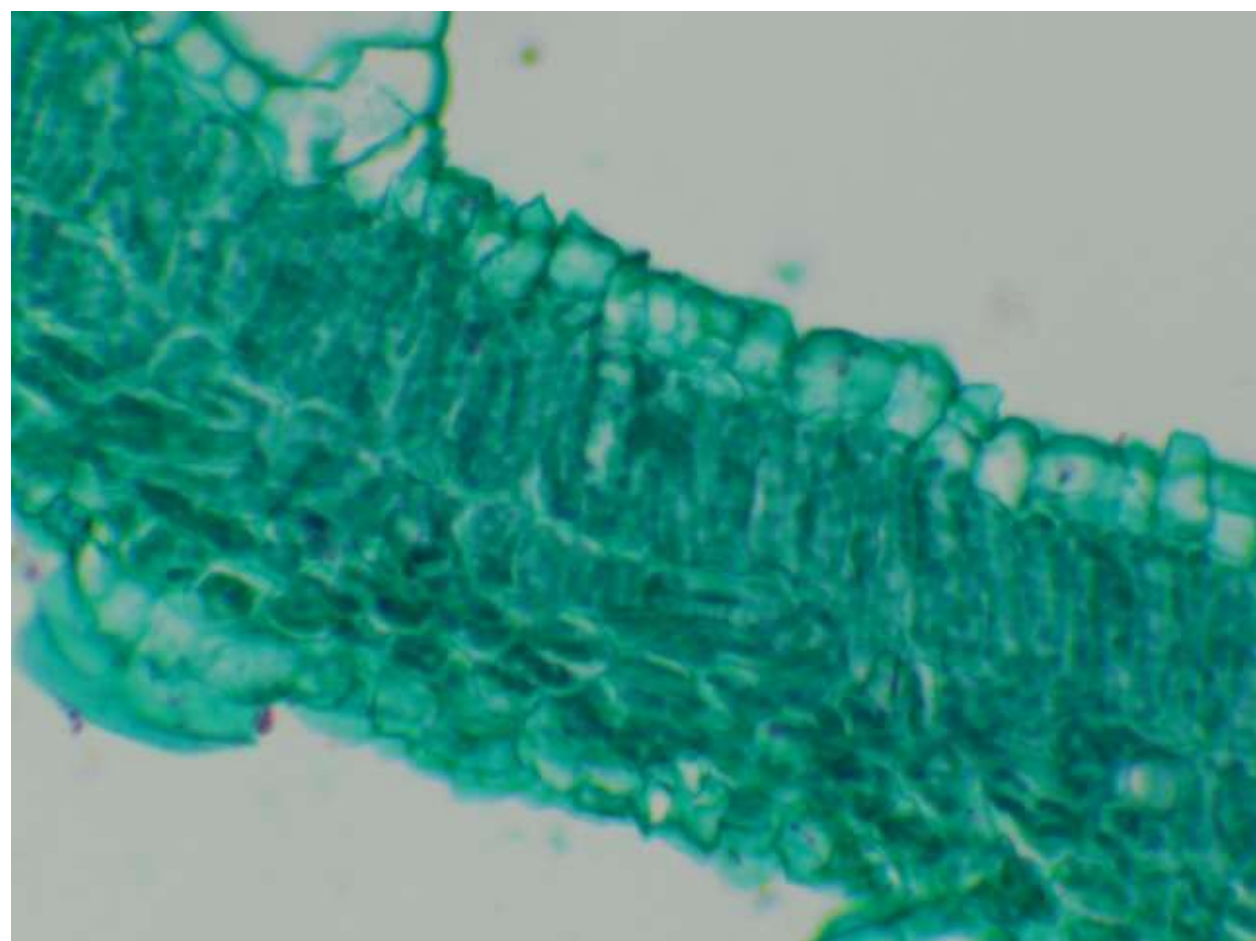

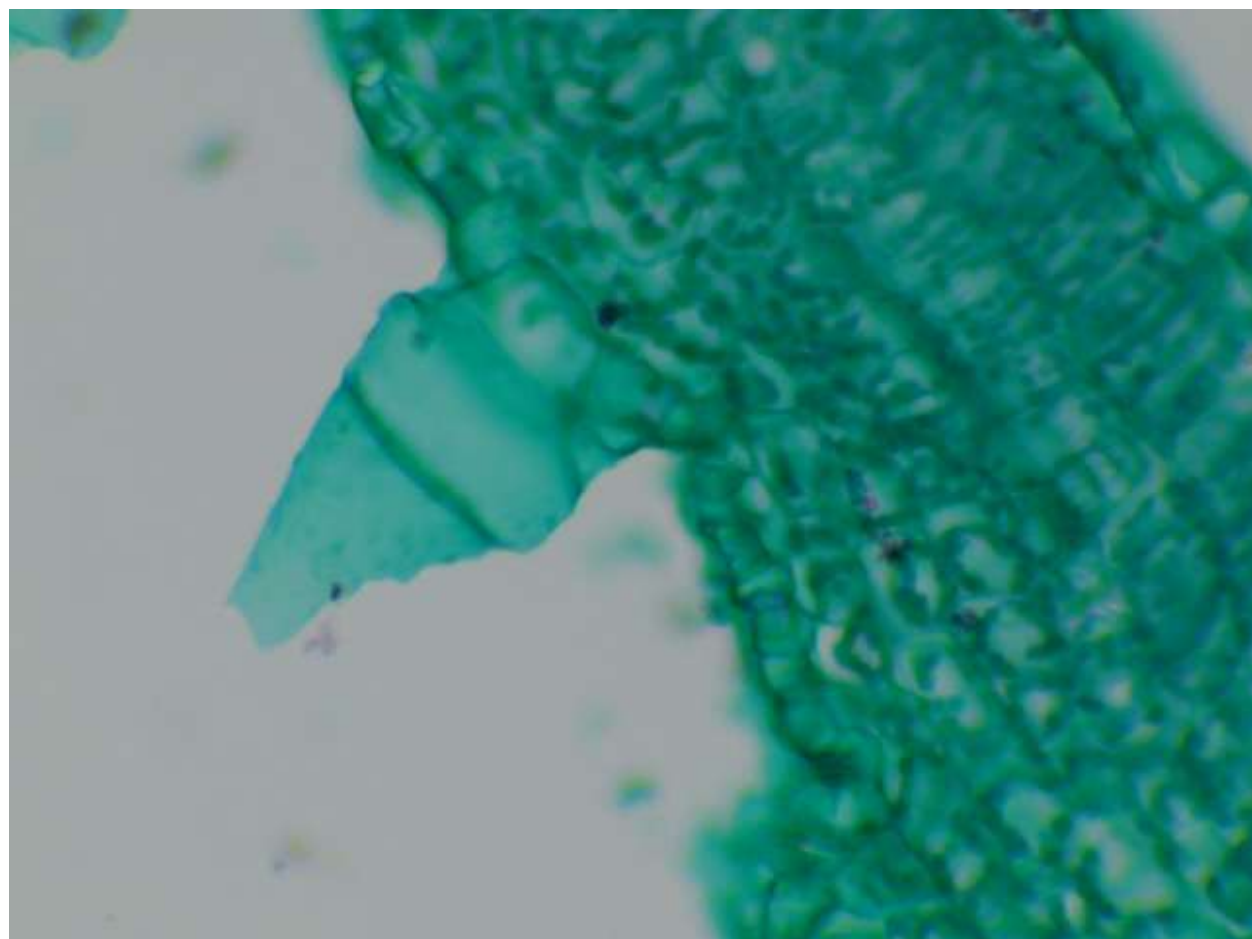

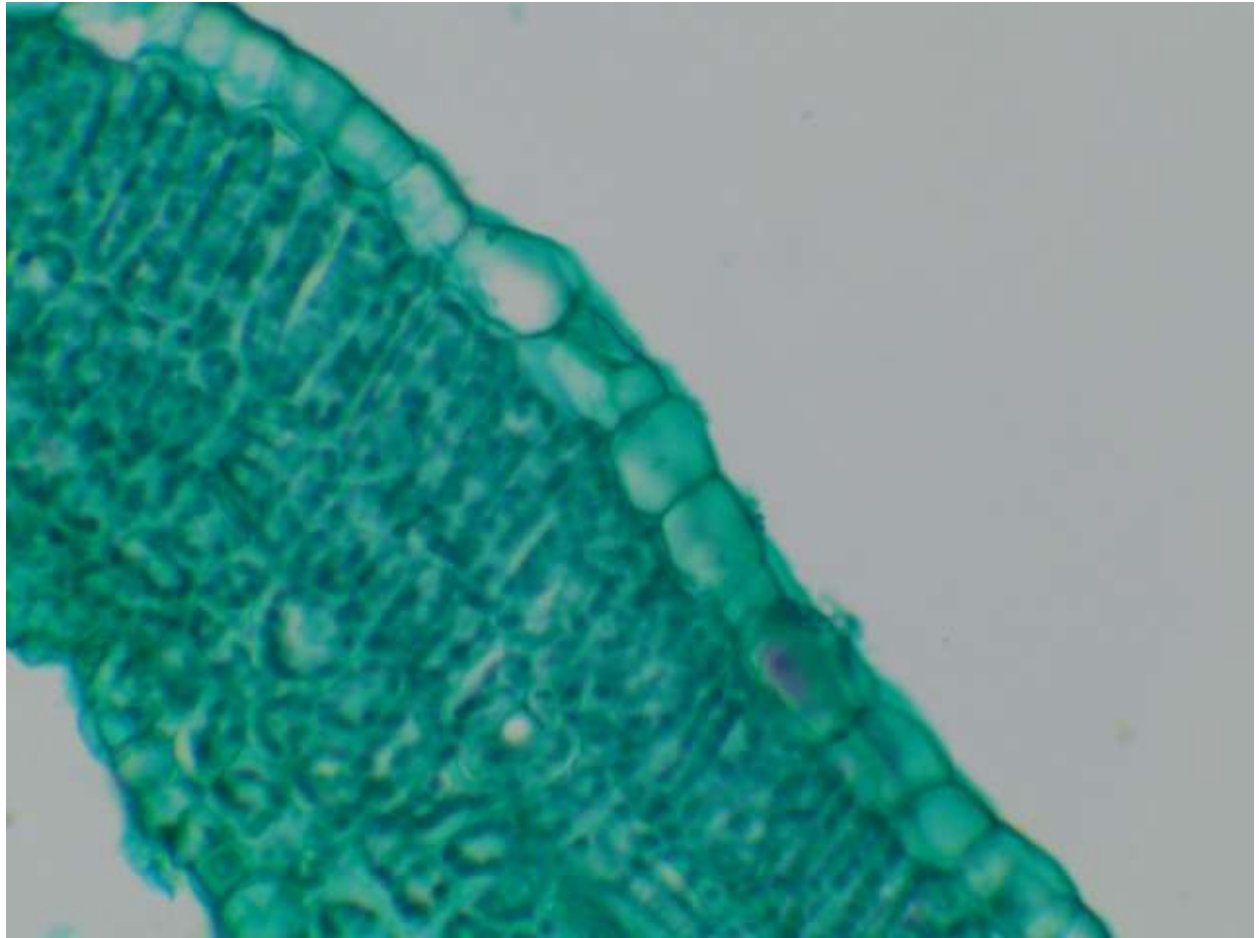

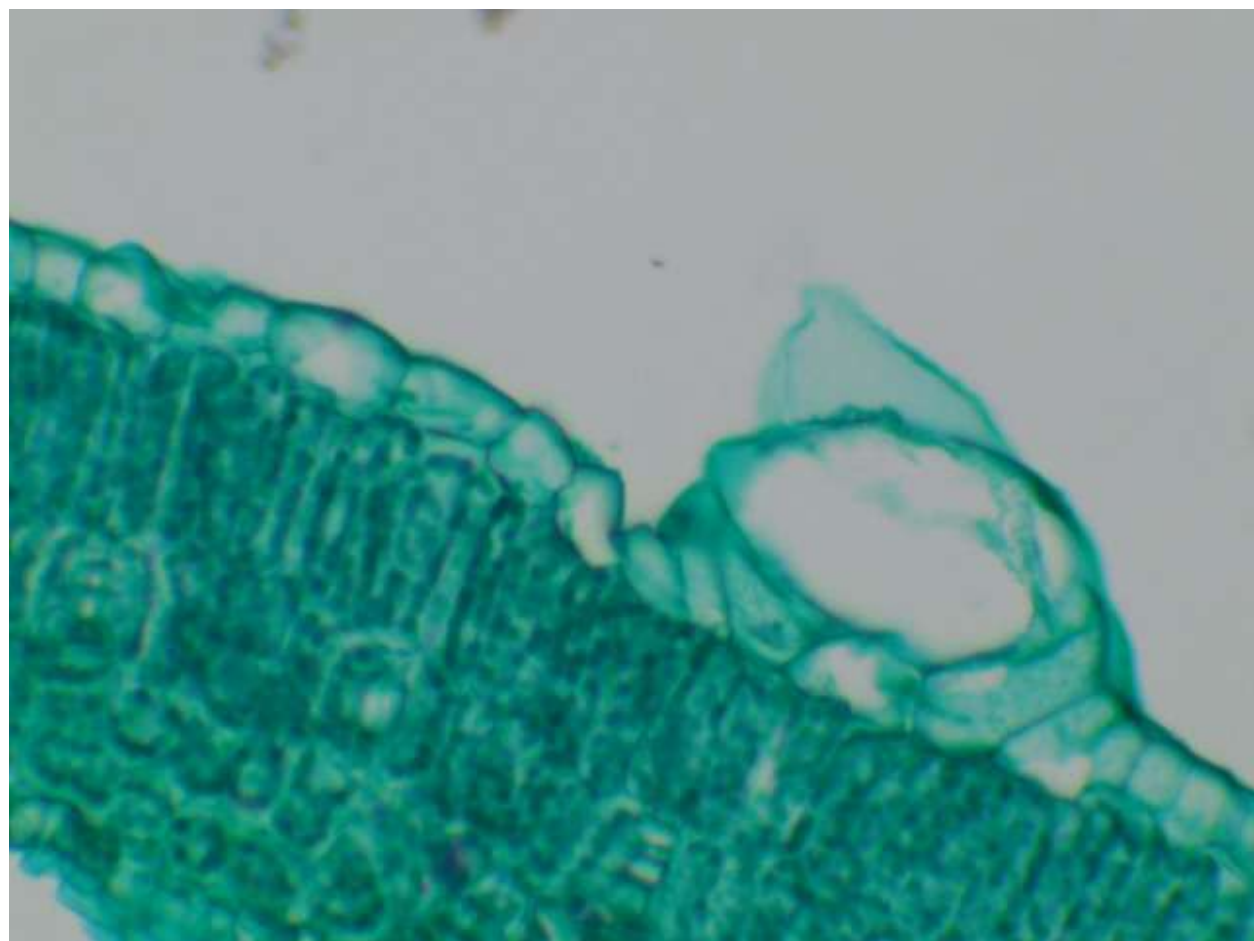

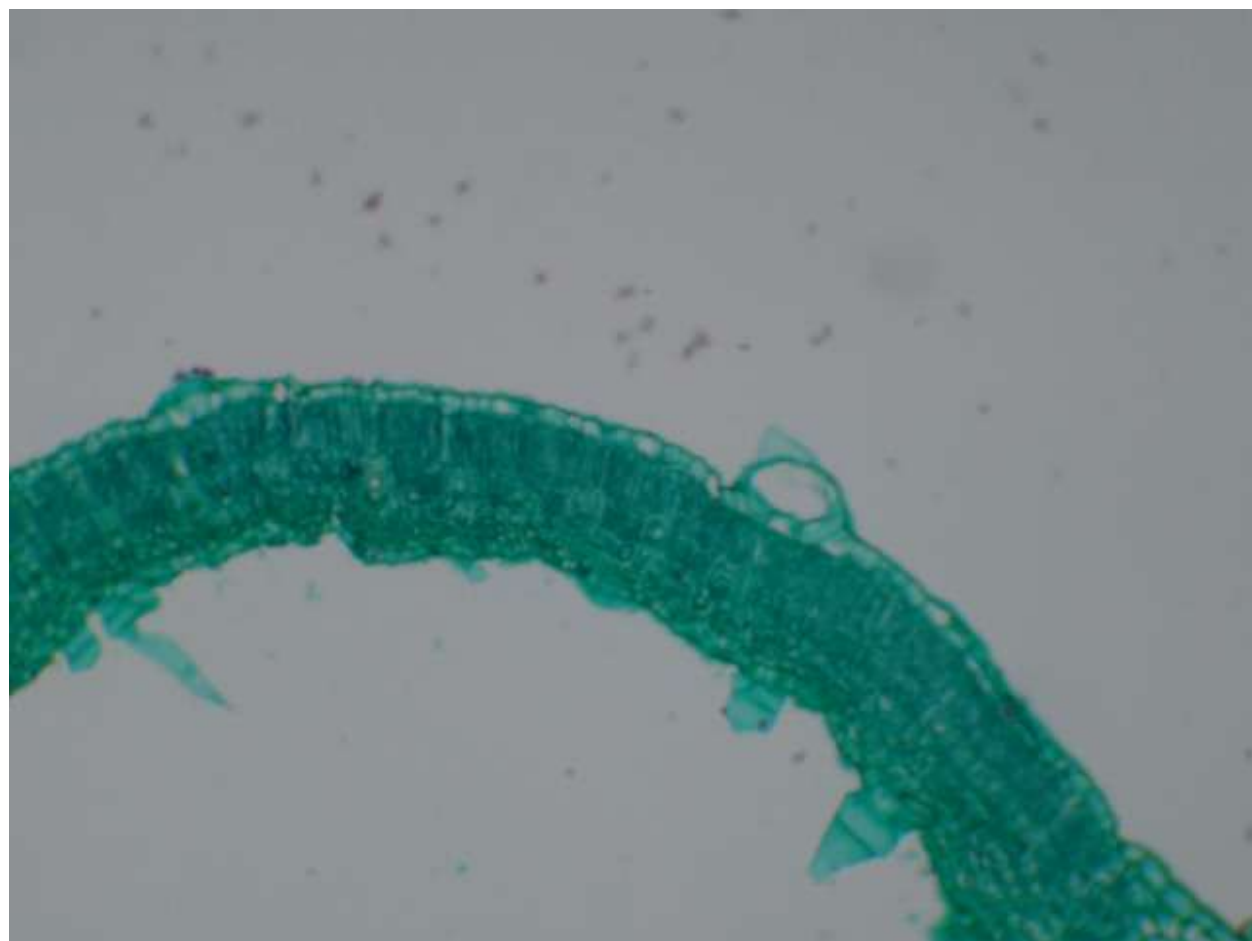

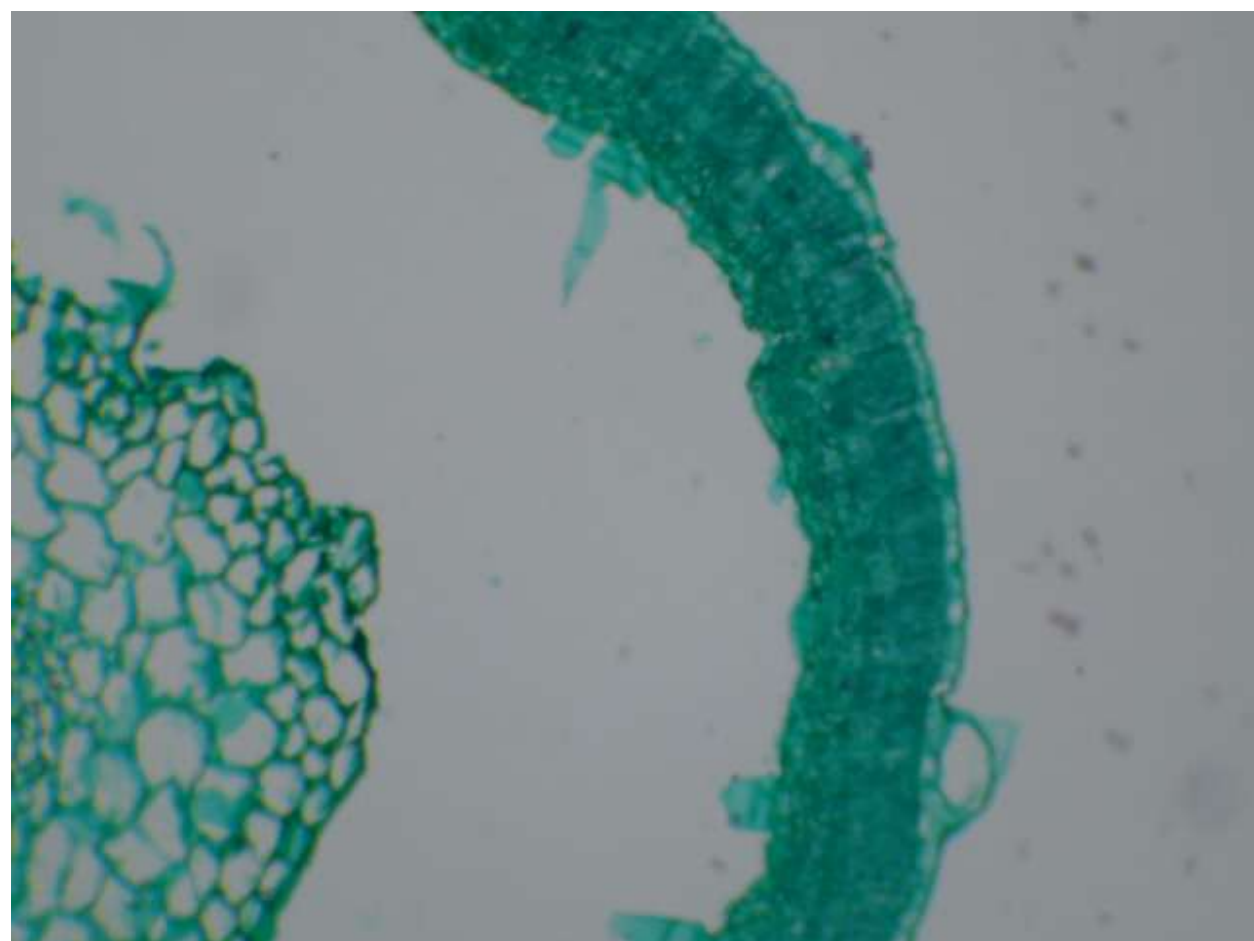

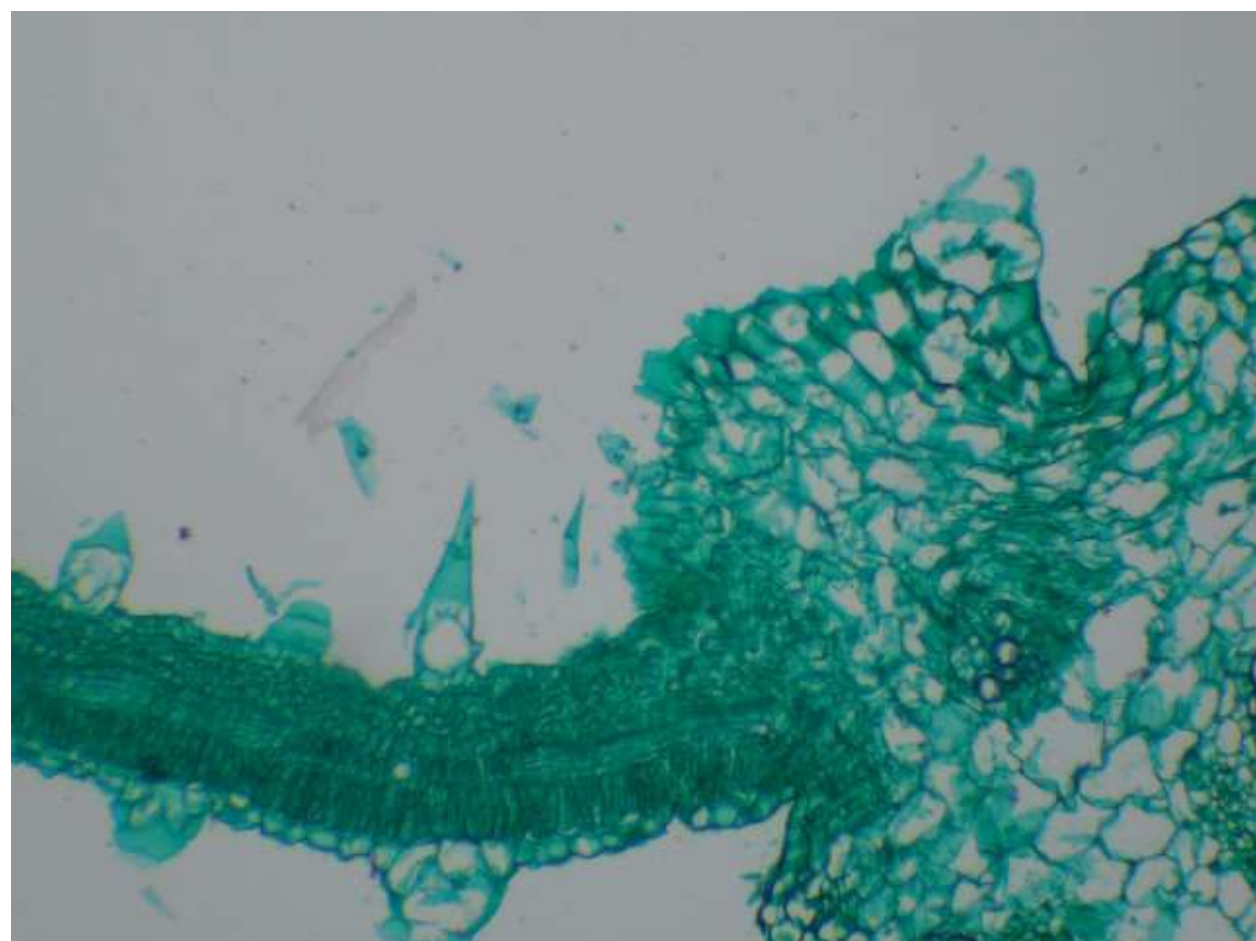

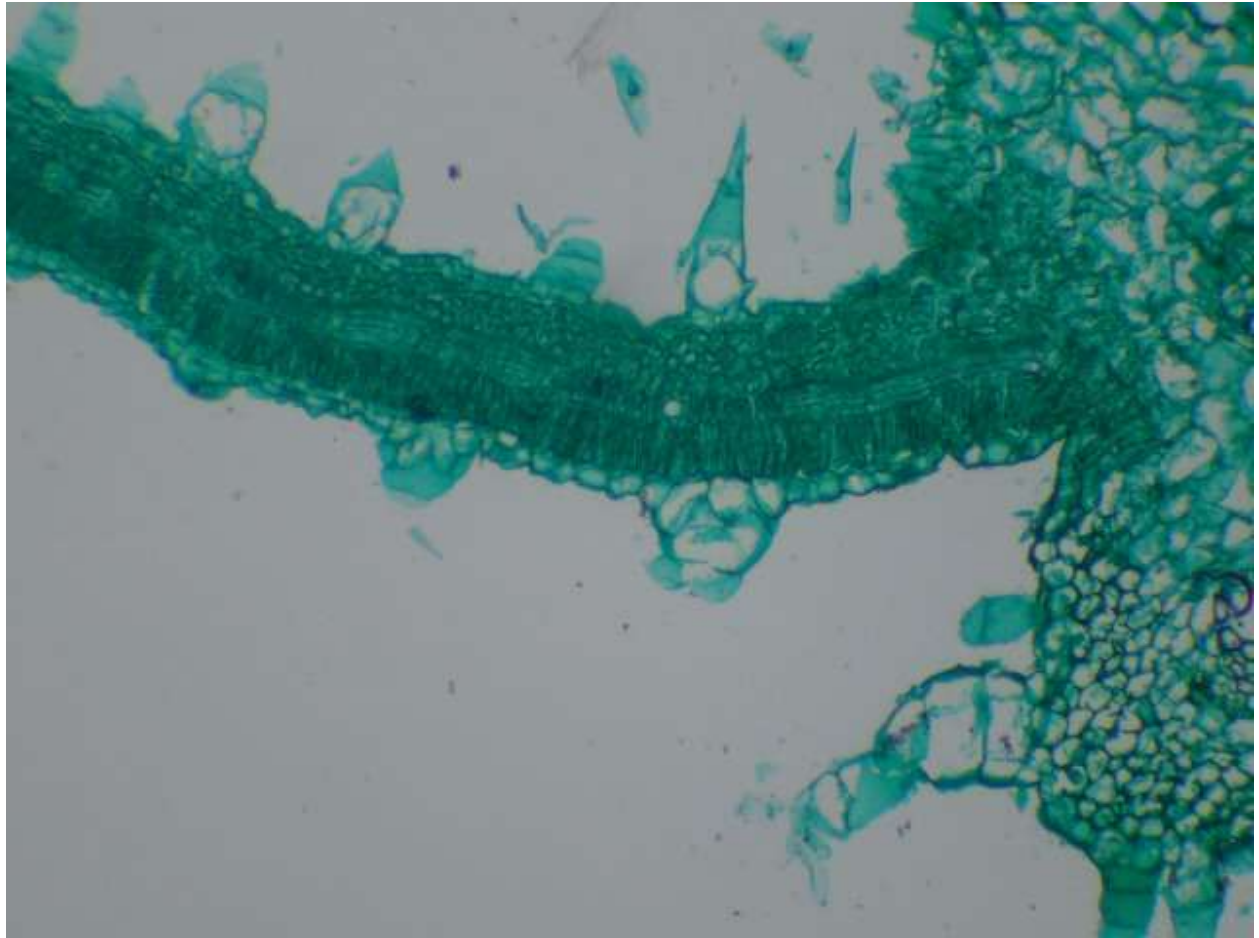

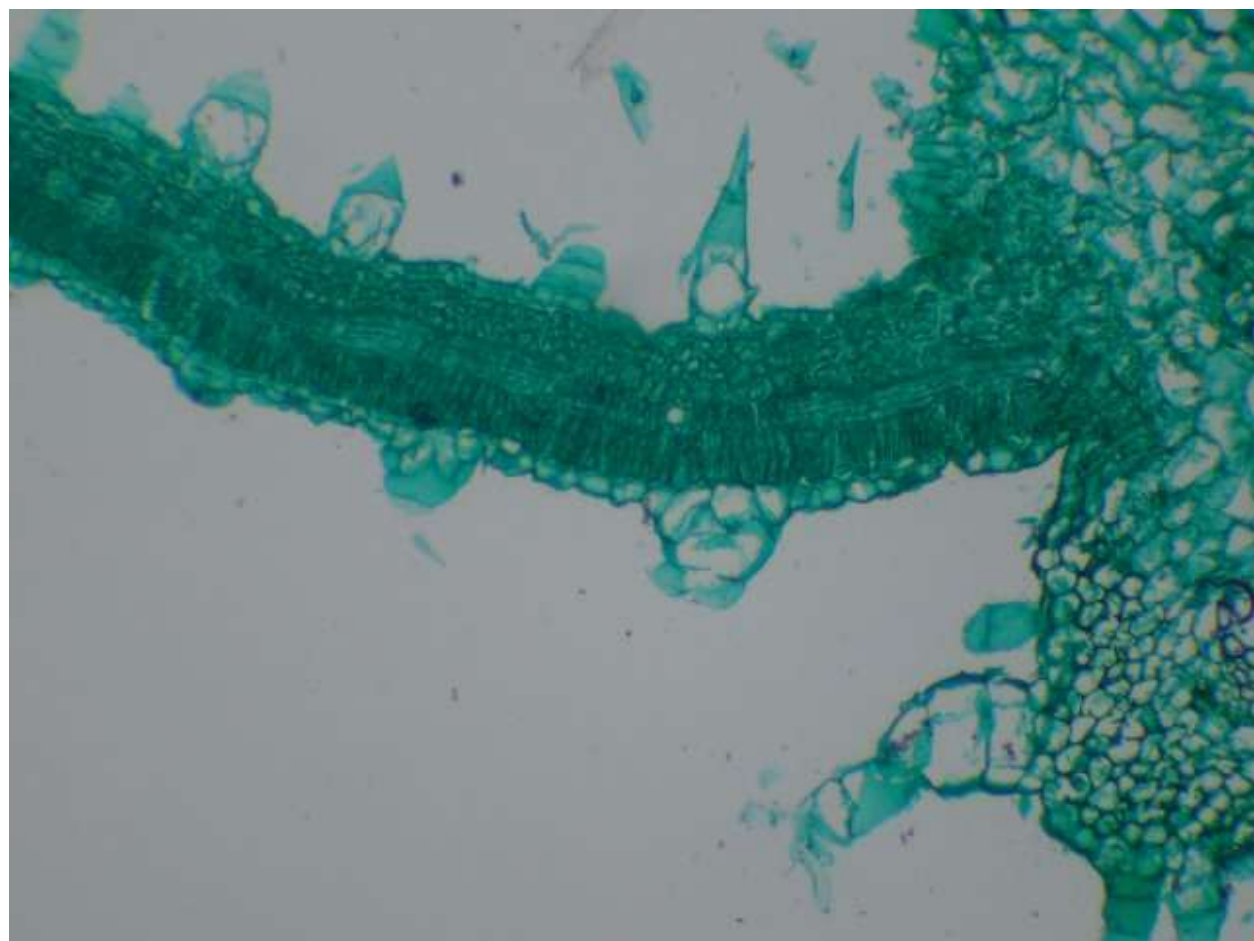

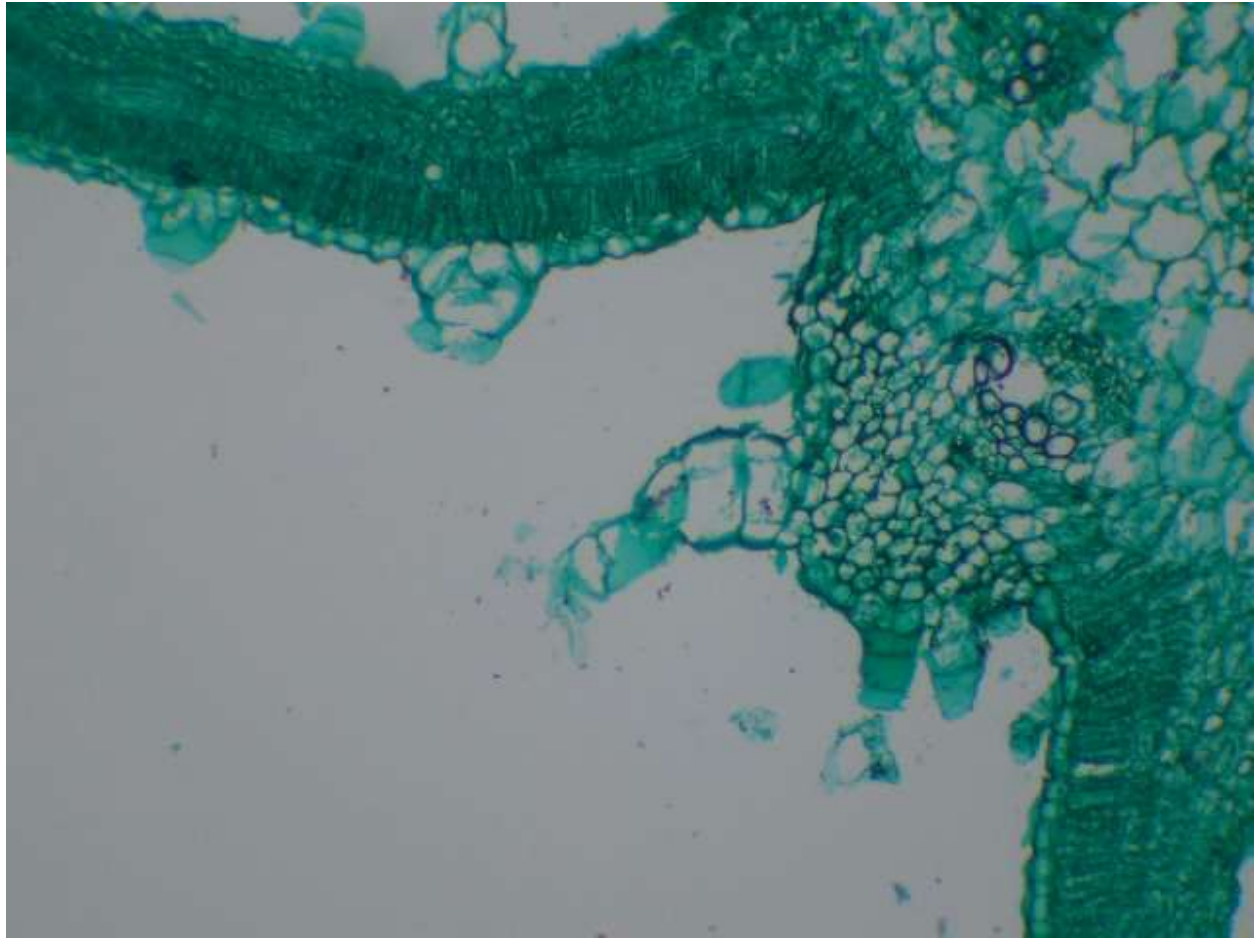

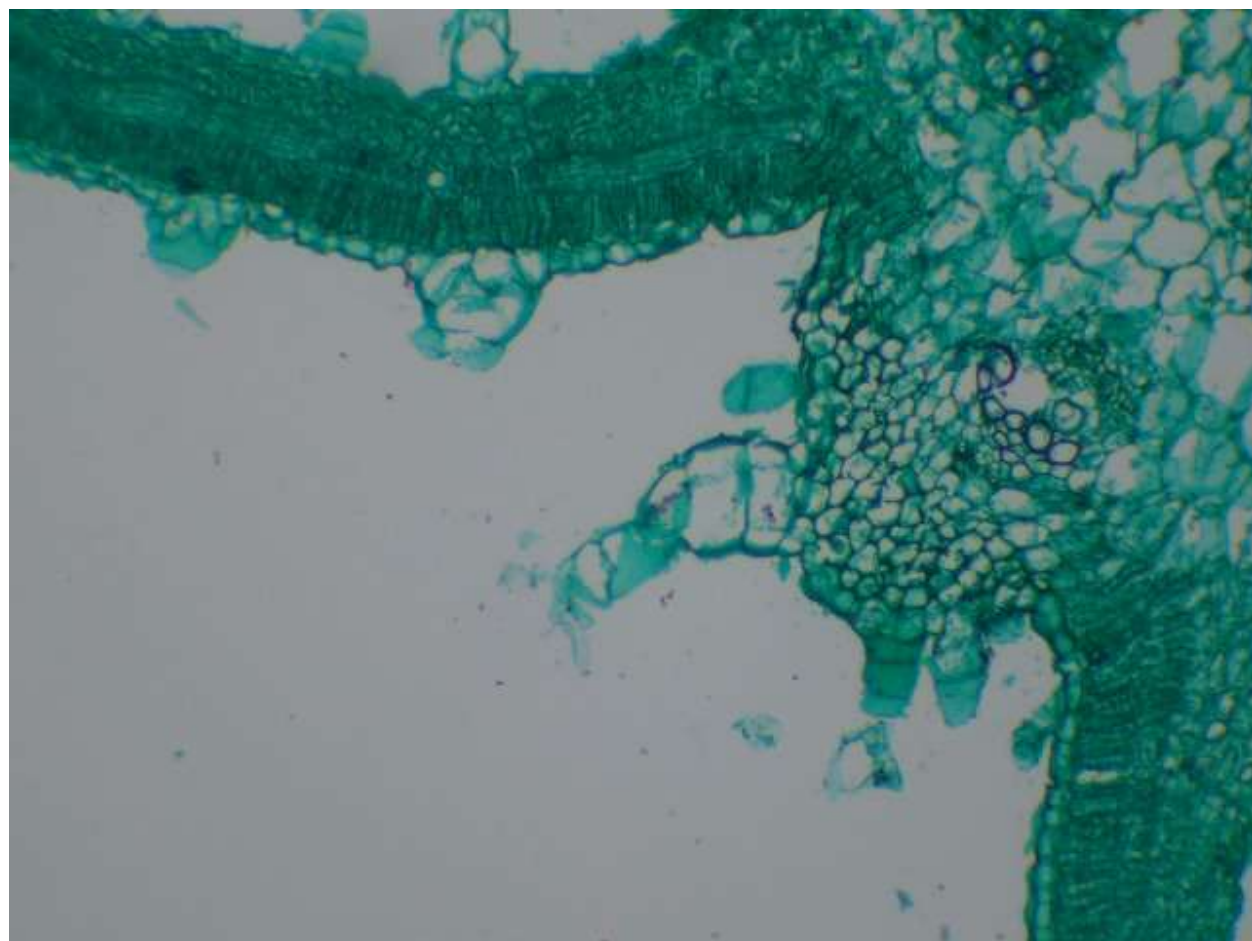

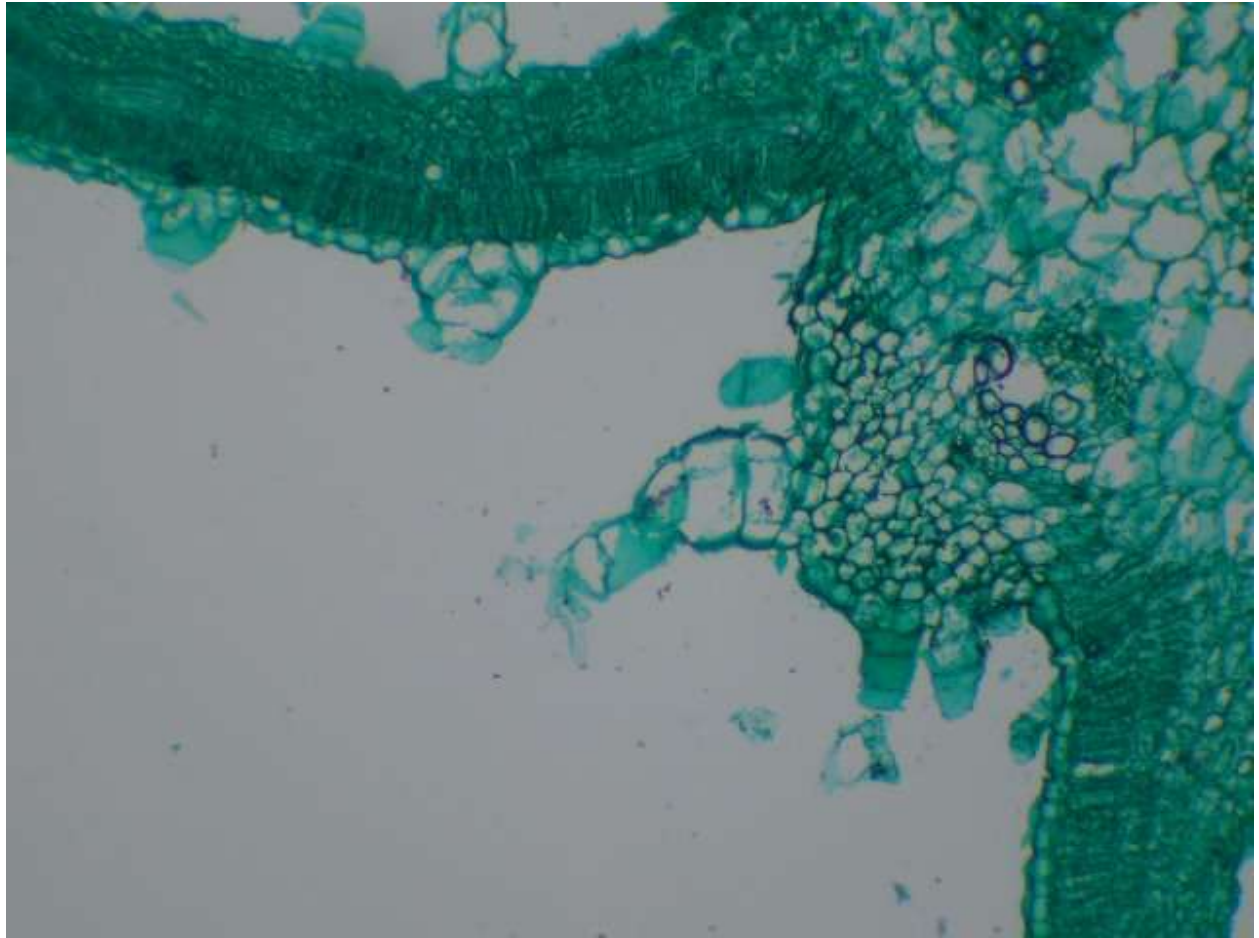

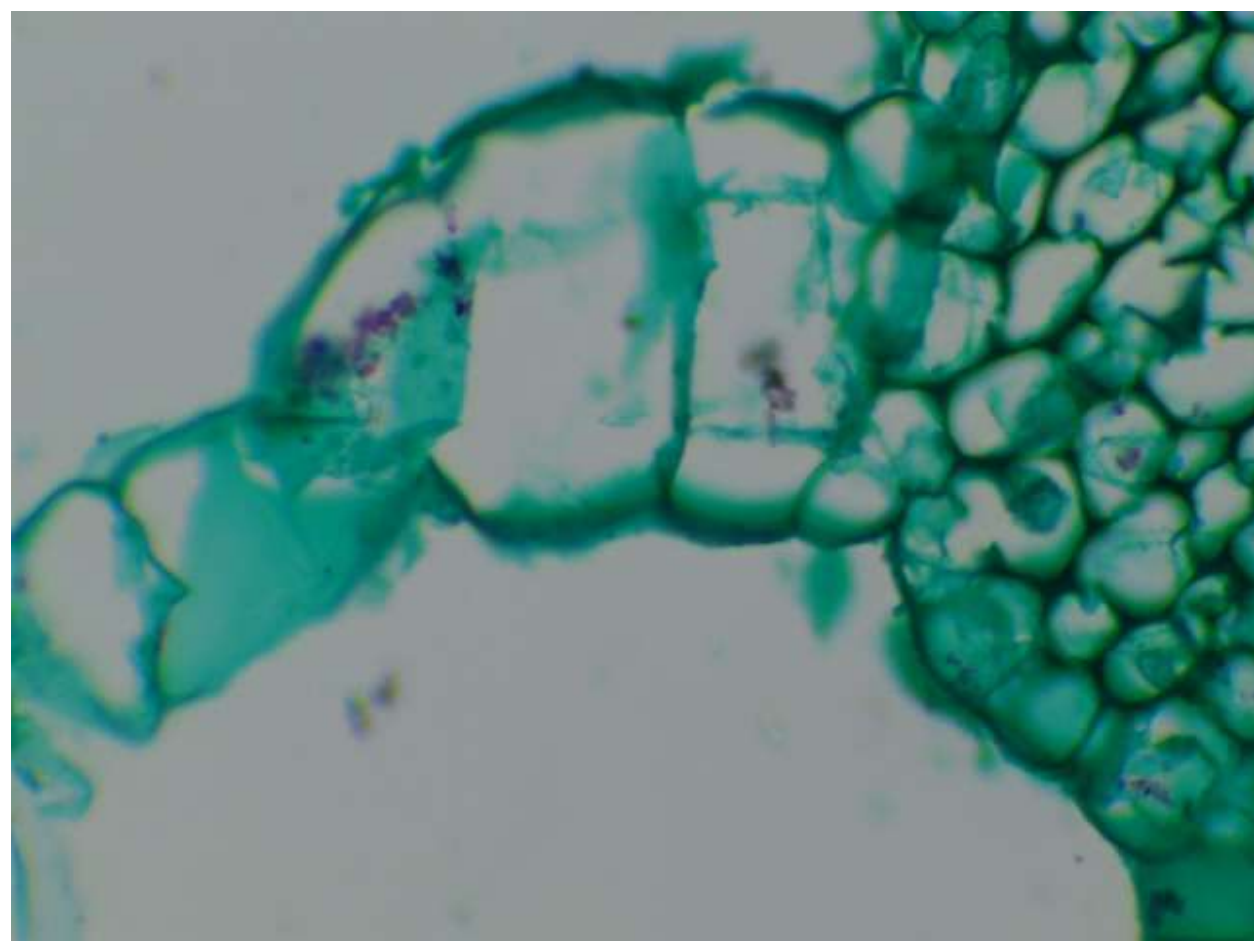

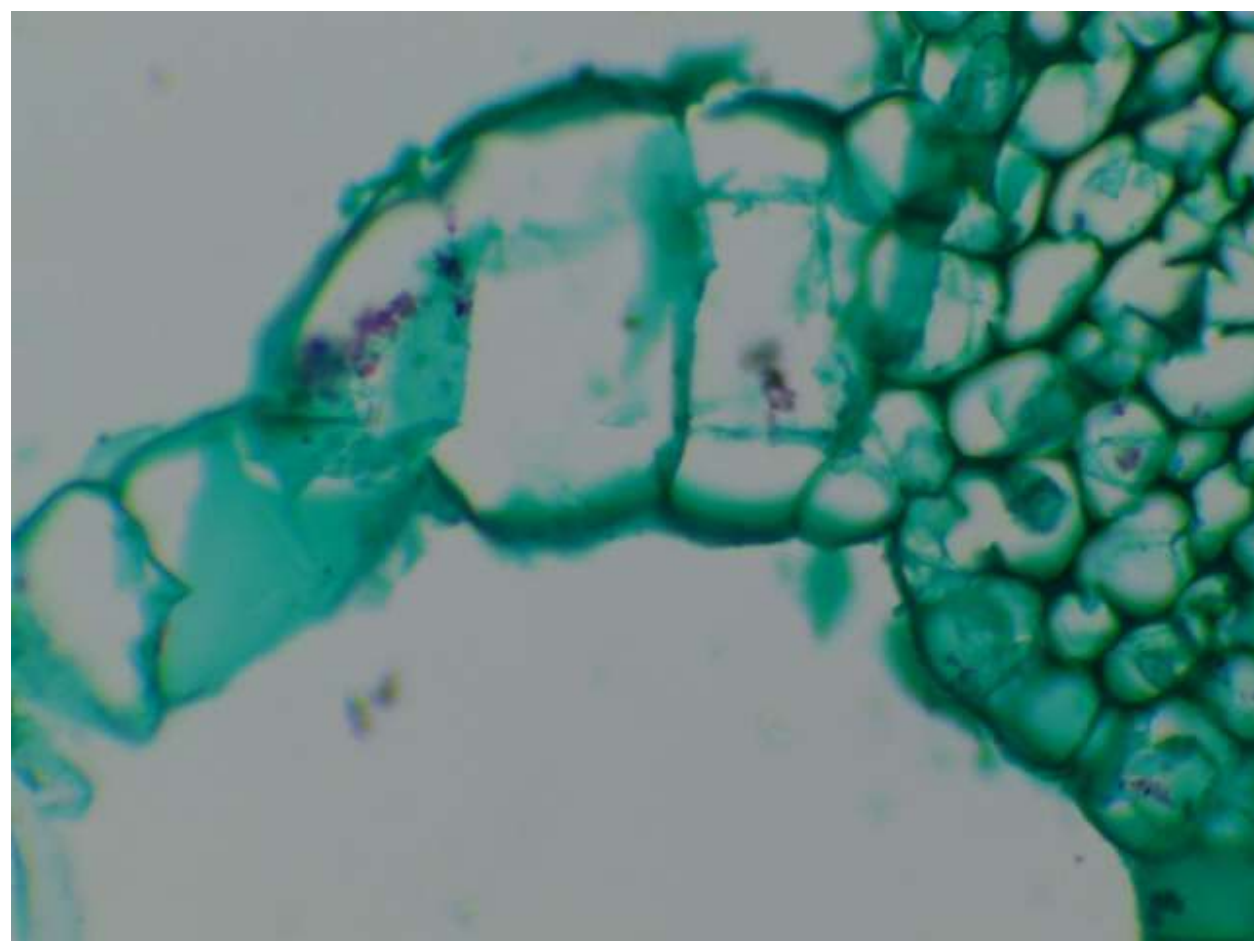

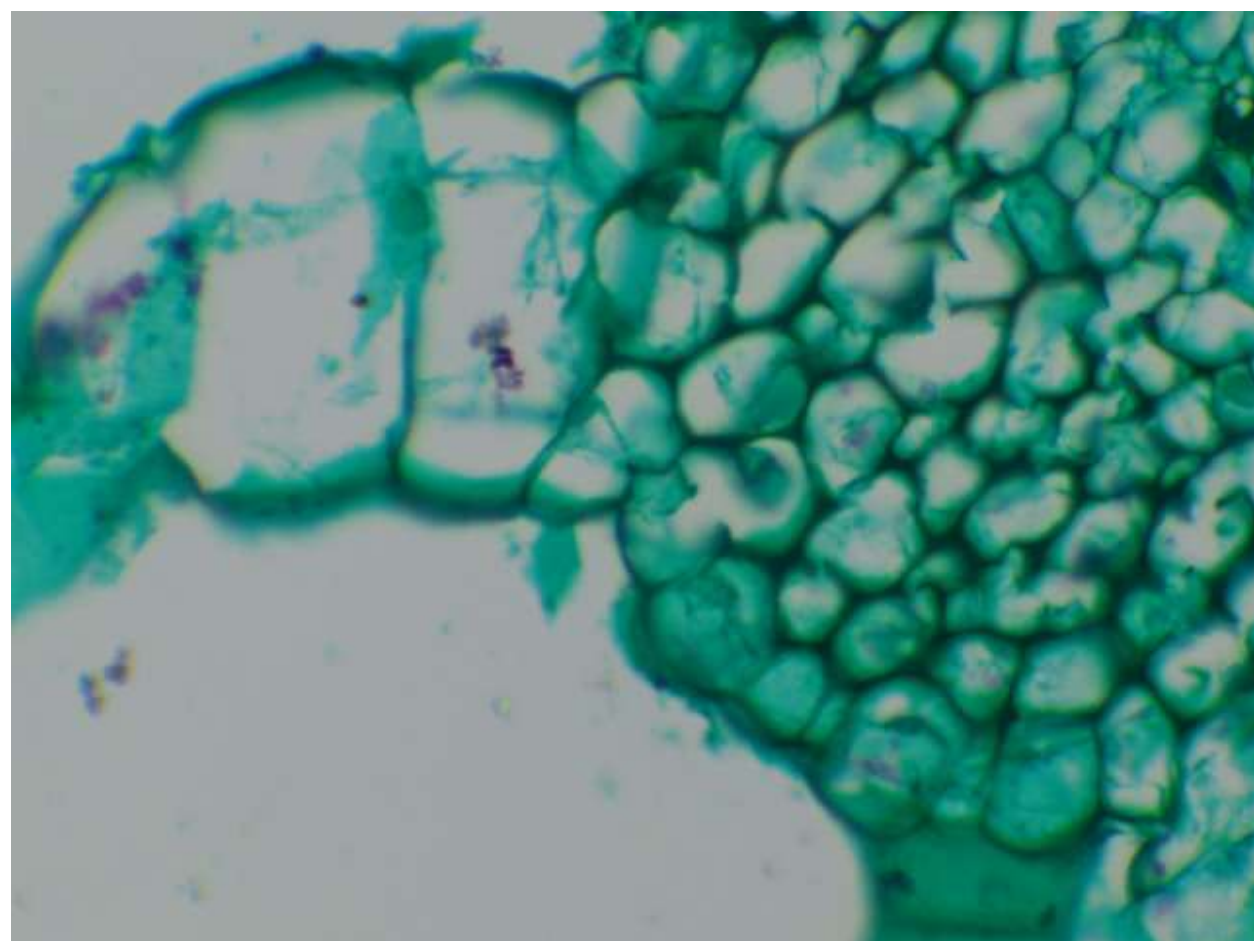

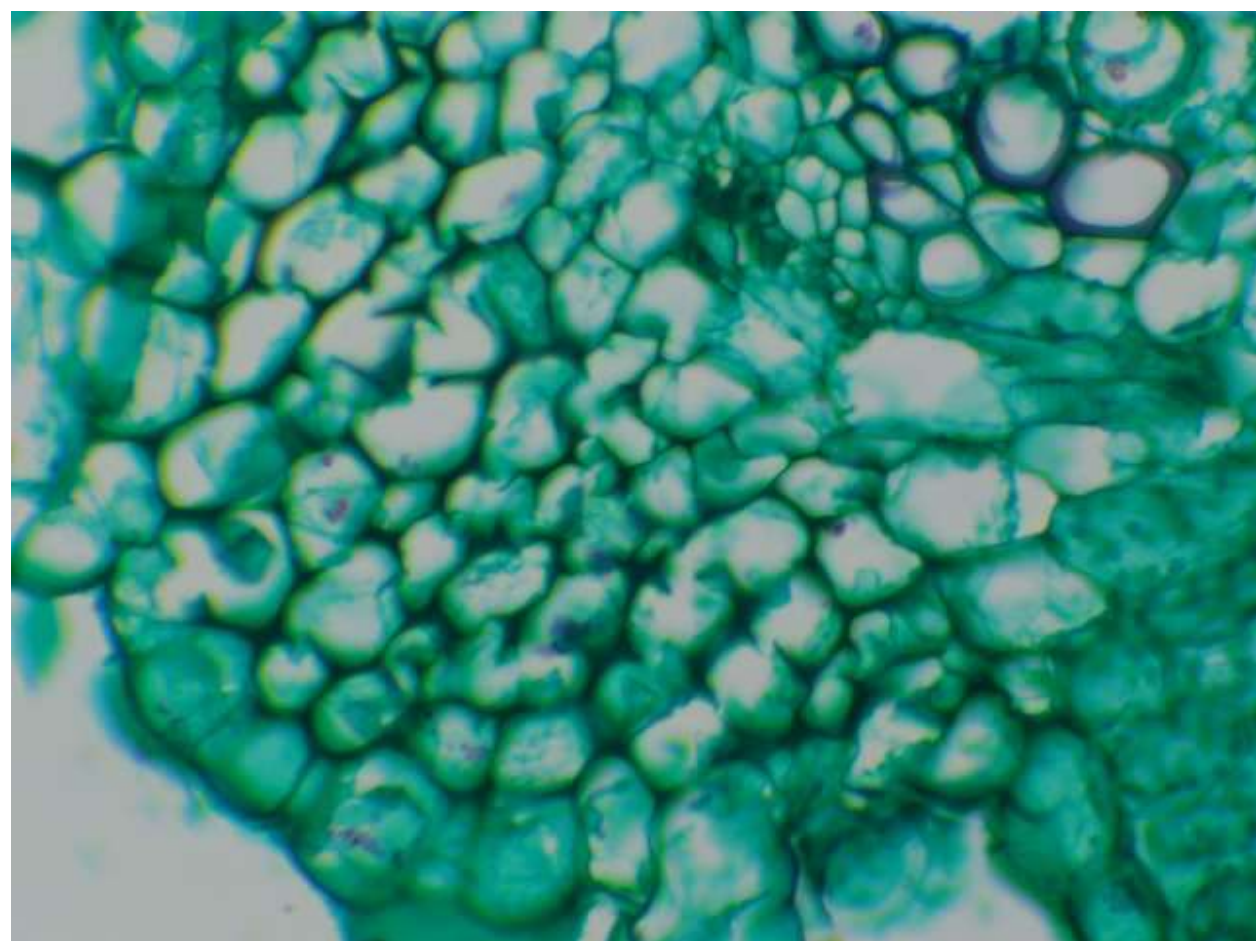

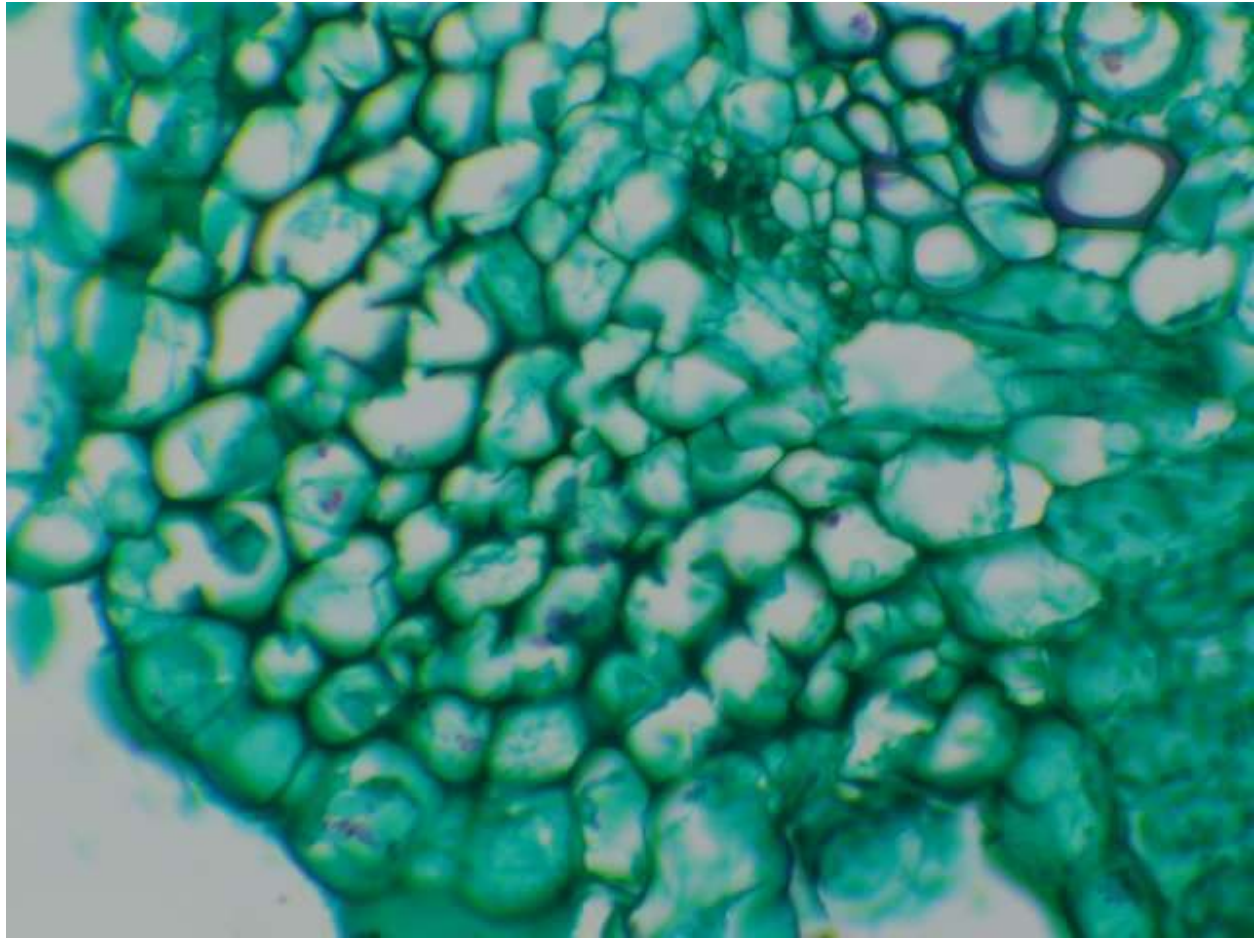

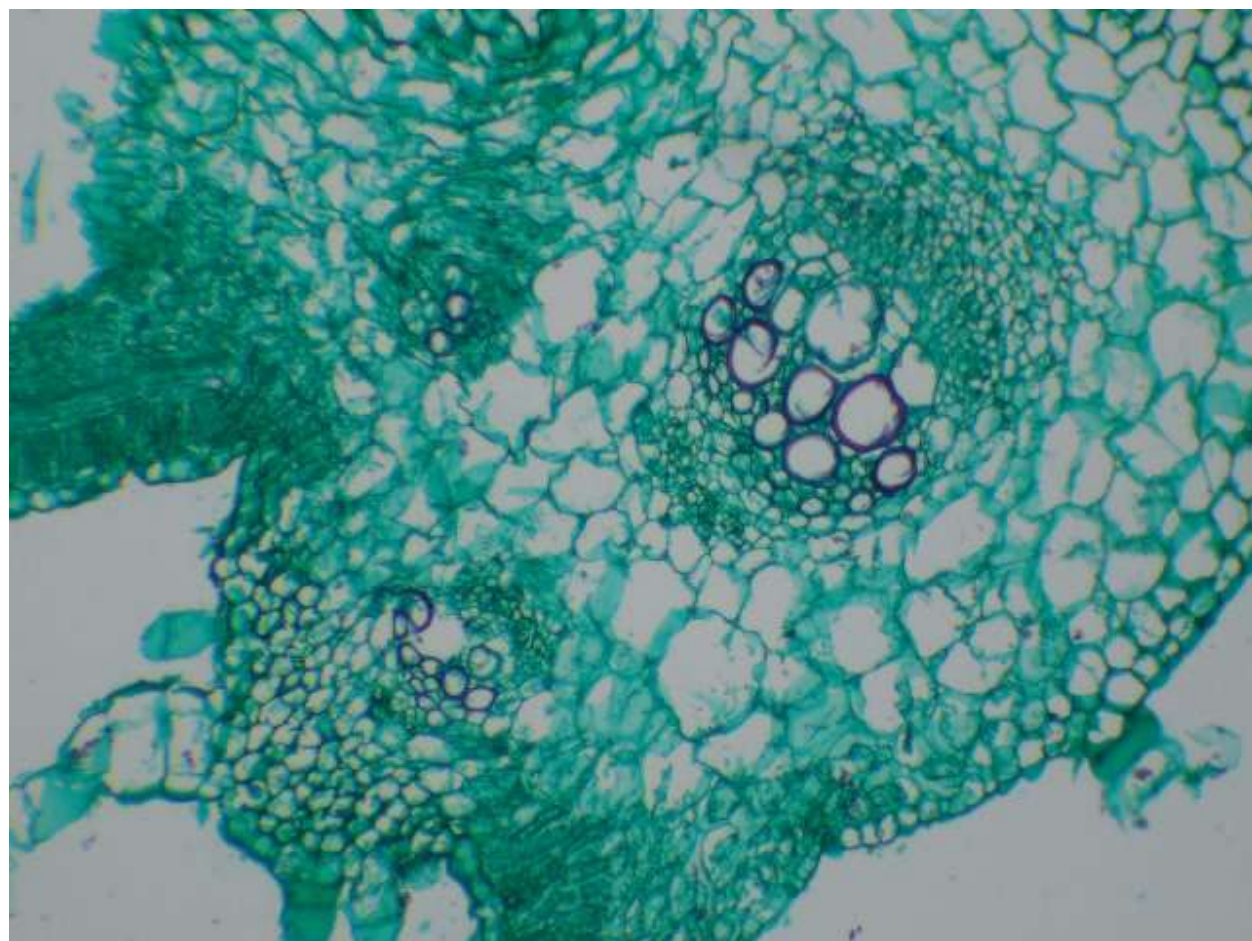

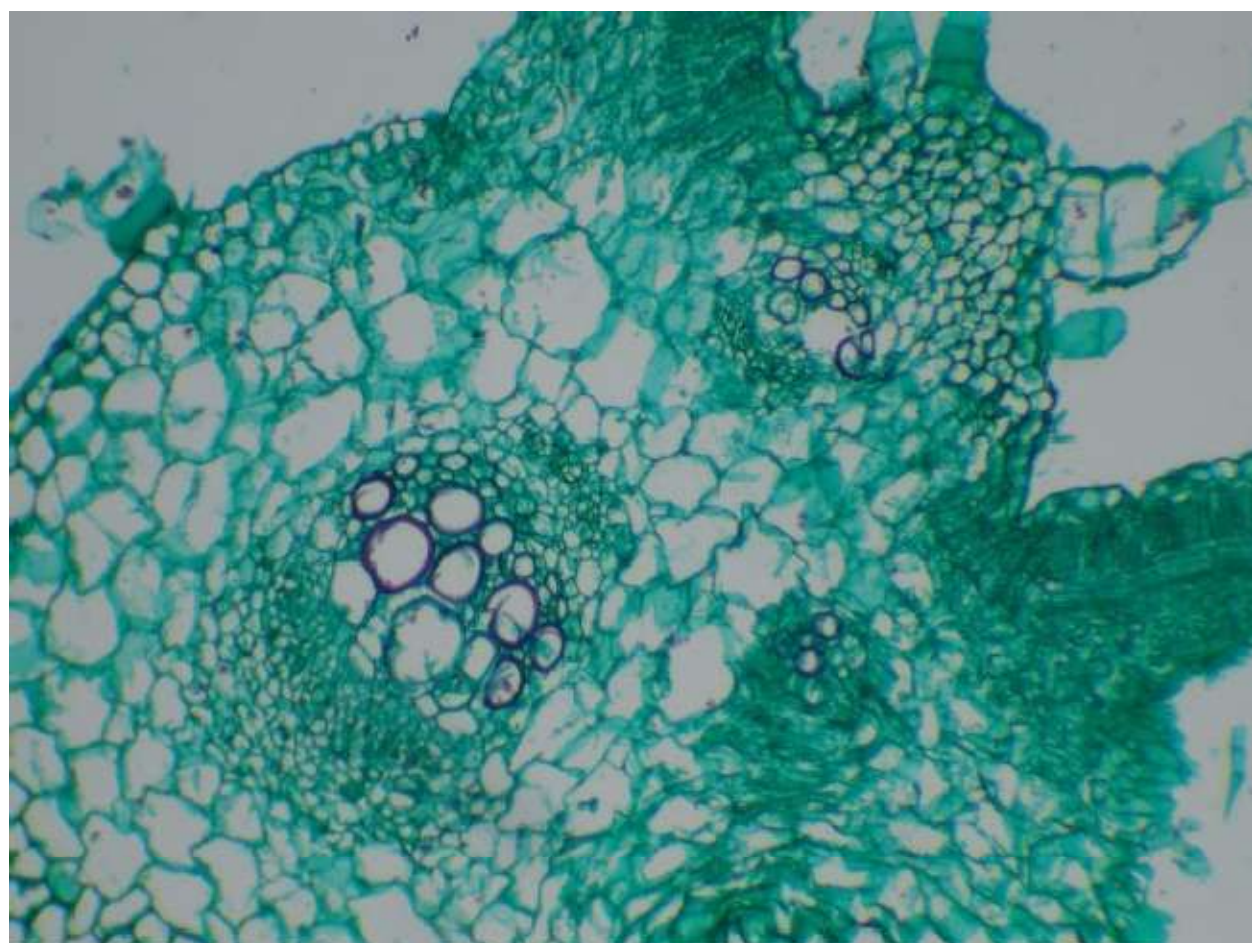

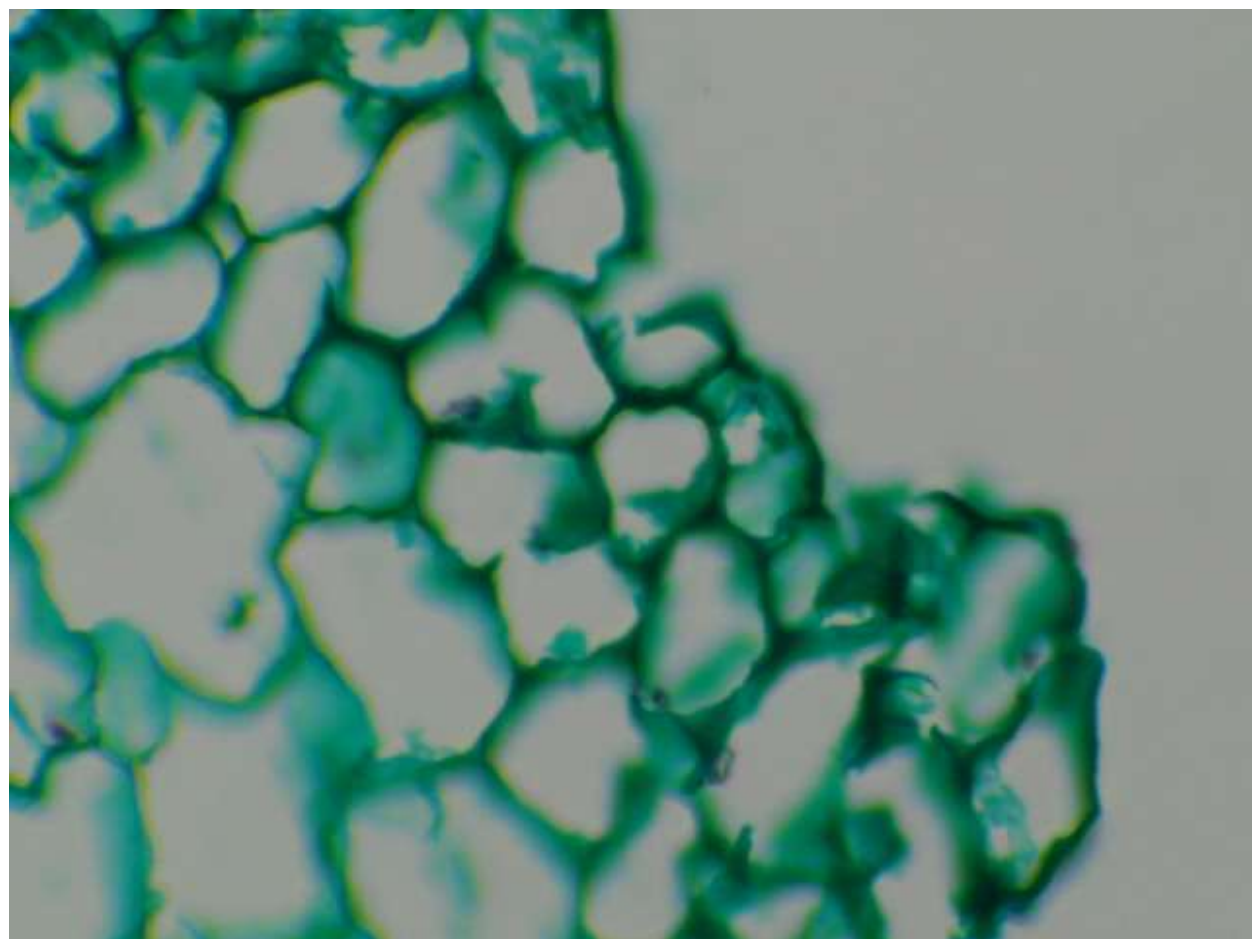

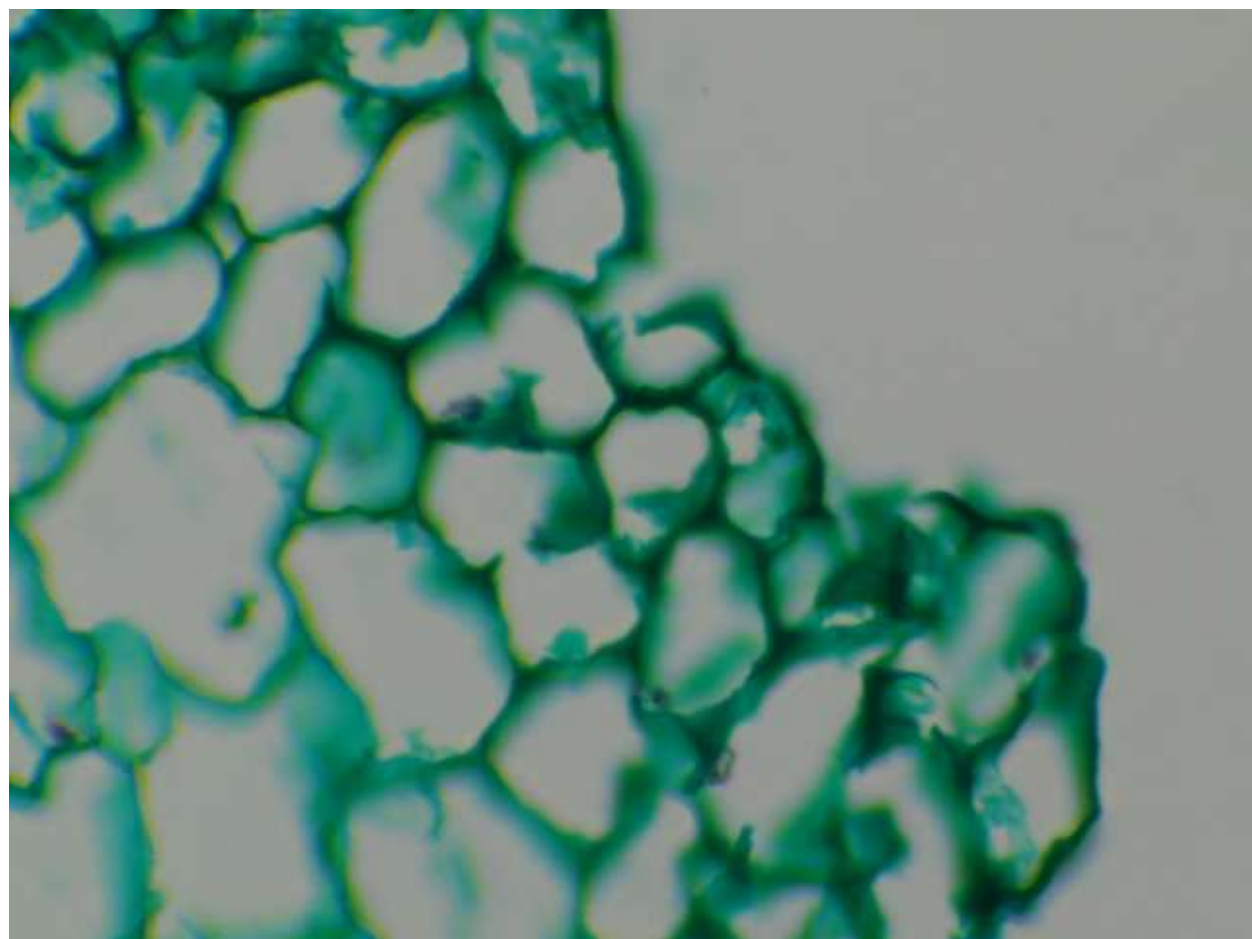

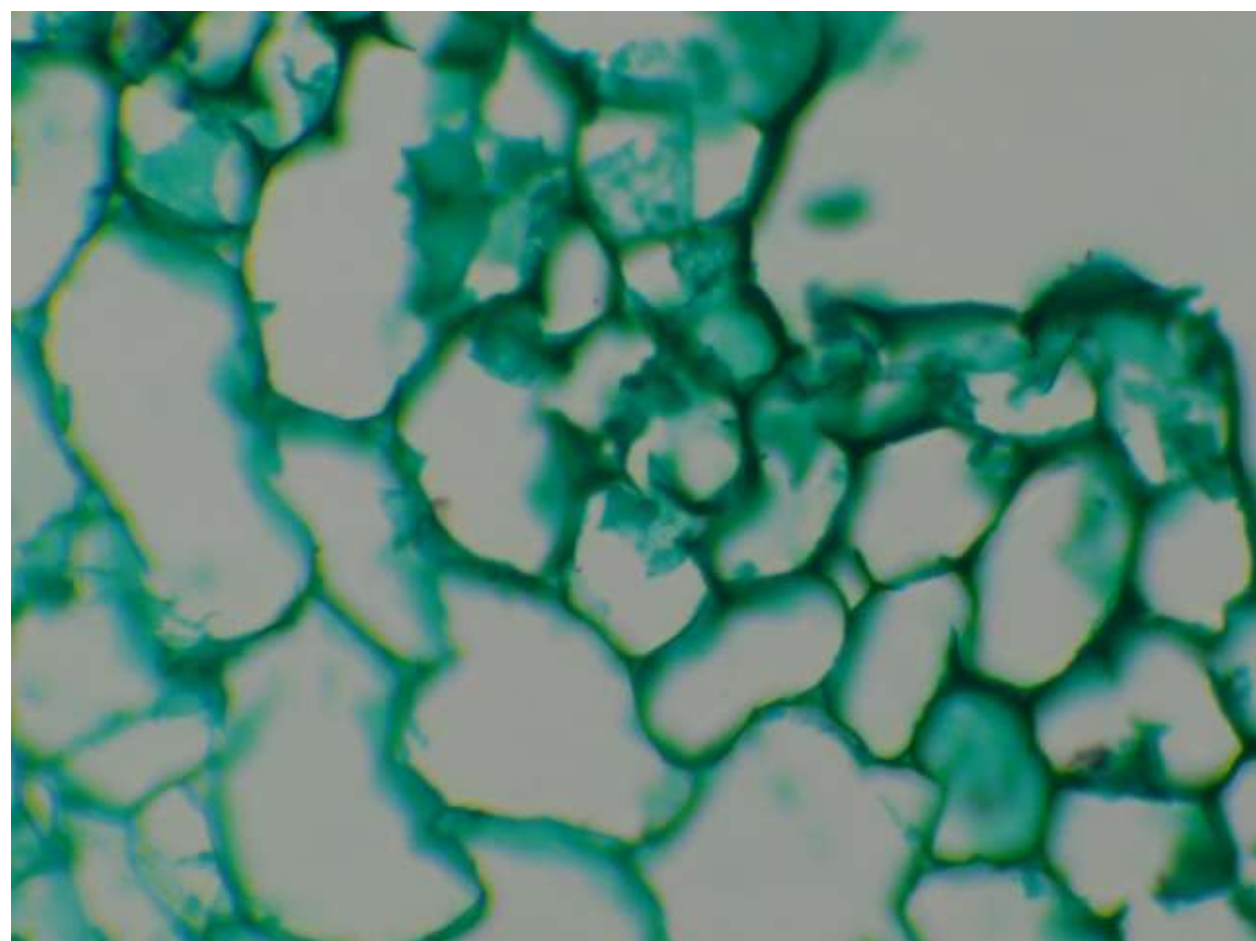

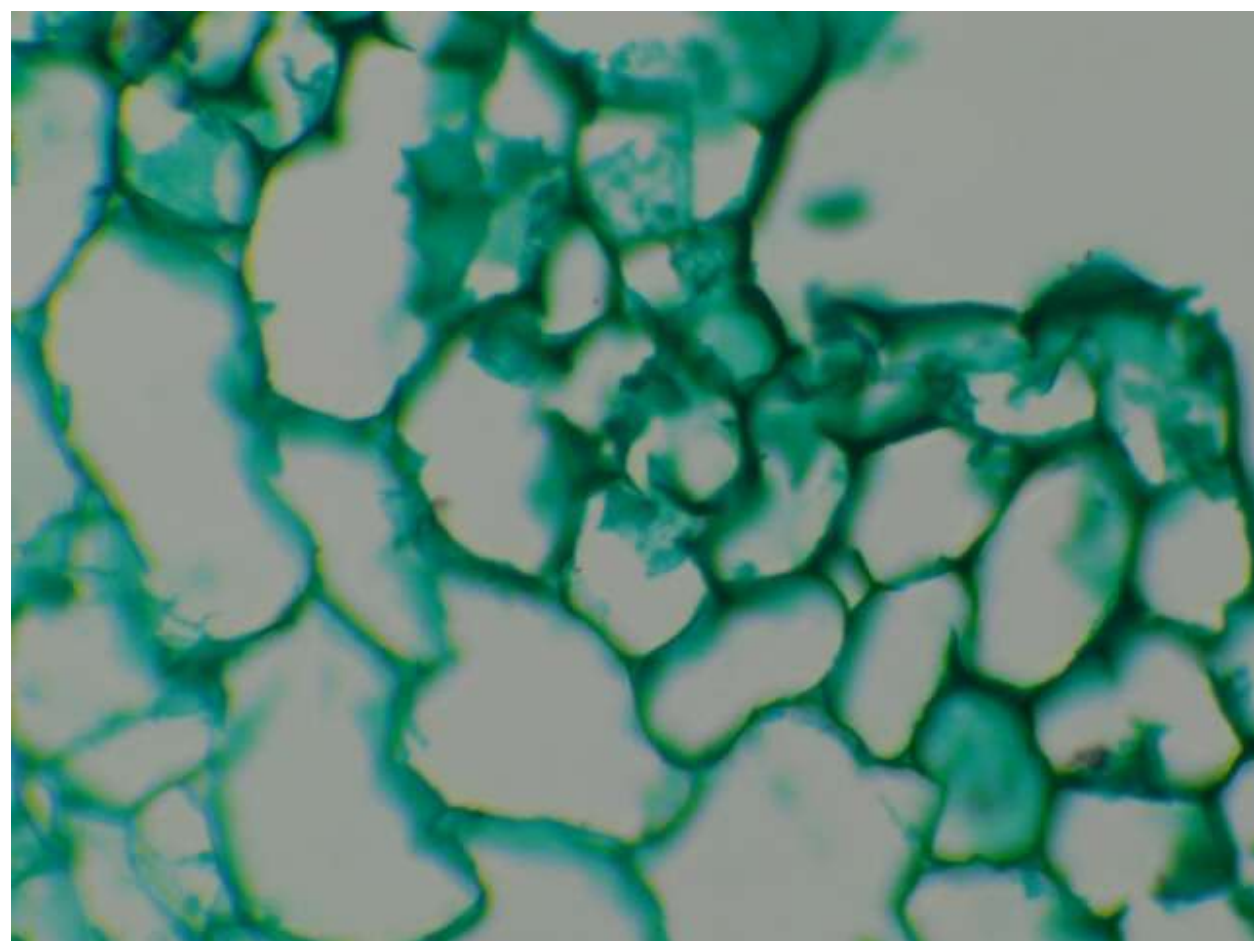

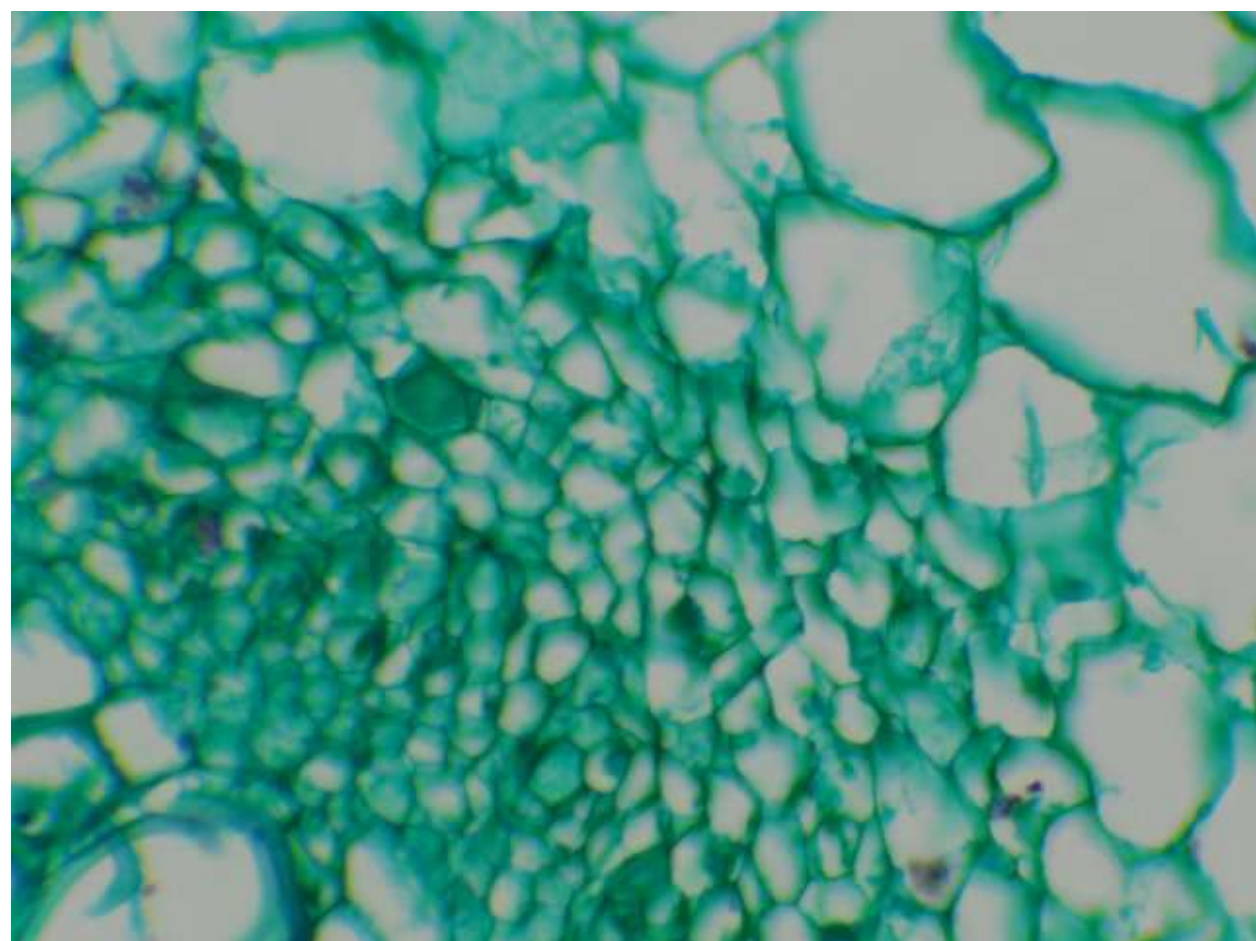

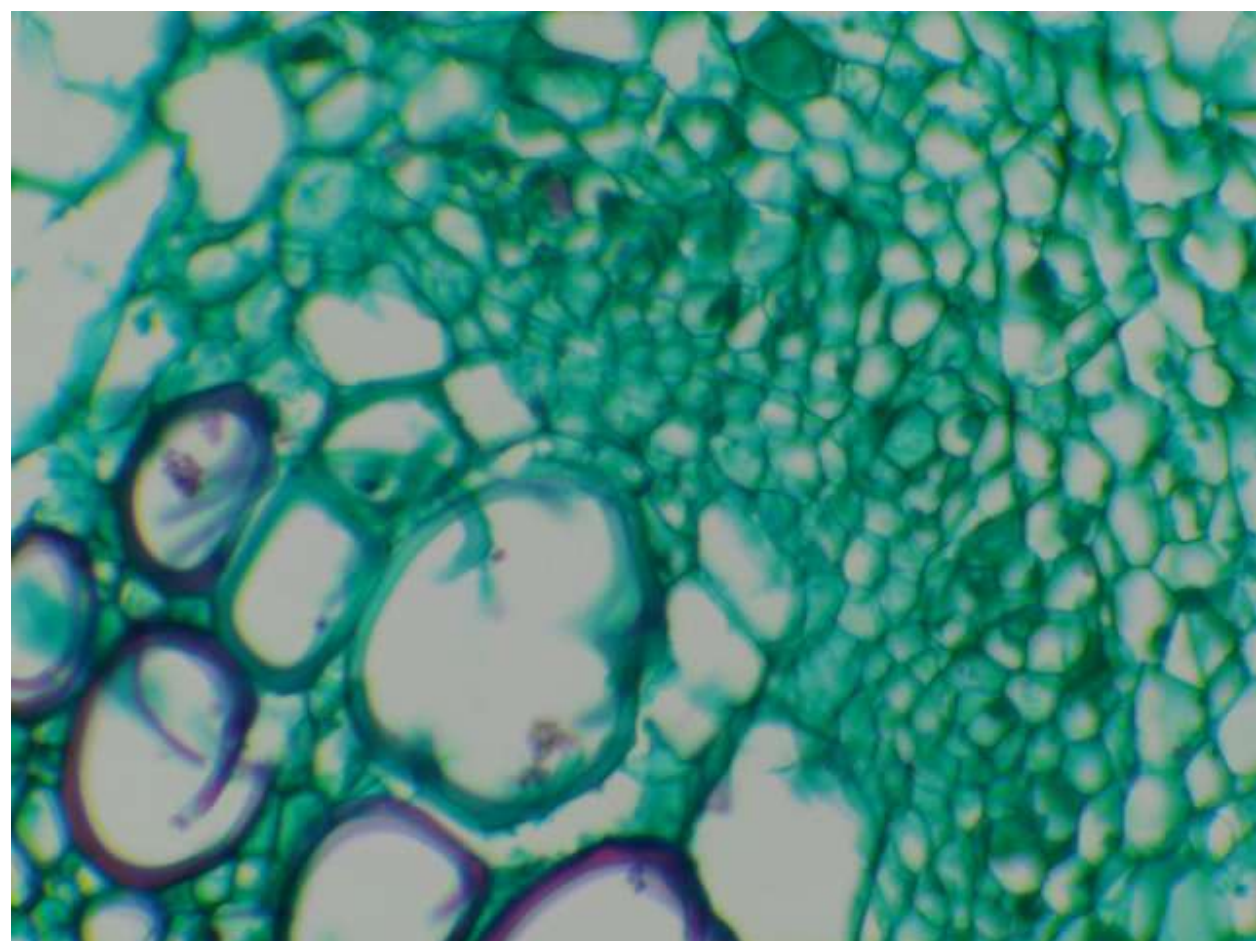

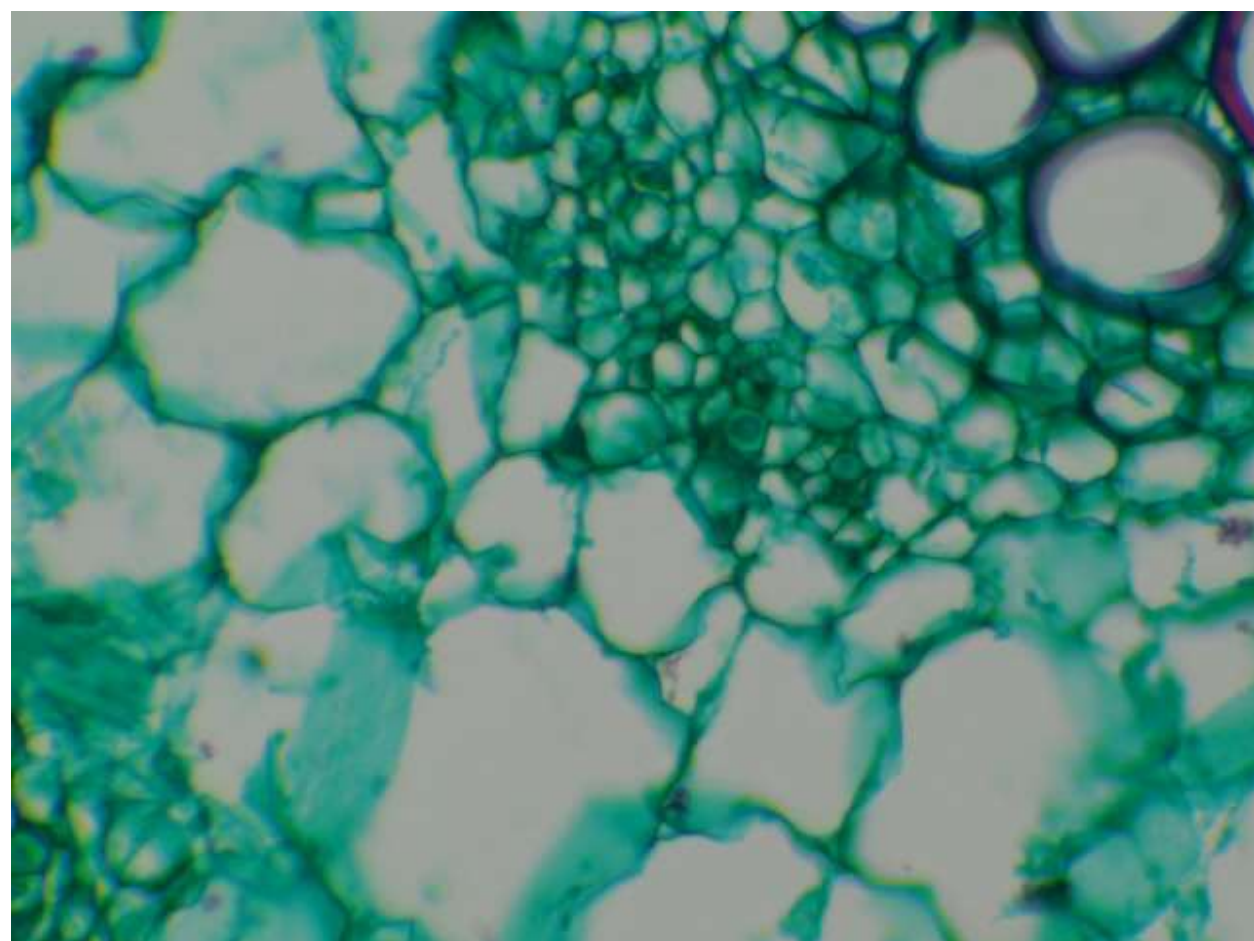

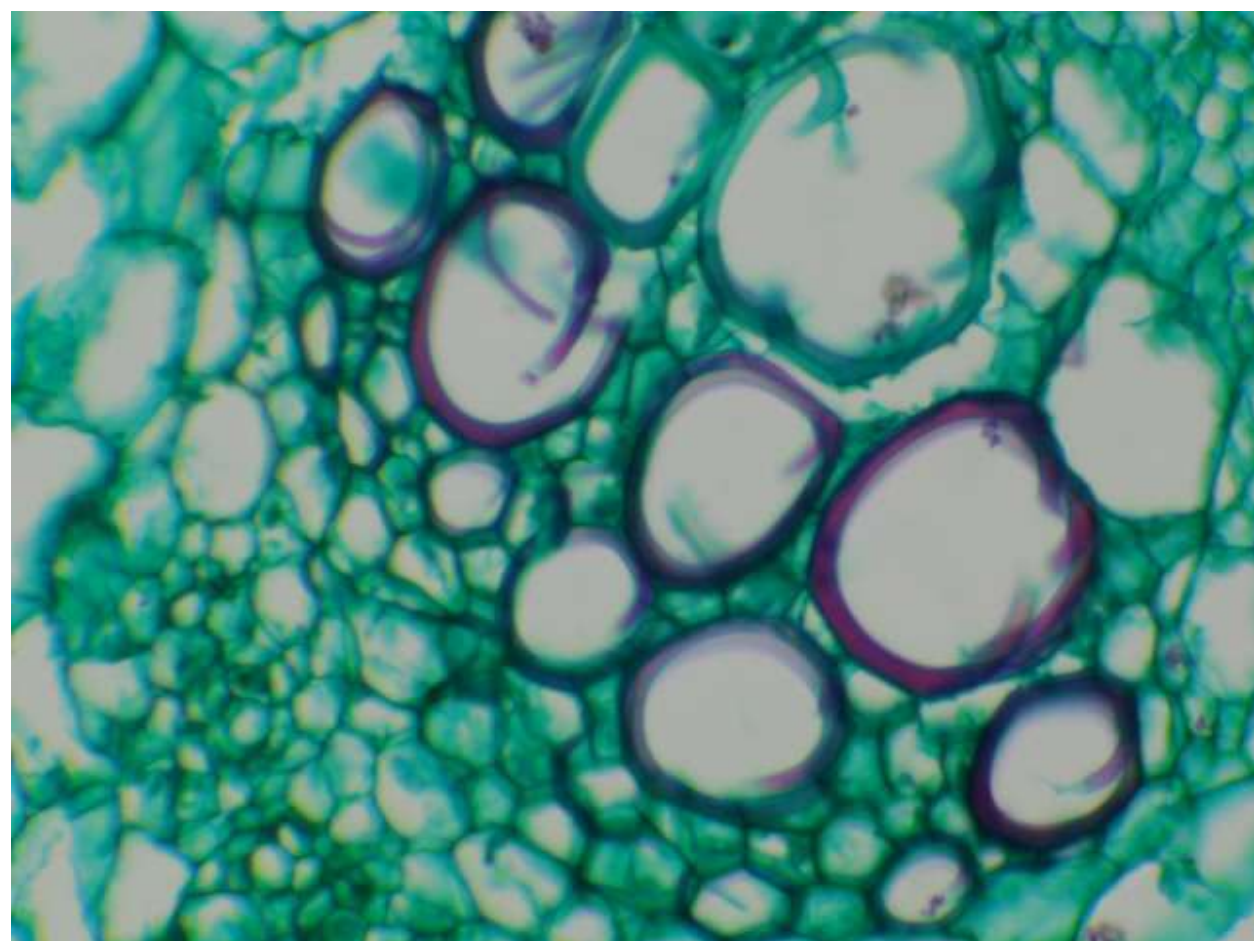

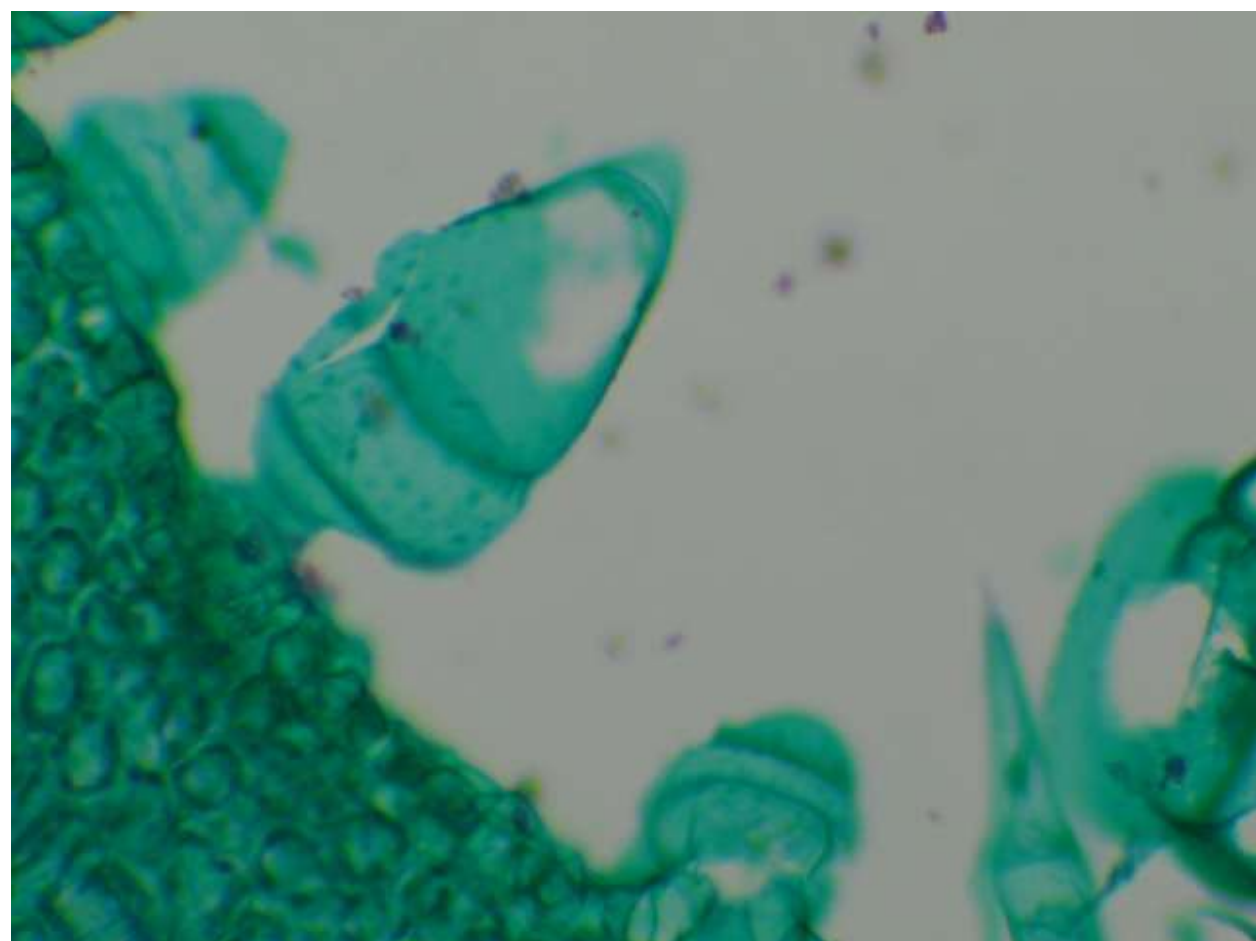

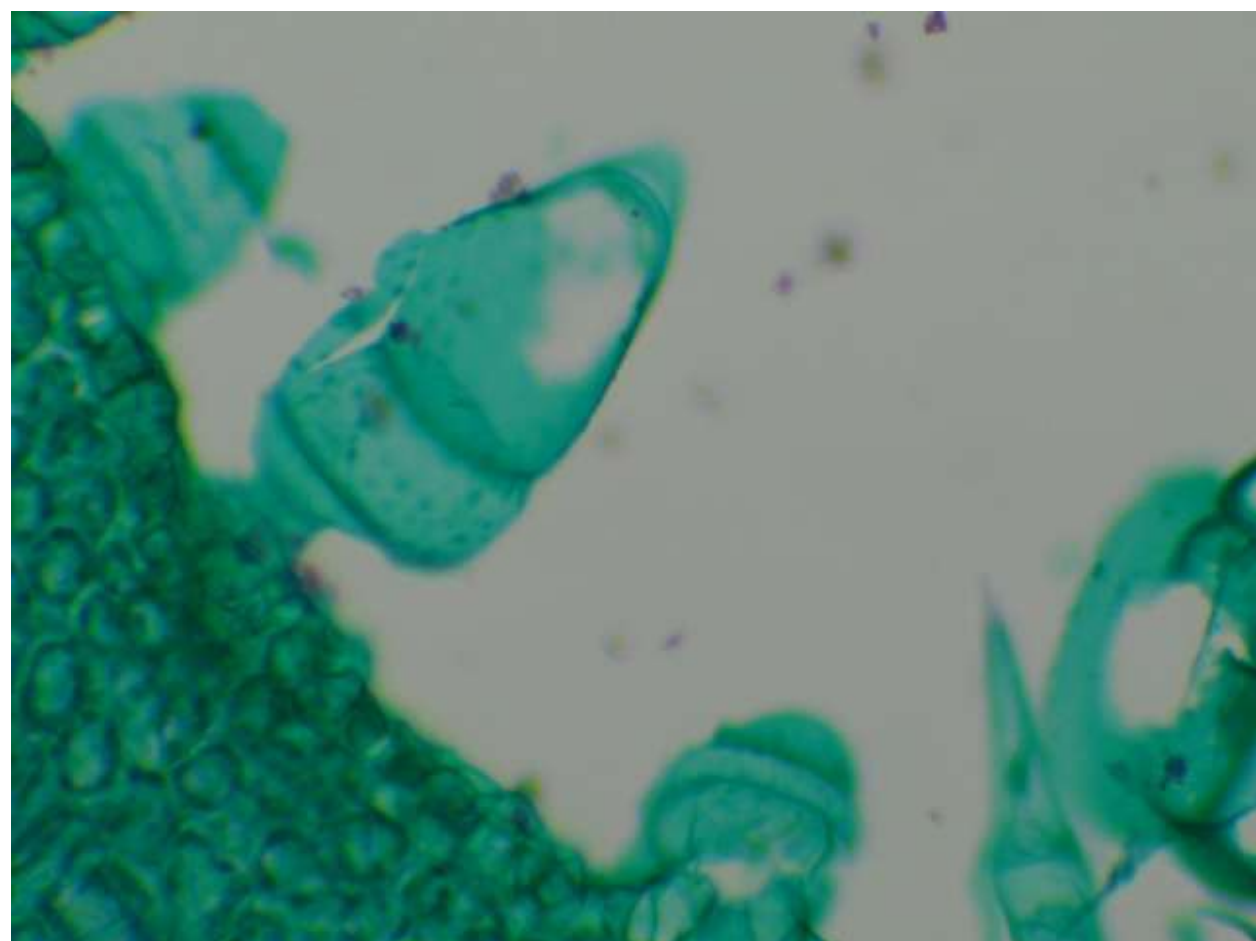

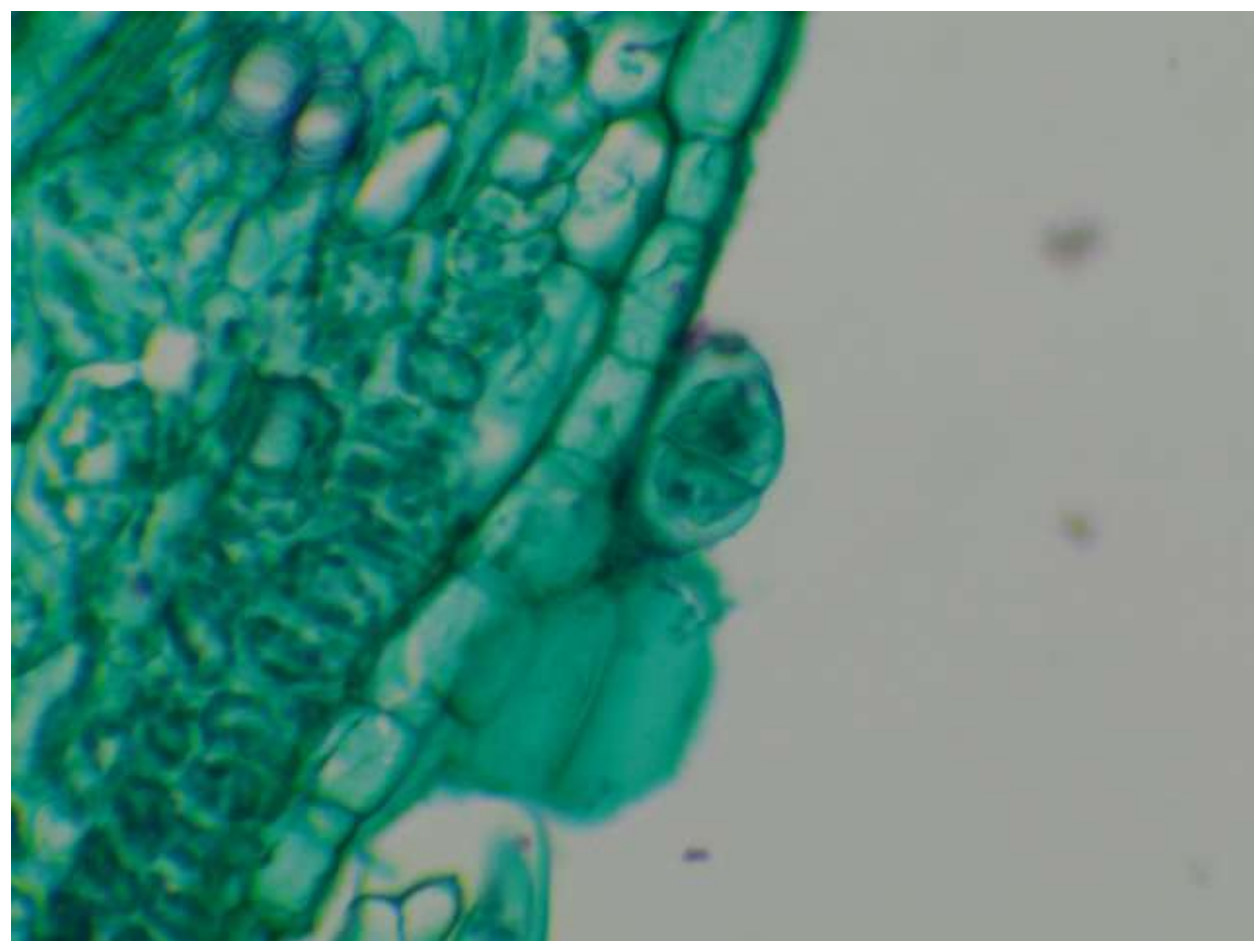

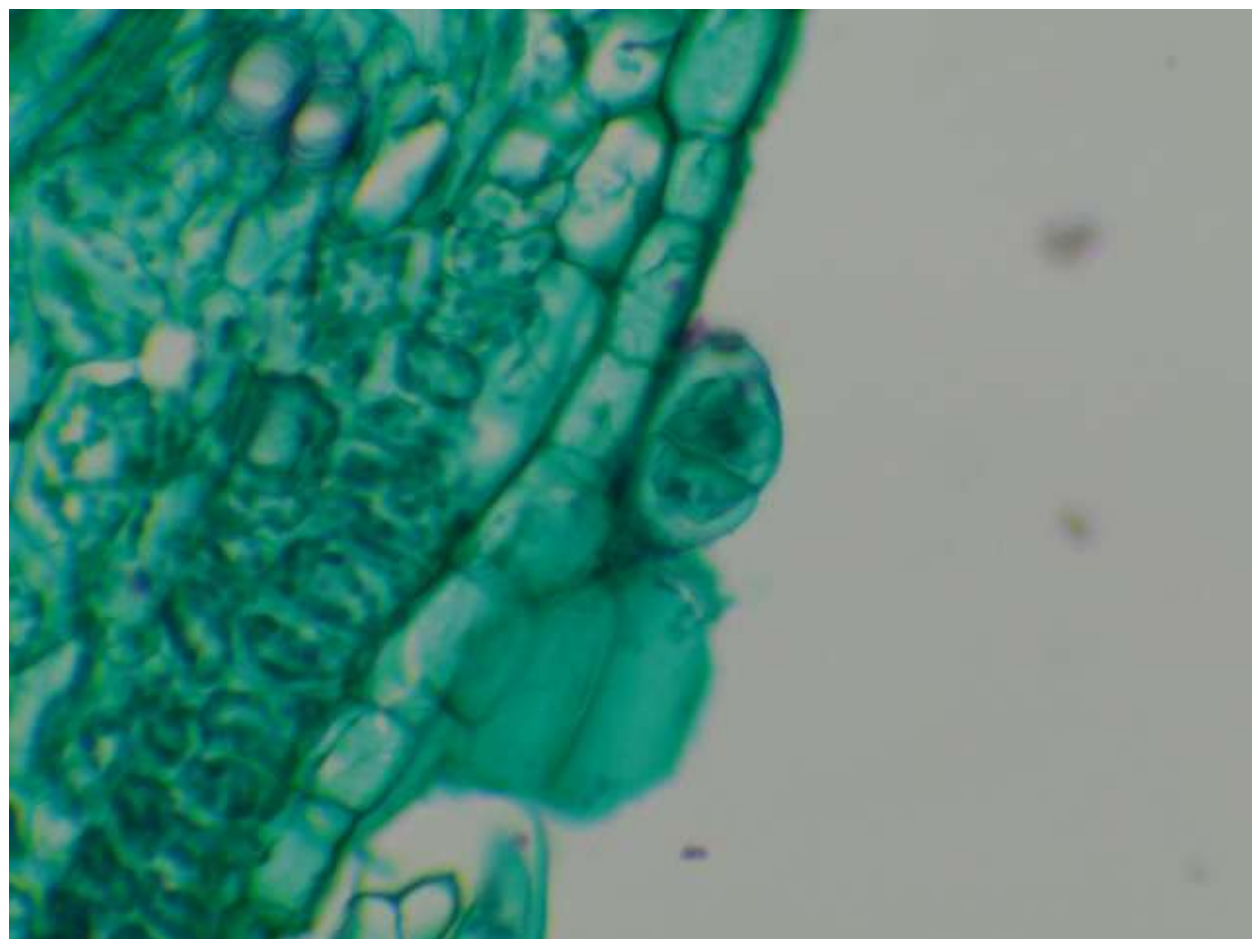

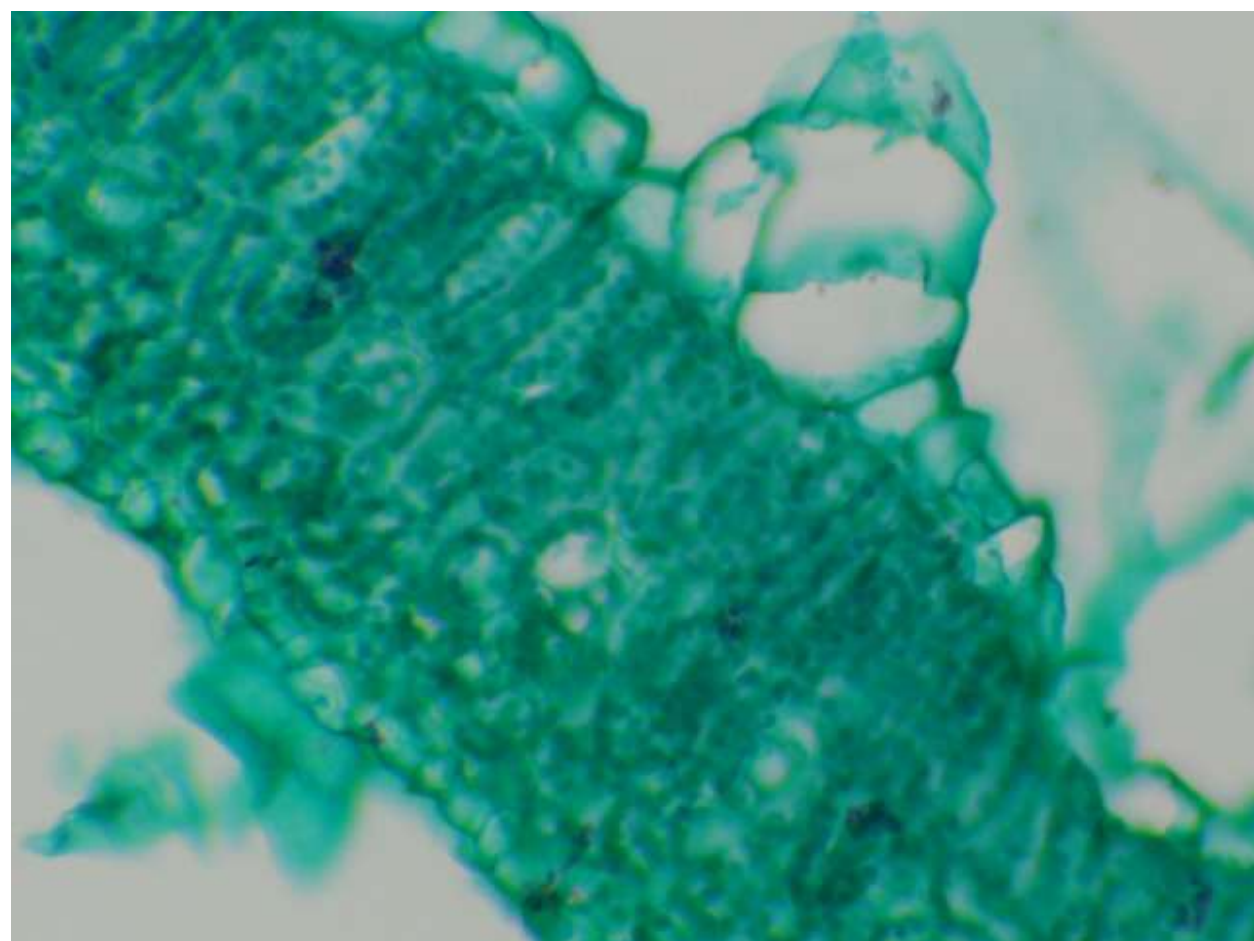

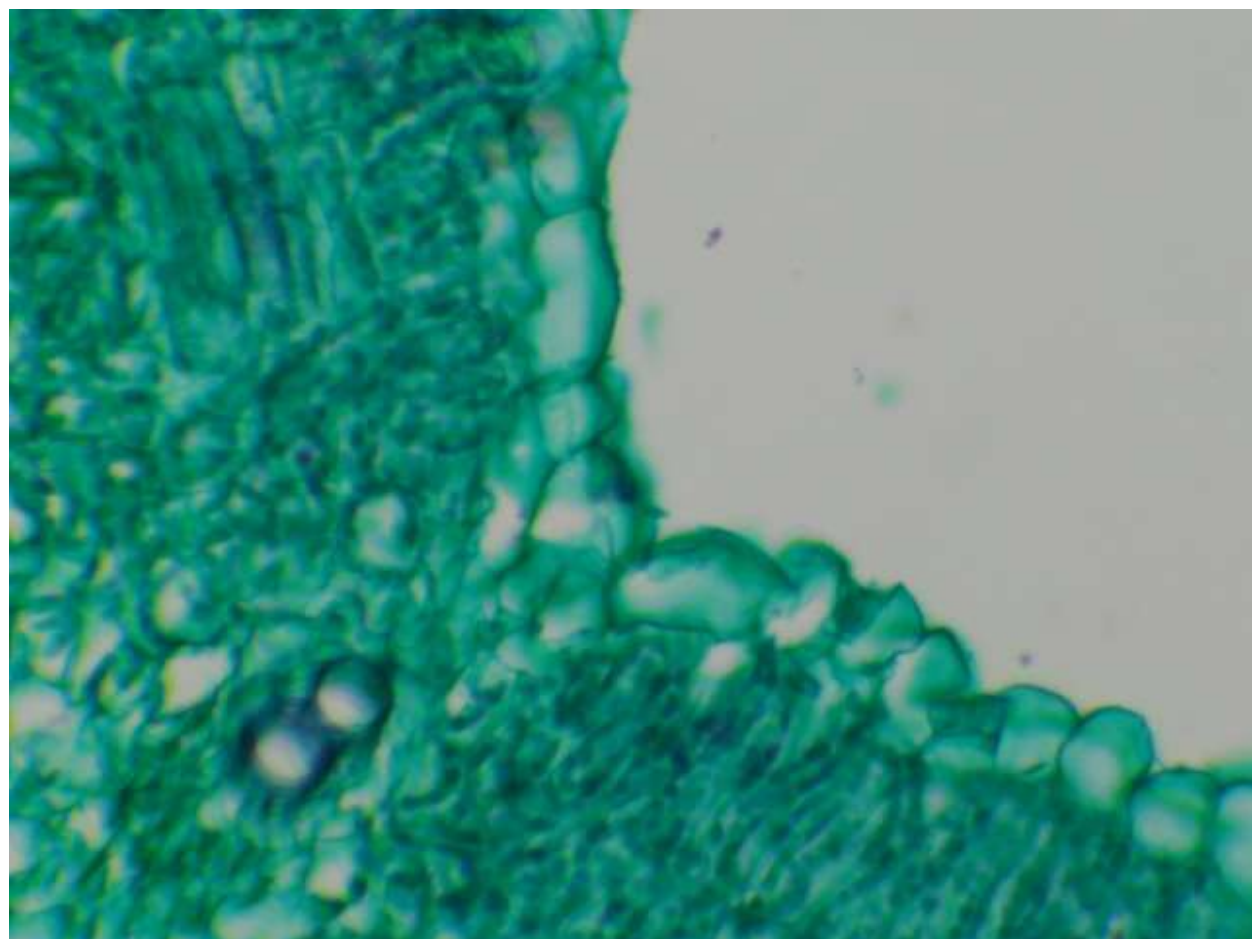

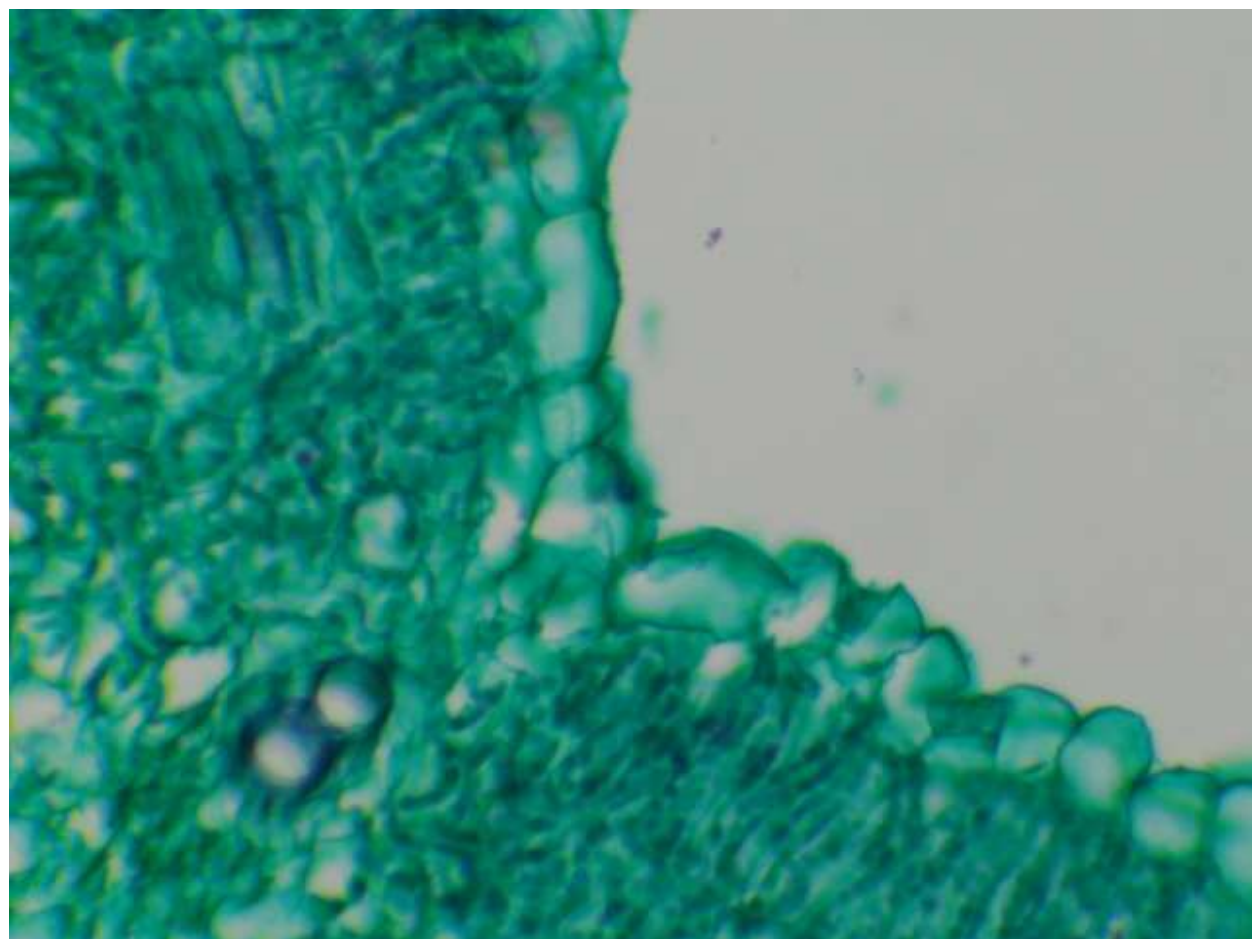

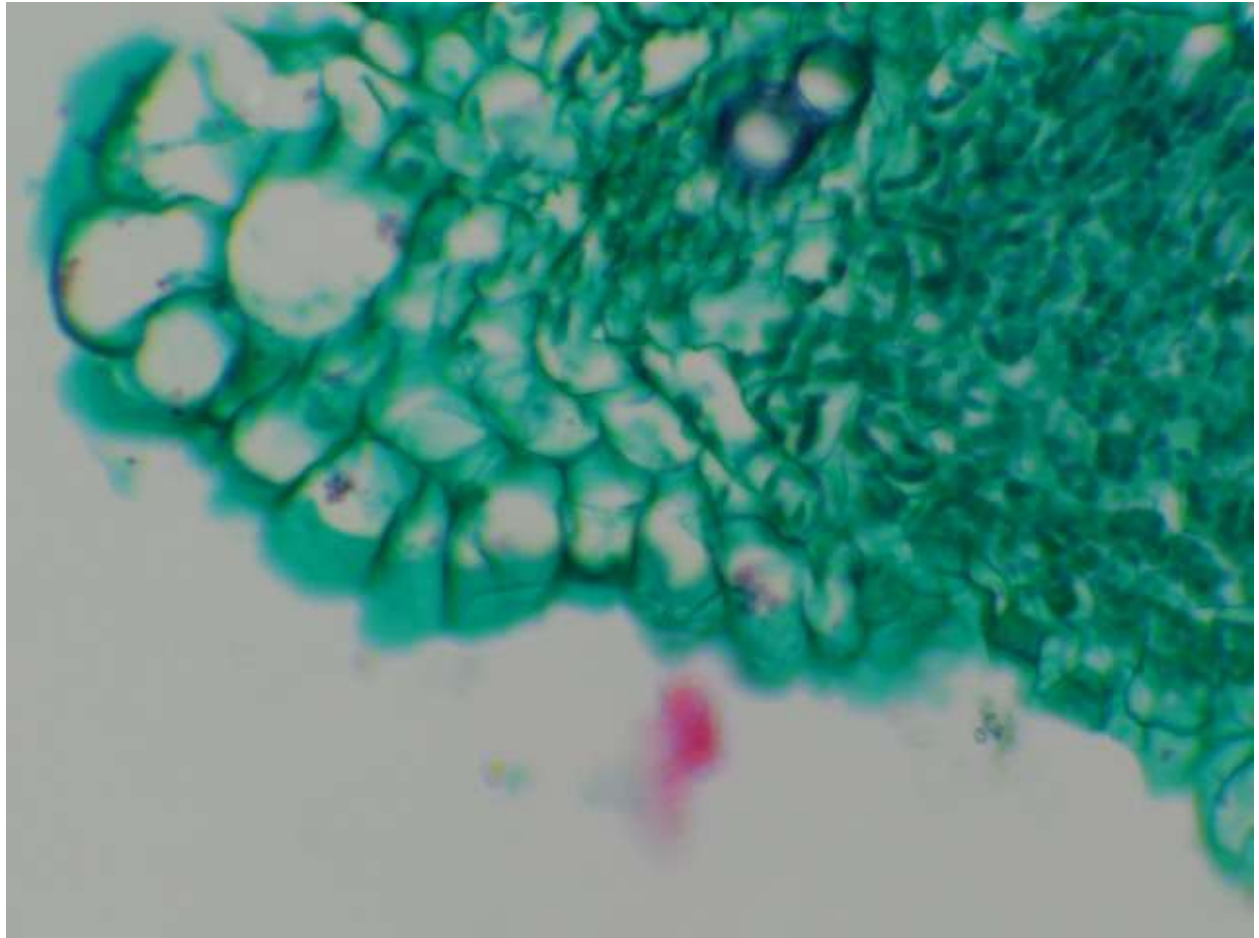

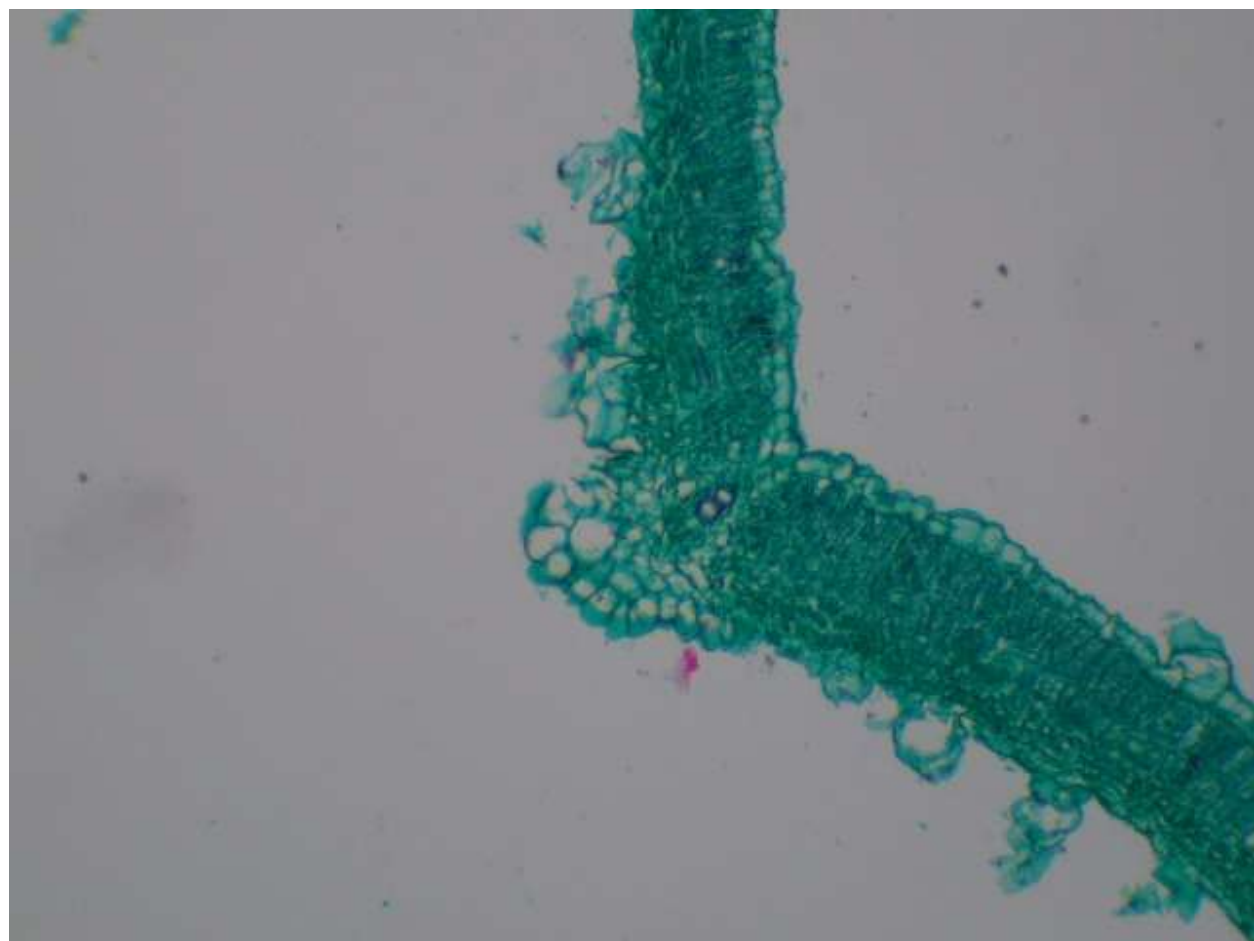

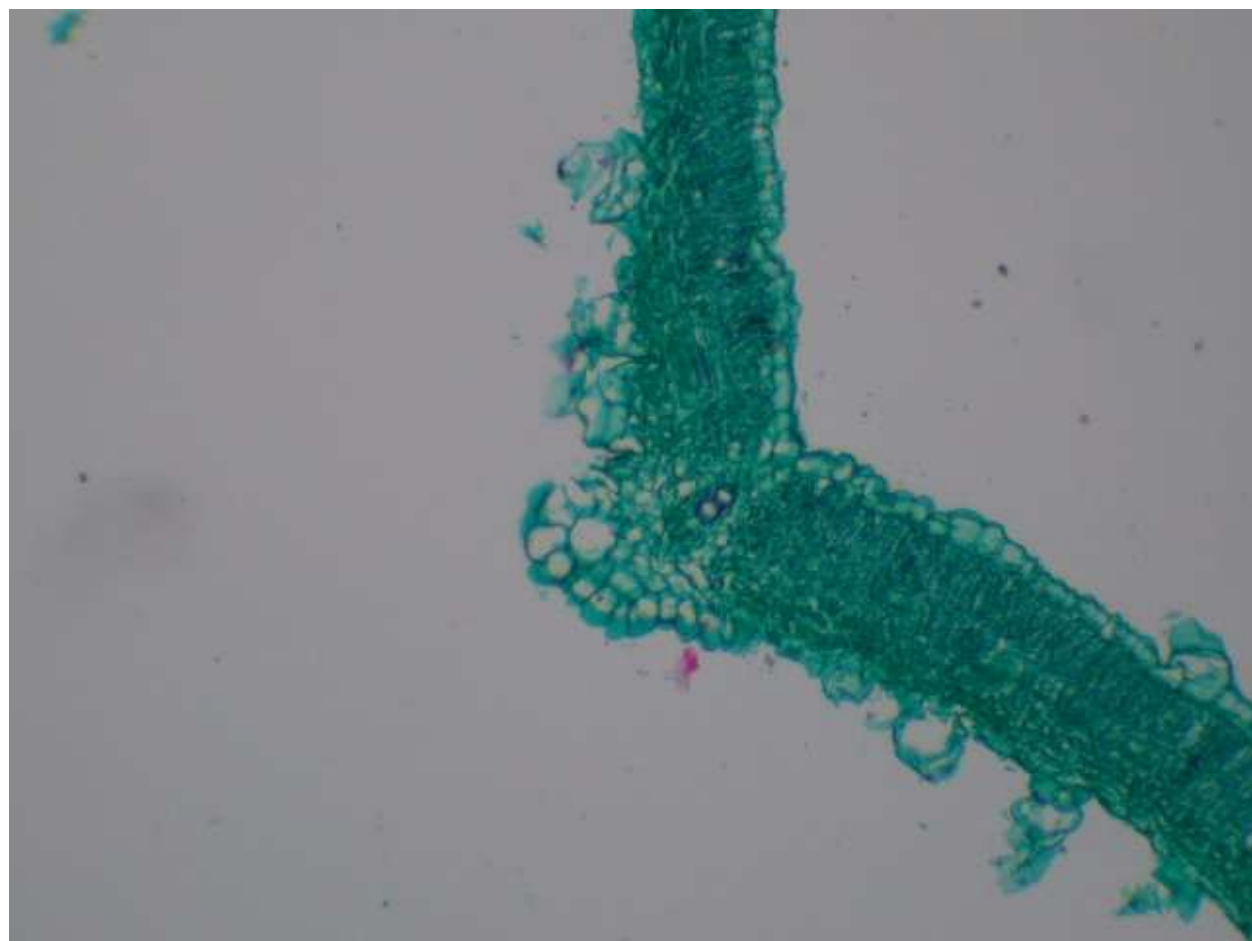

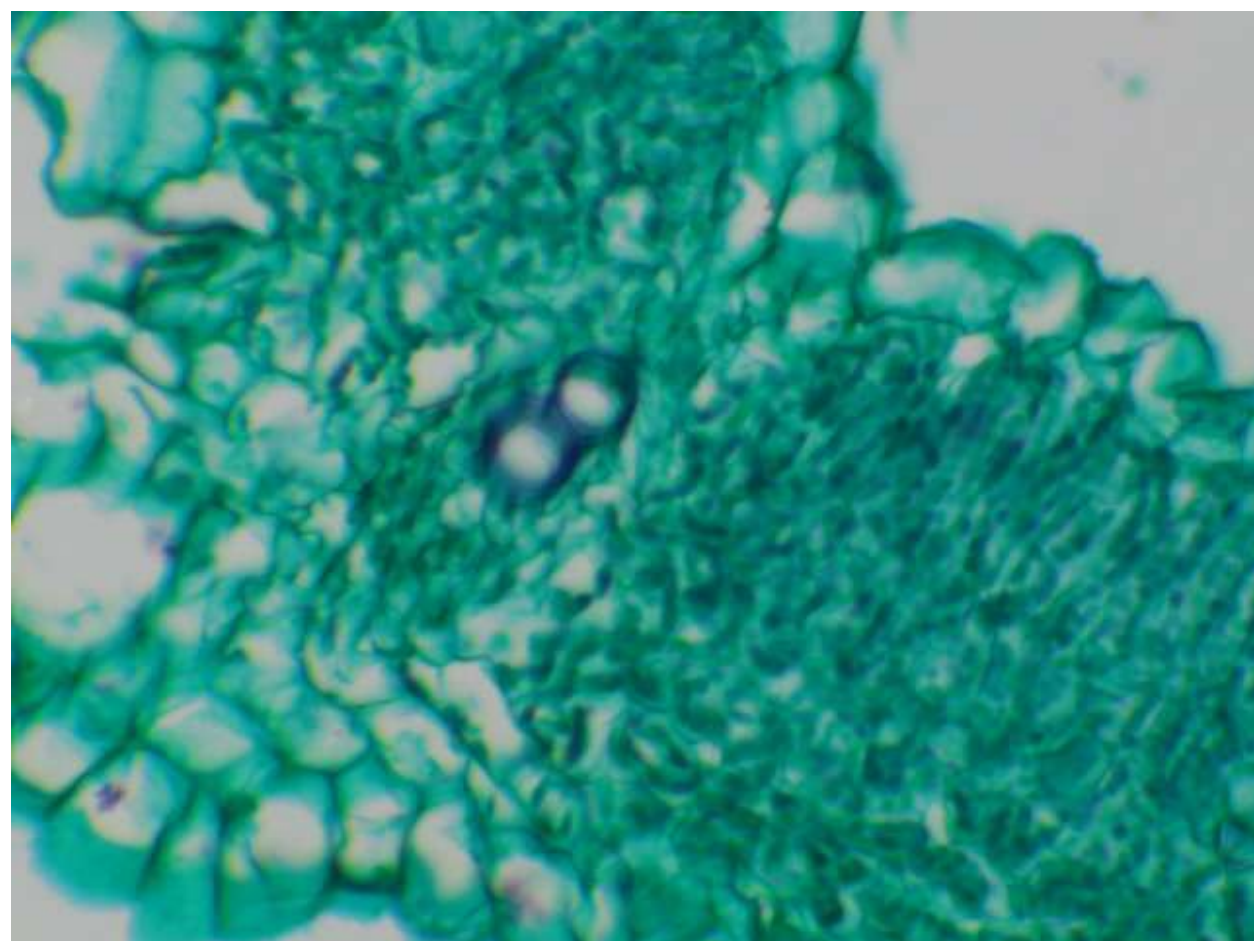

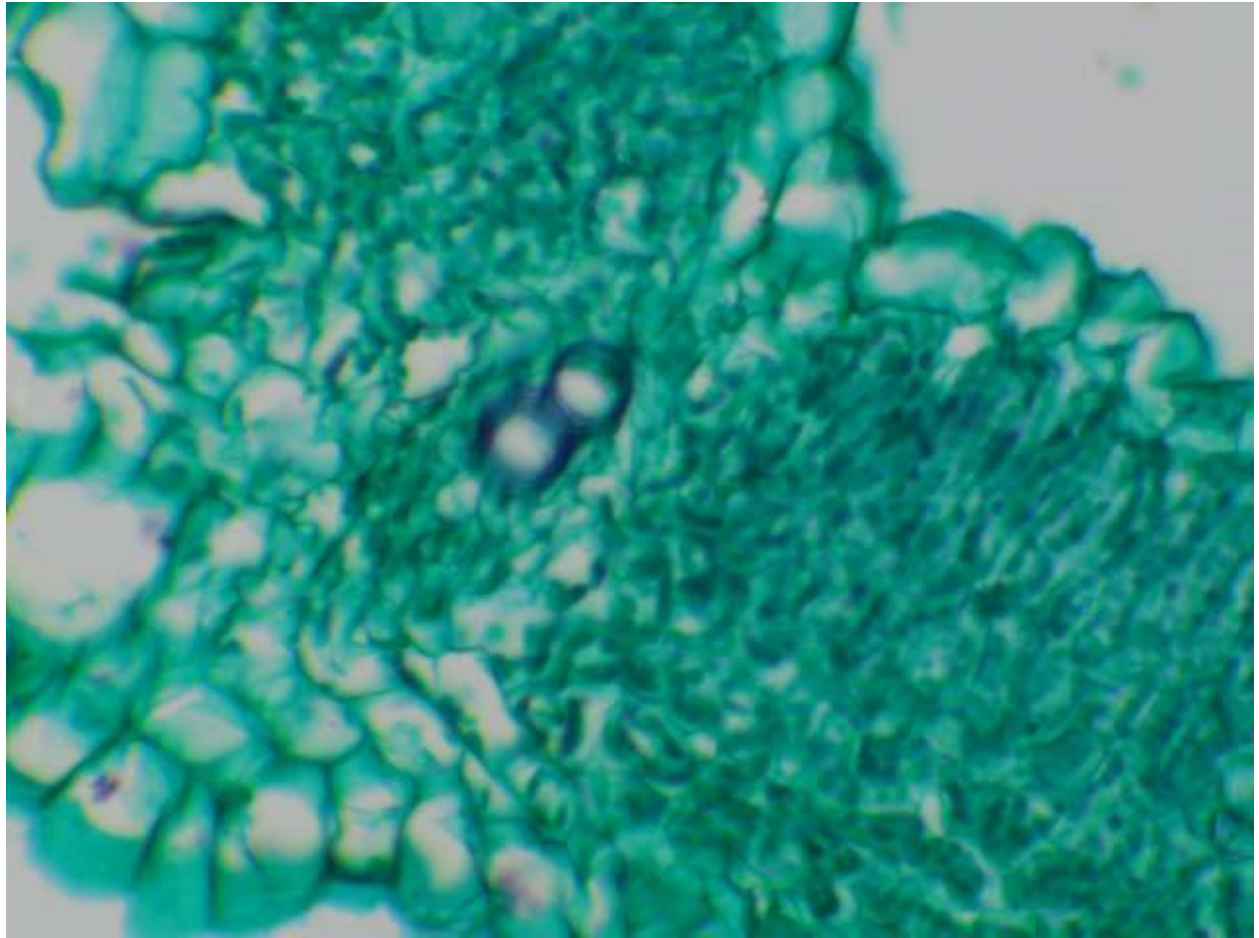

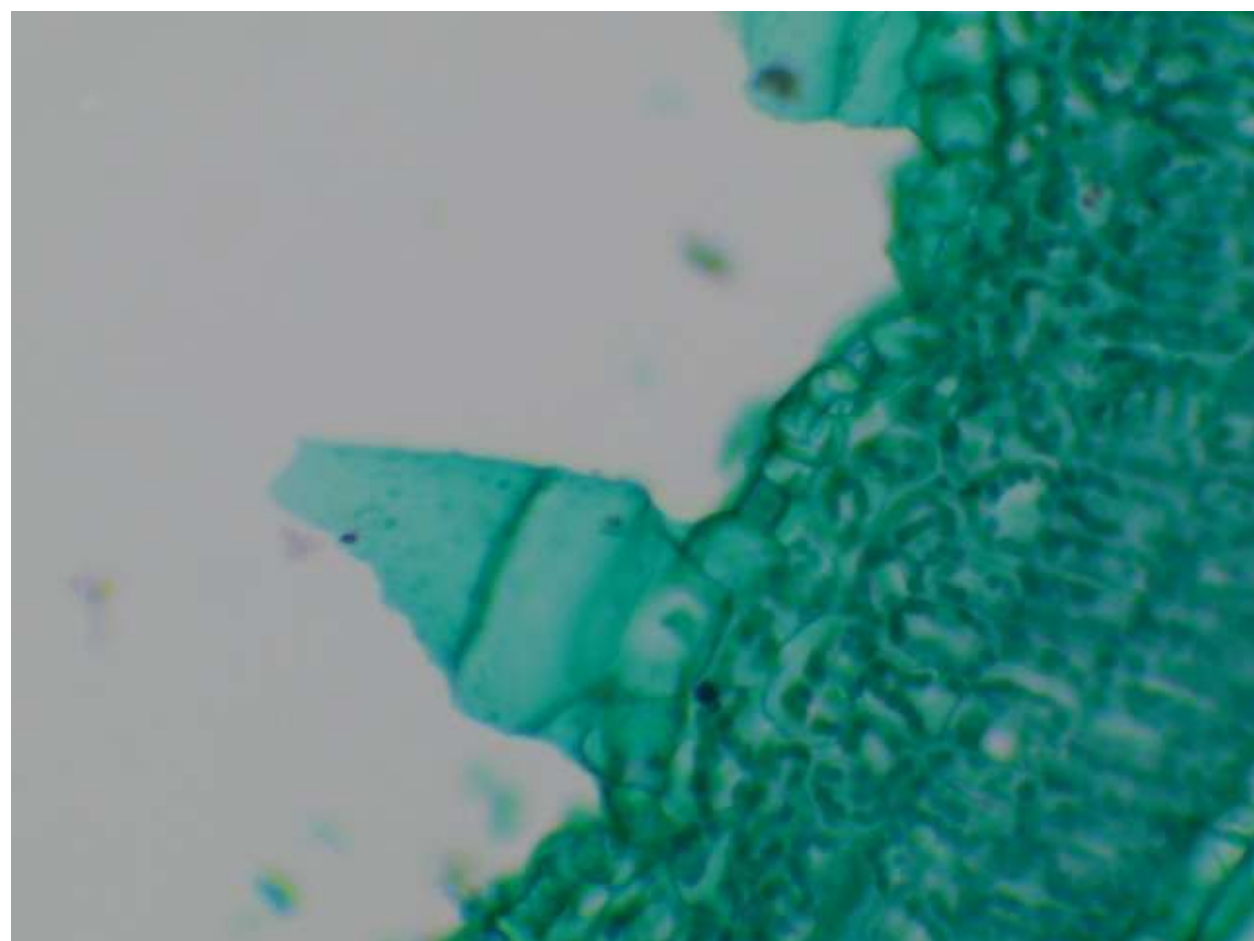

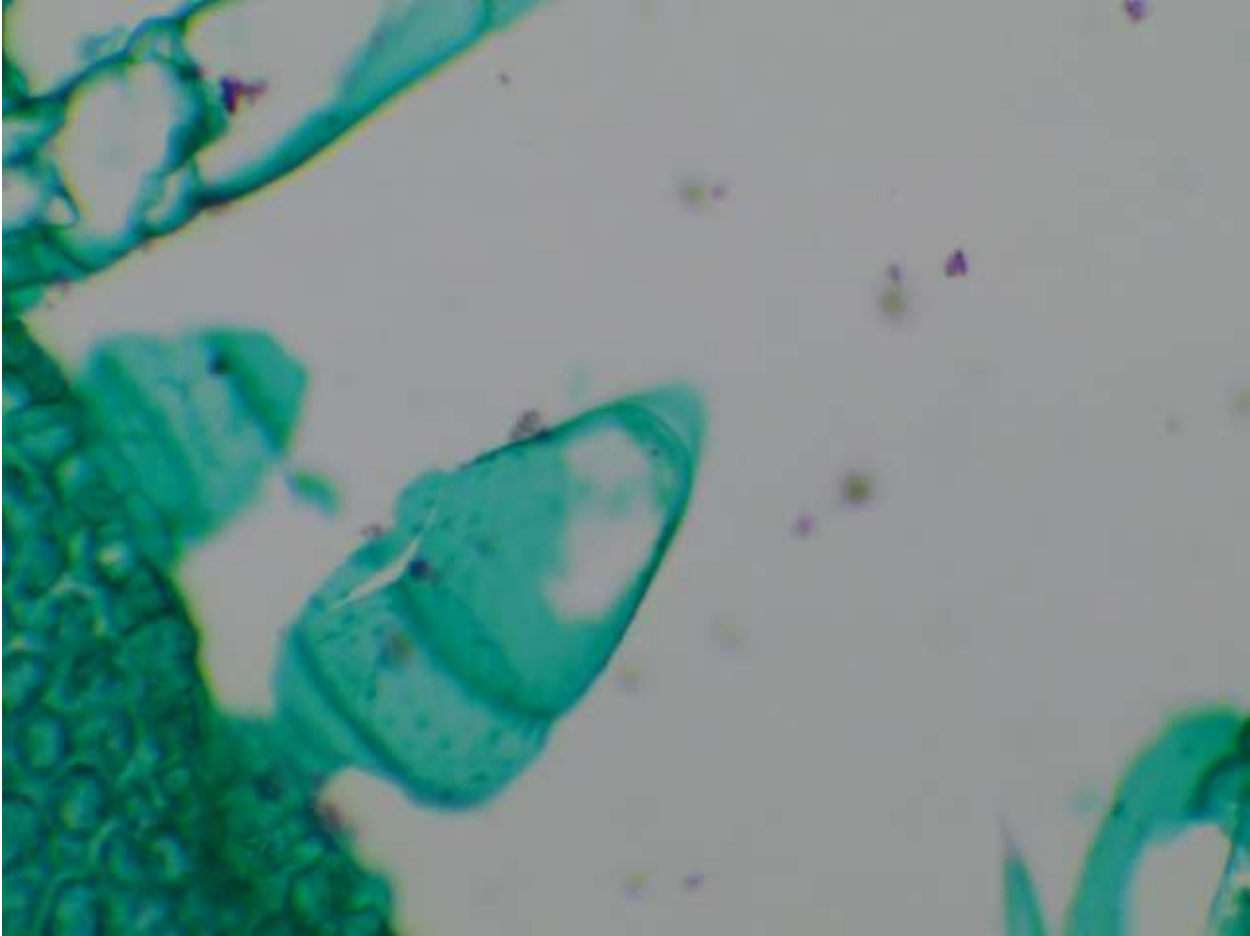

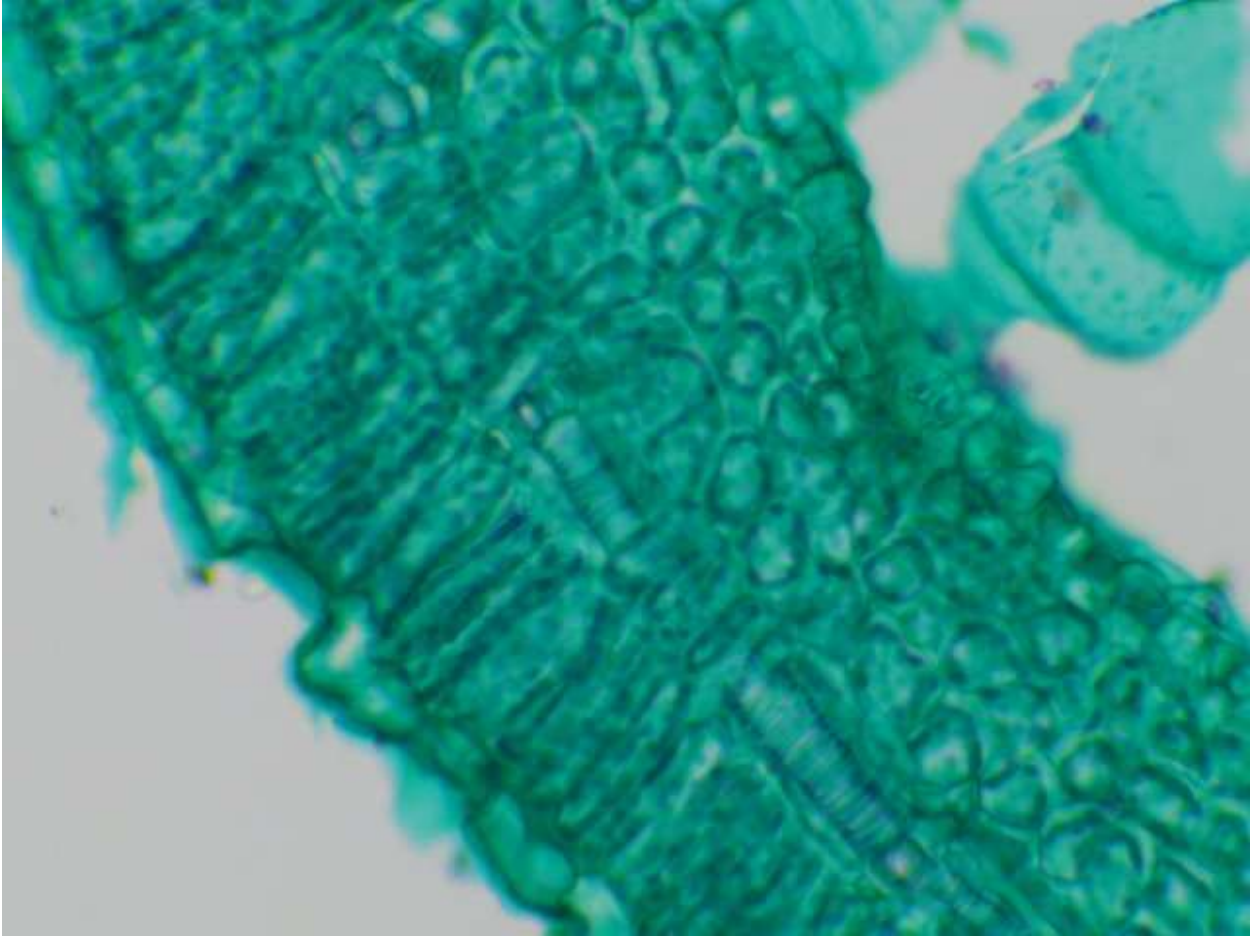

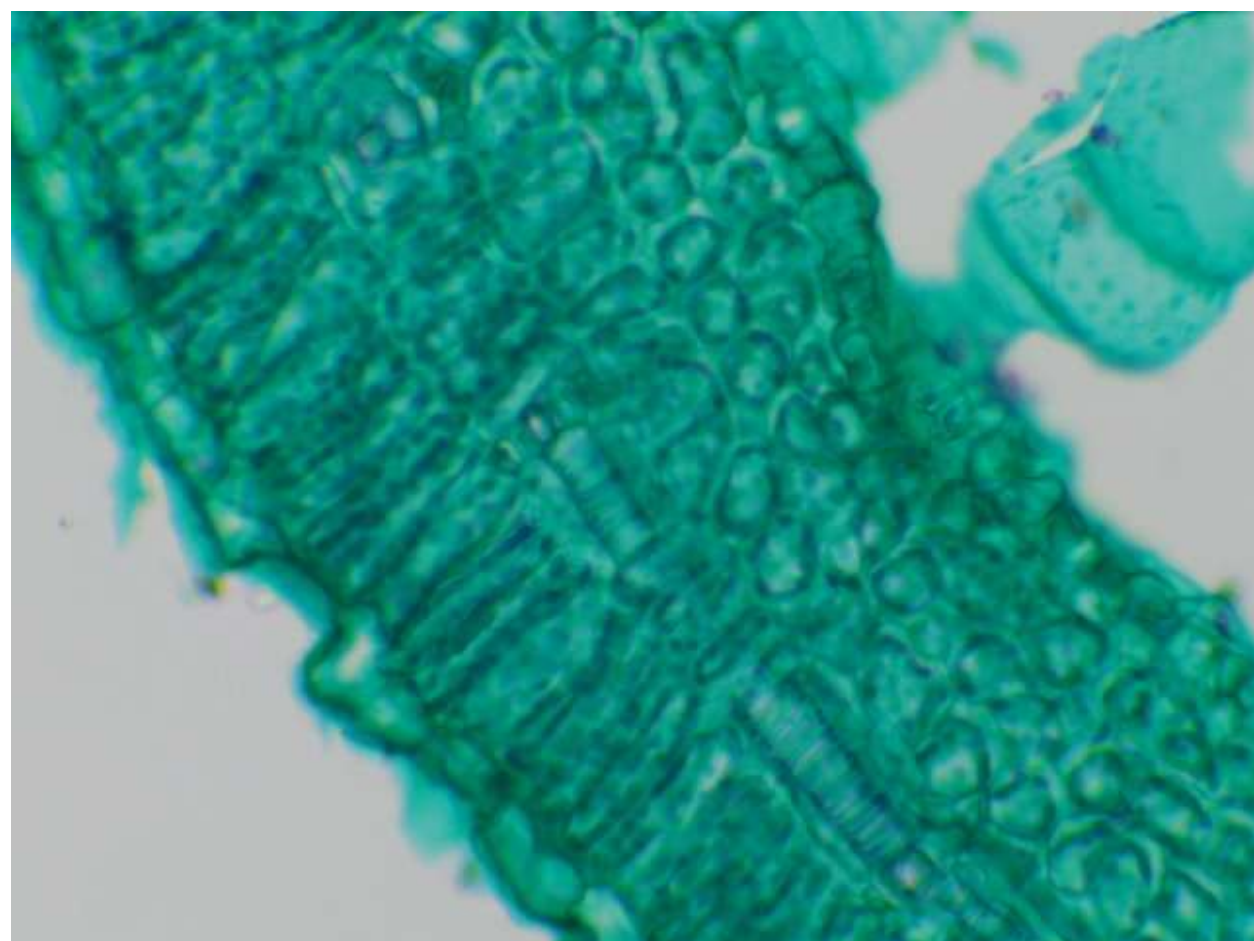

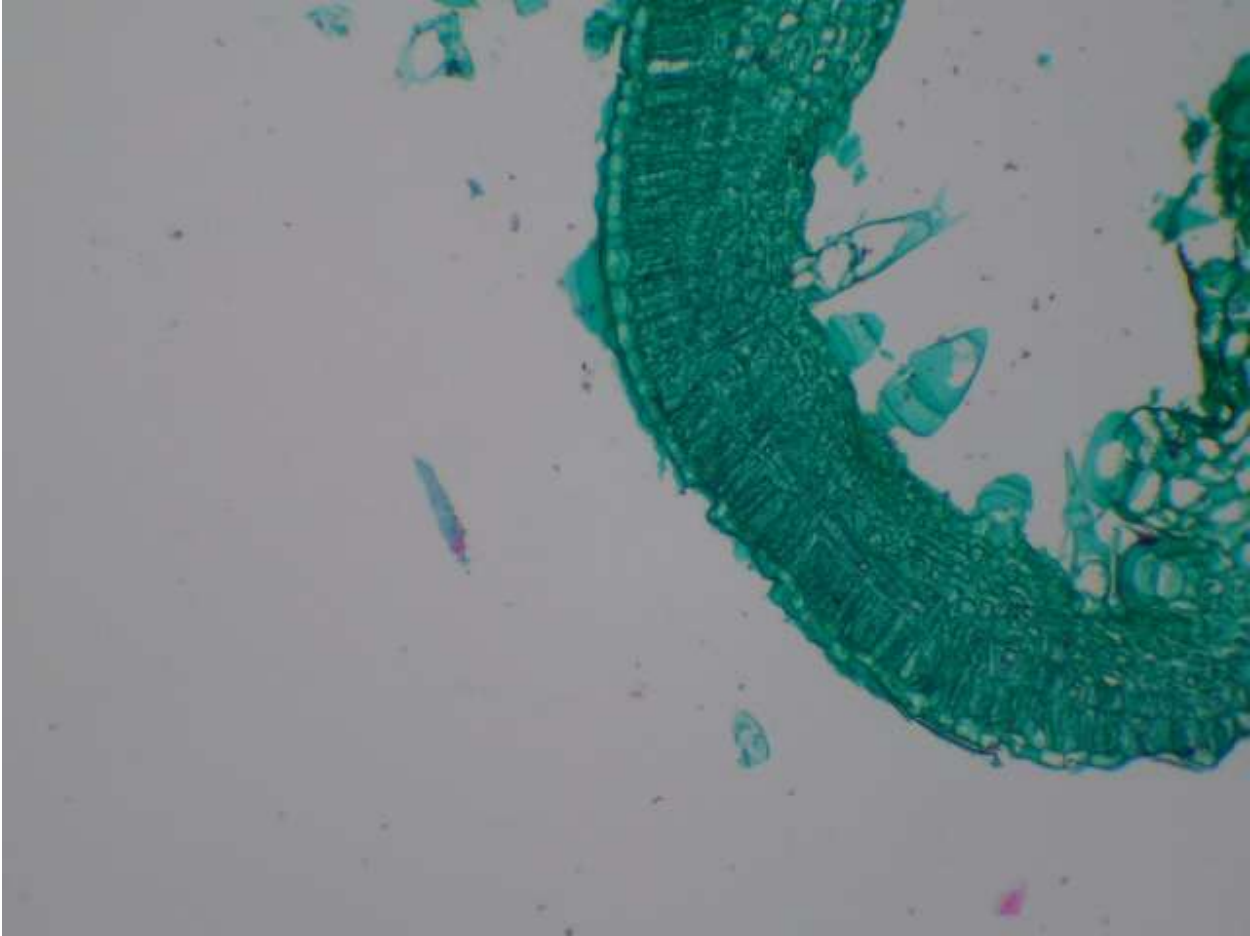

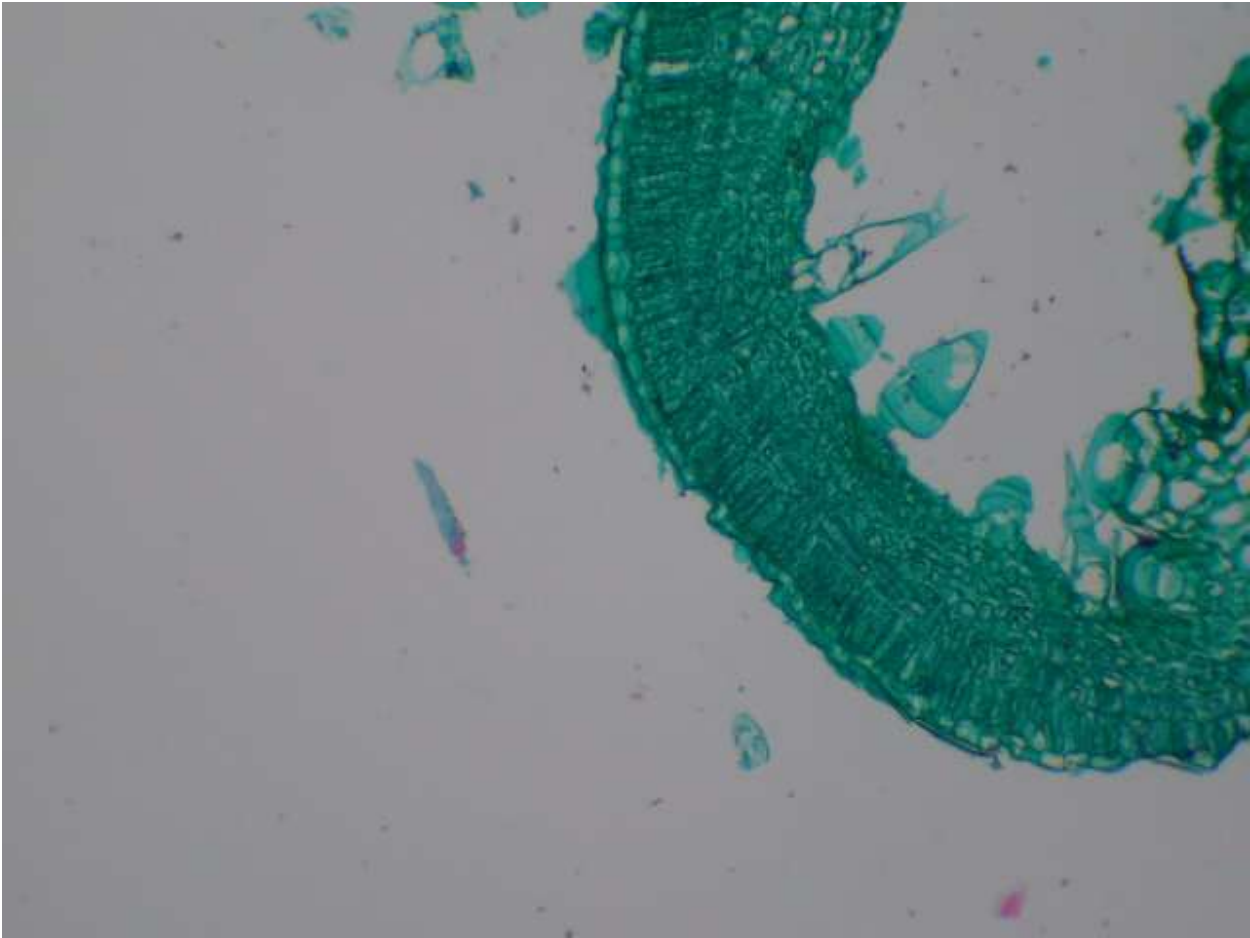

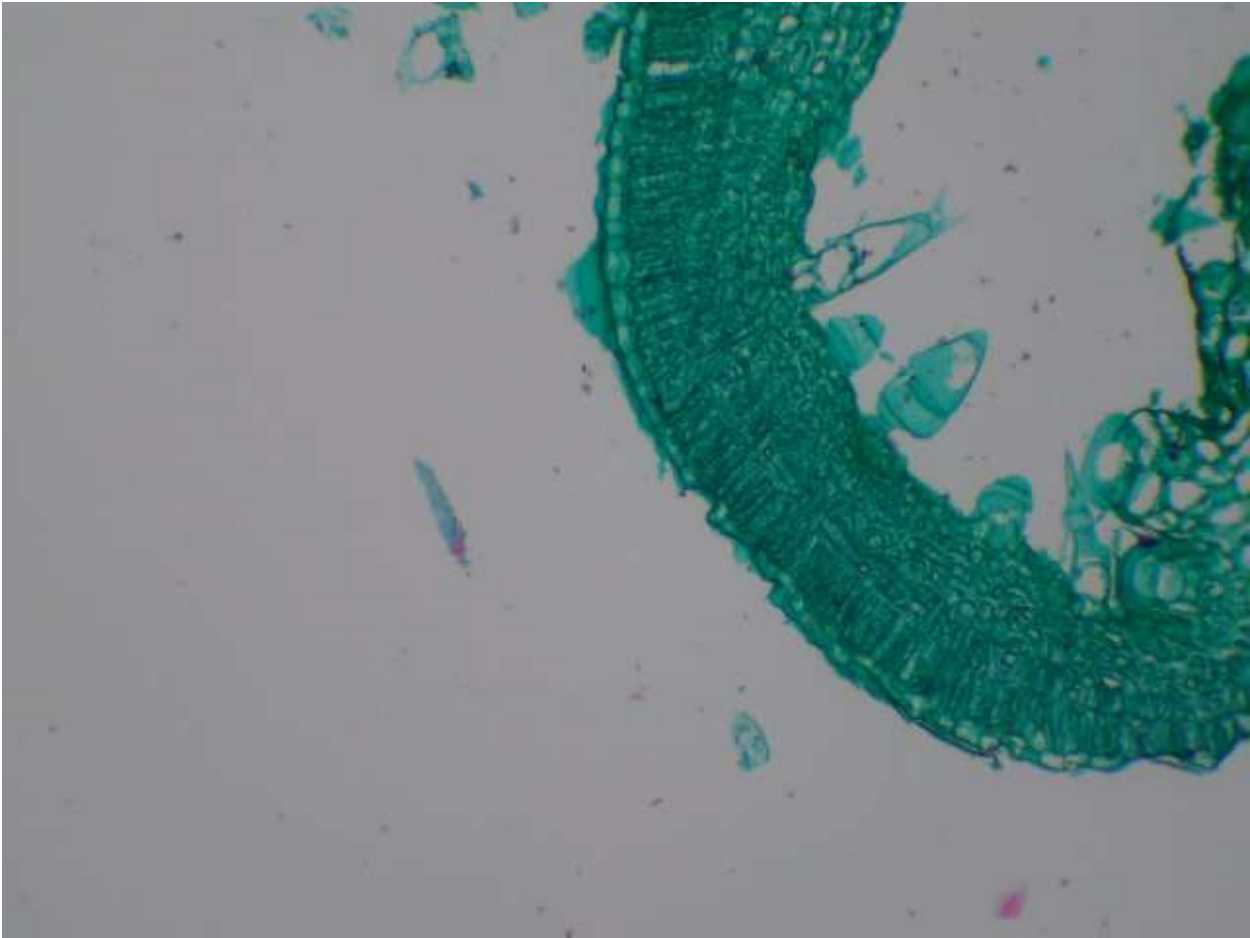

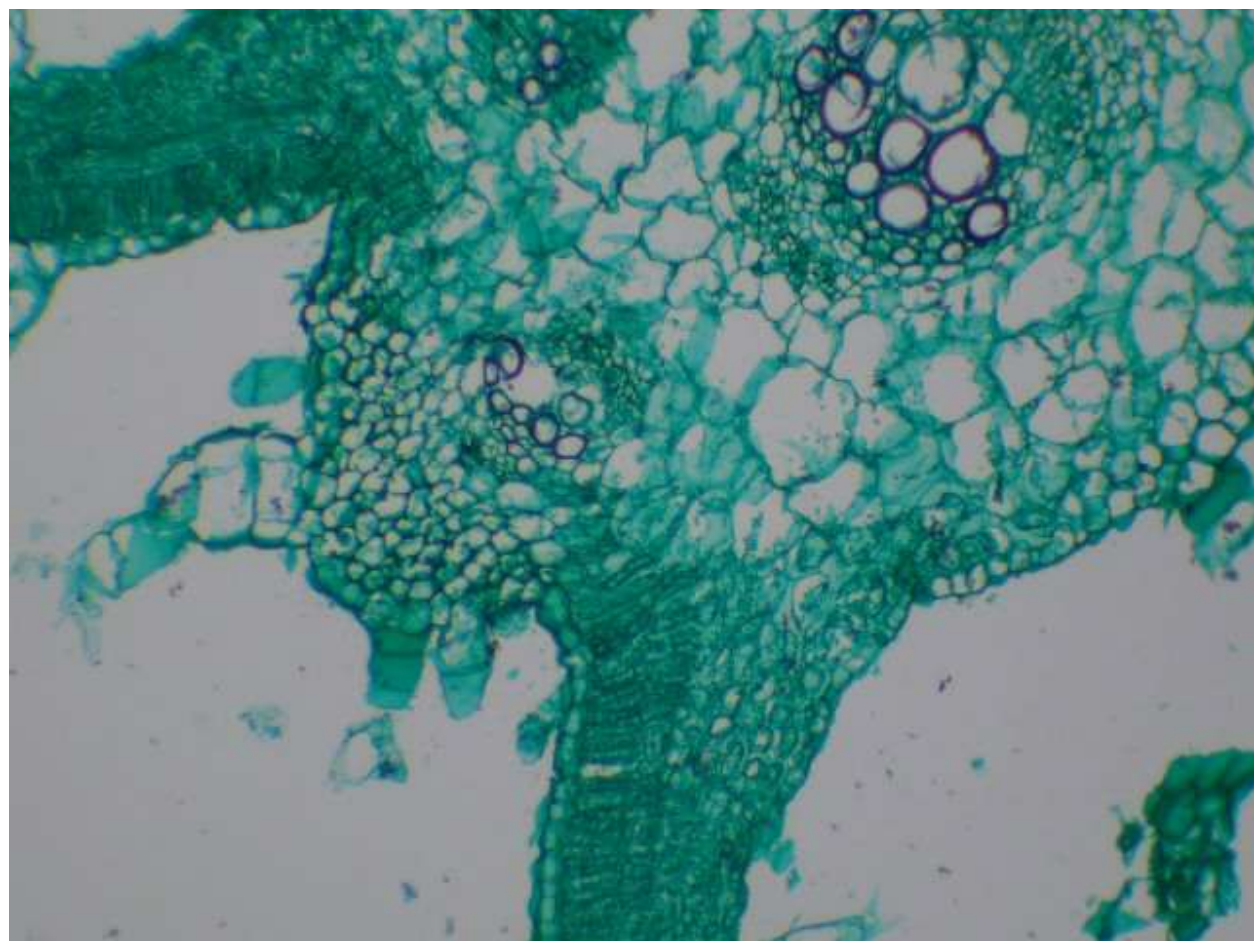

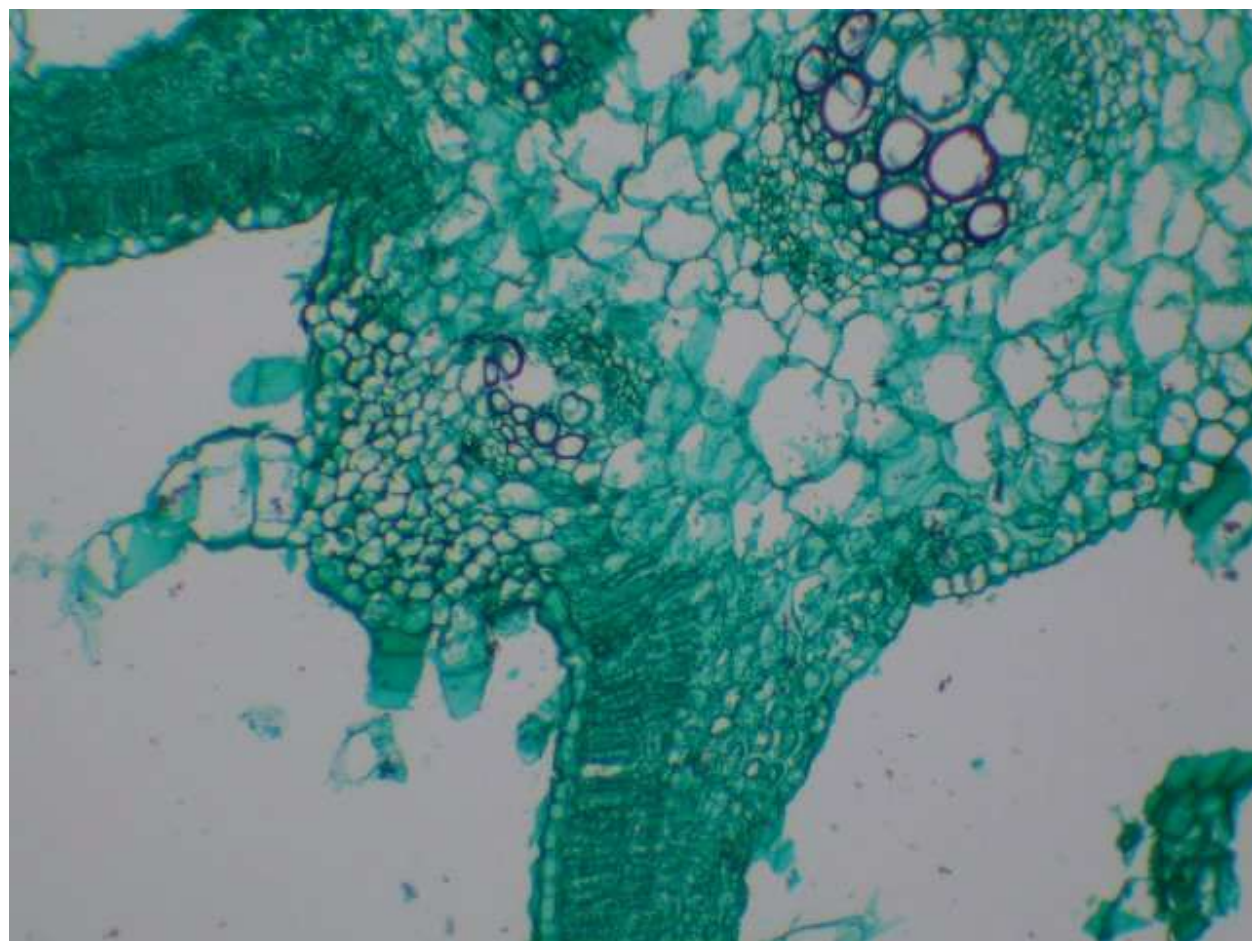

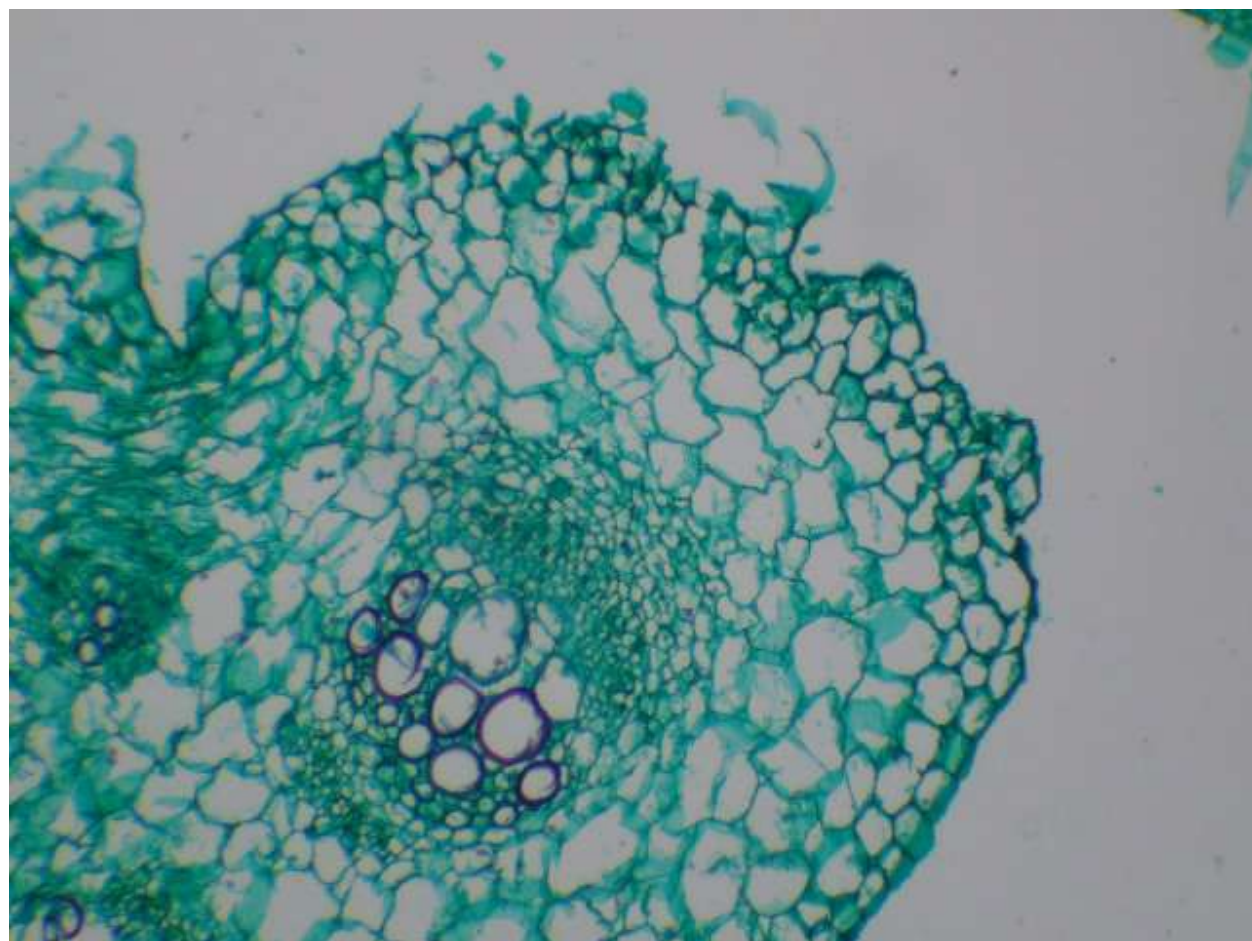

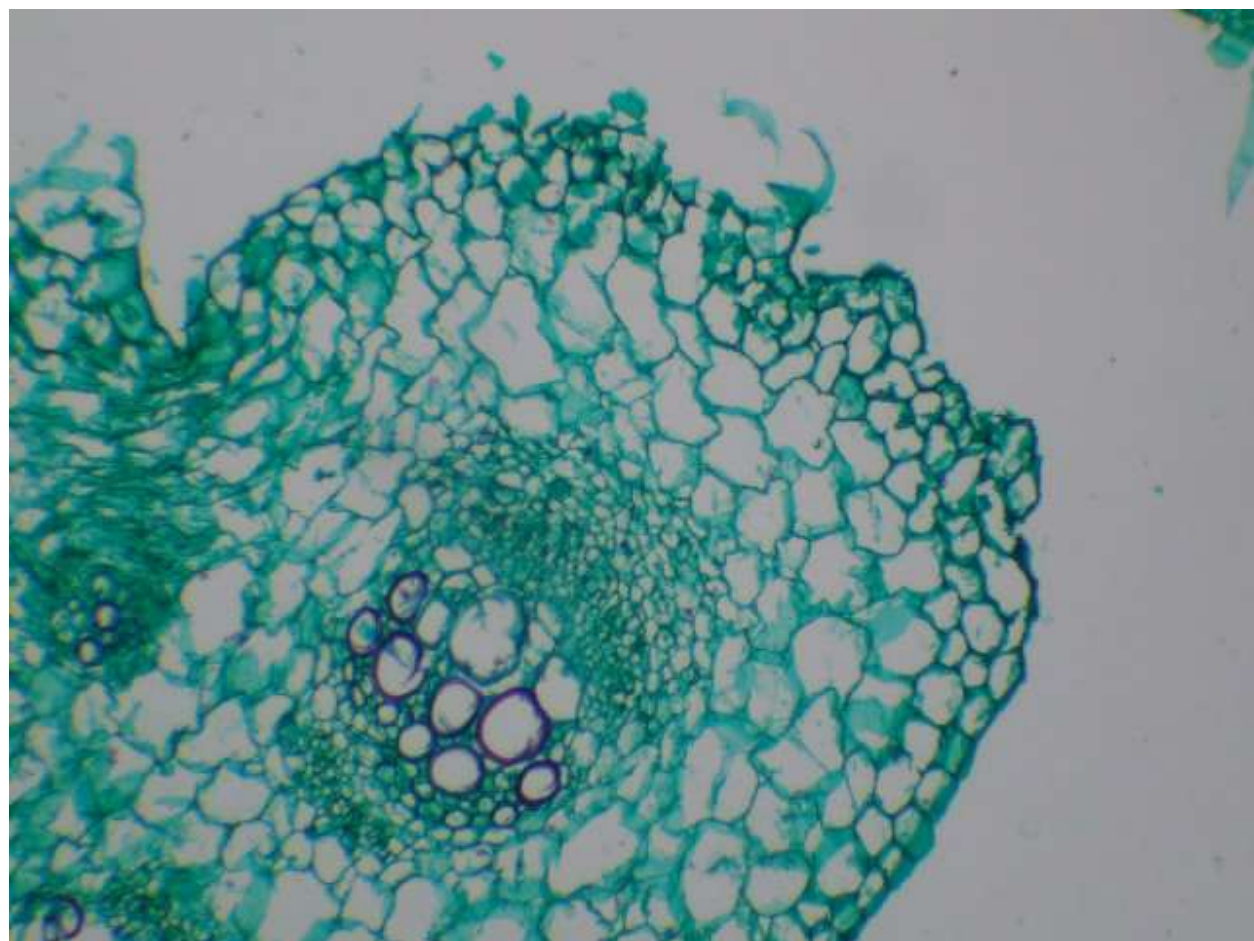

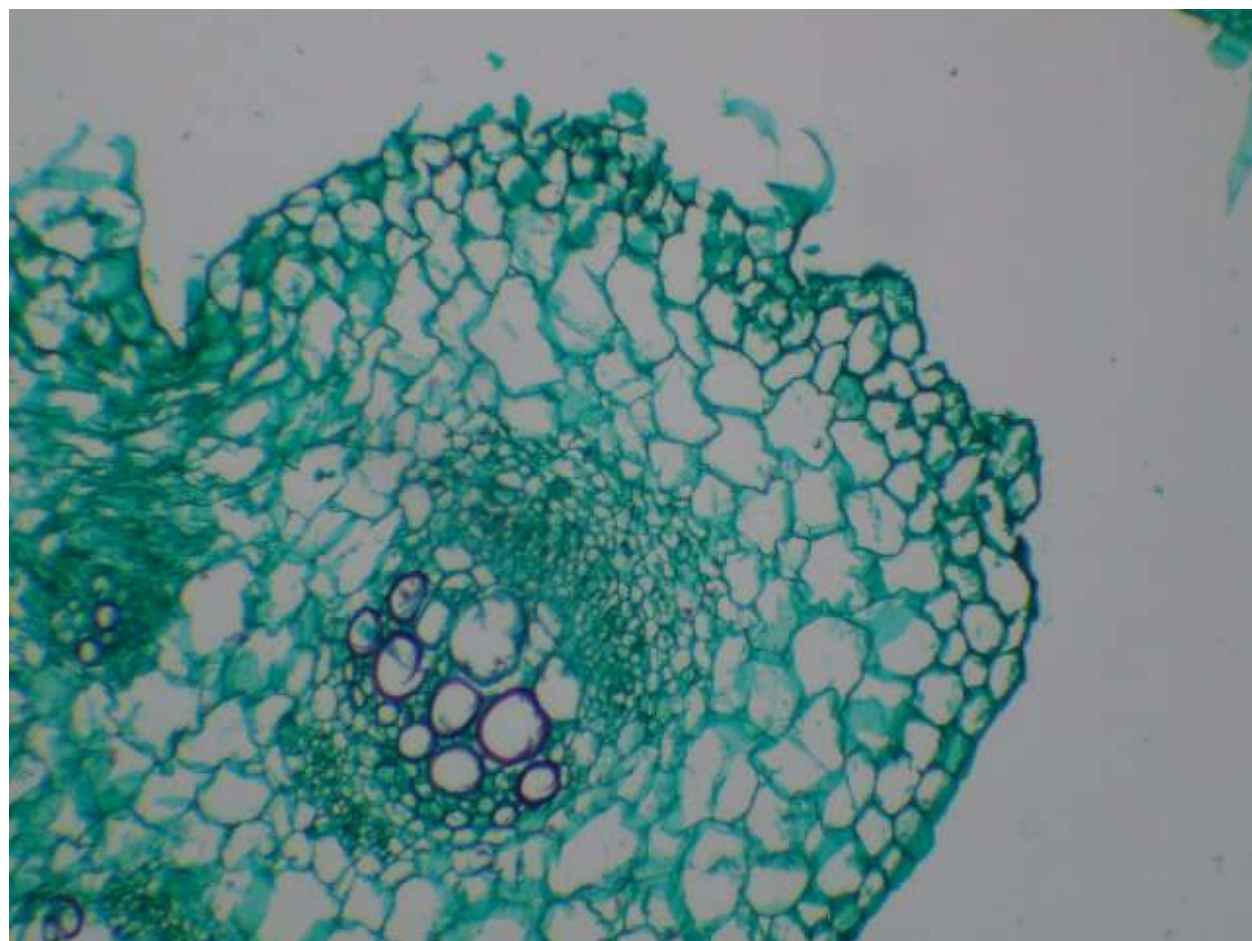

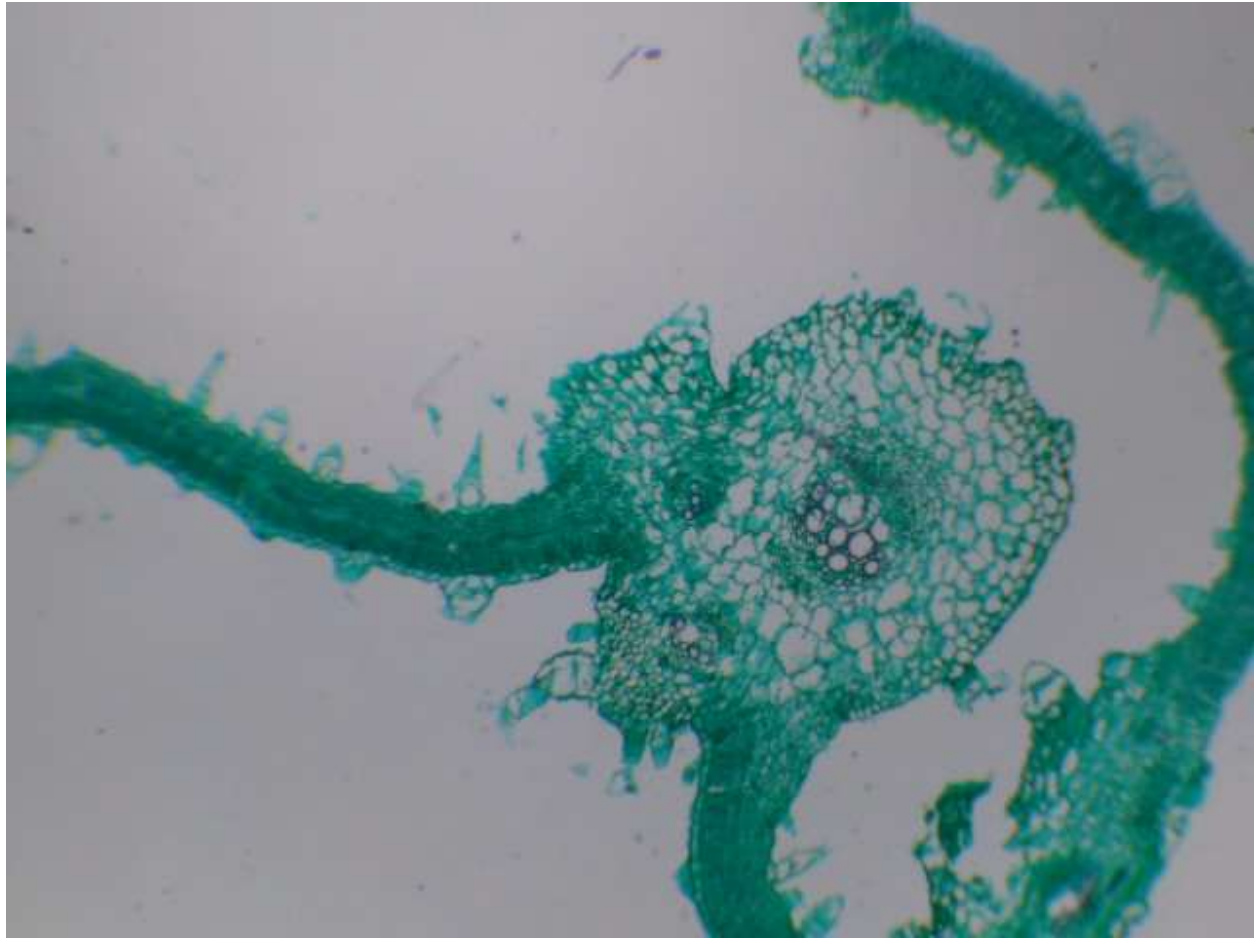

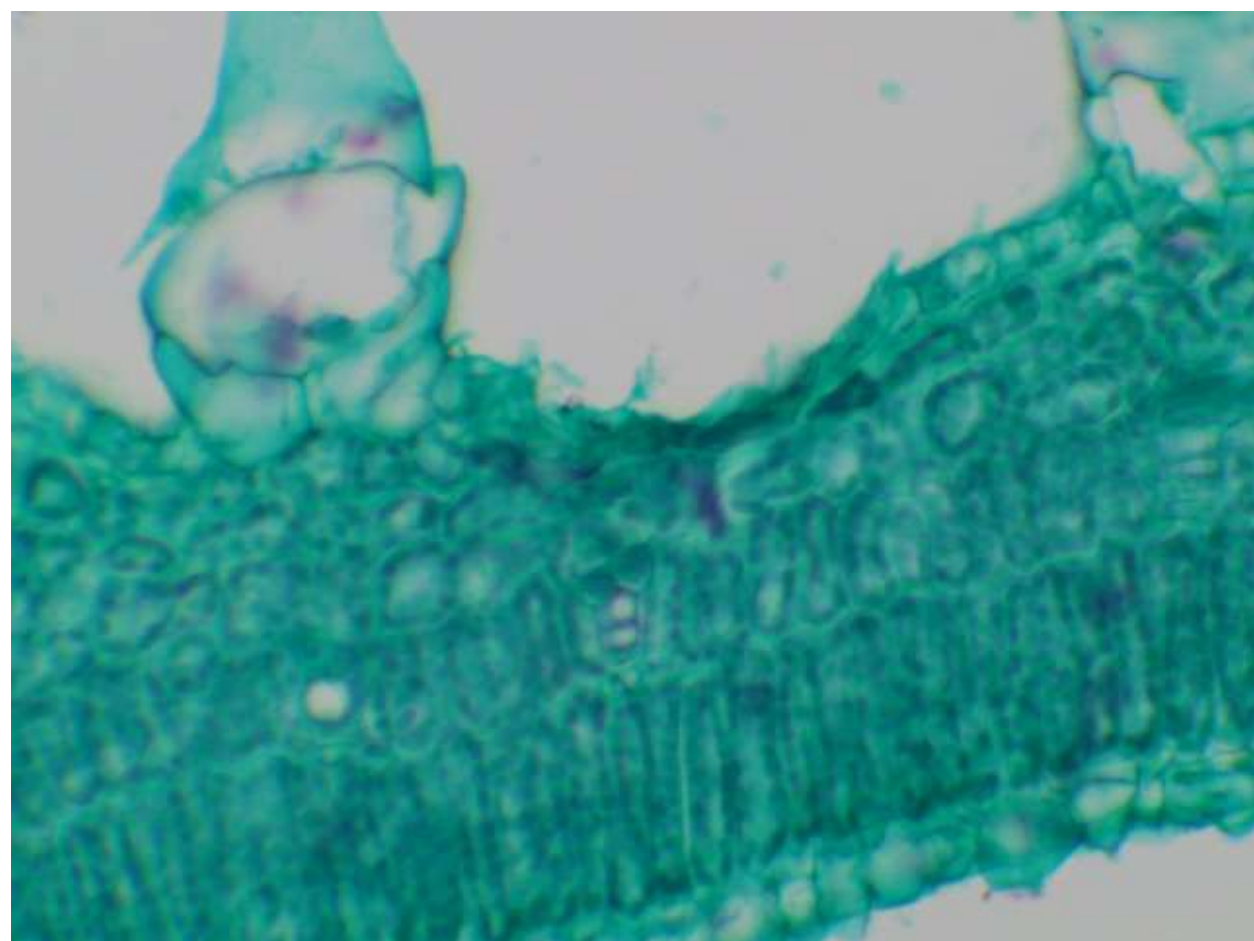

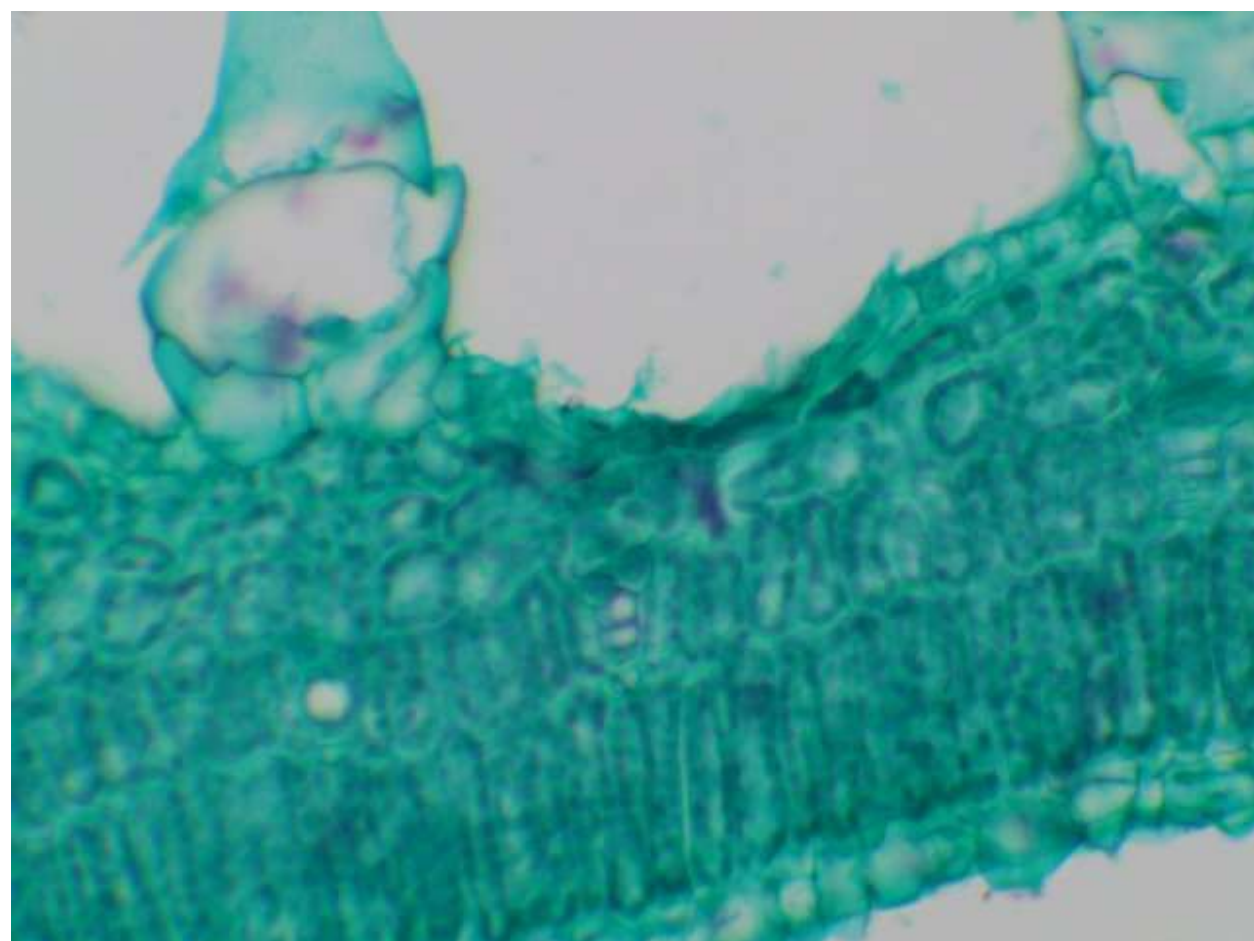

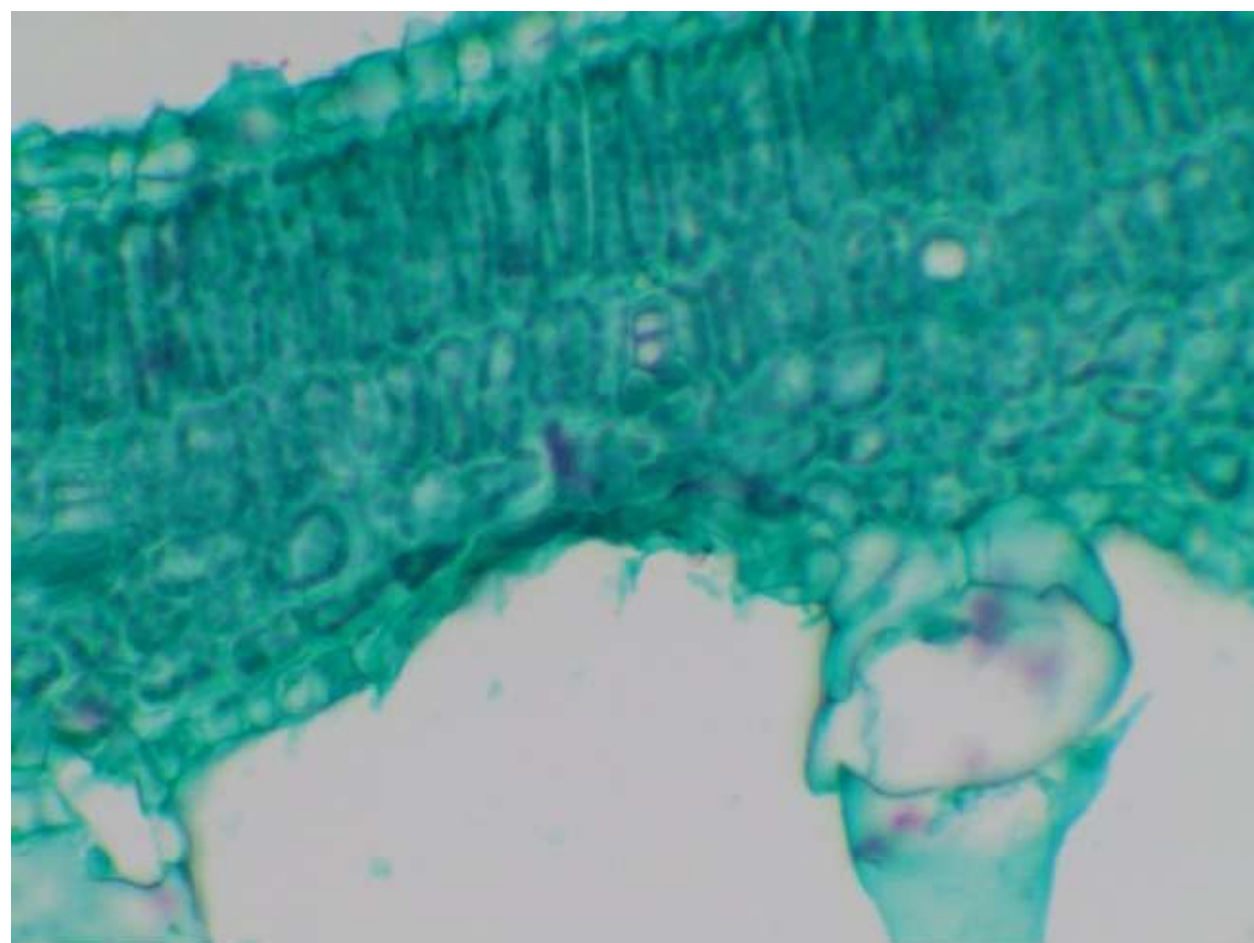

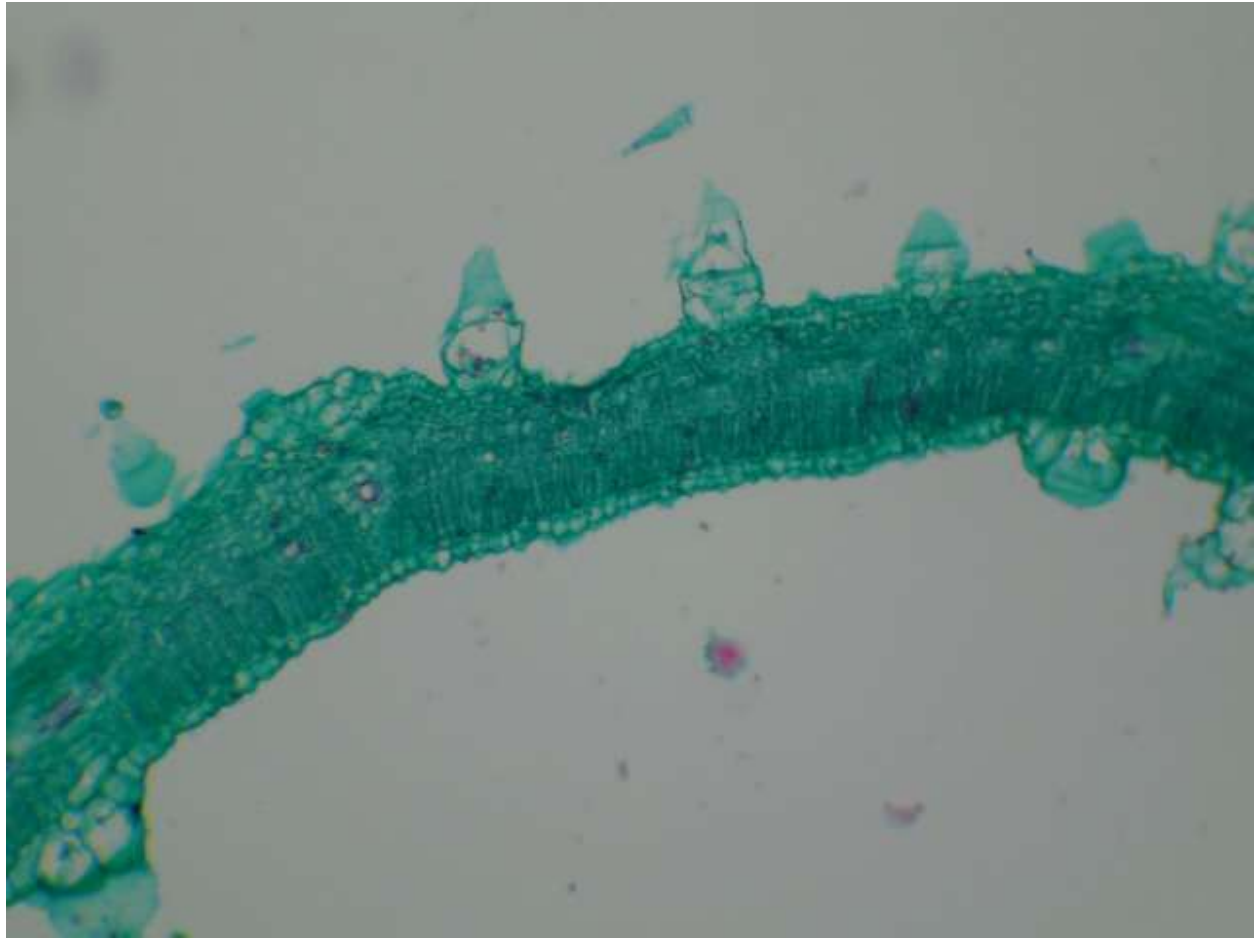

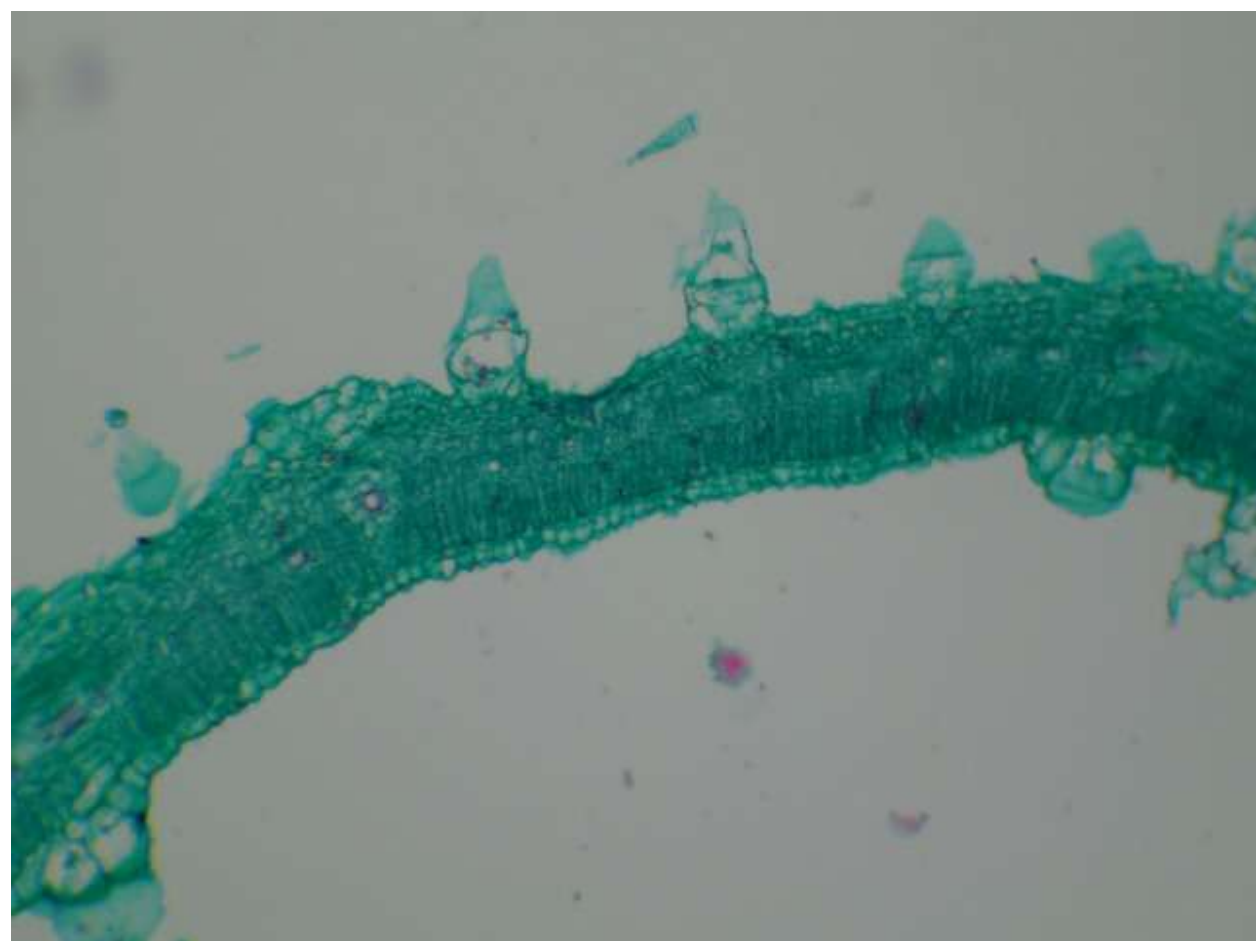

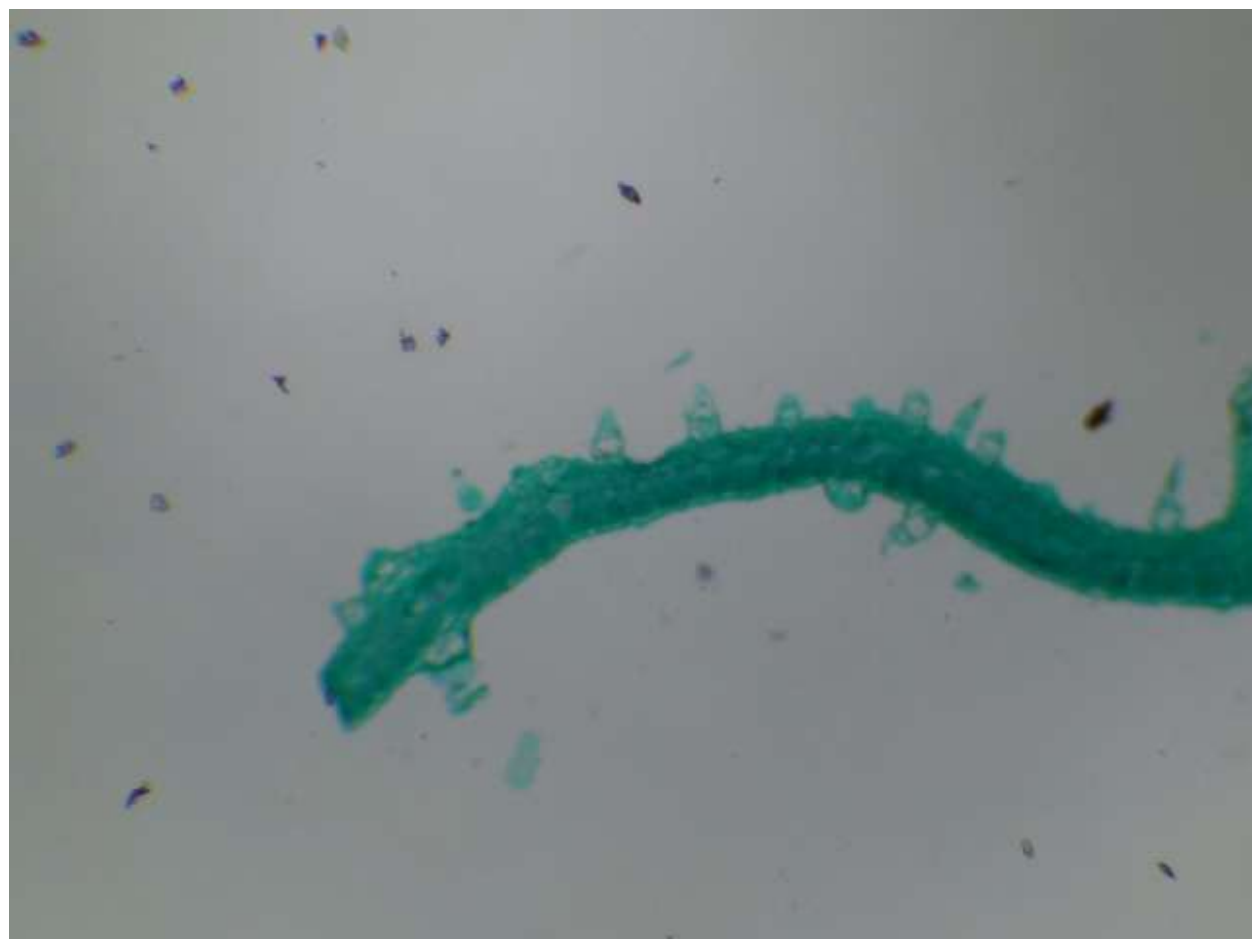

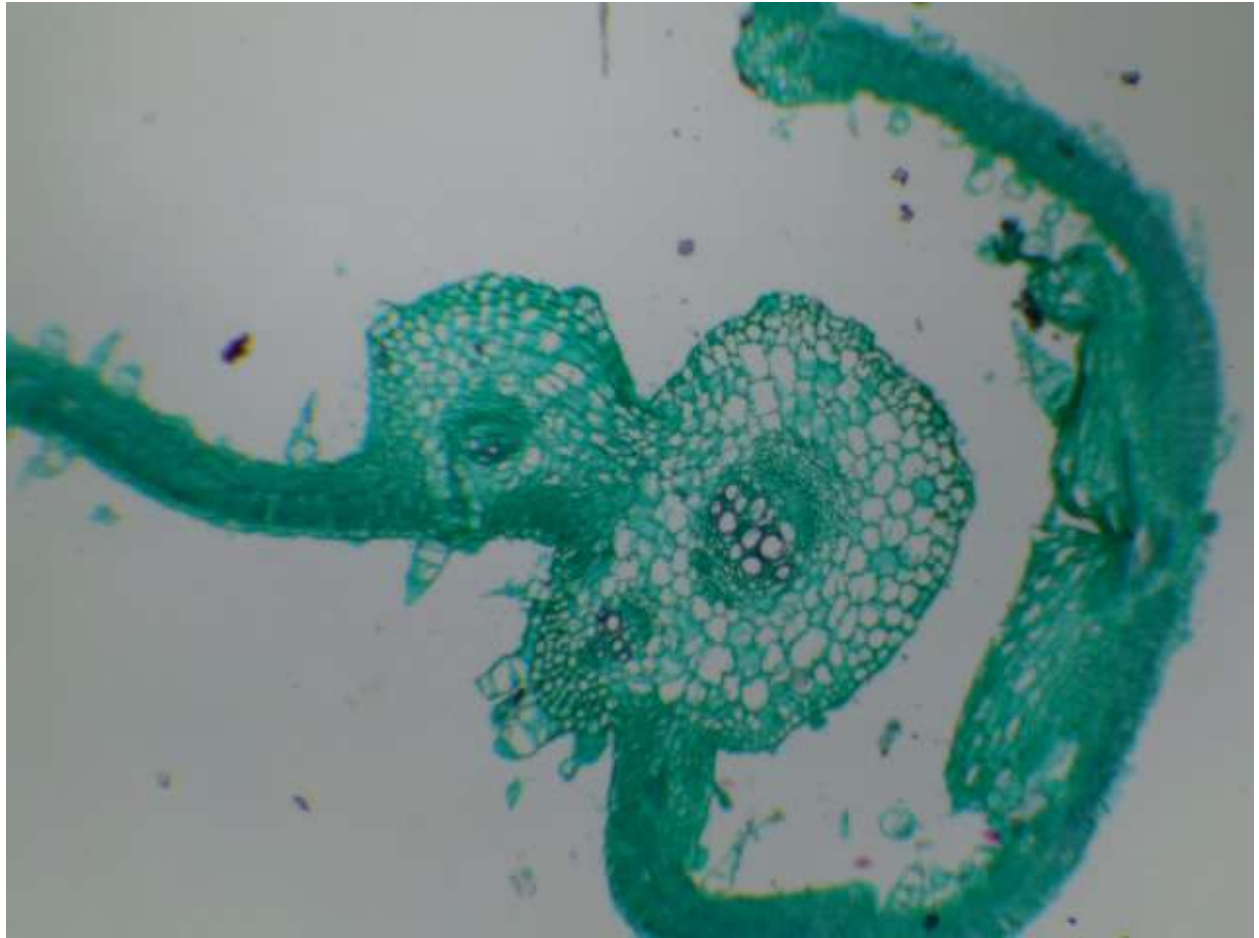

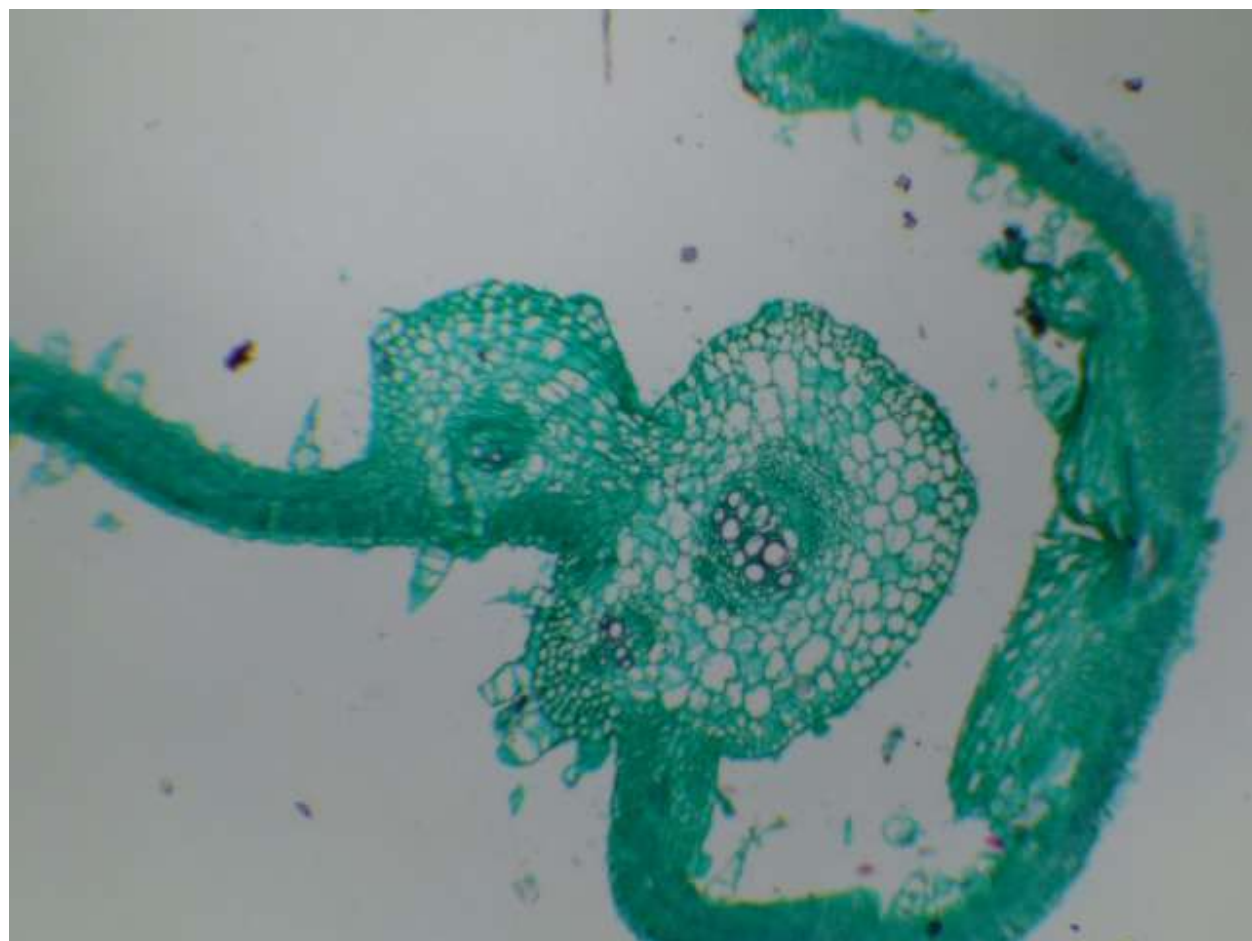

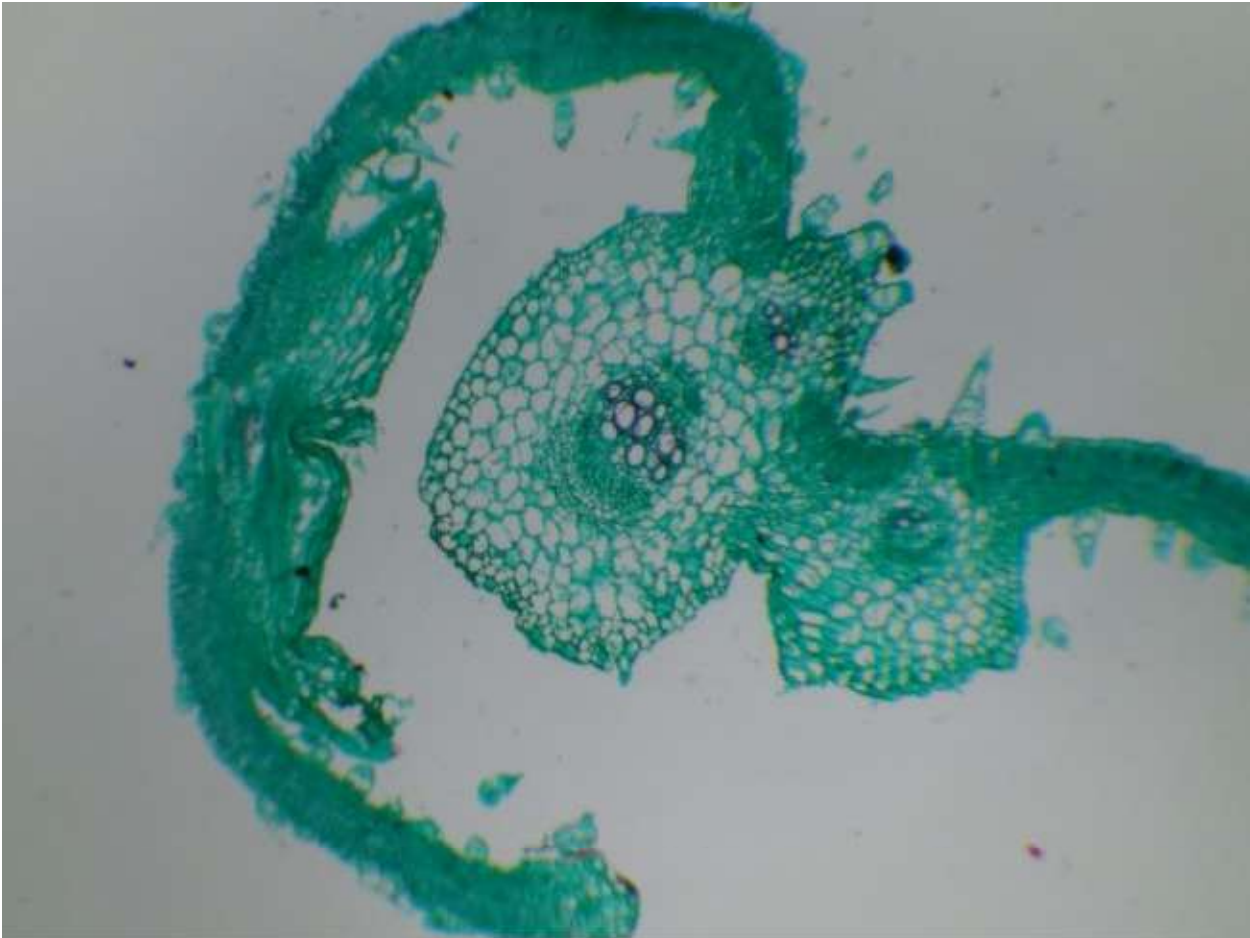

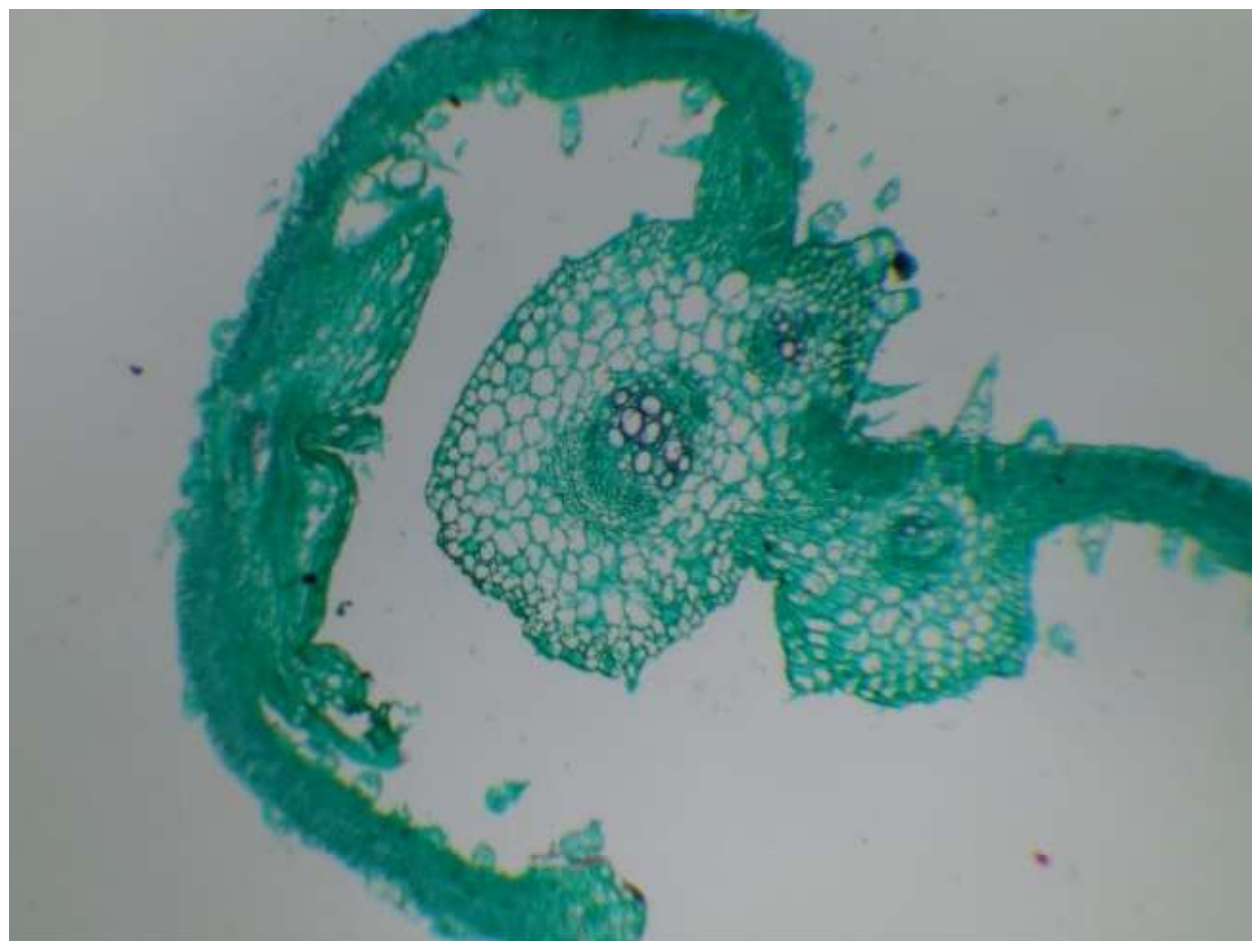

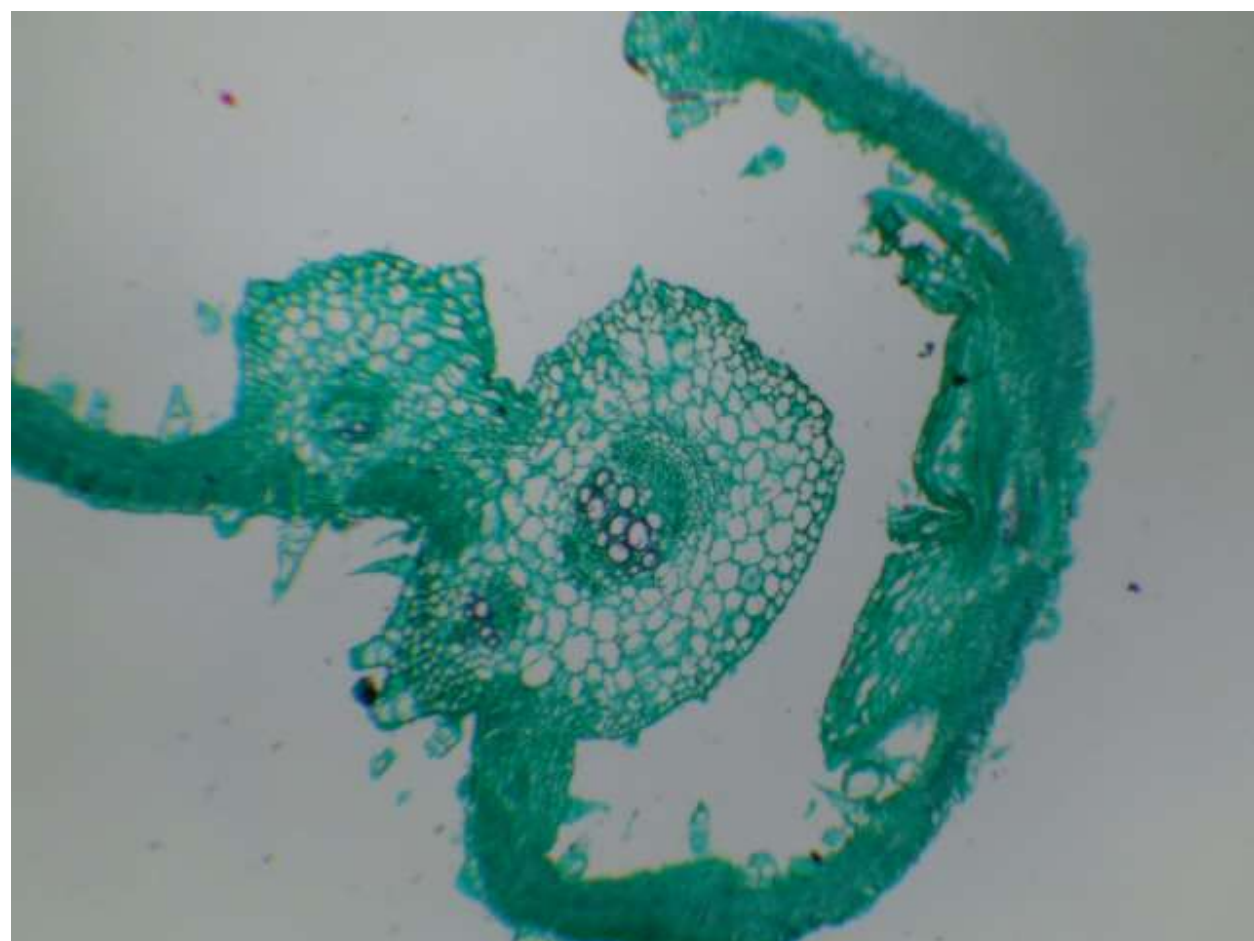

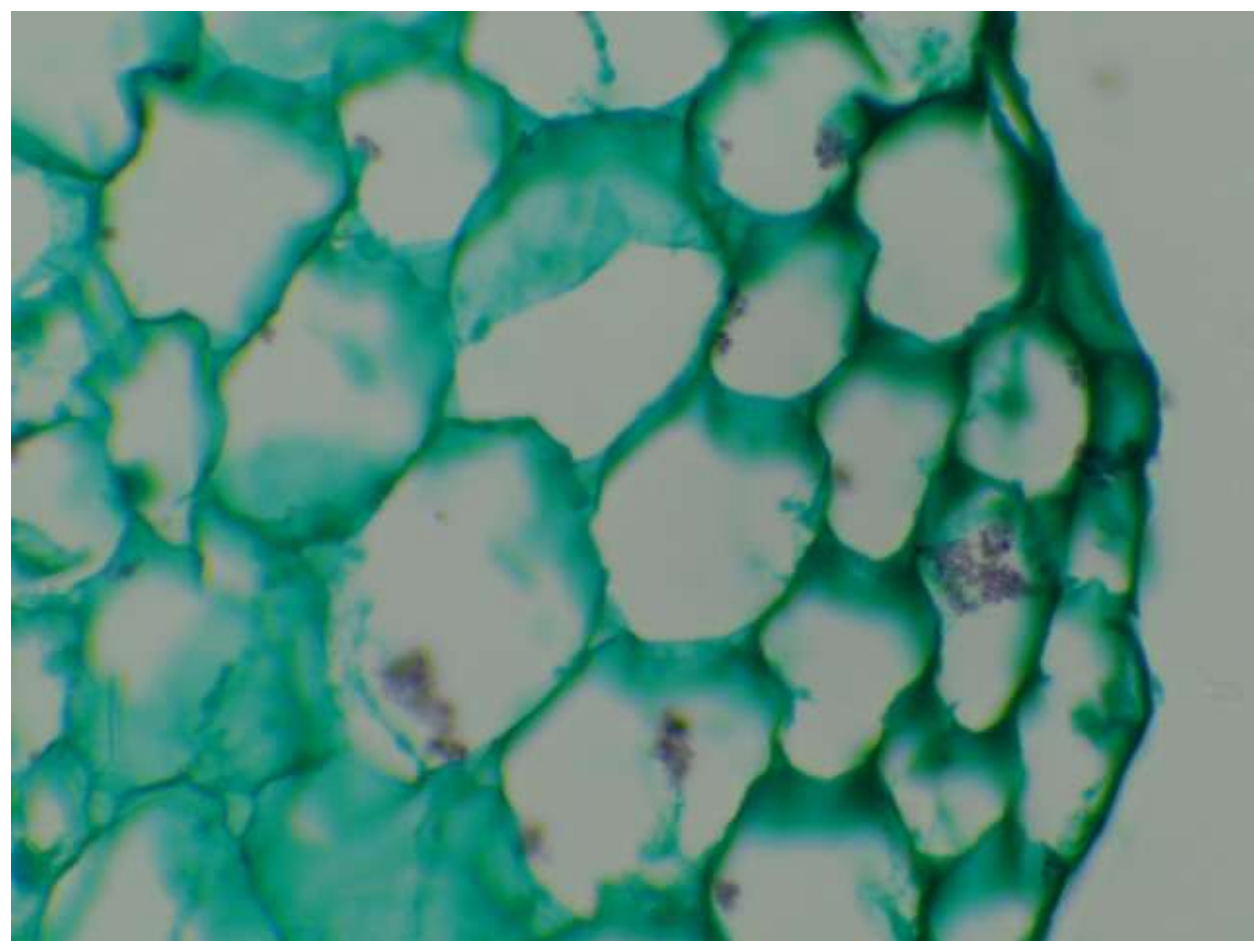

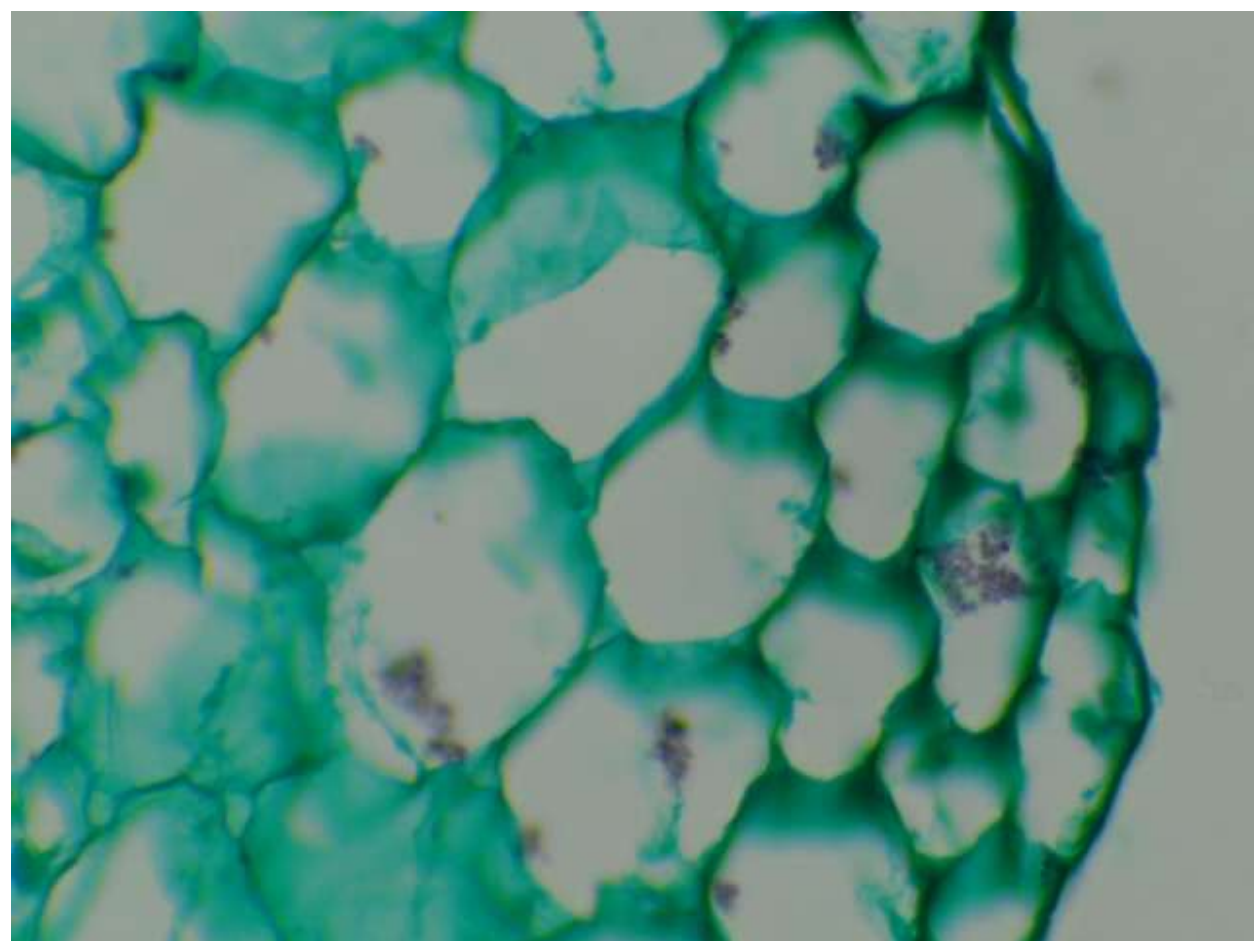

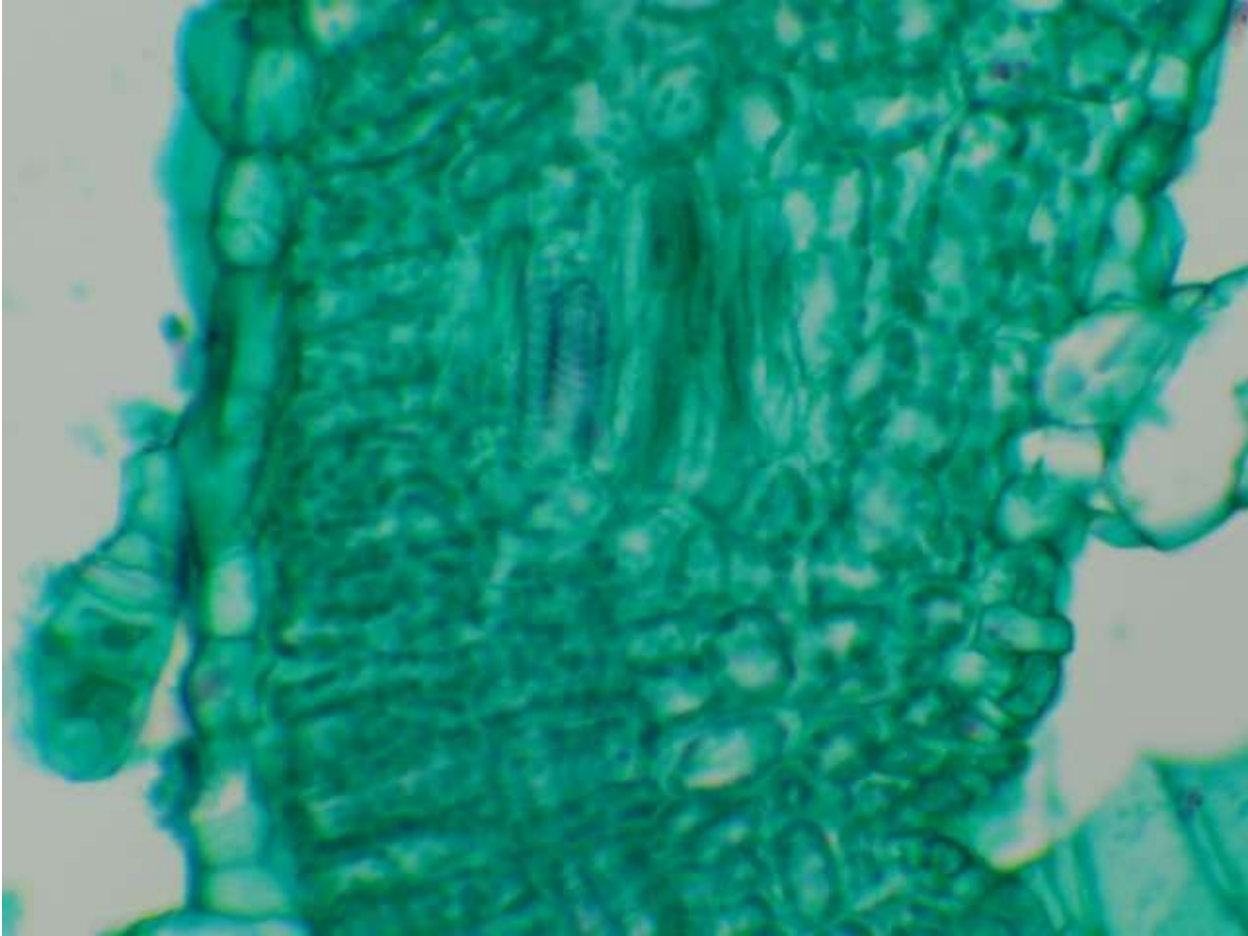

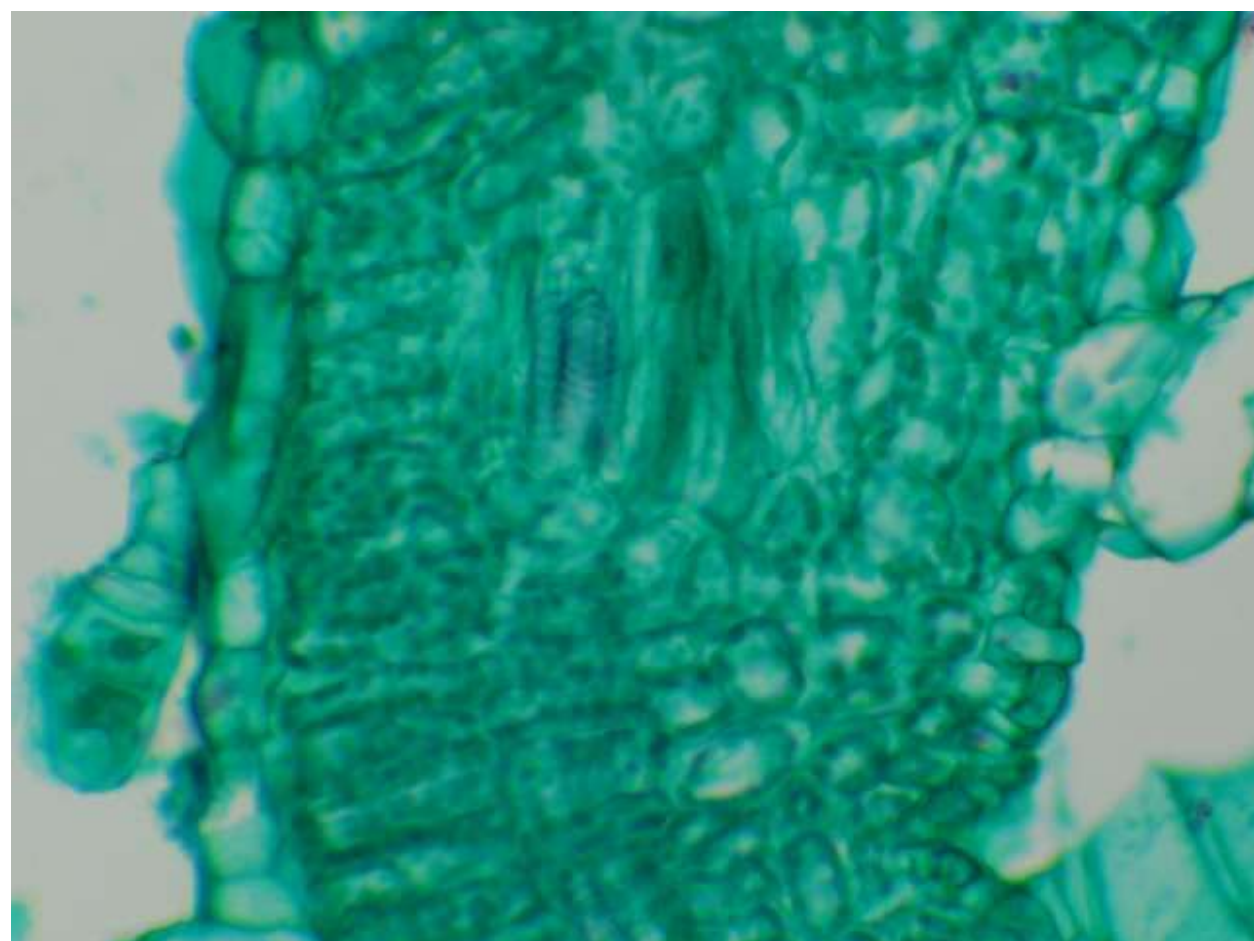

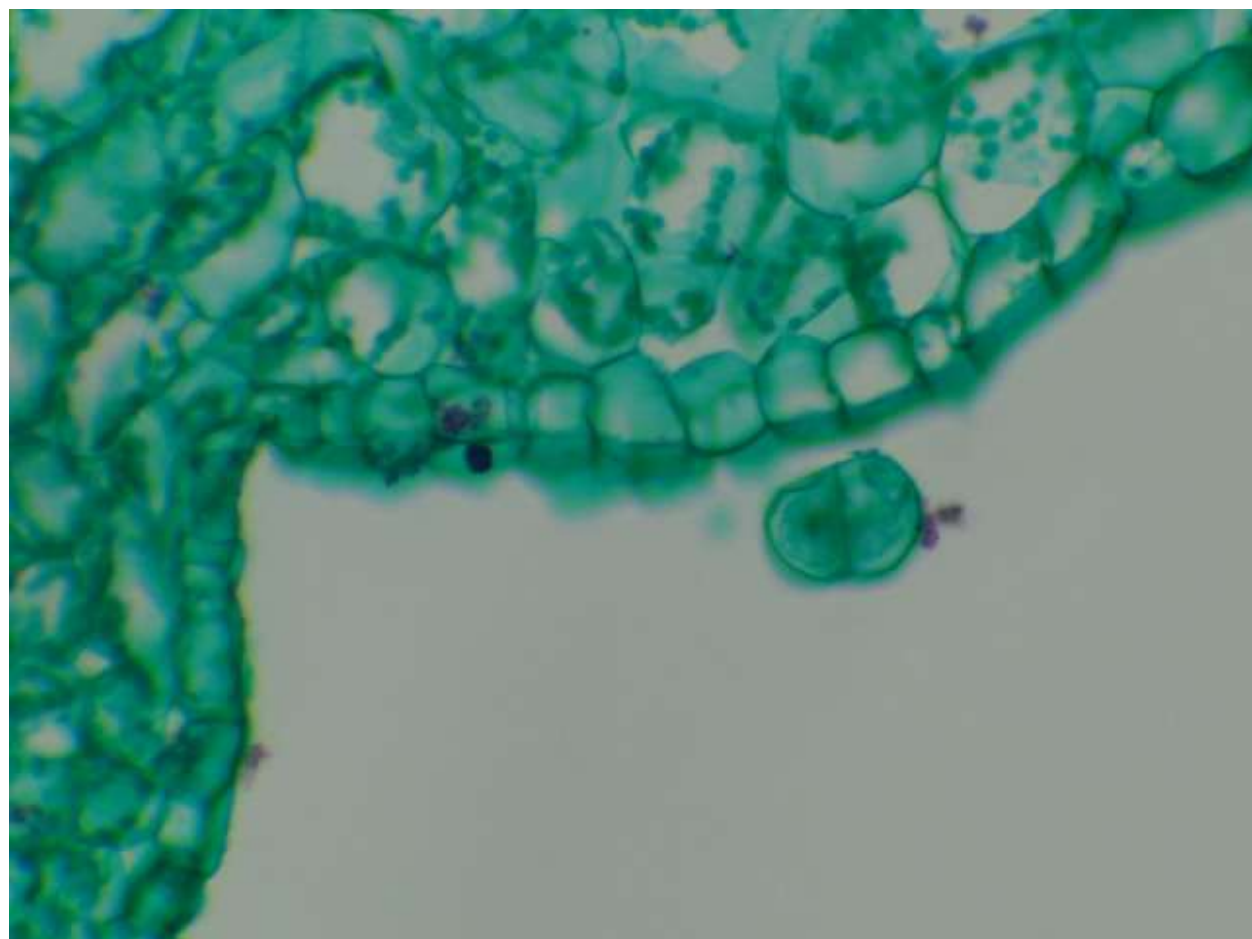

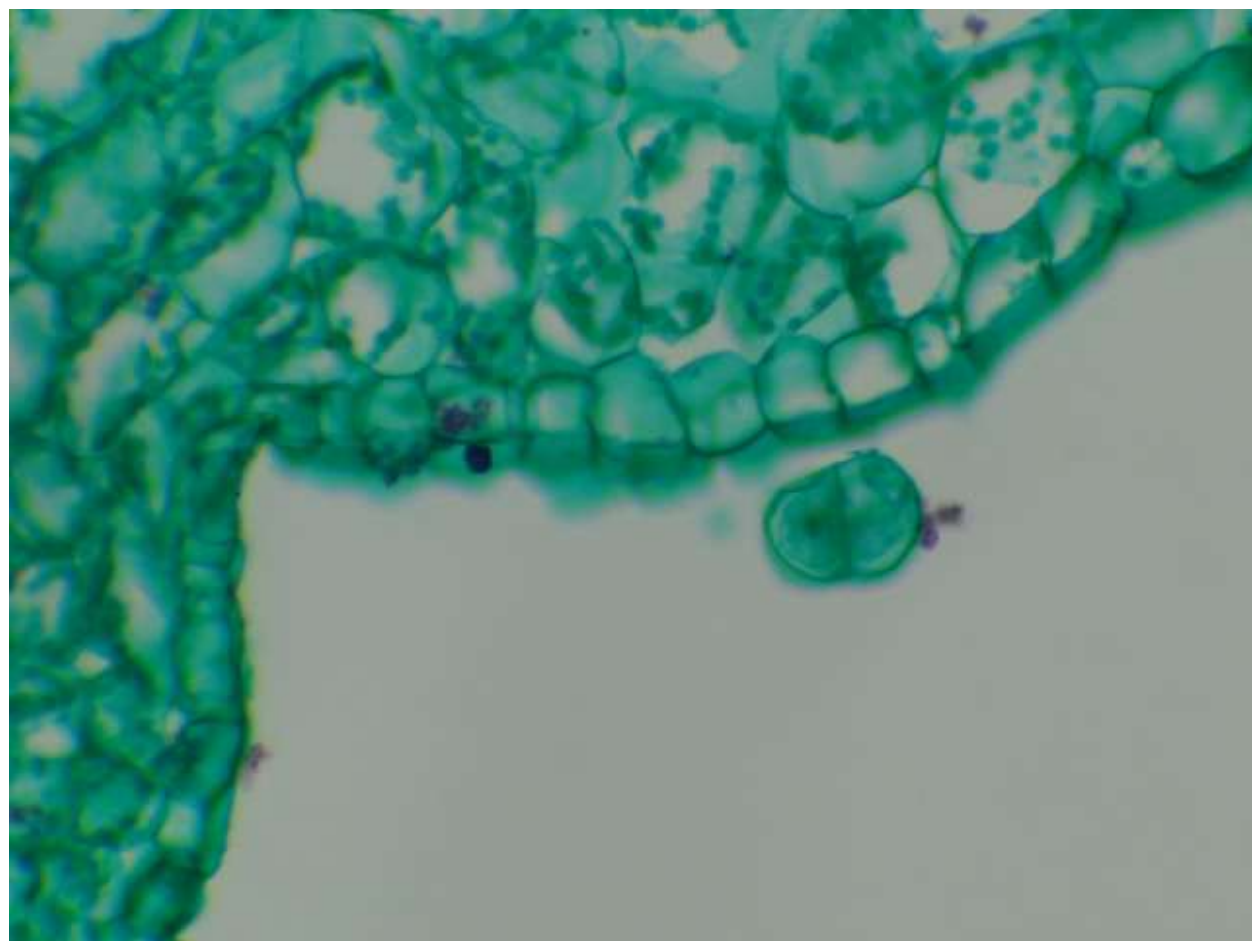

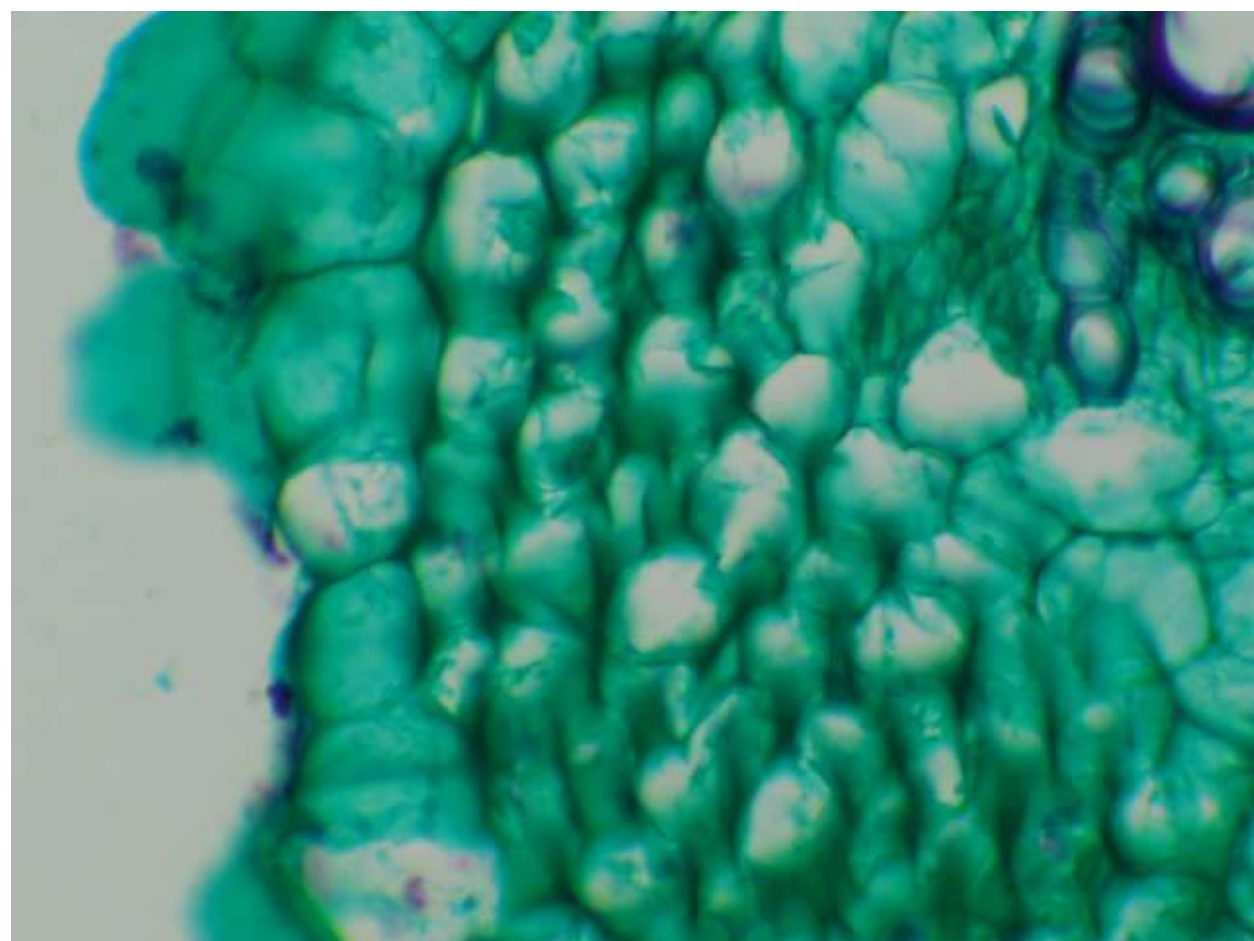

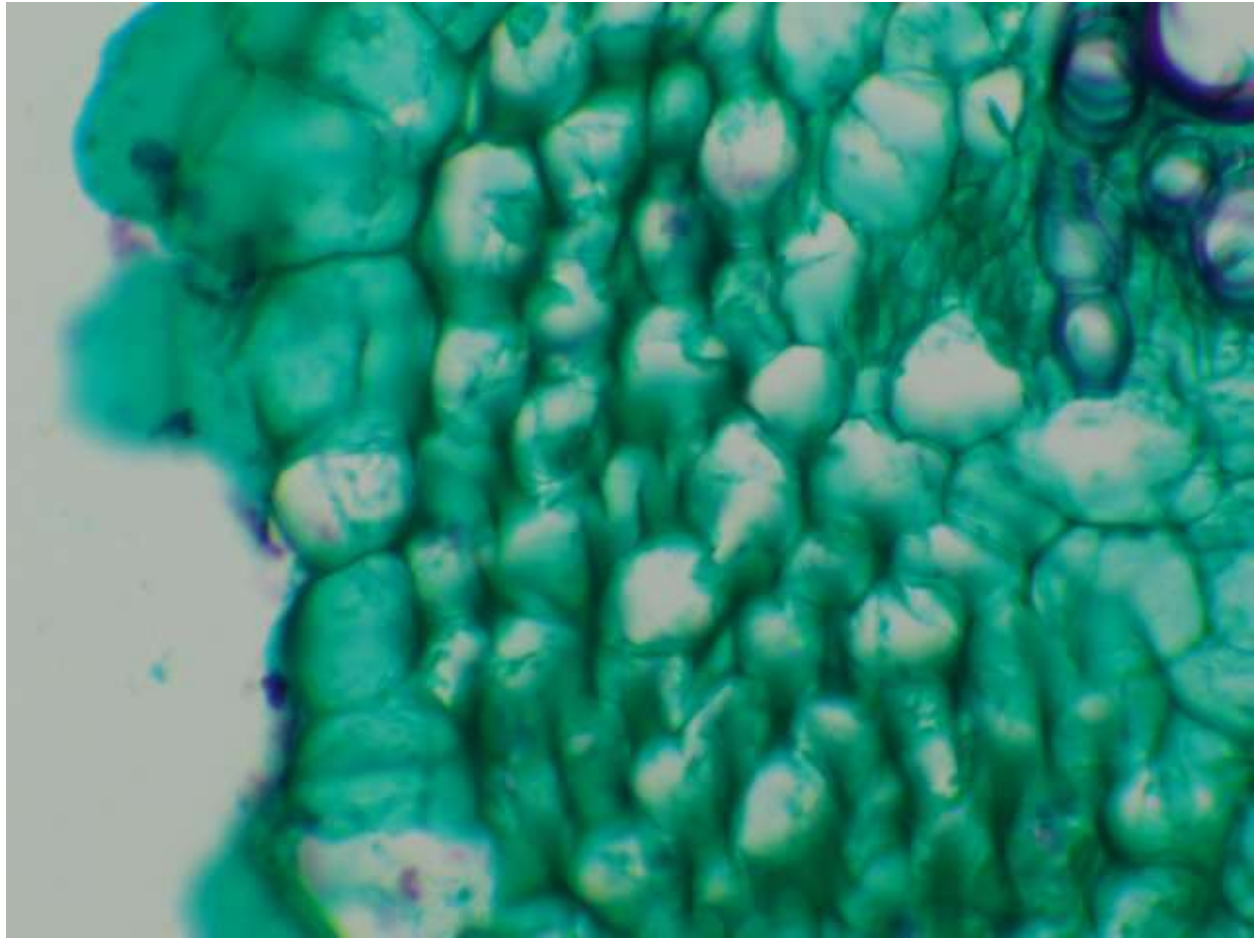

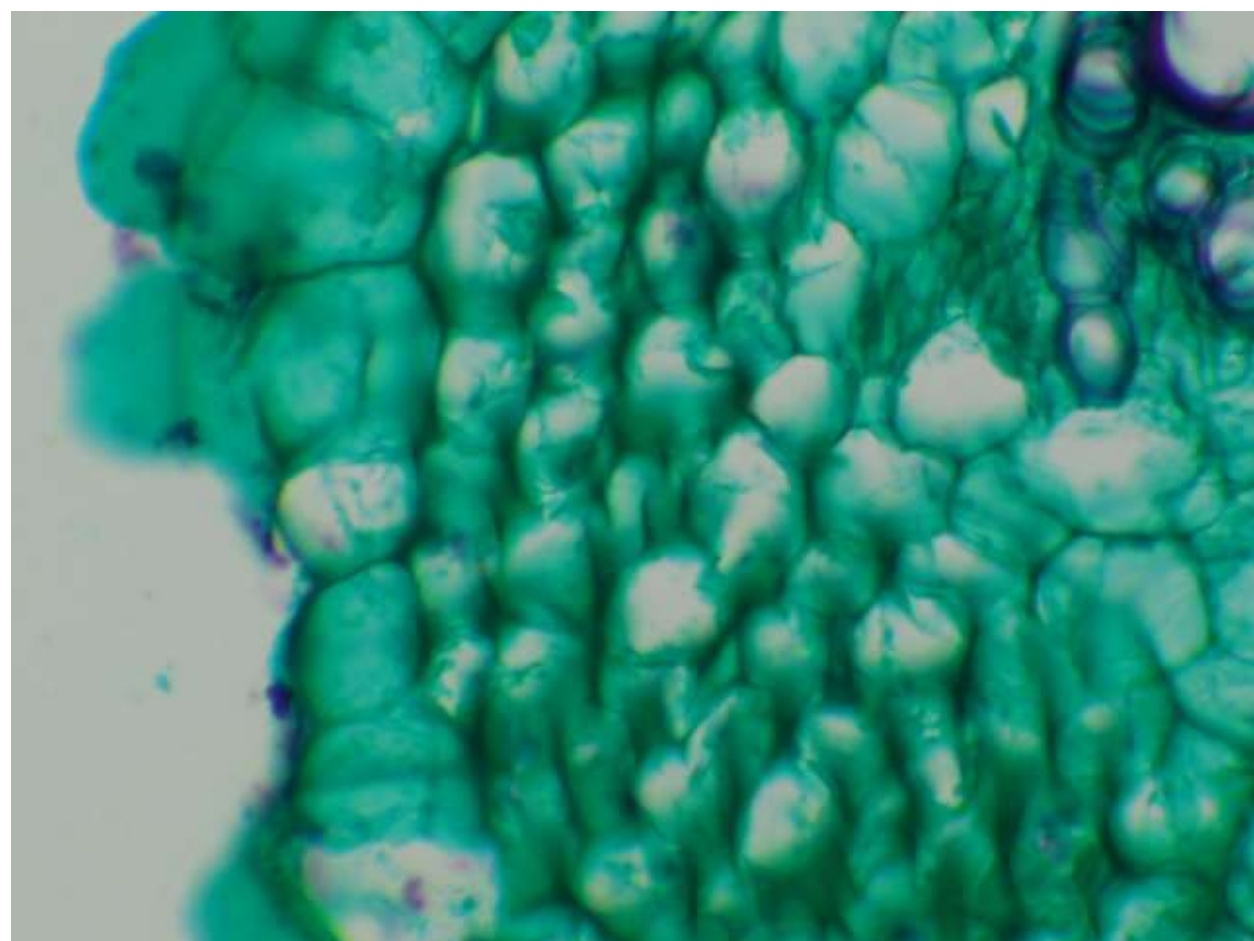

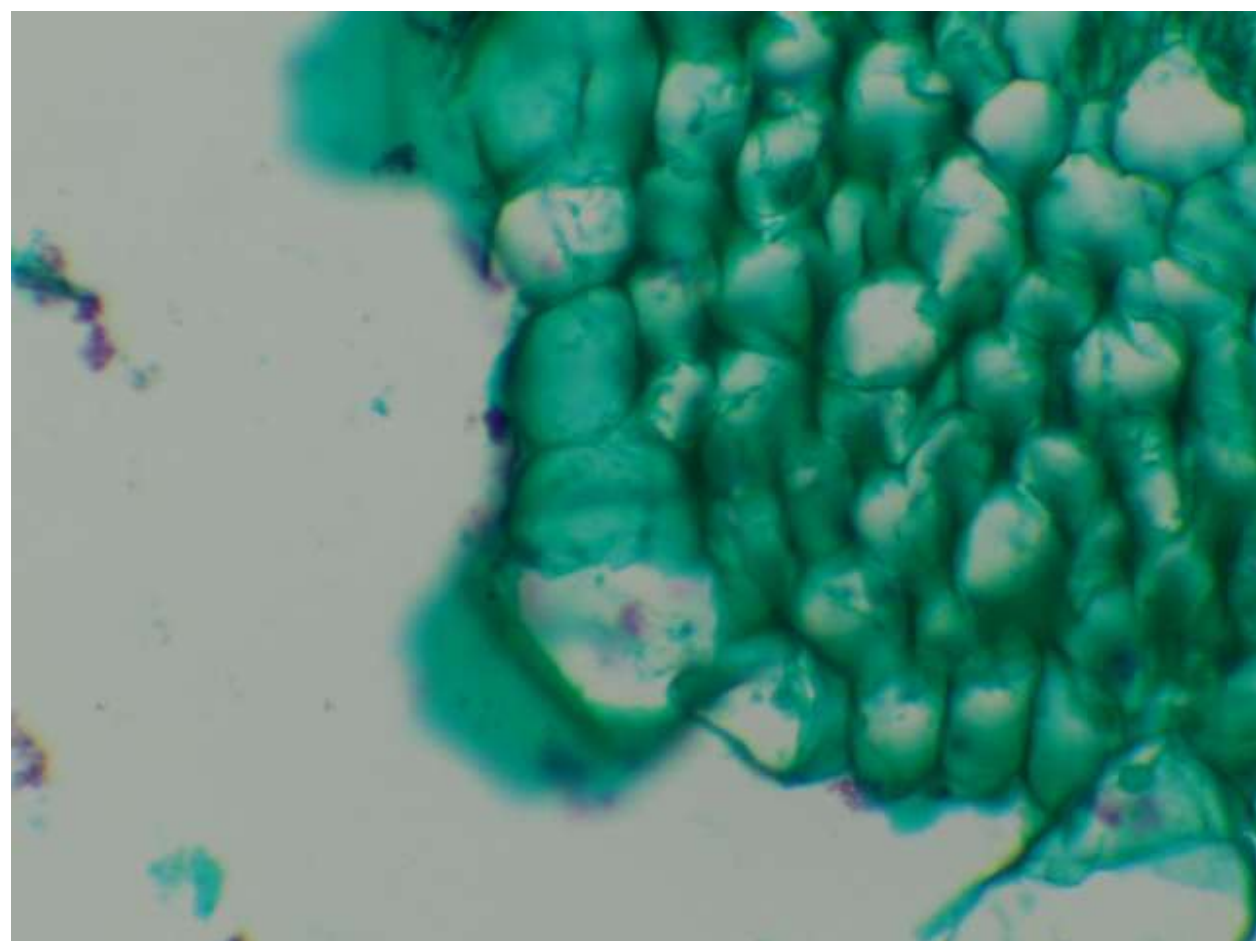

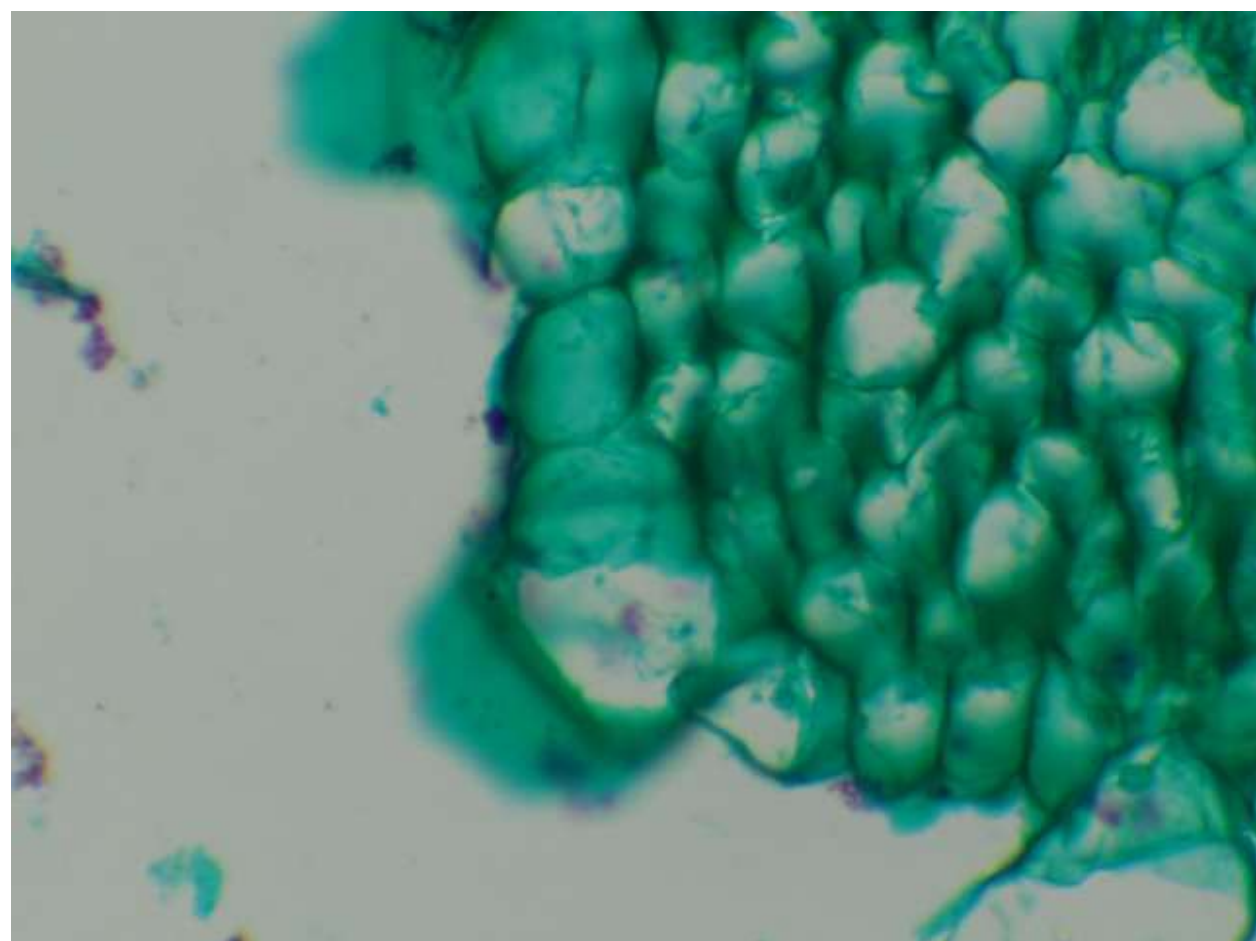

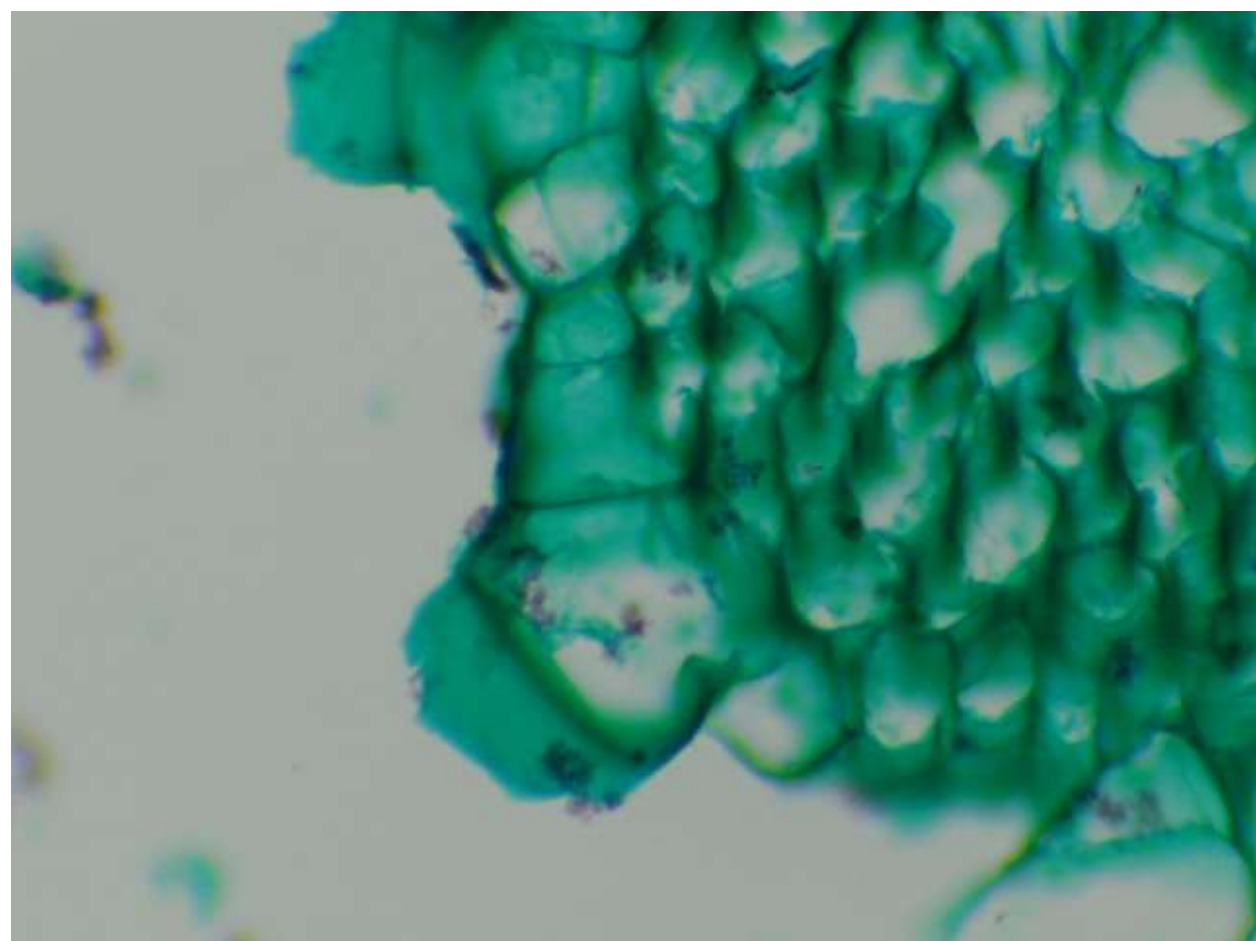

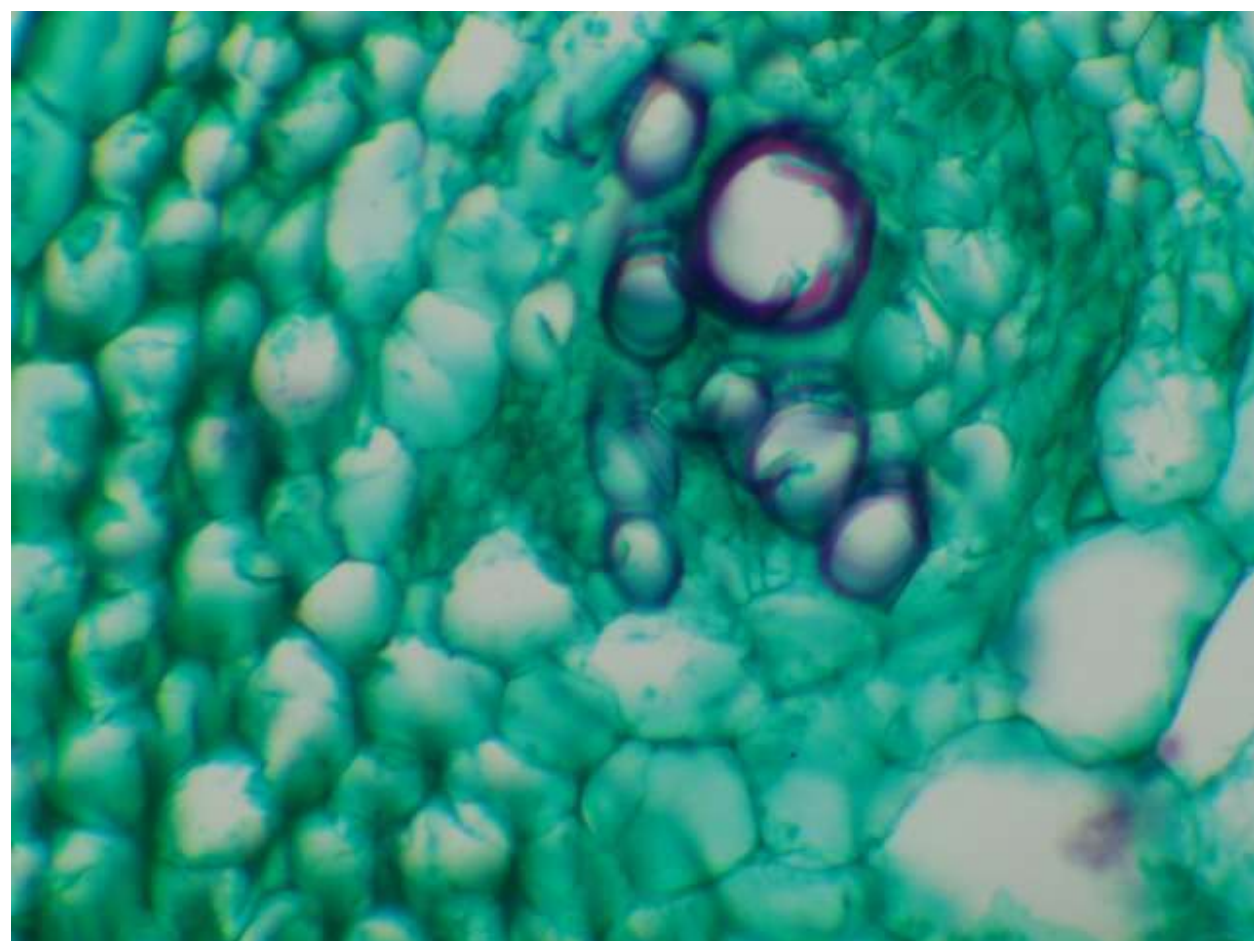

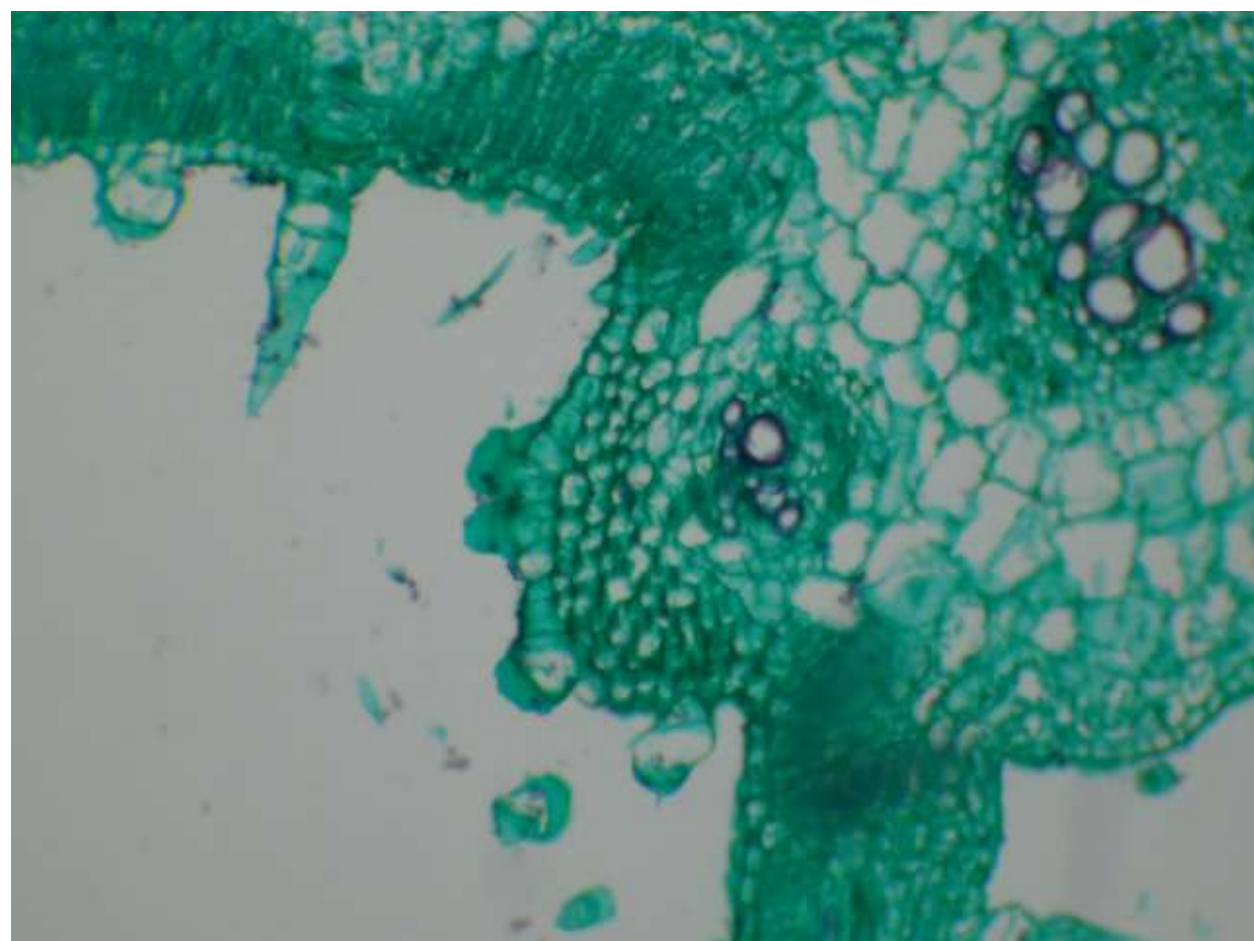

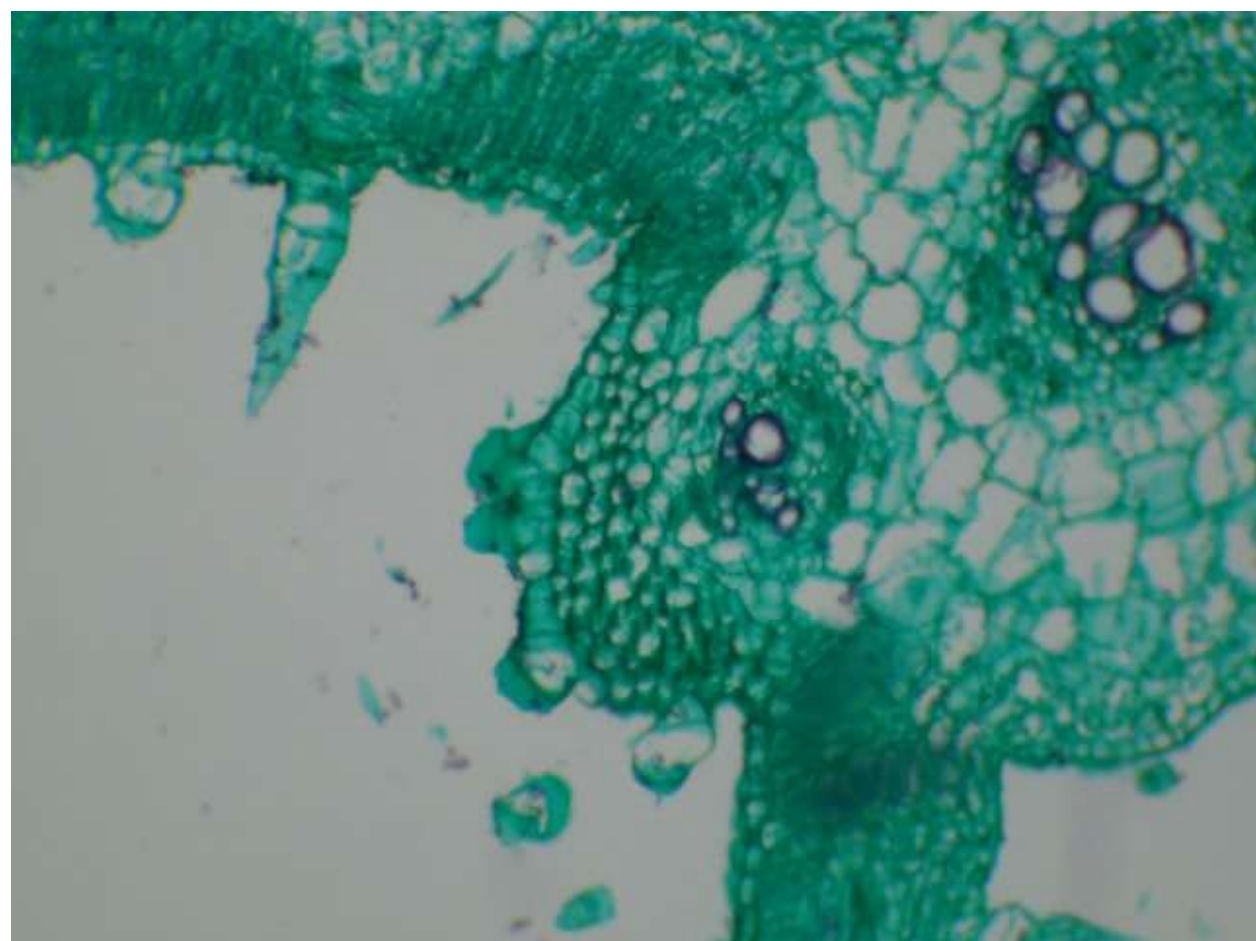

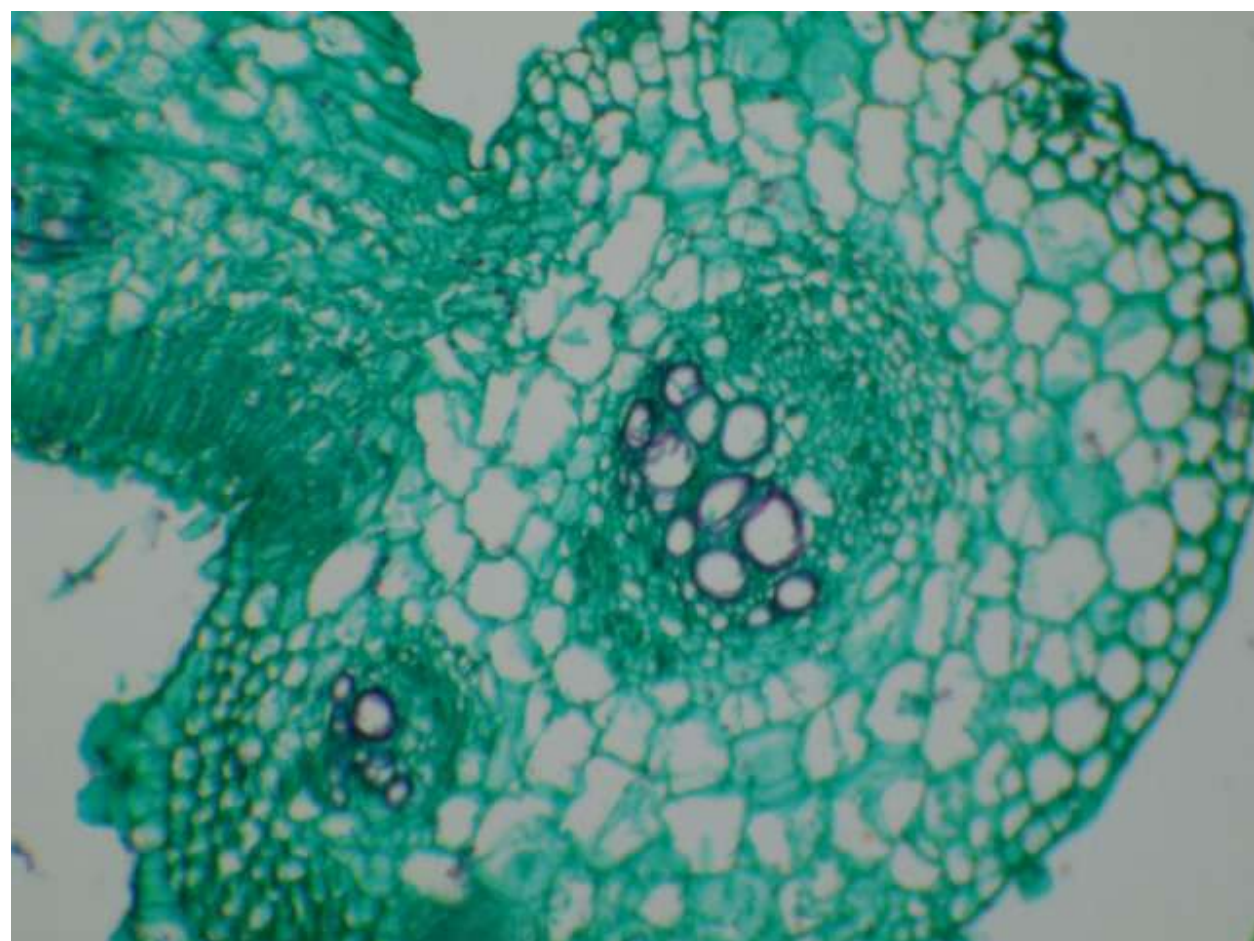

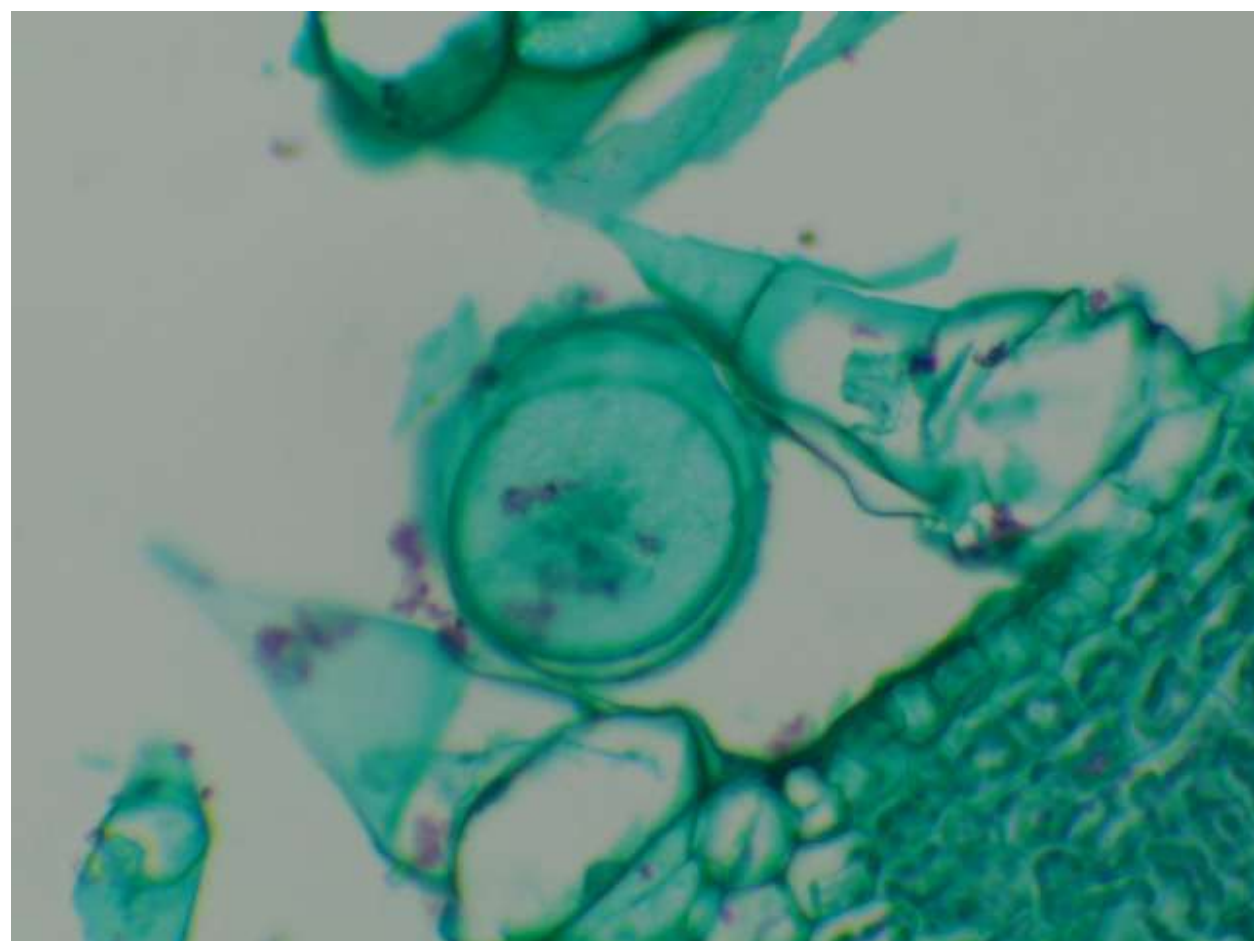

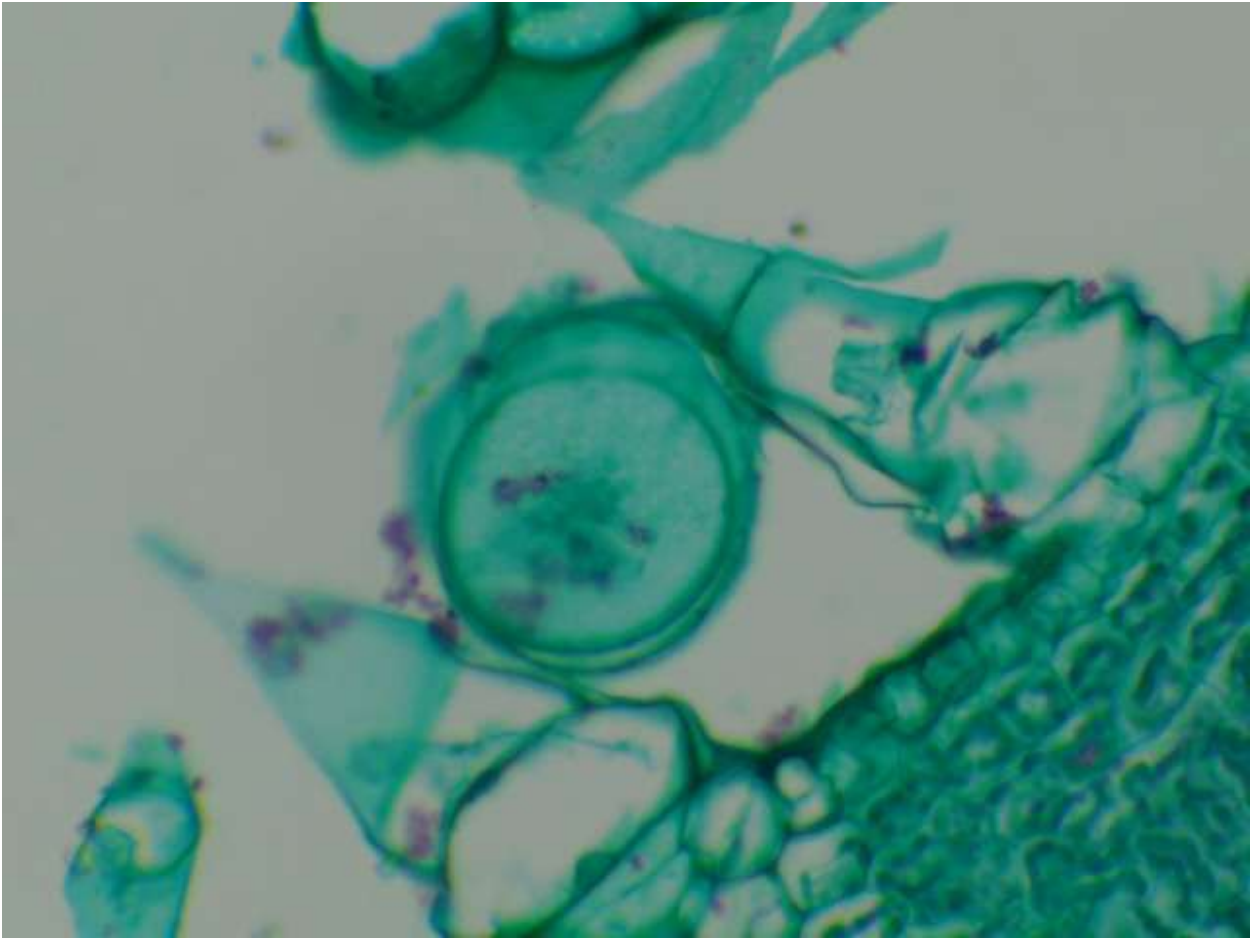

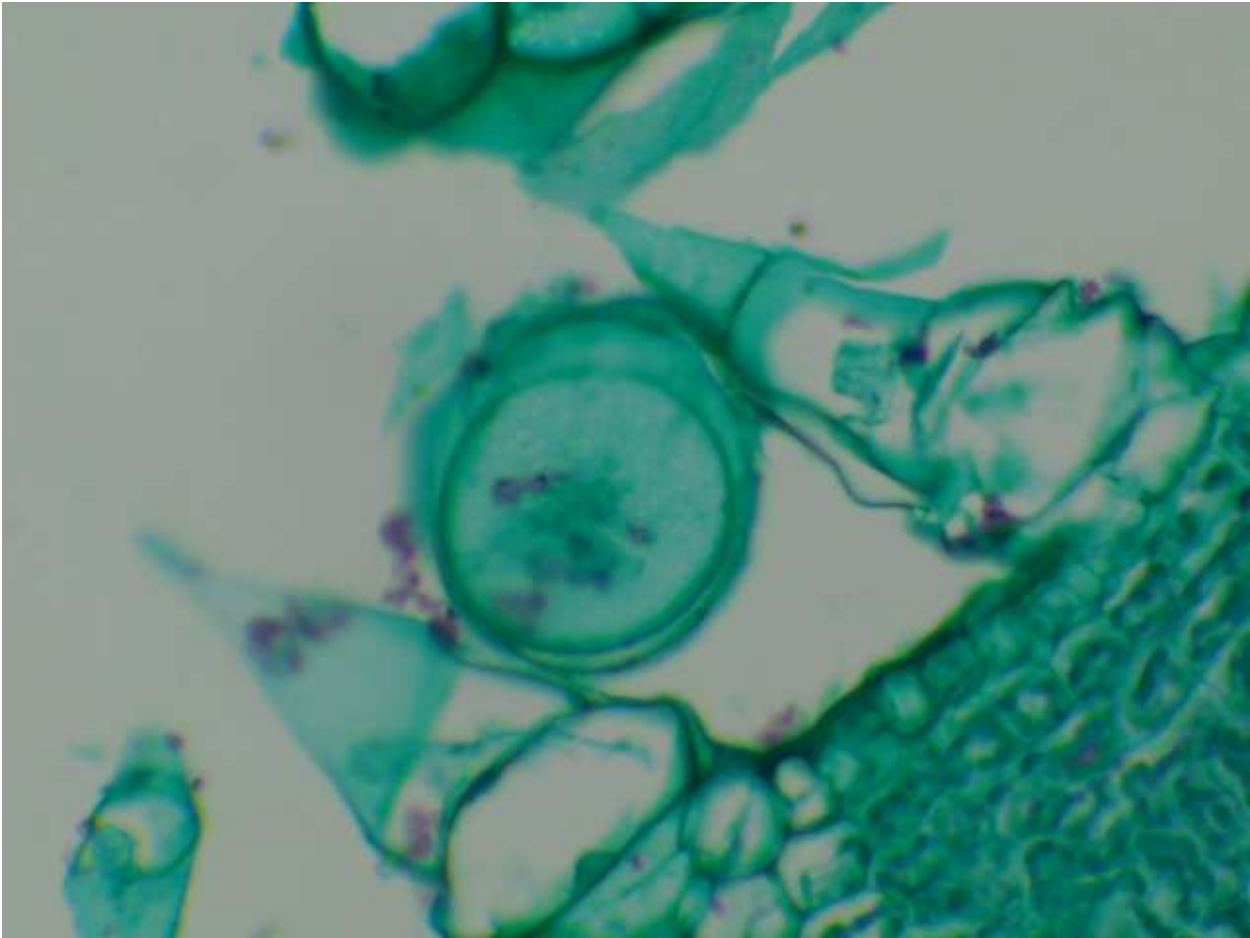

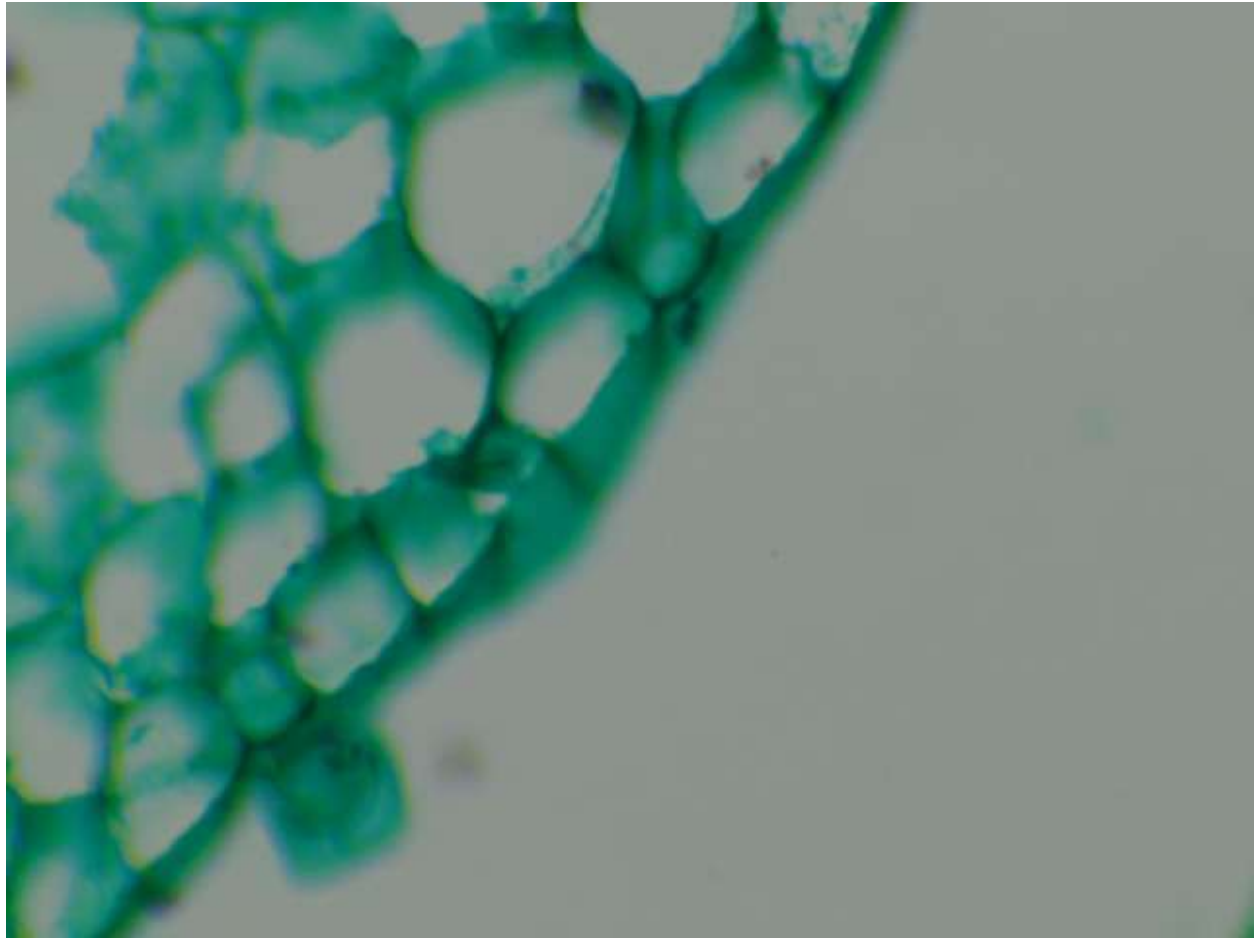

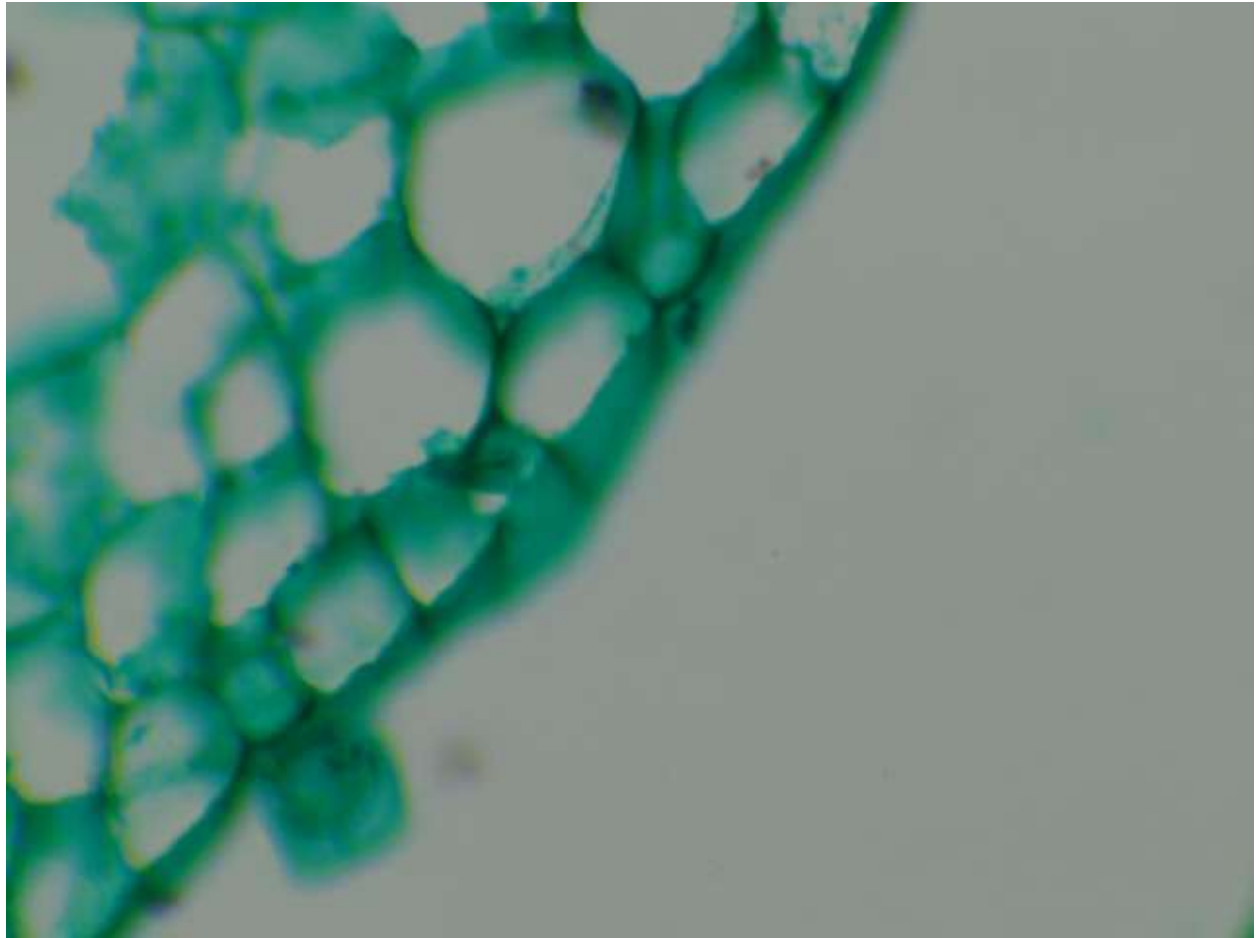

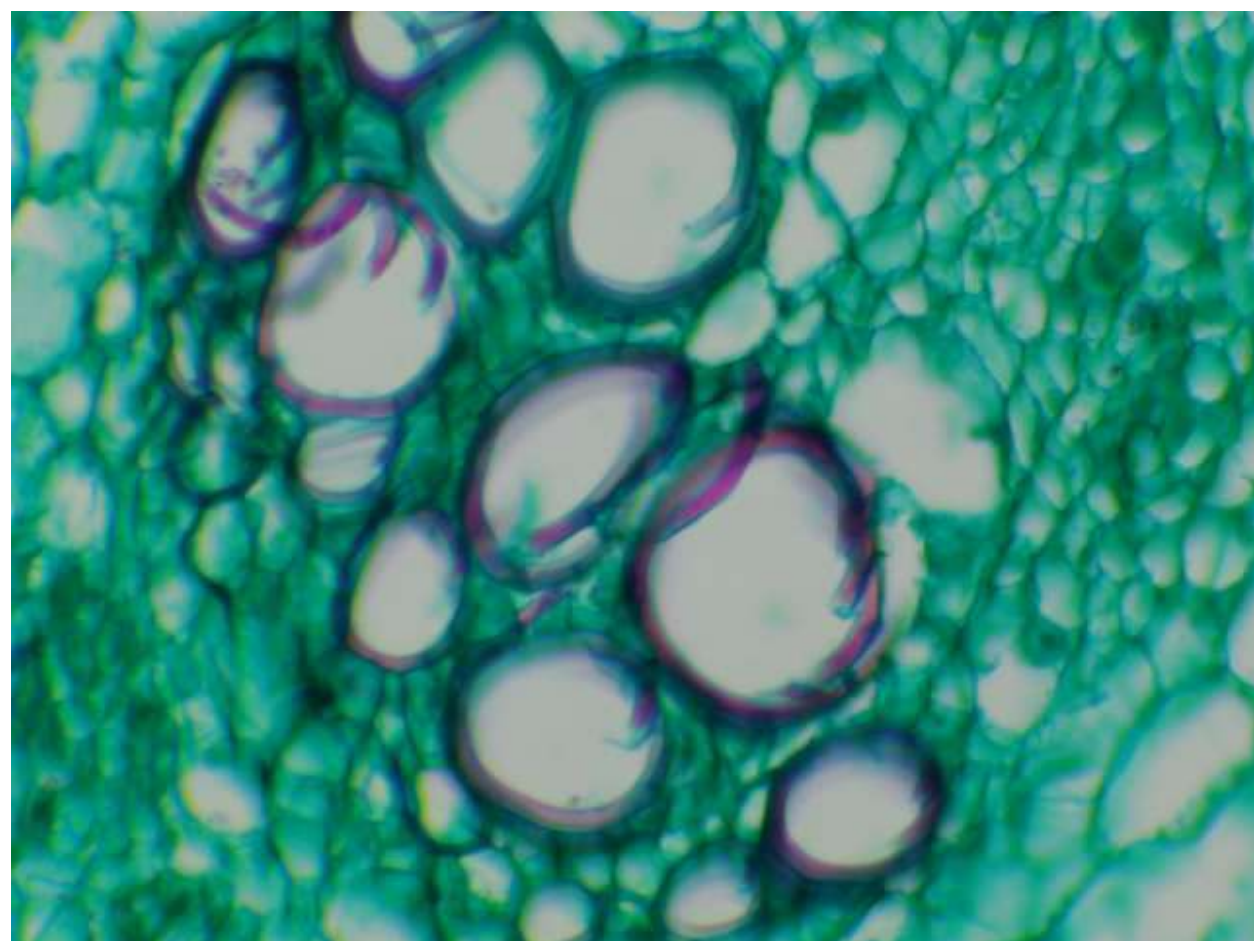

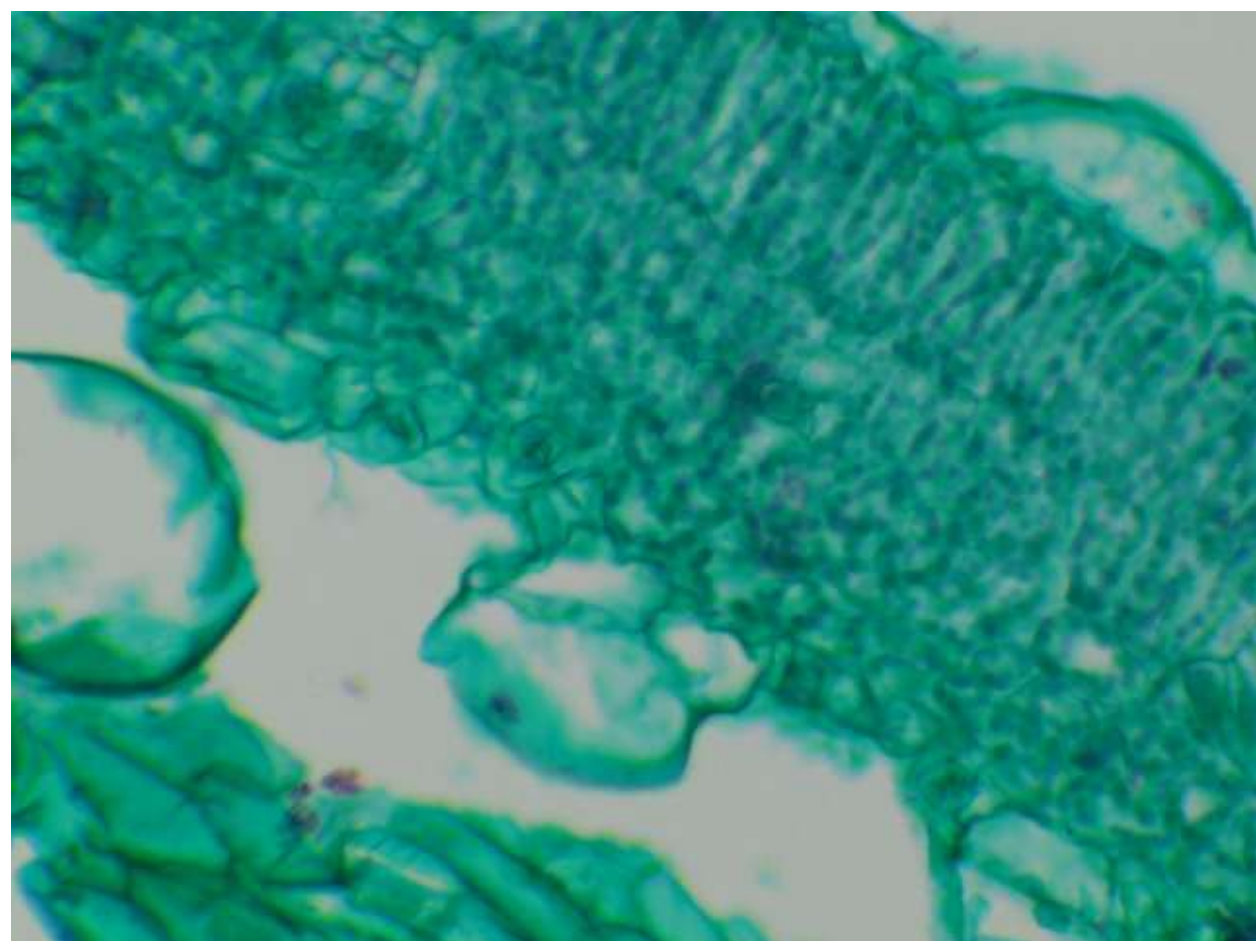

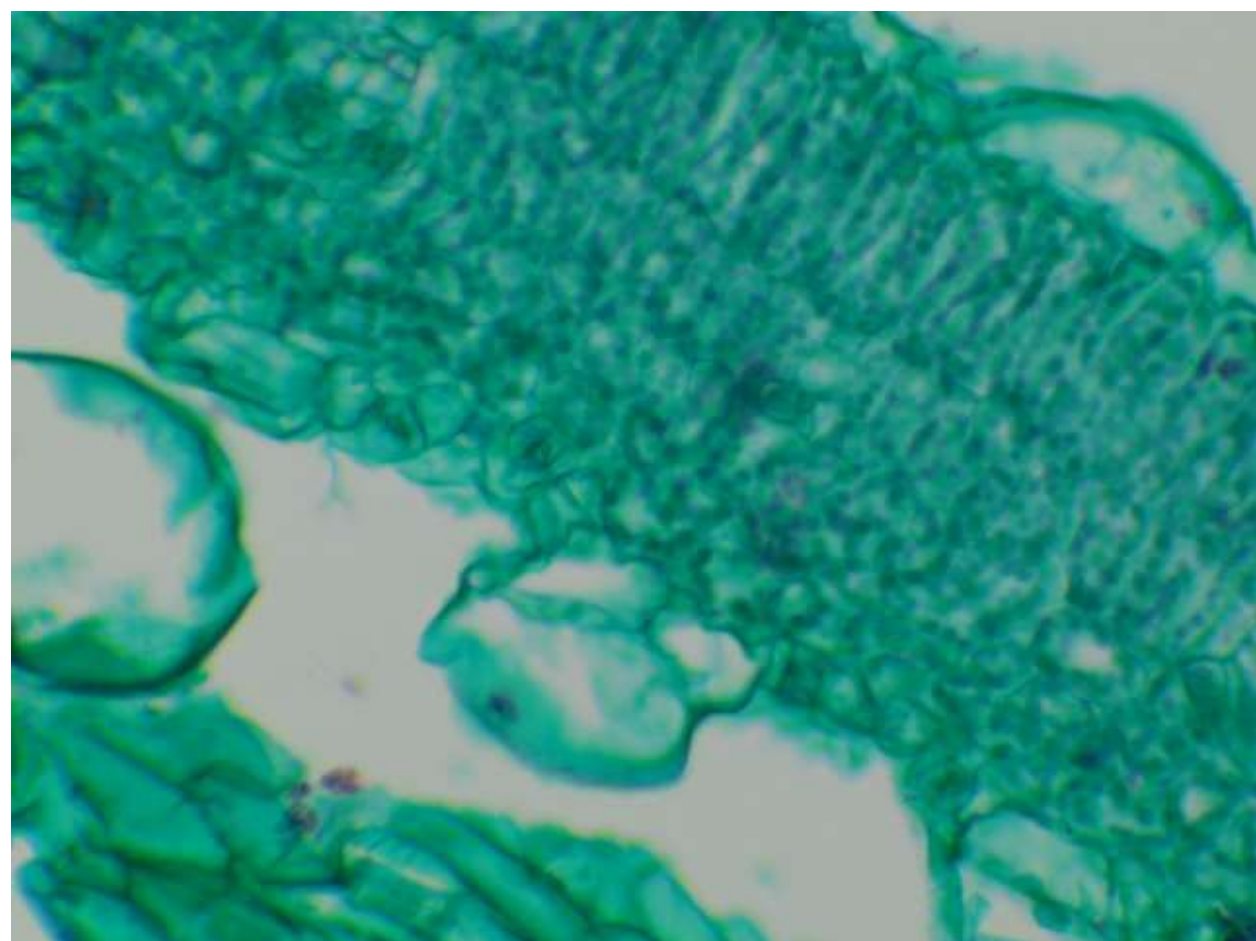

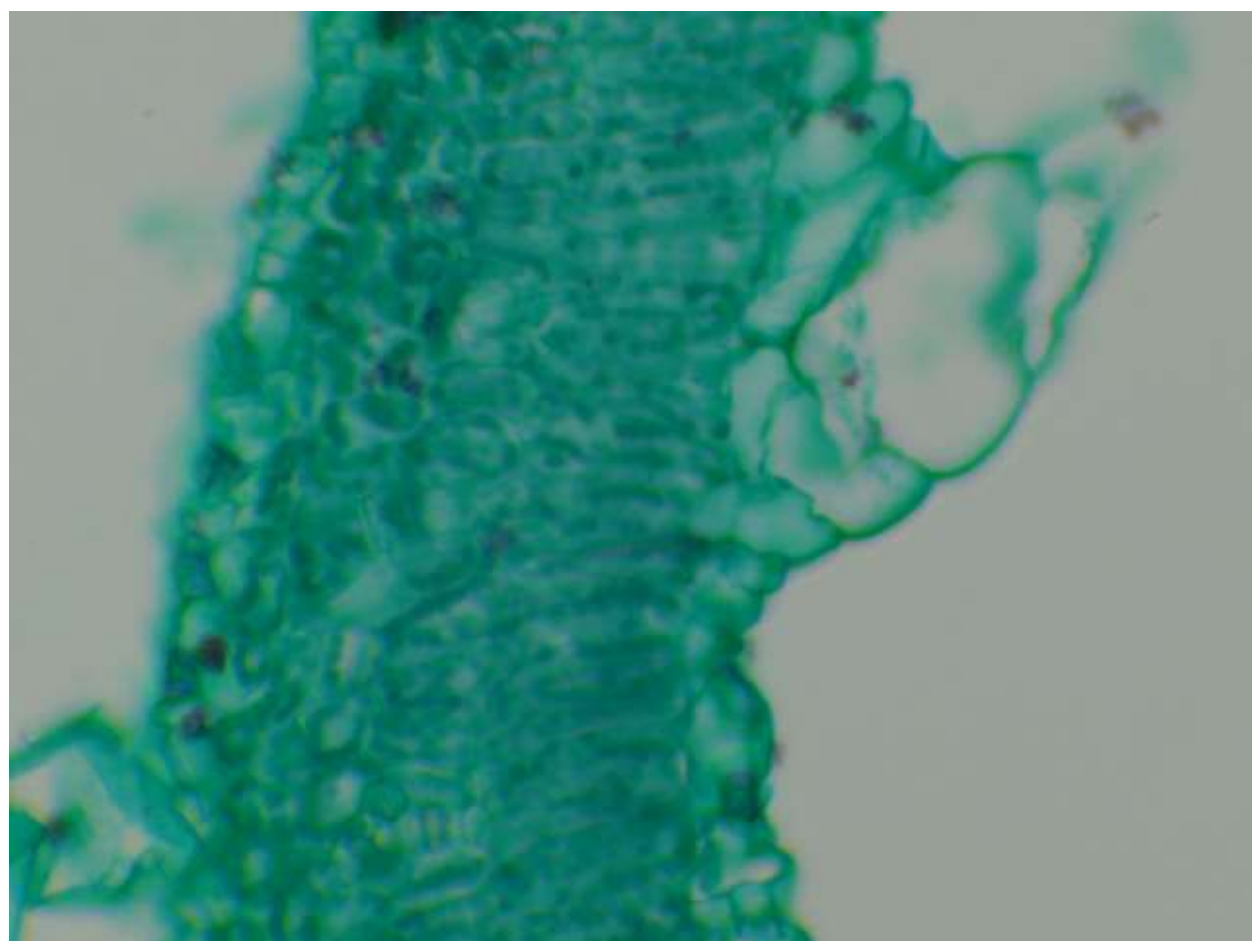

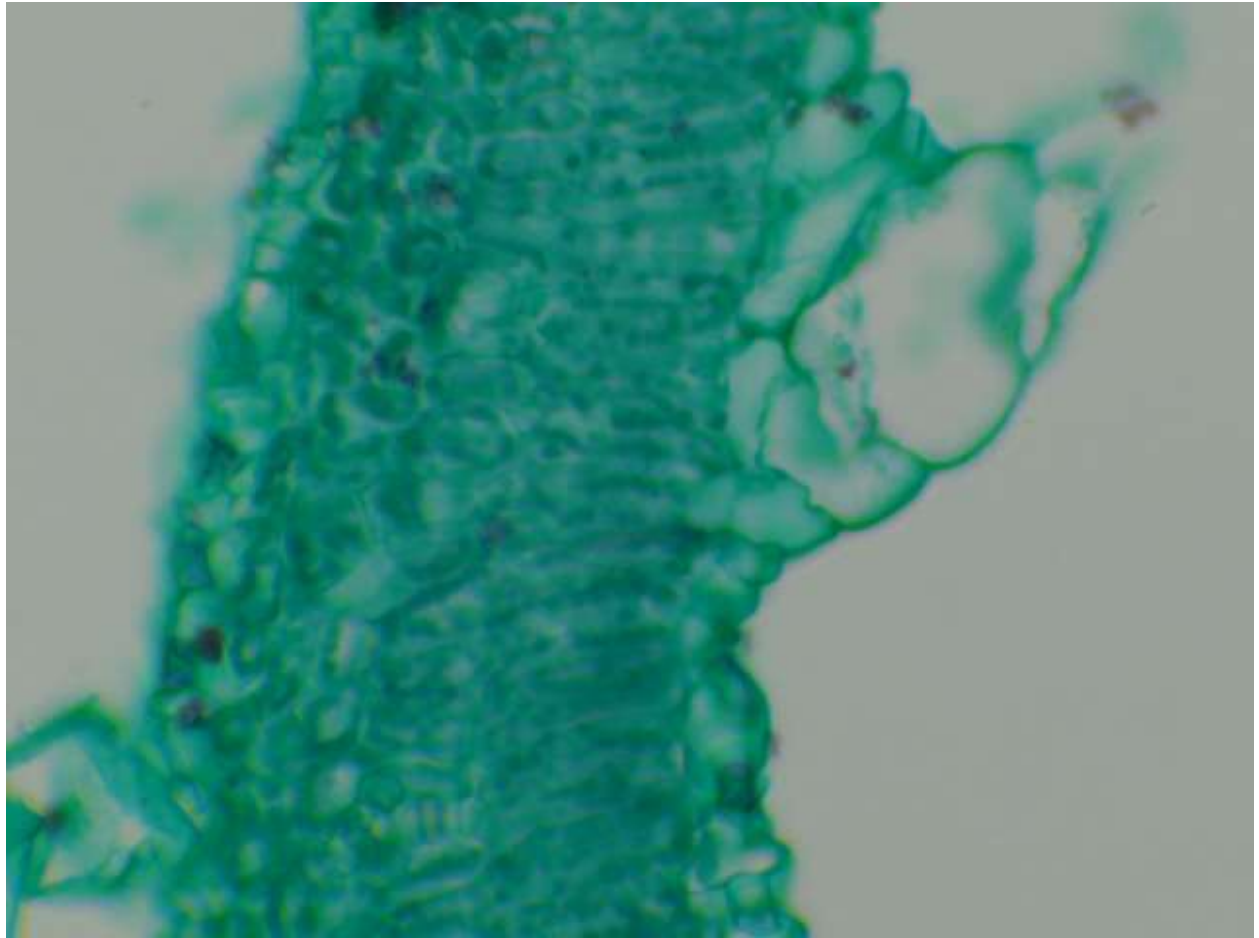

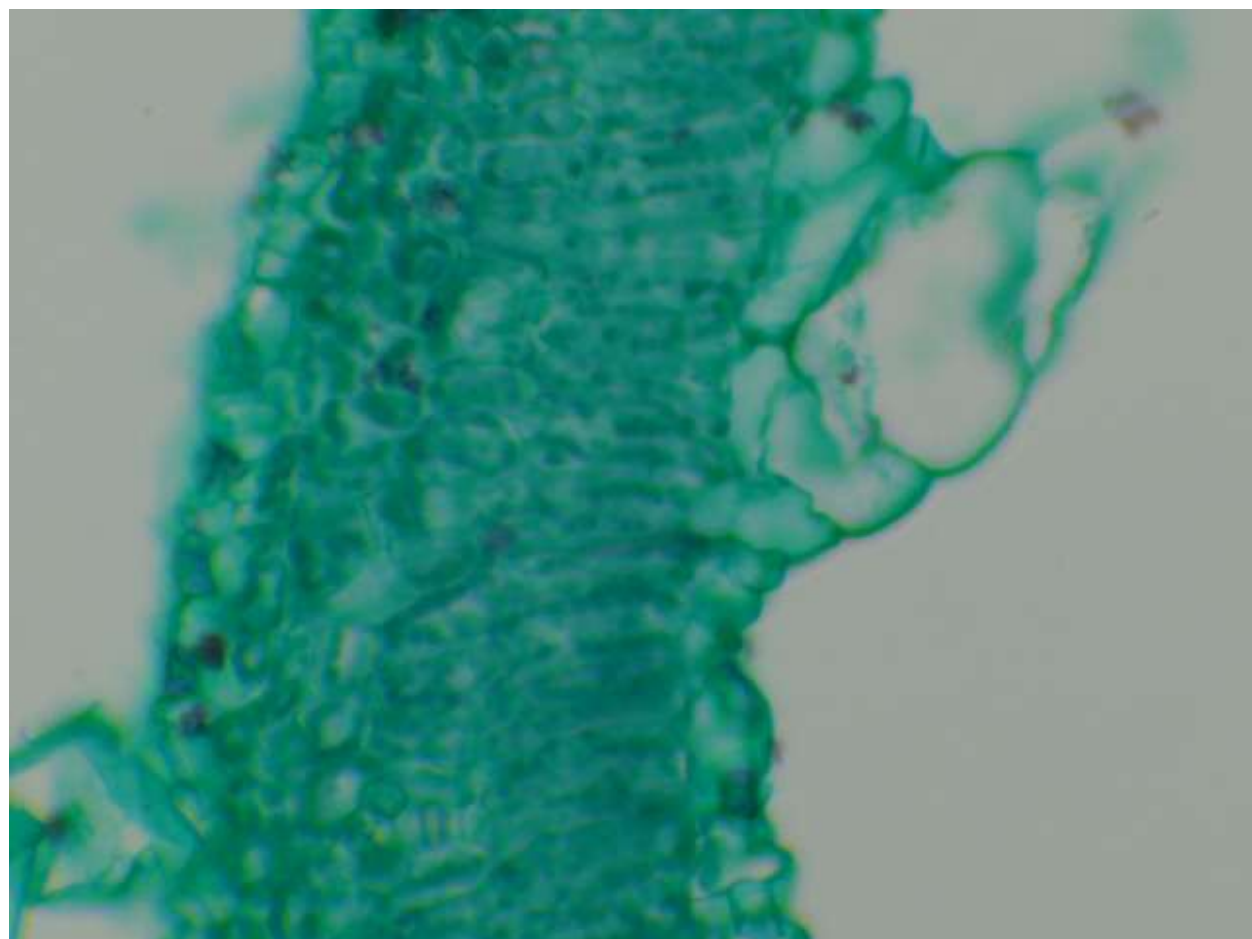

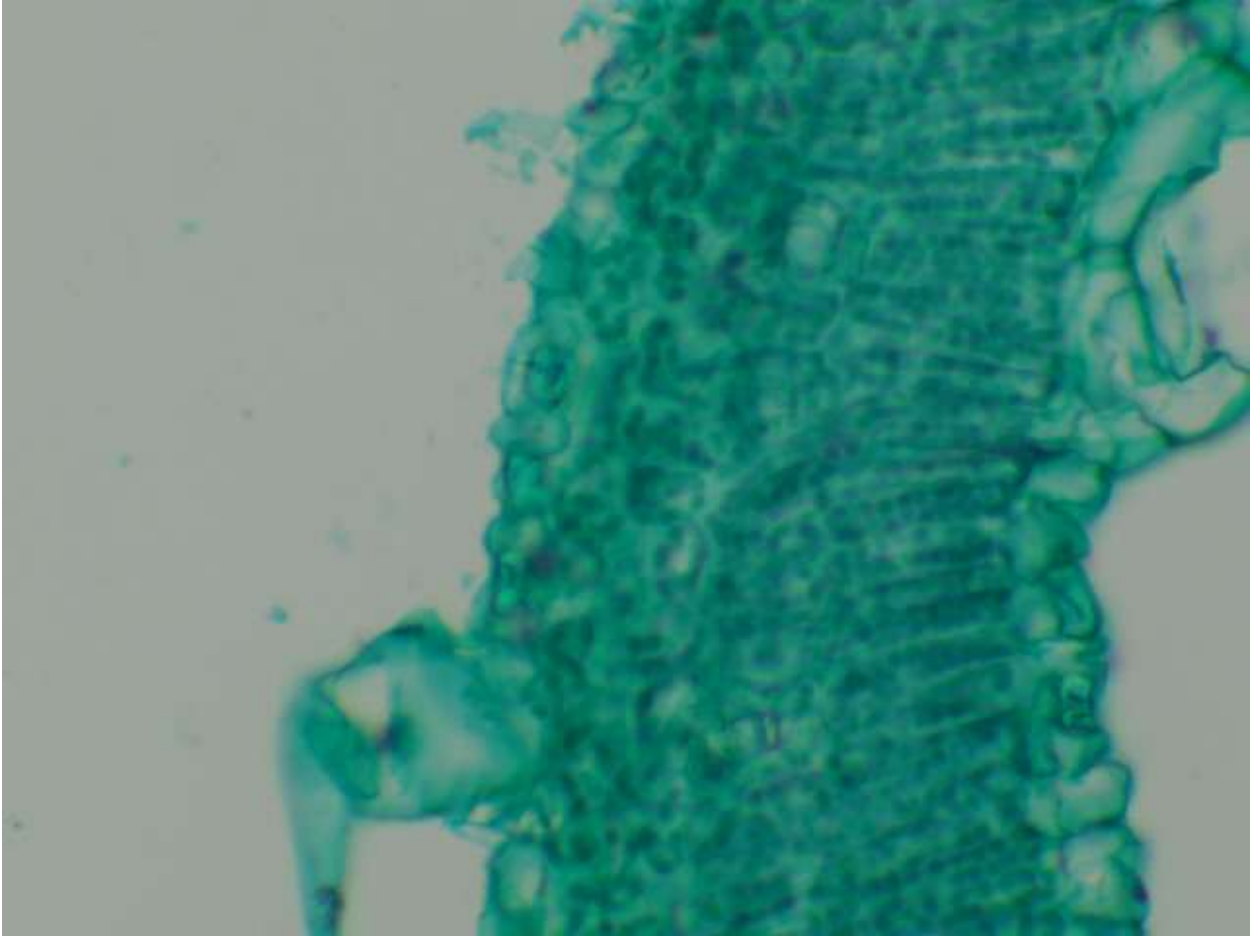

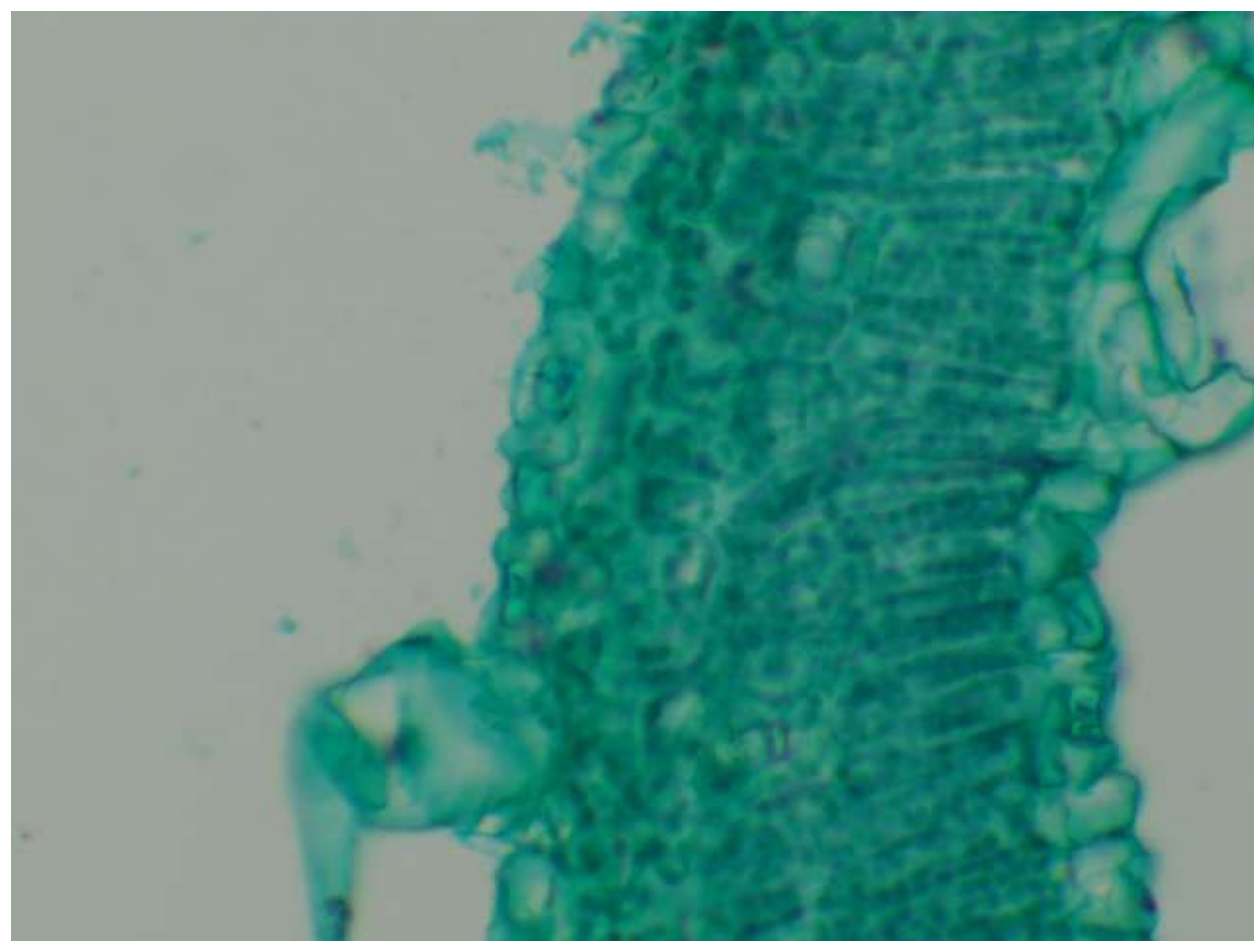

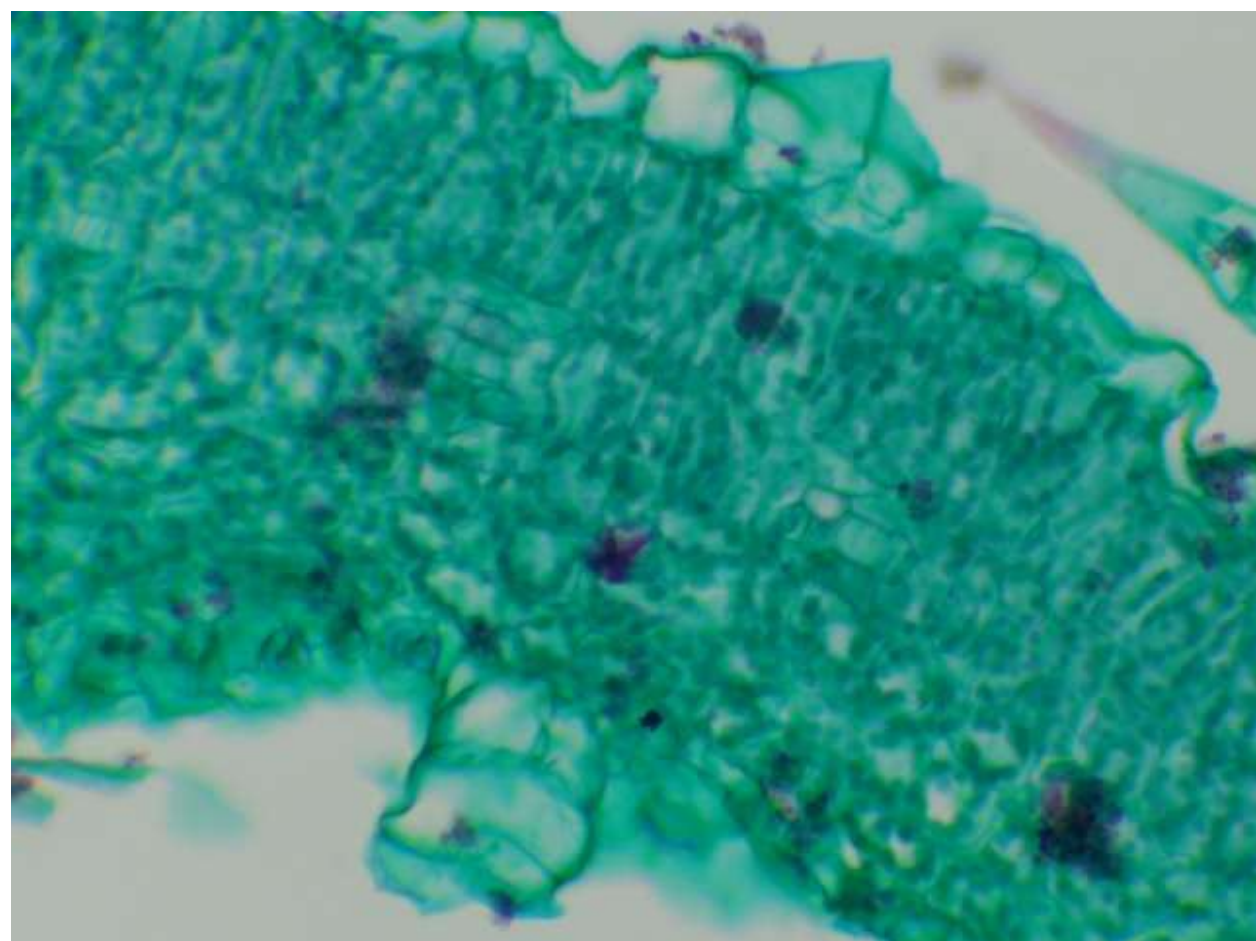

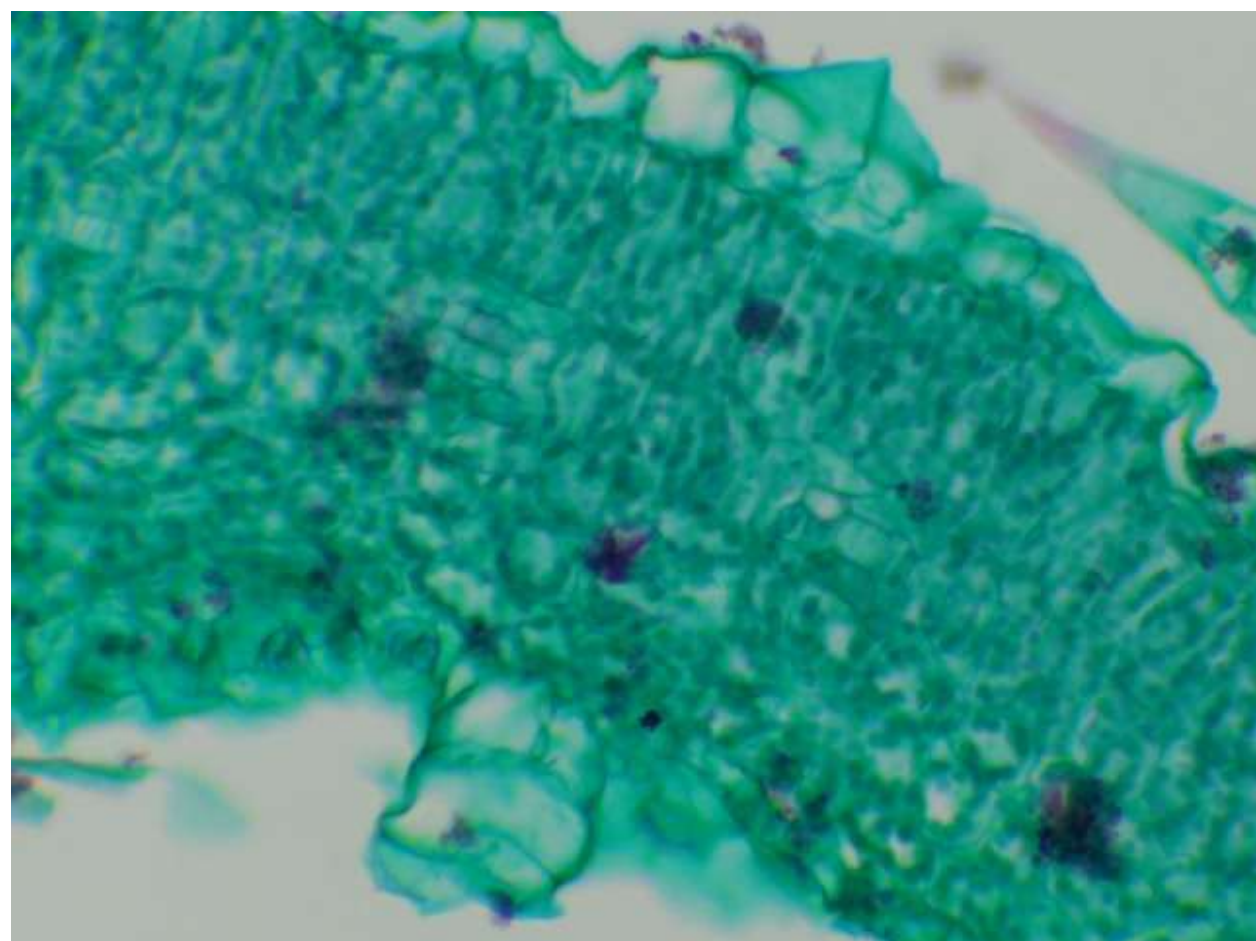

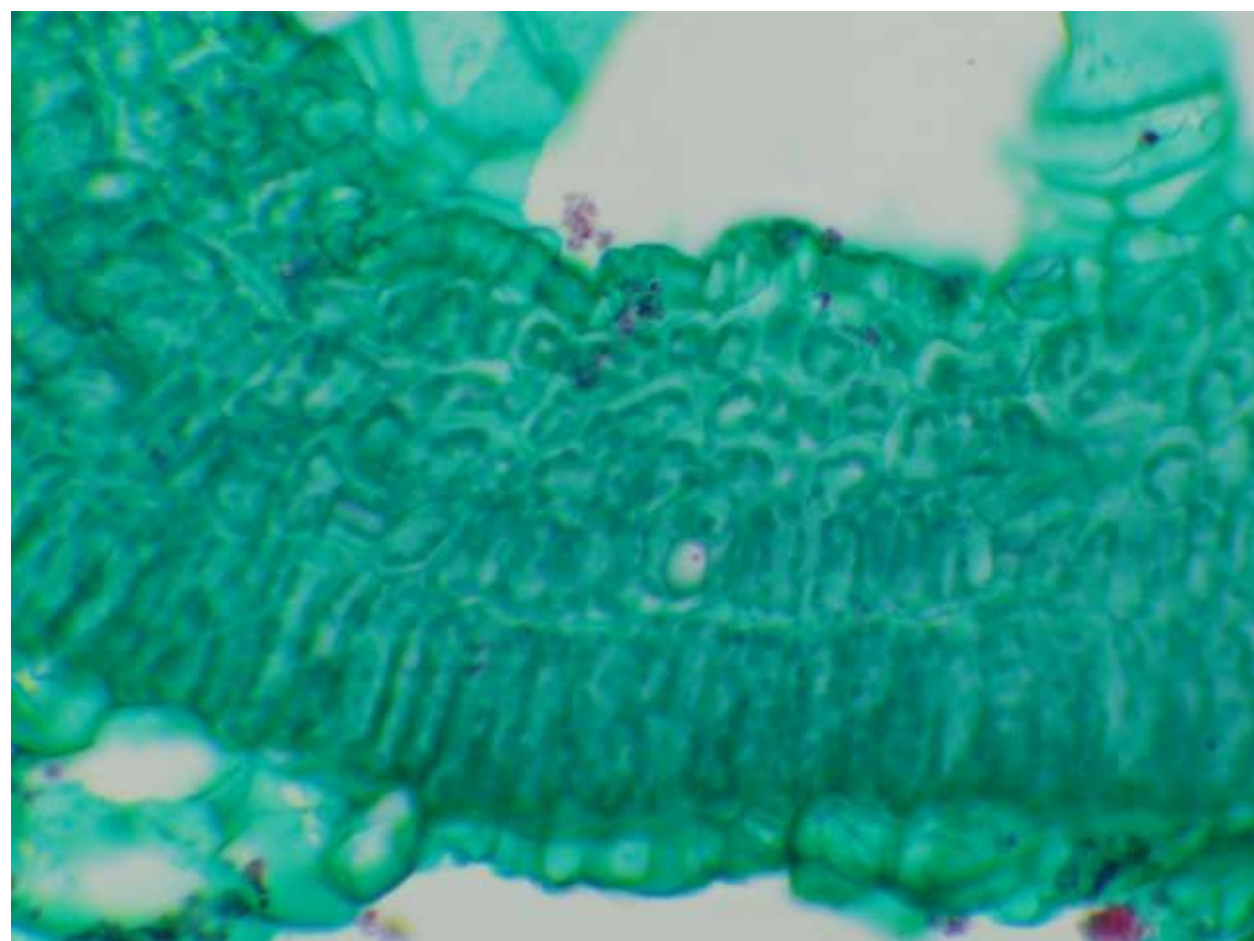

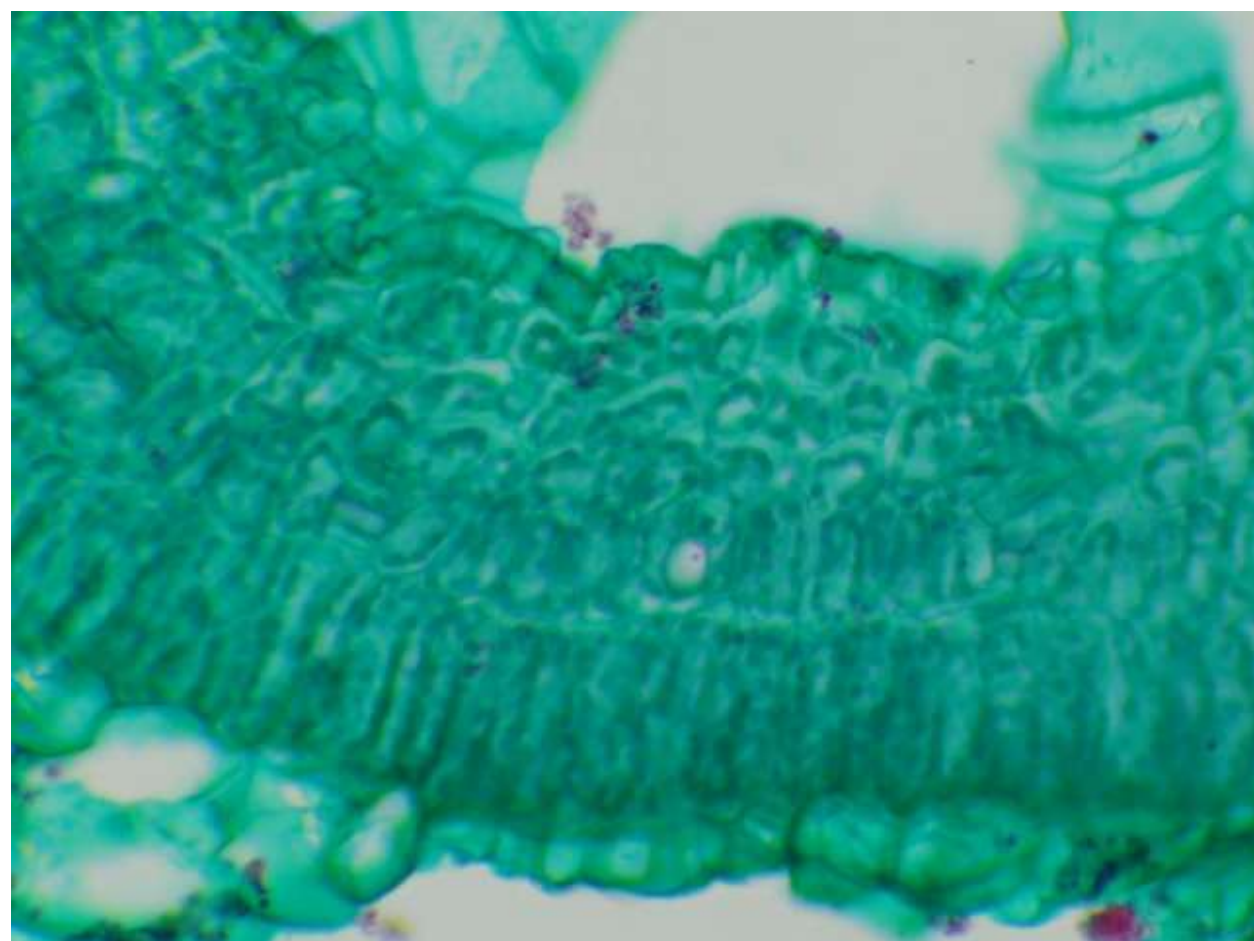

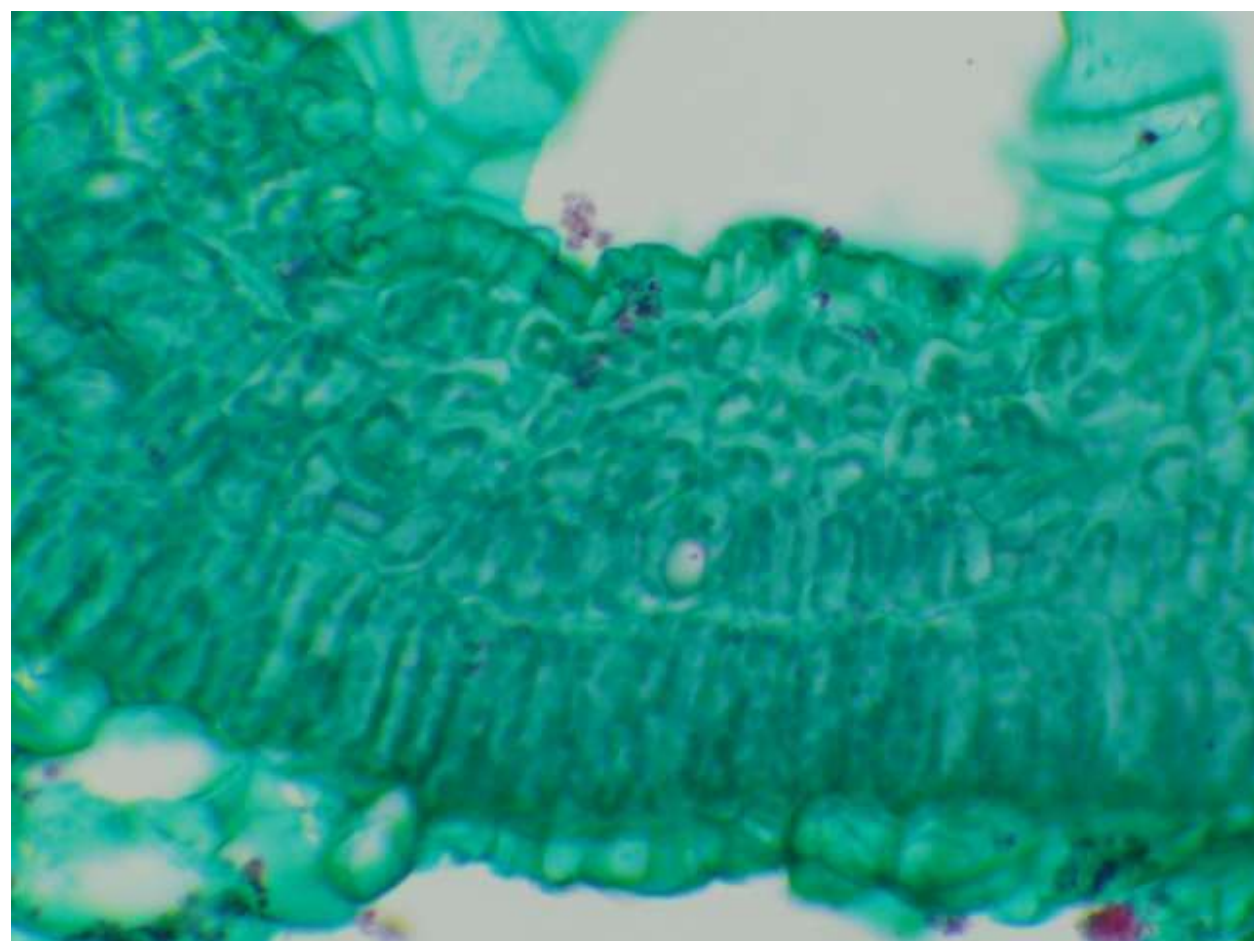

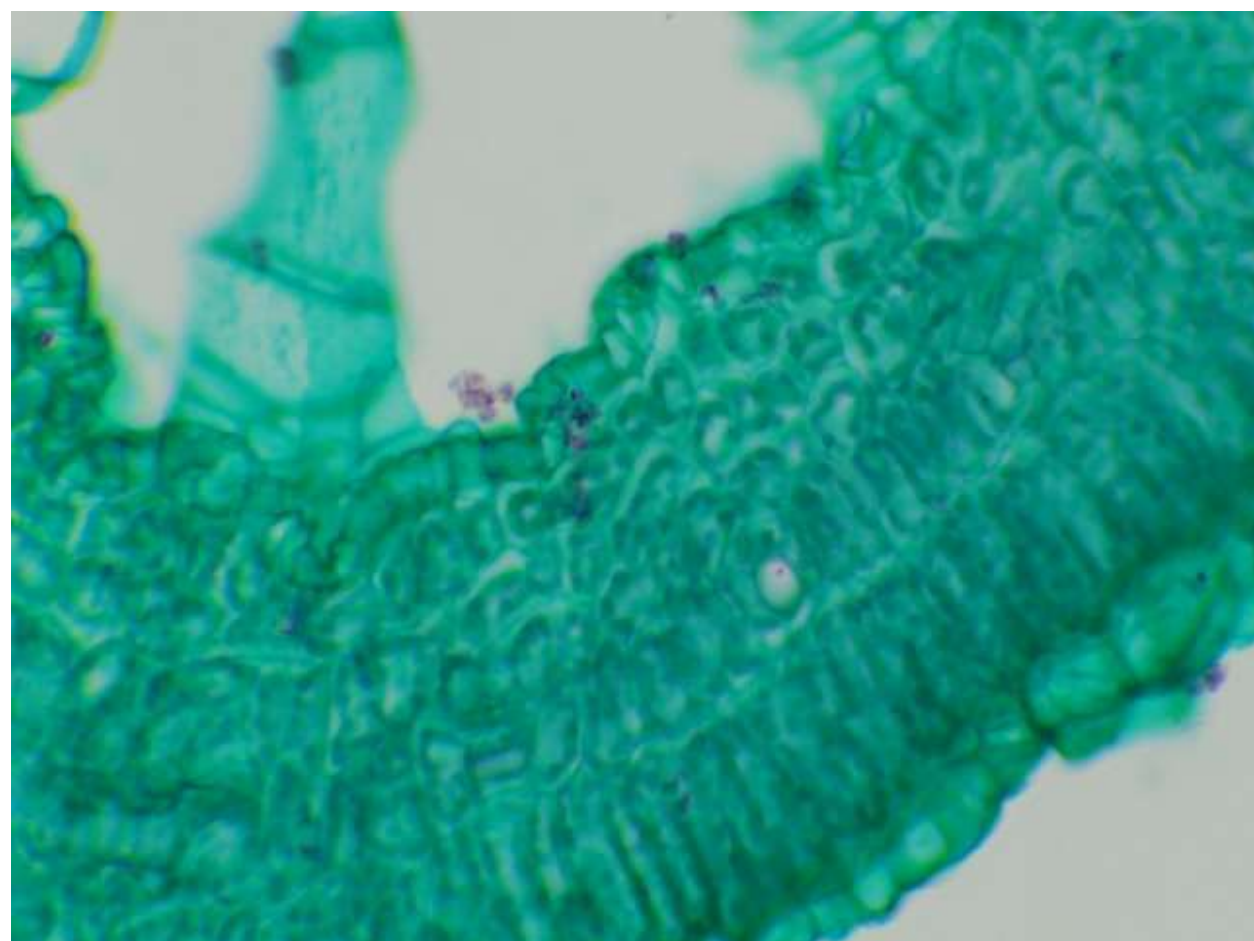

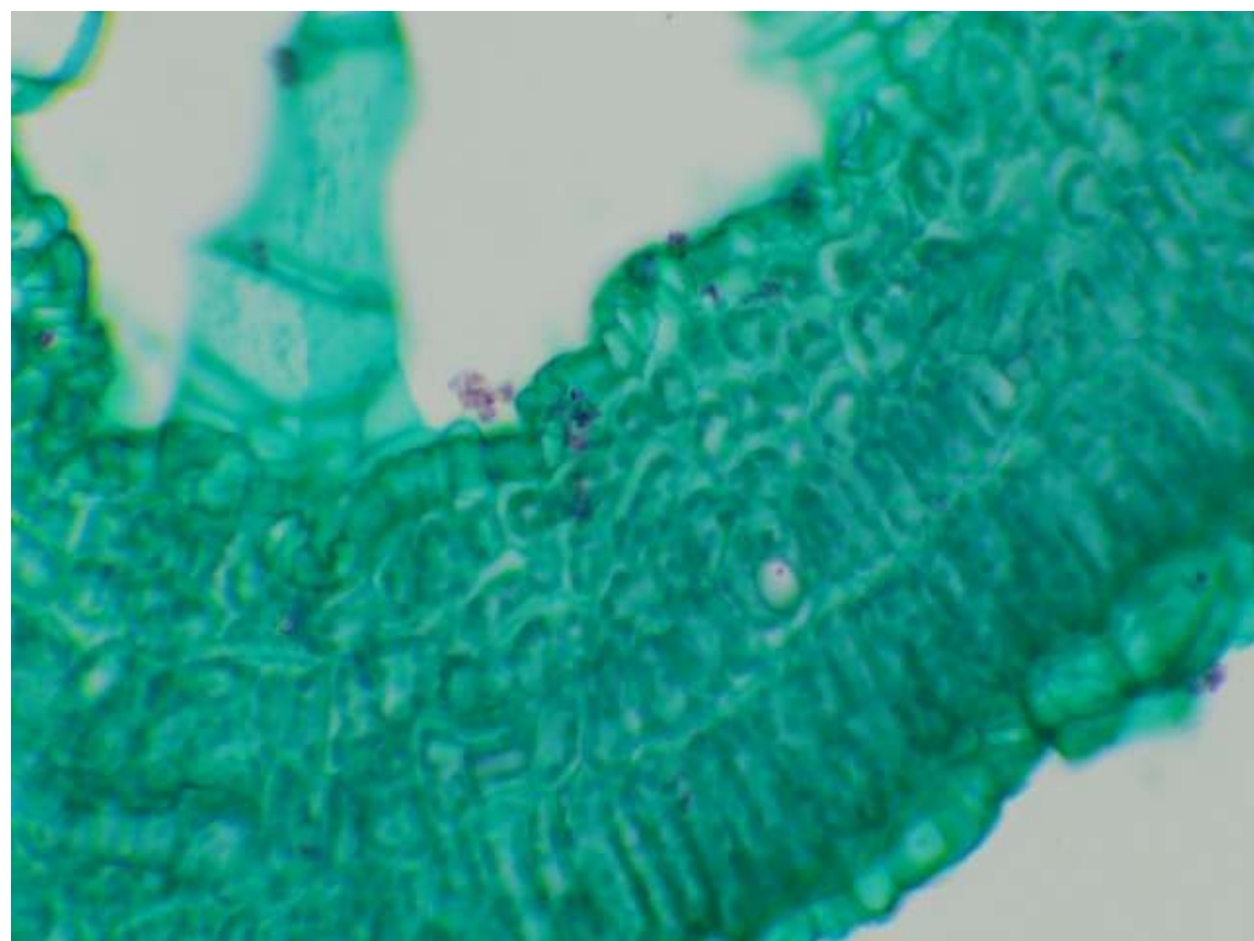

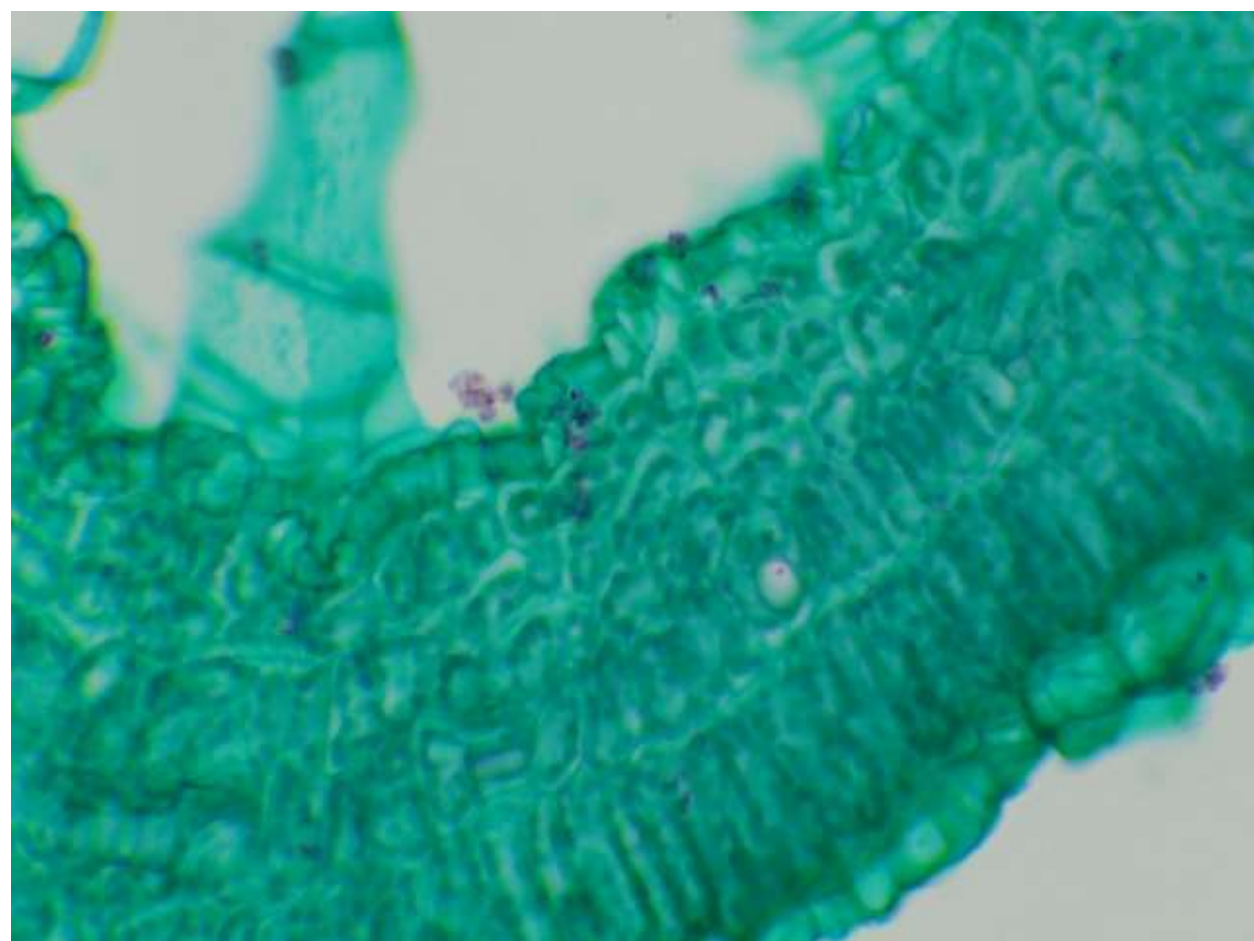

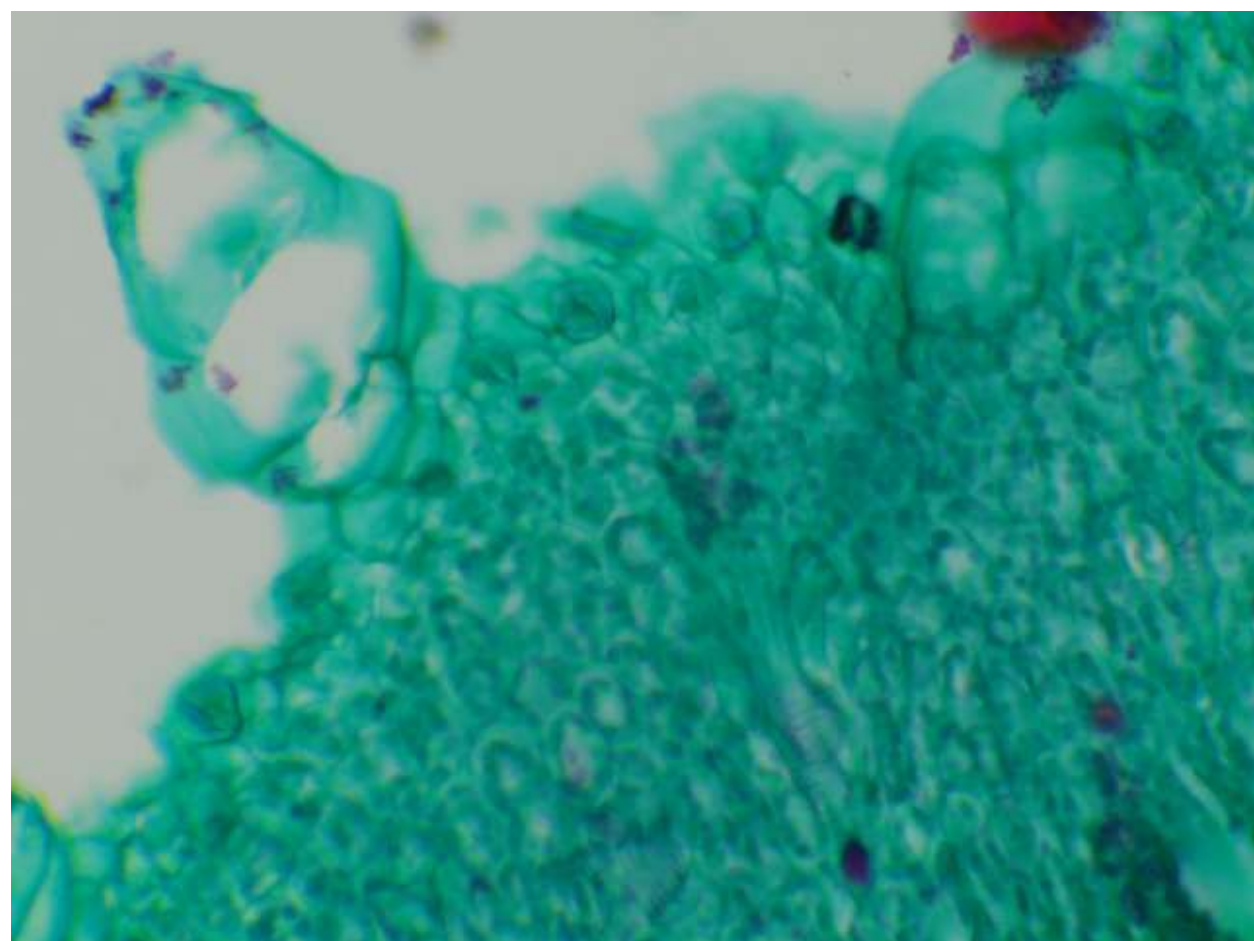

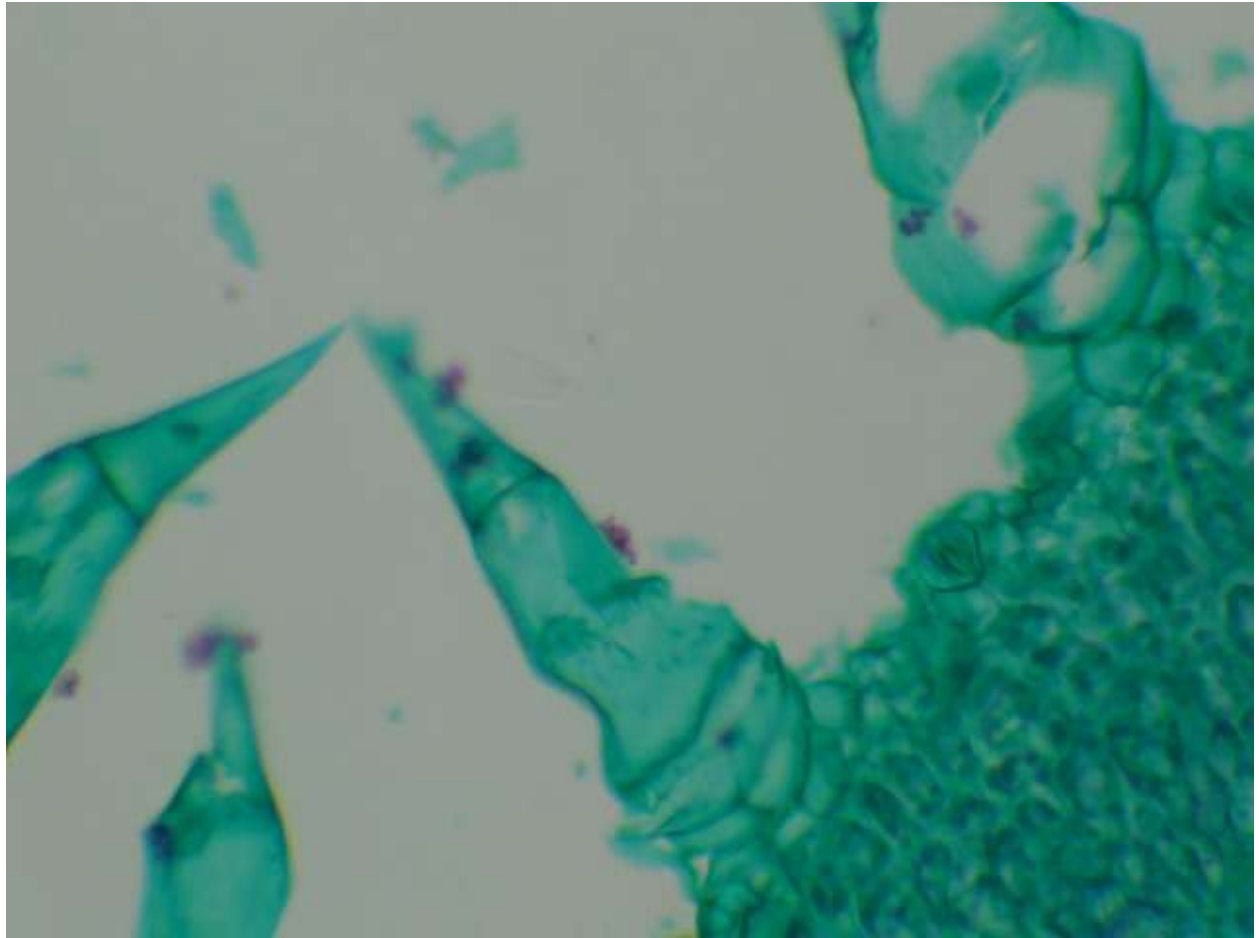

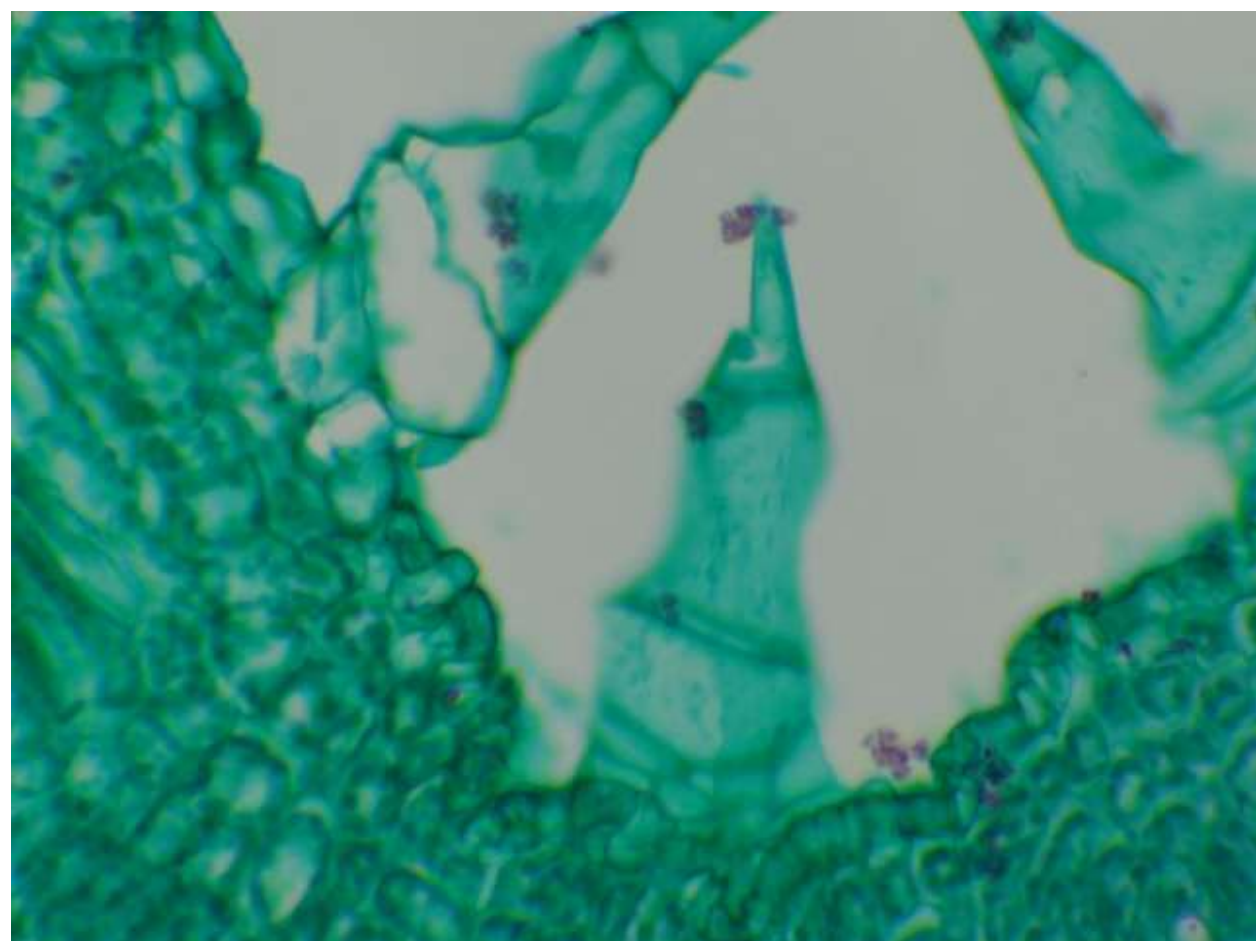

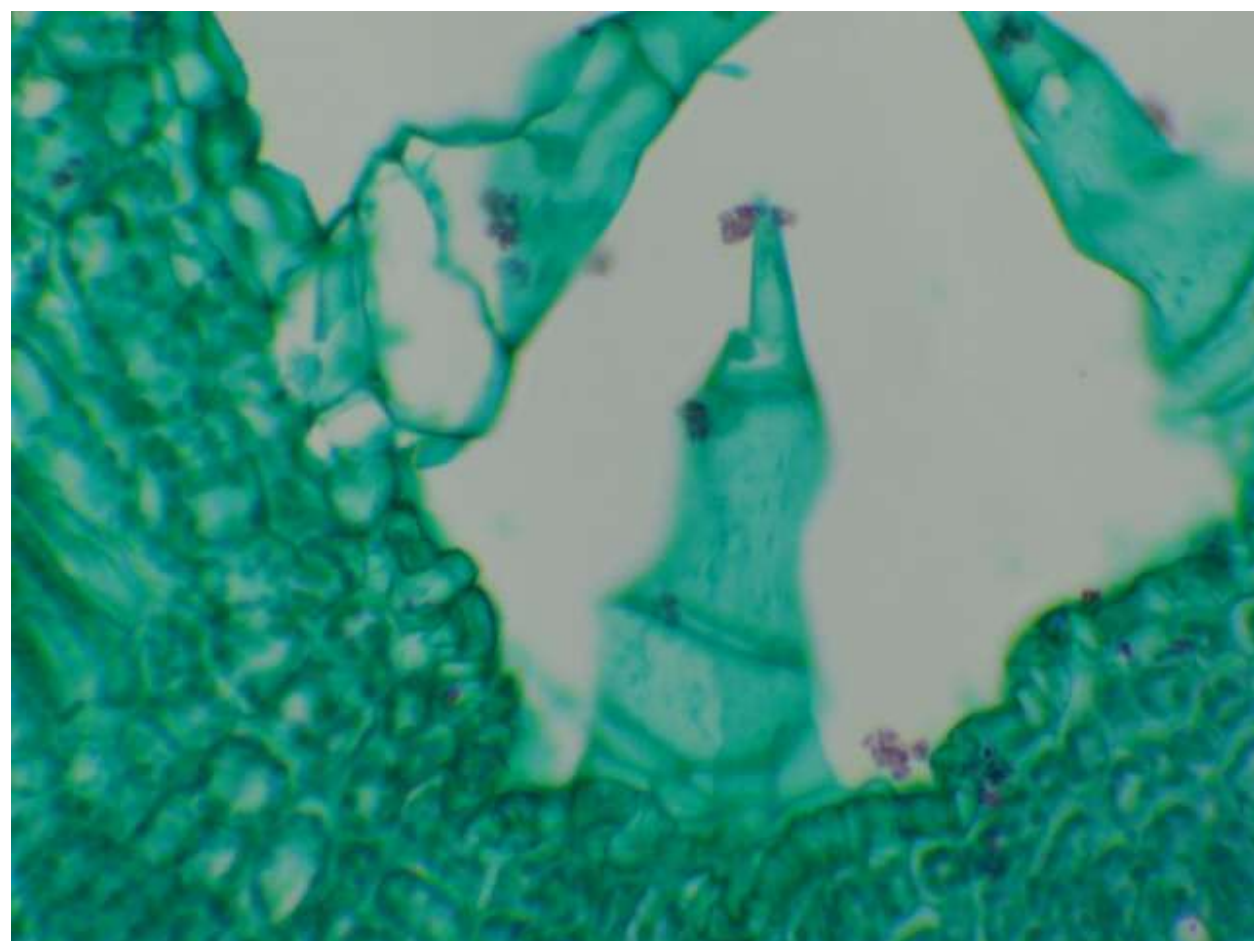

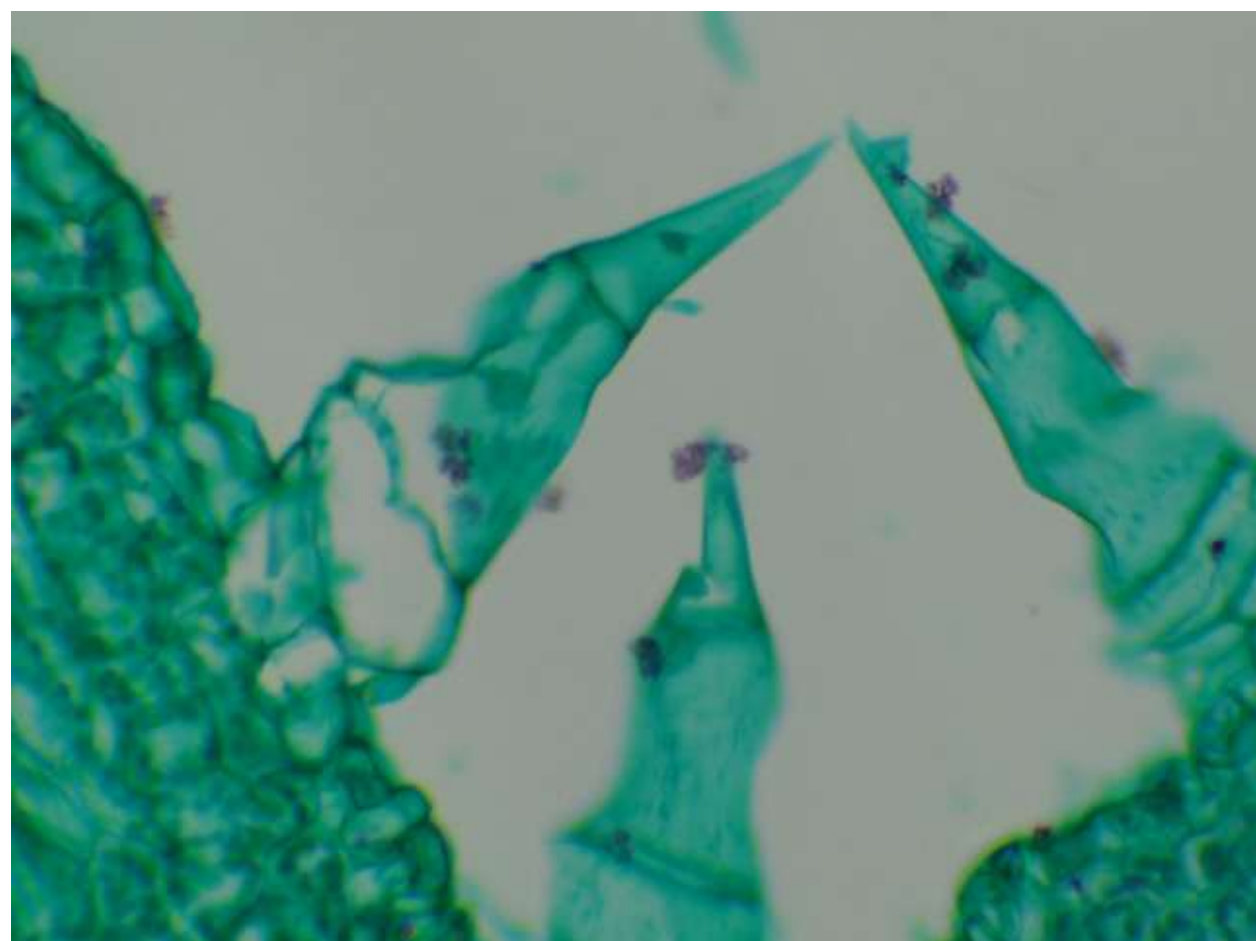

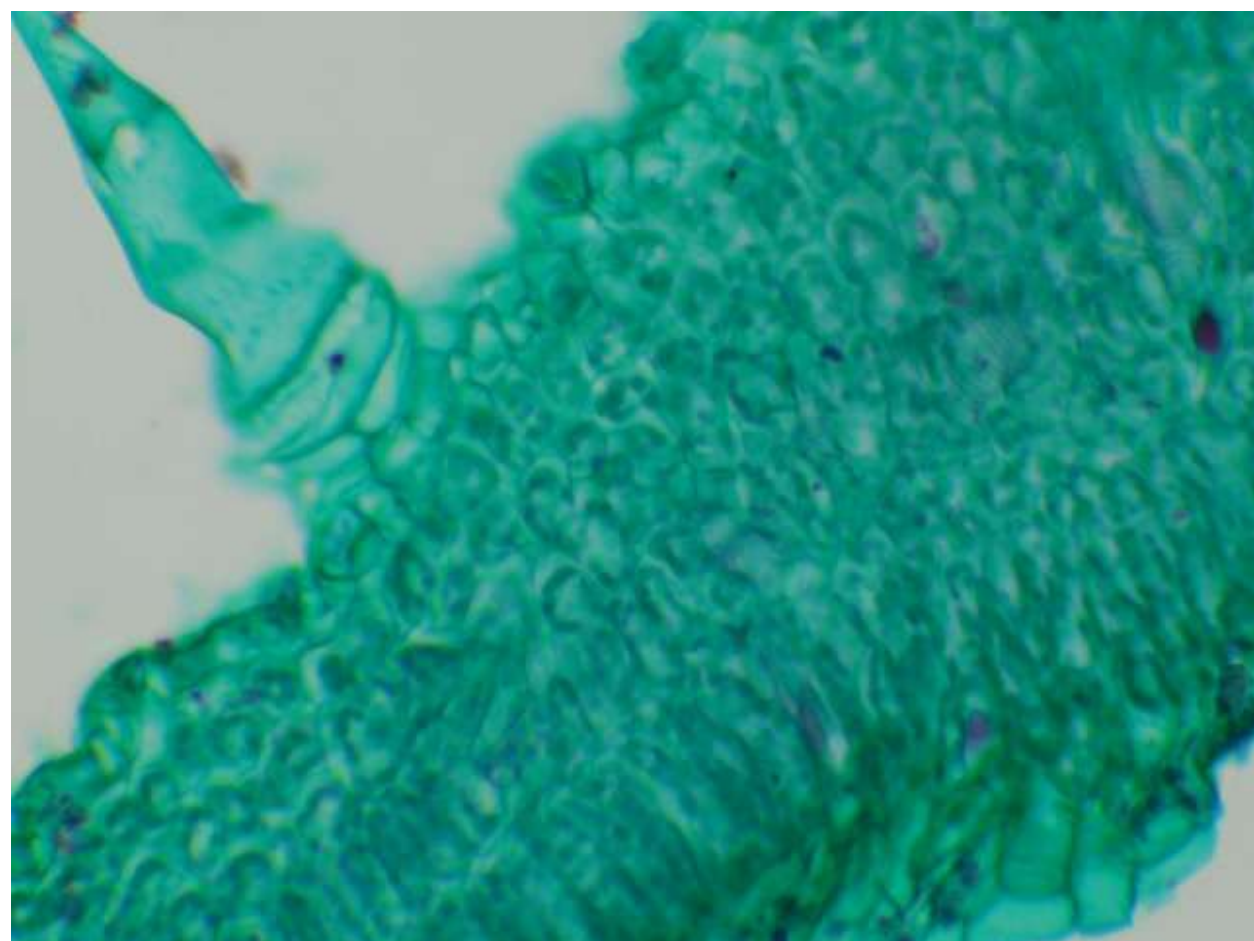

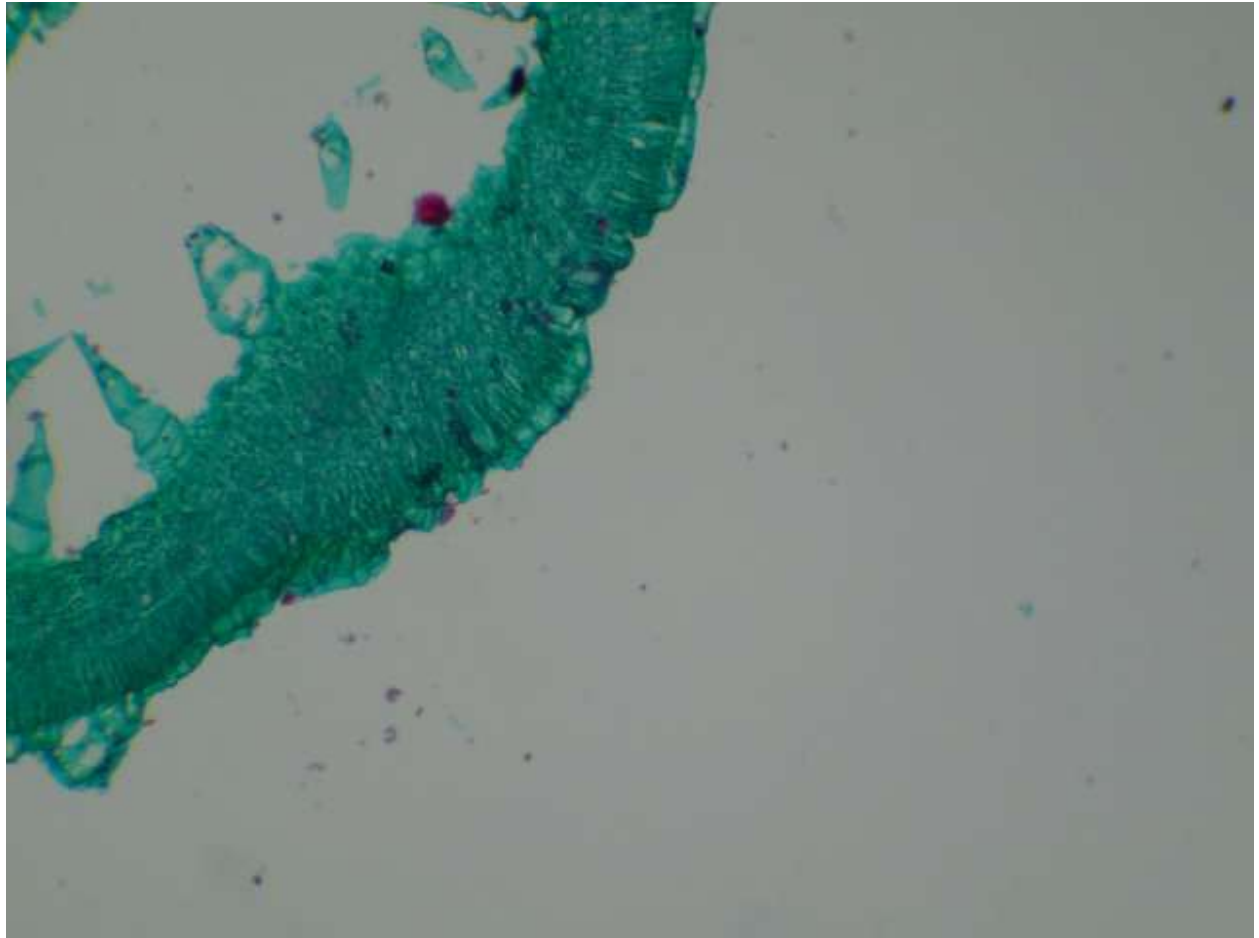

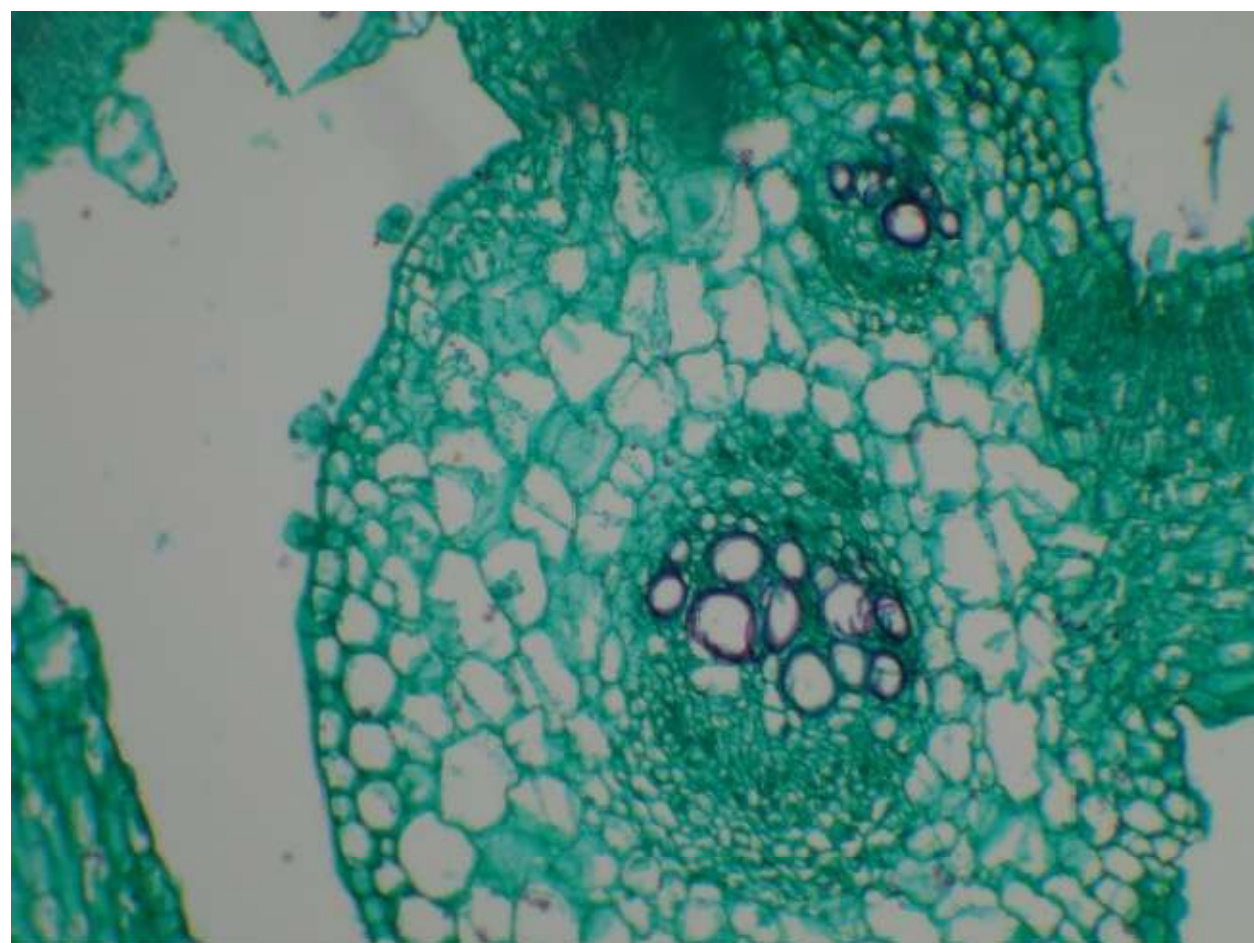

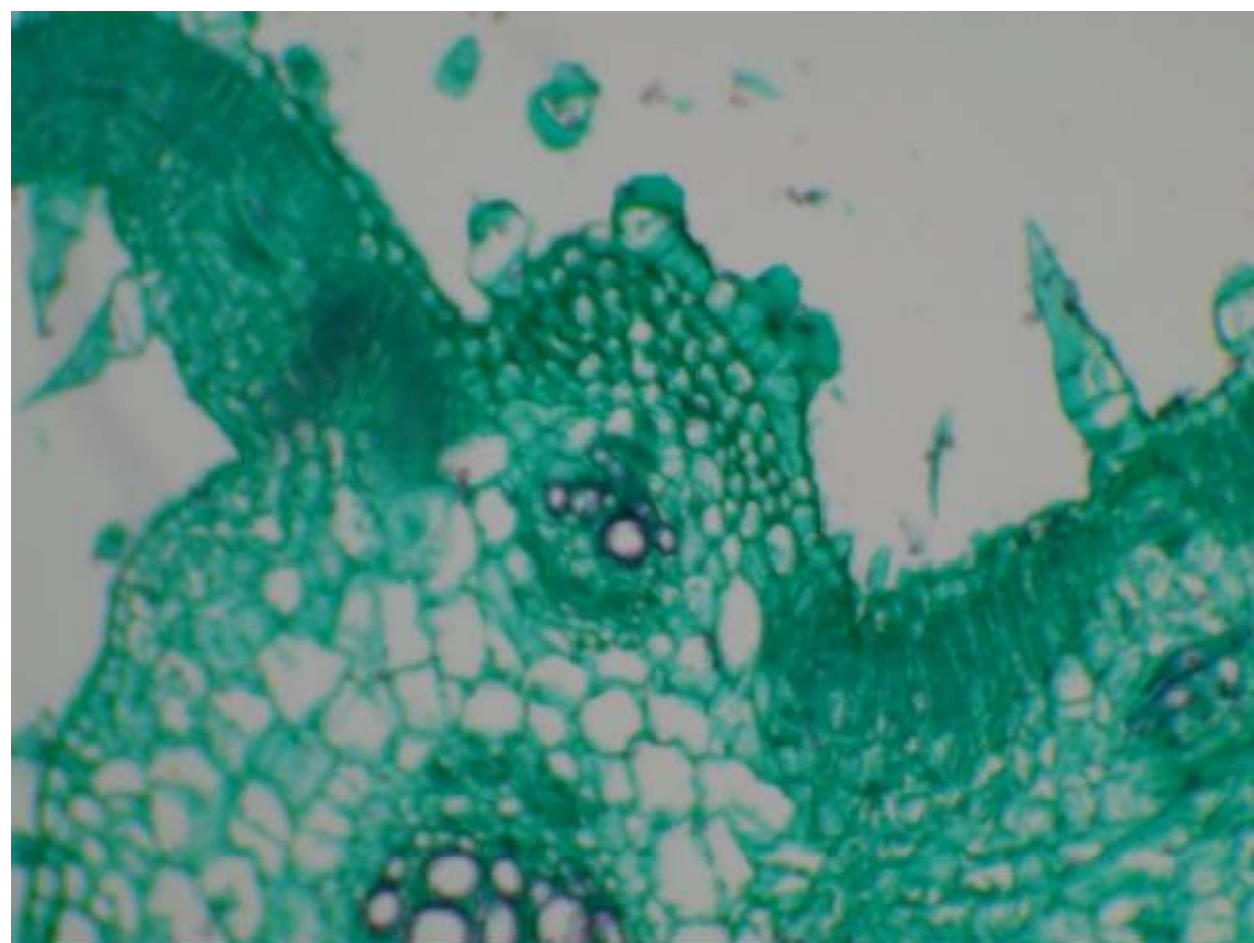

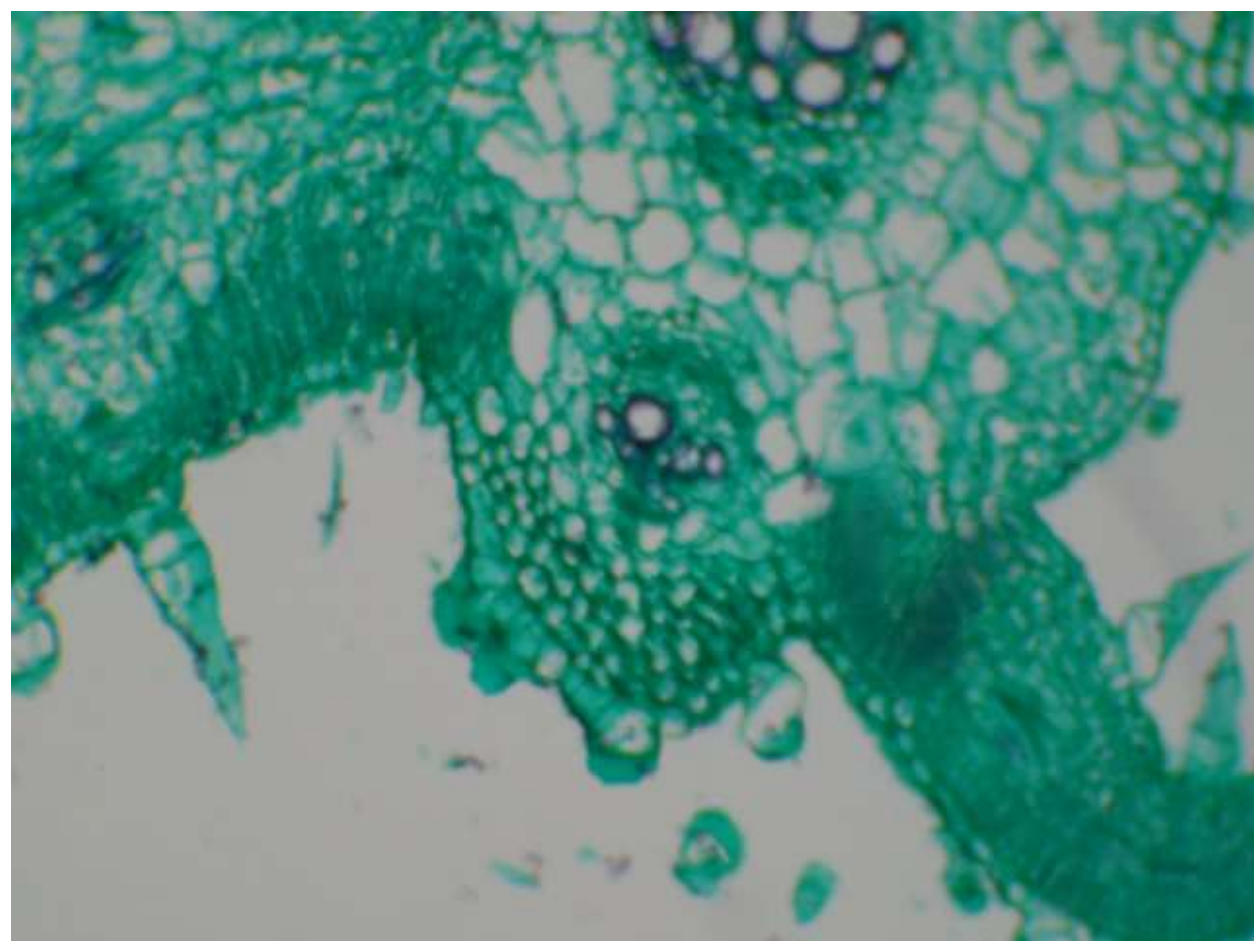

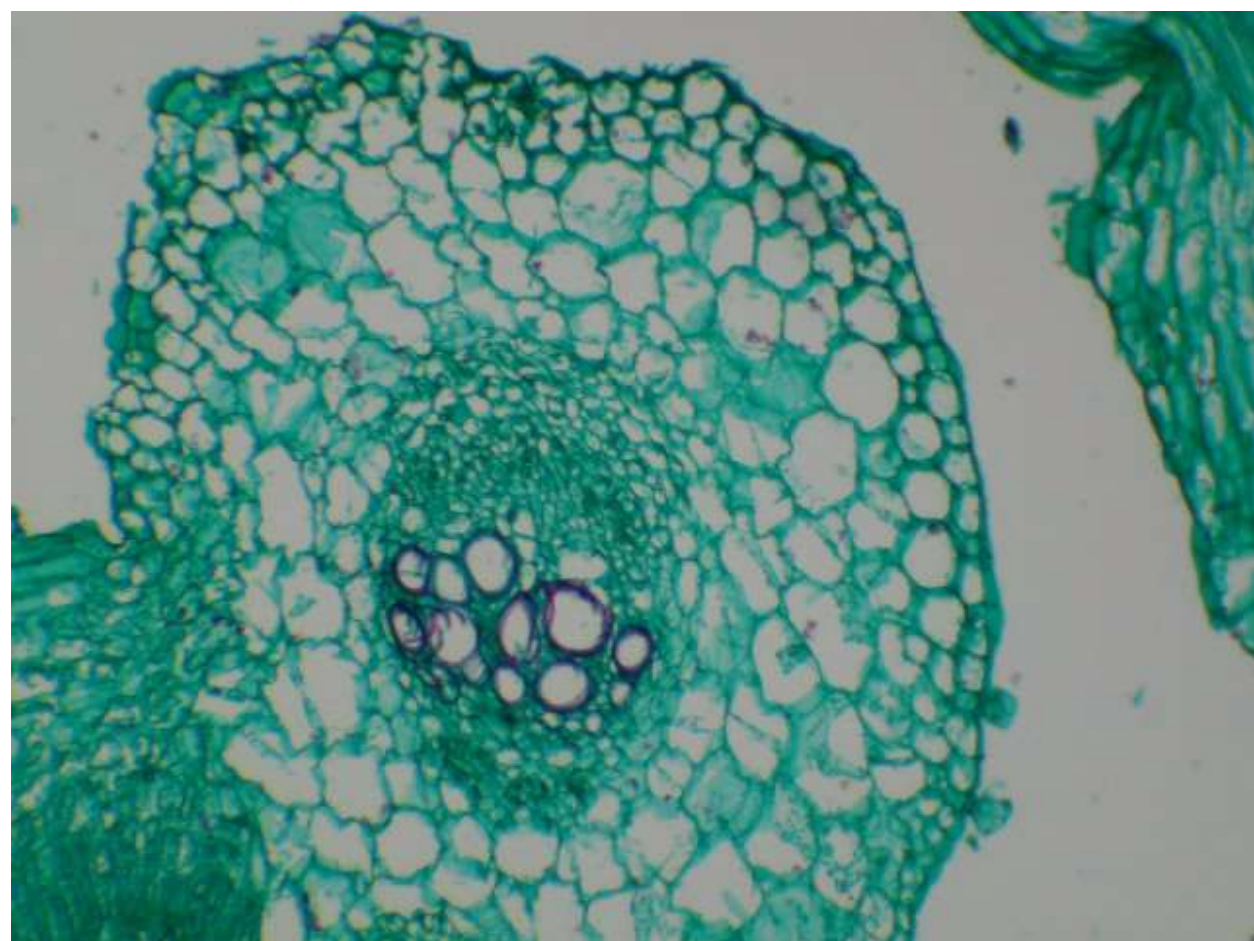

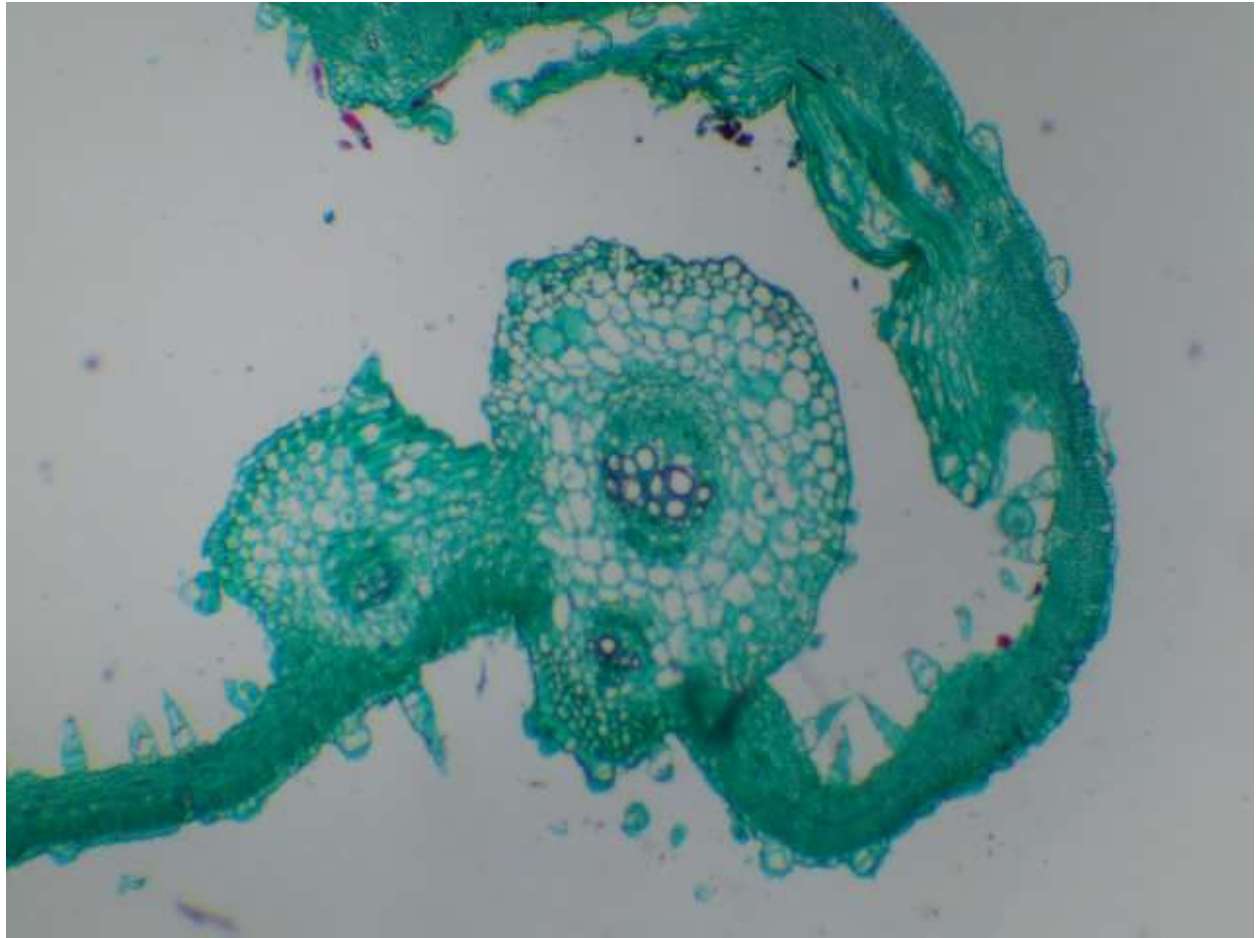

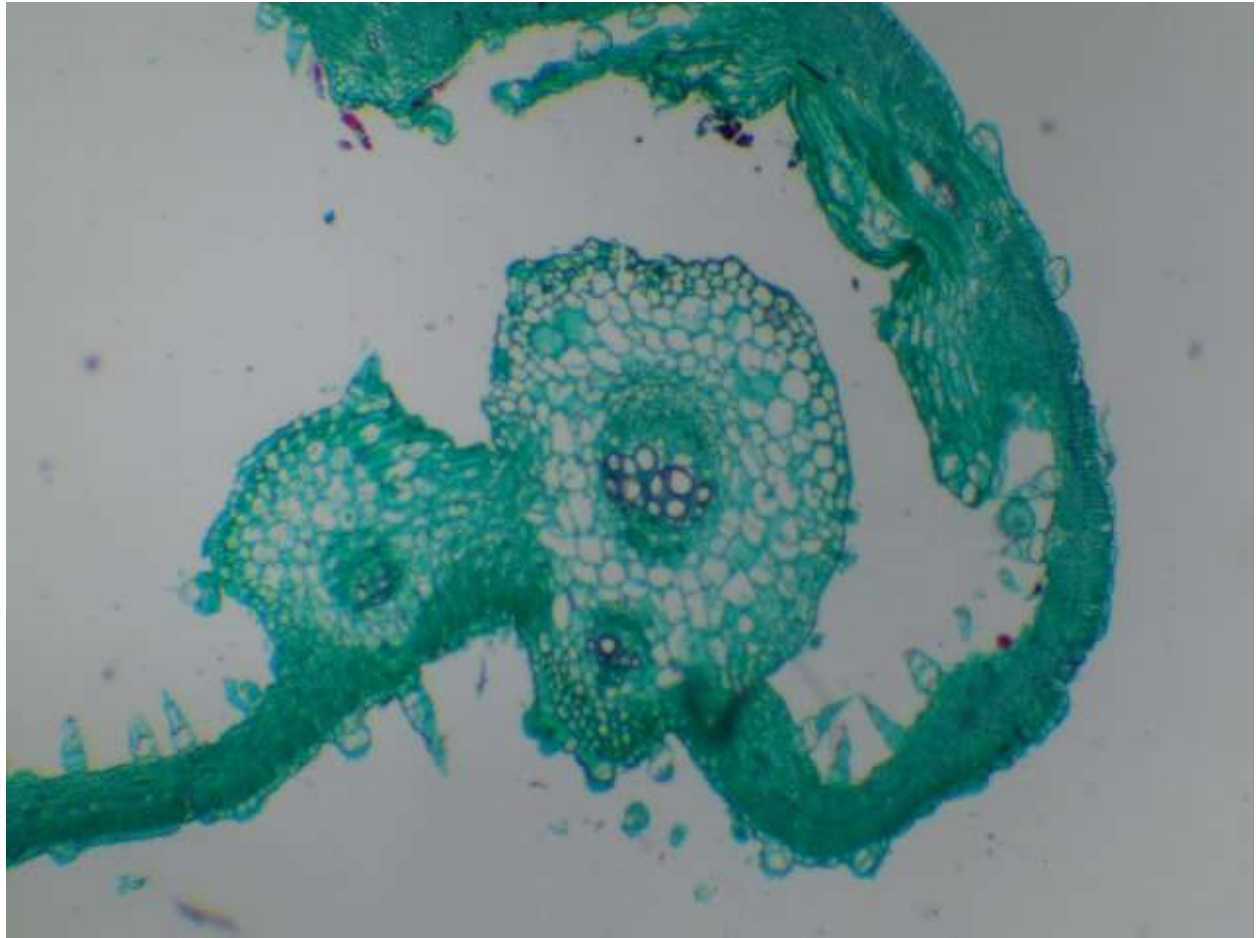

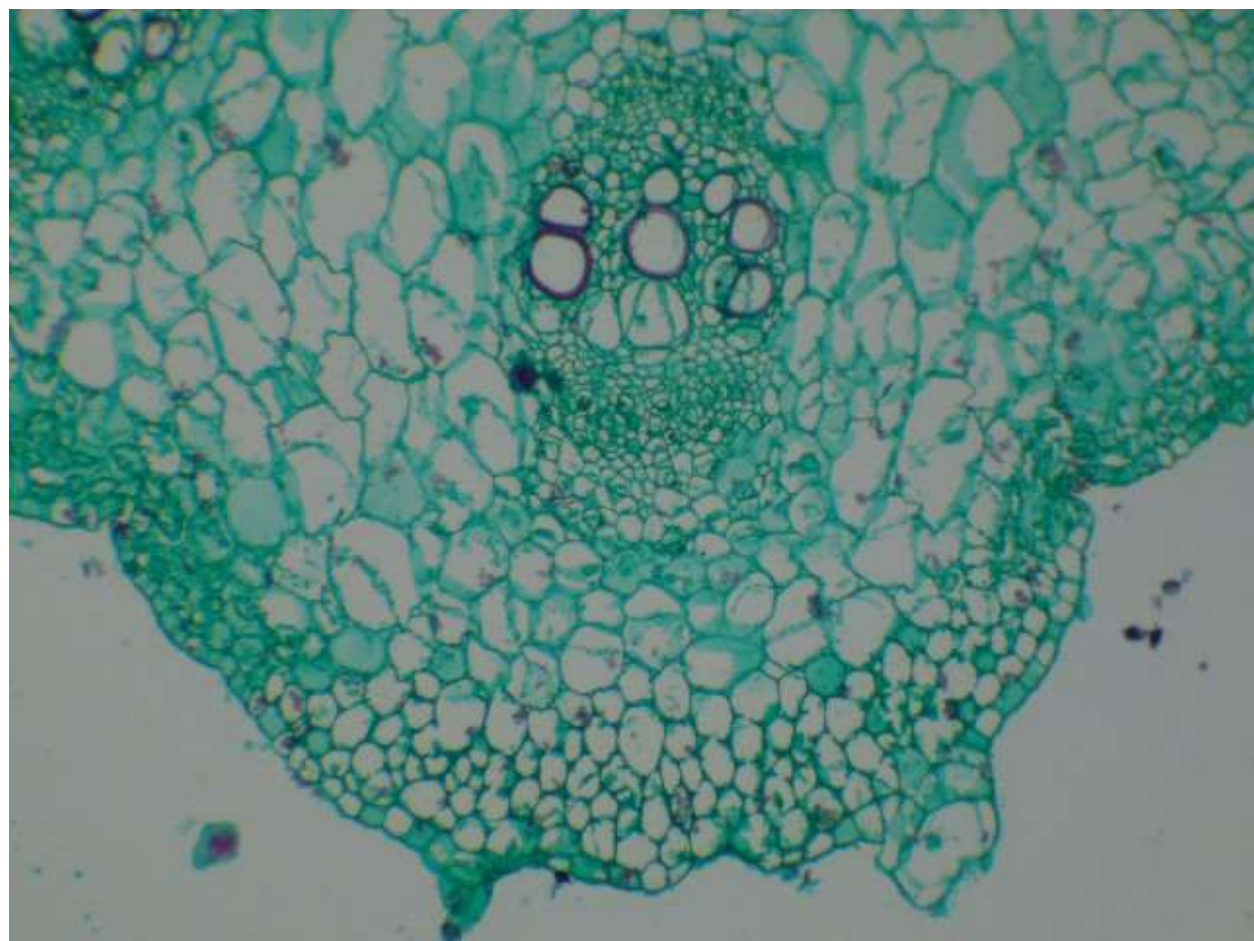

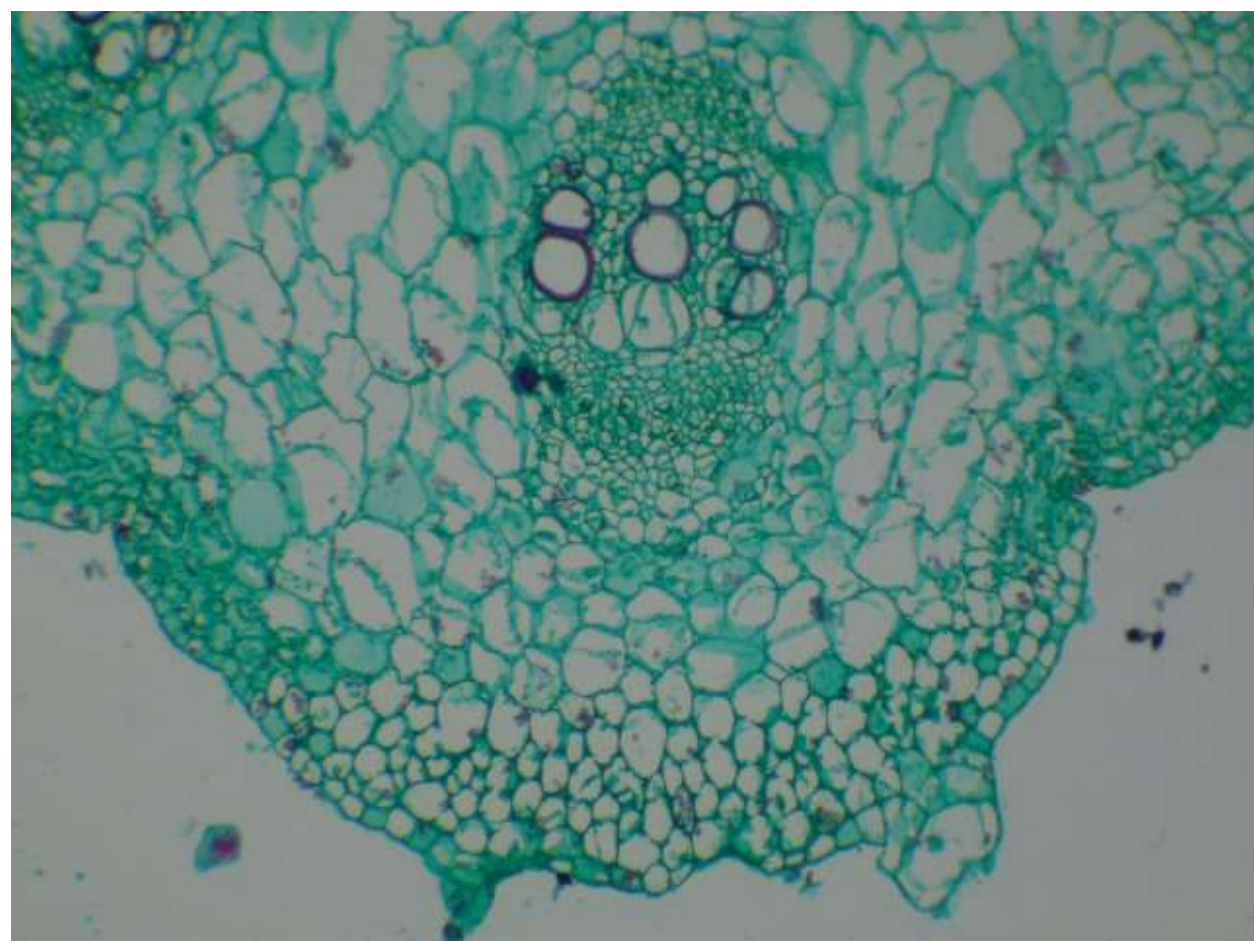

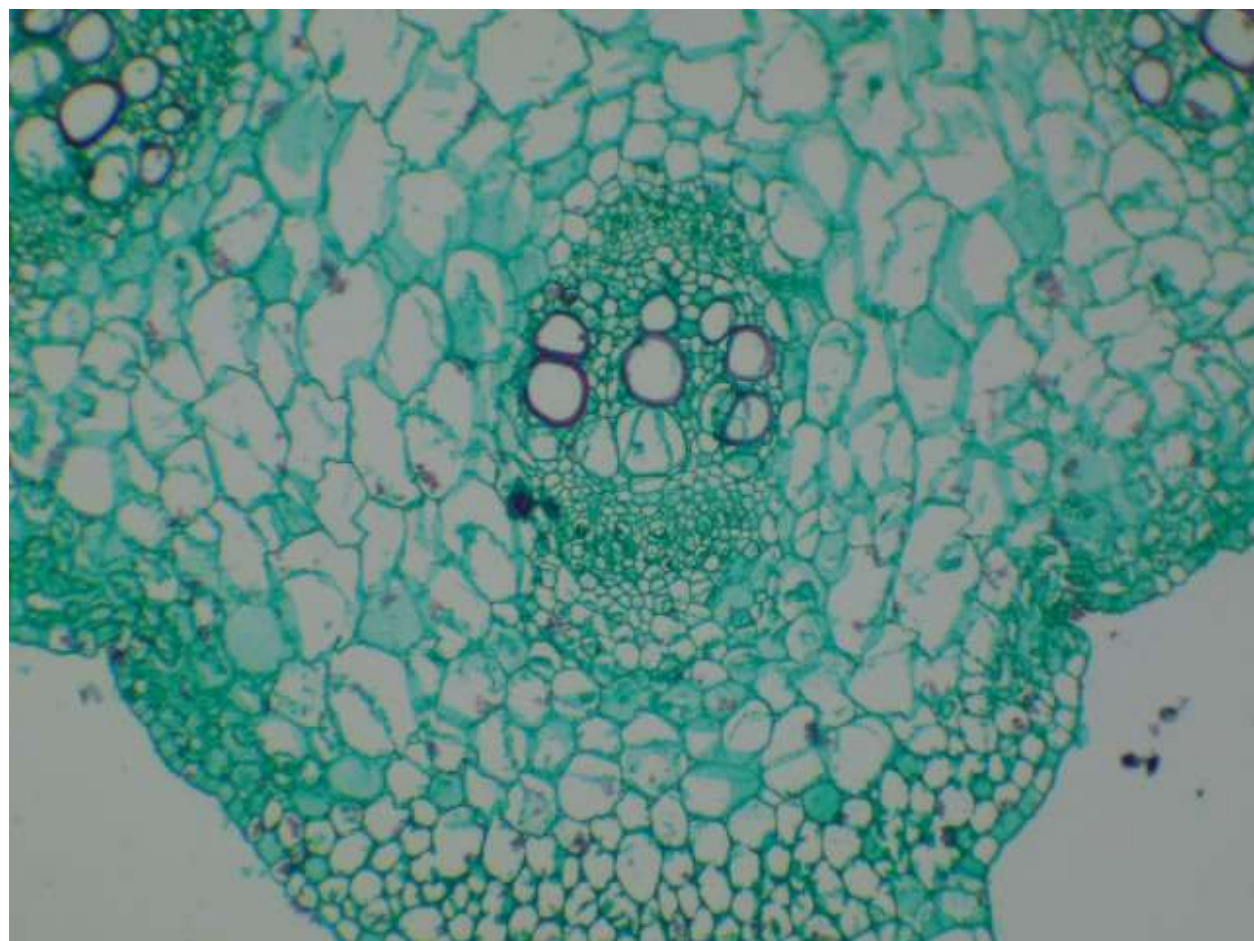

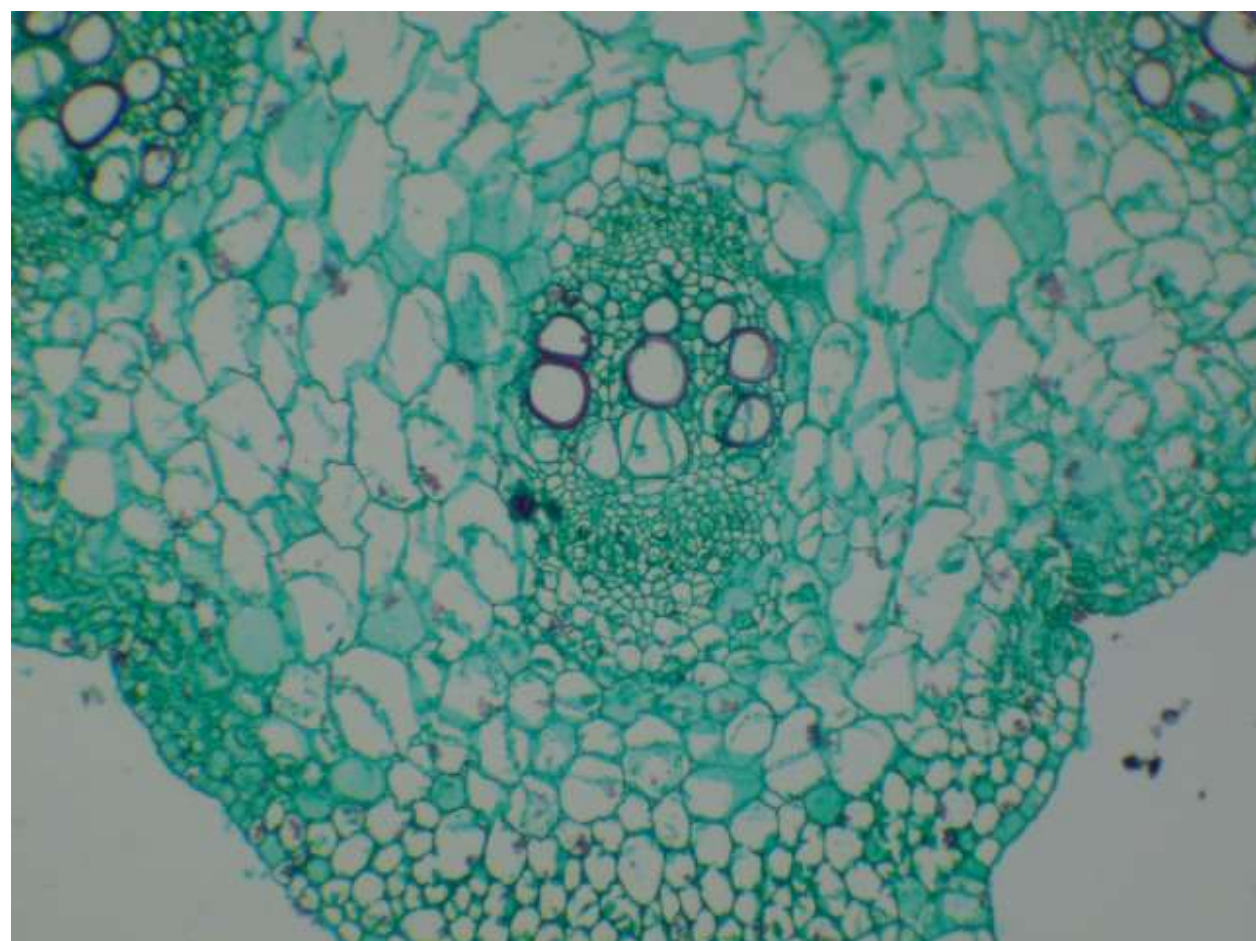

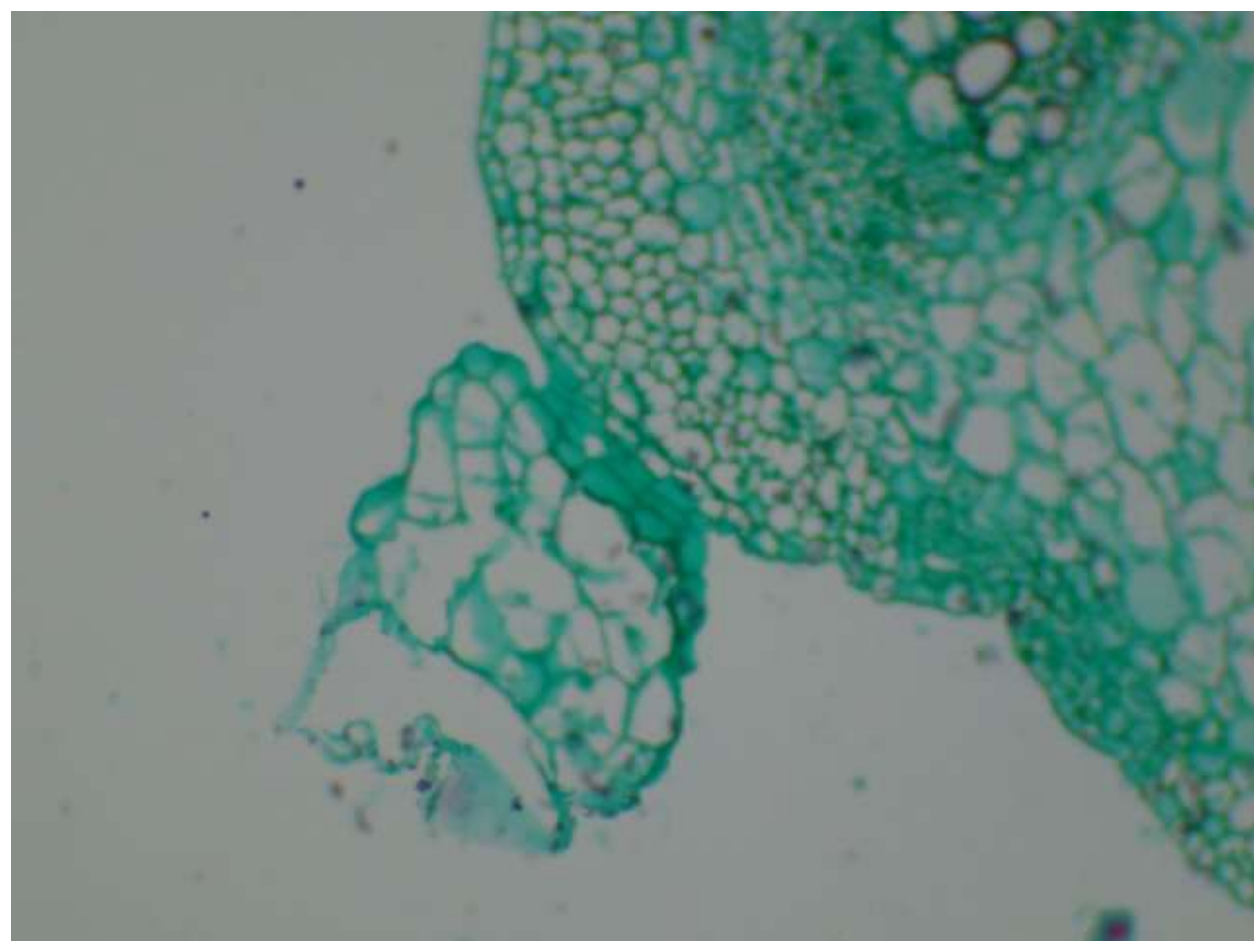

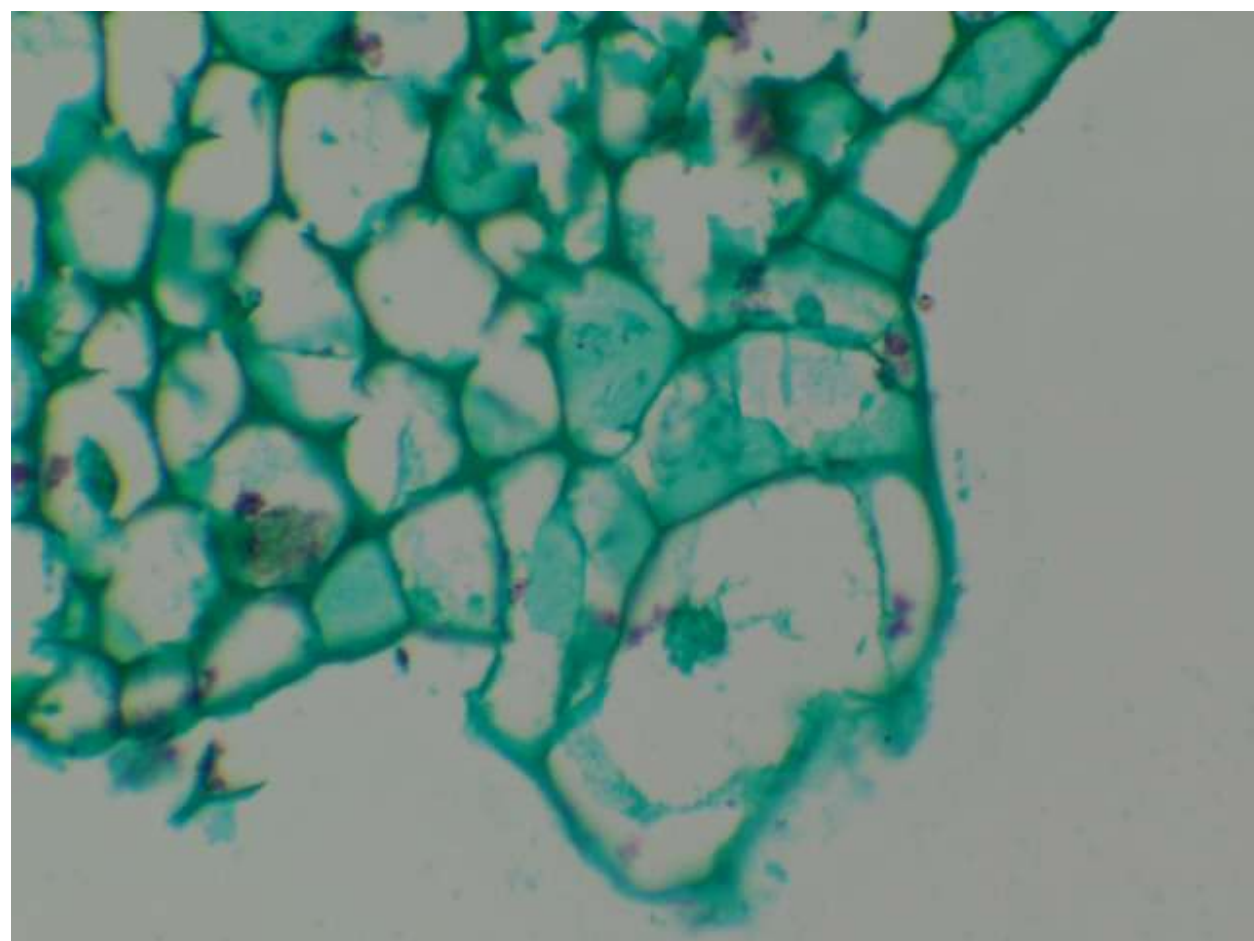

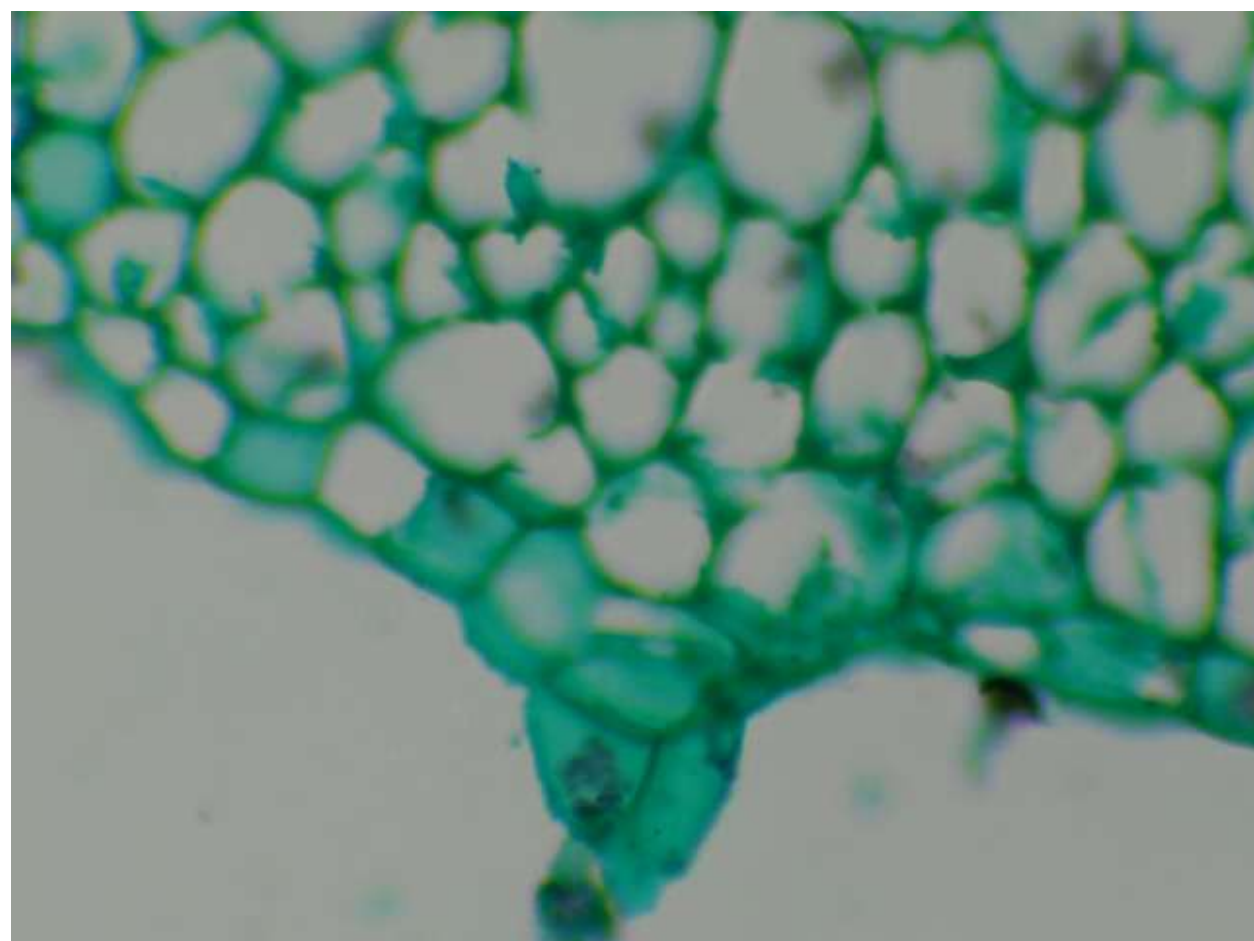

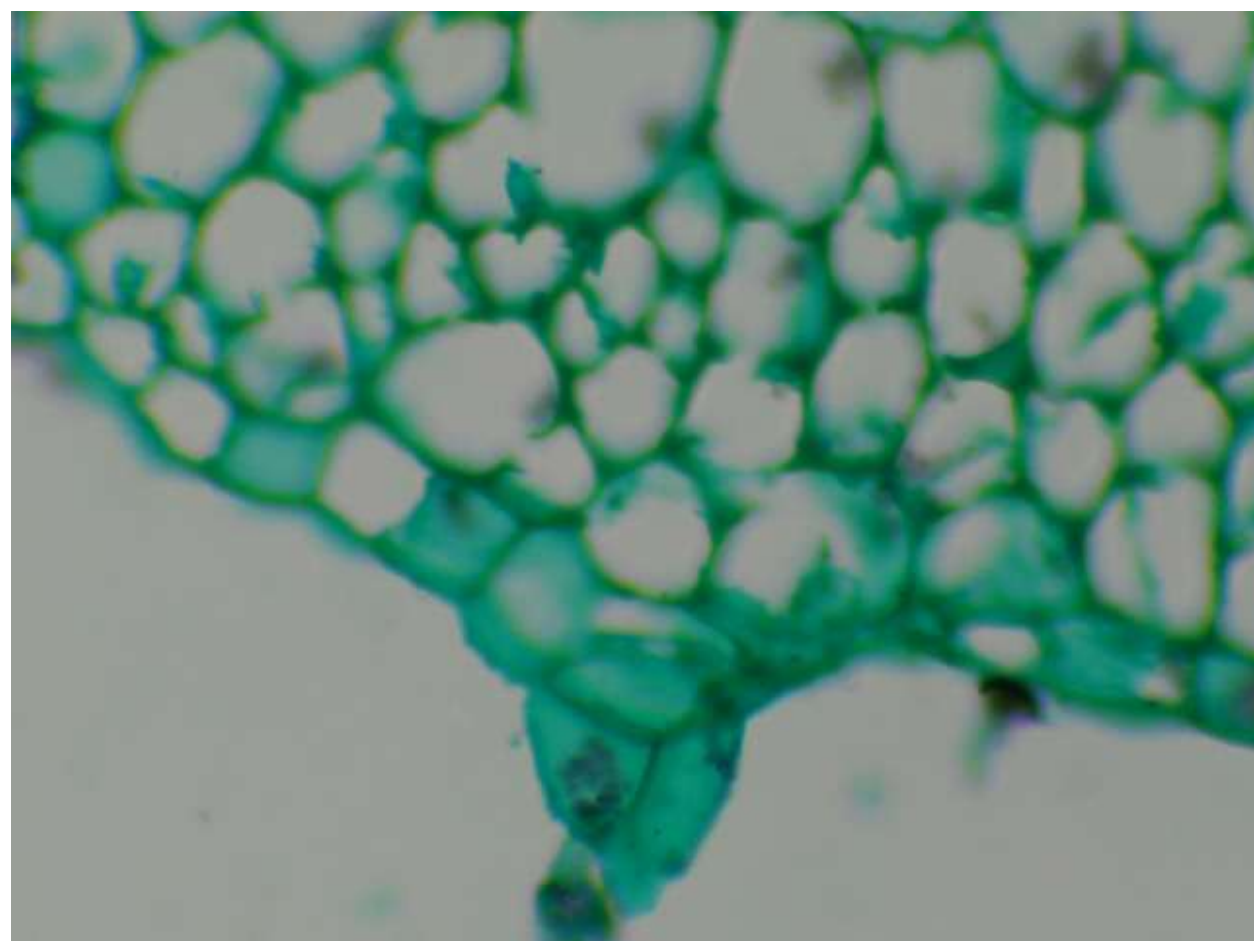

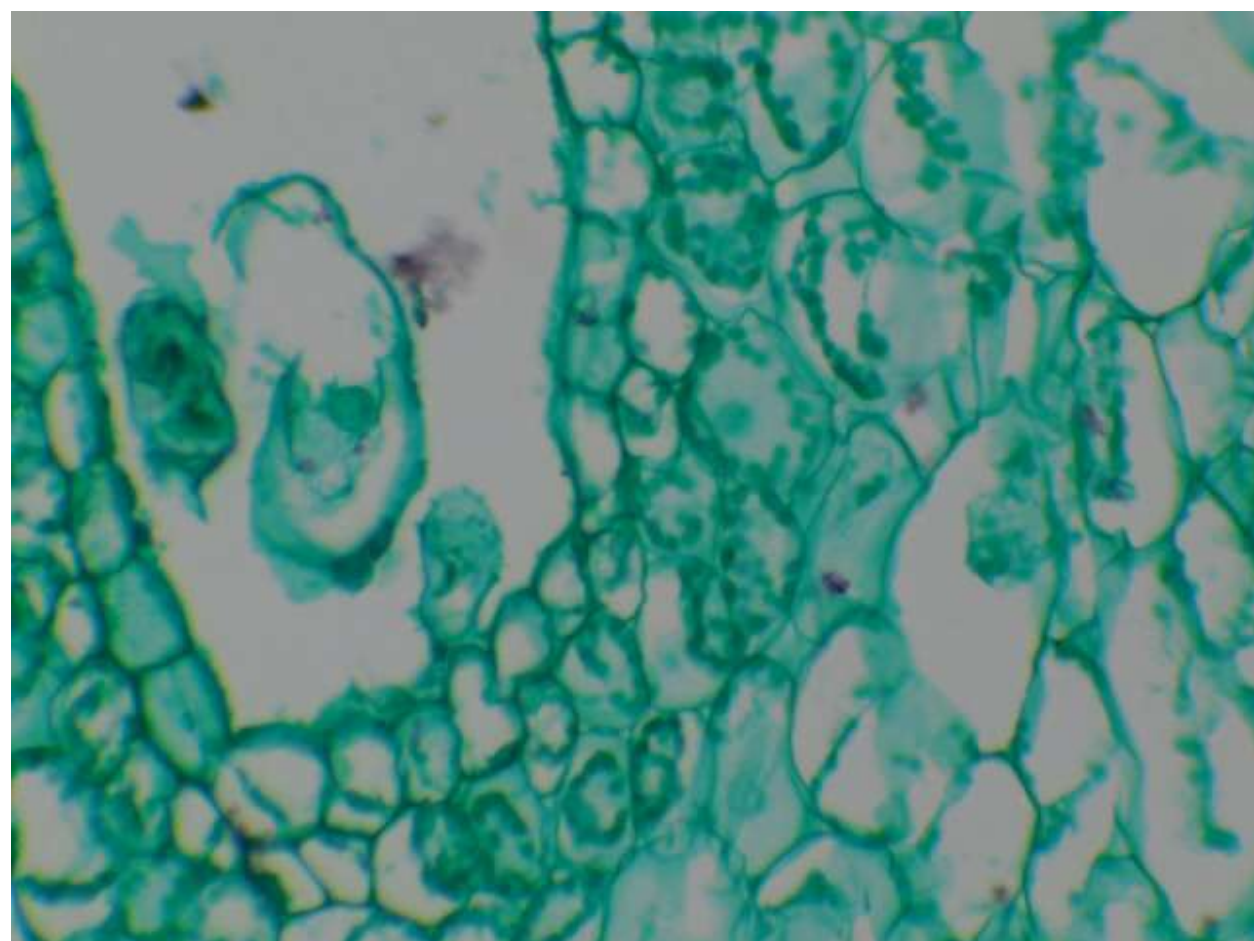

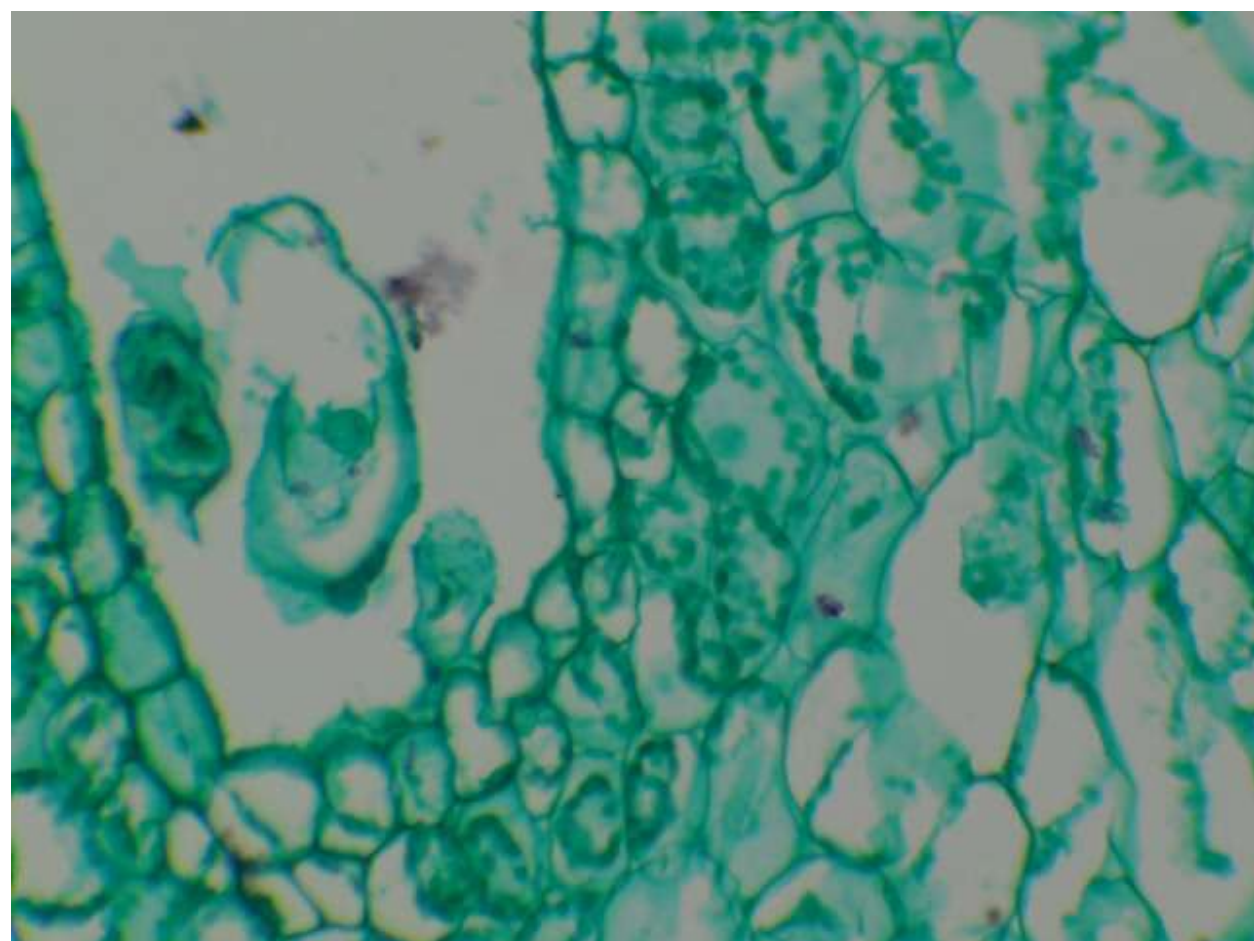

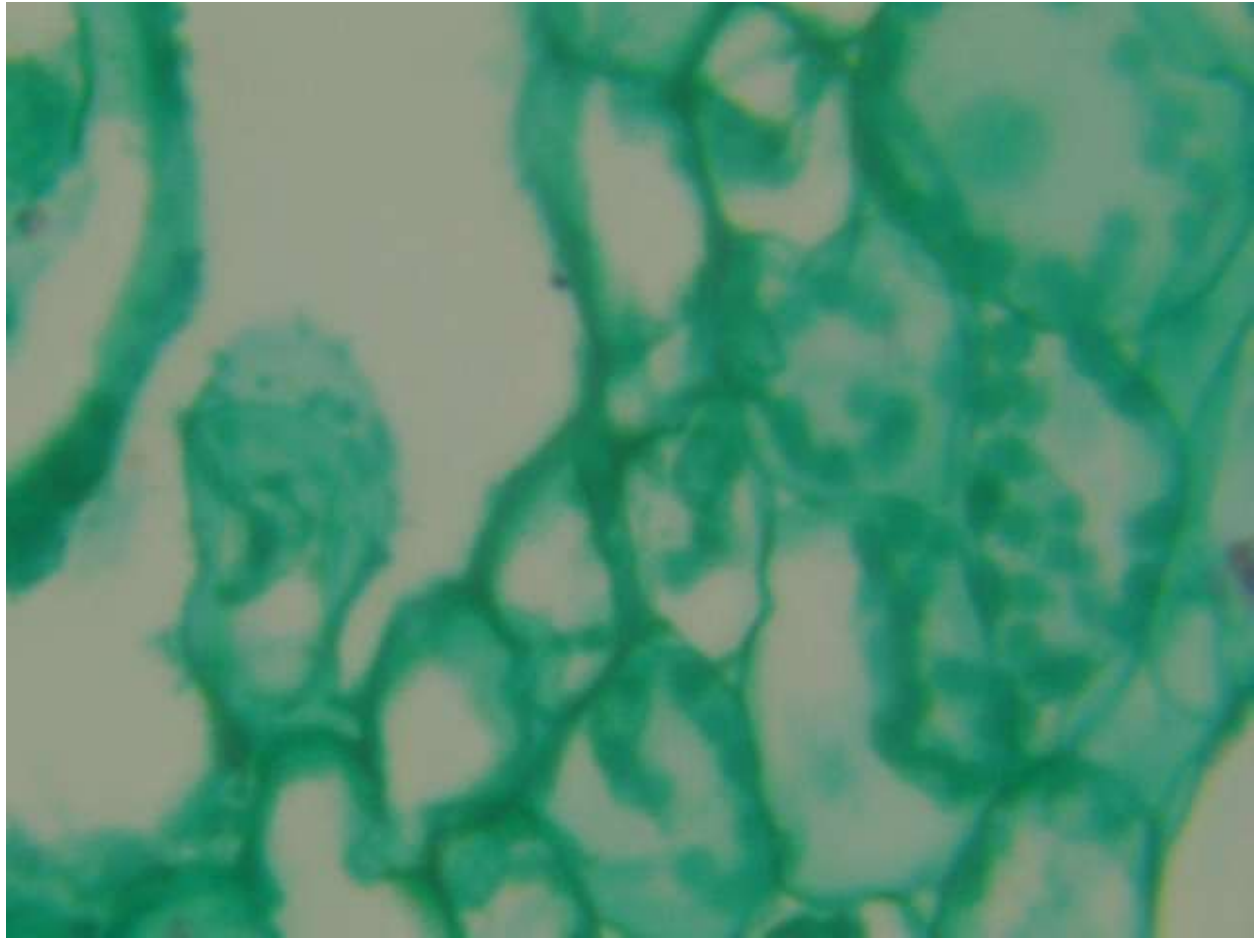

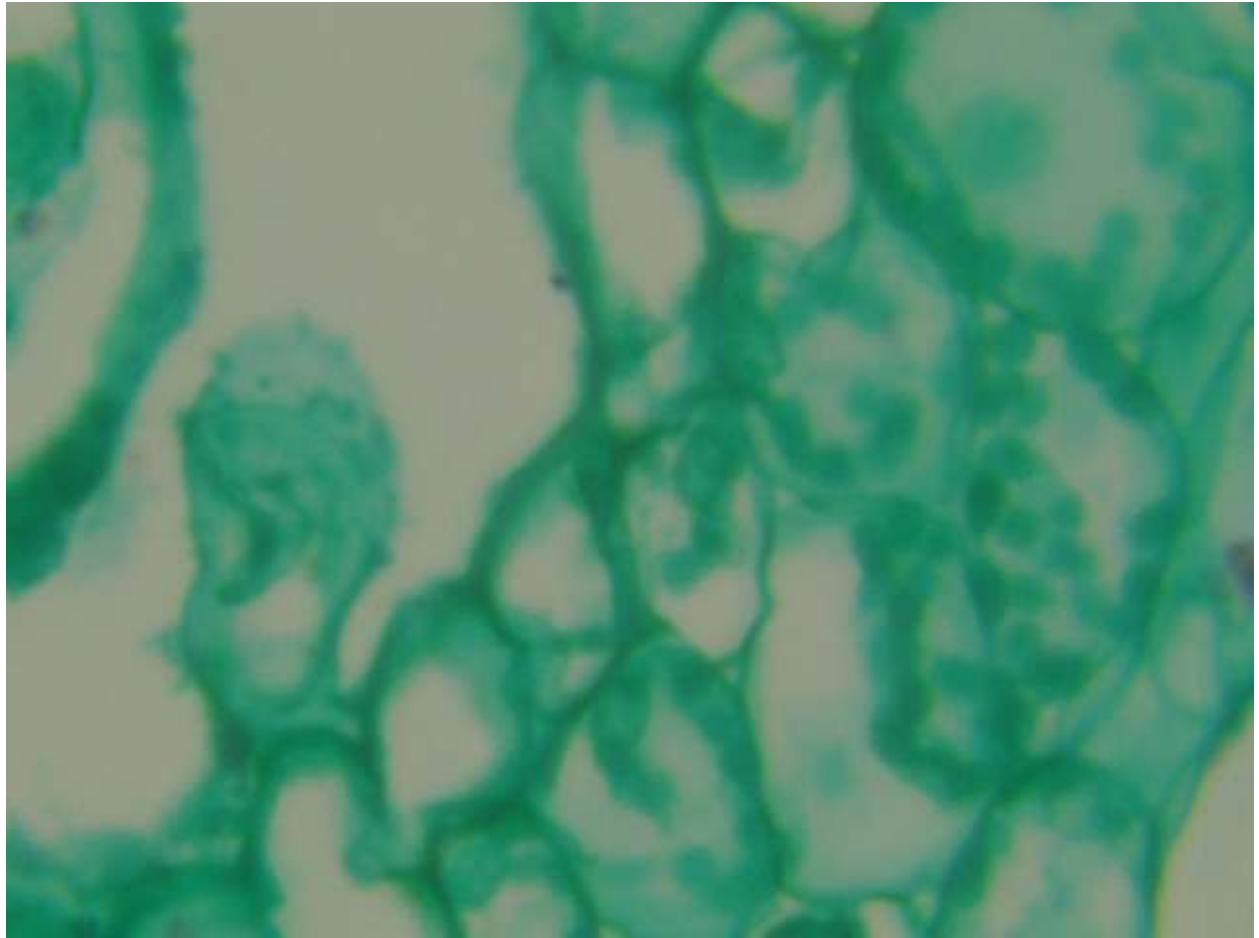

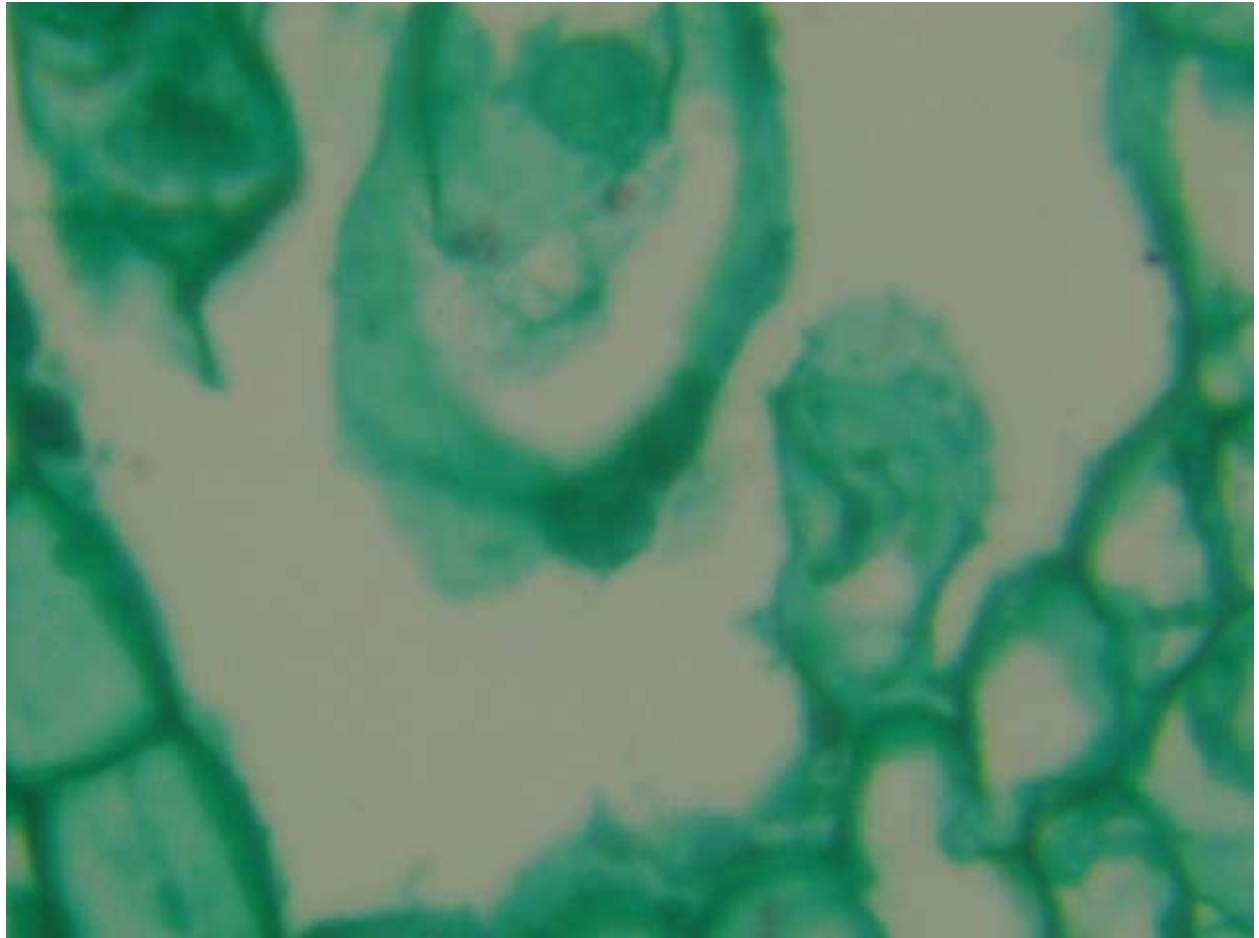

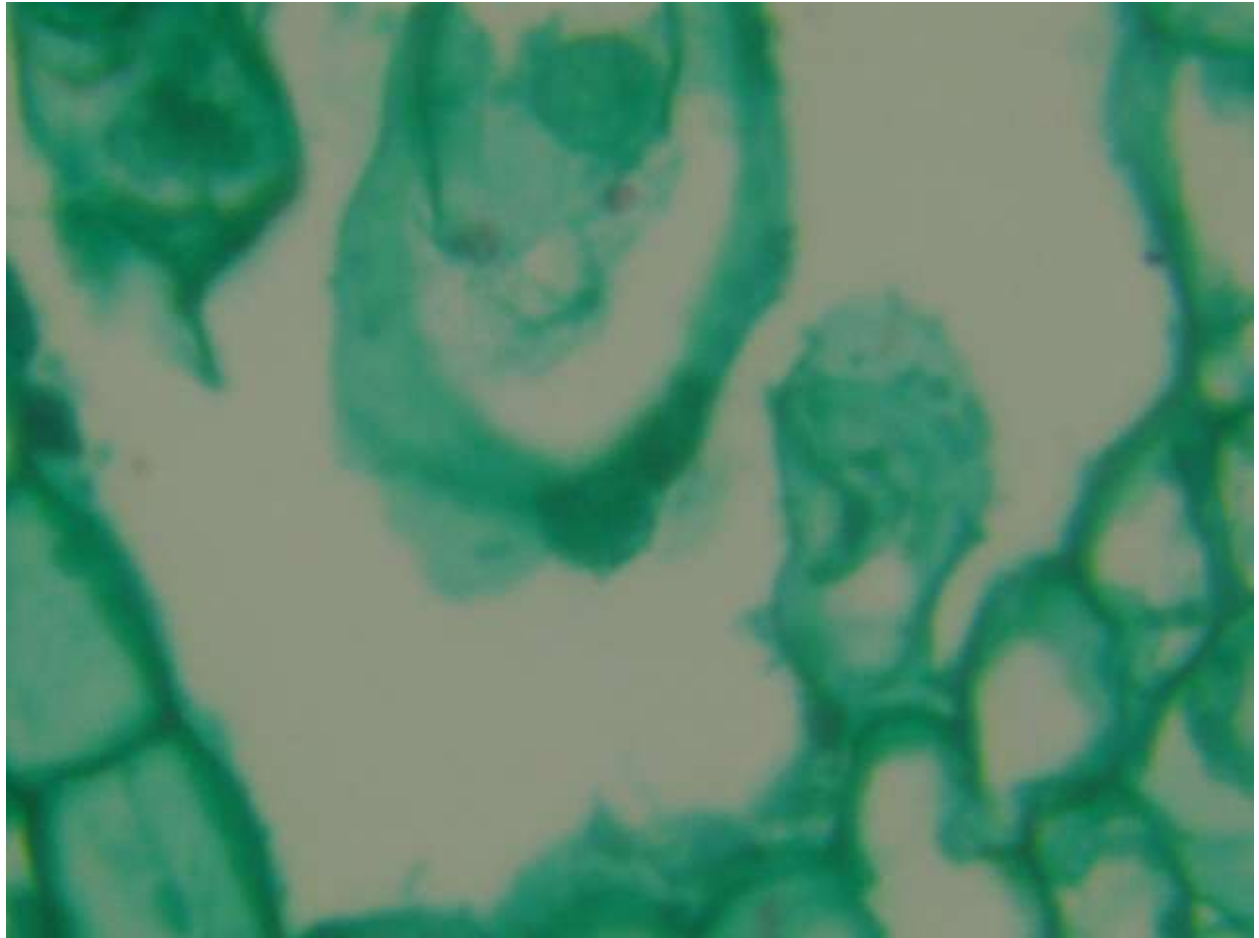

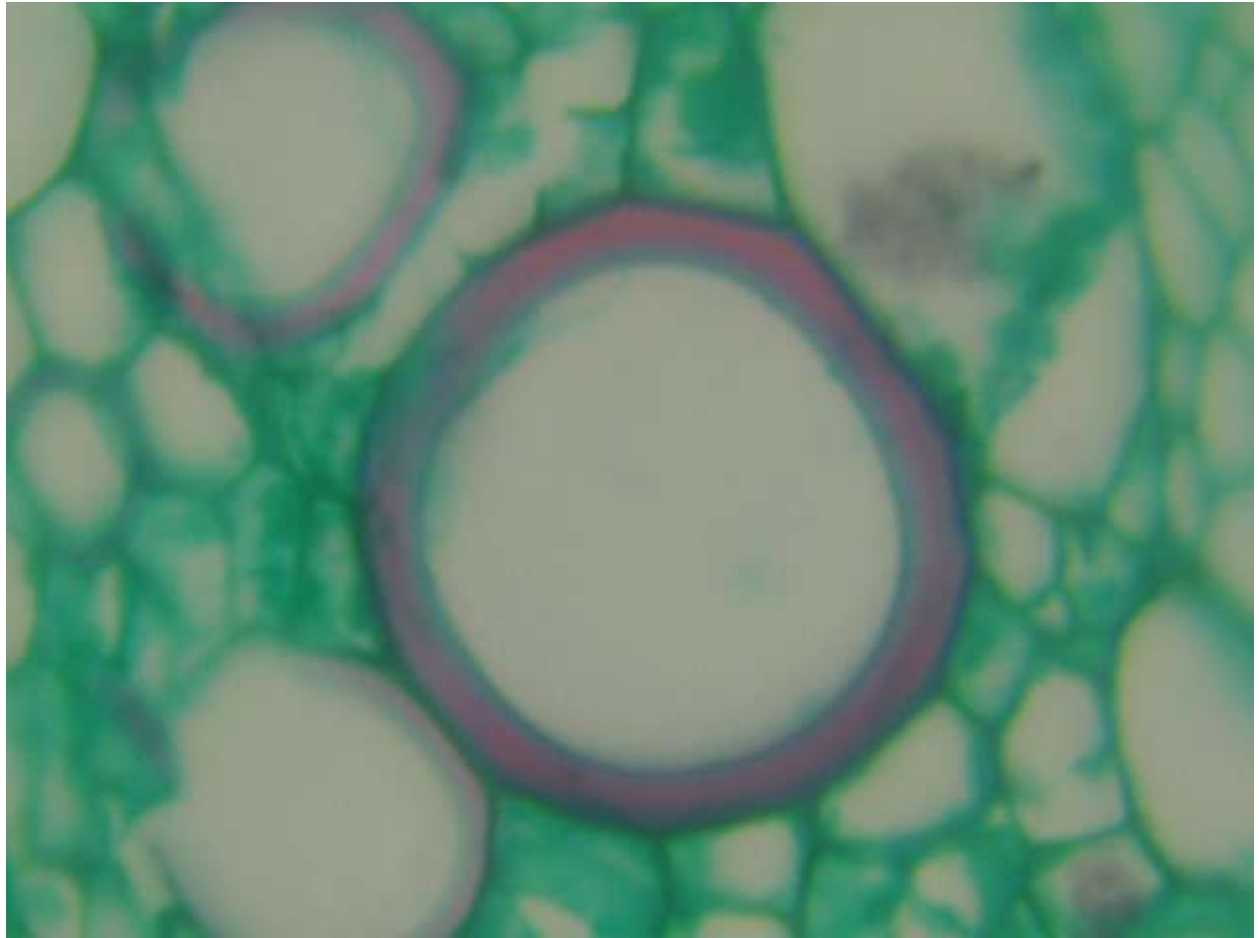

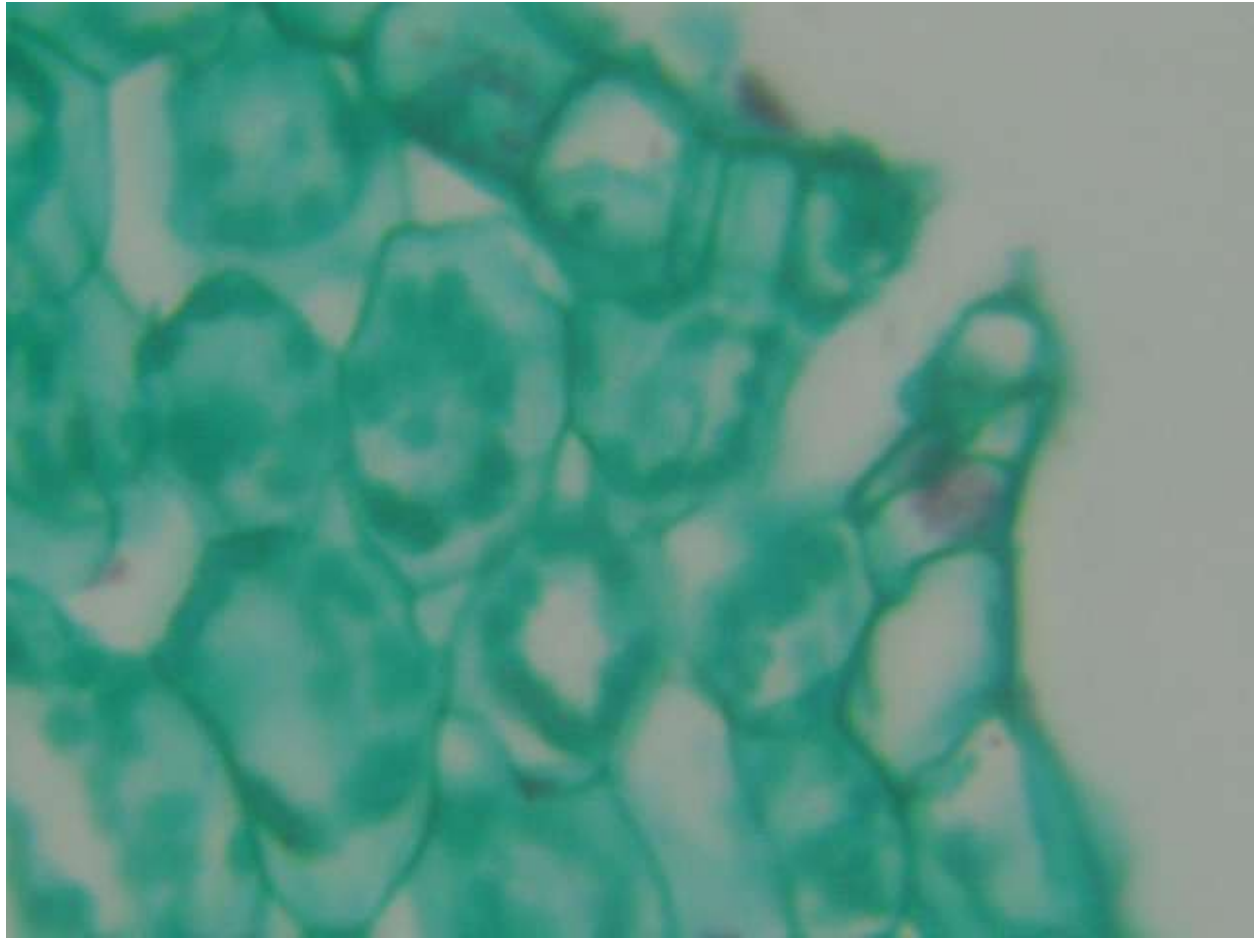

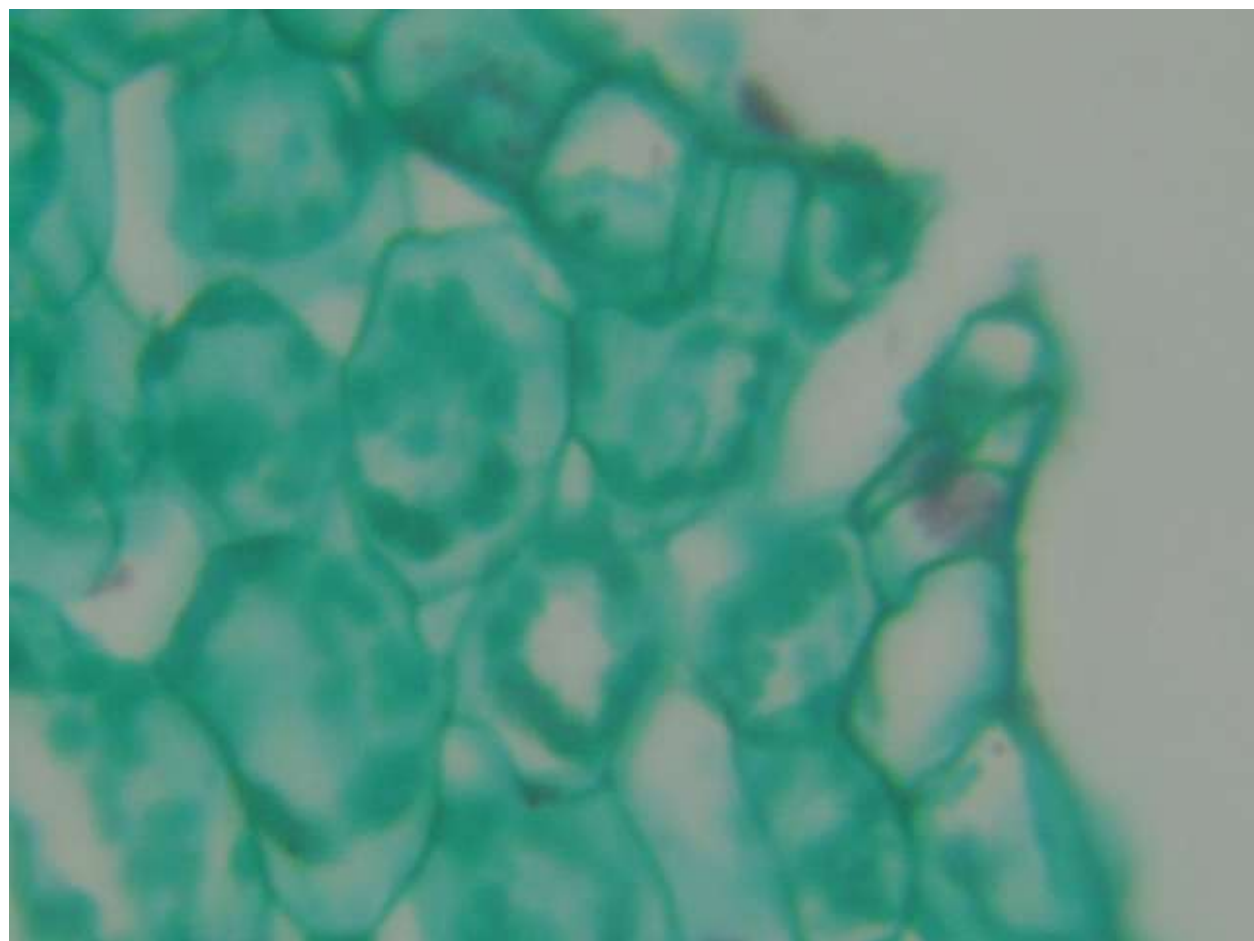

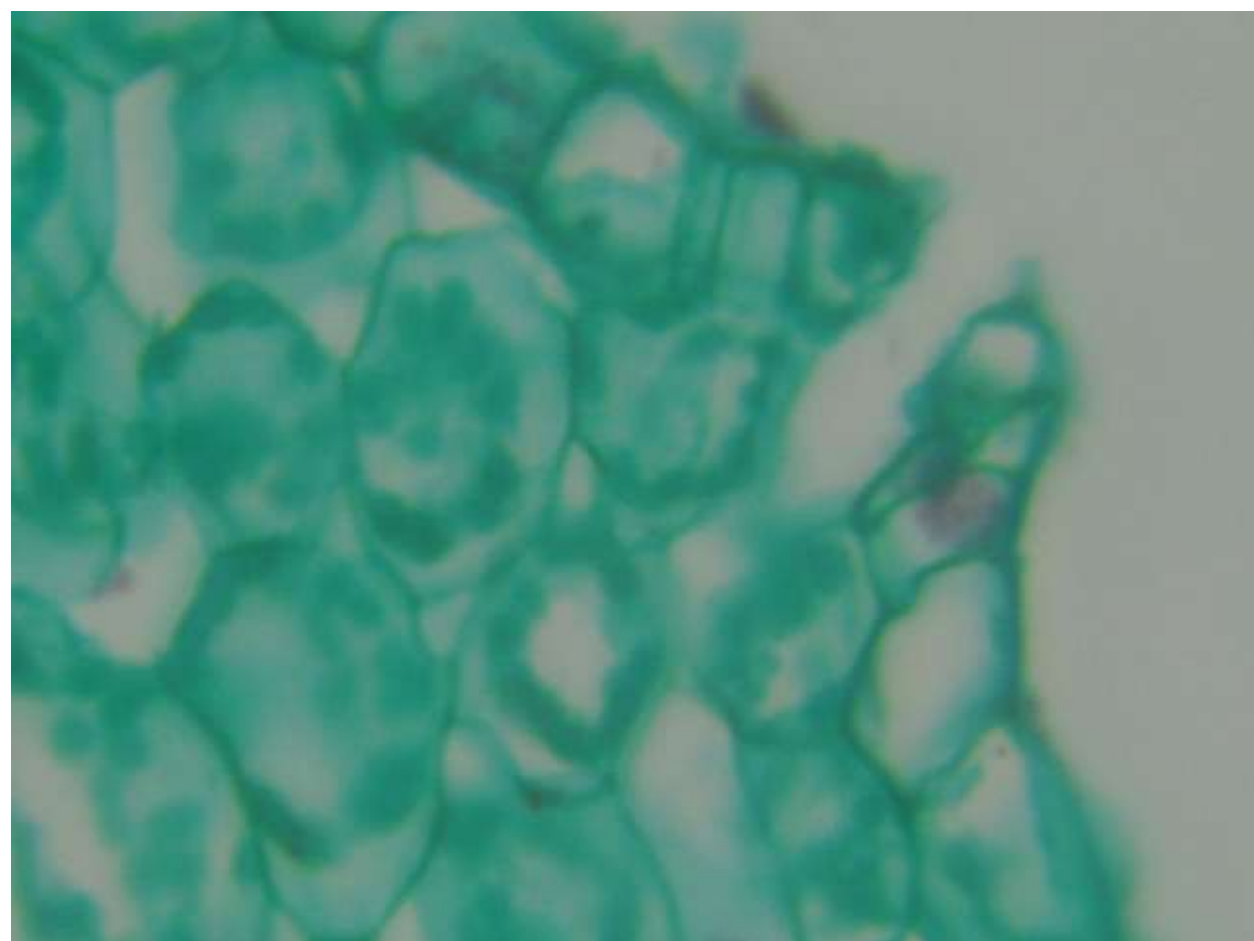

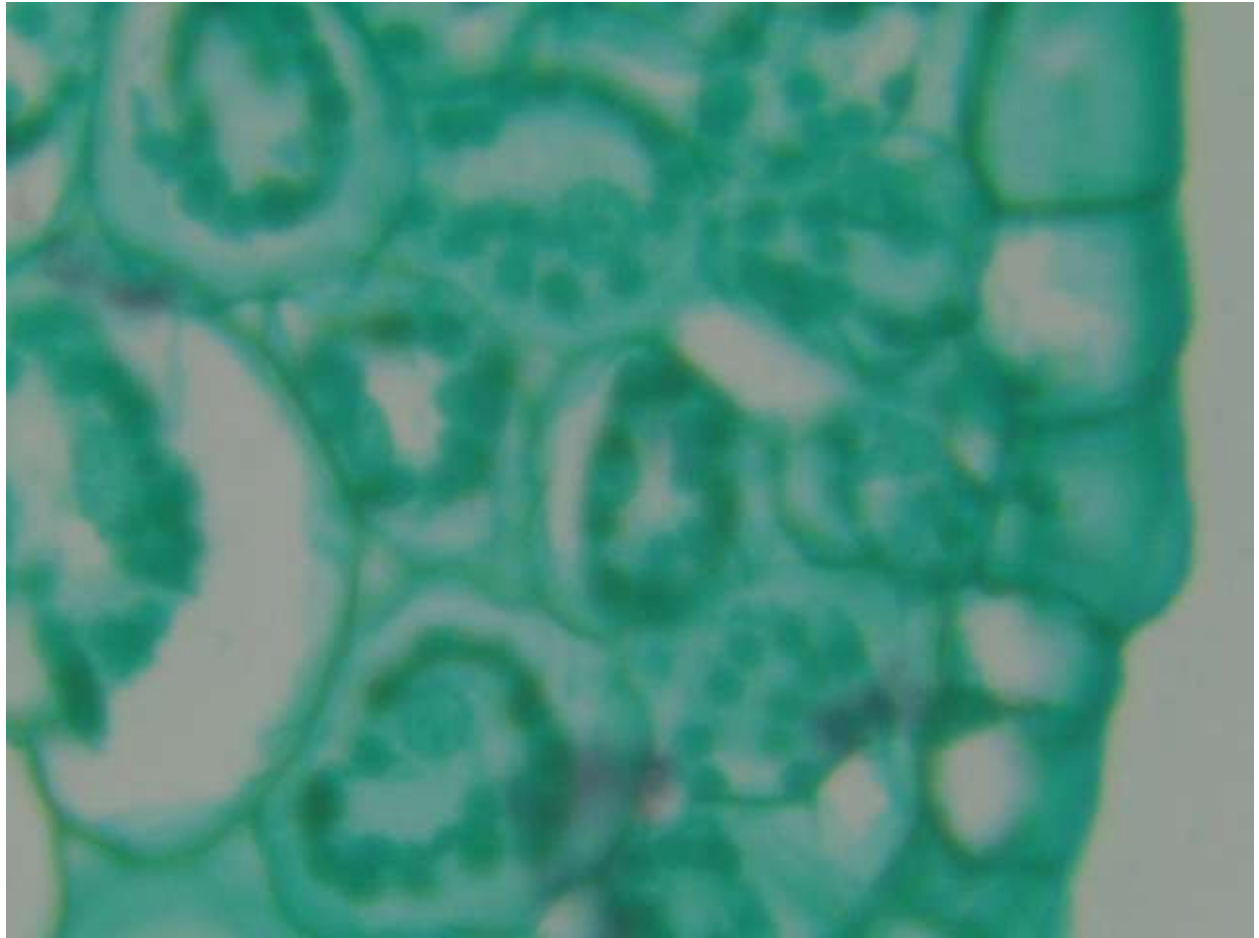

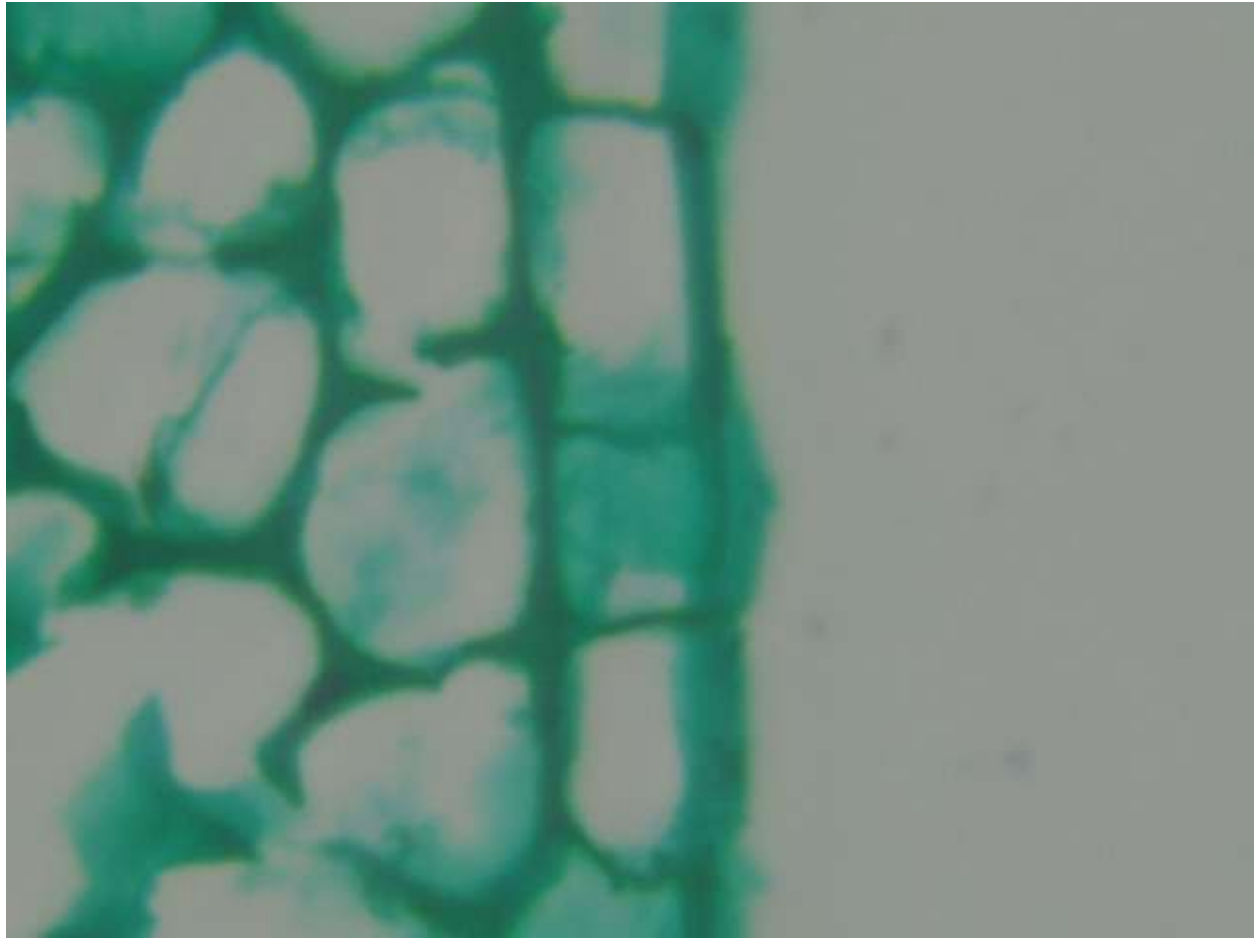

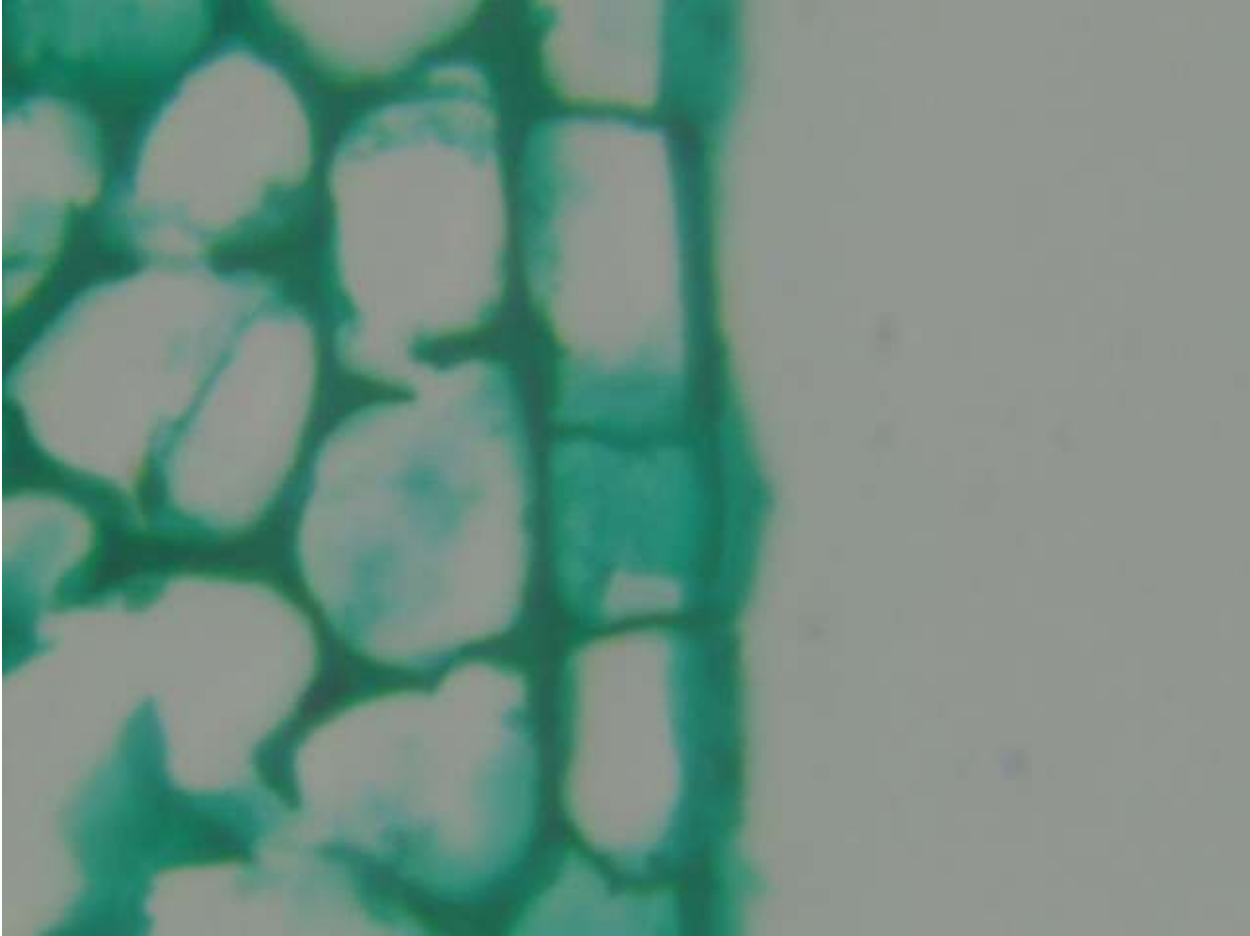

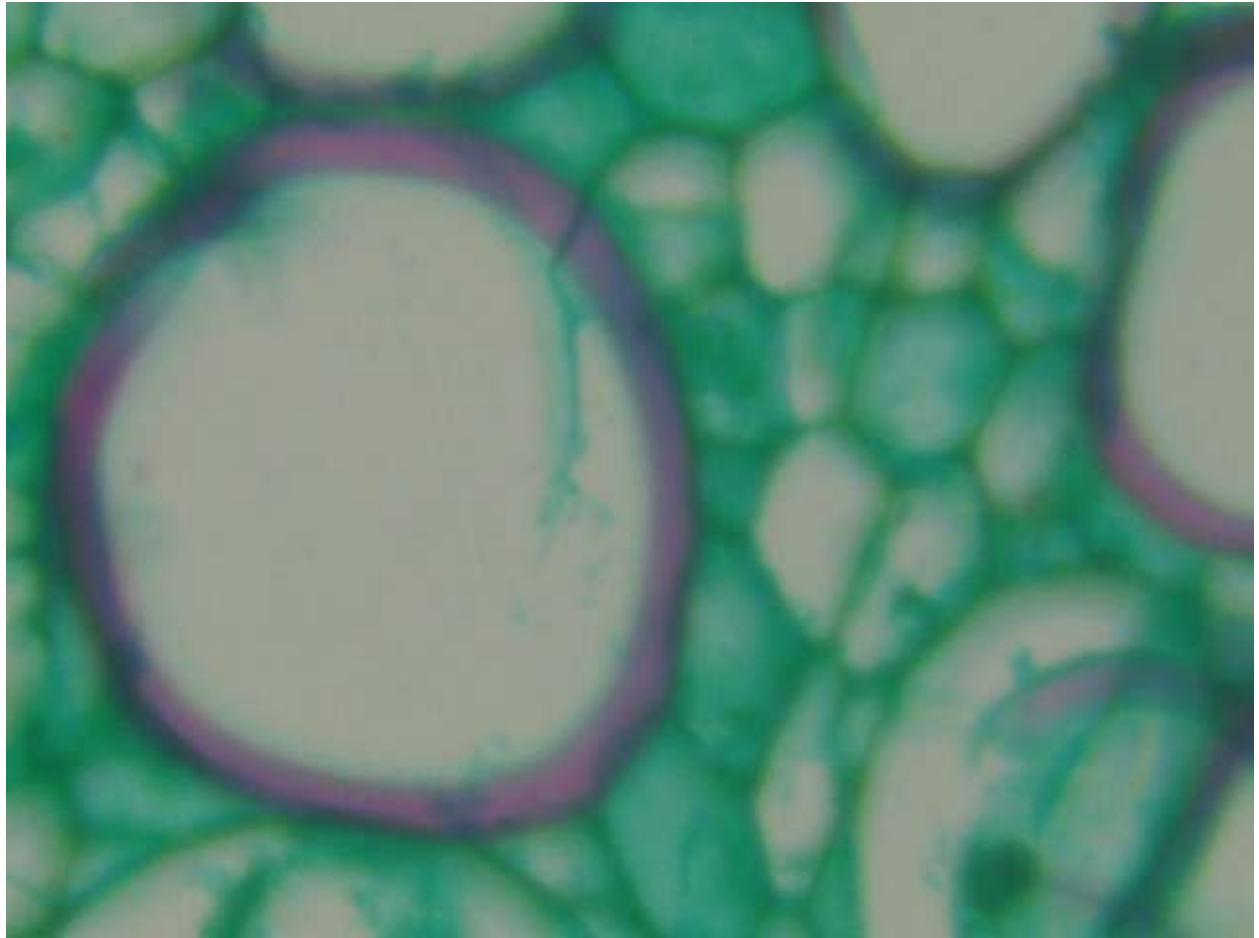

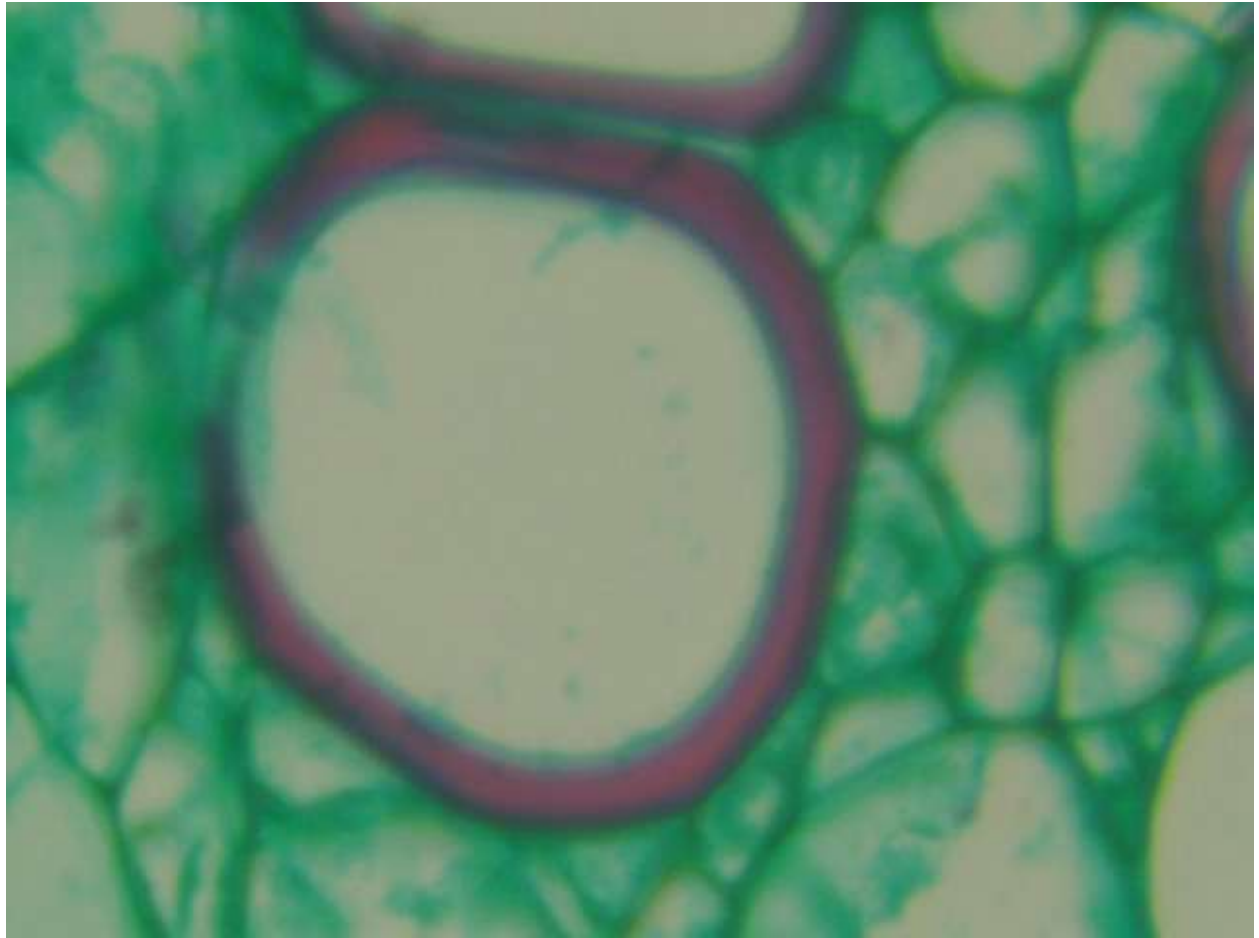

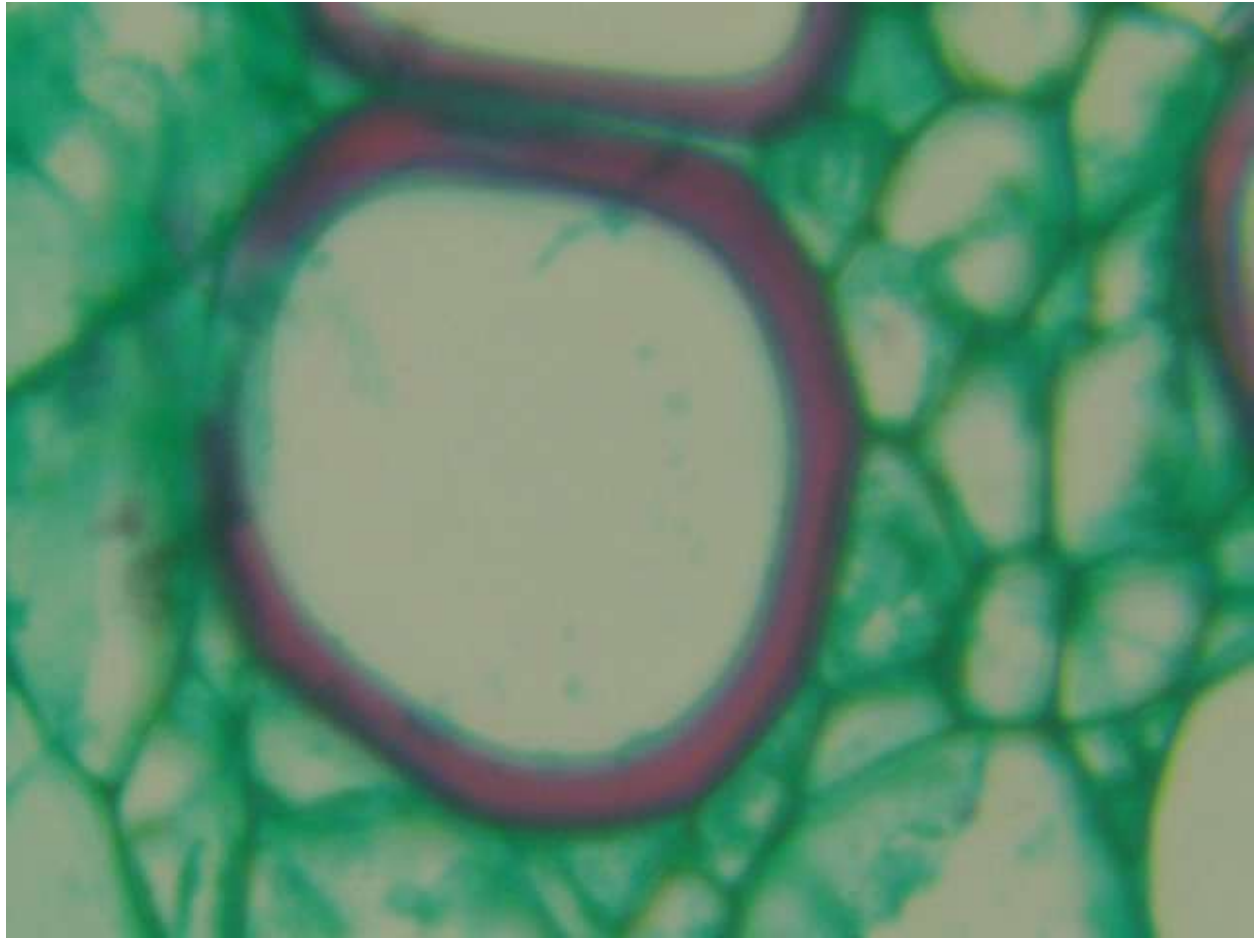

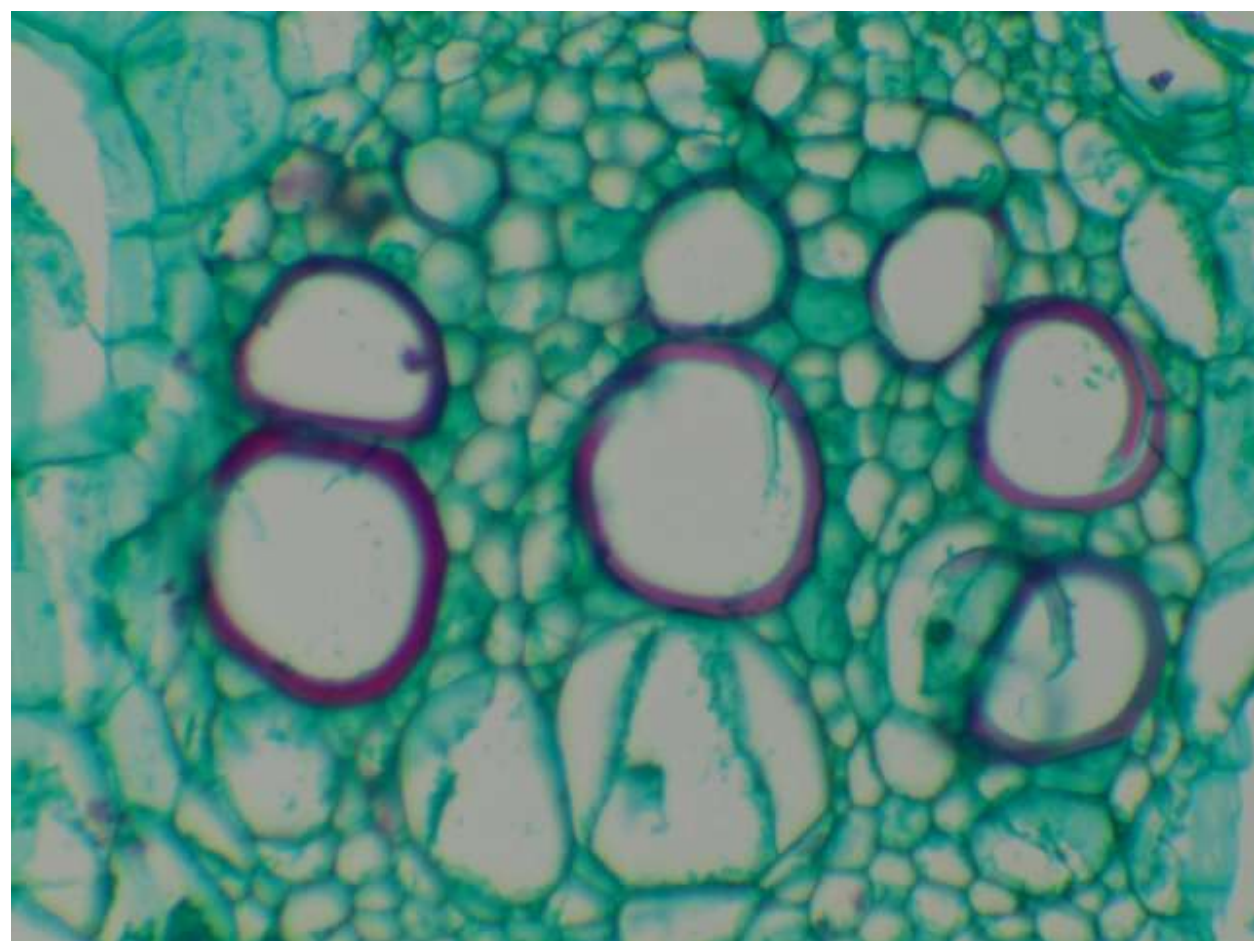

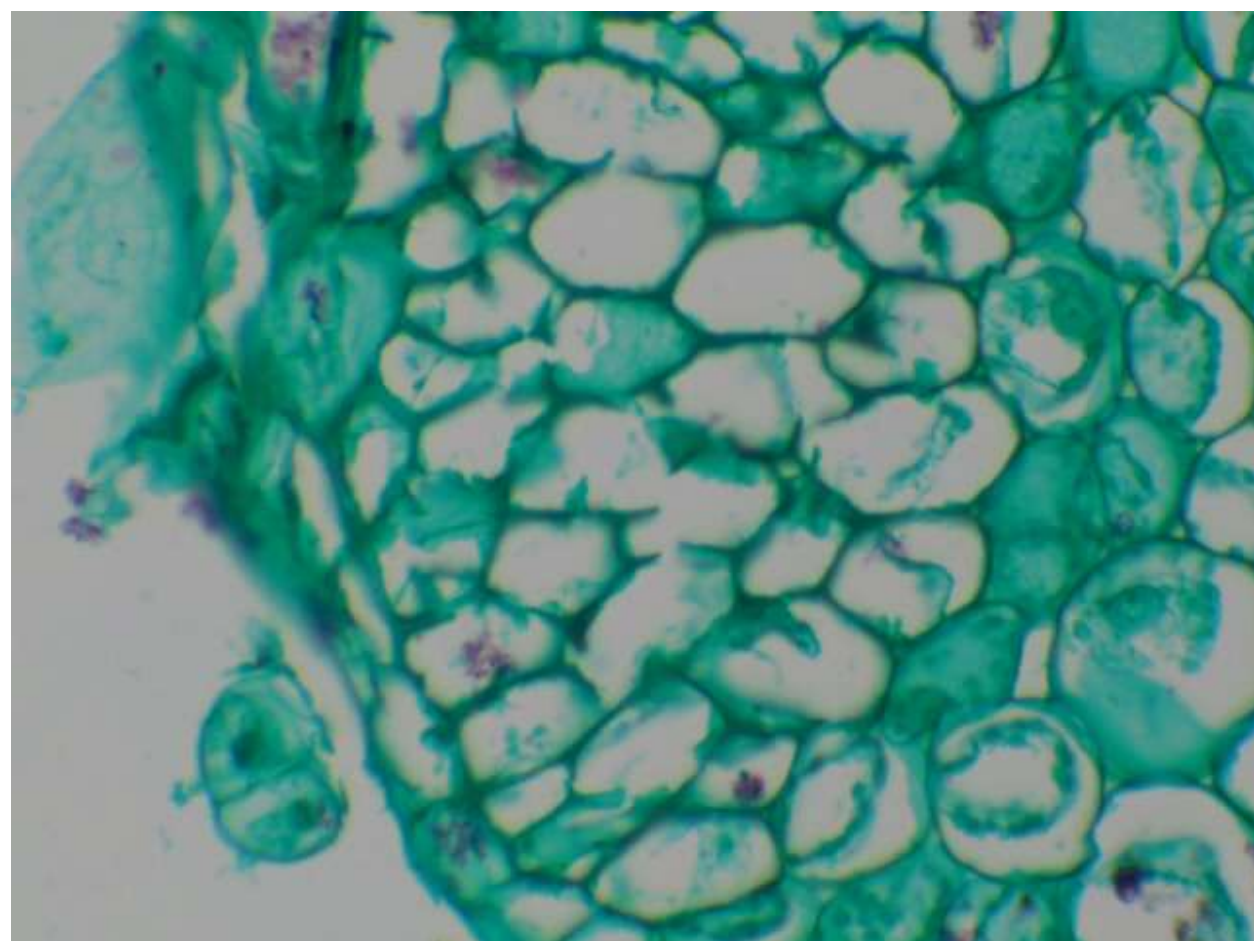

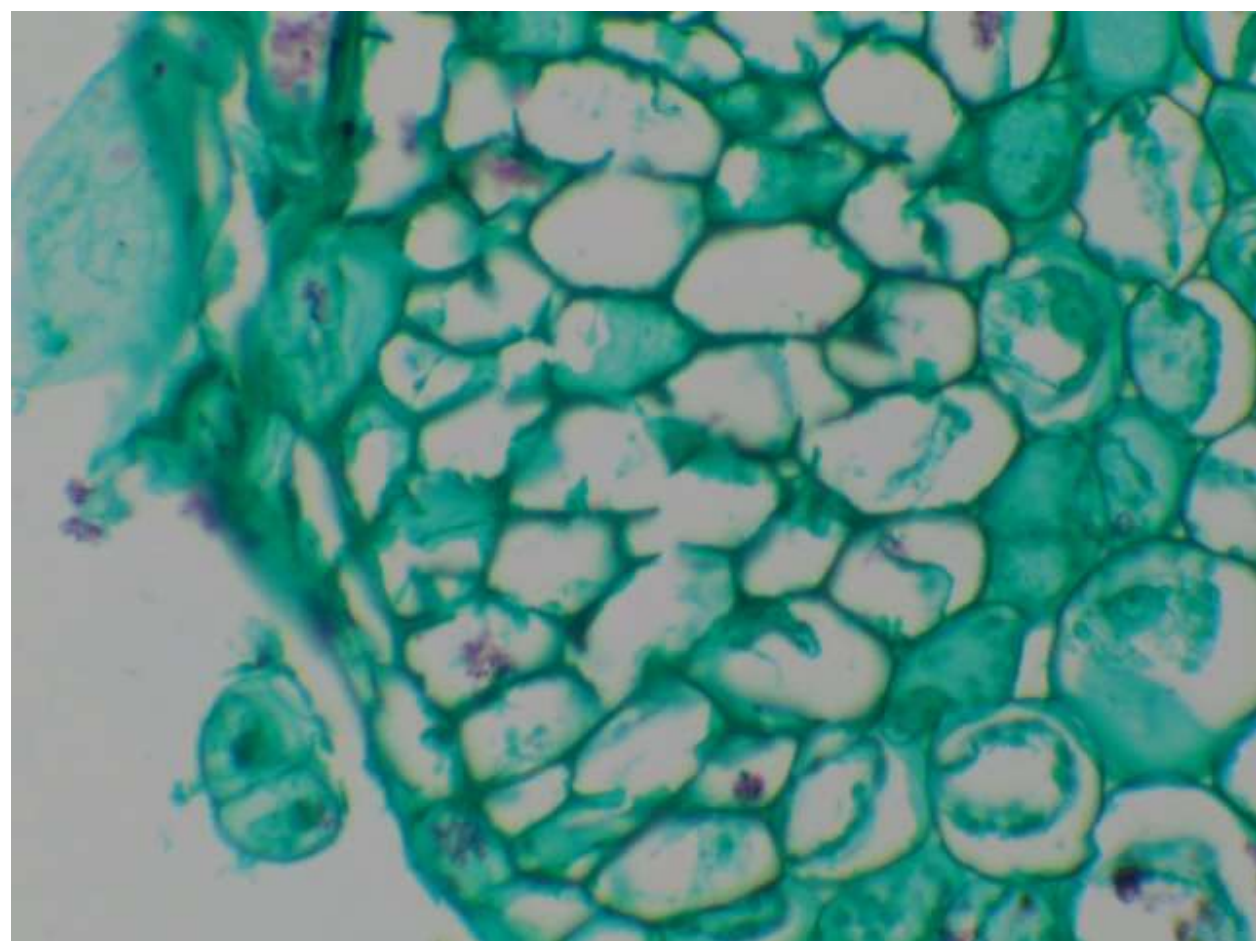

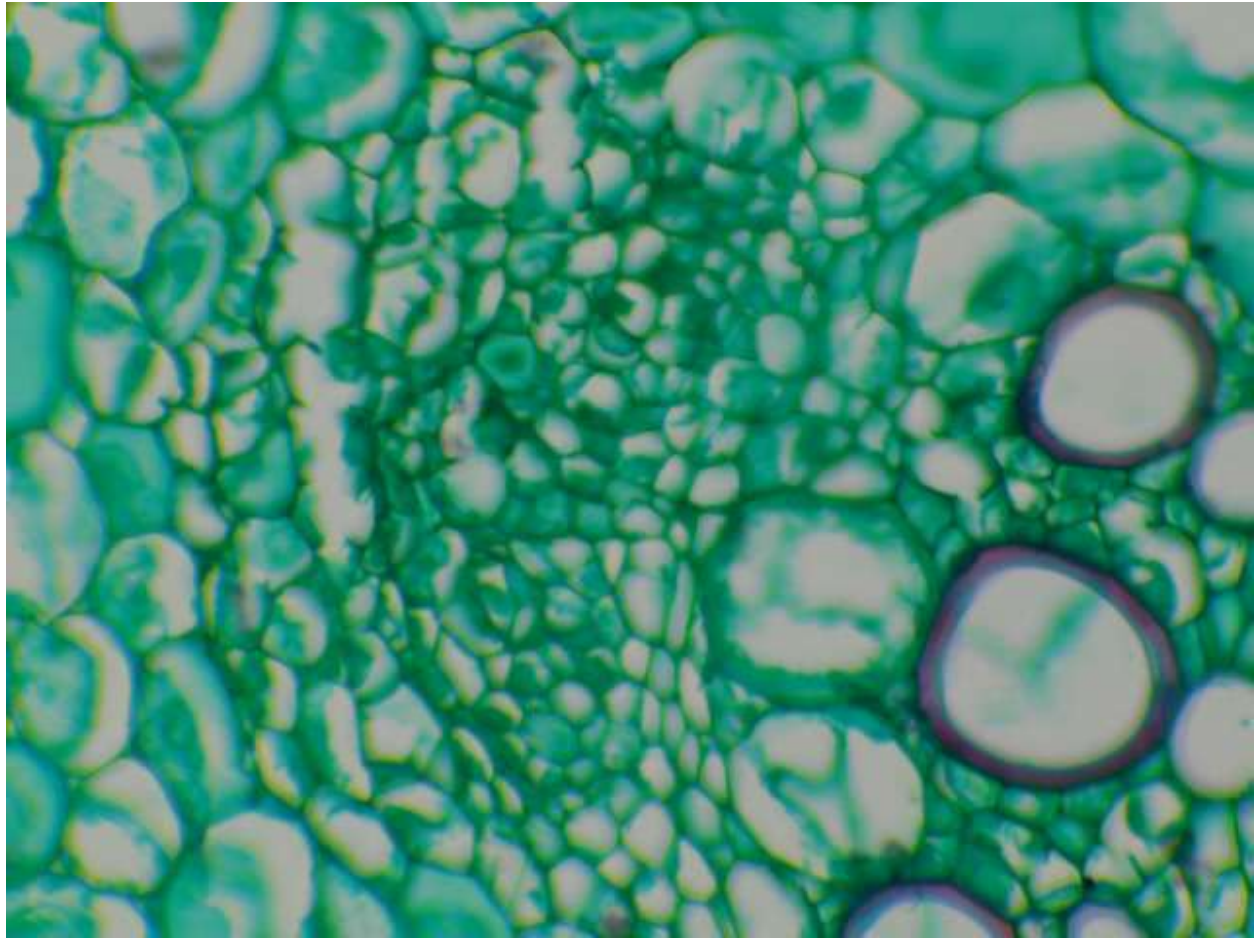

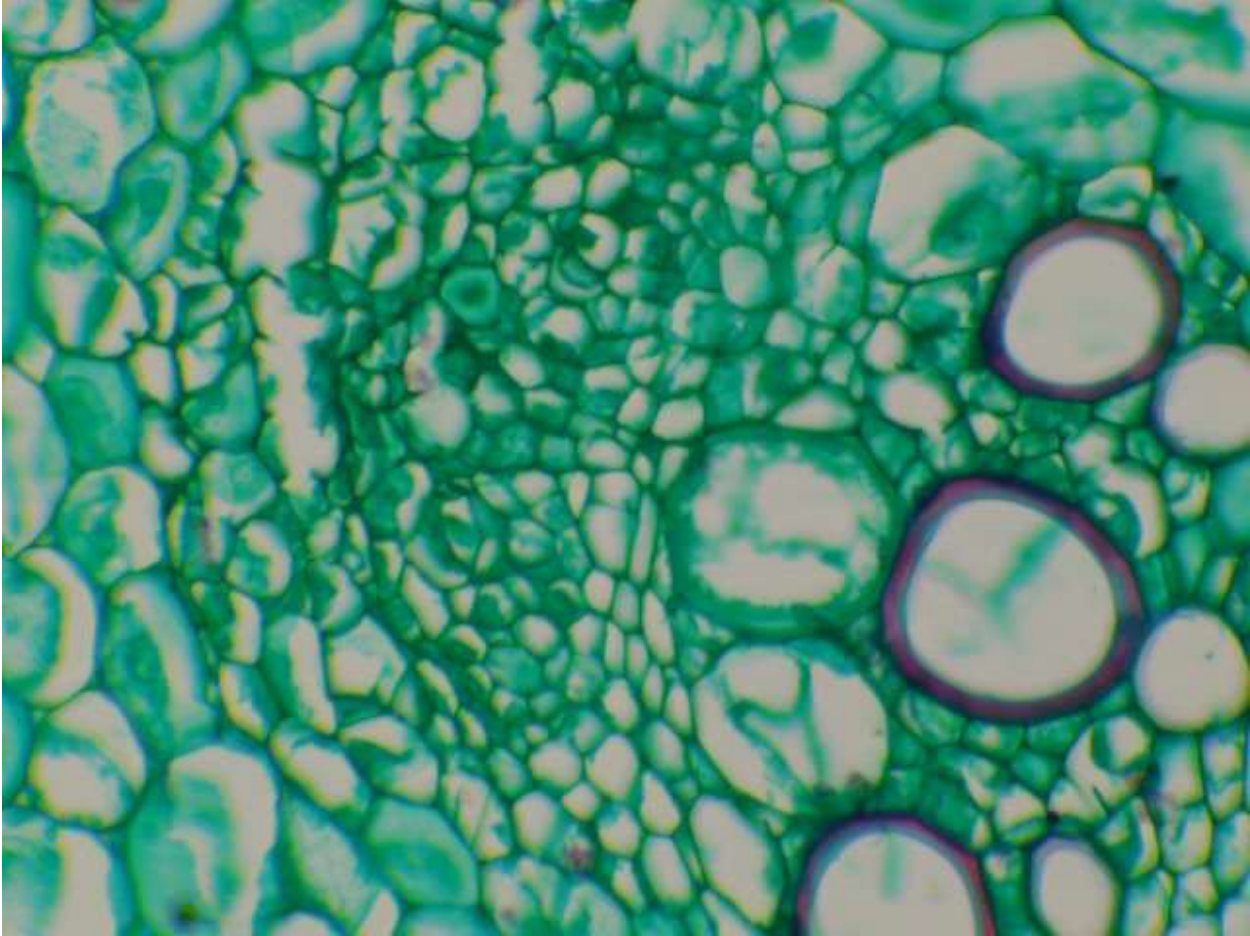

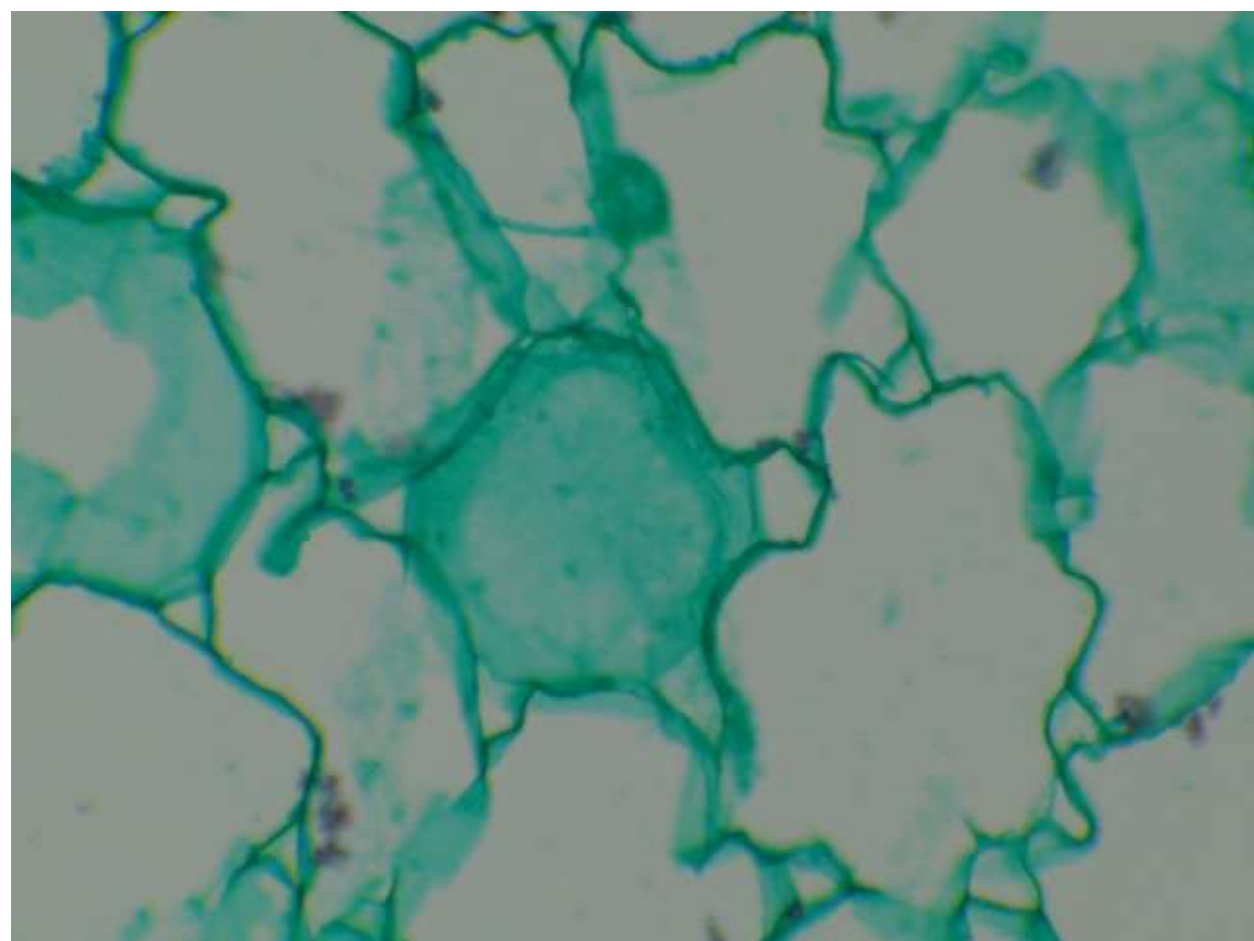

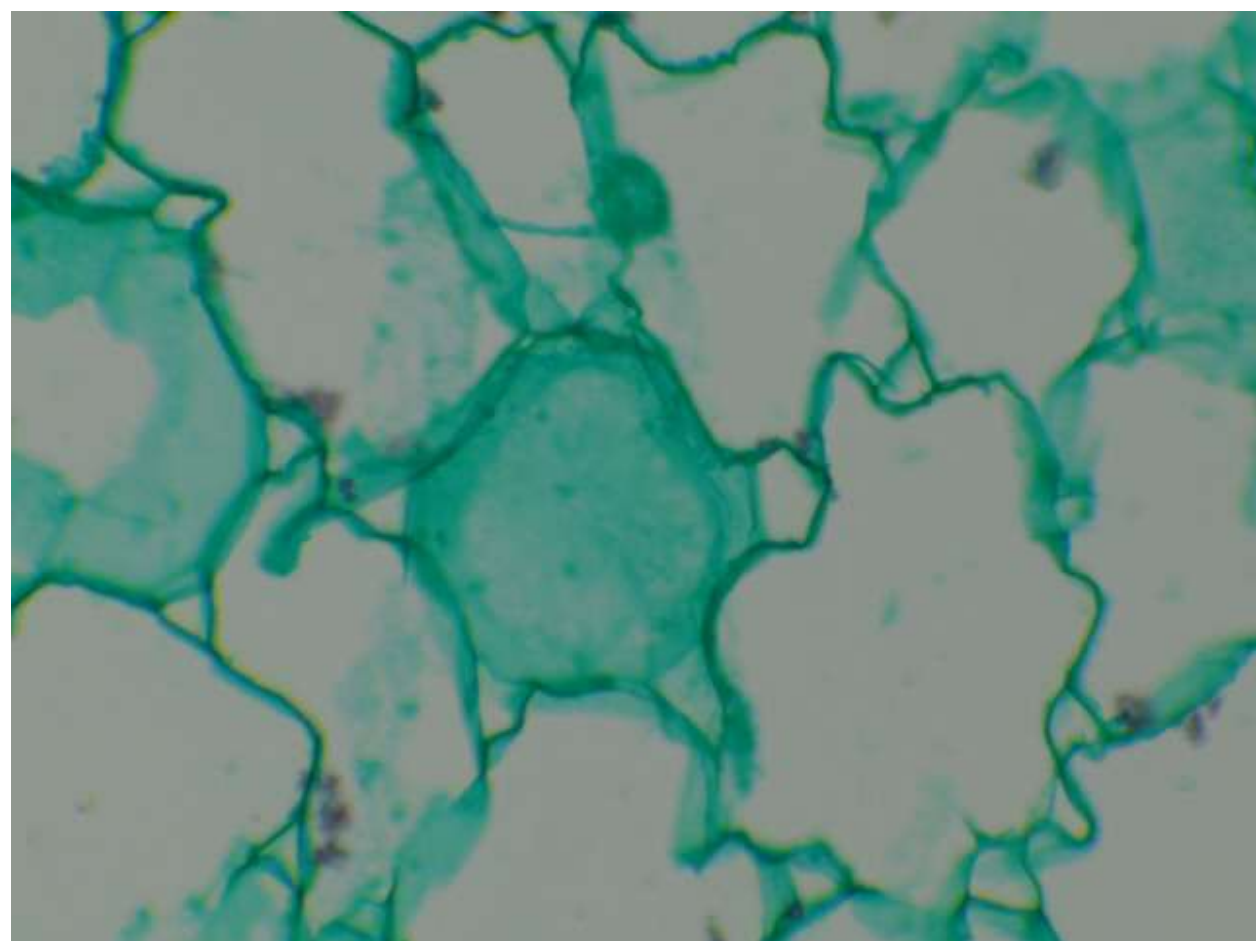

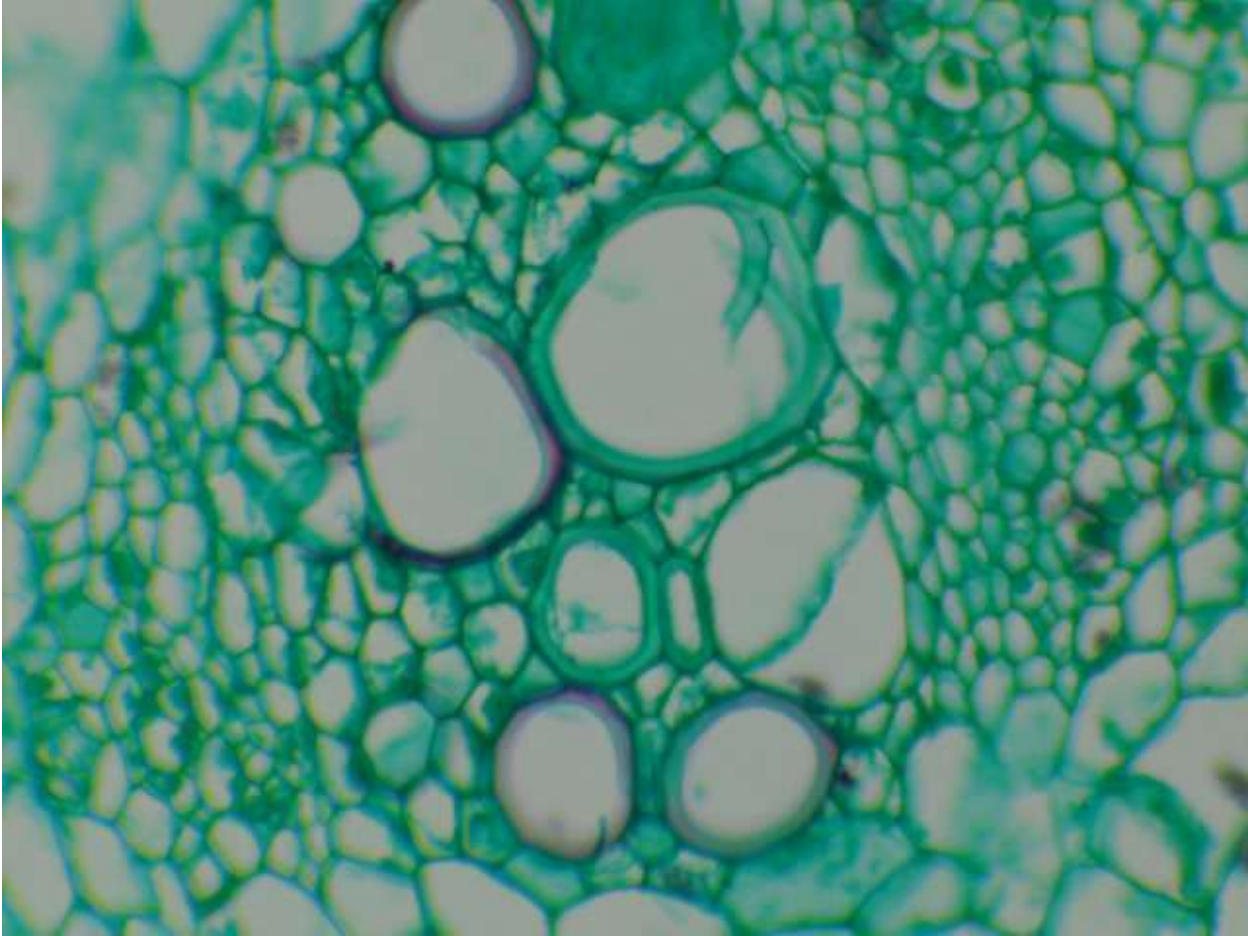

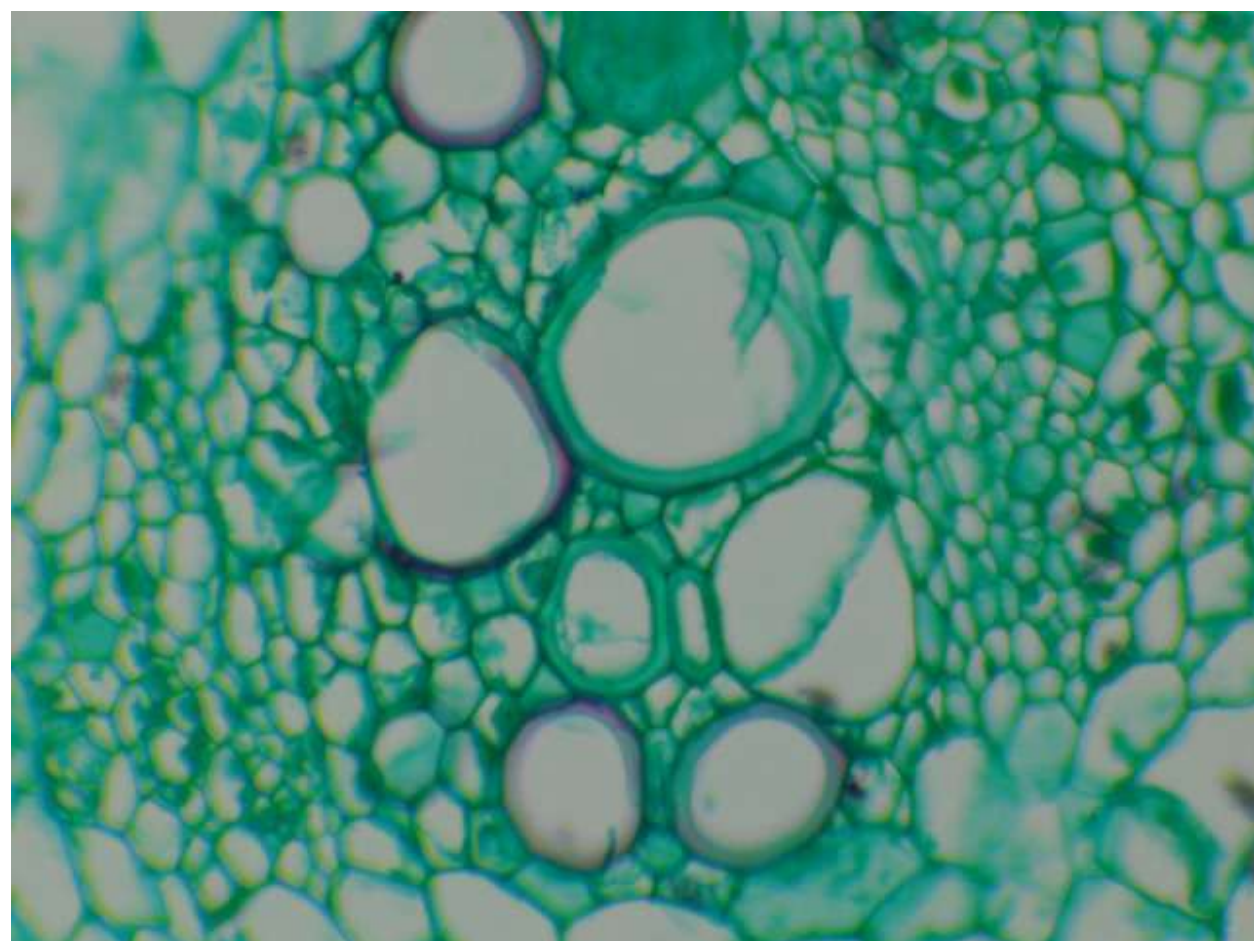

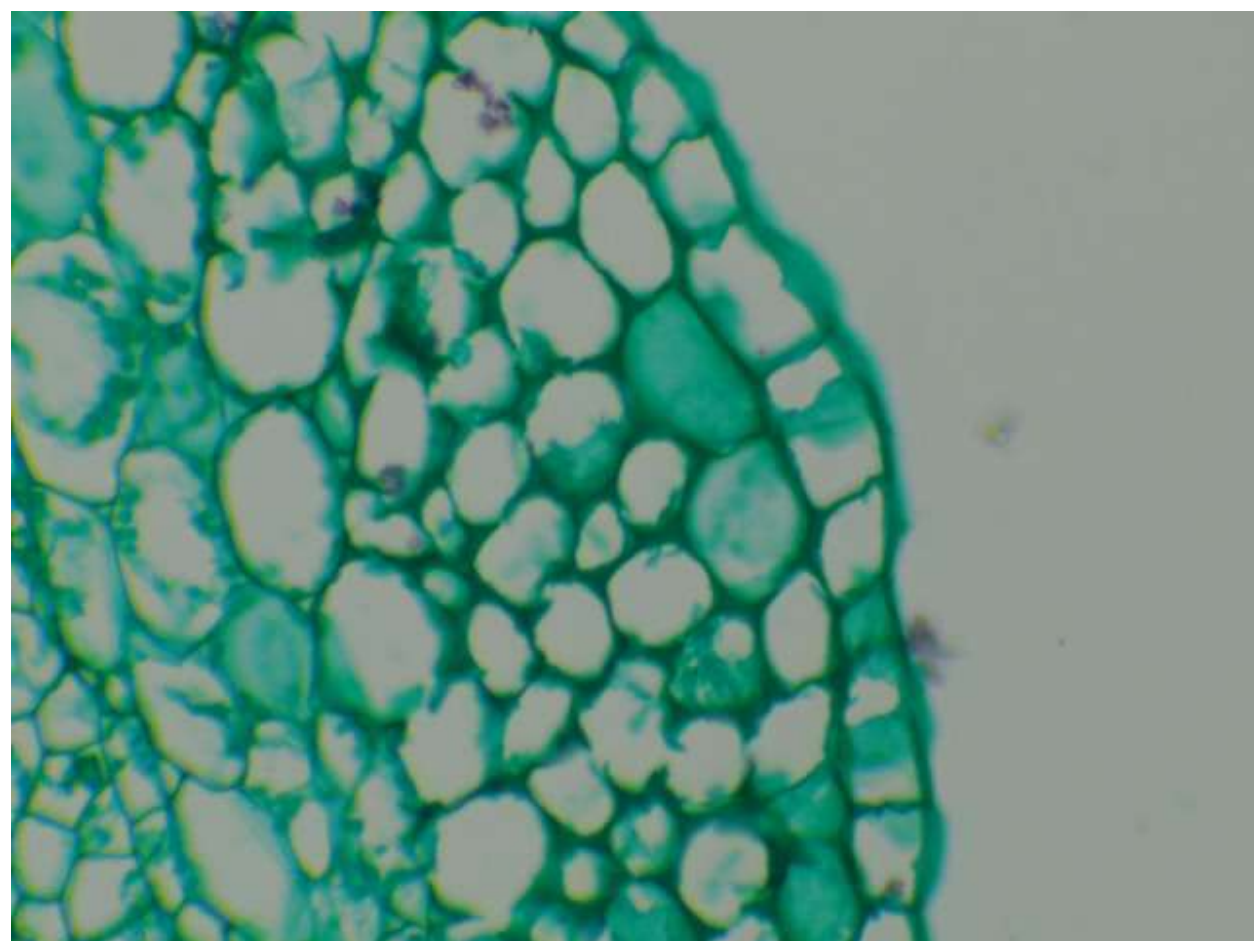

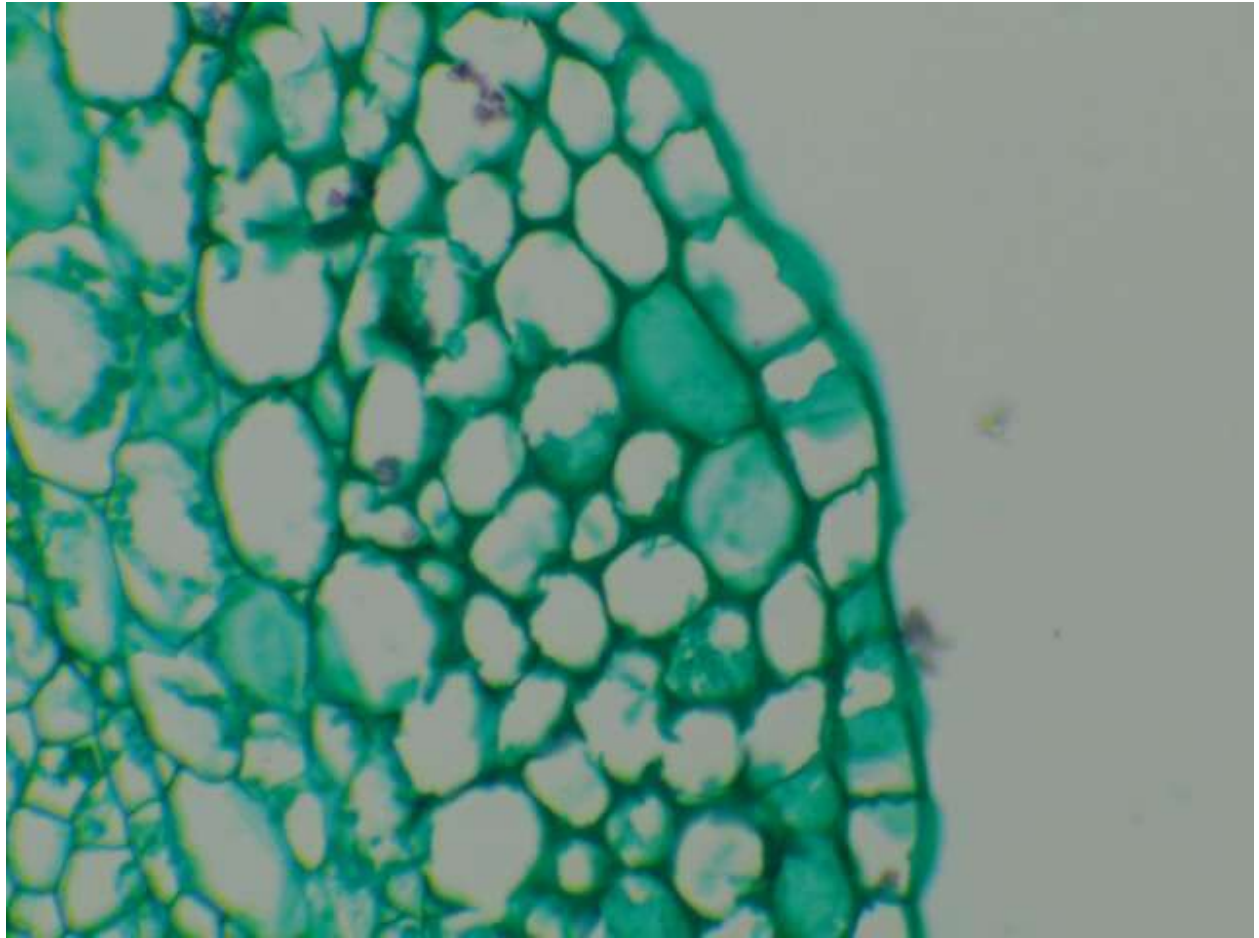

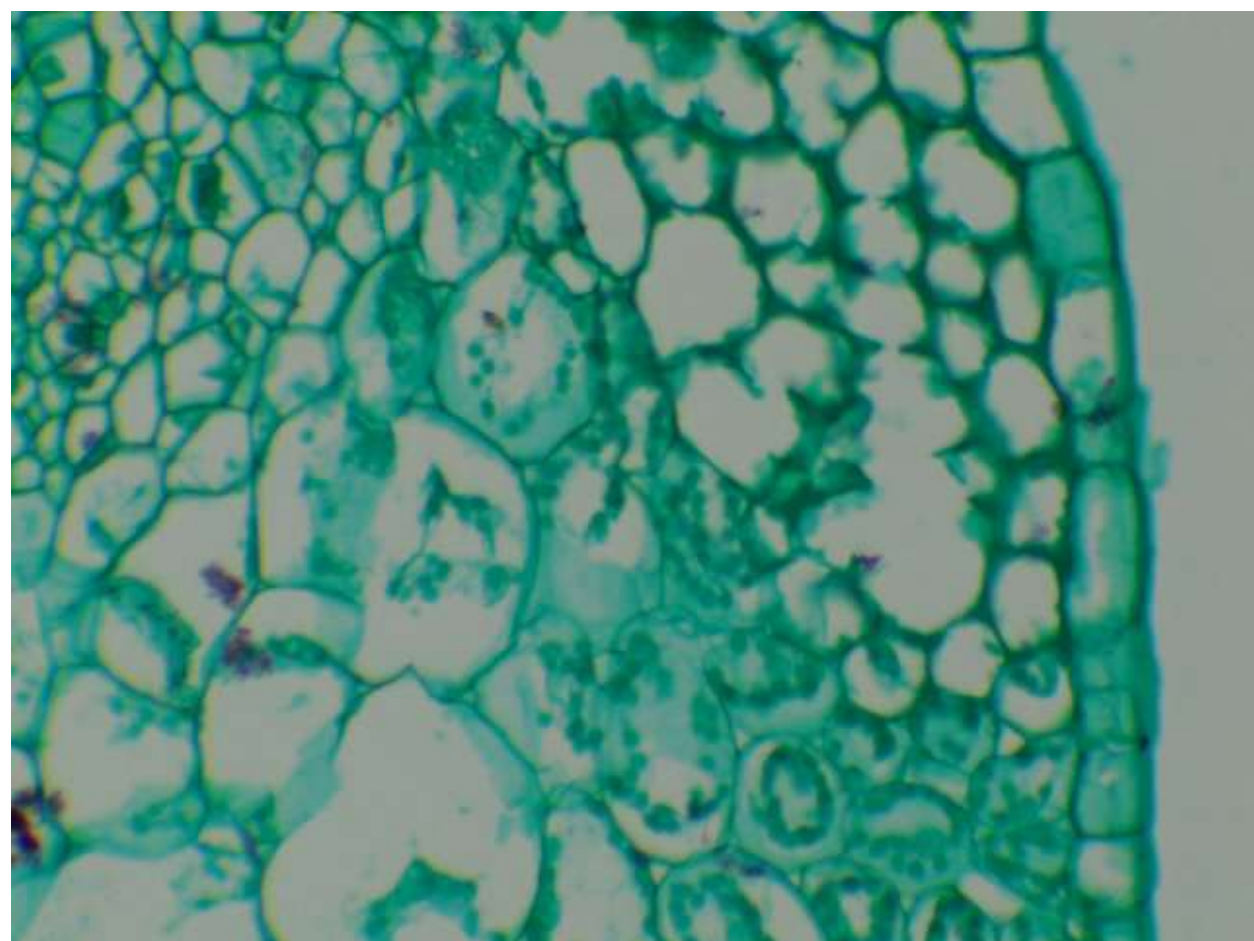

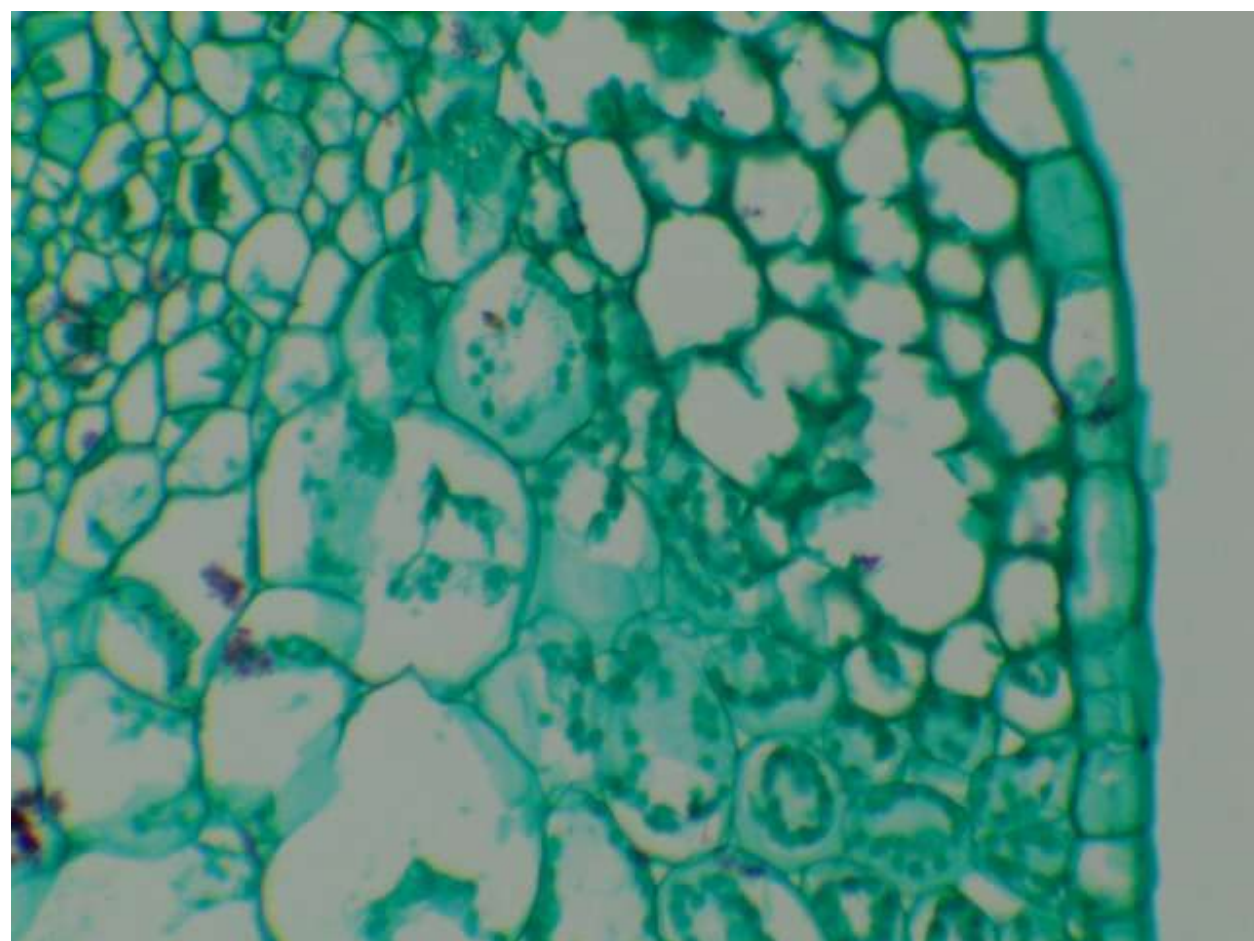

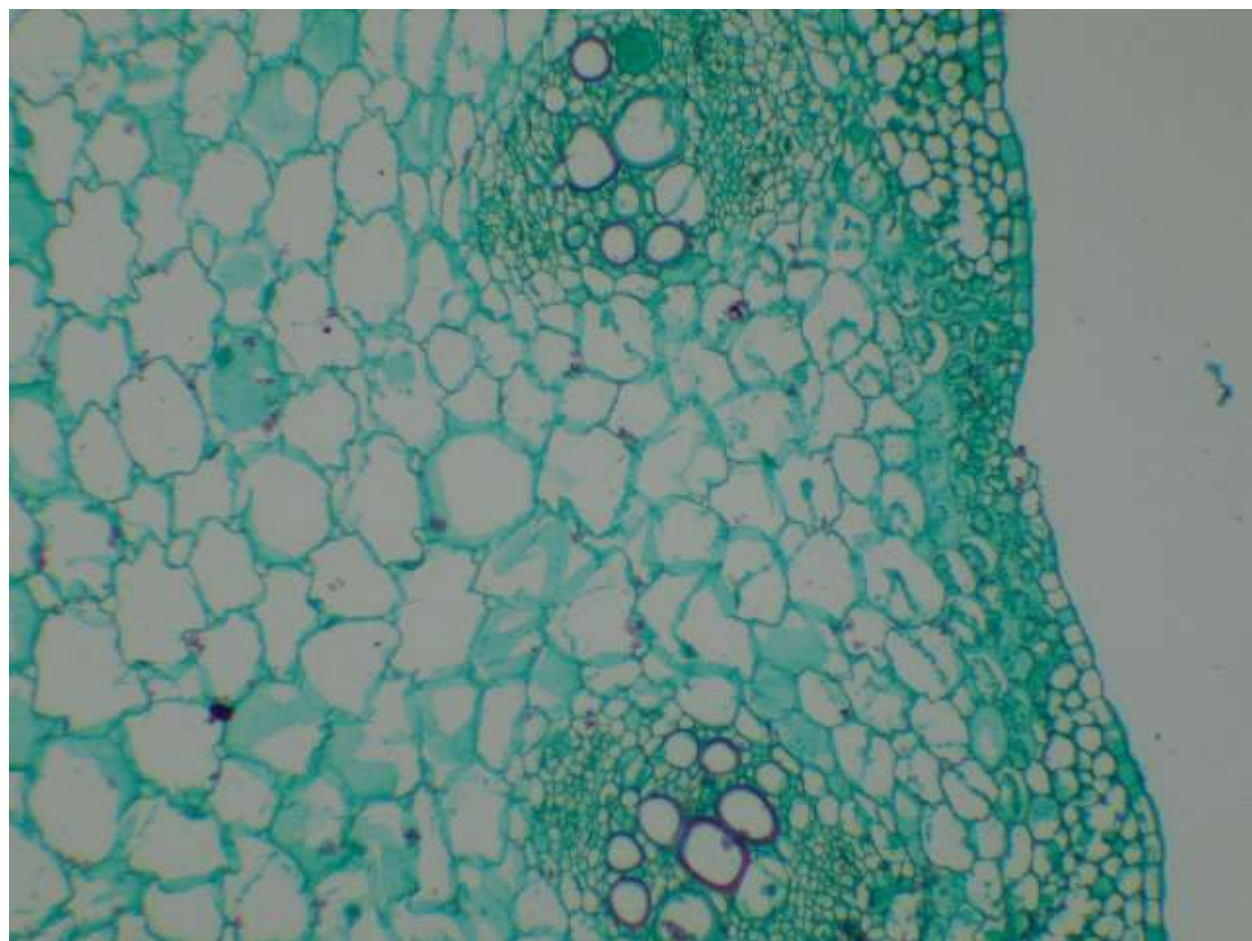

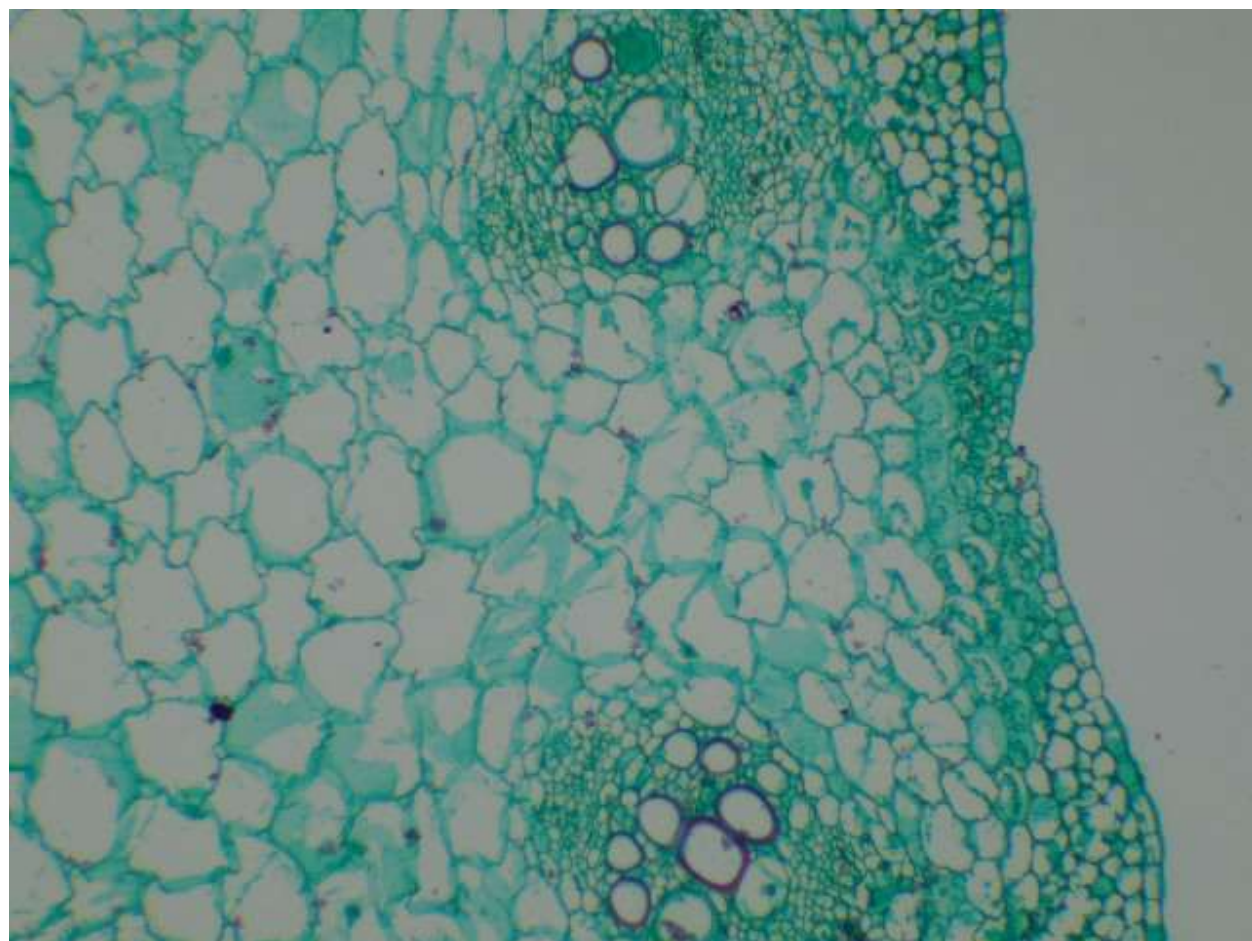

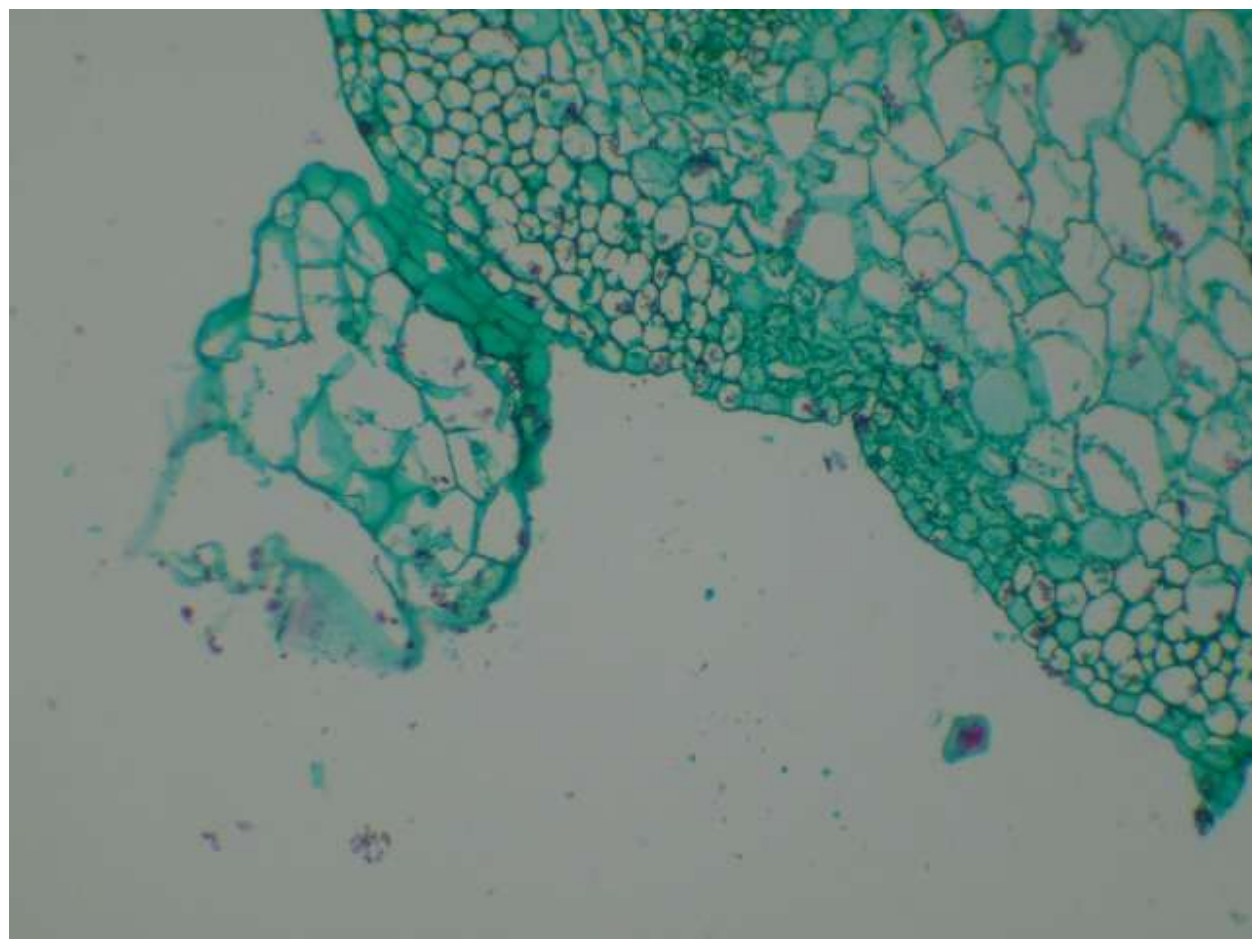

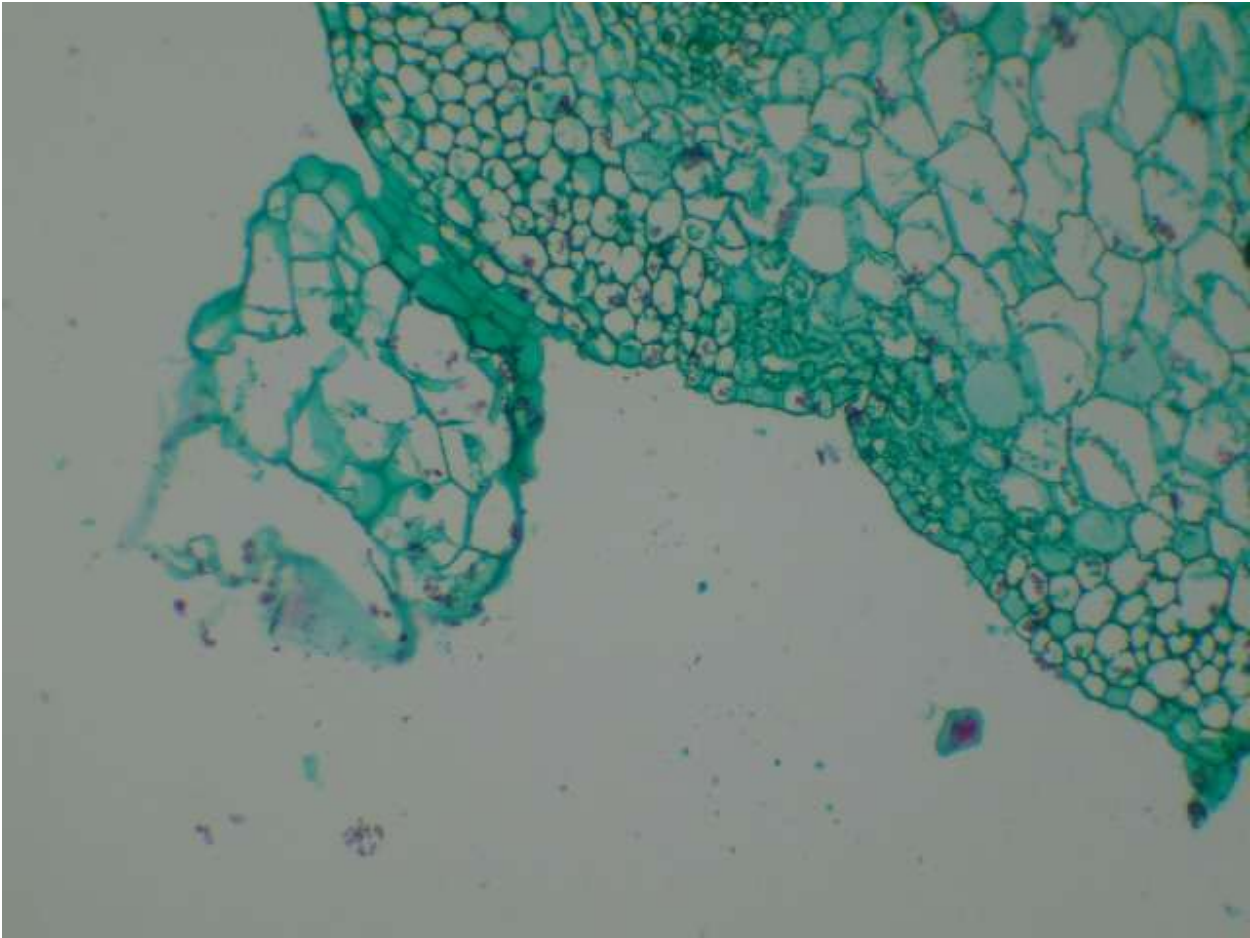

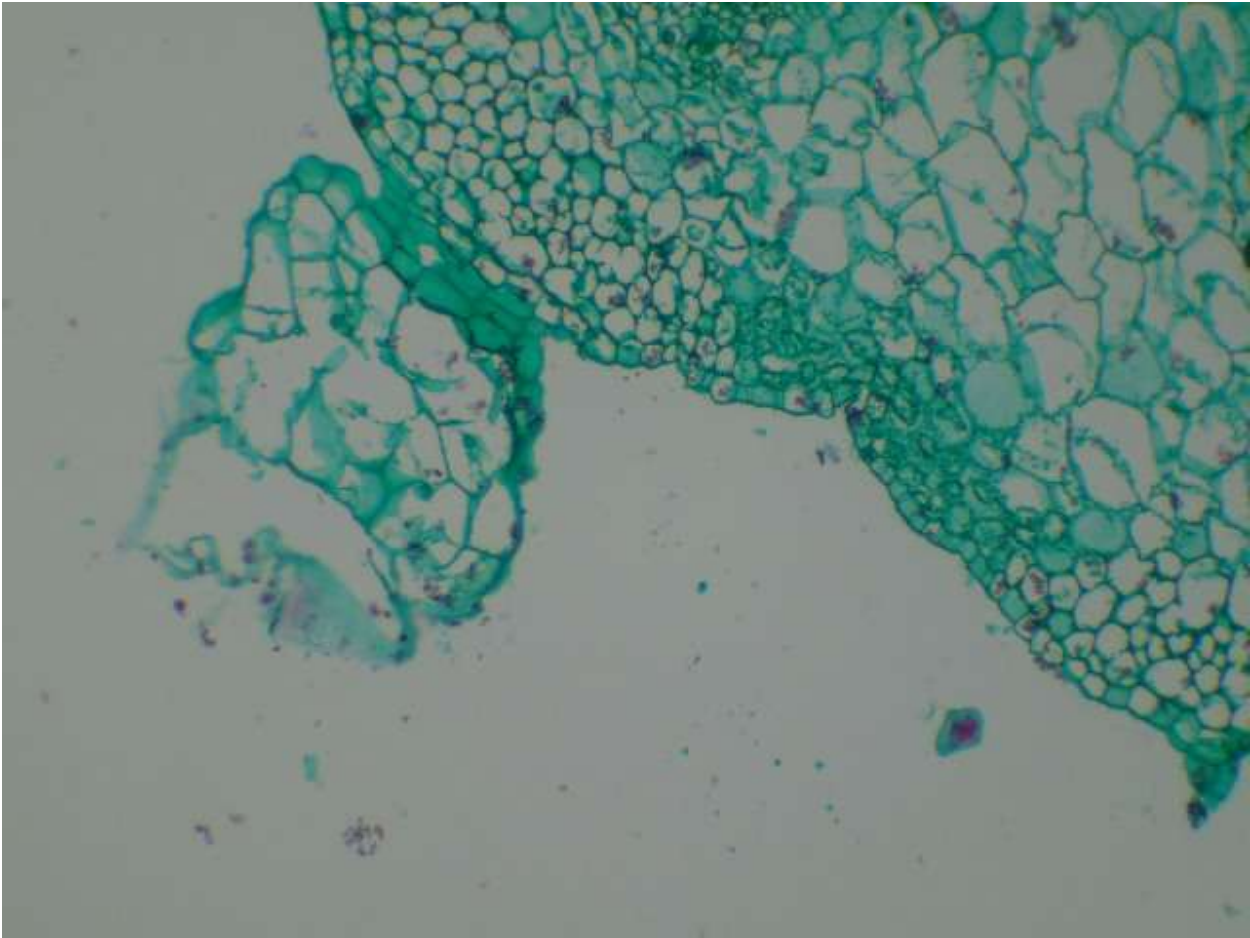

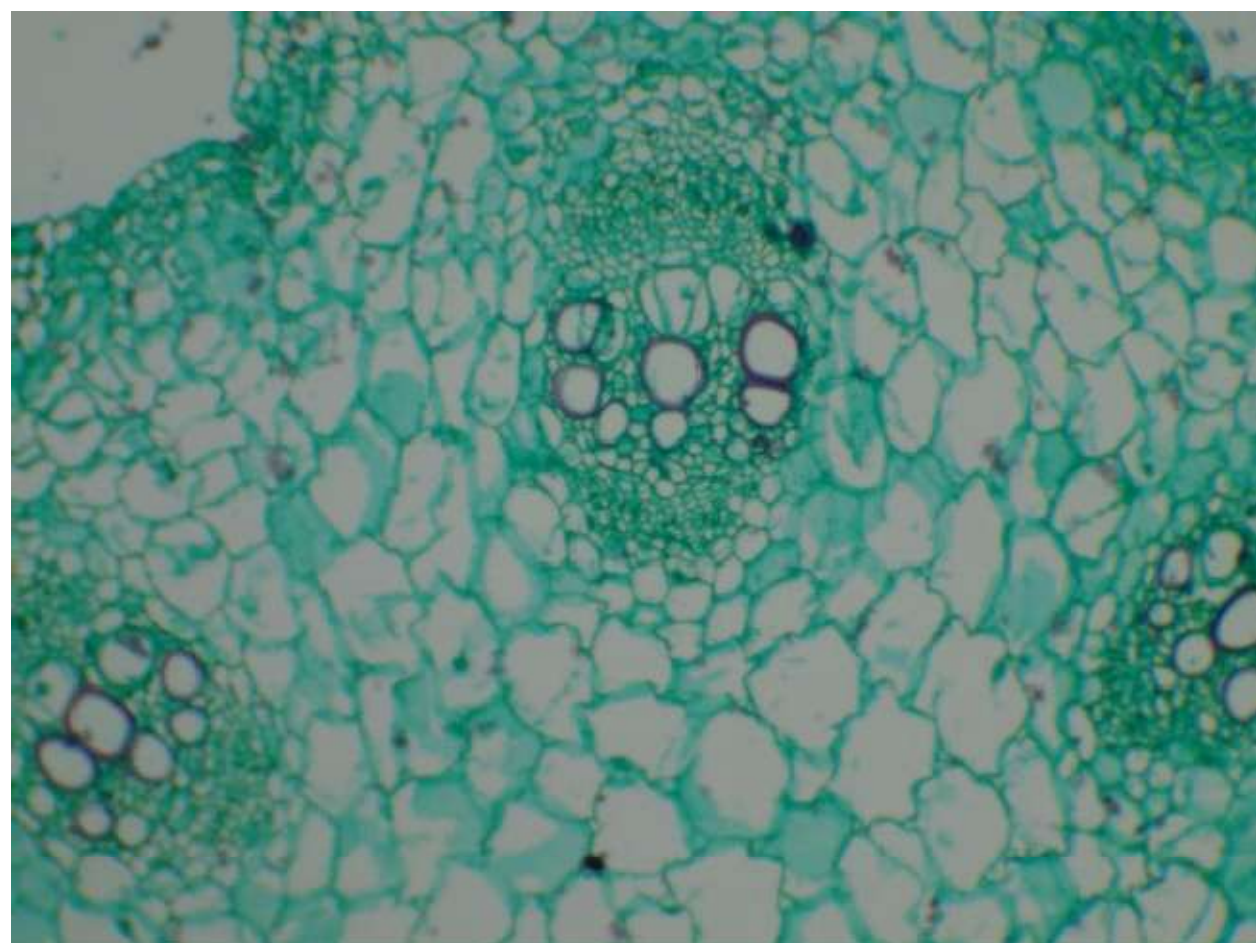

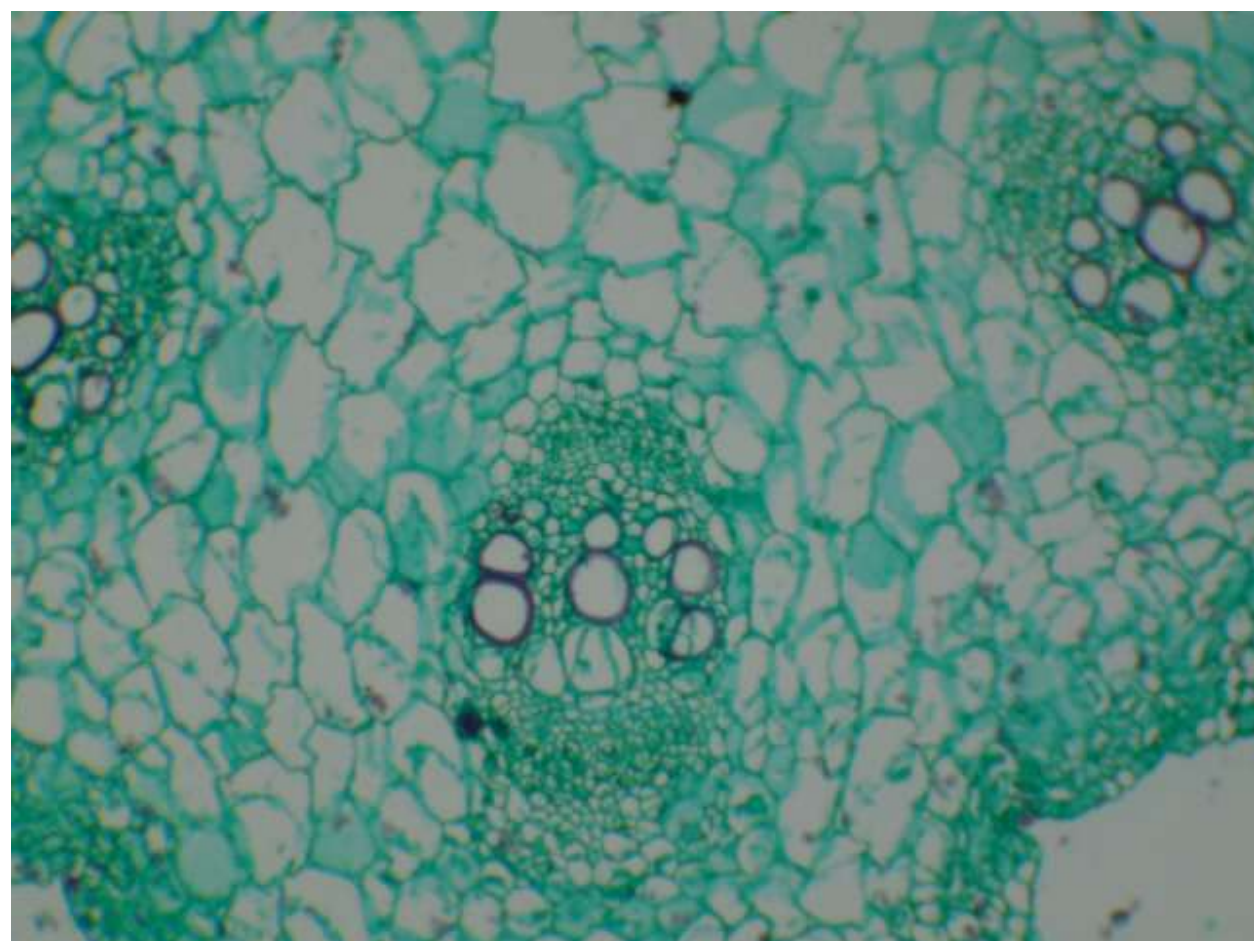

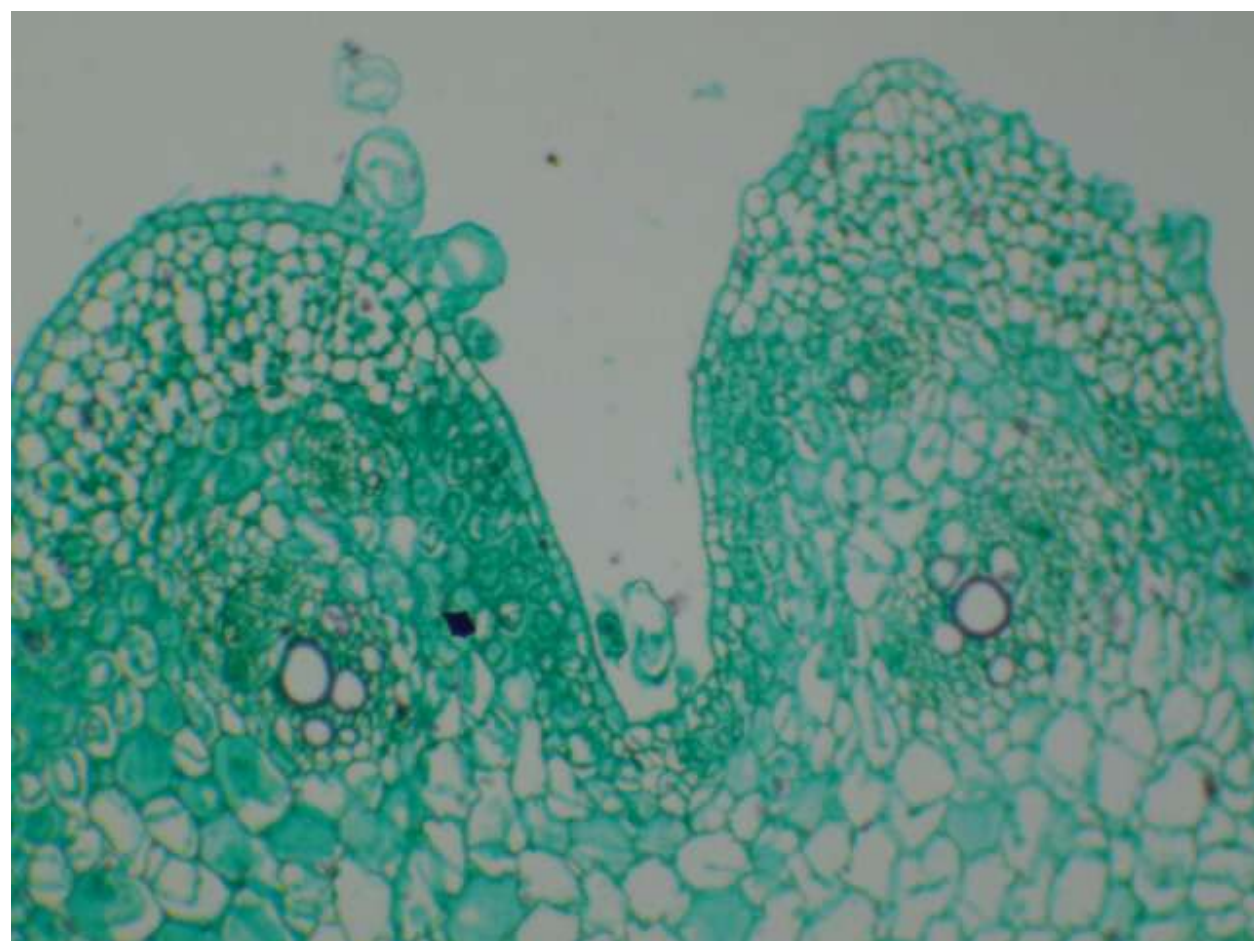

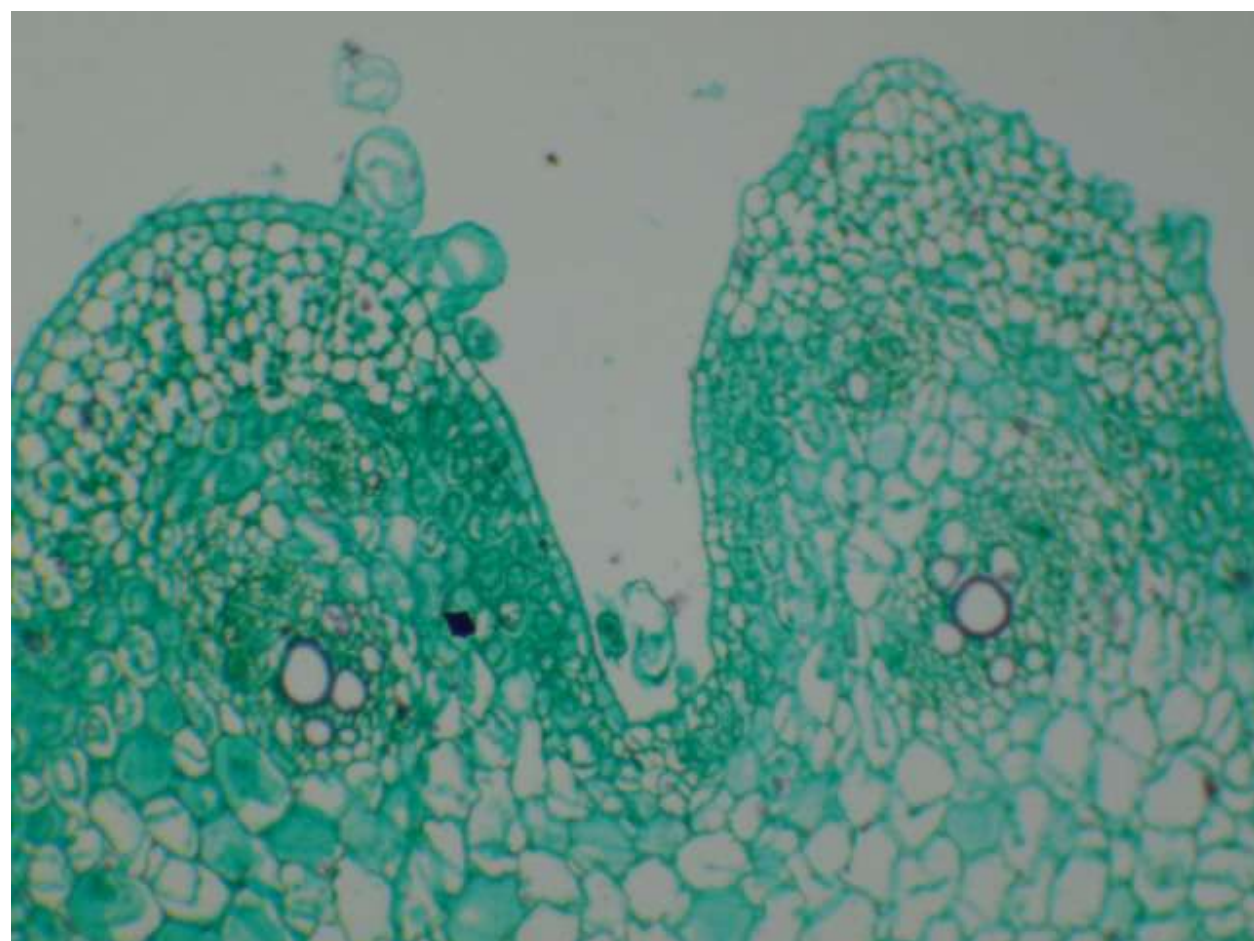

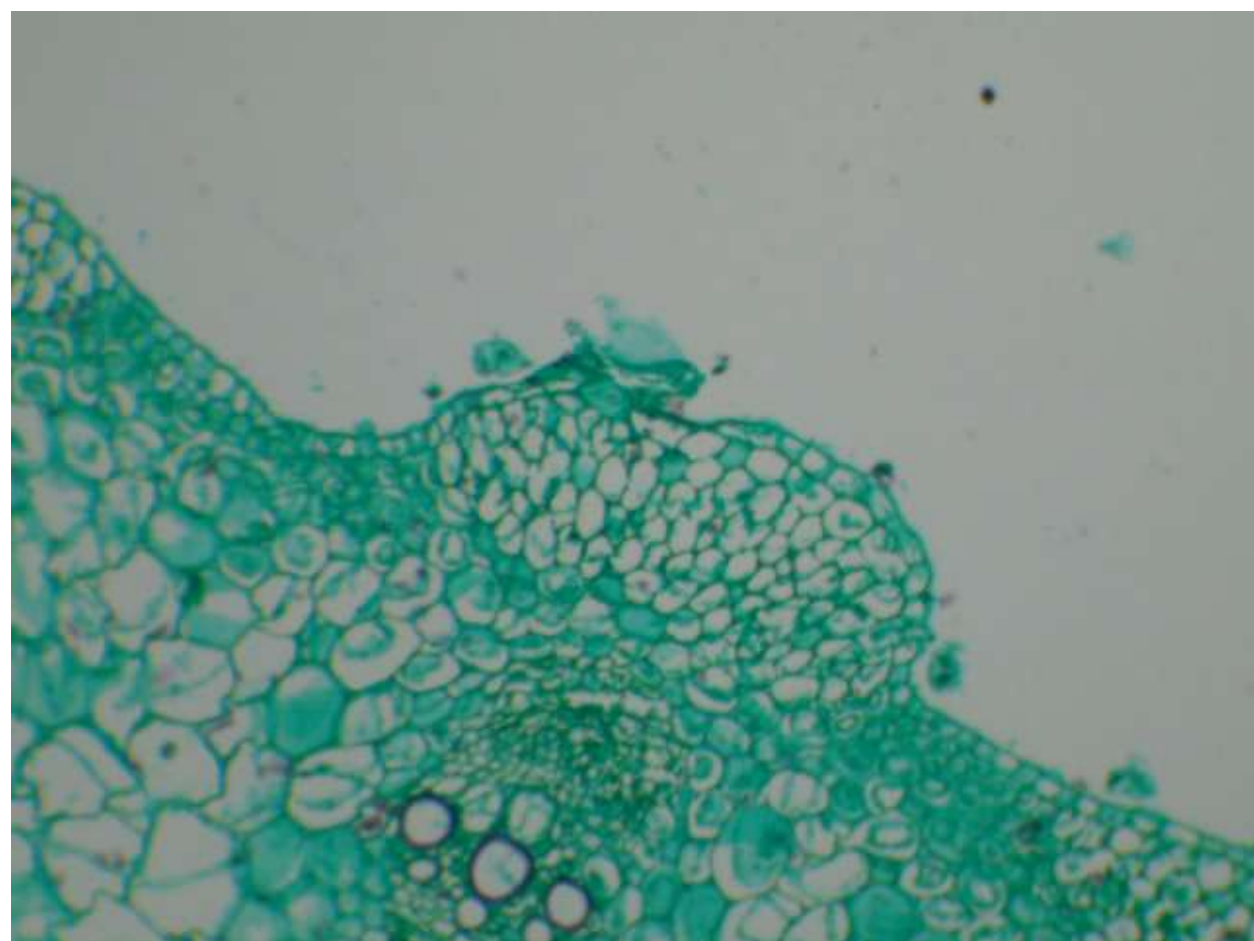

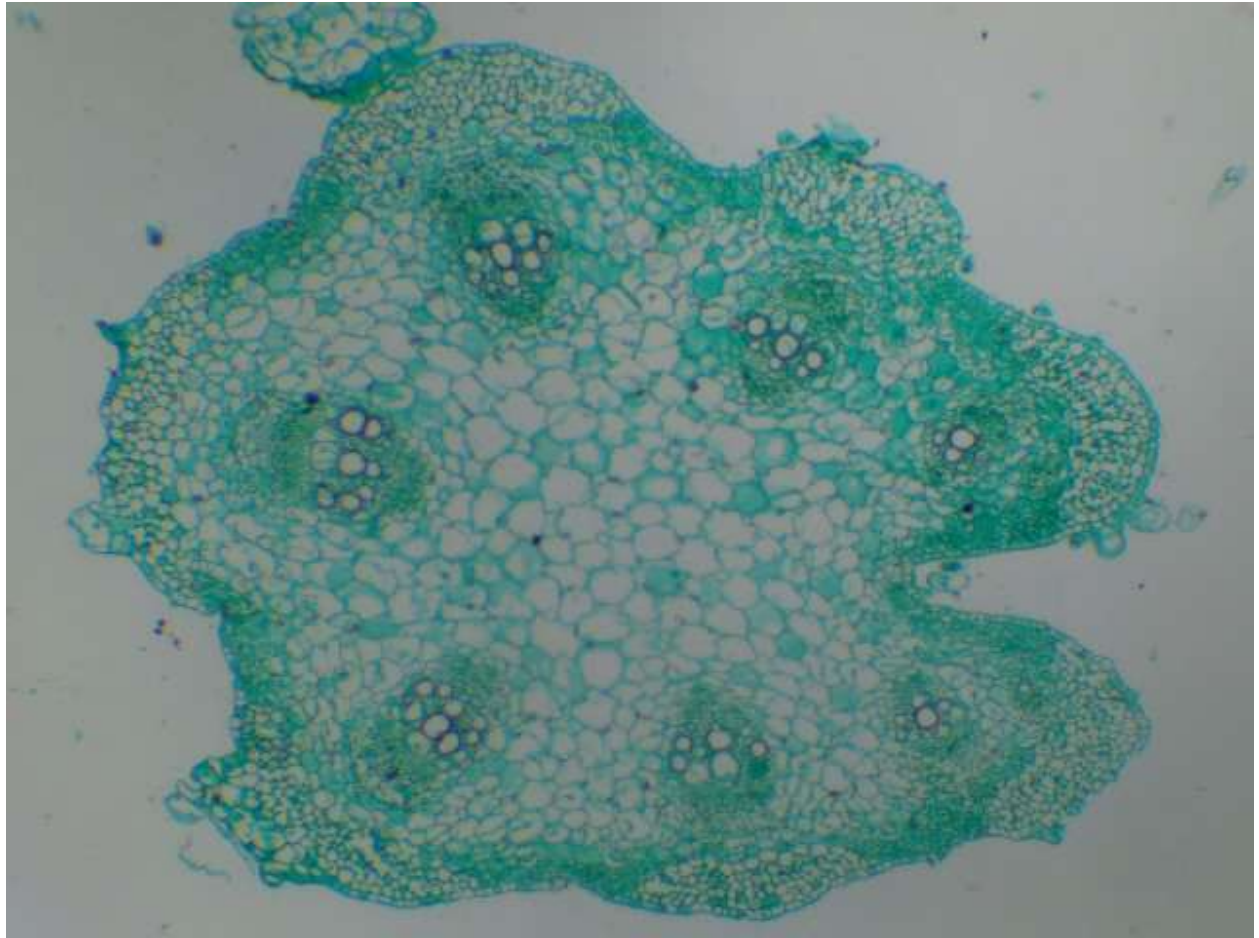

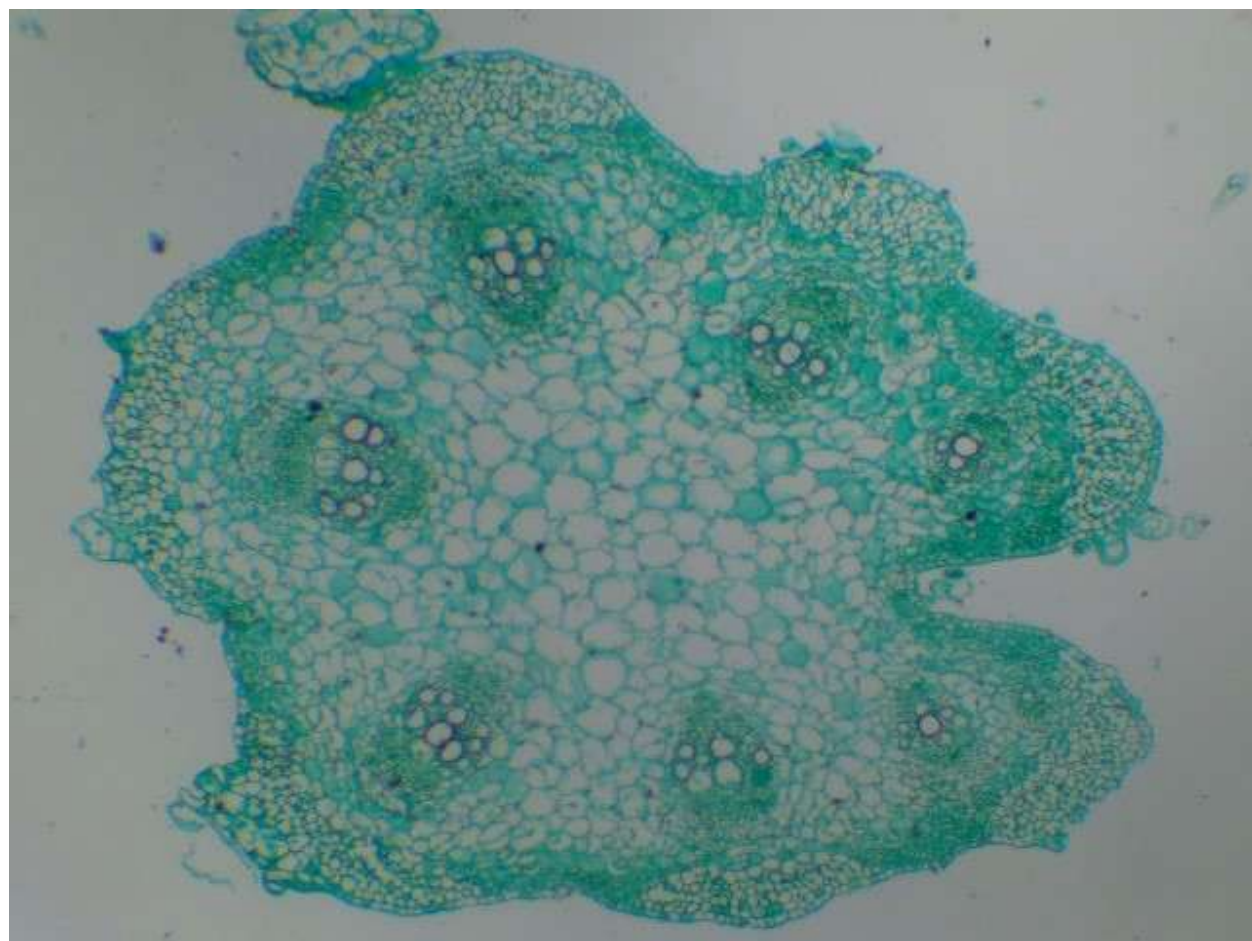

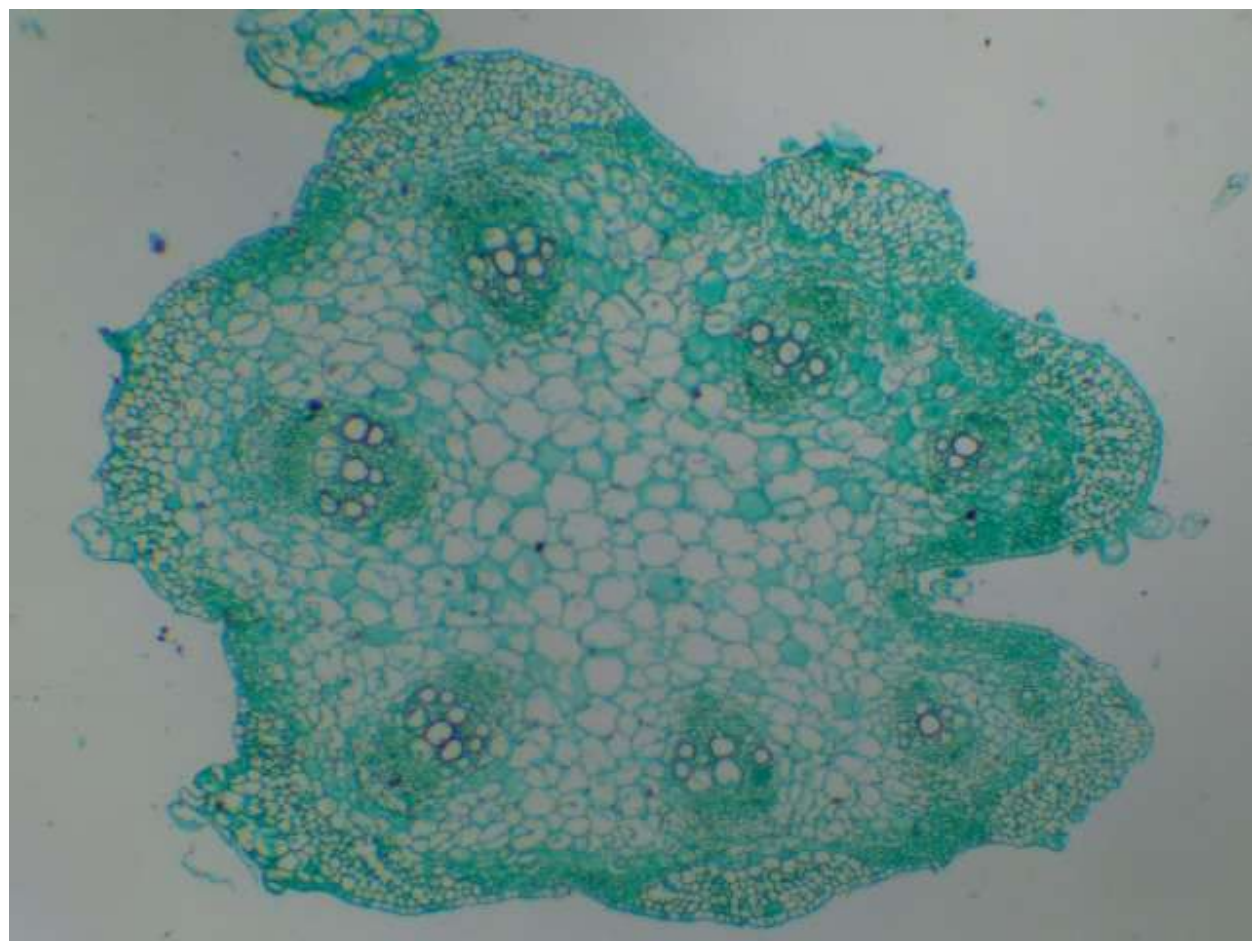

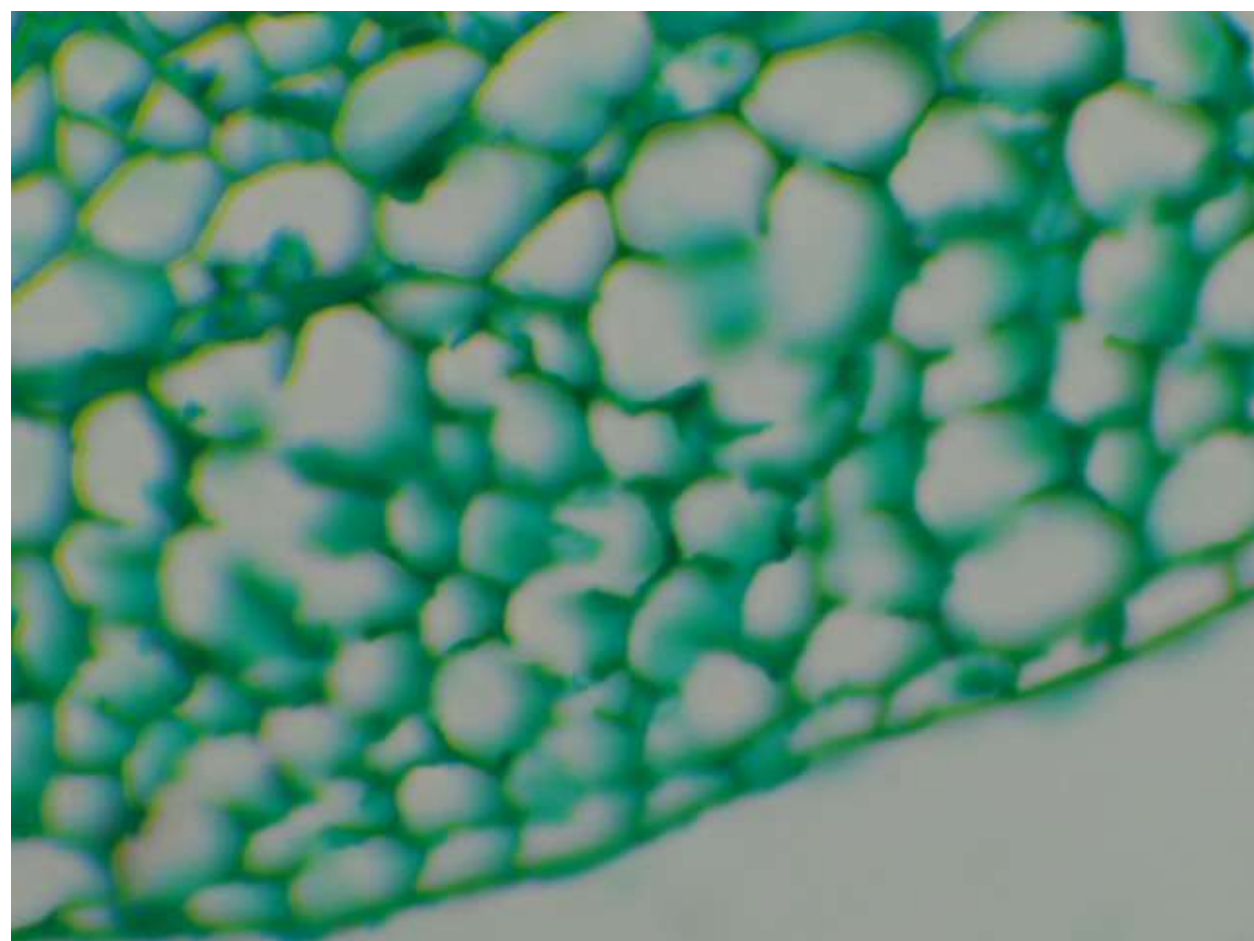

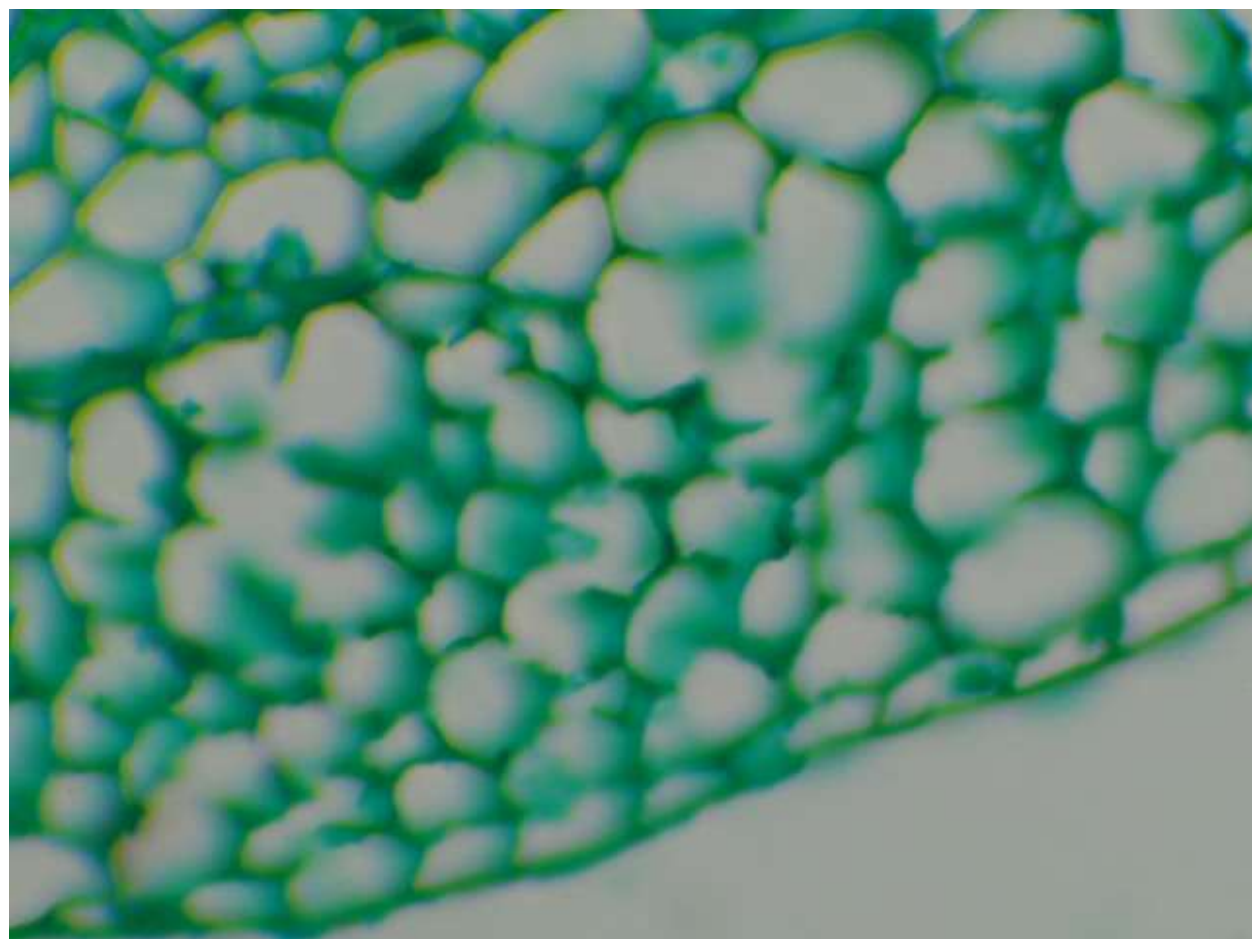

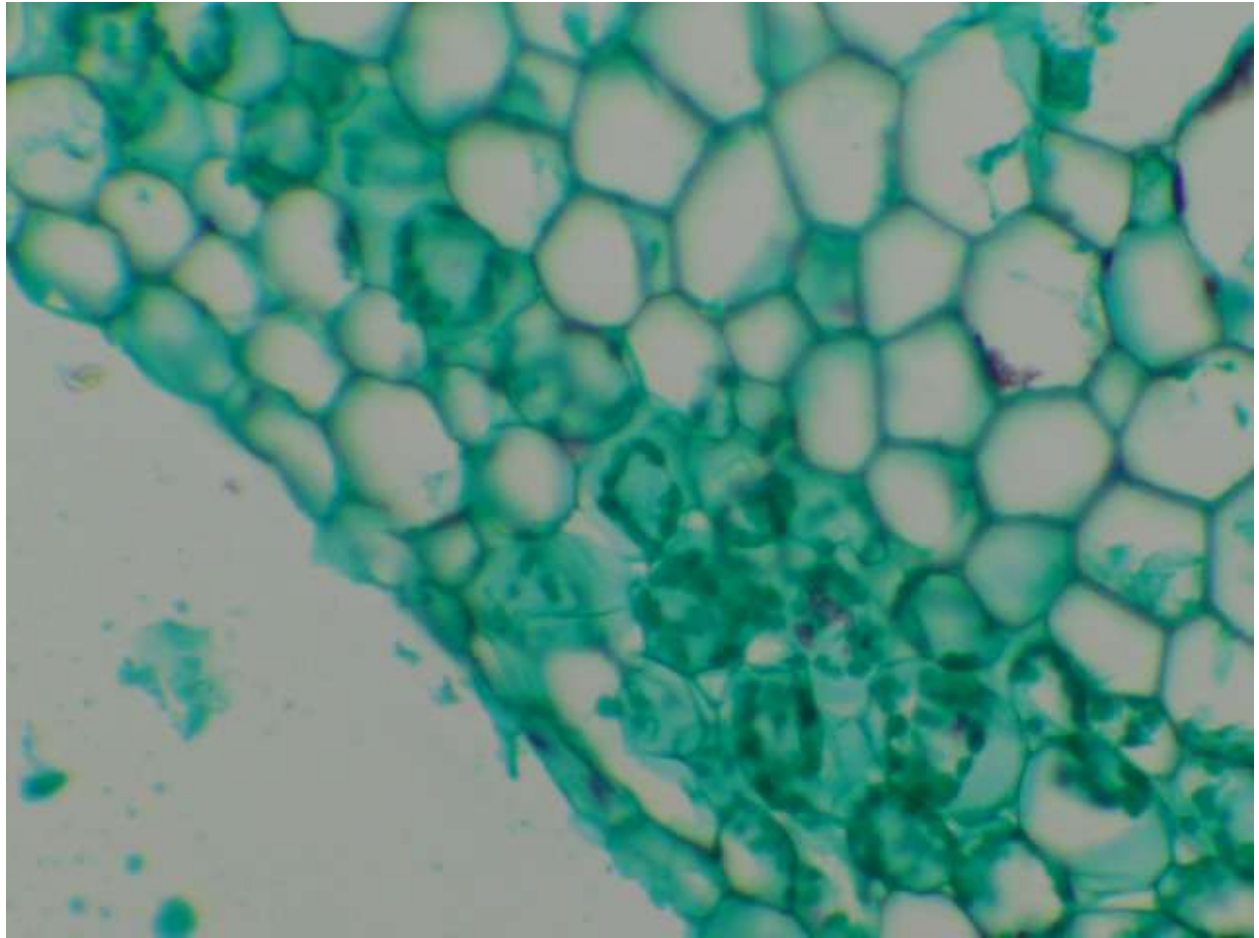

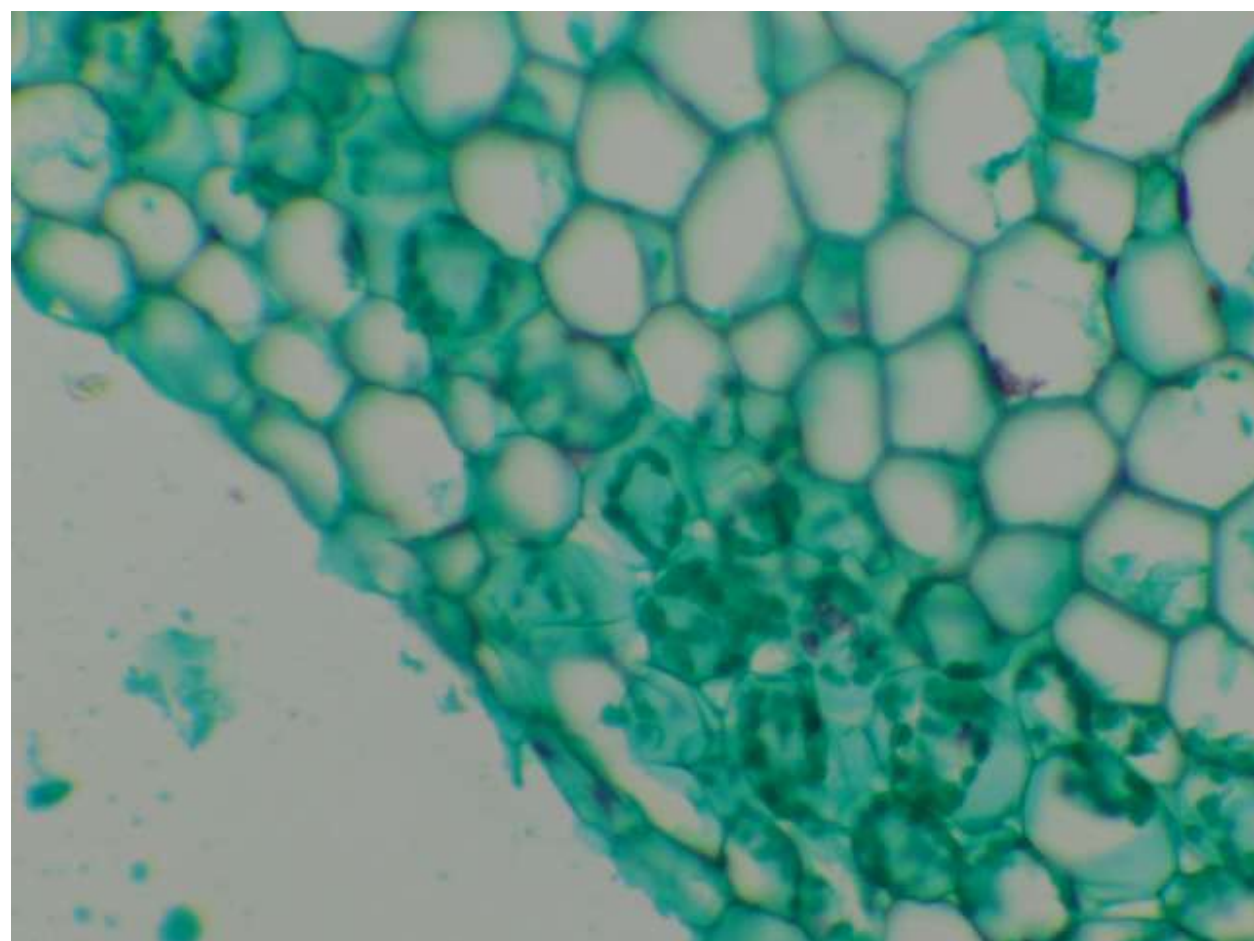

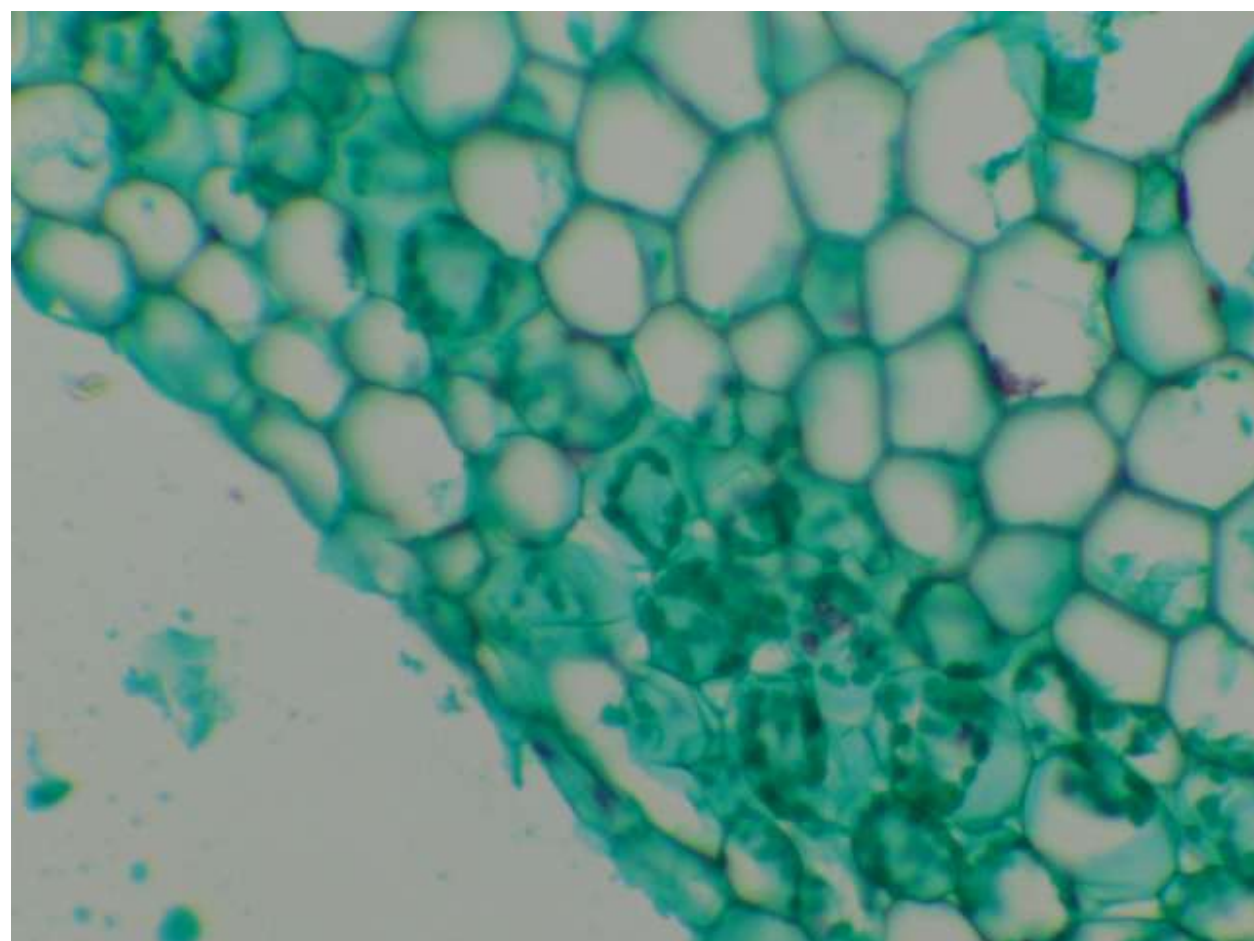

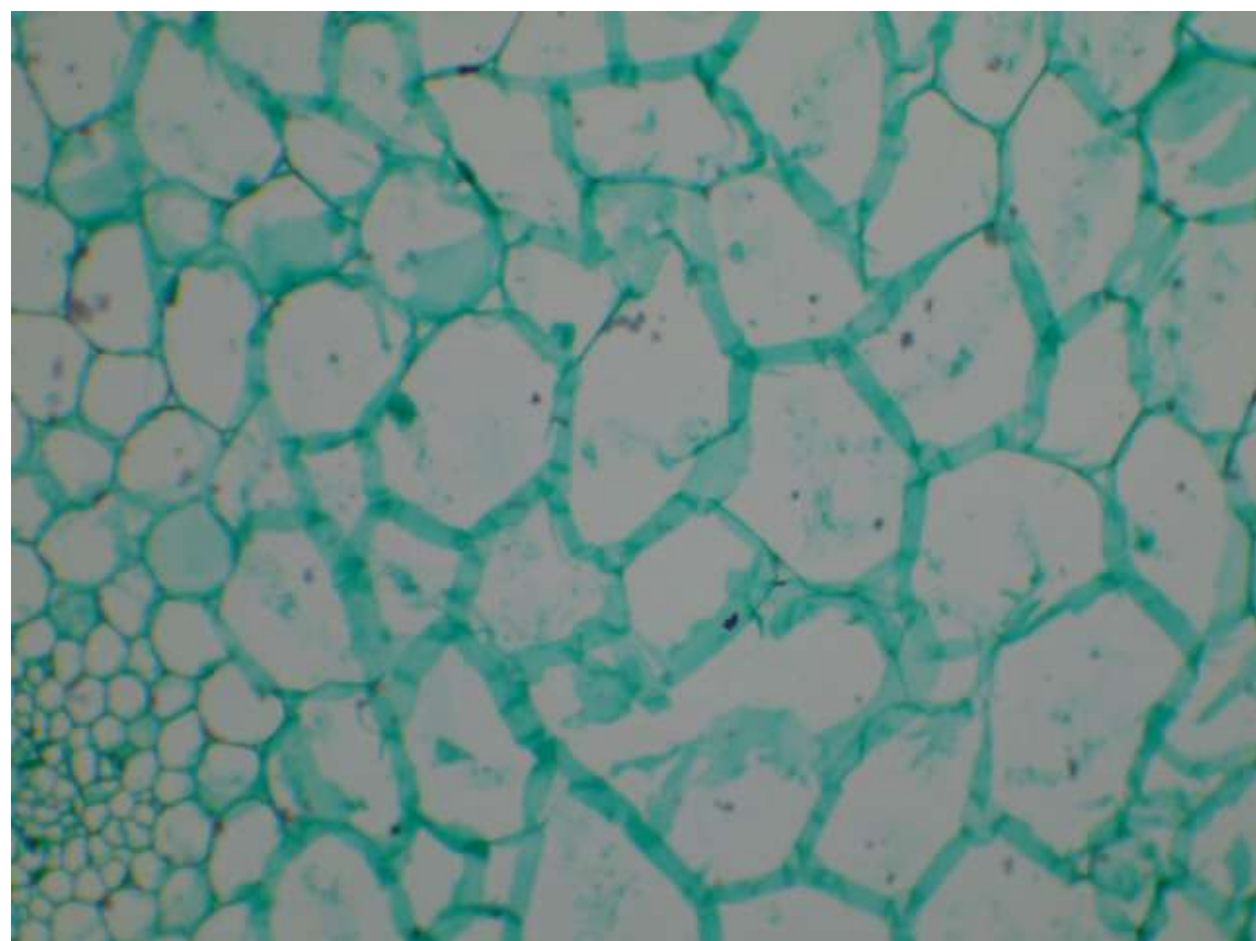

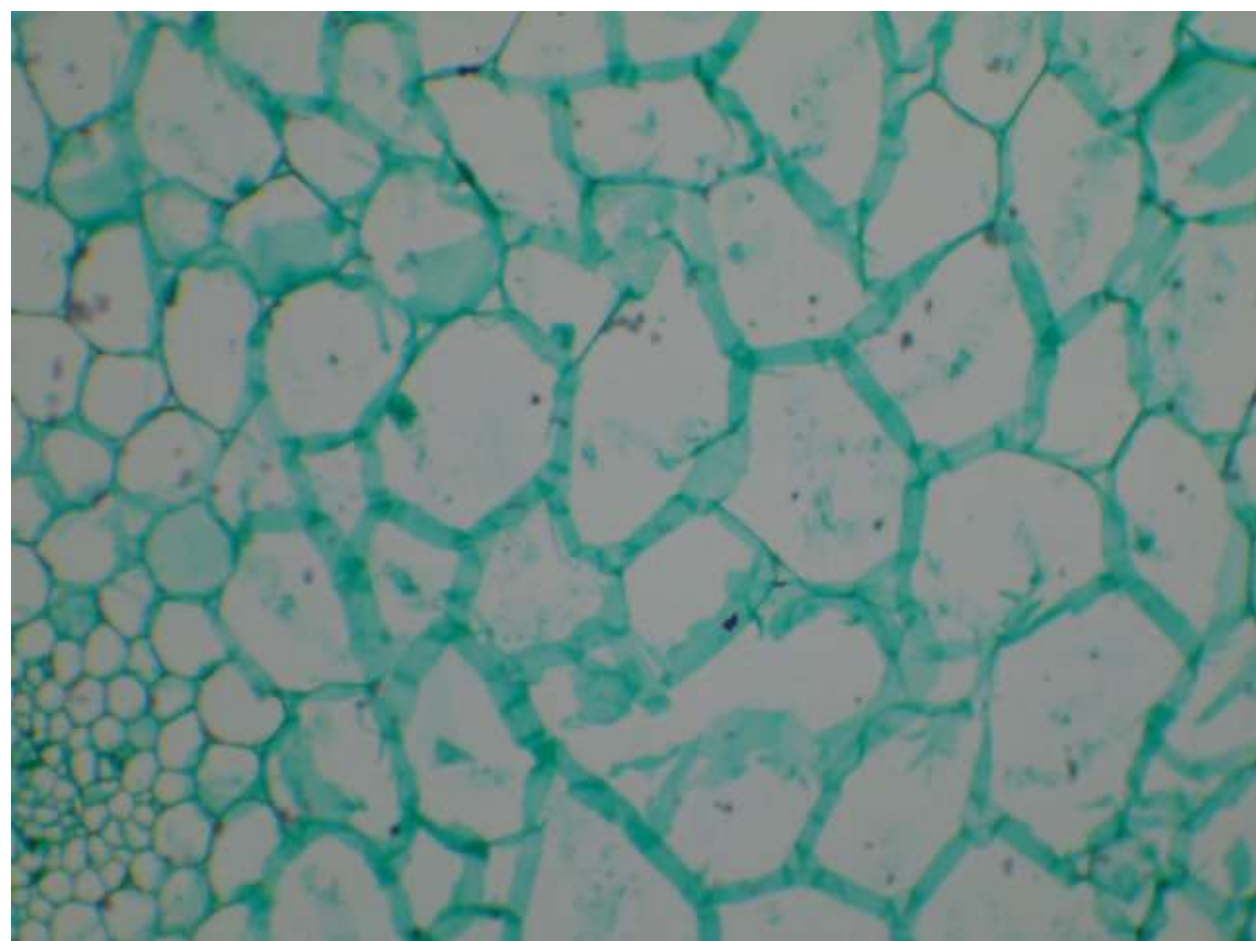

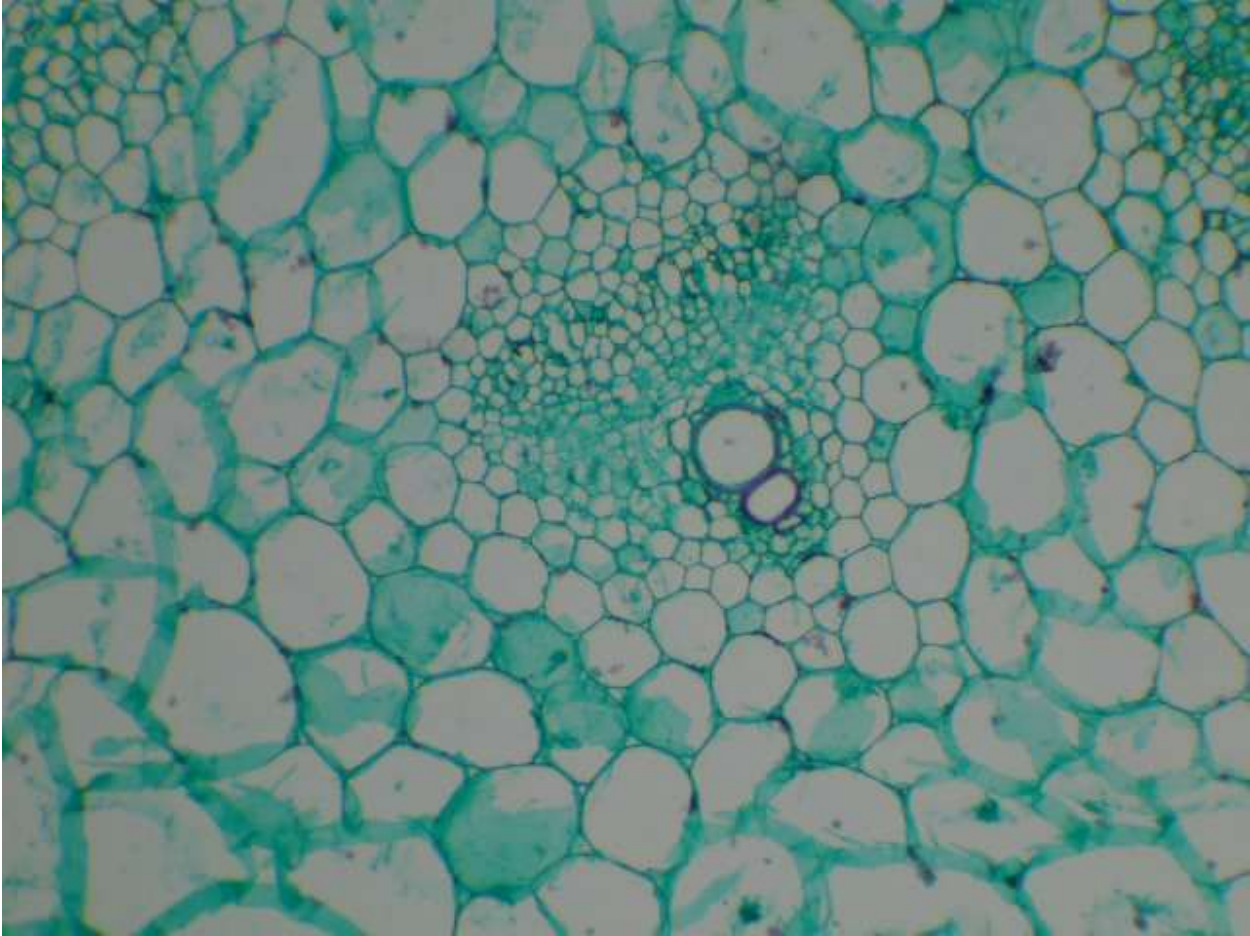

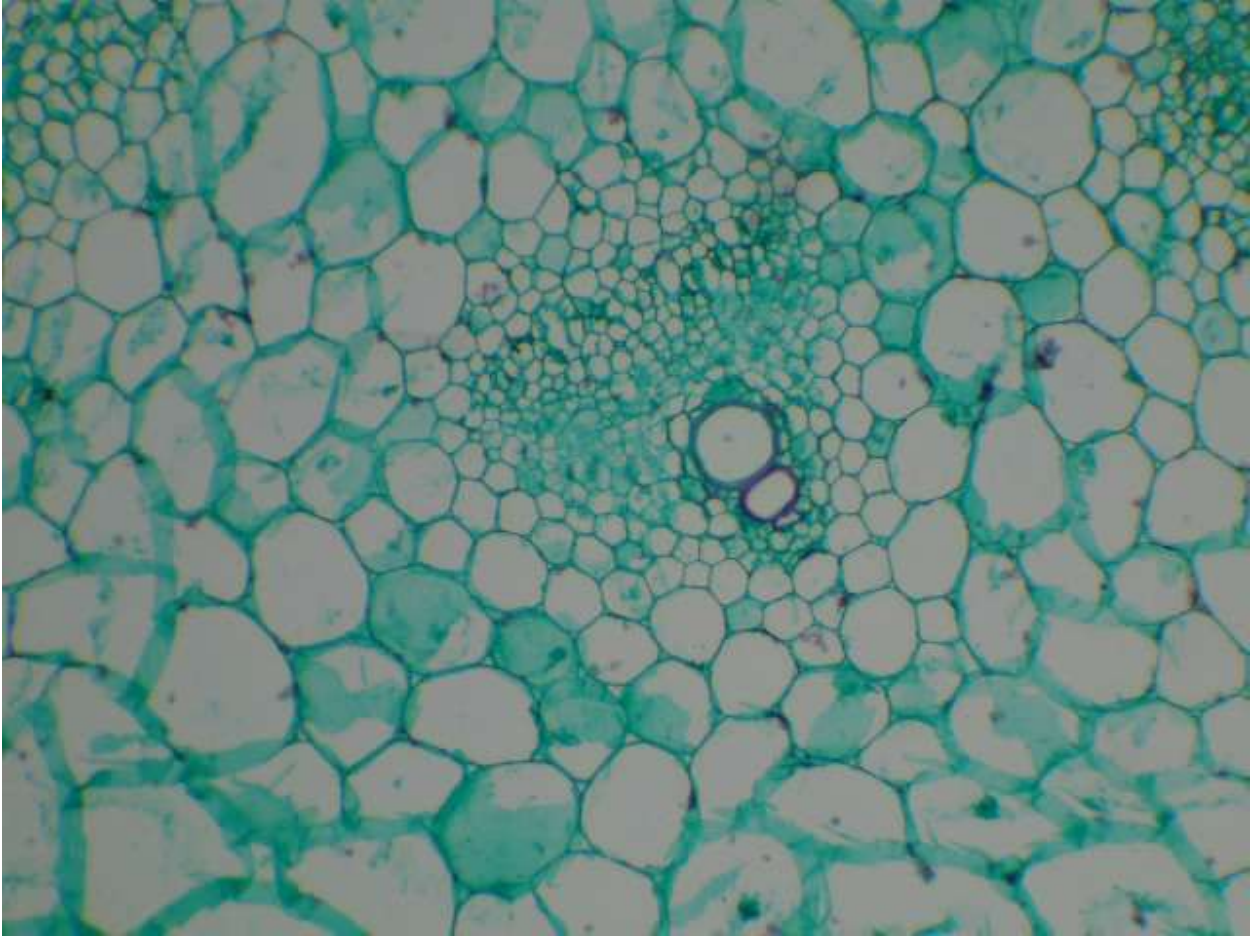

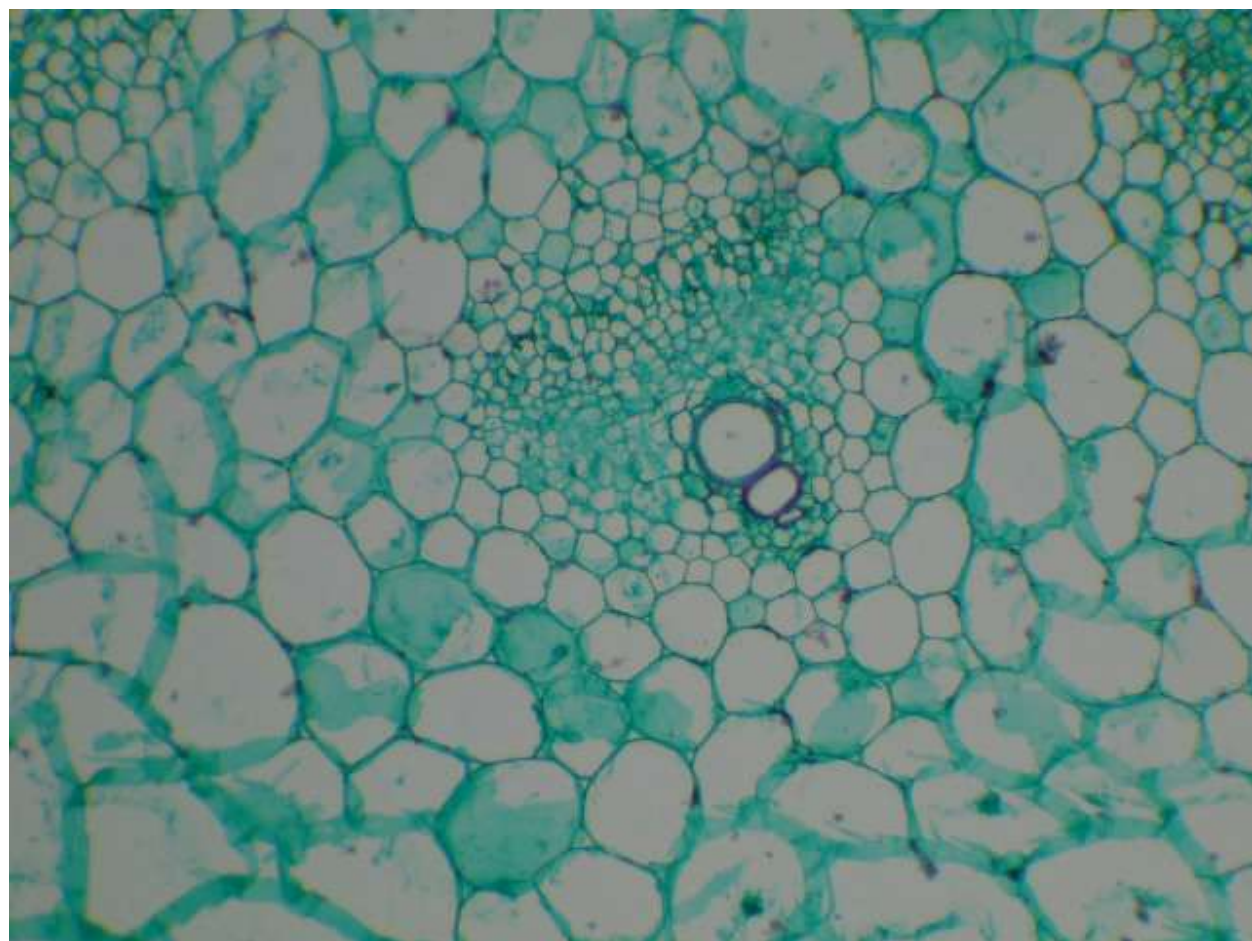

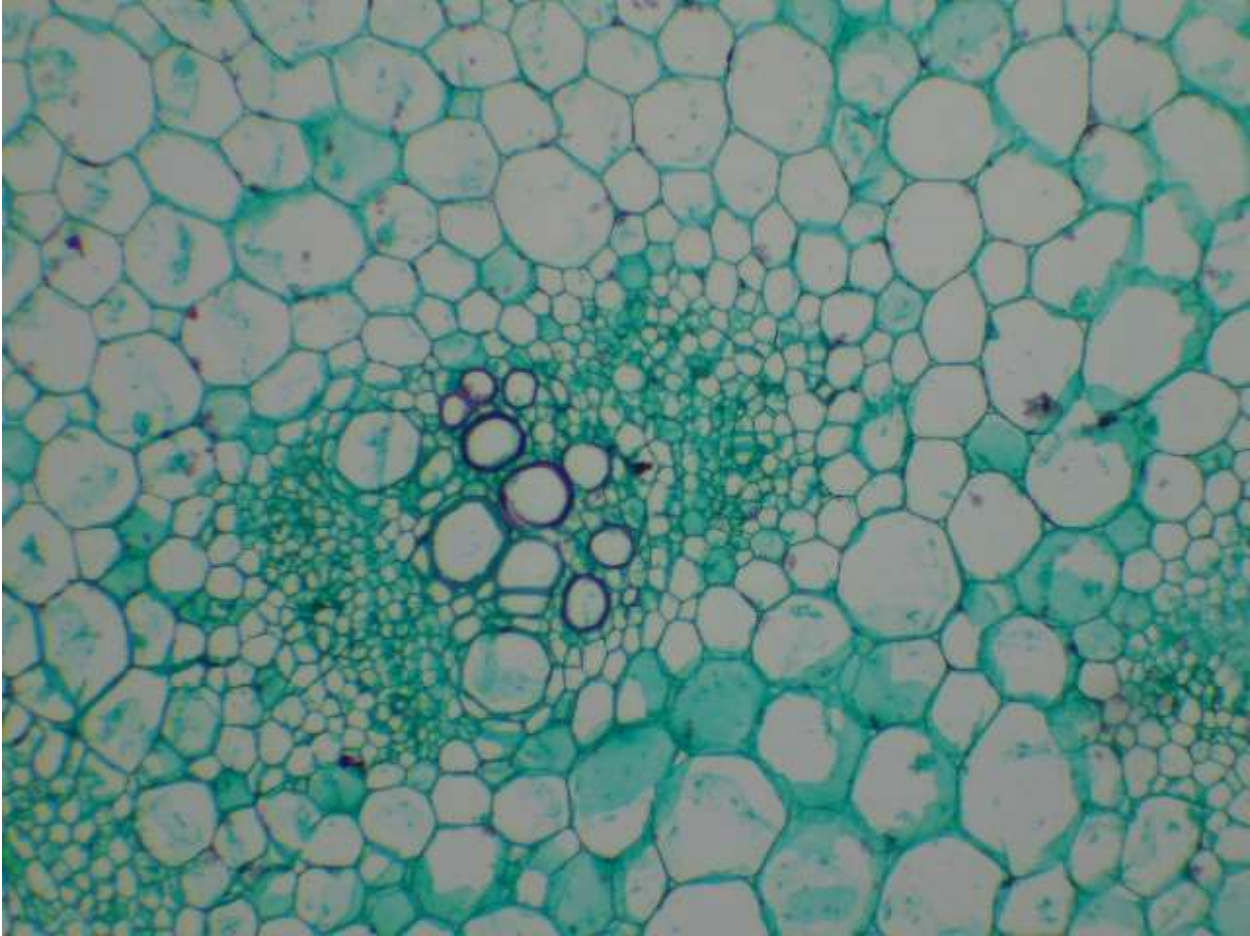

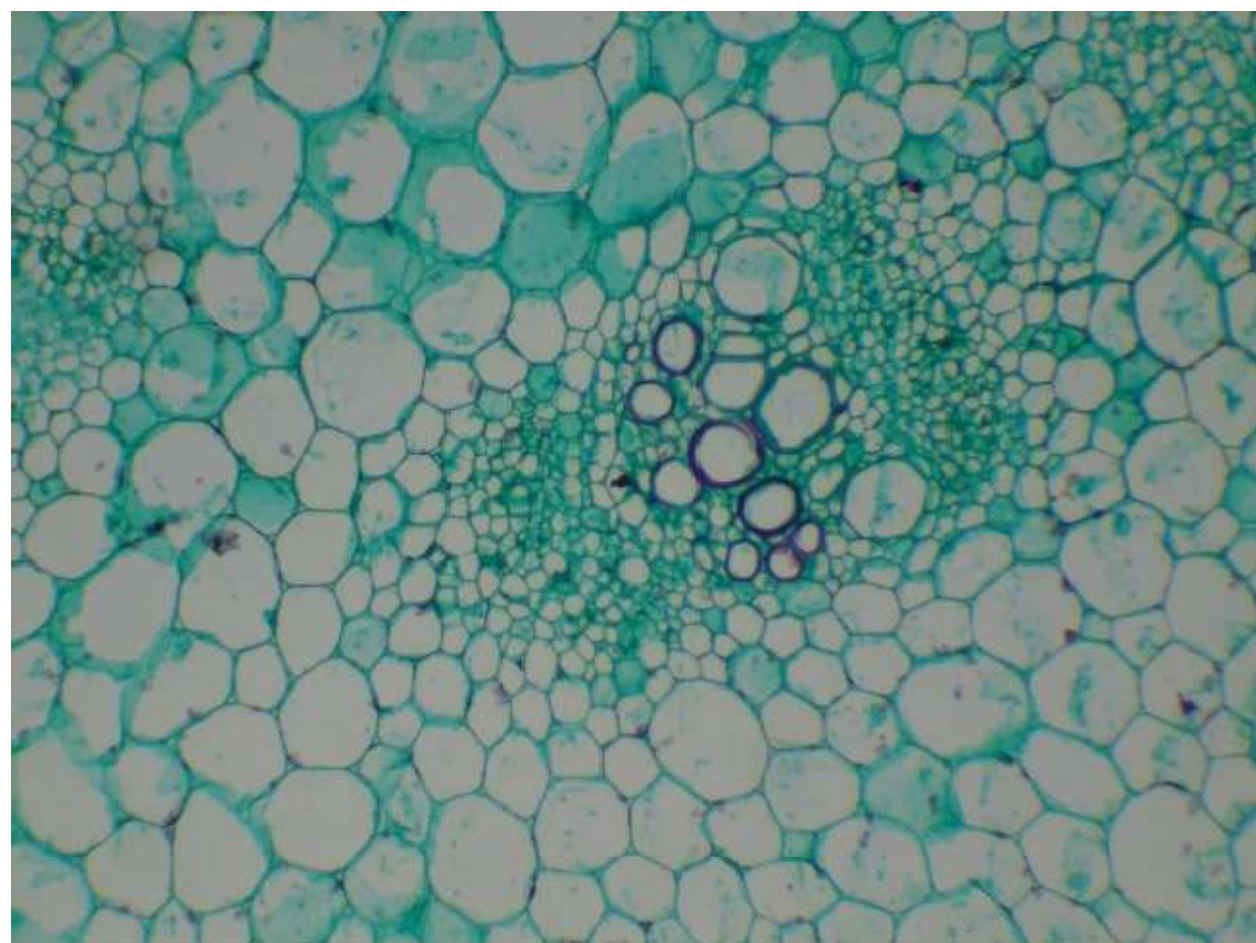

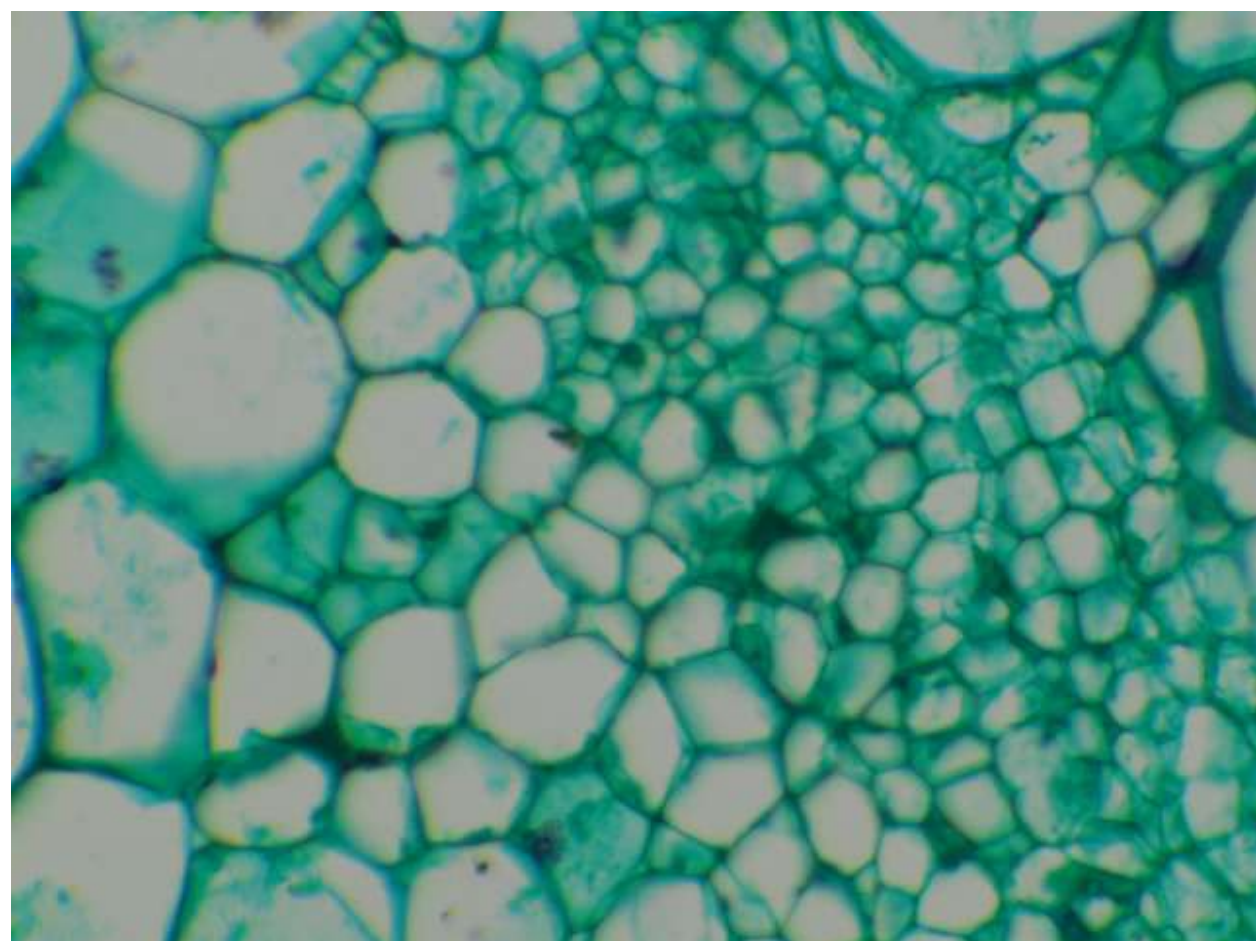

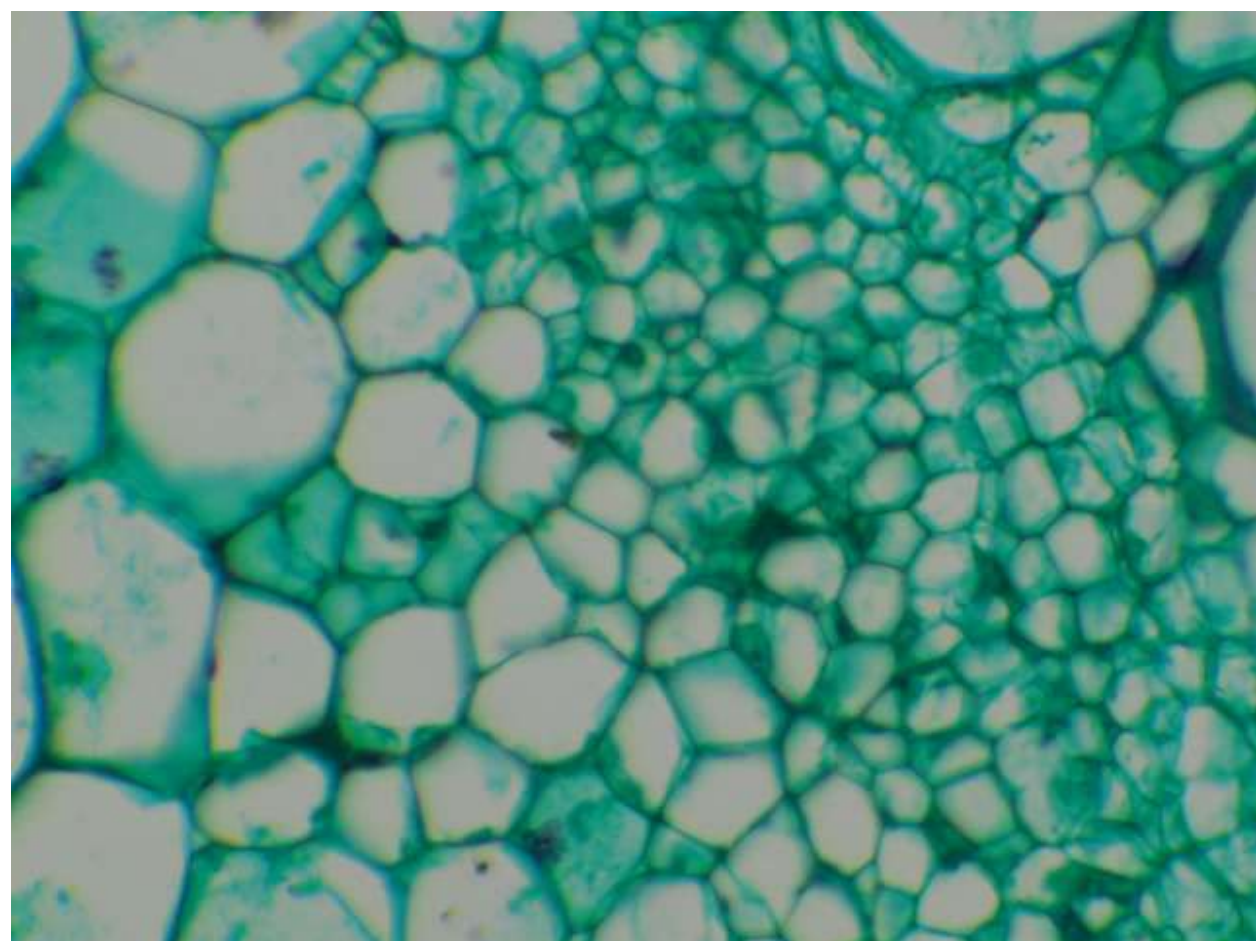

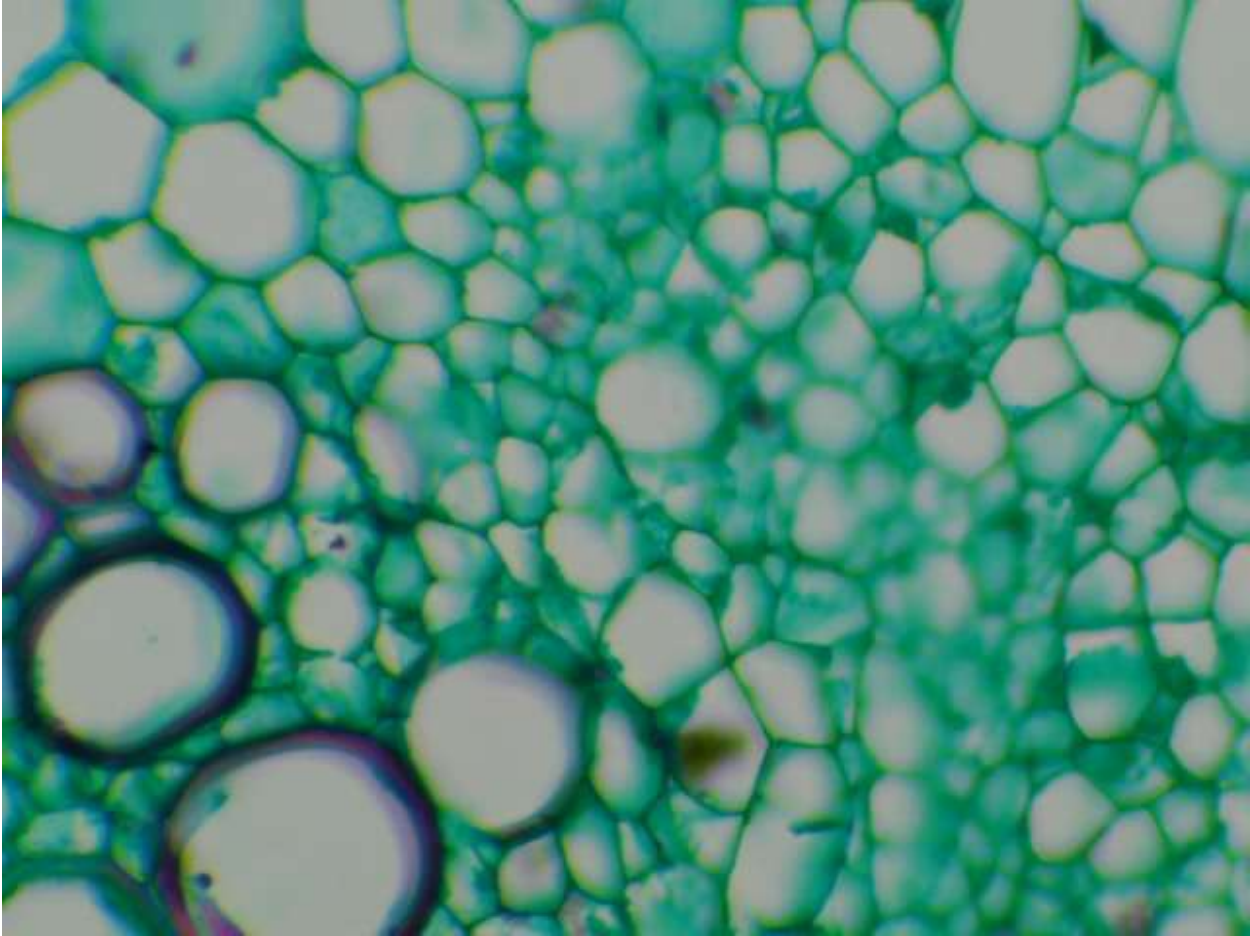

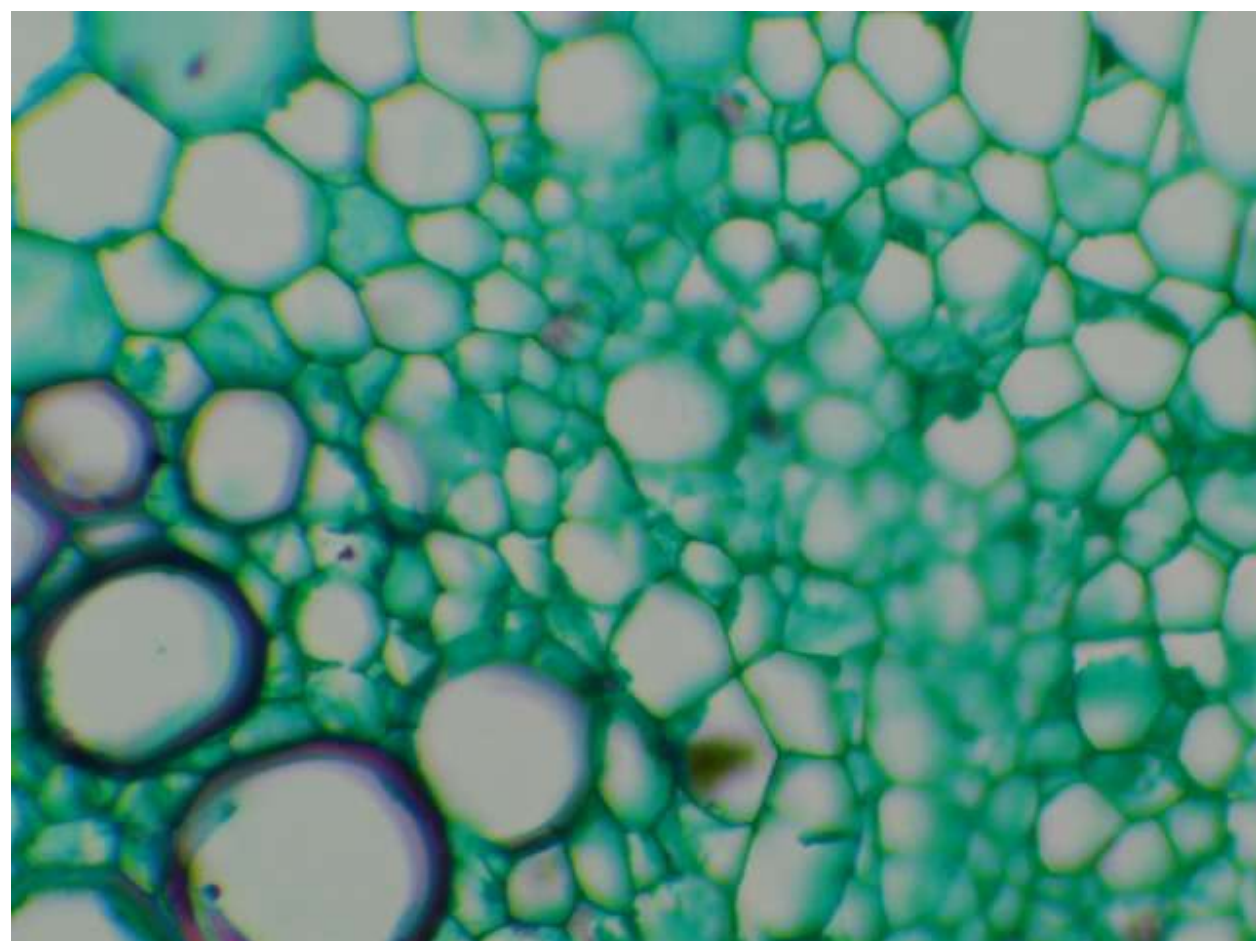

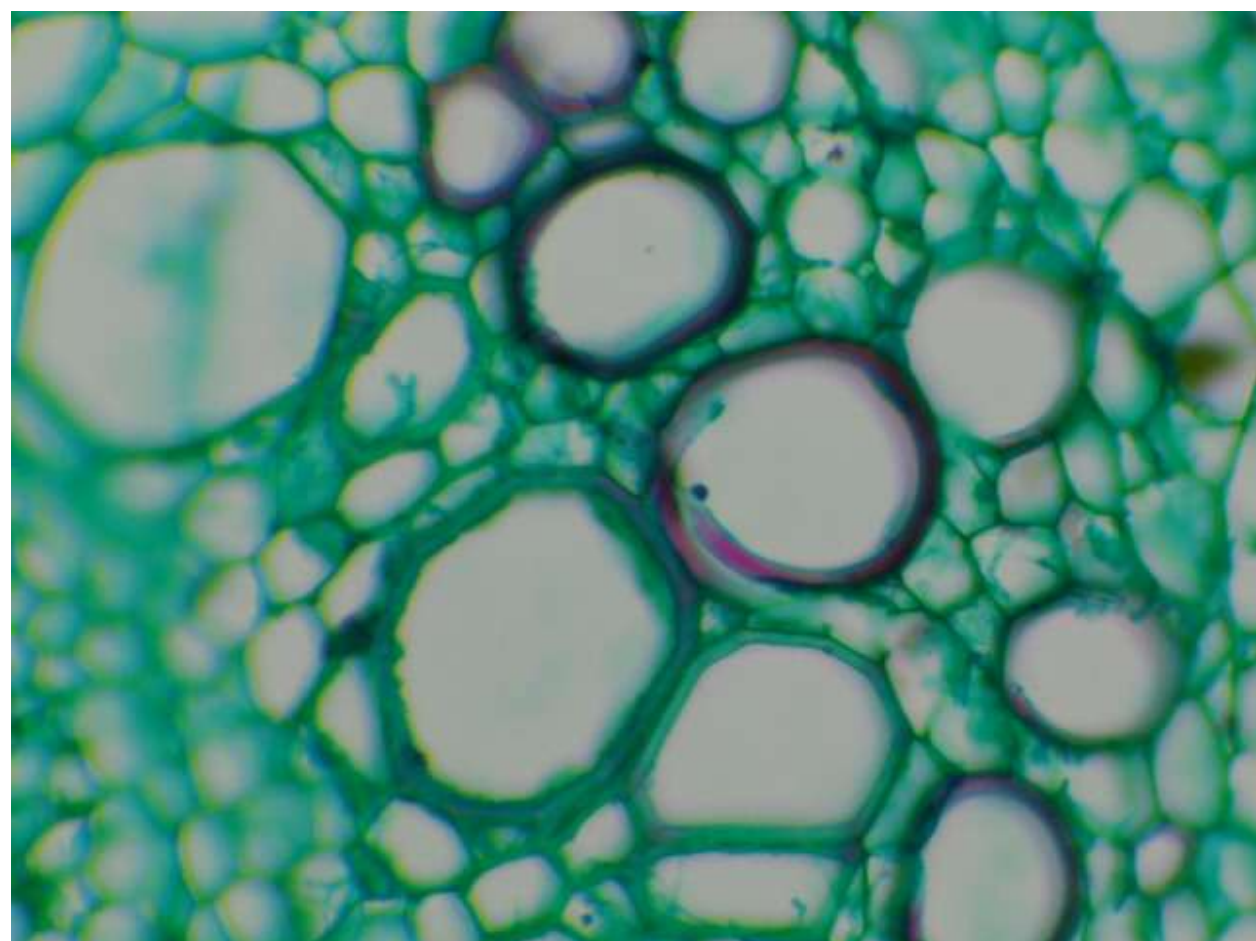

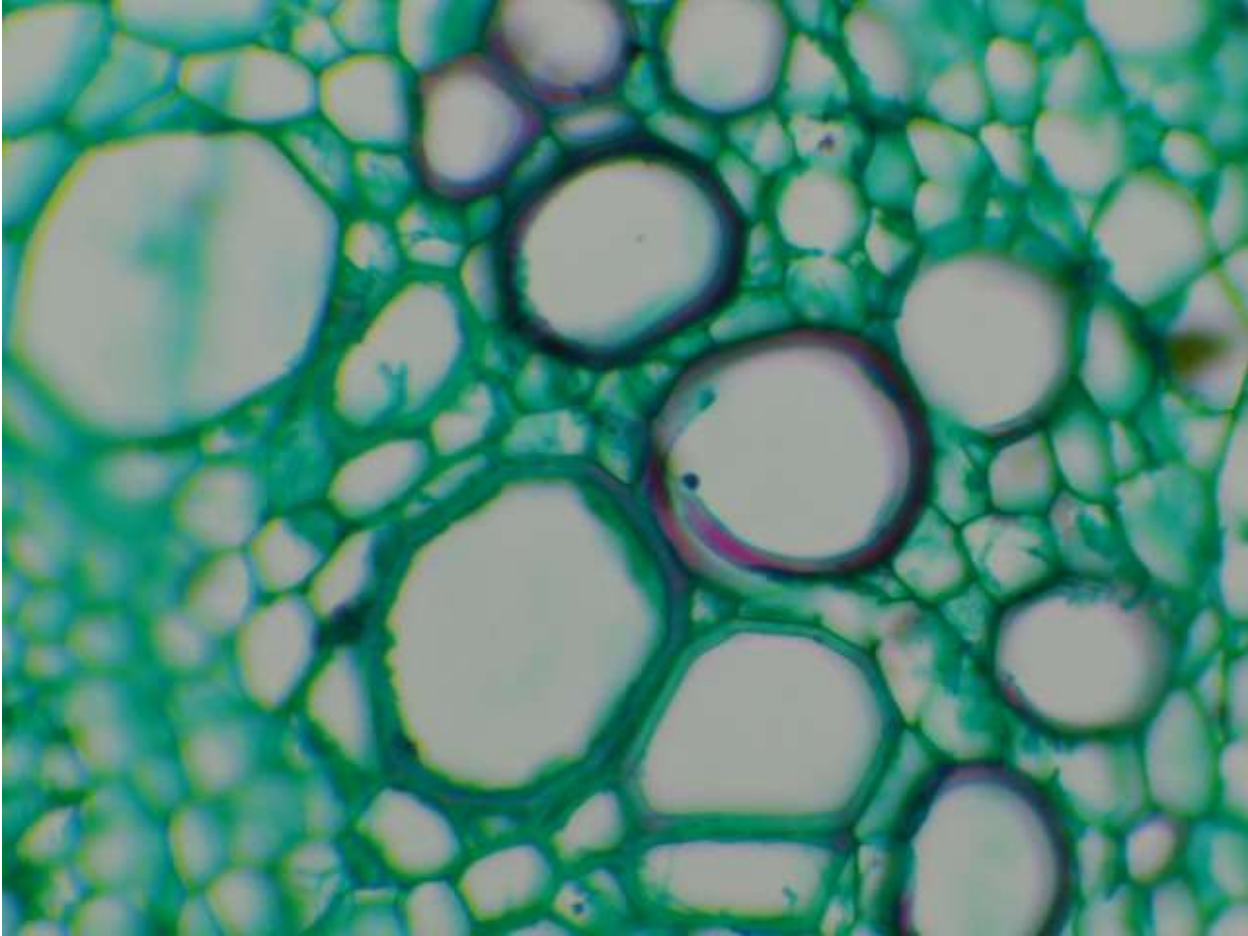

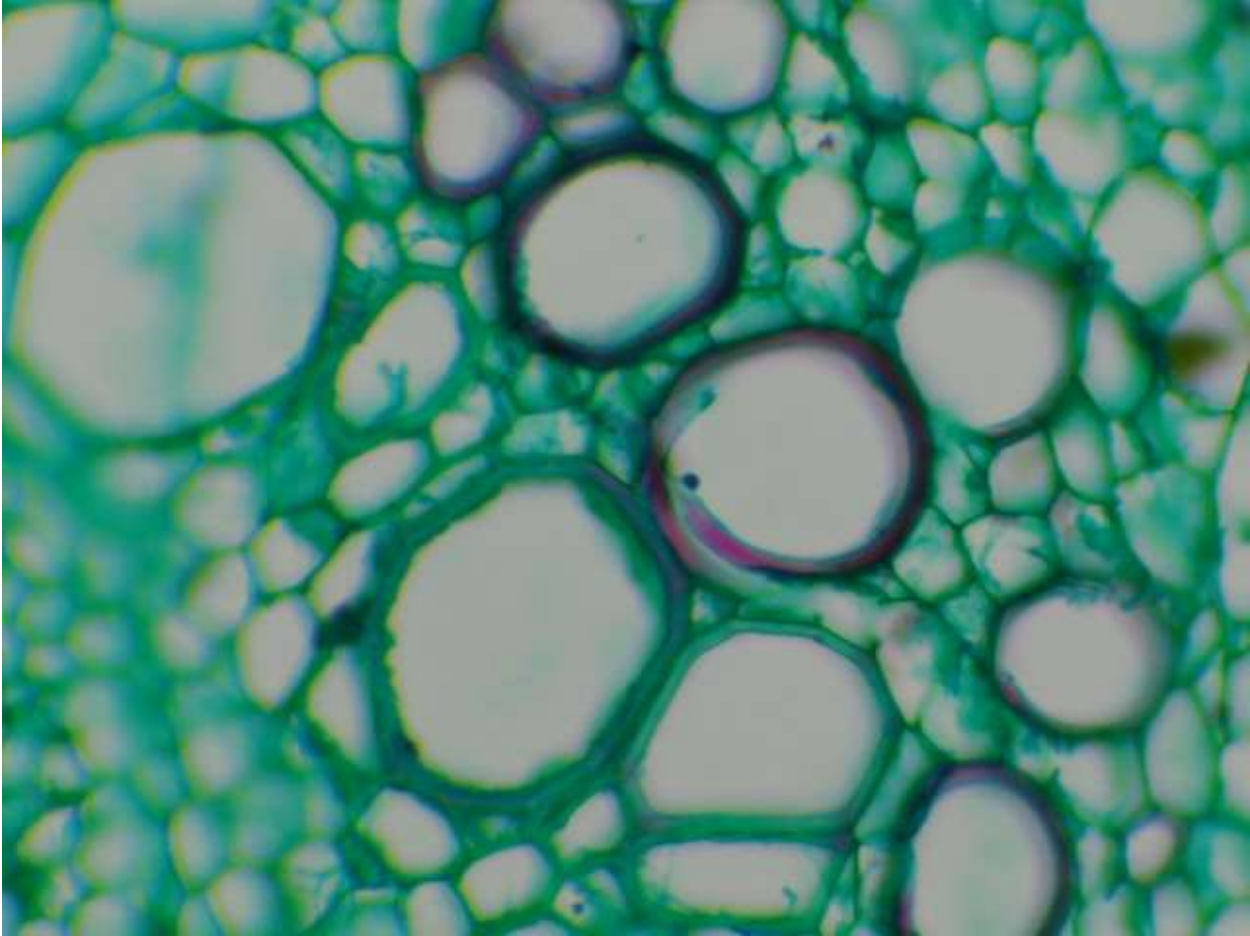

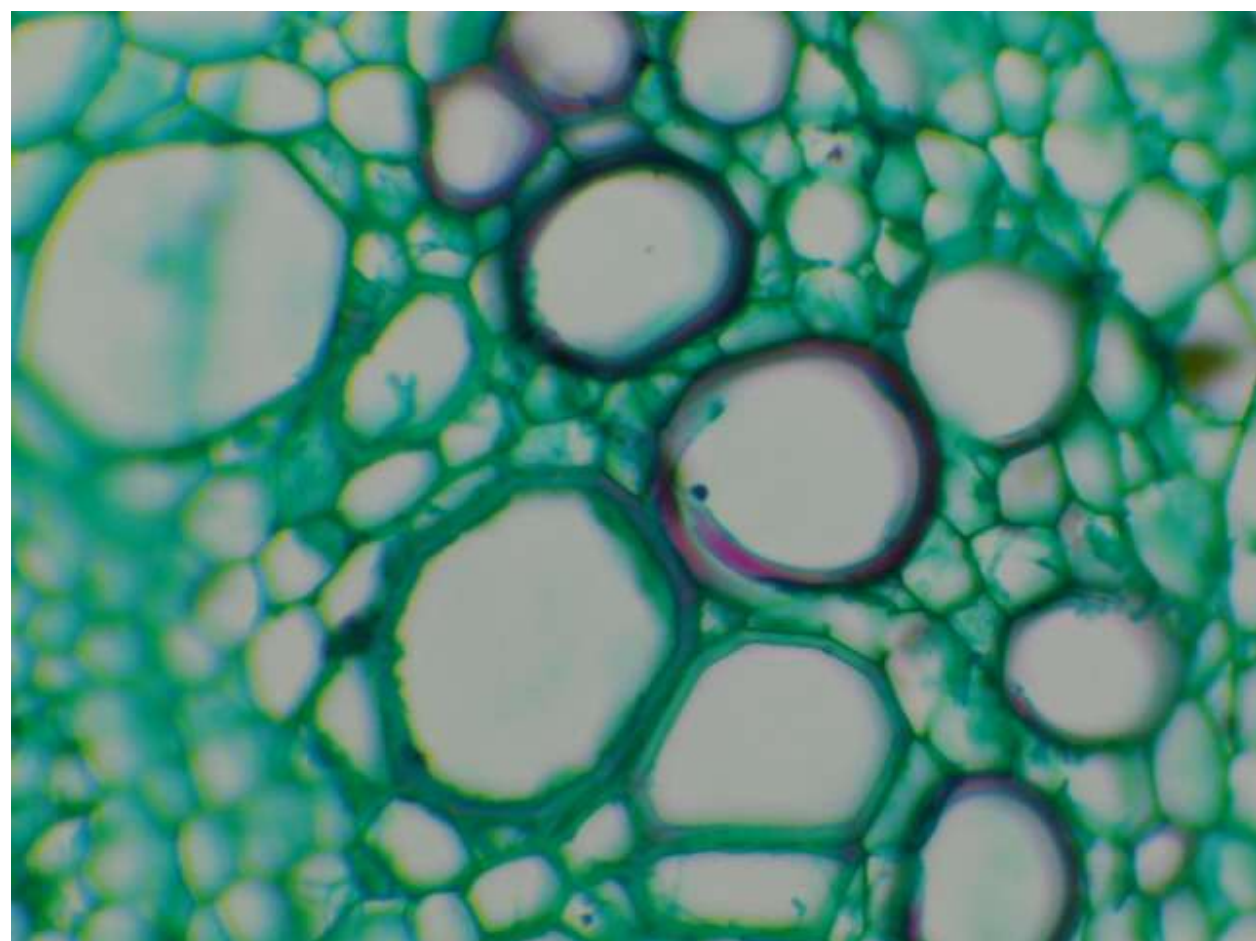

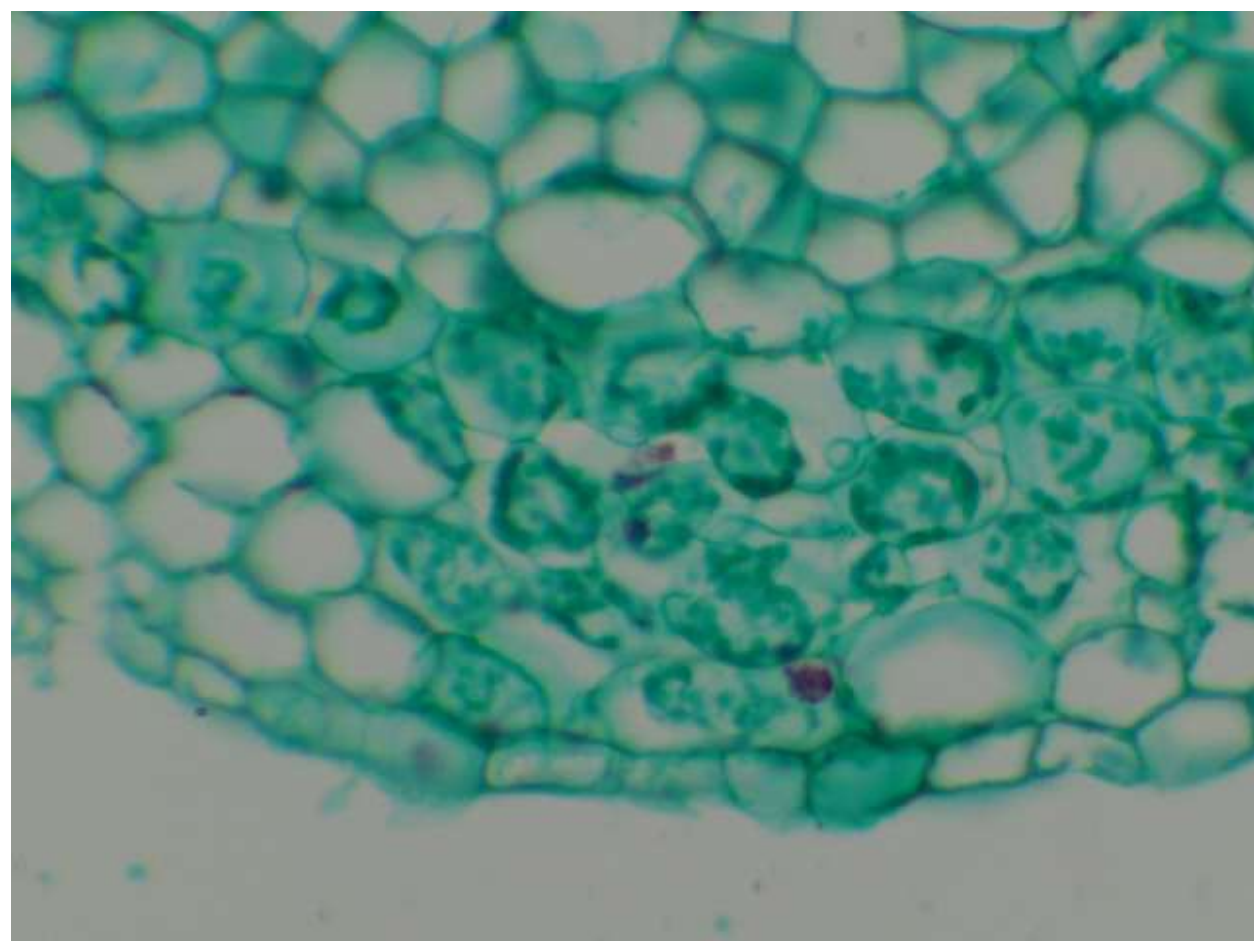

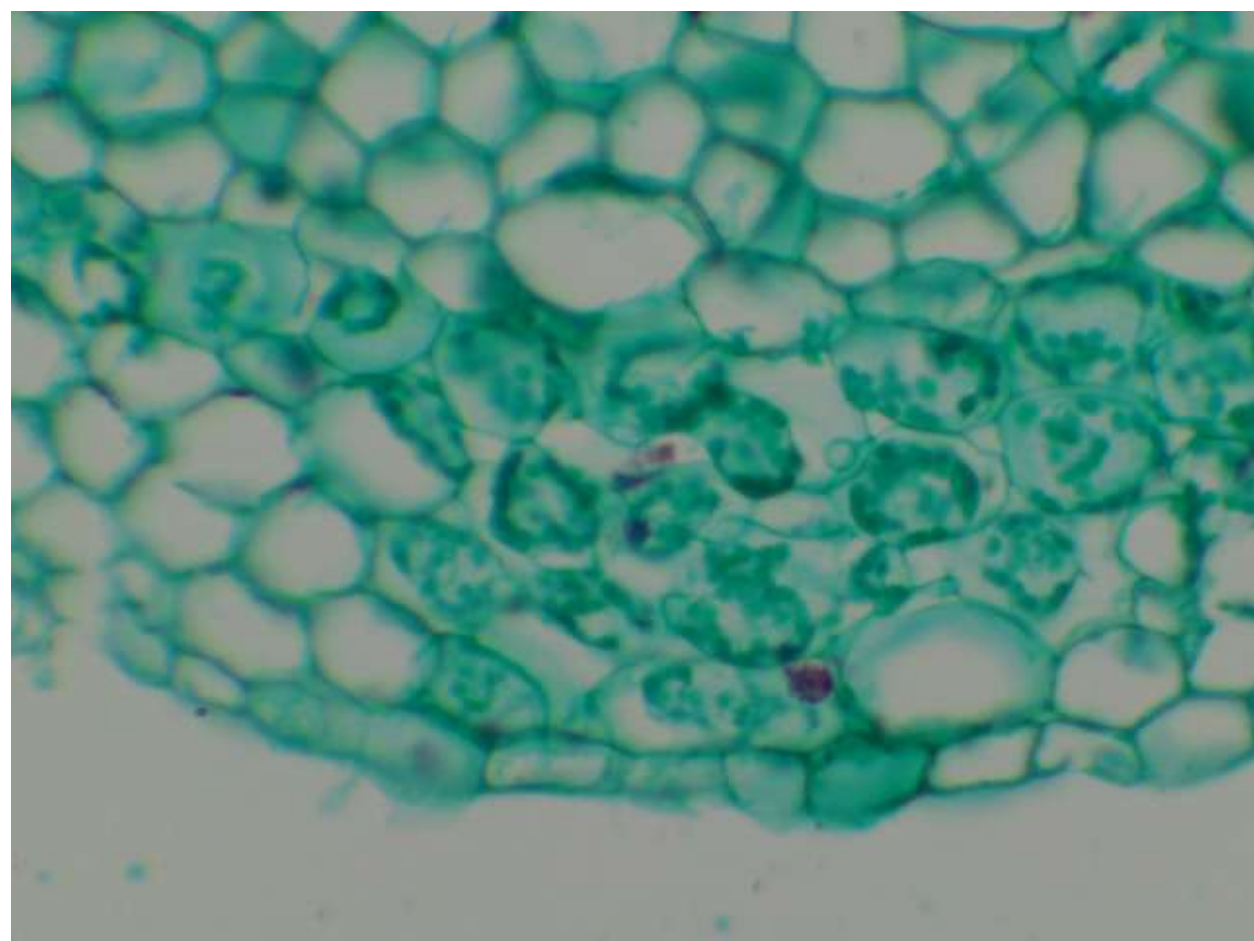

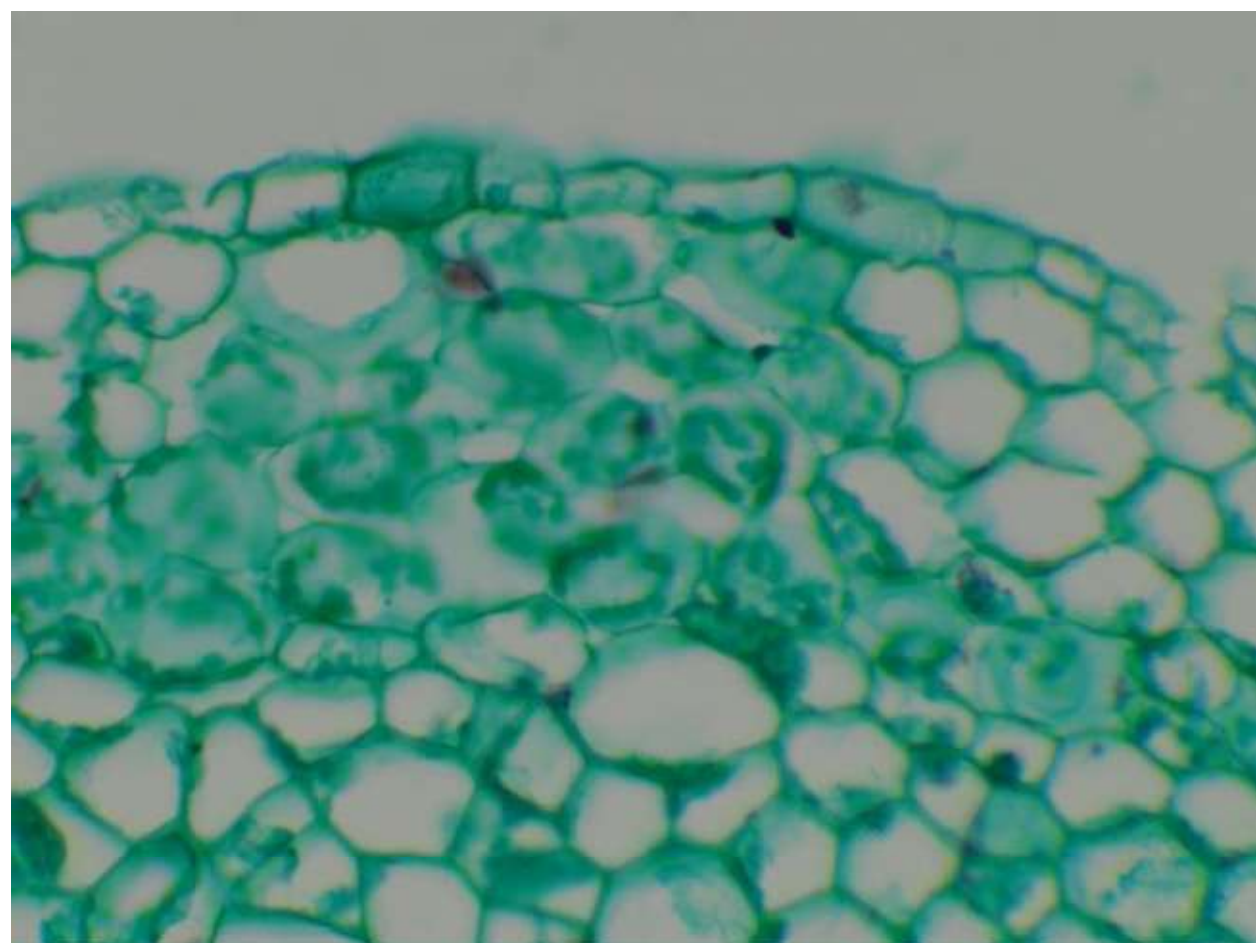

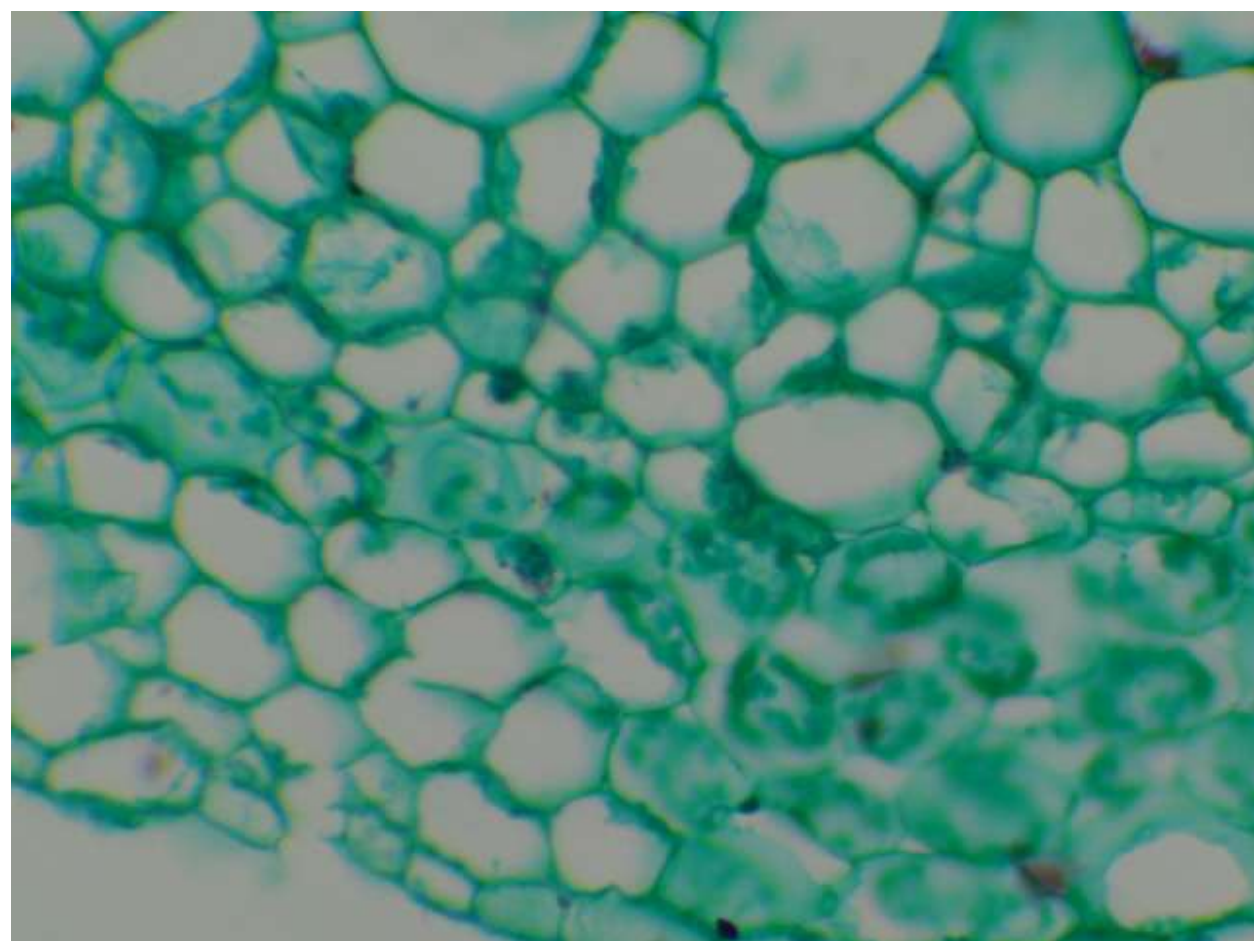

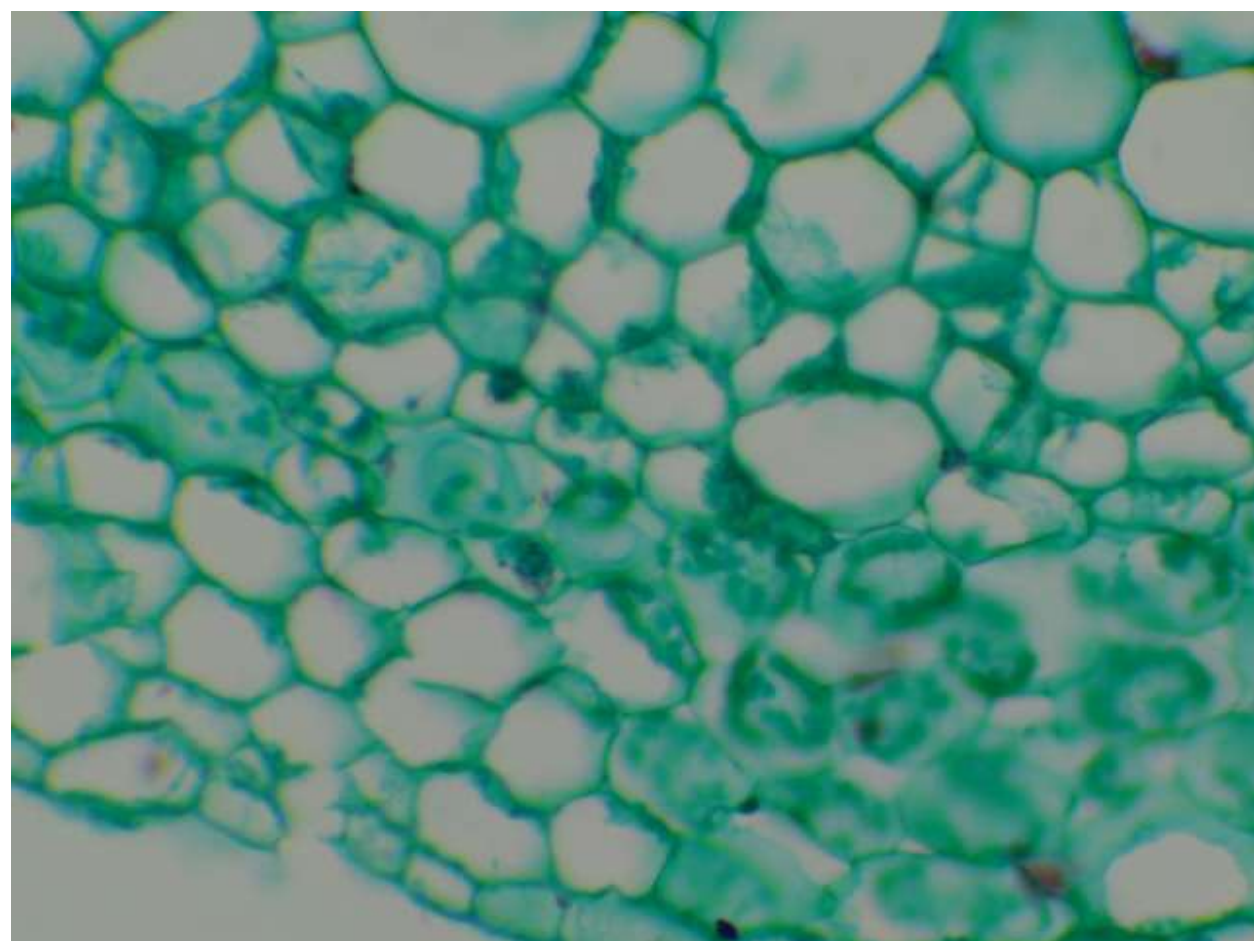

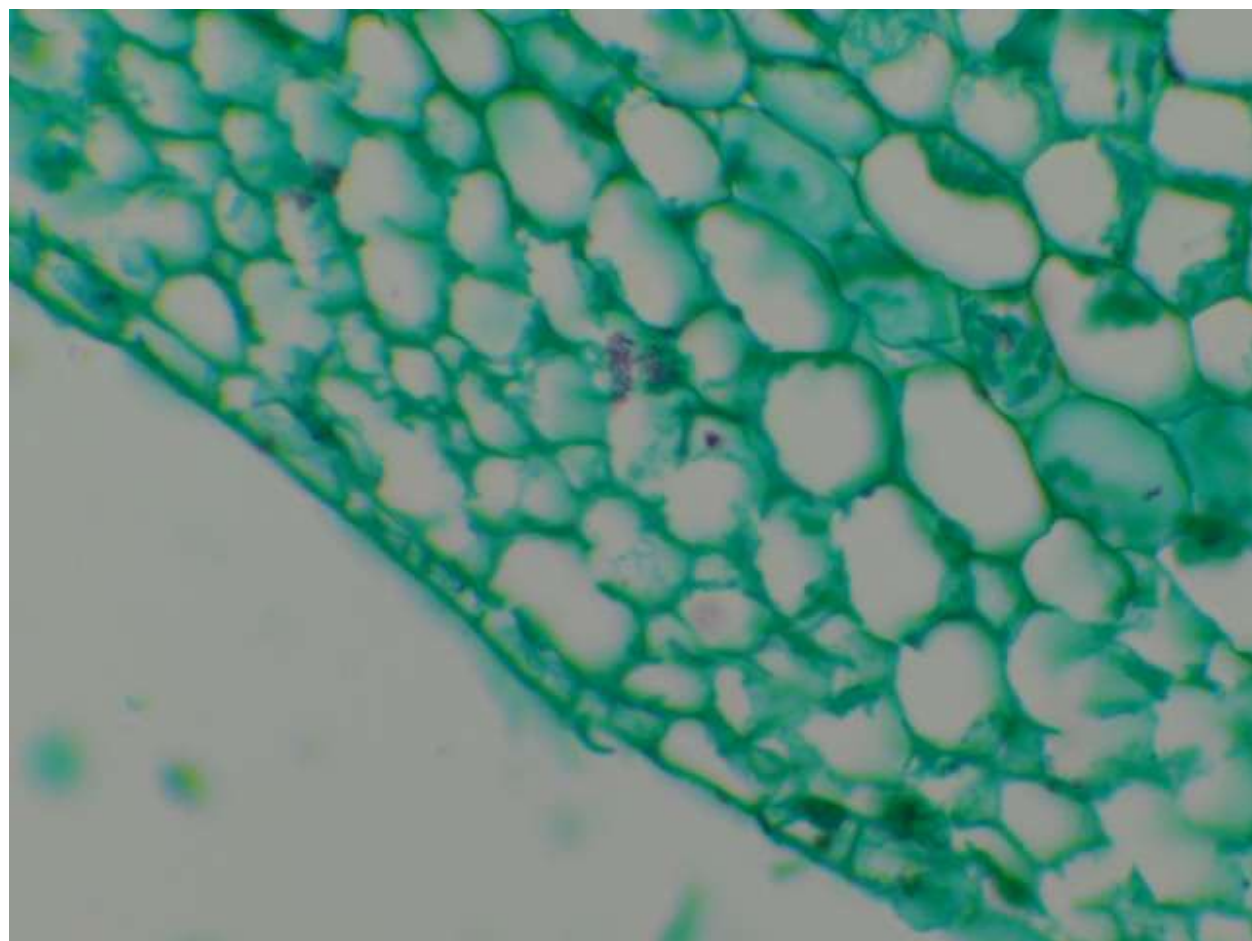

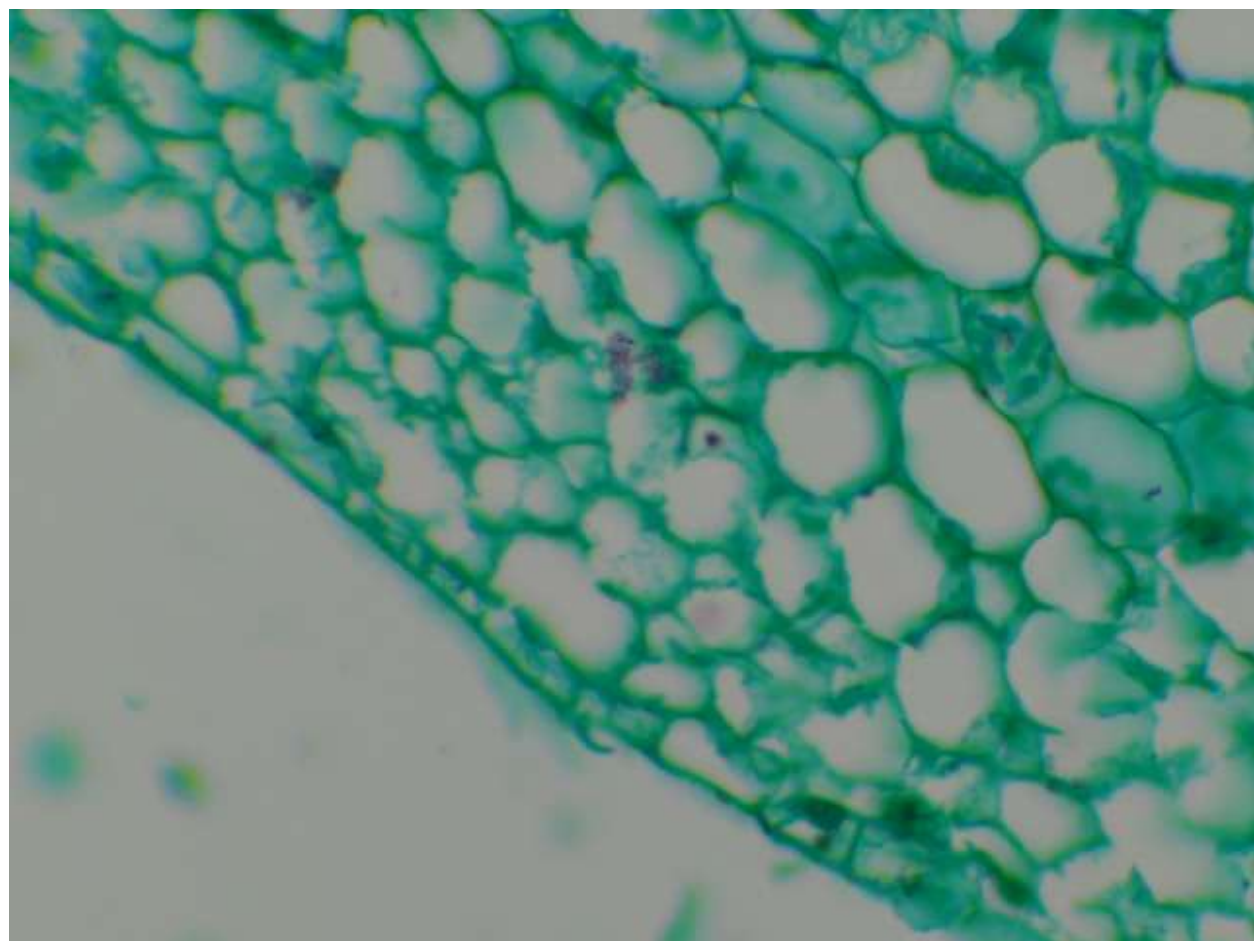

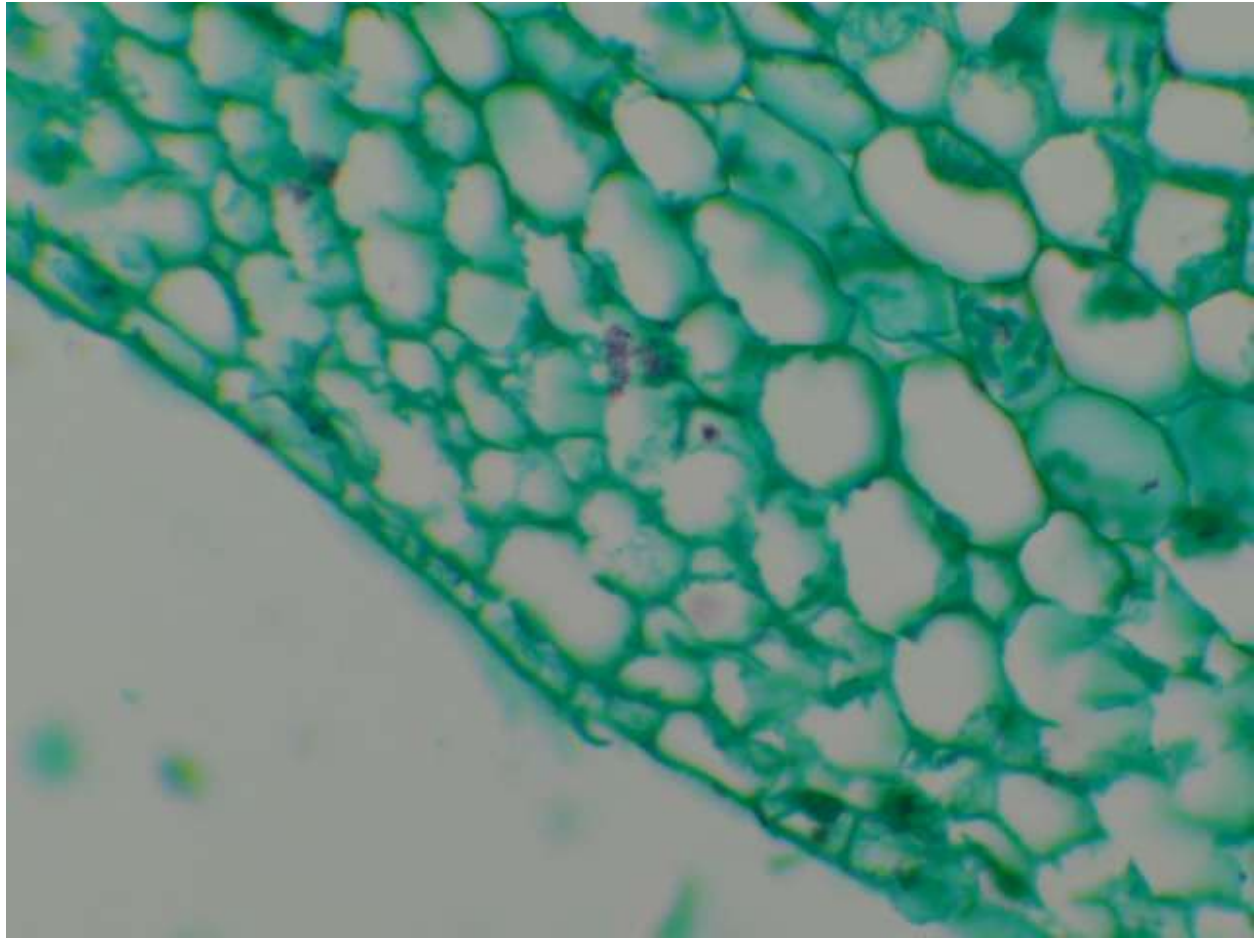

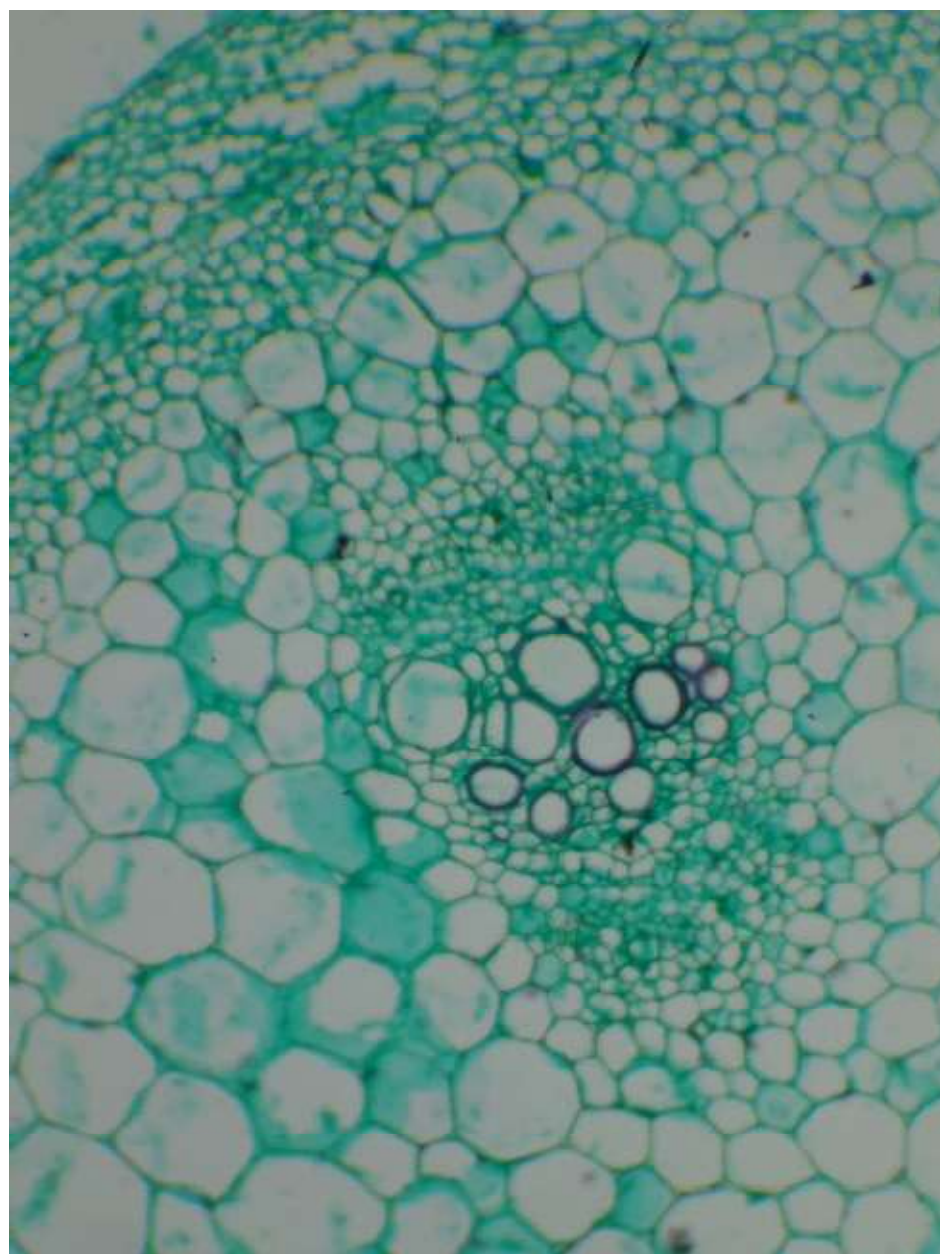

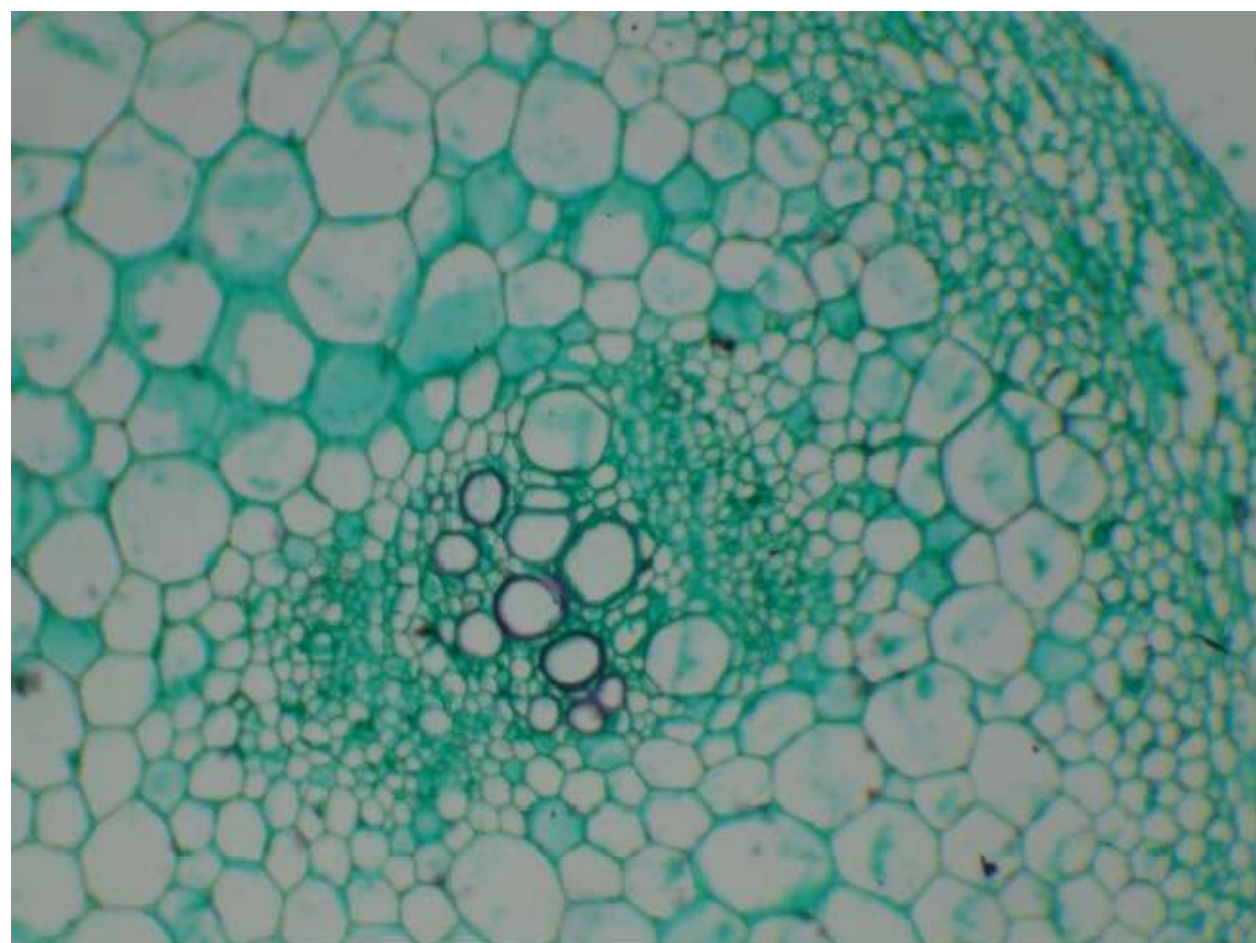

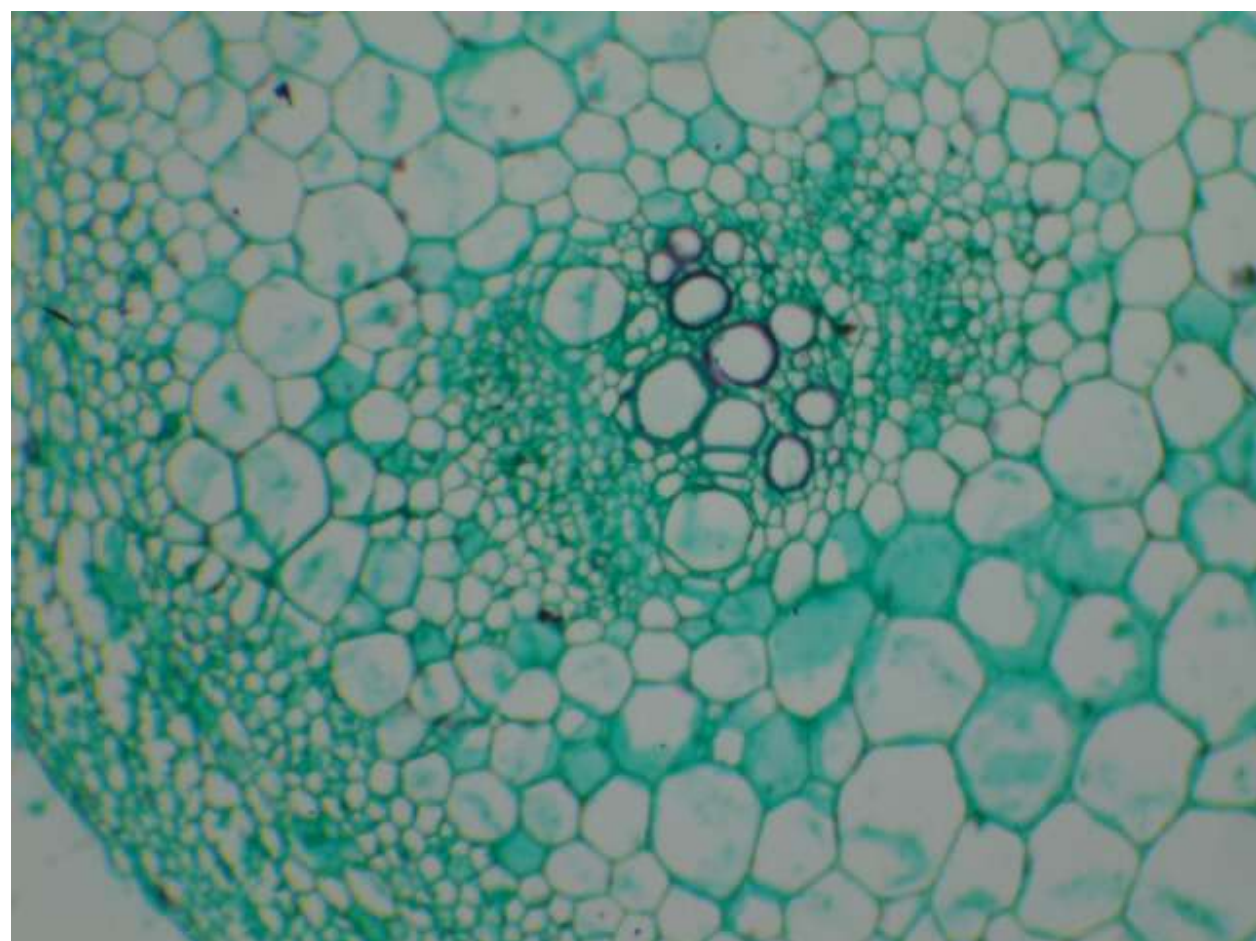

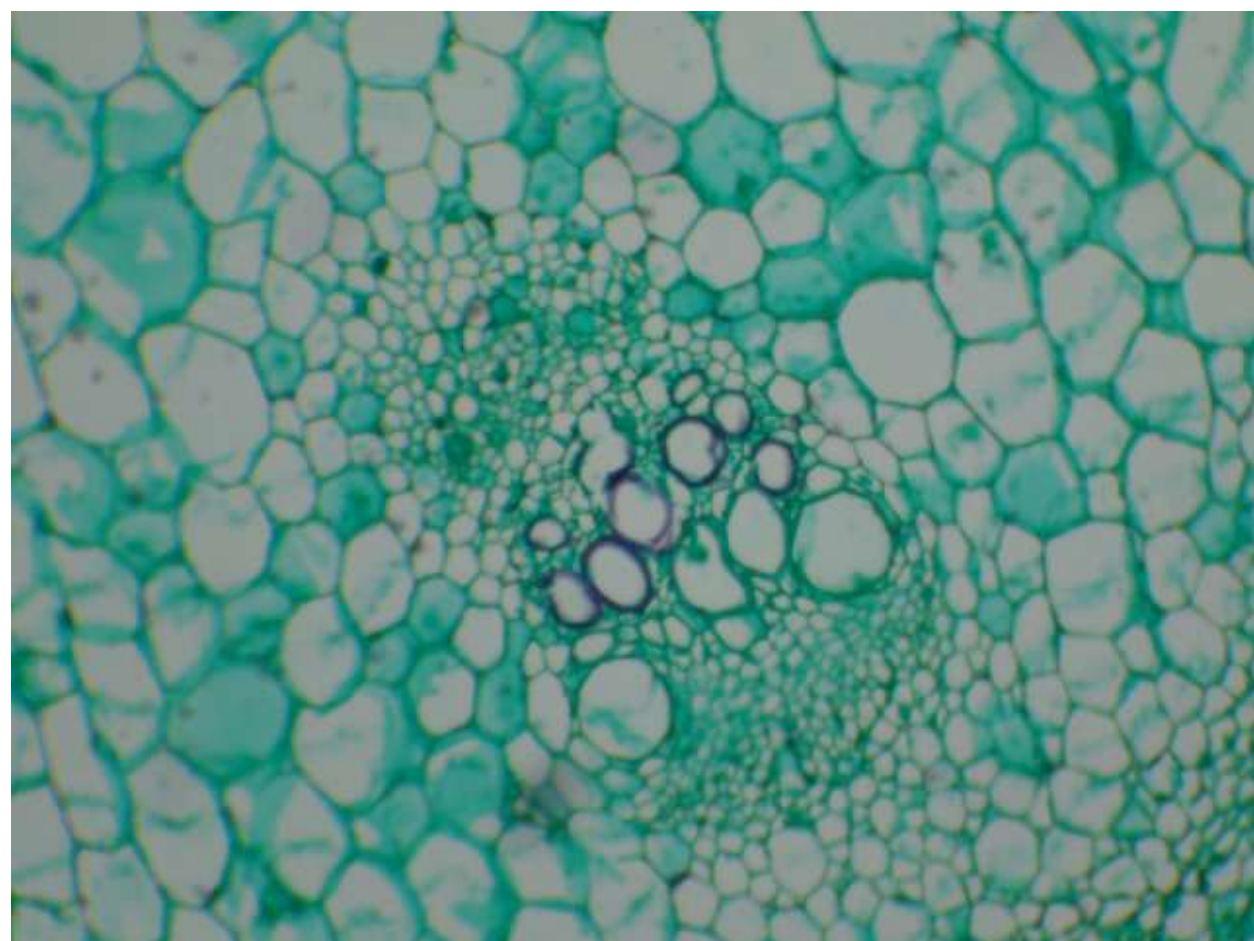

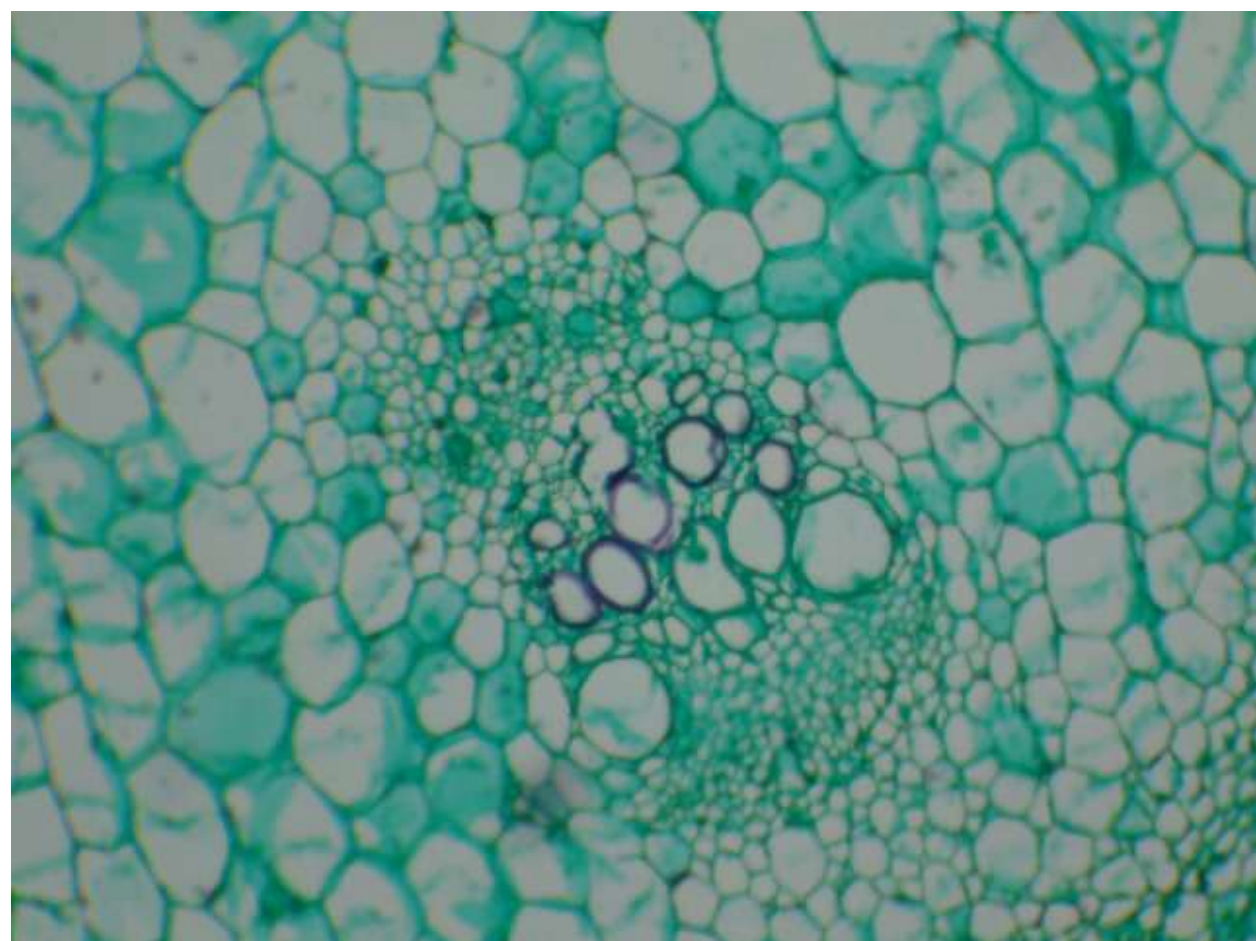

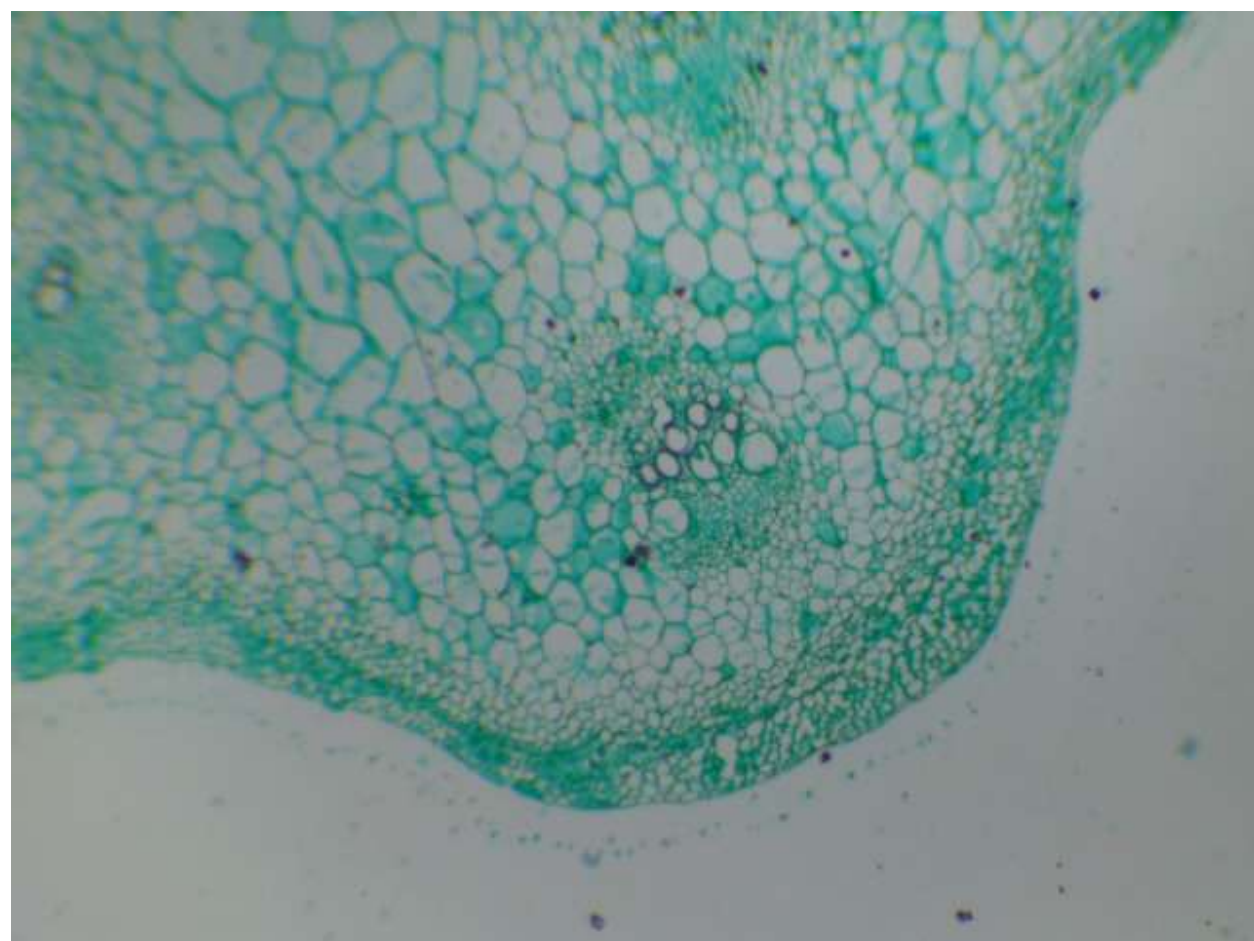

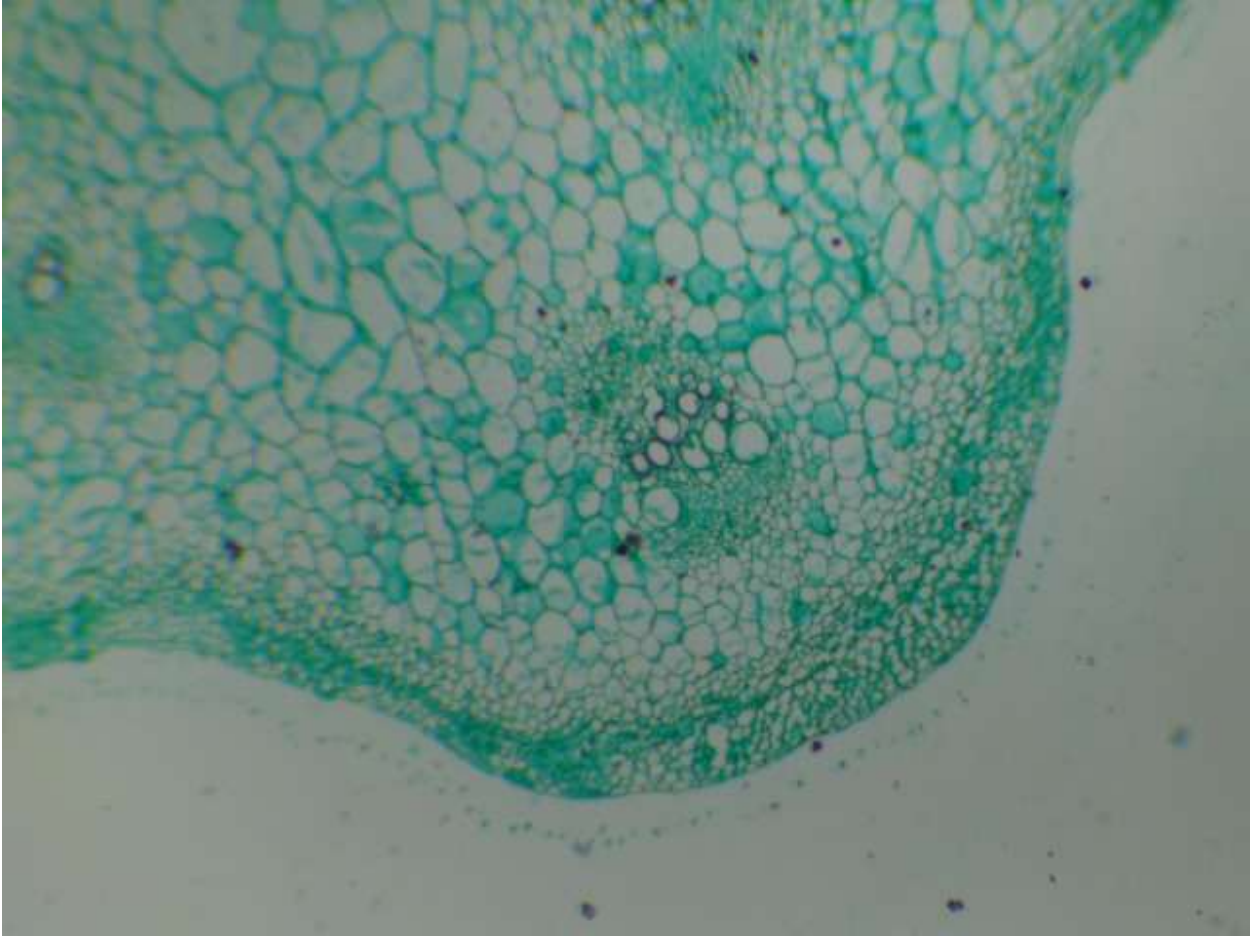

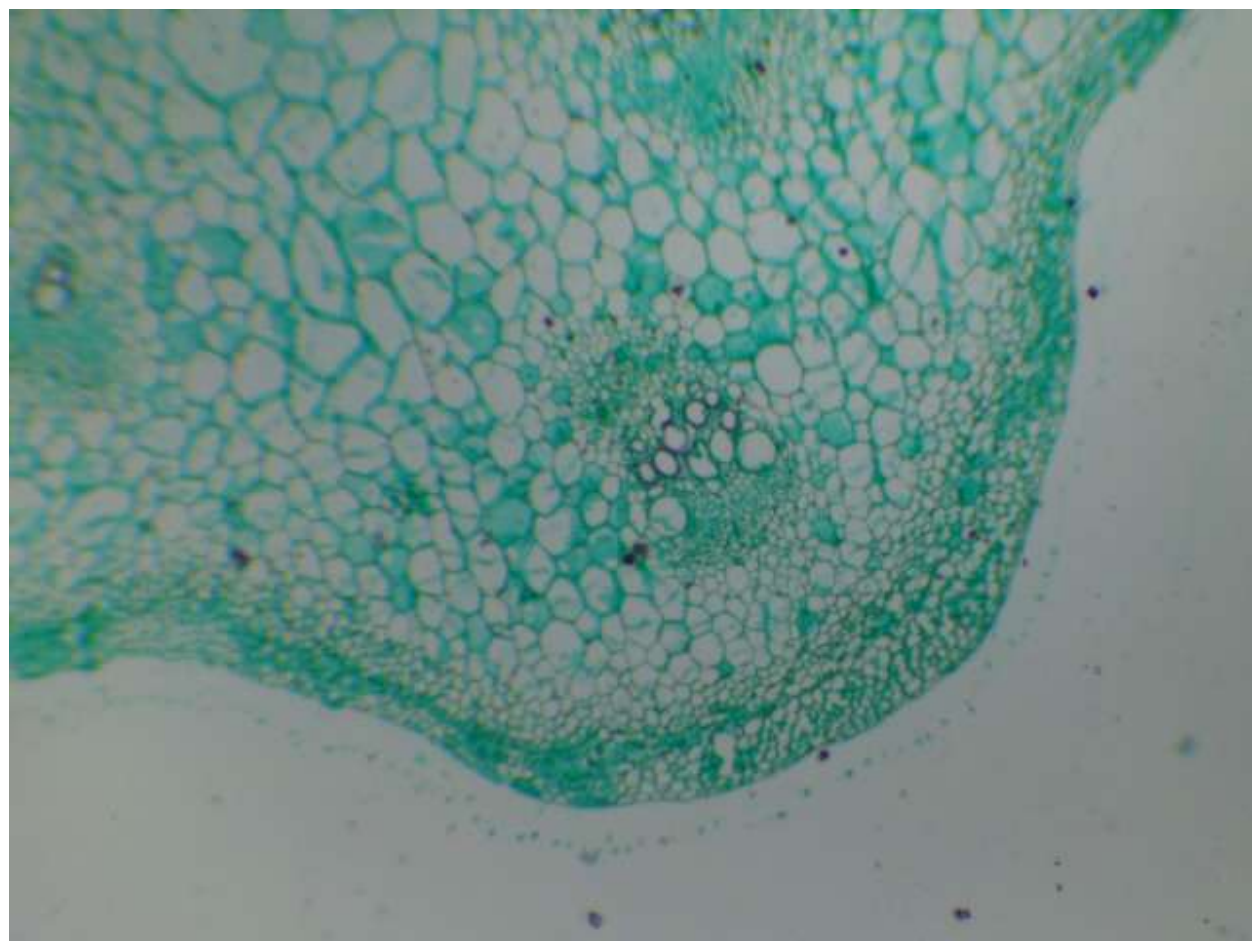

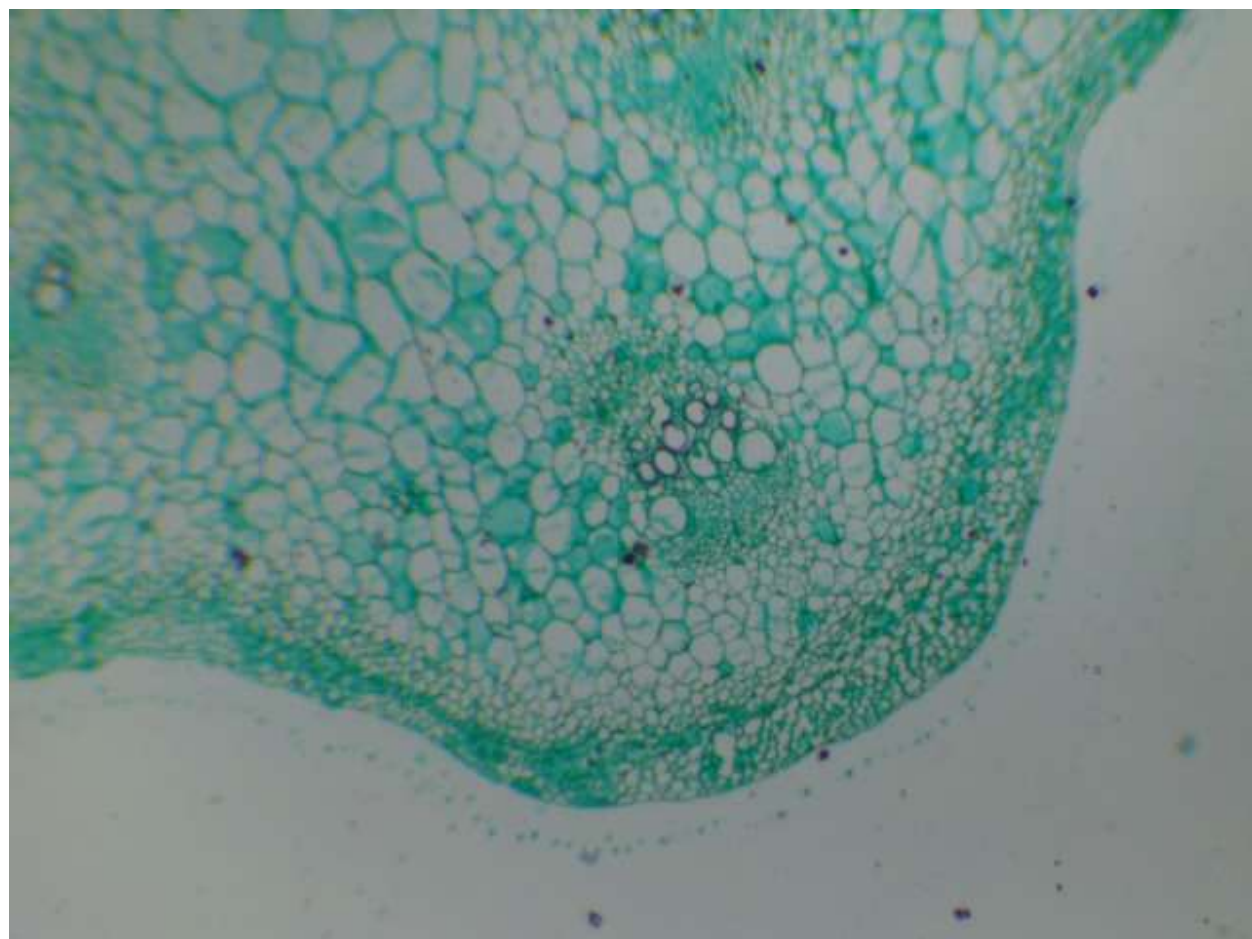

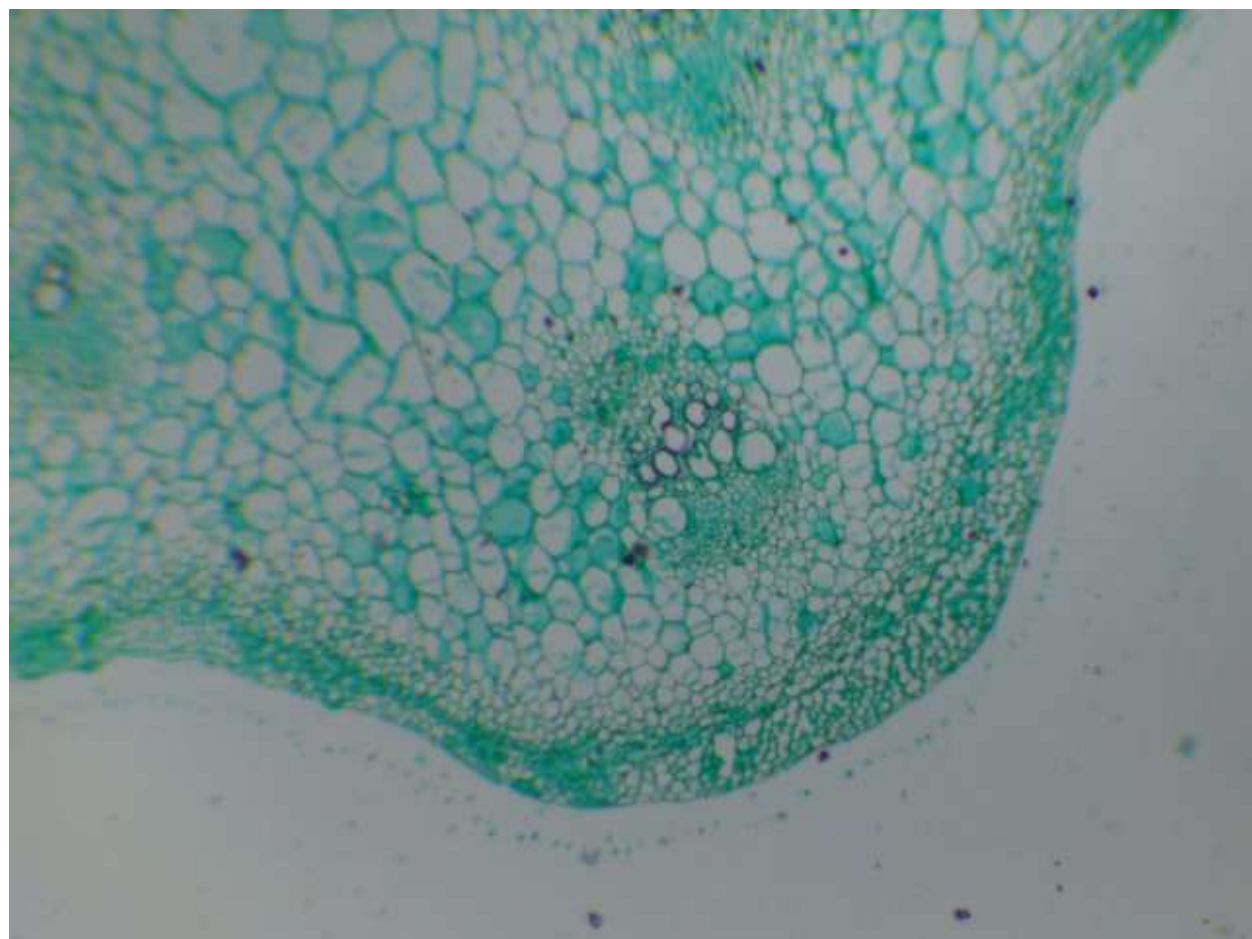

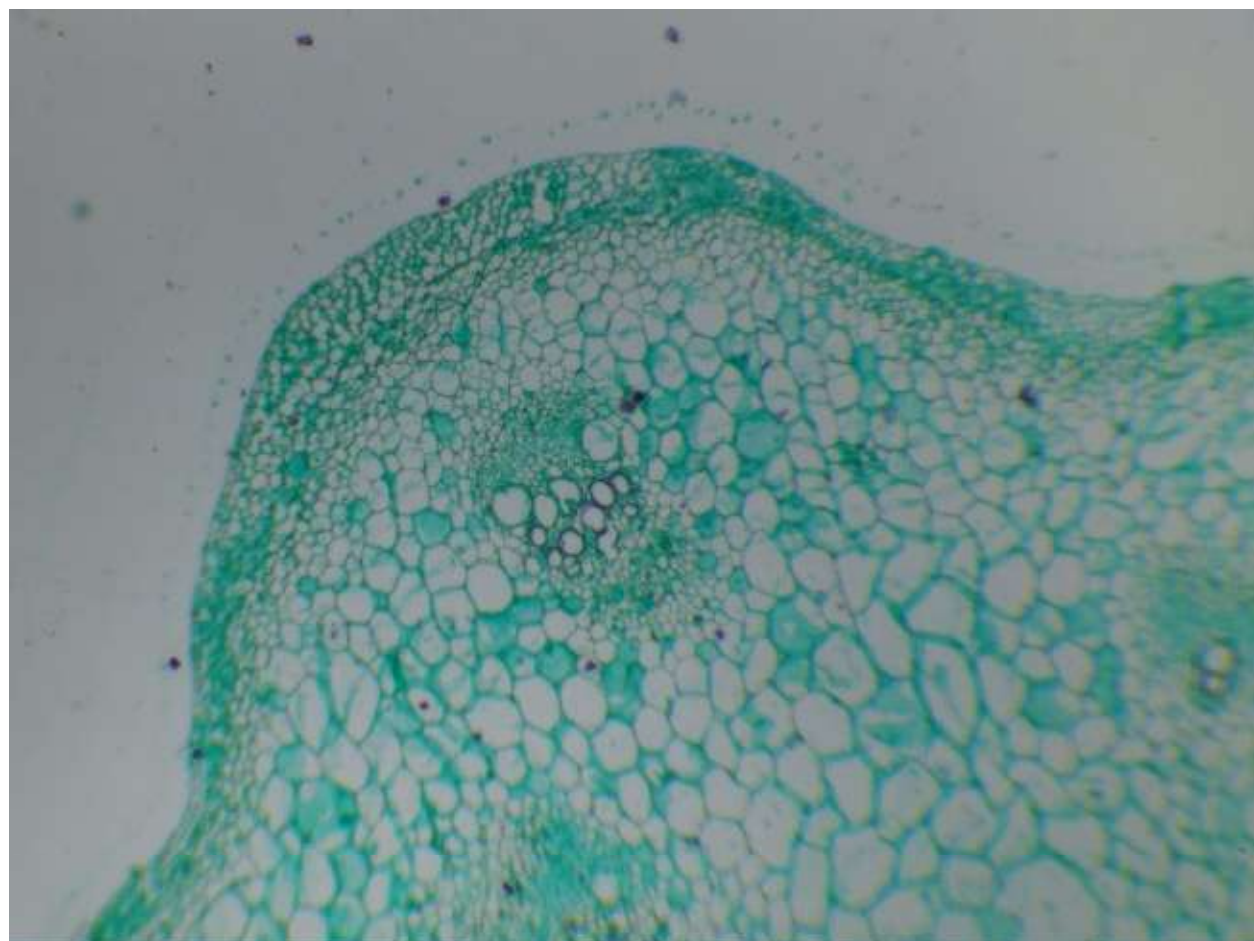

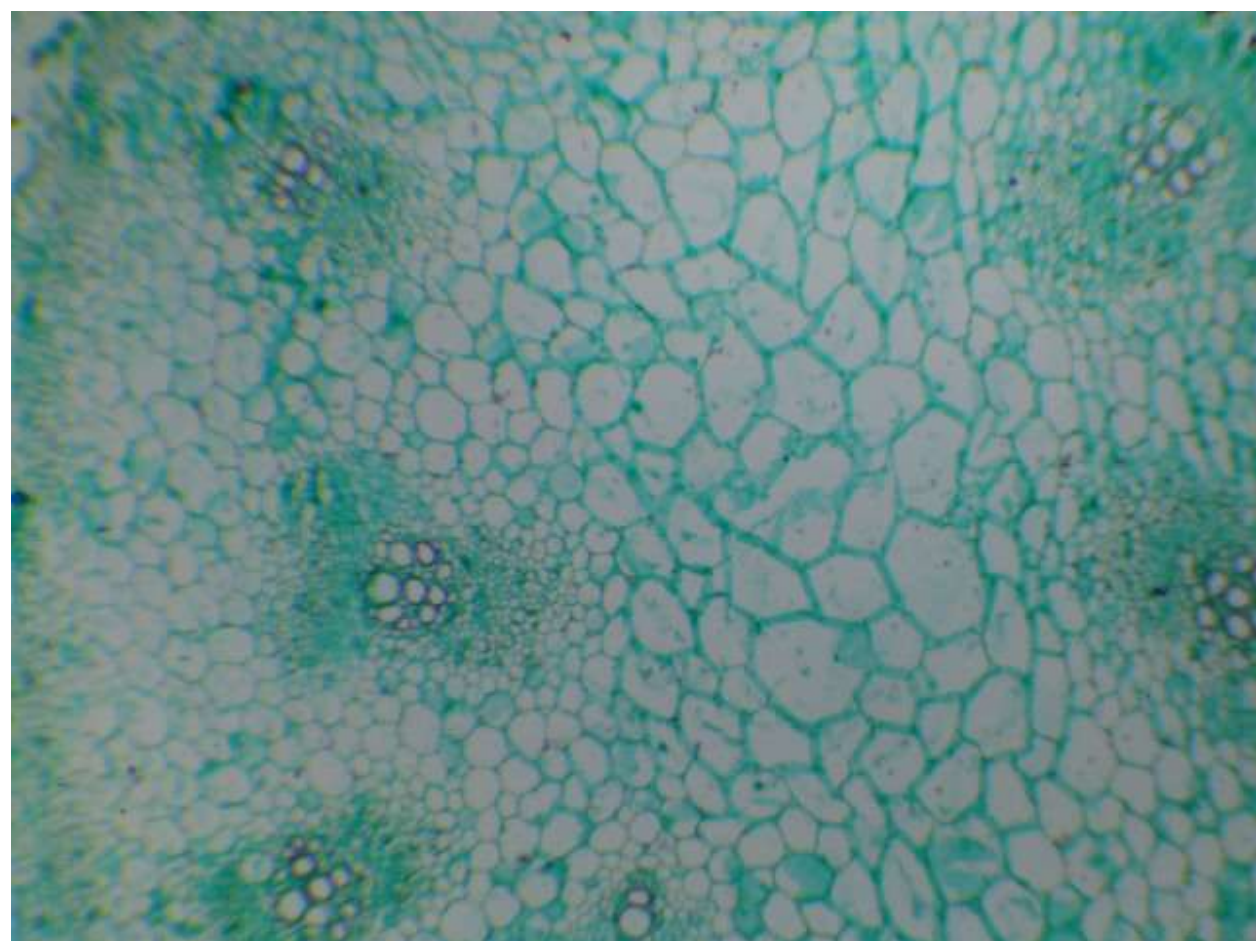

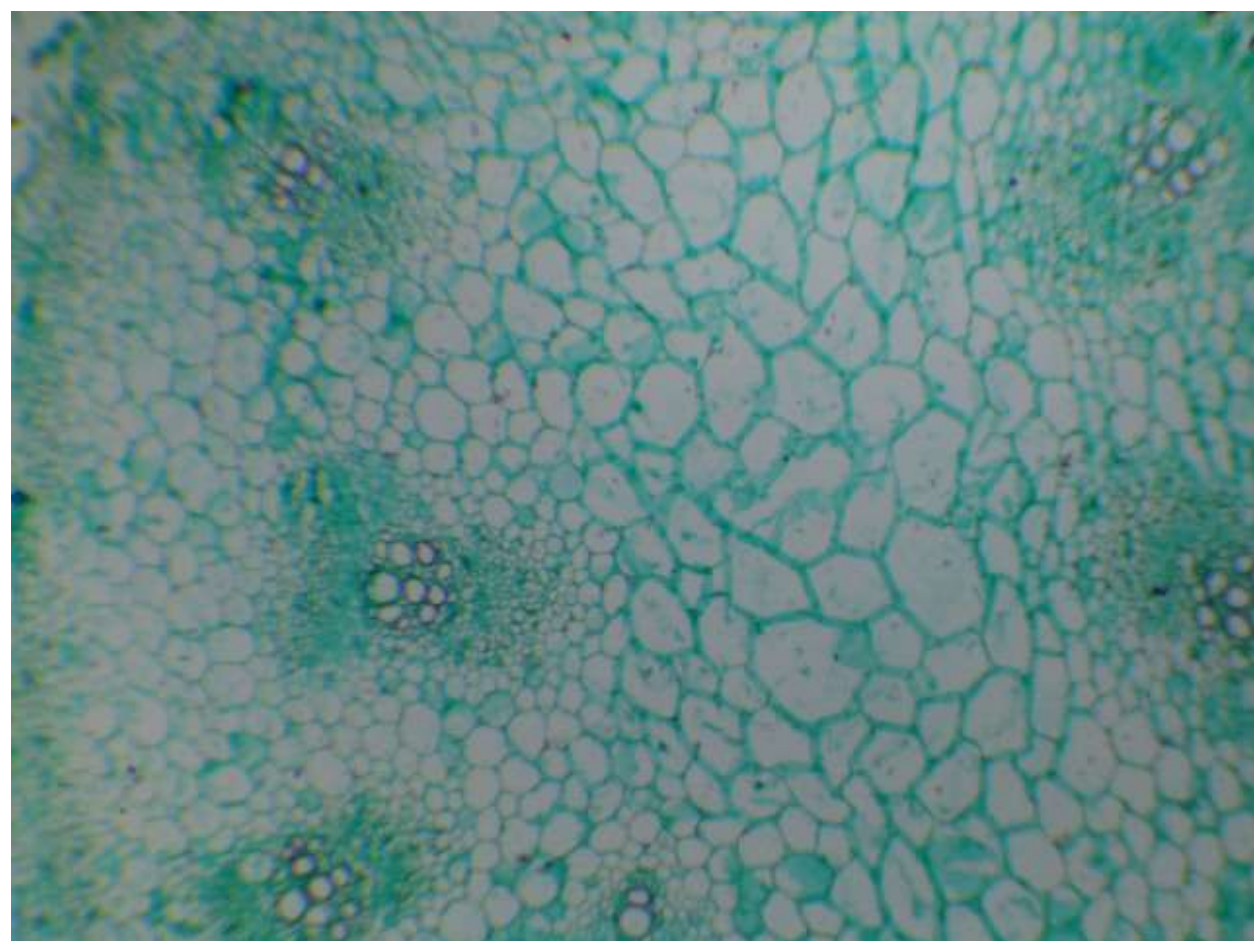

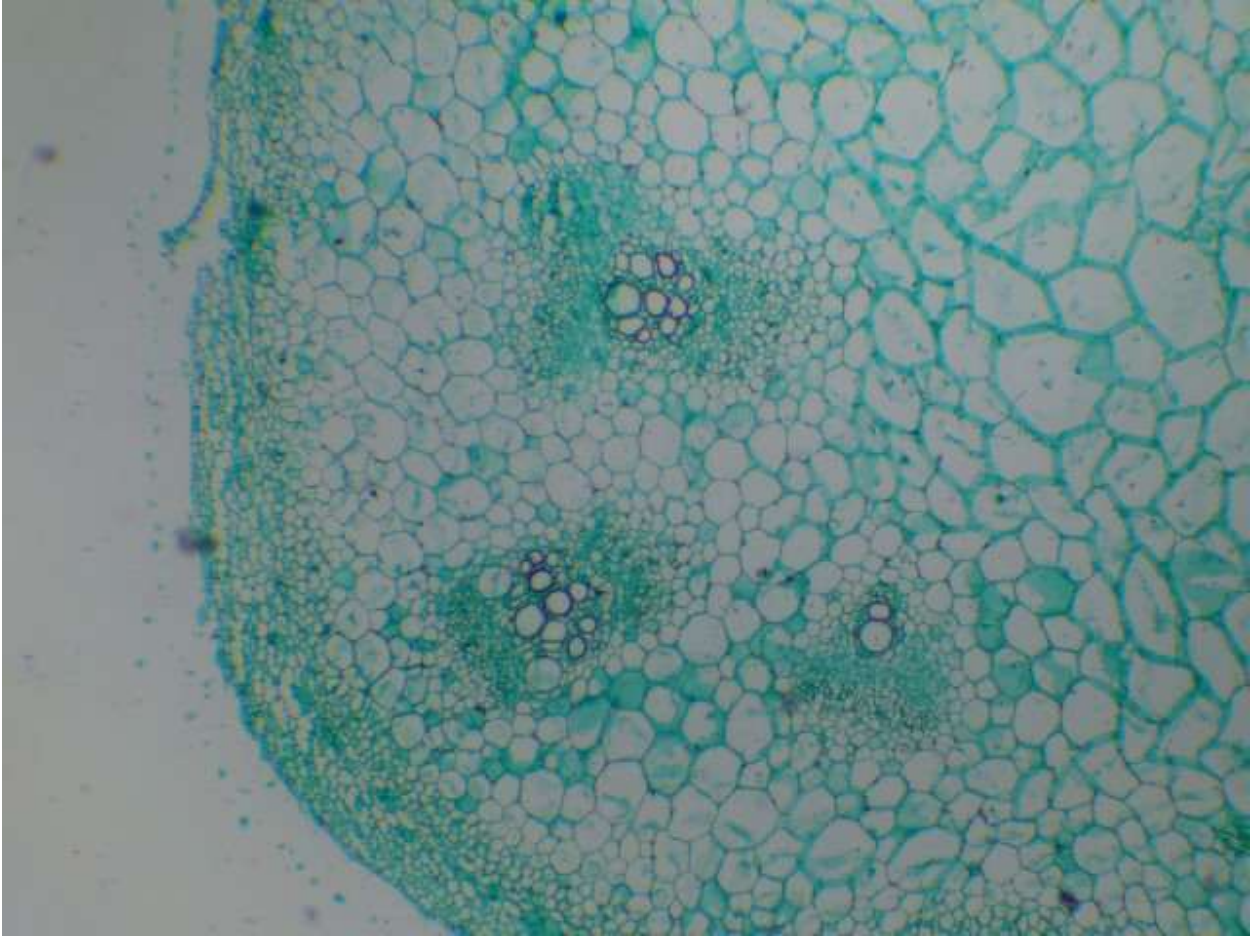

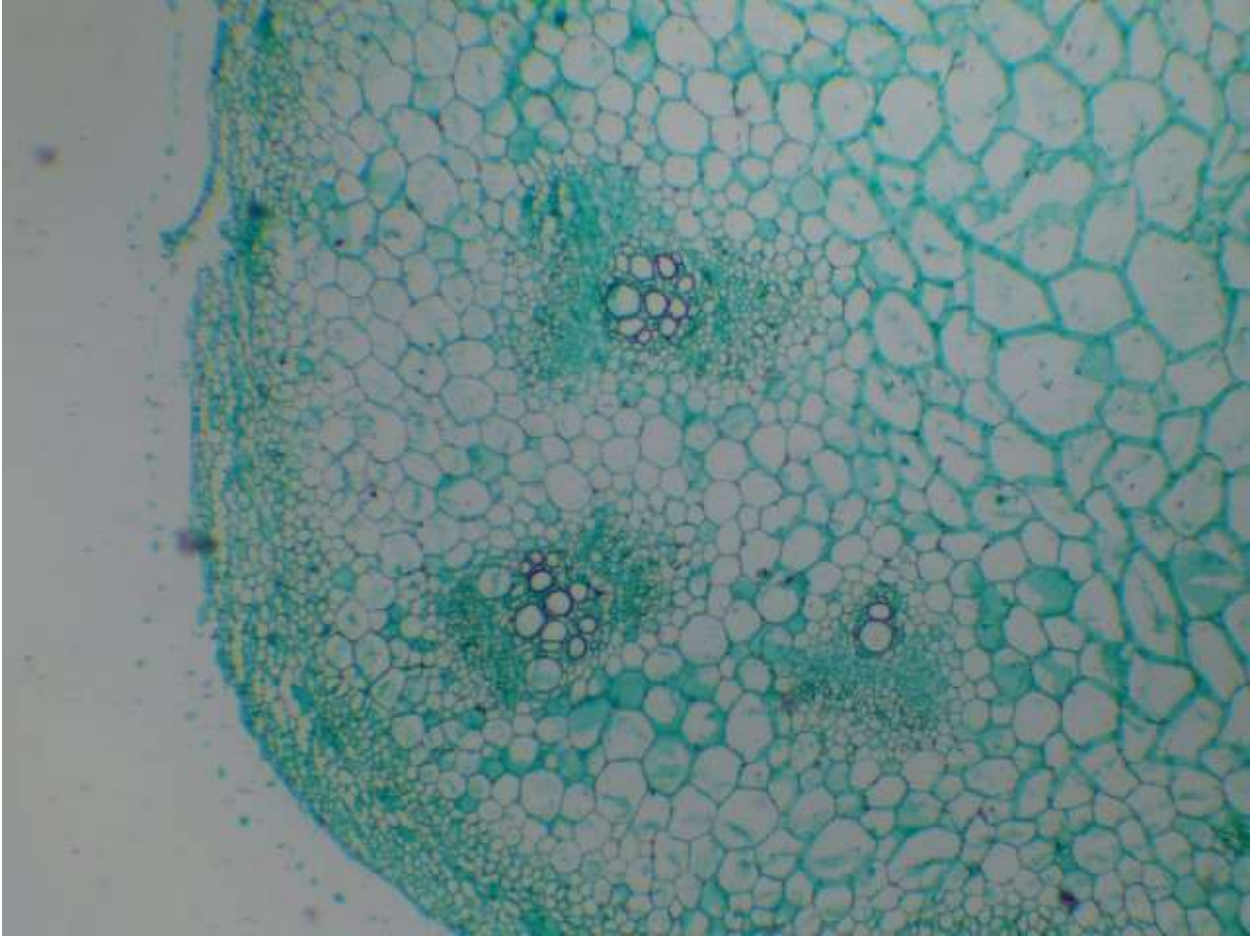

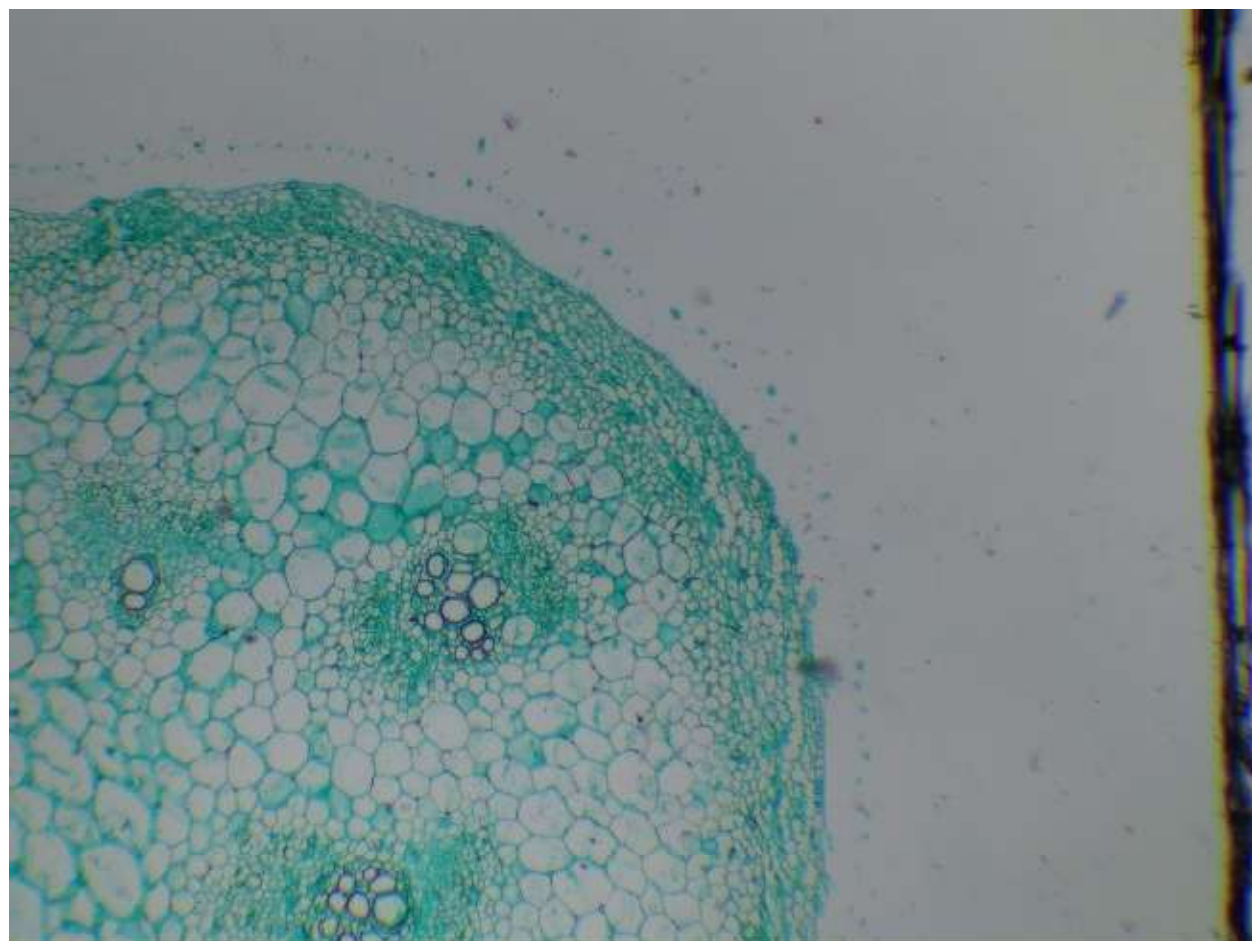

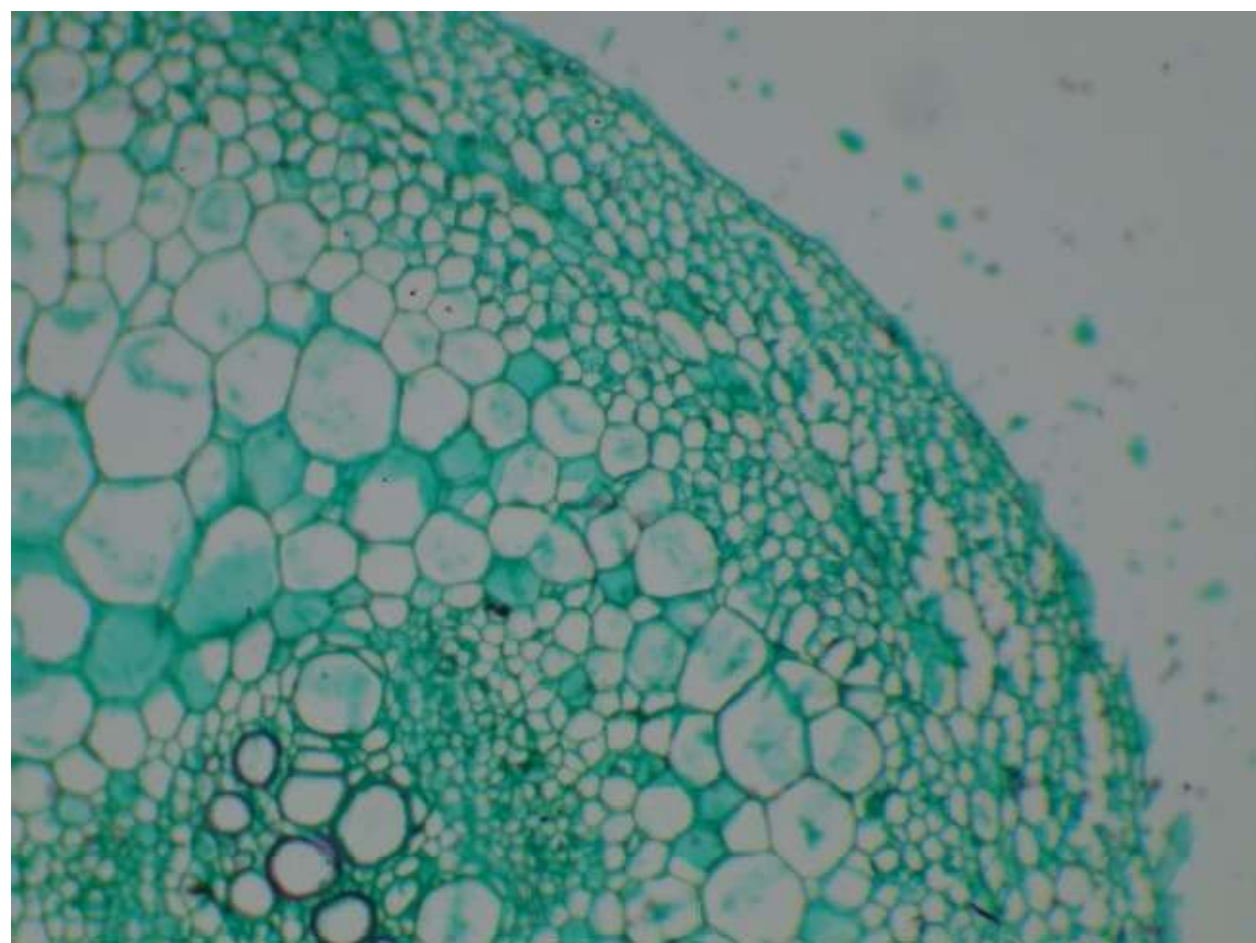

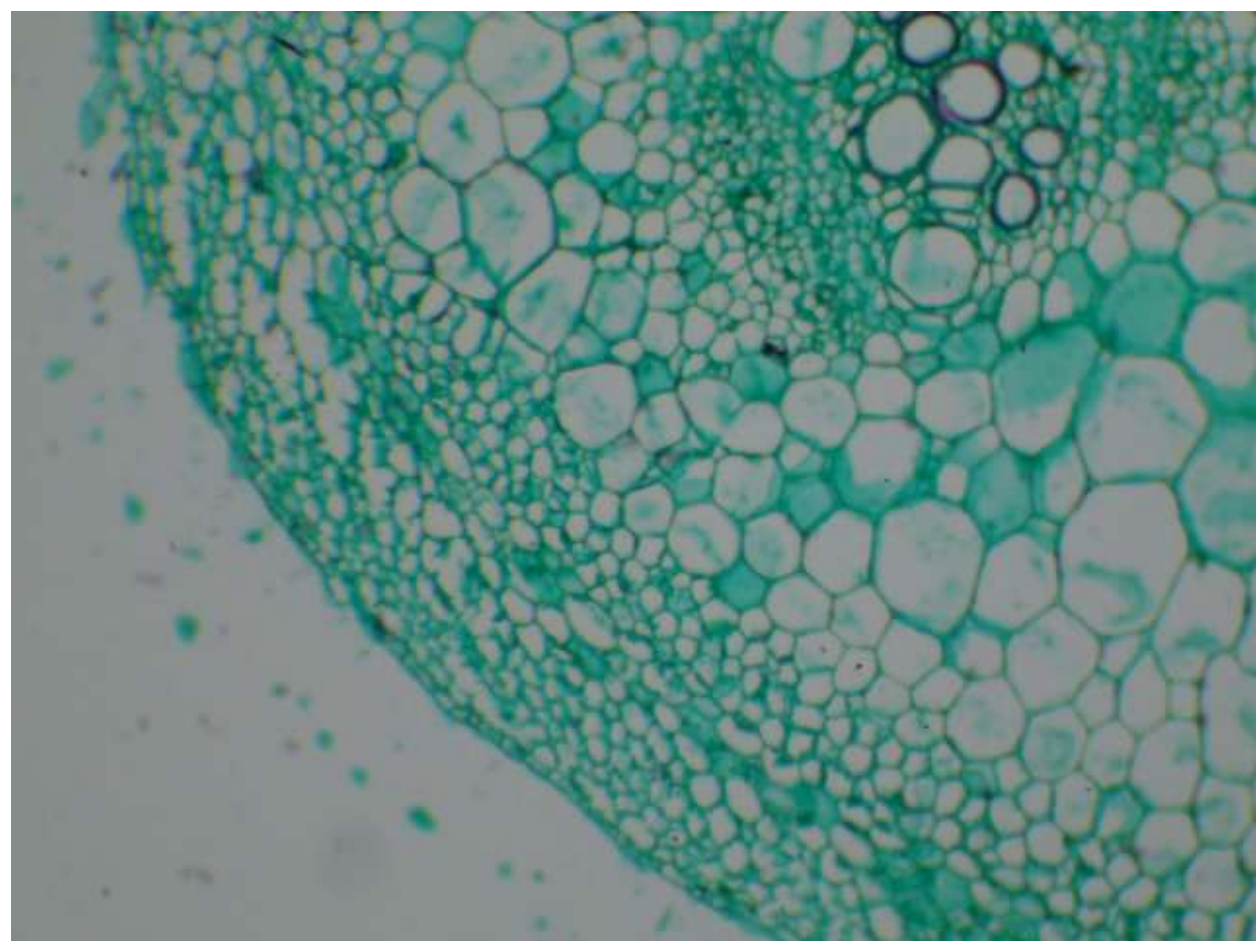

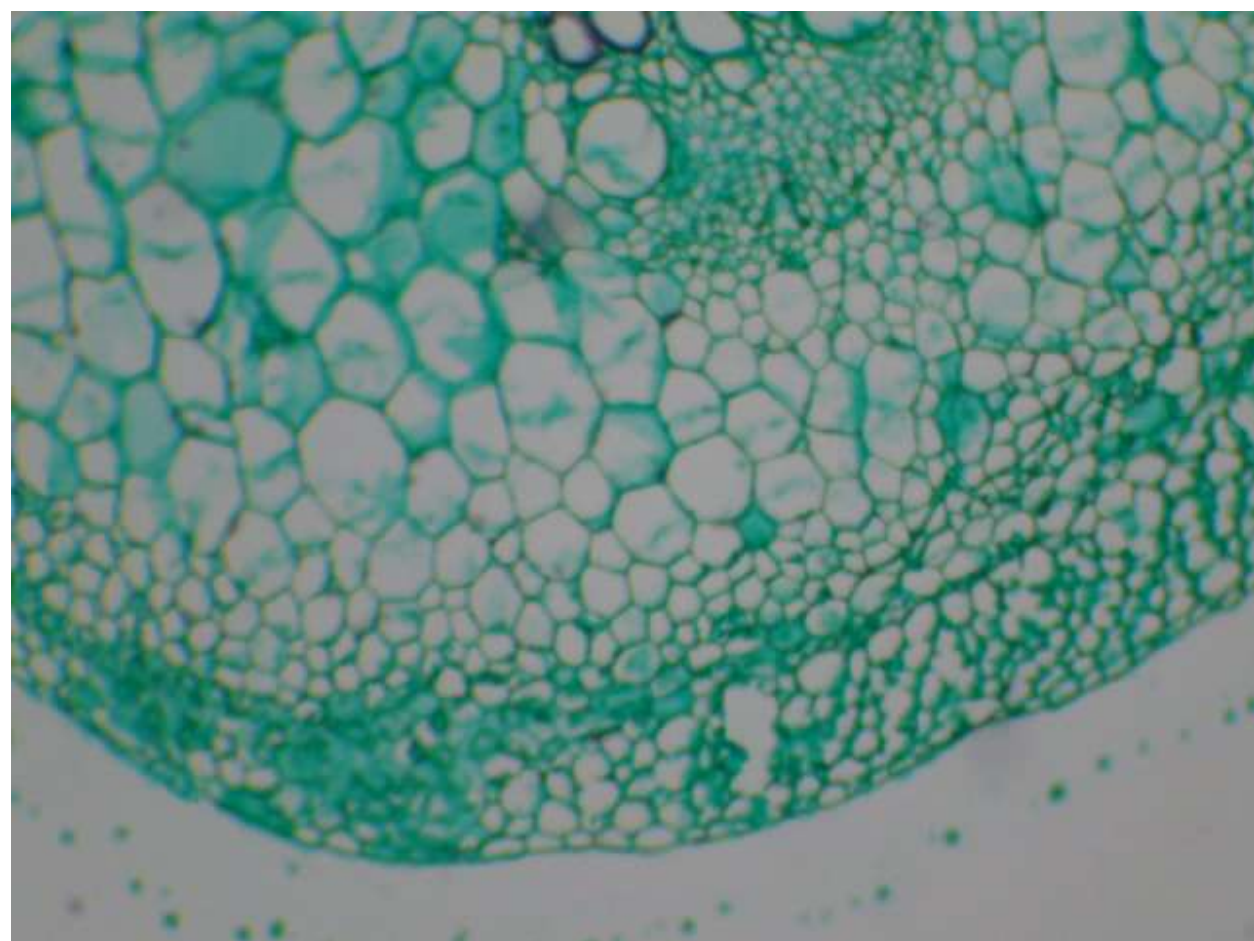

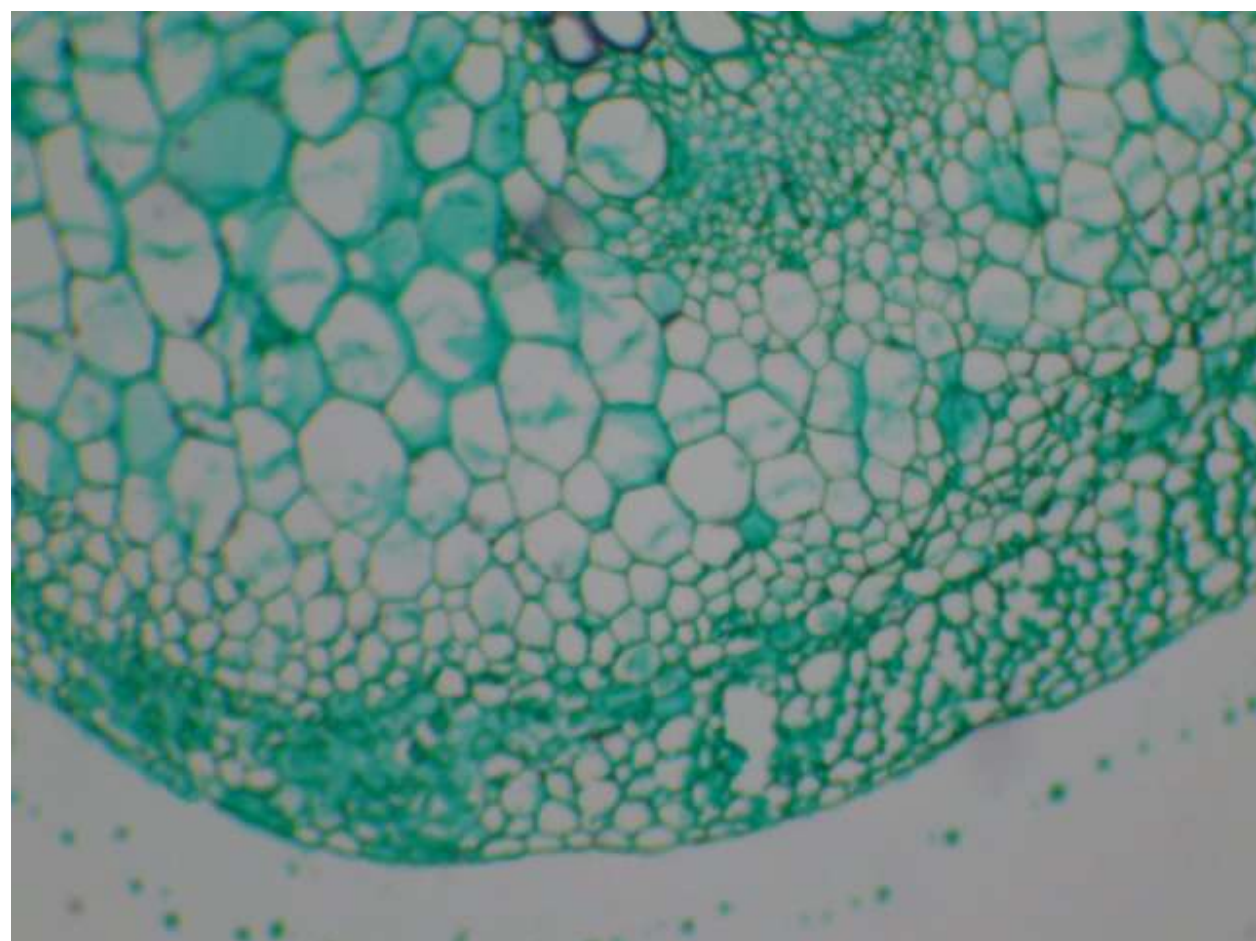

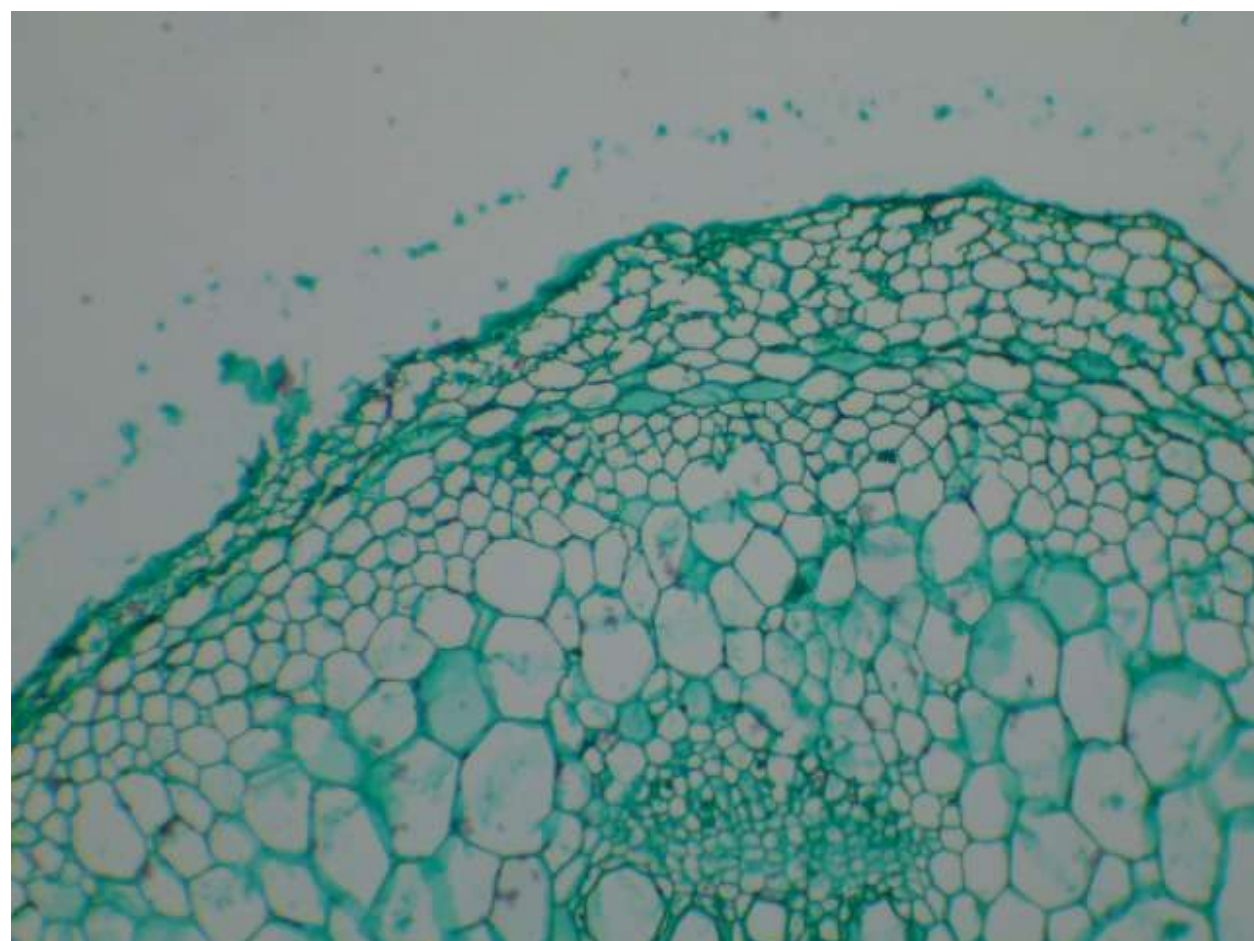

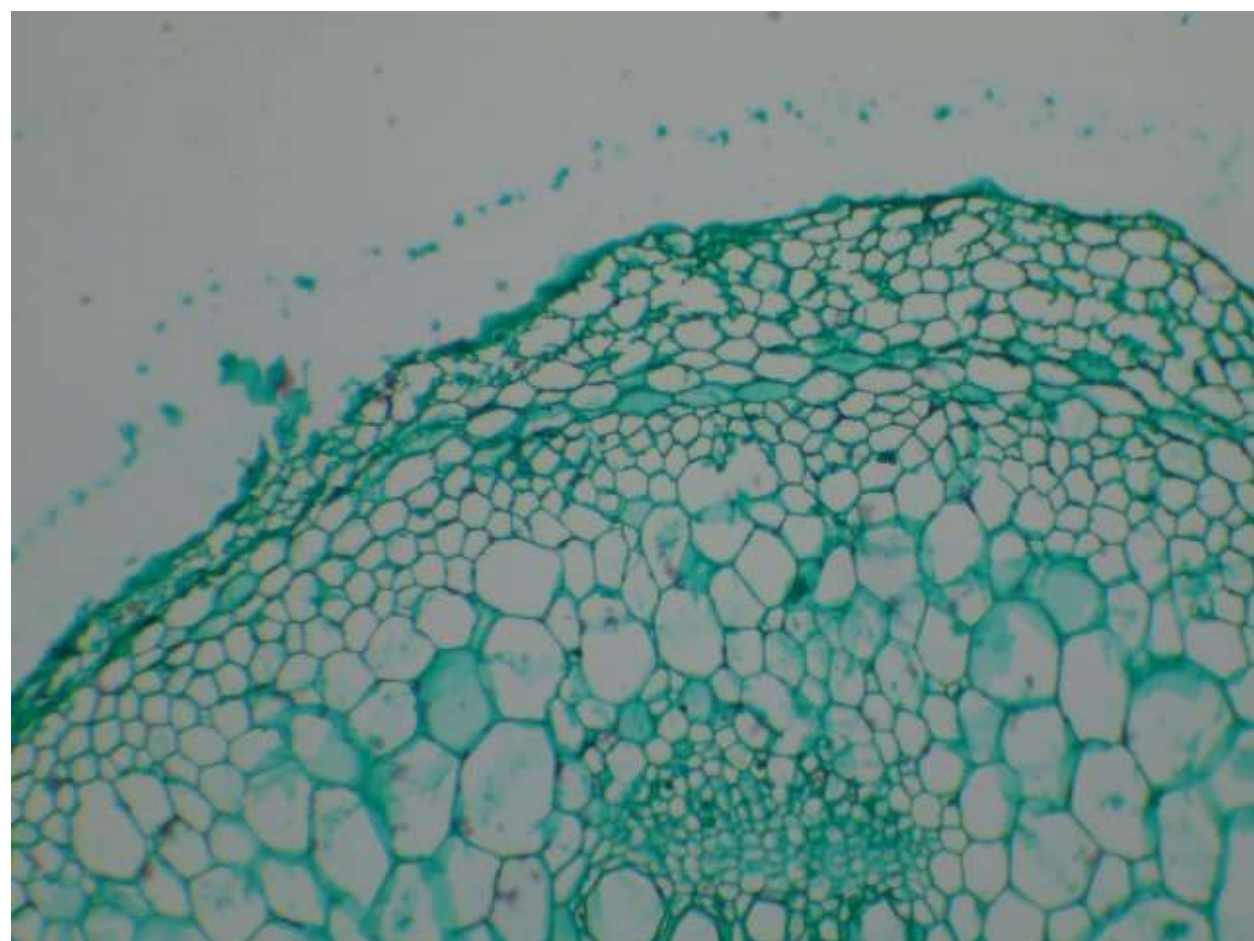

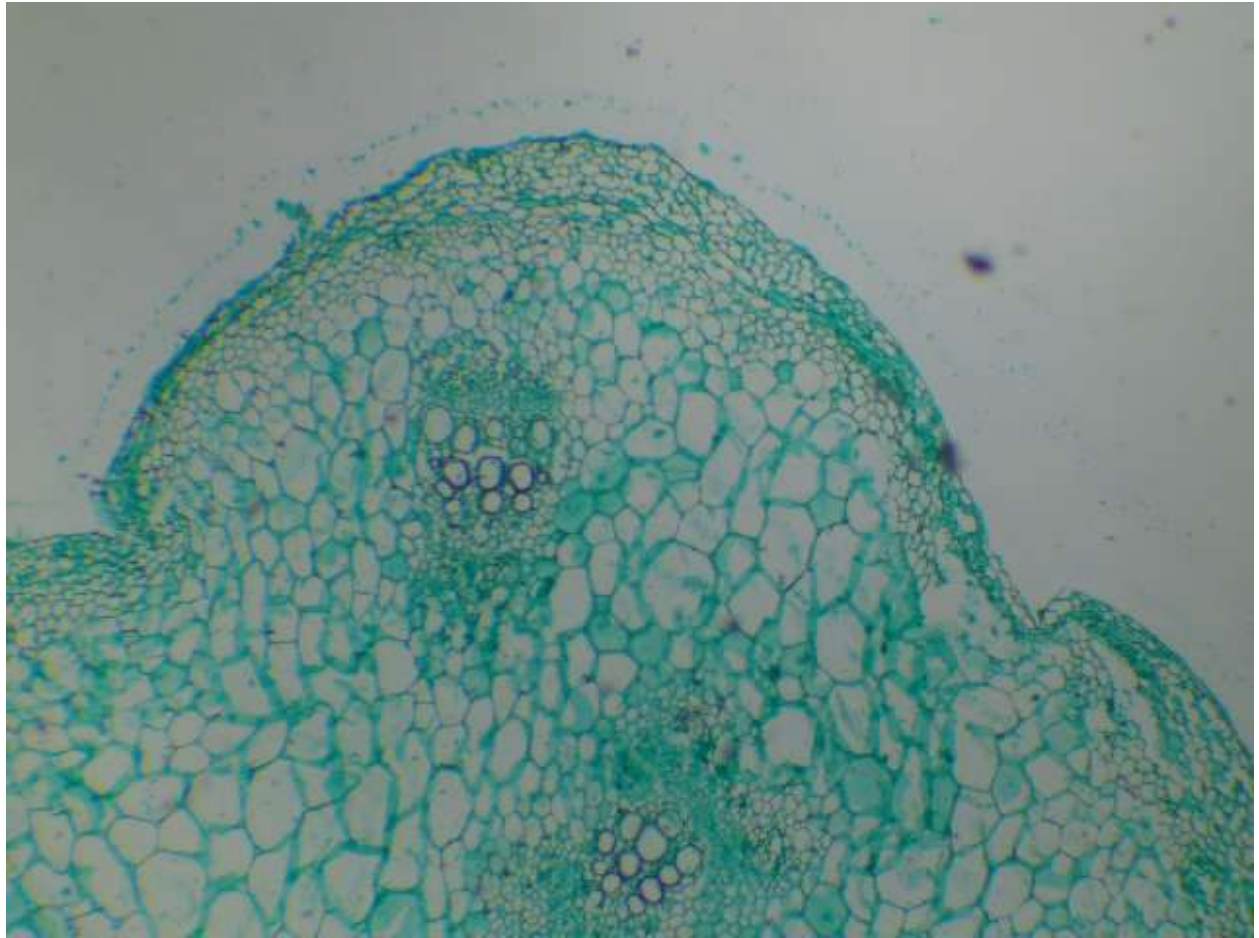

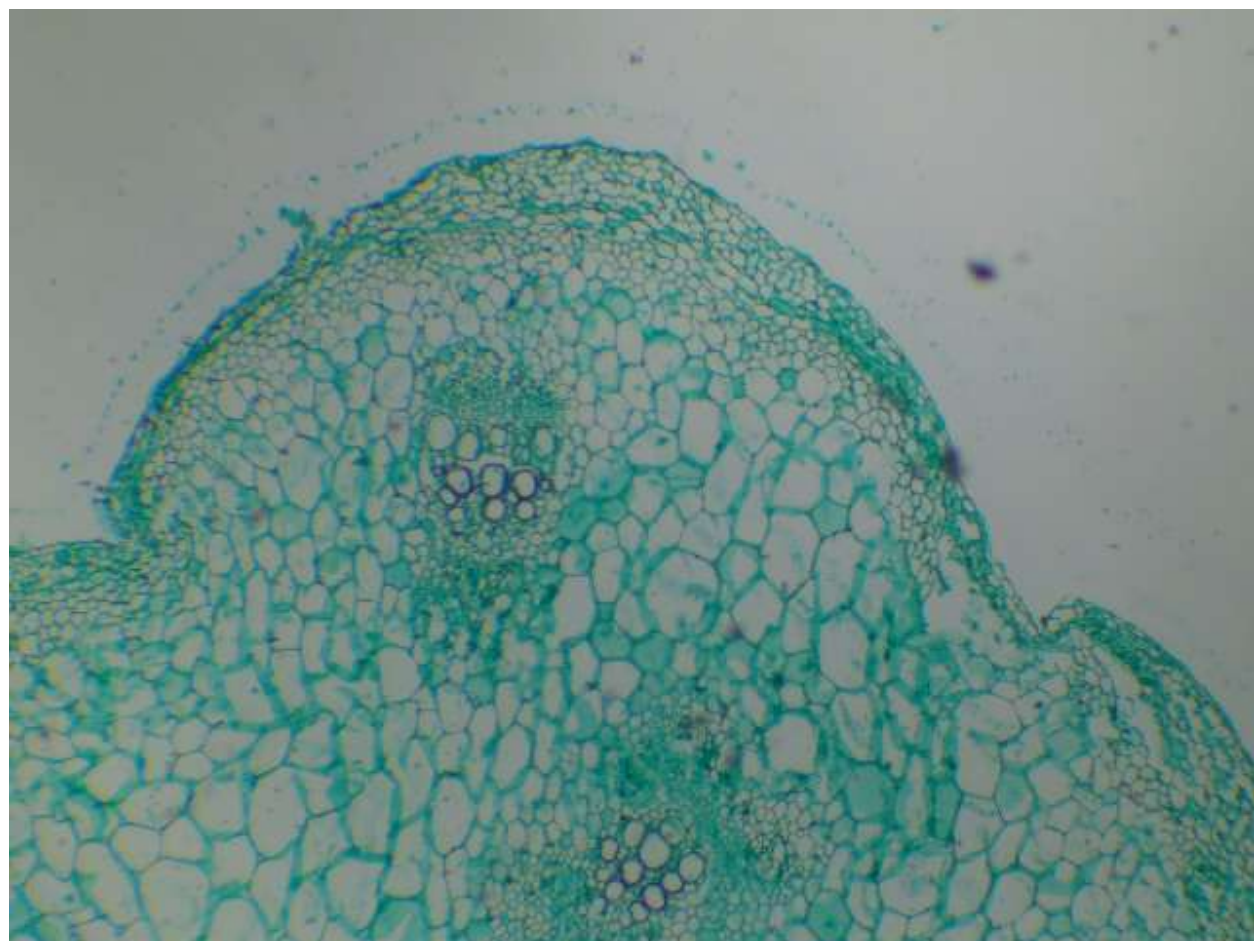

Supplement: Supplementary file 1 — Supplementary Material 1 [file 41598_2026_47246_MOESM1_ESM.pdf]

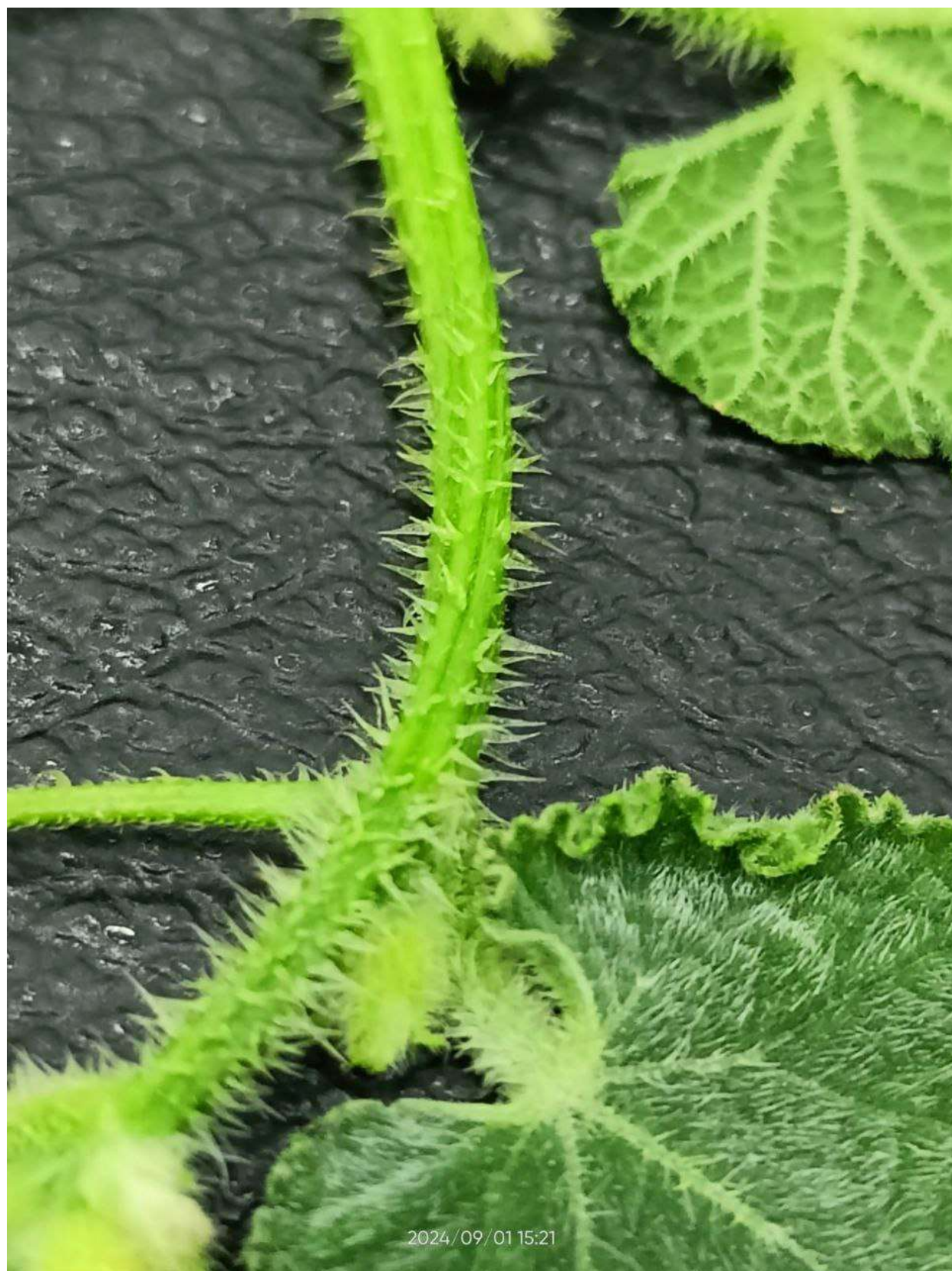

2024/09/01 15:21

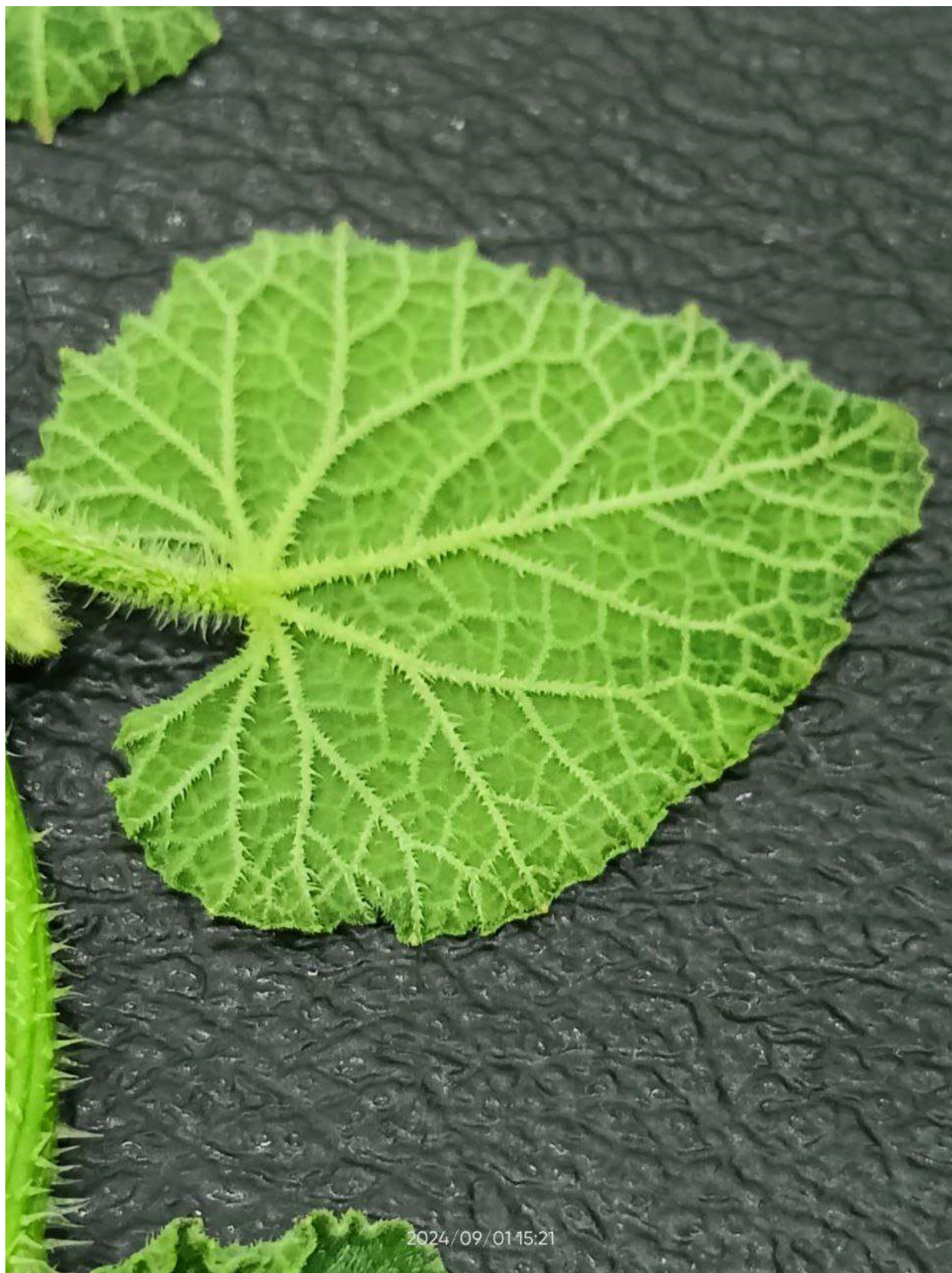

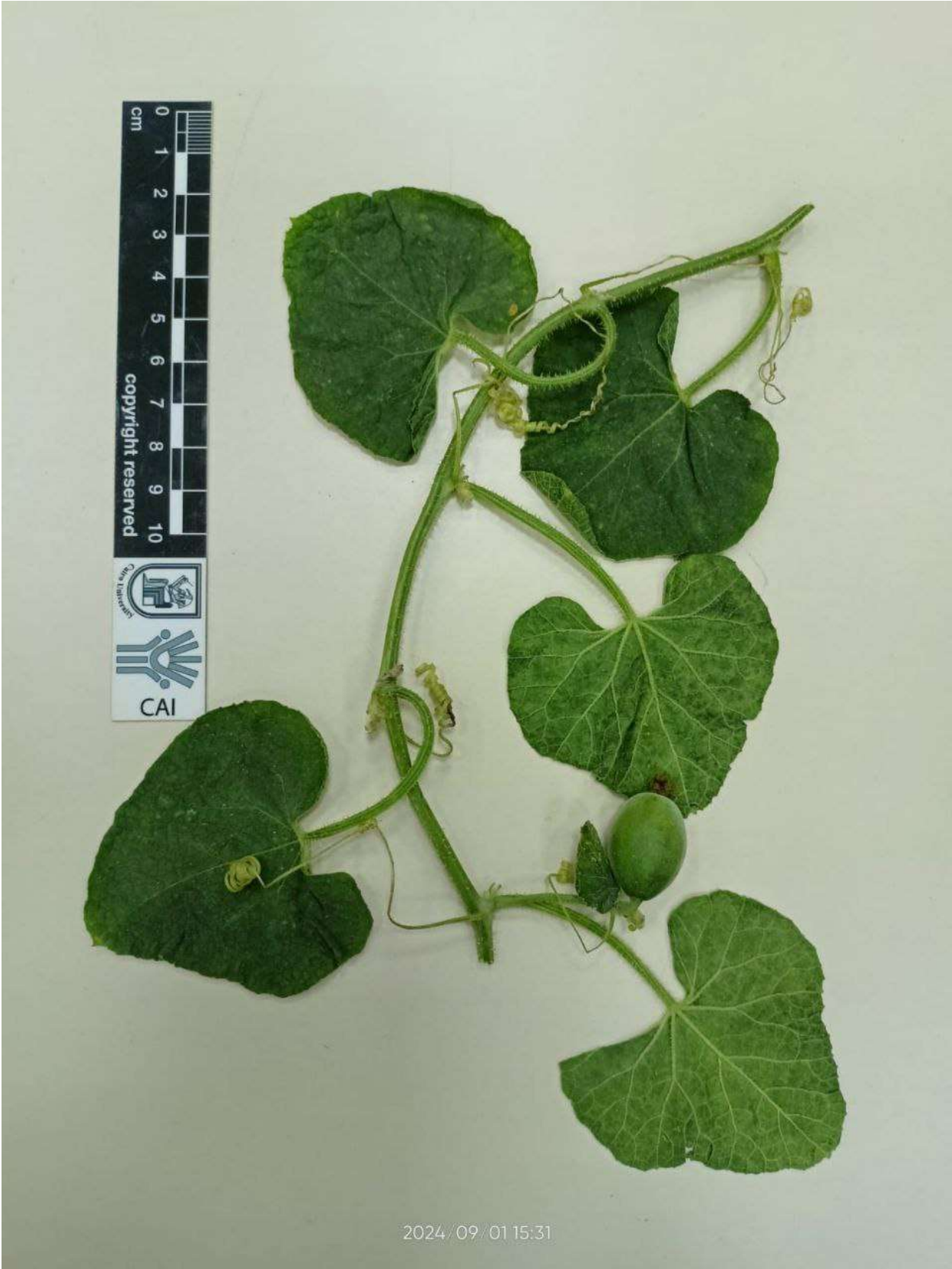

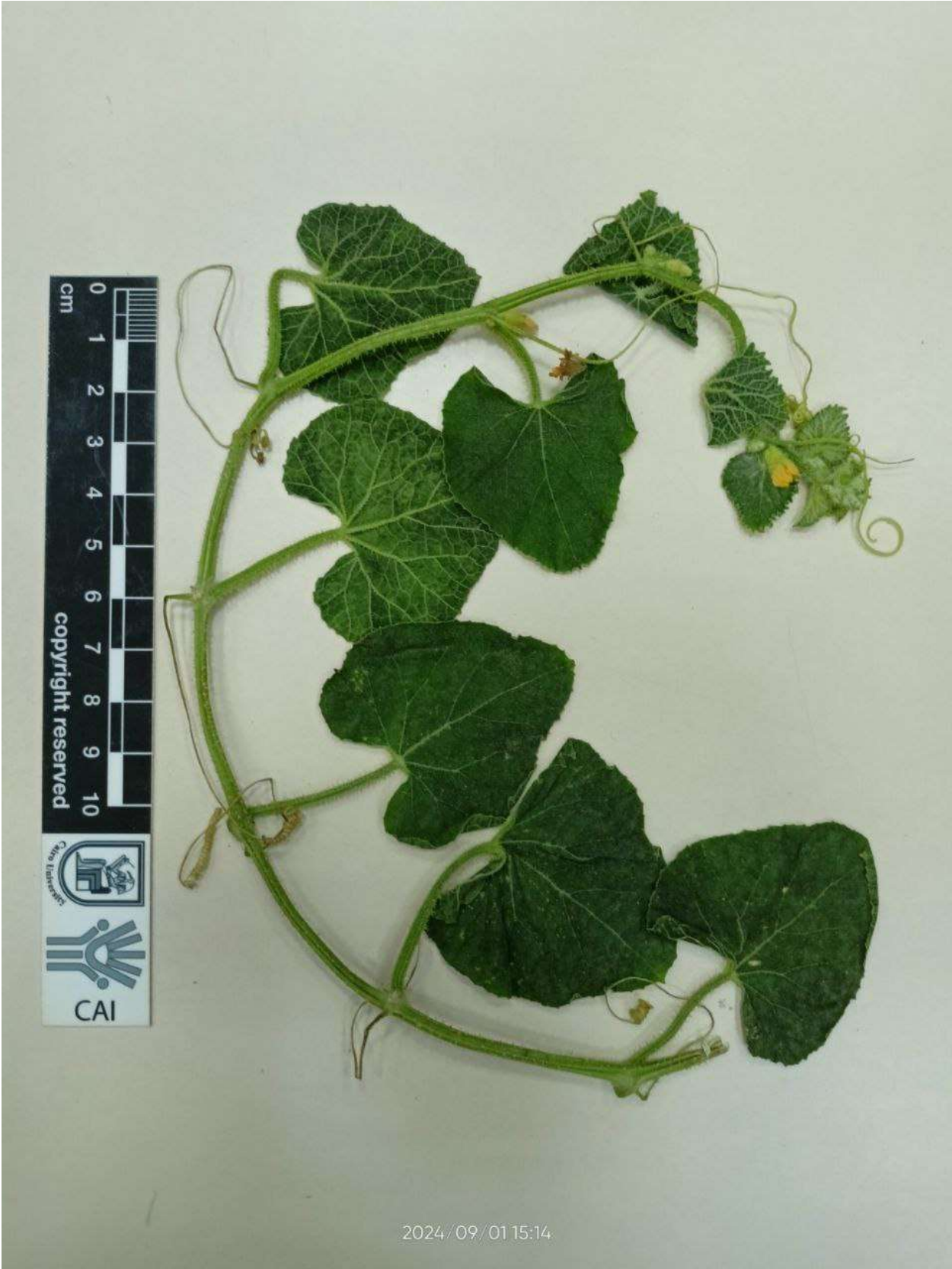

2024/09/01 15:14

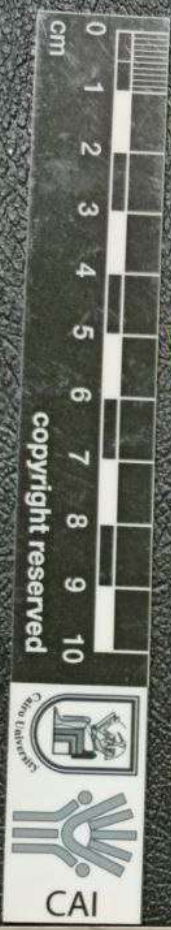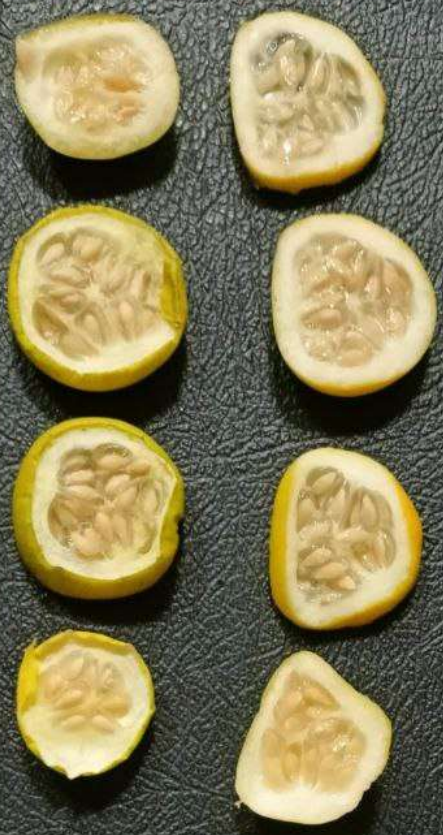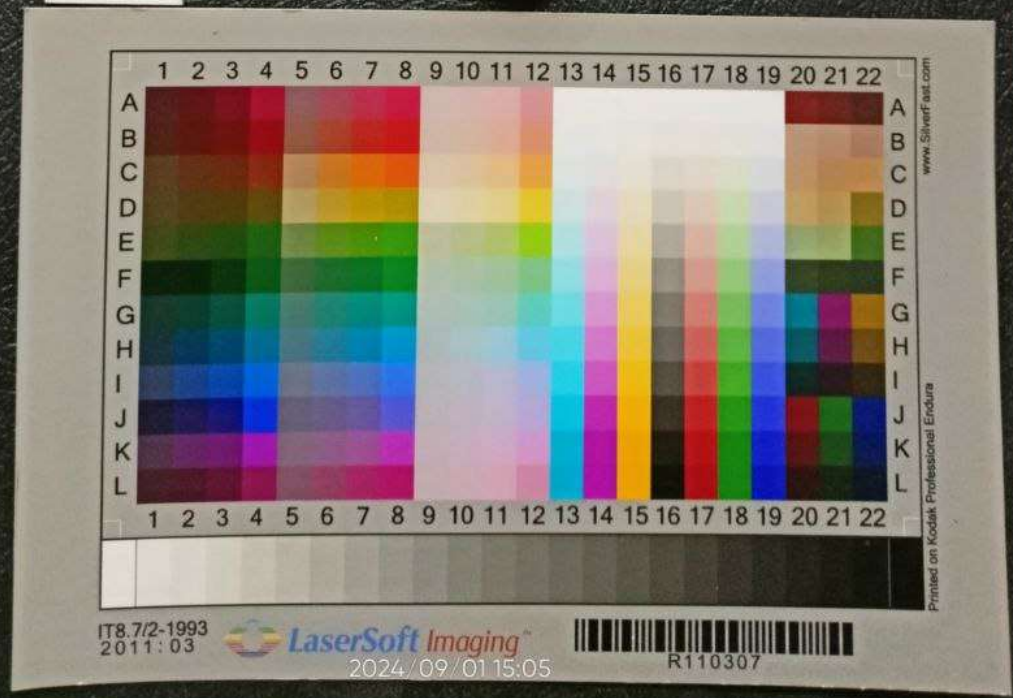

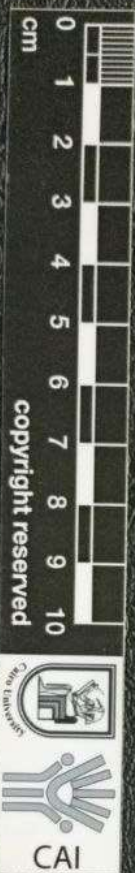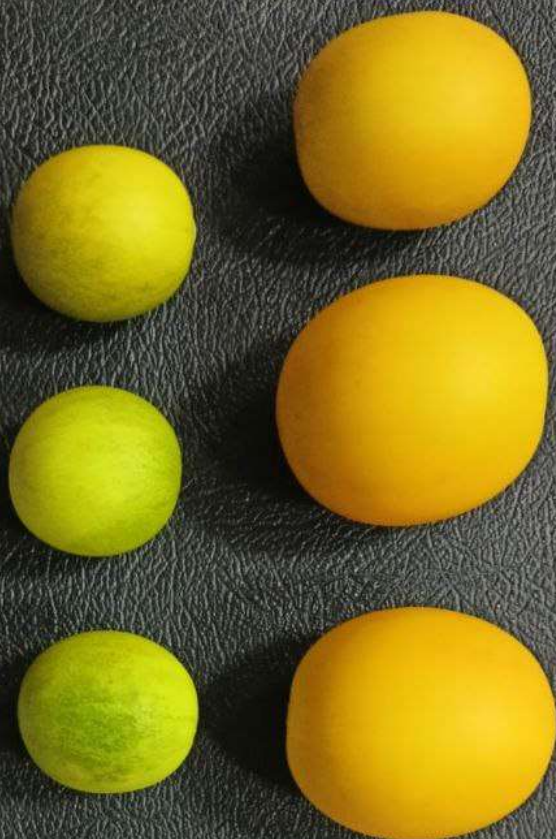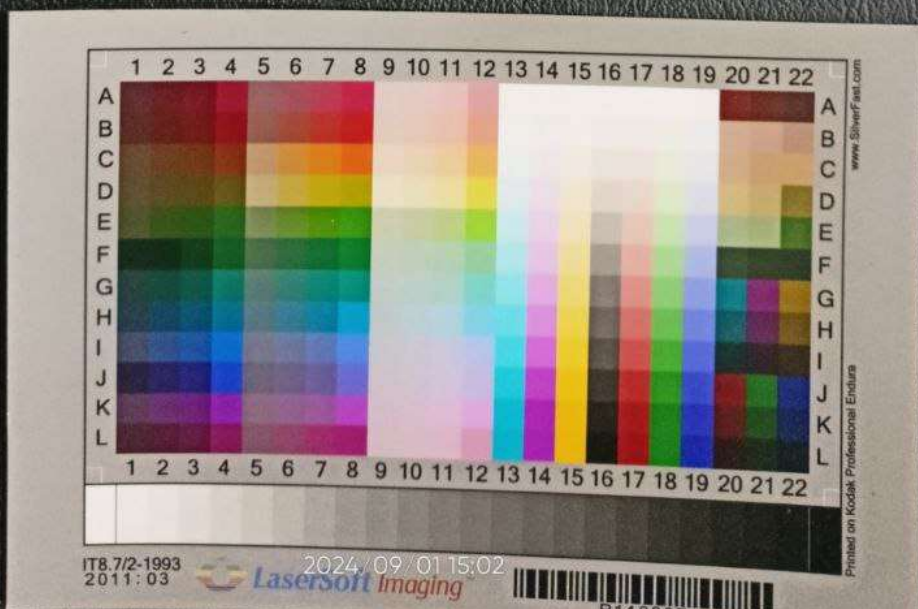

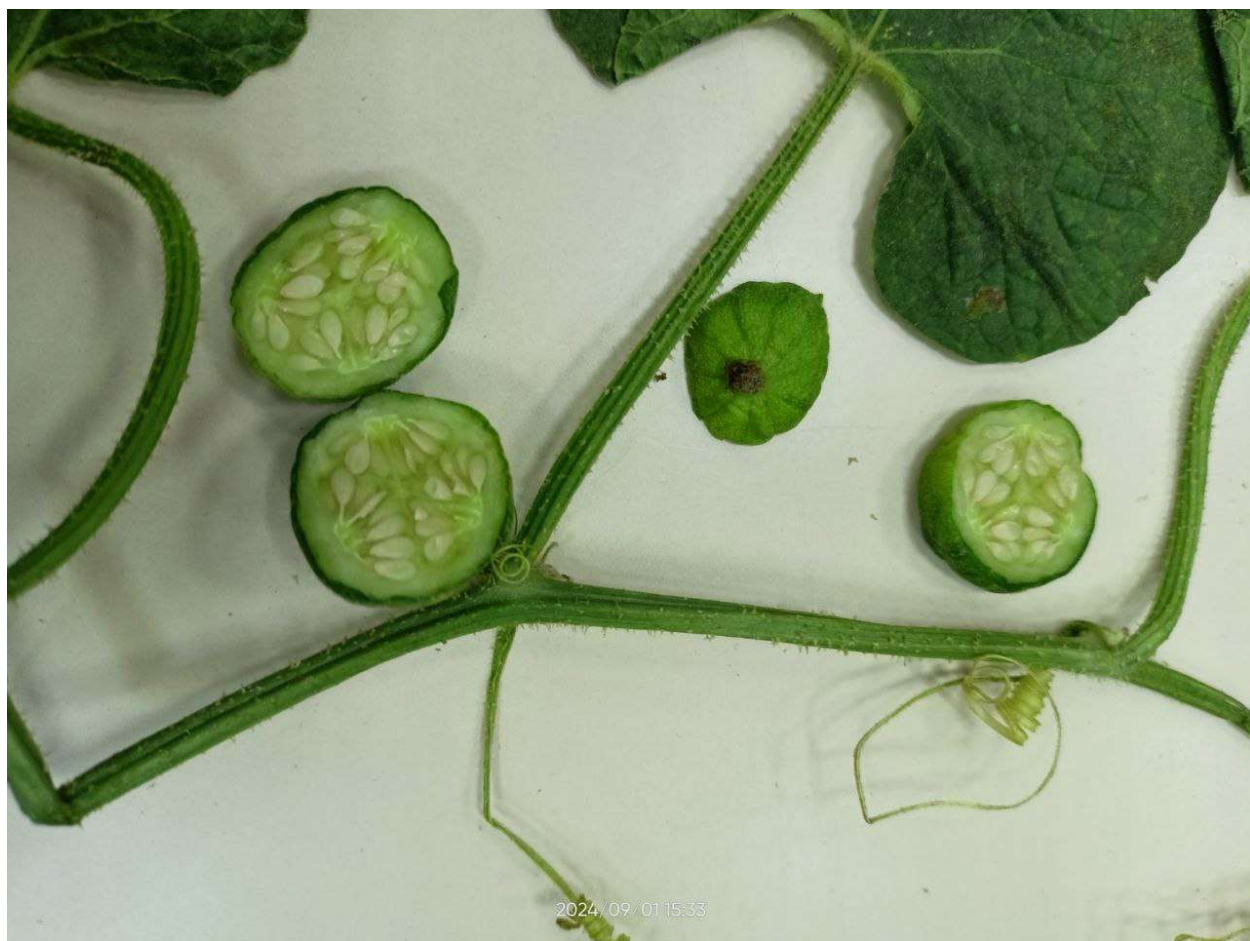

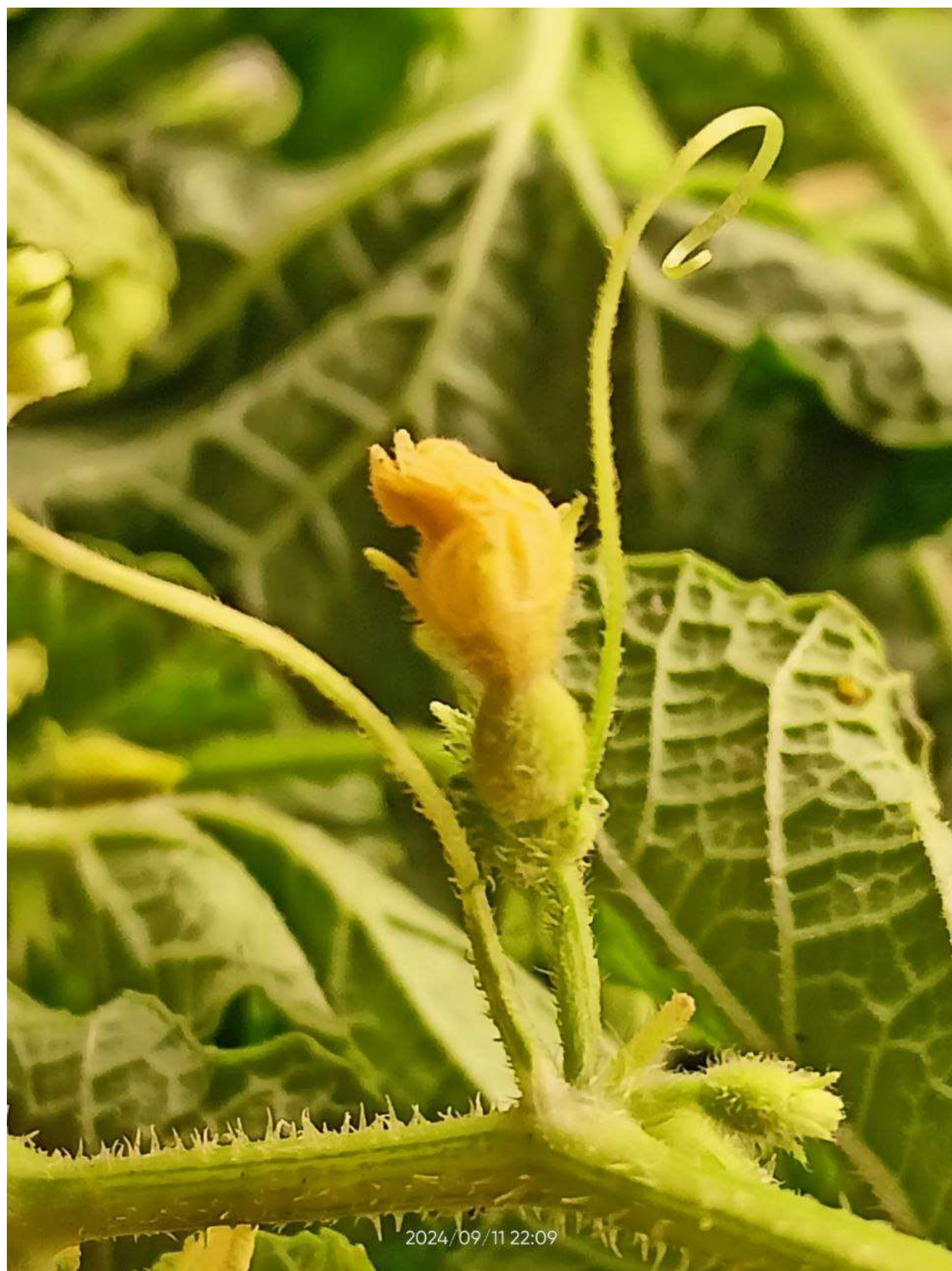

2024/09/11 22:09

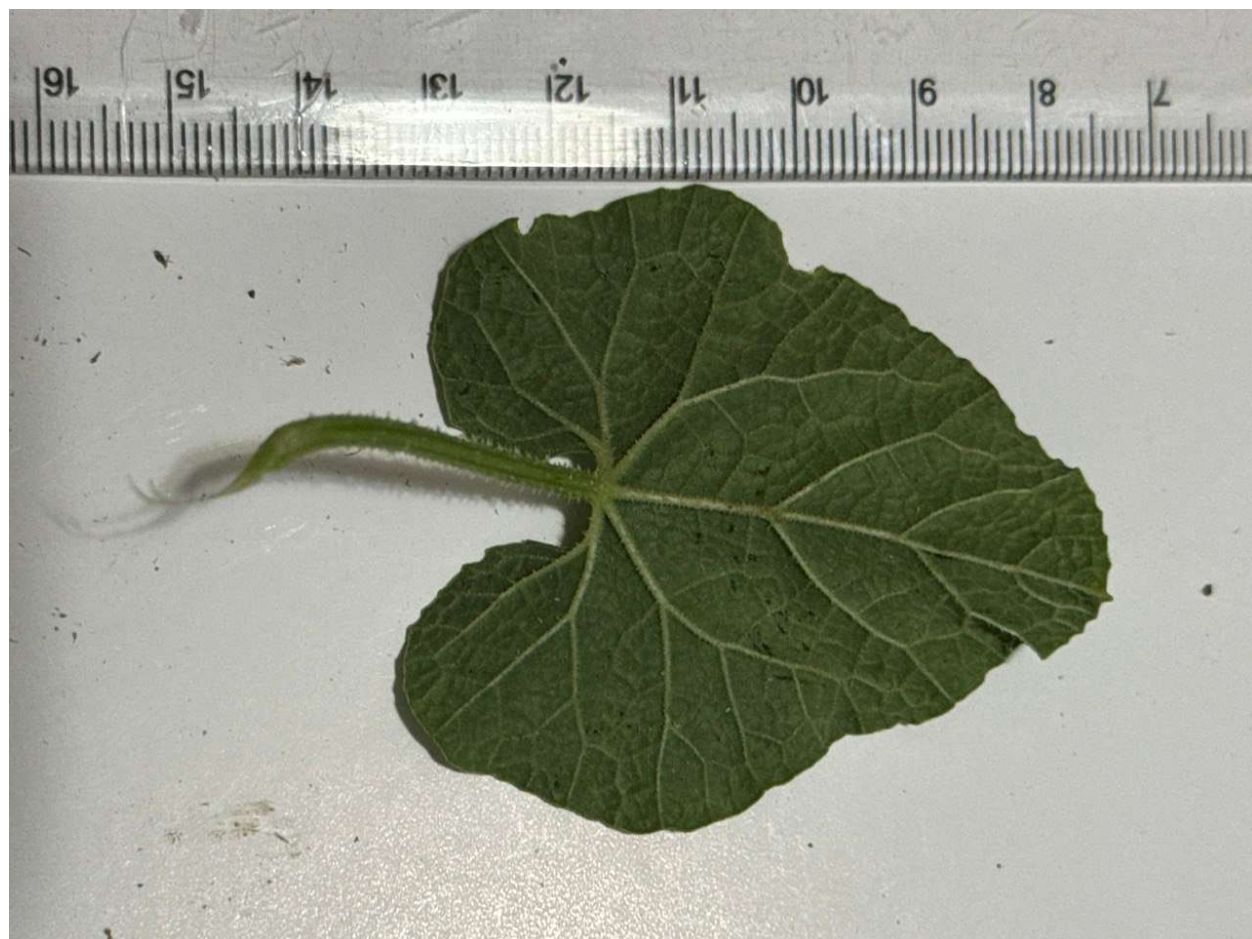

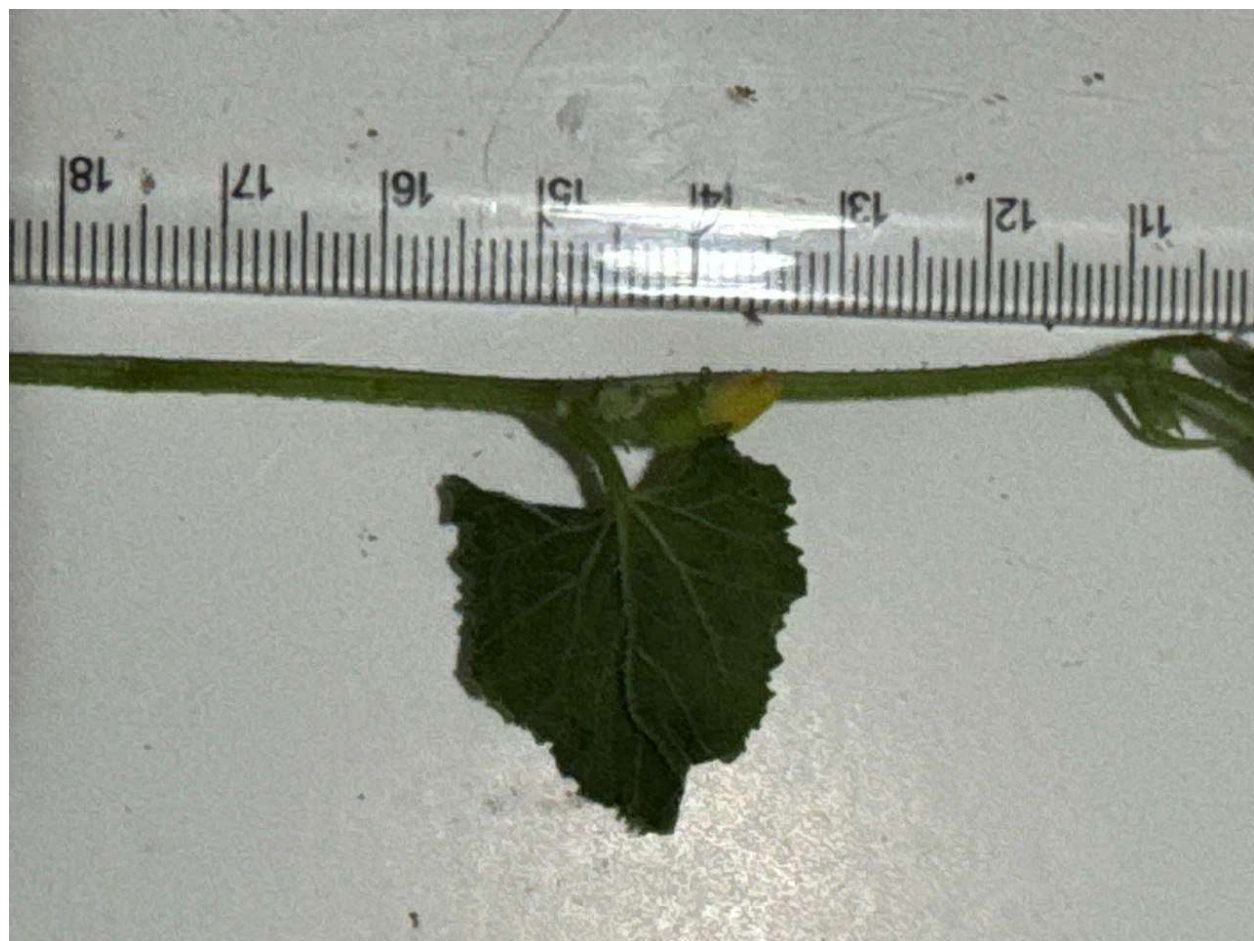

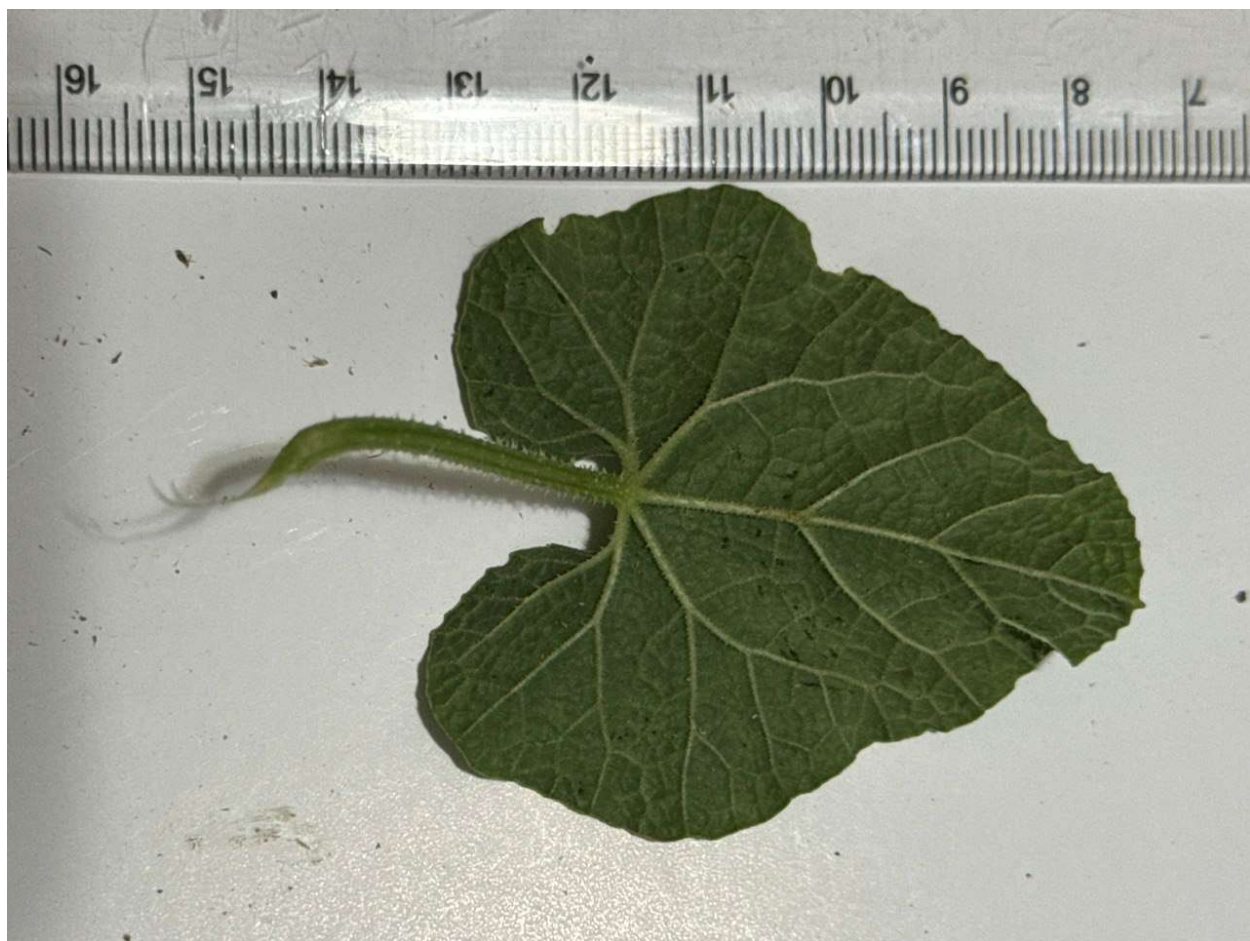

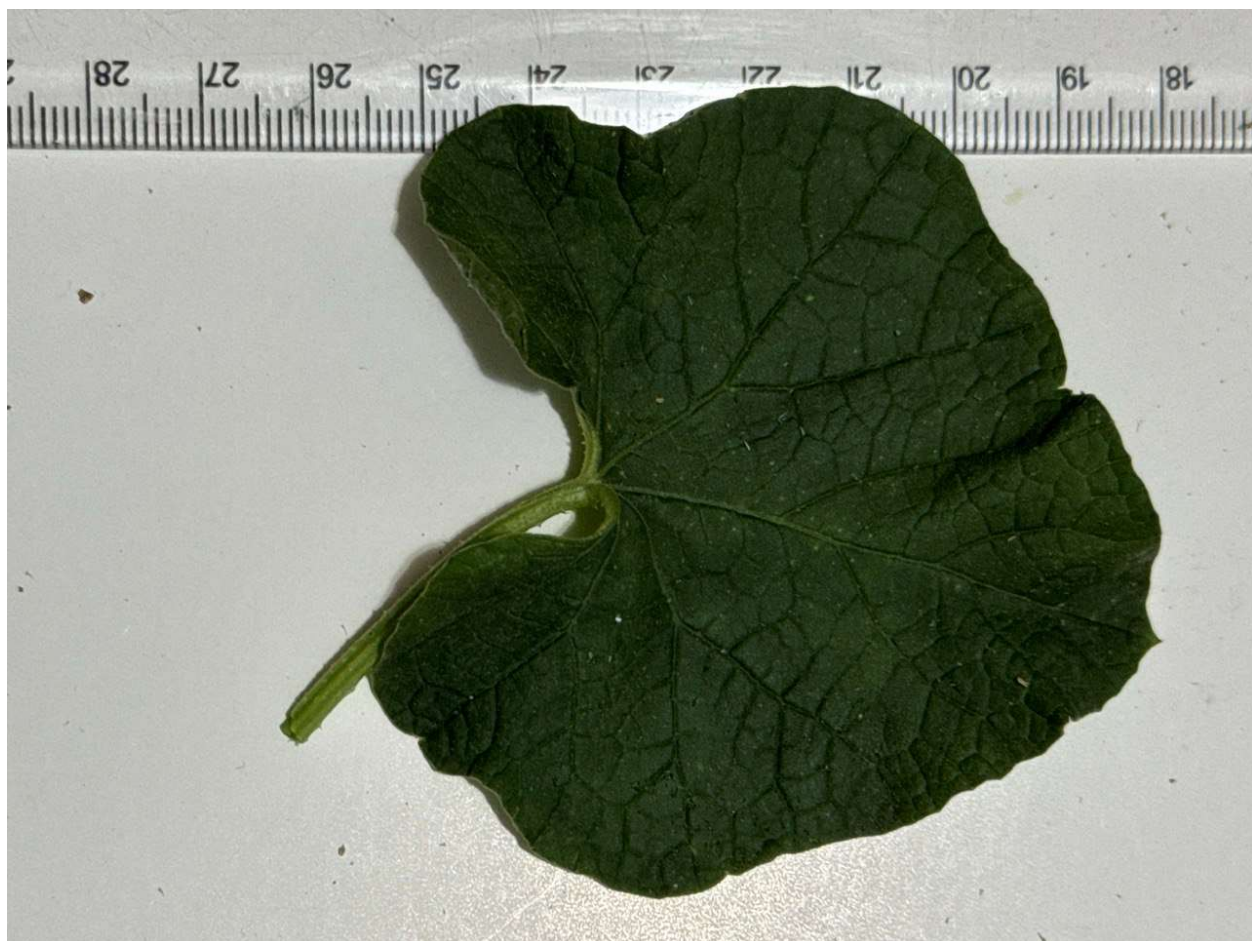

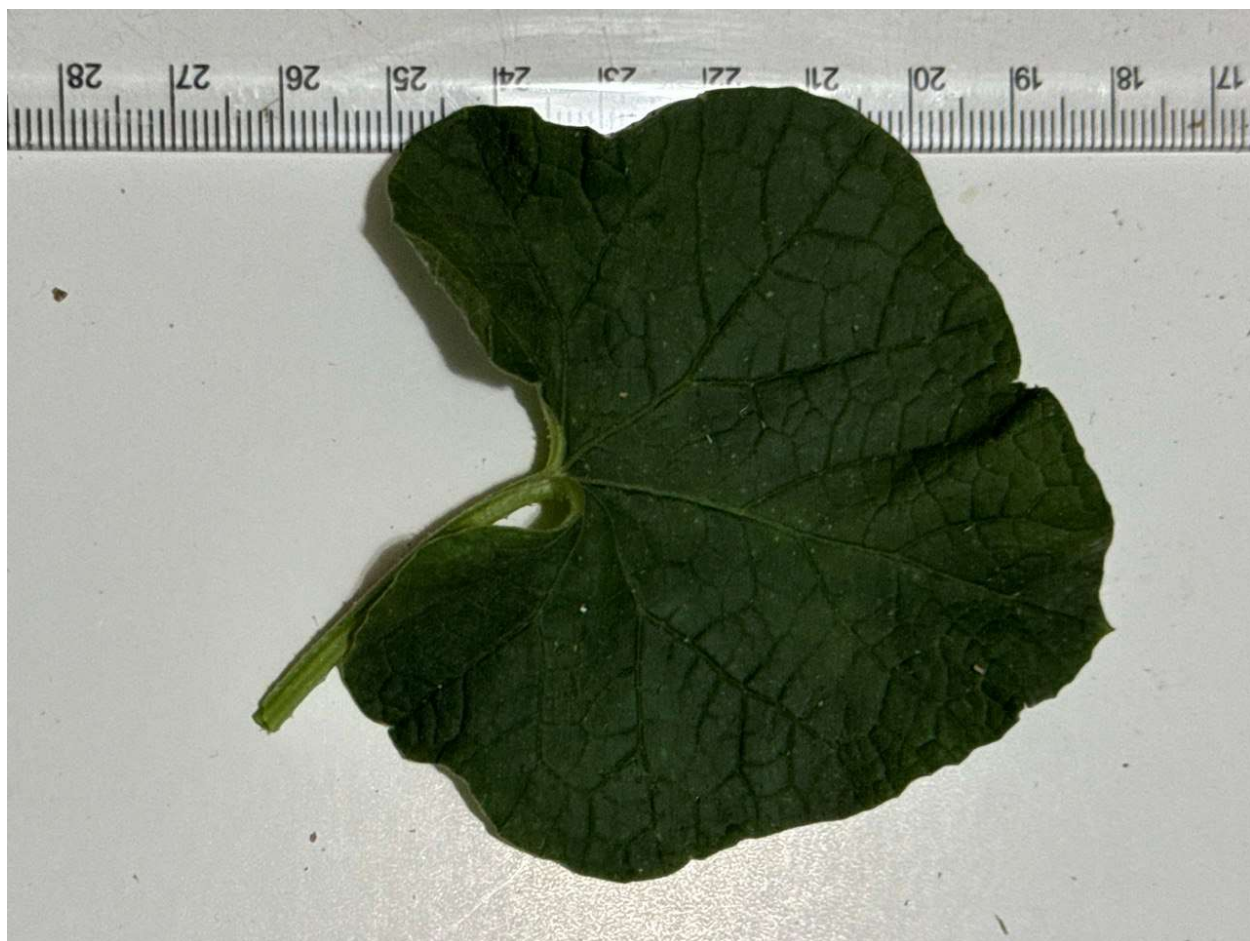

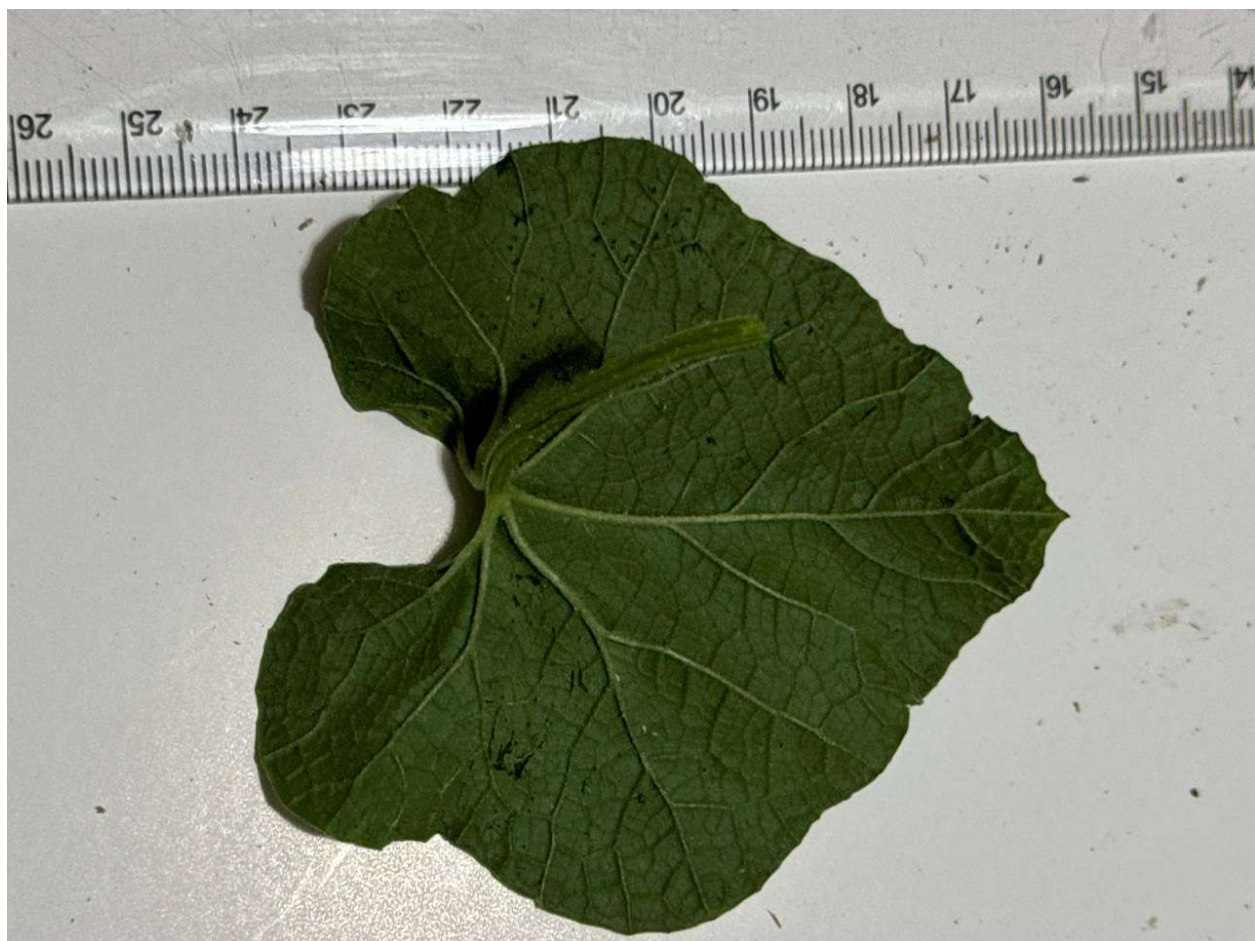

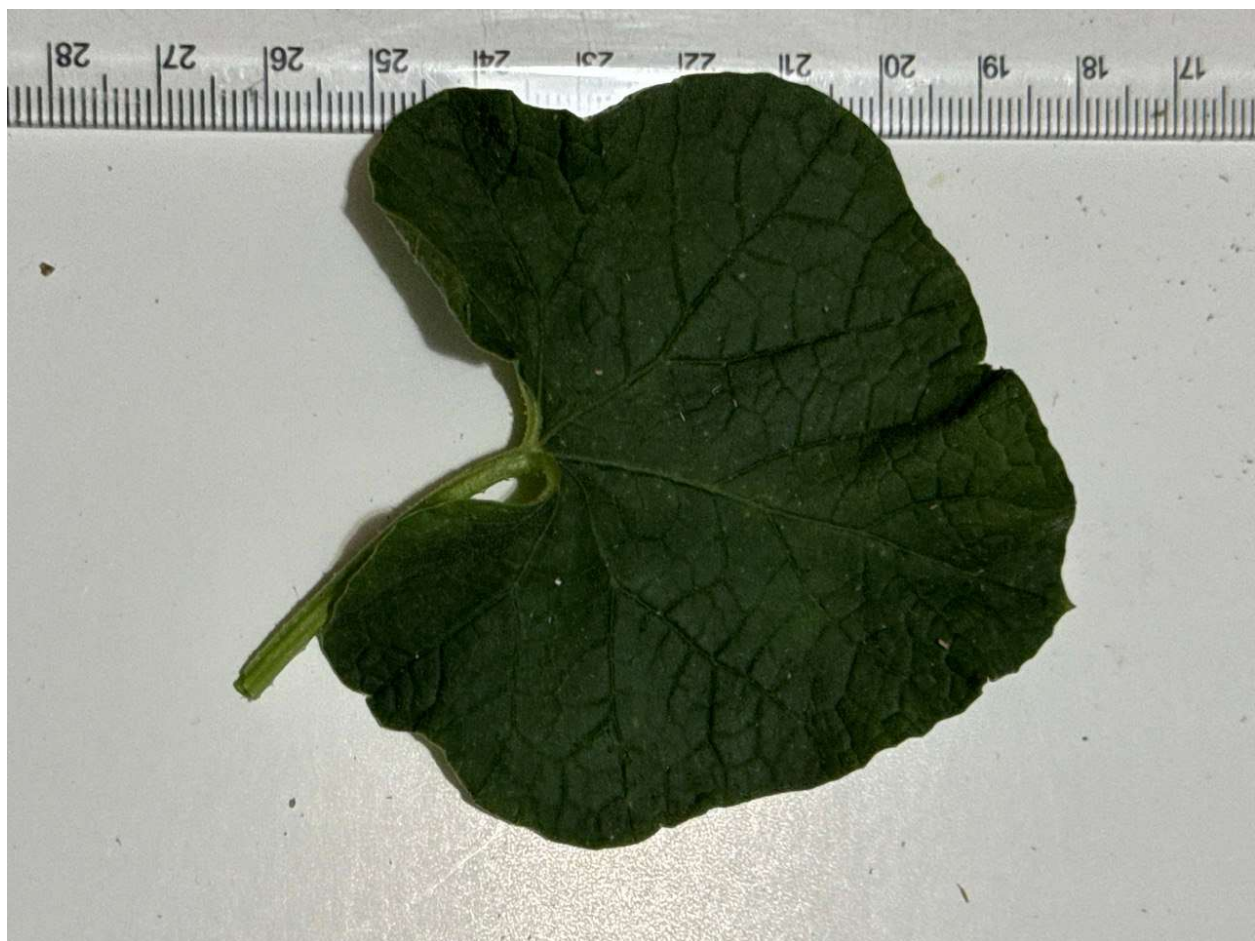

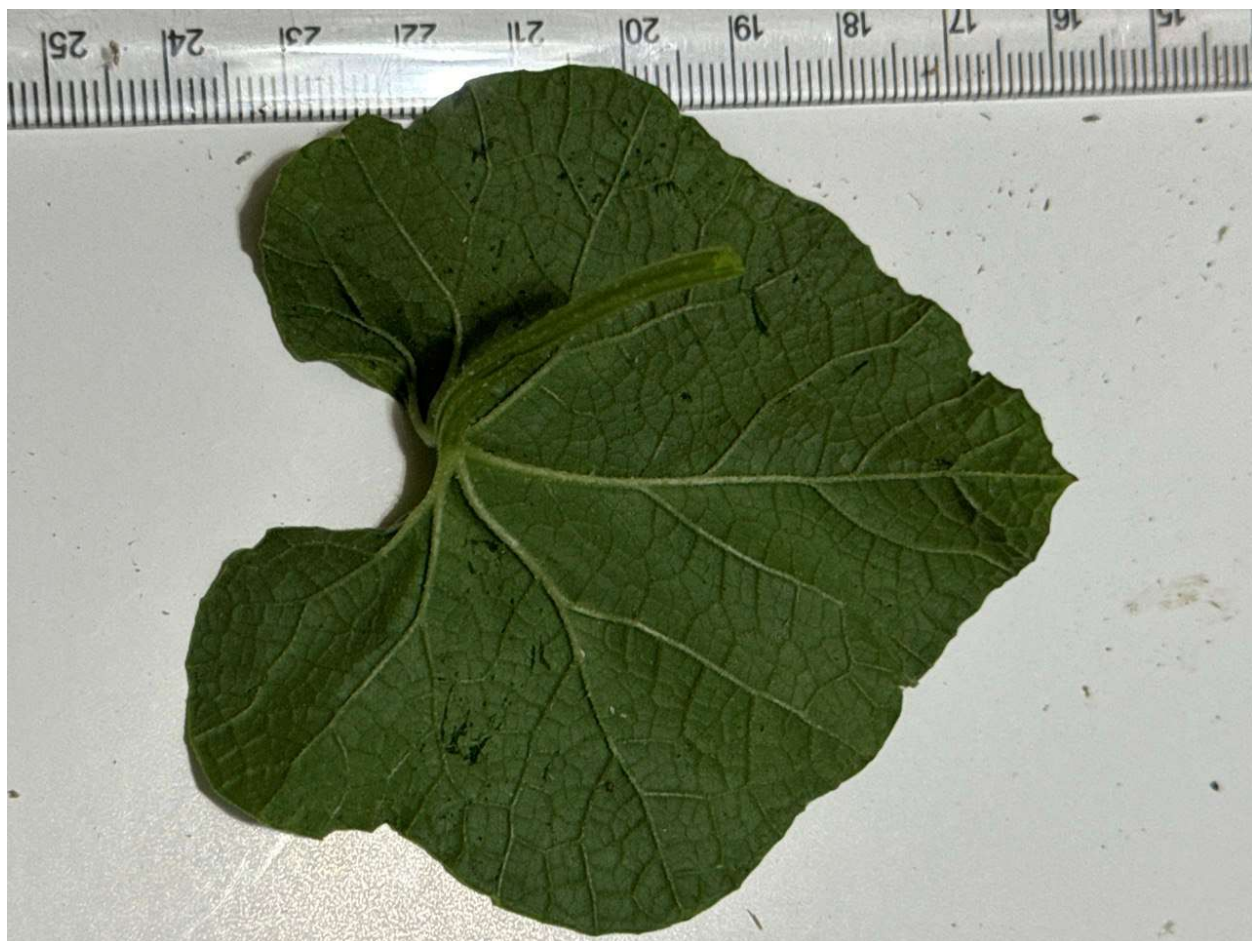

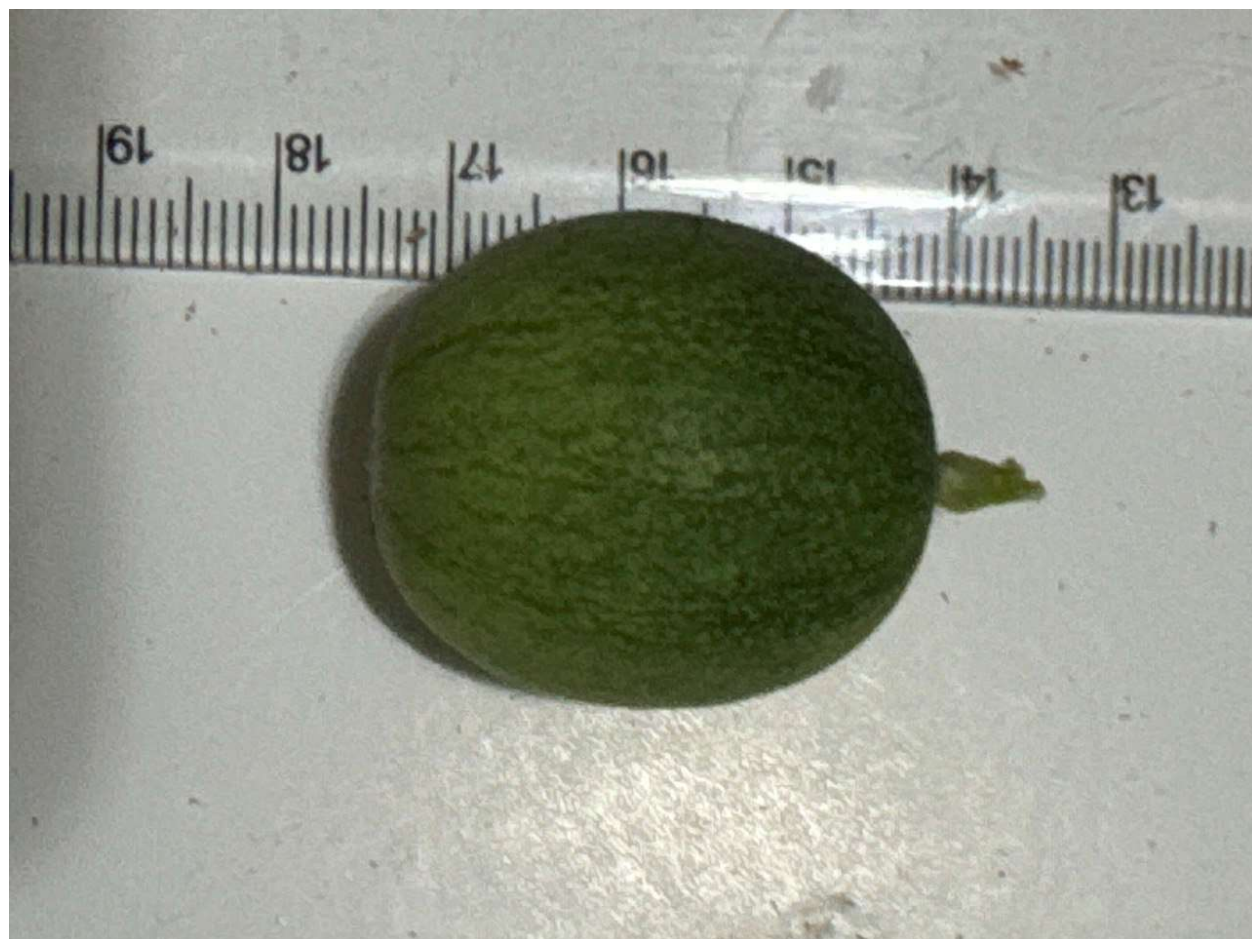

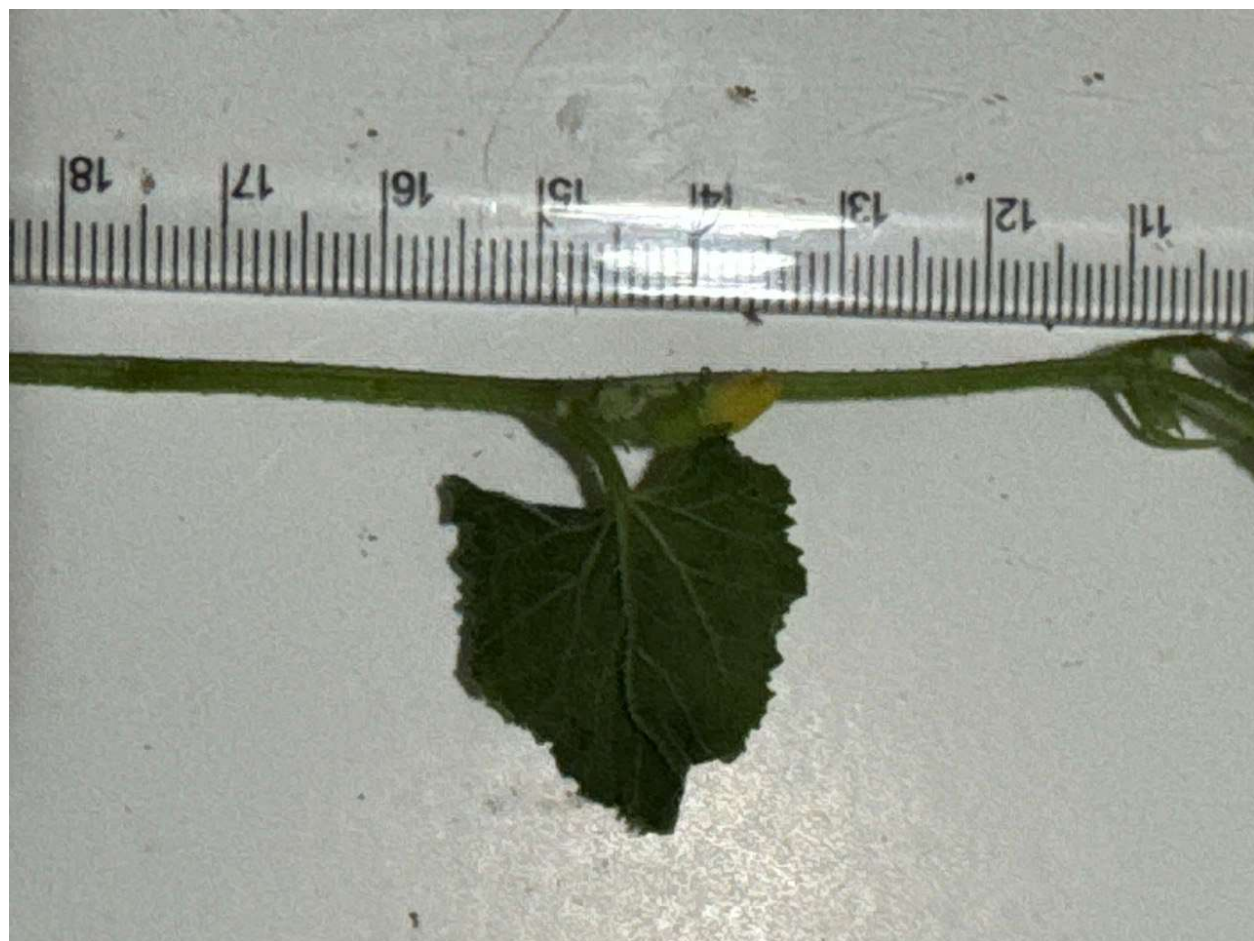

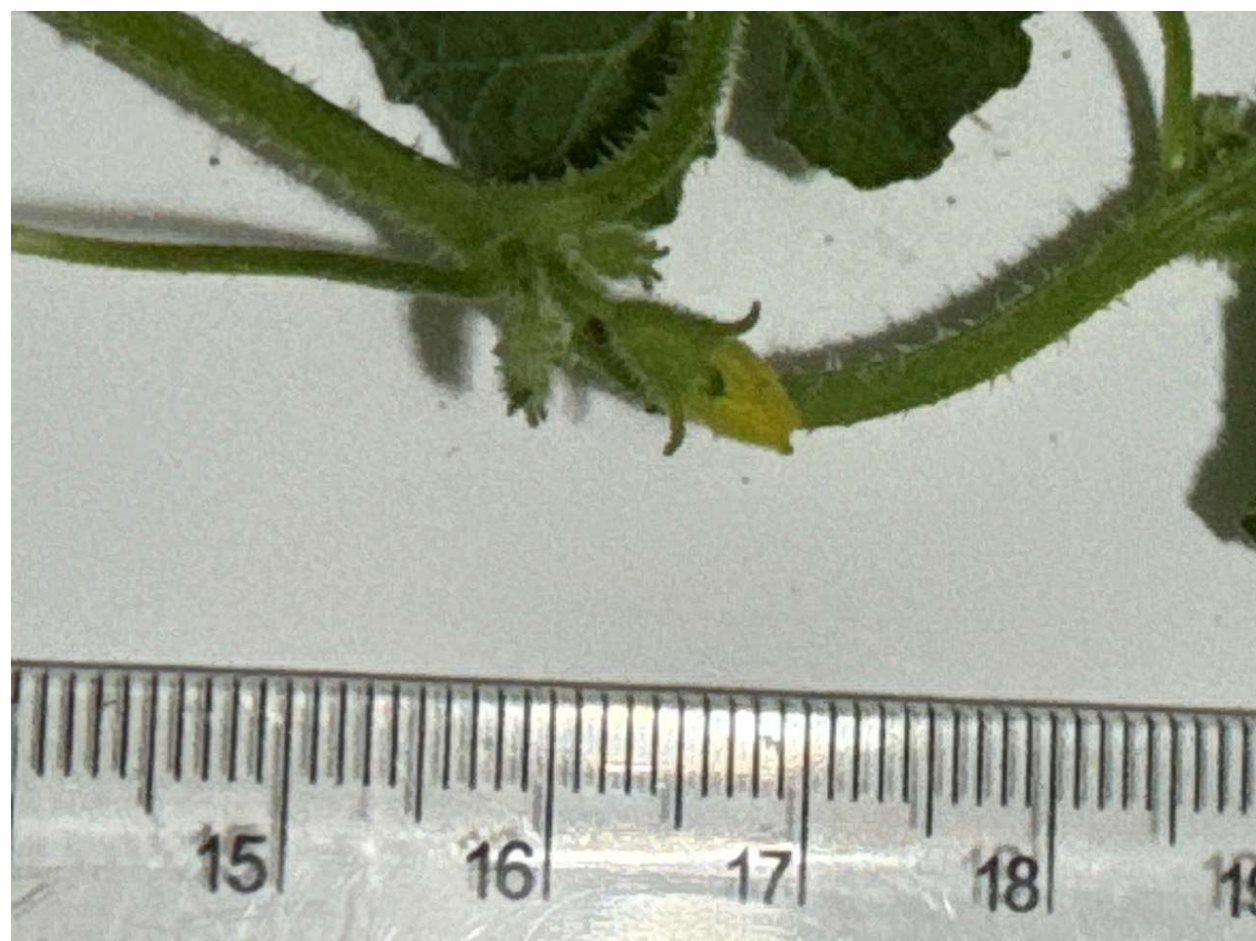

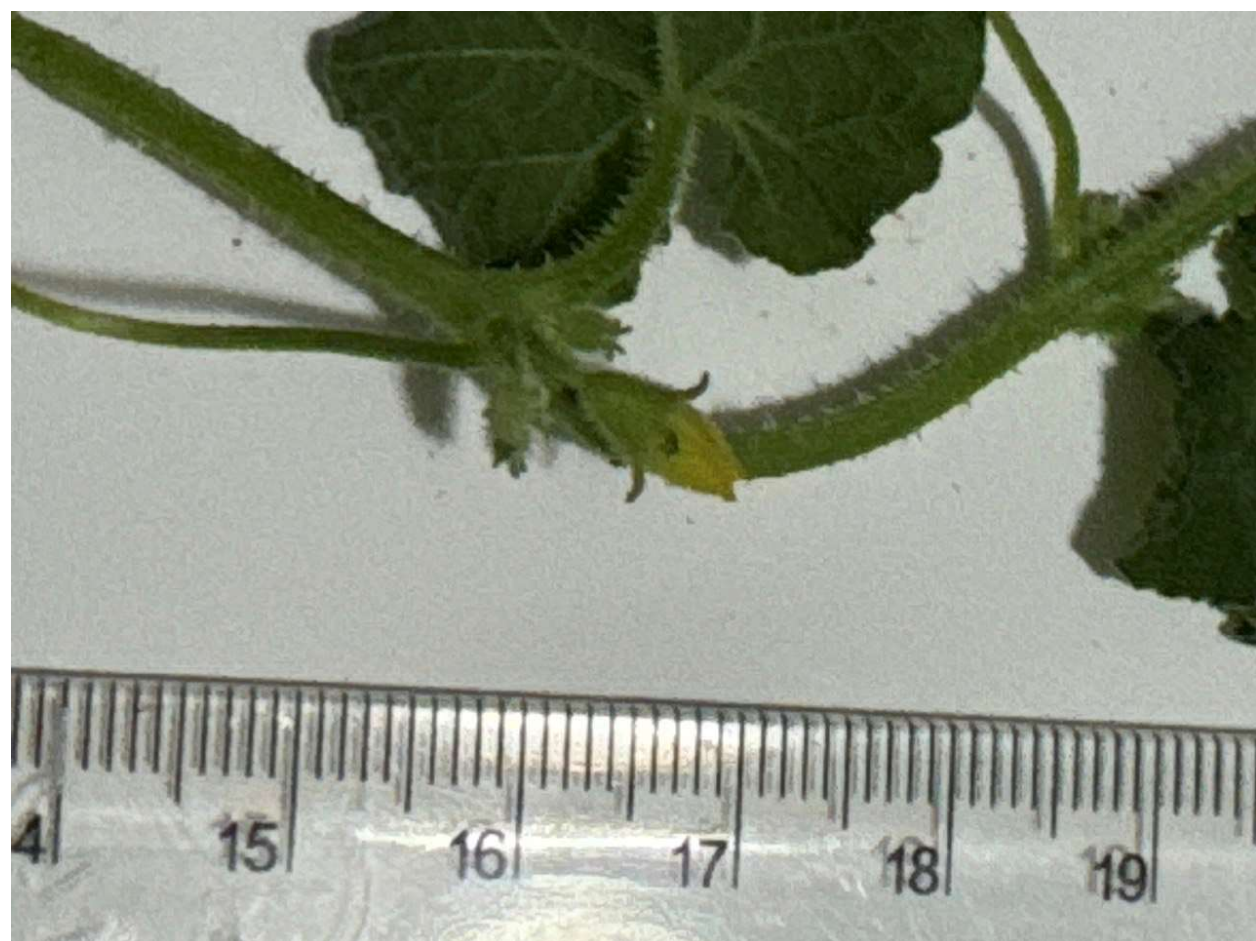

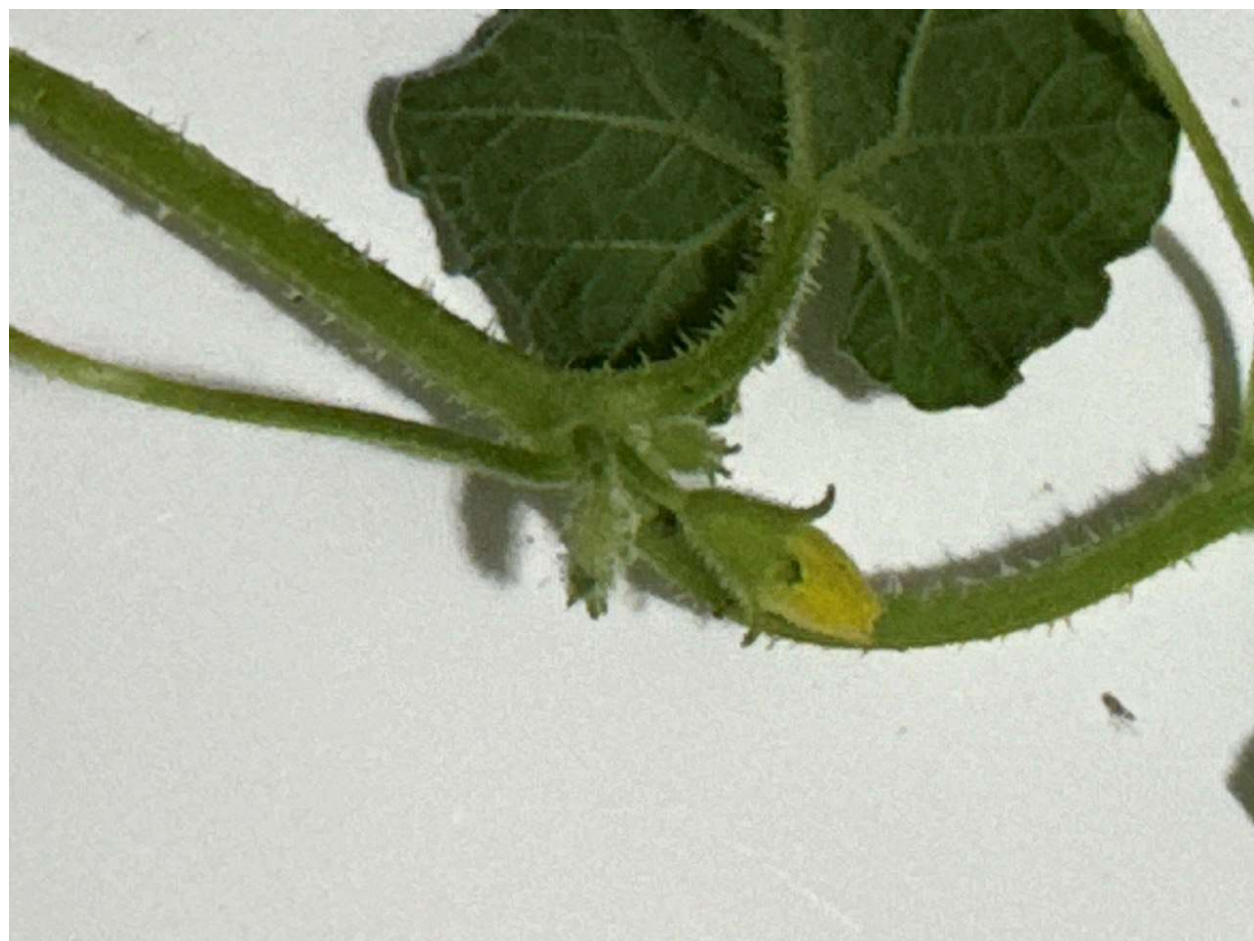

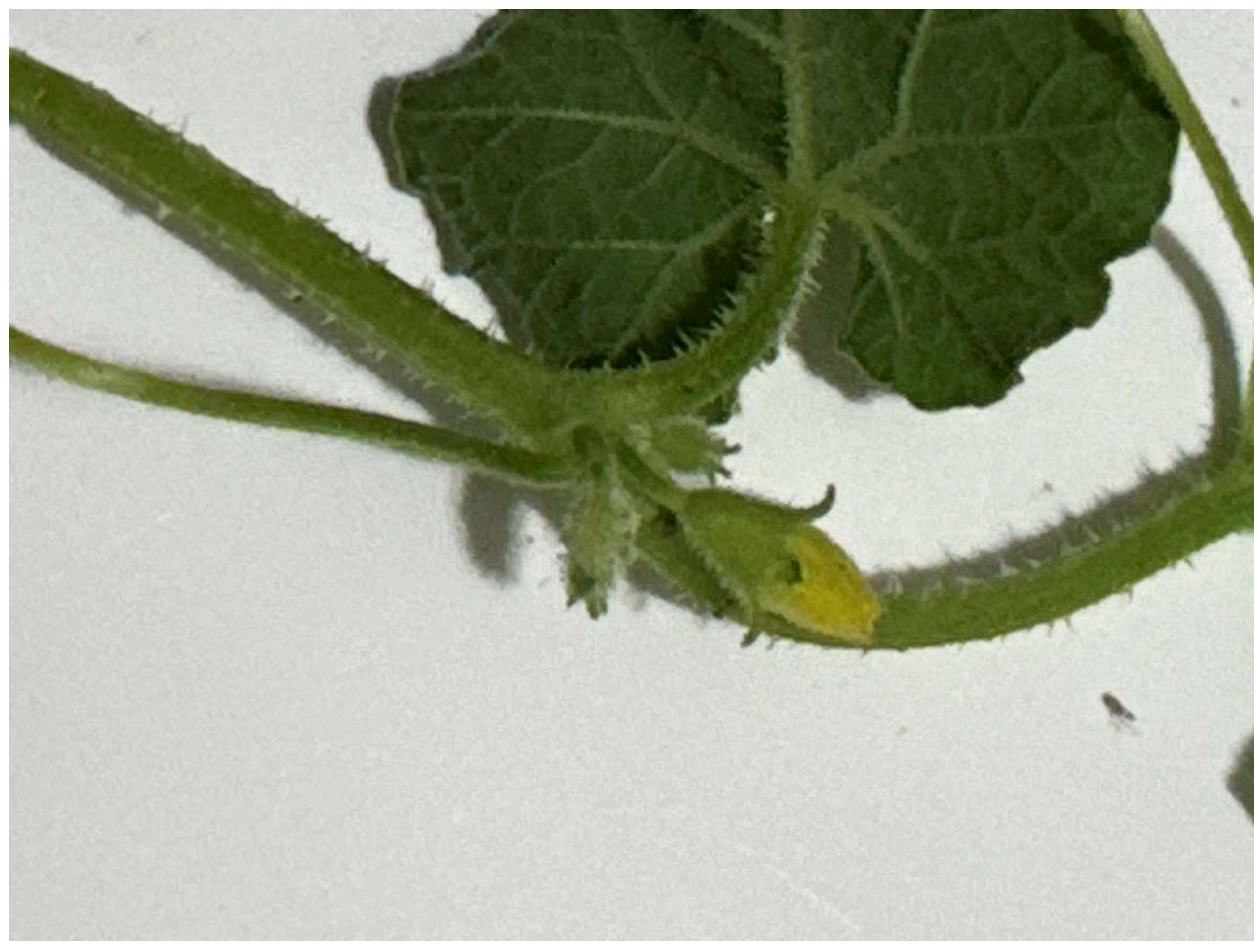

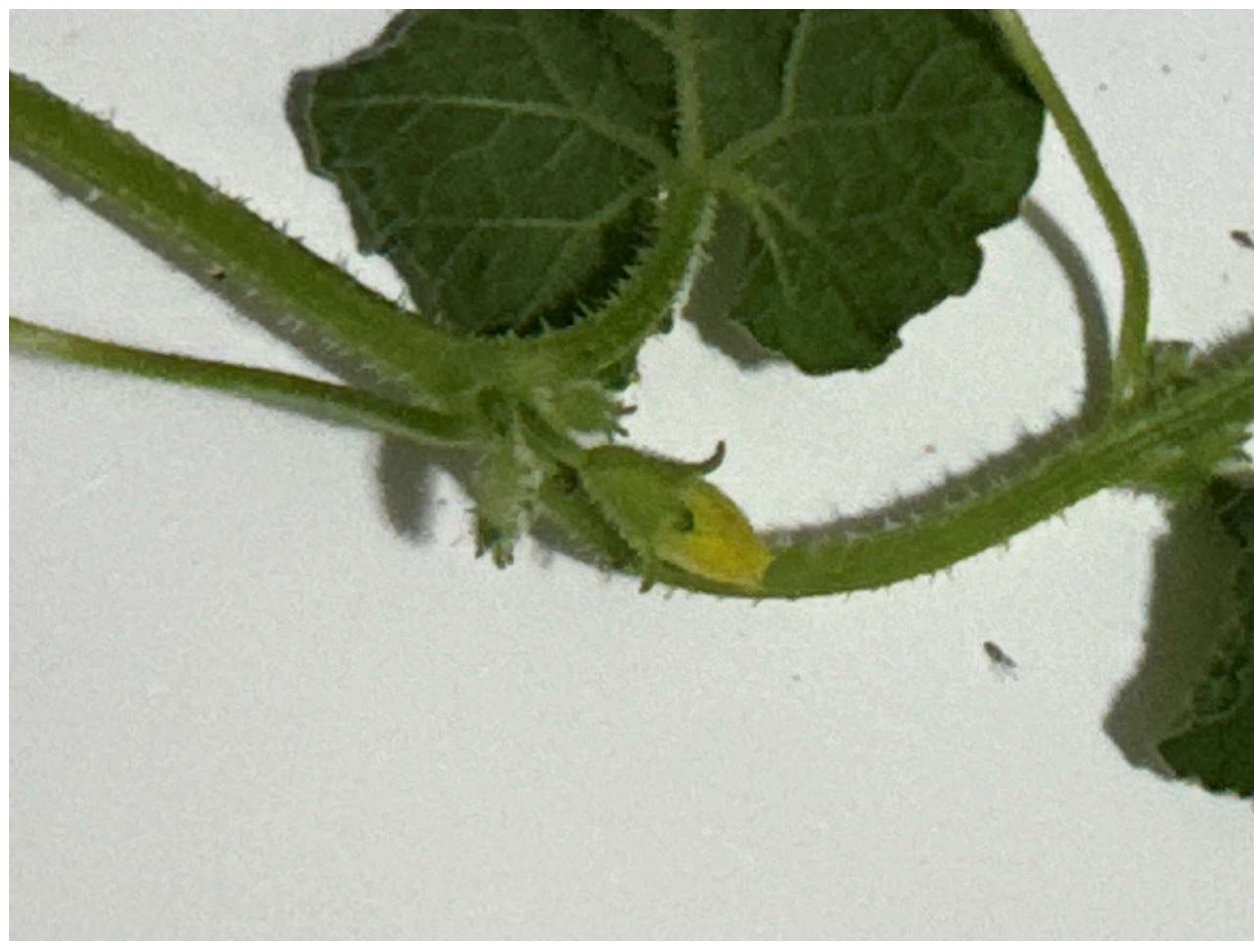

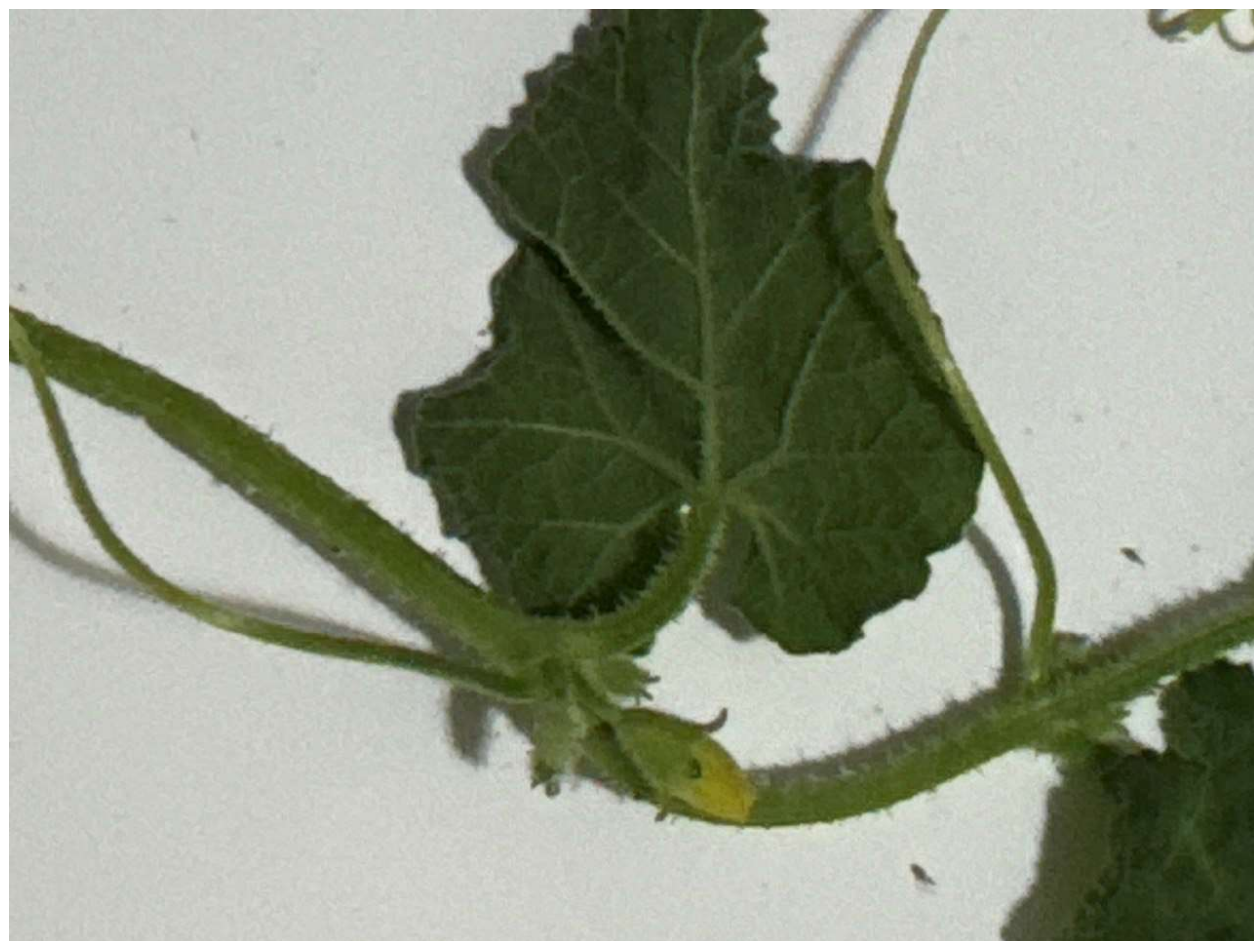

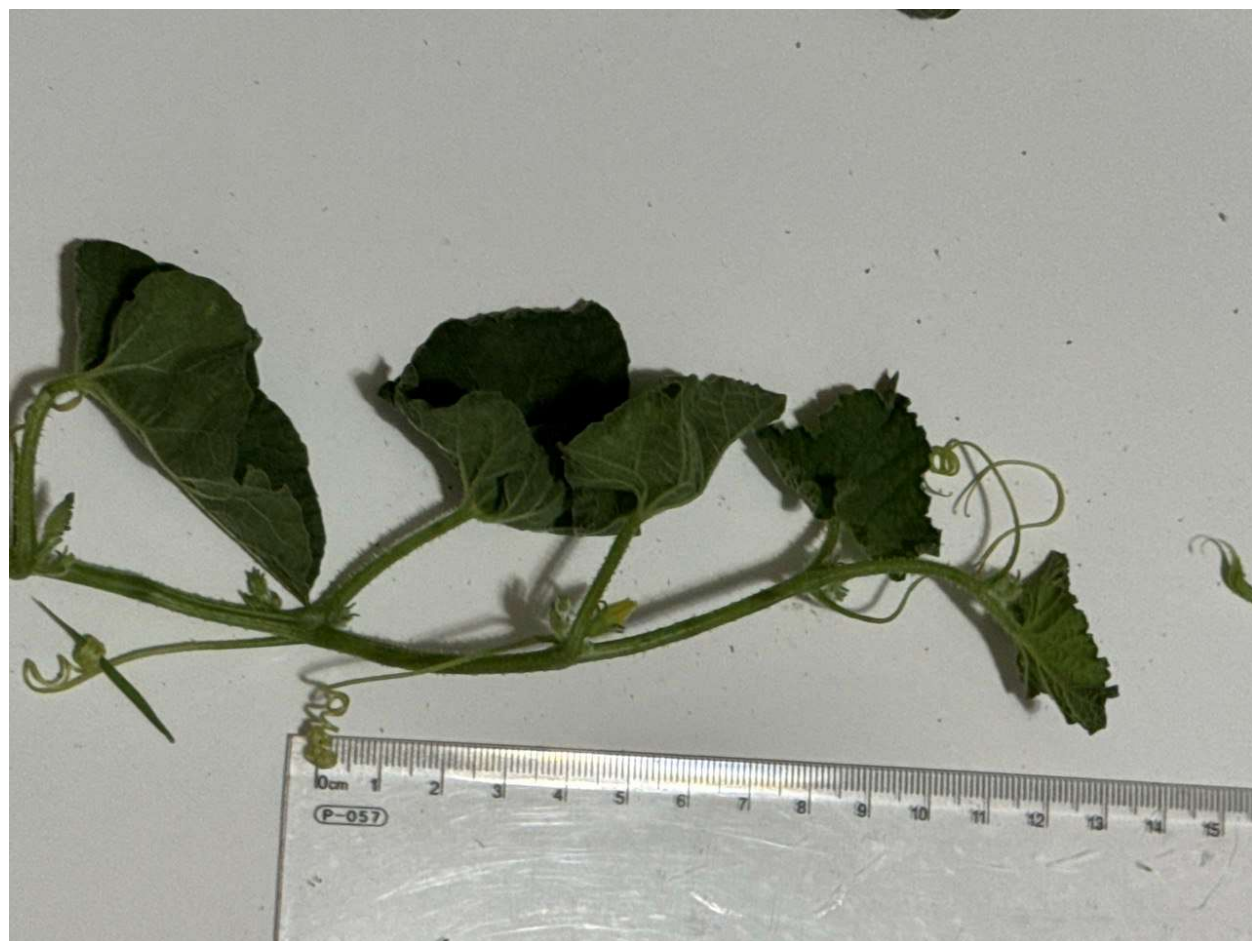

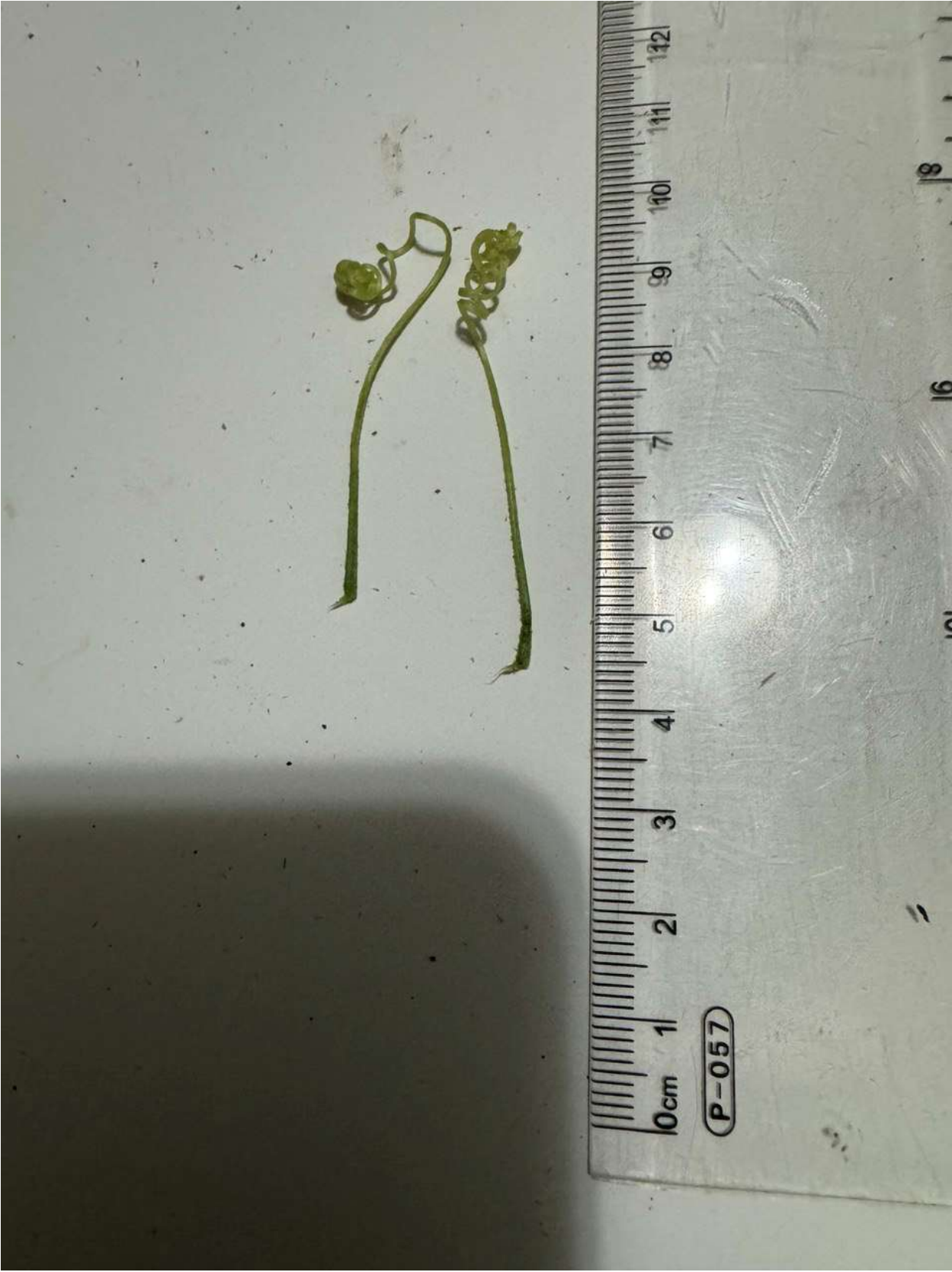

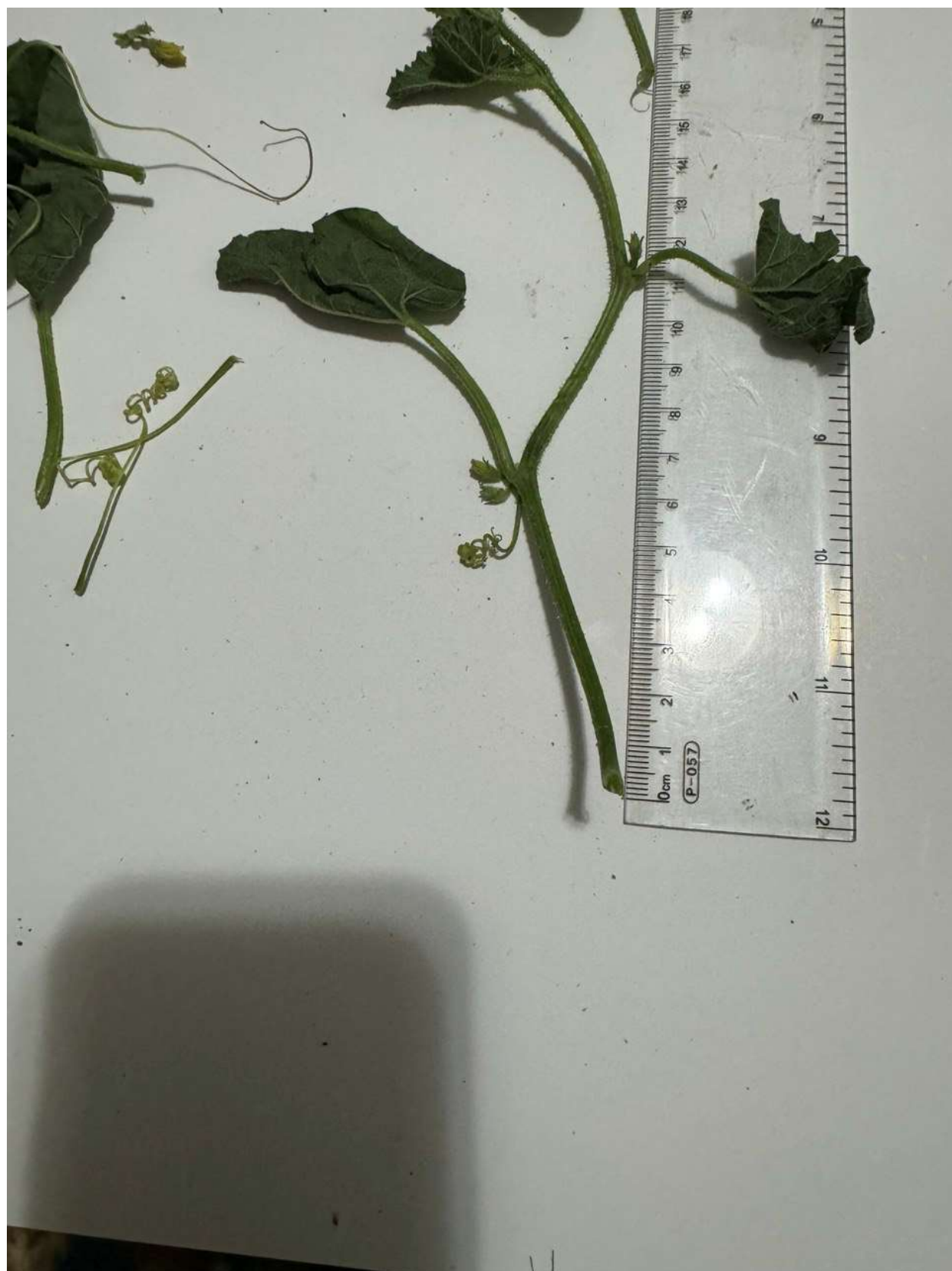

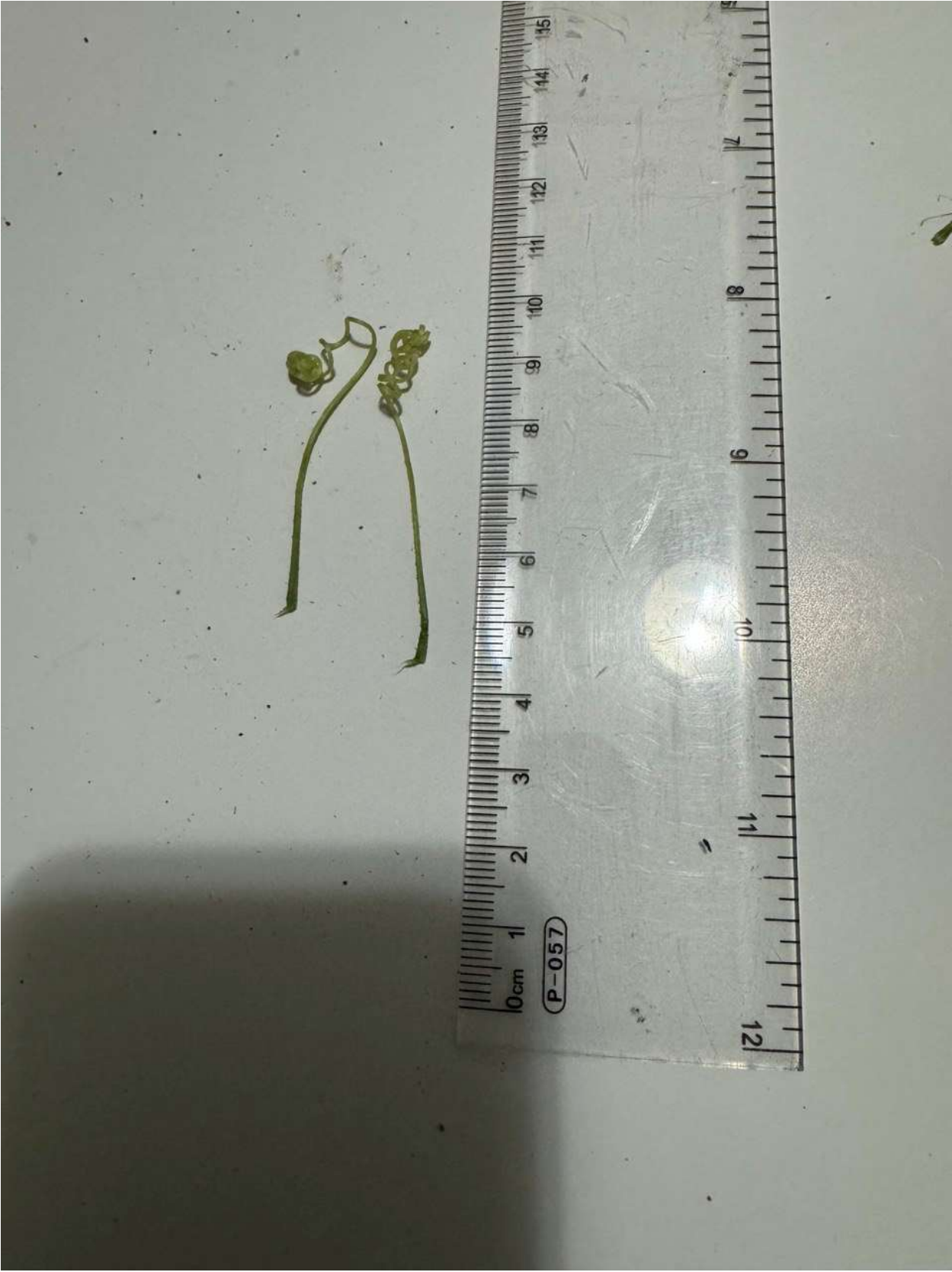

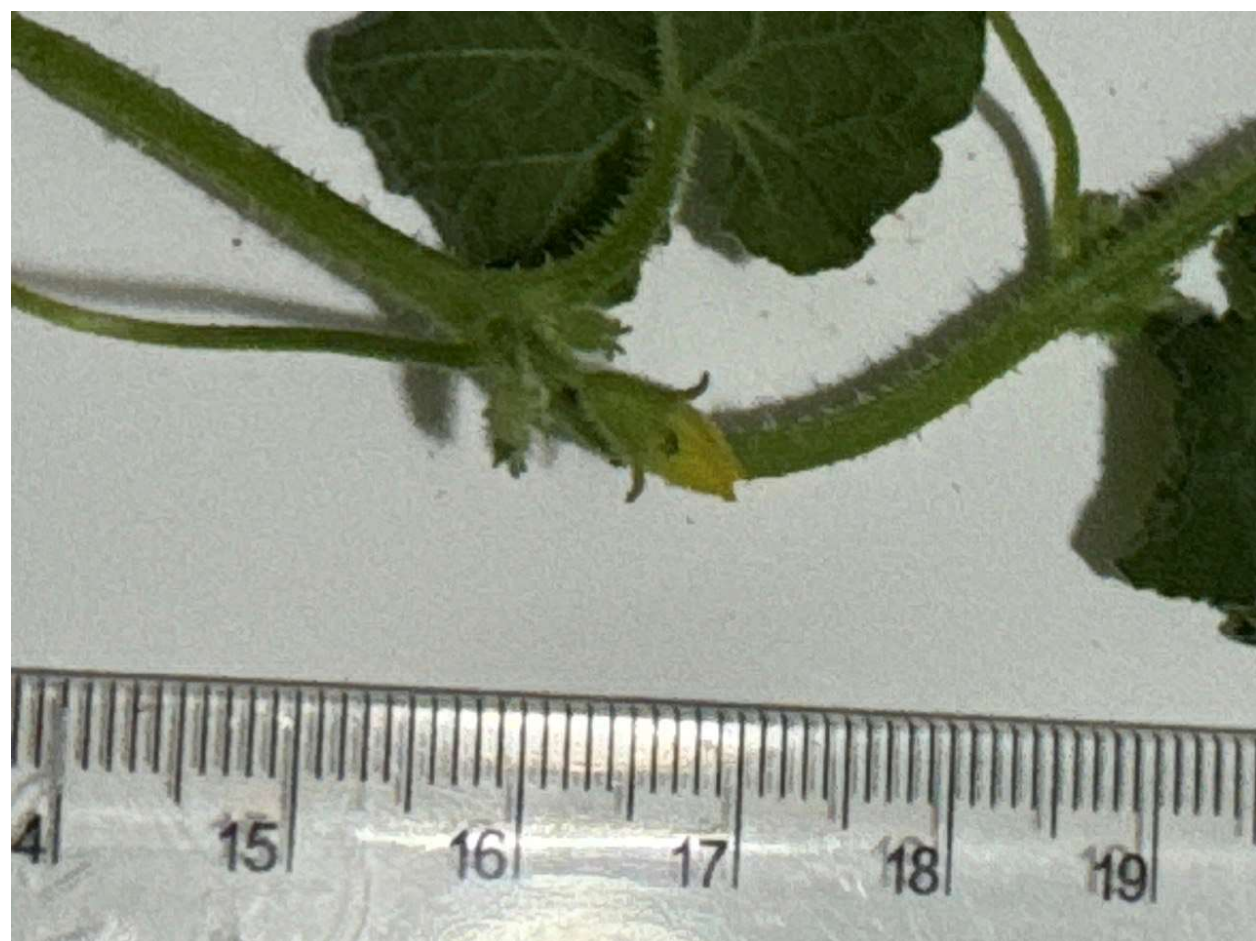

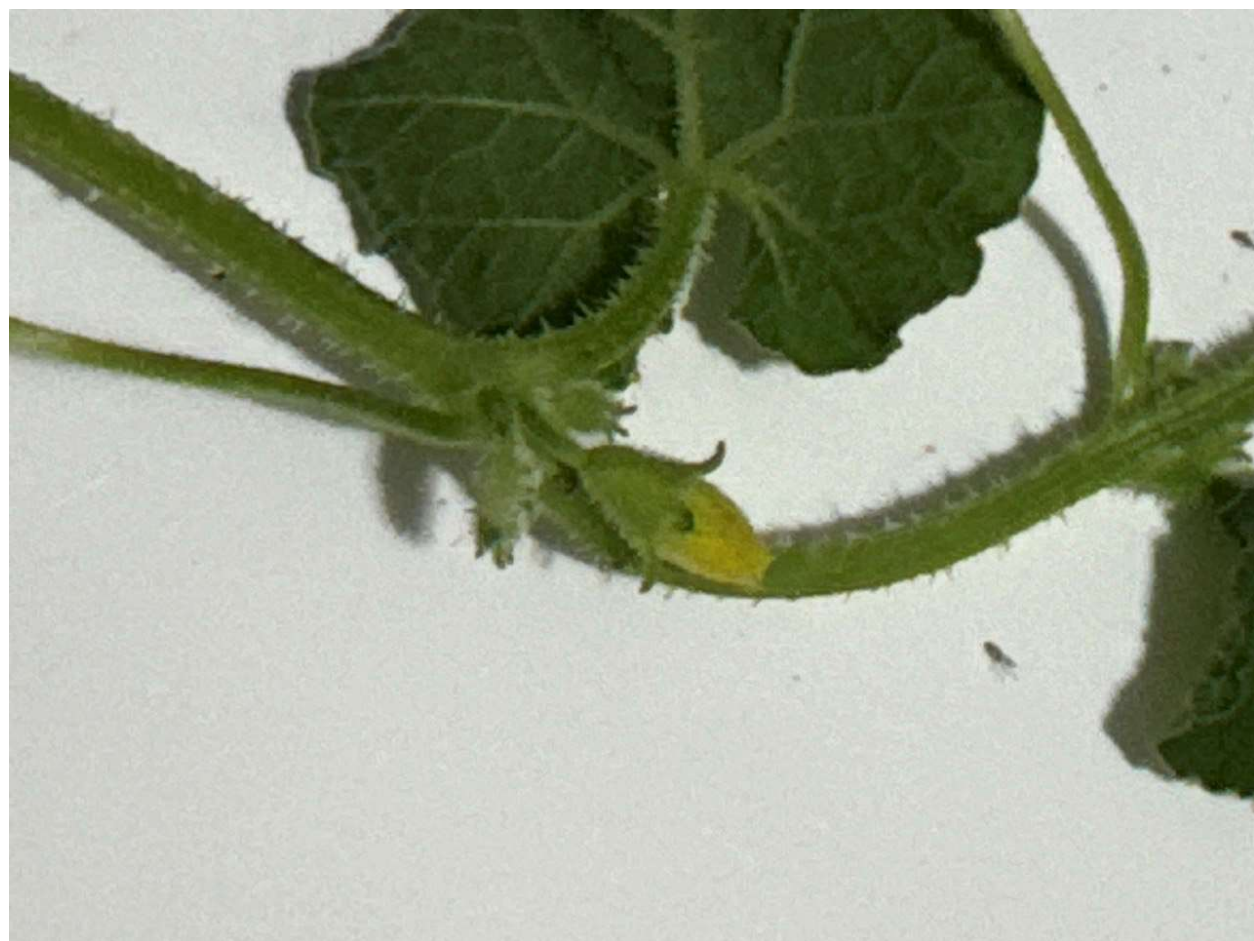

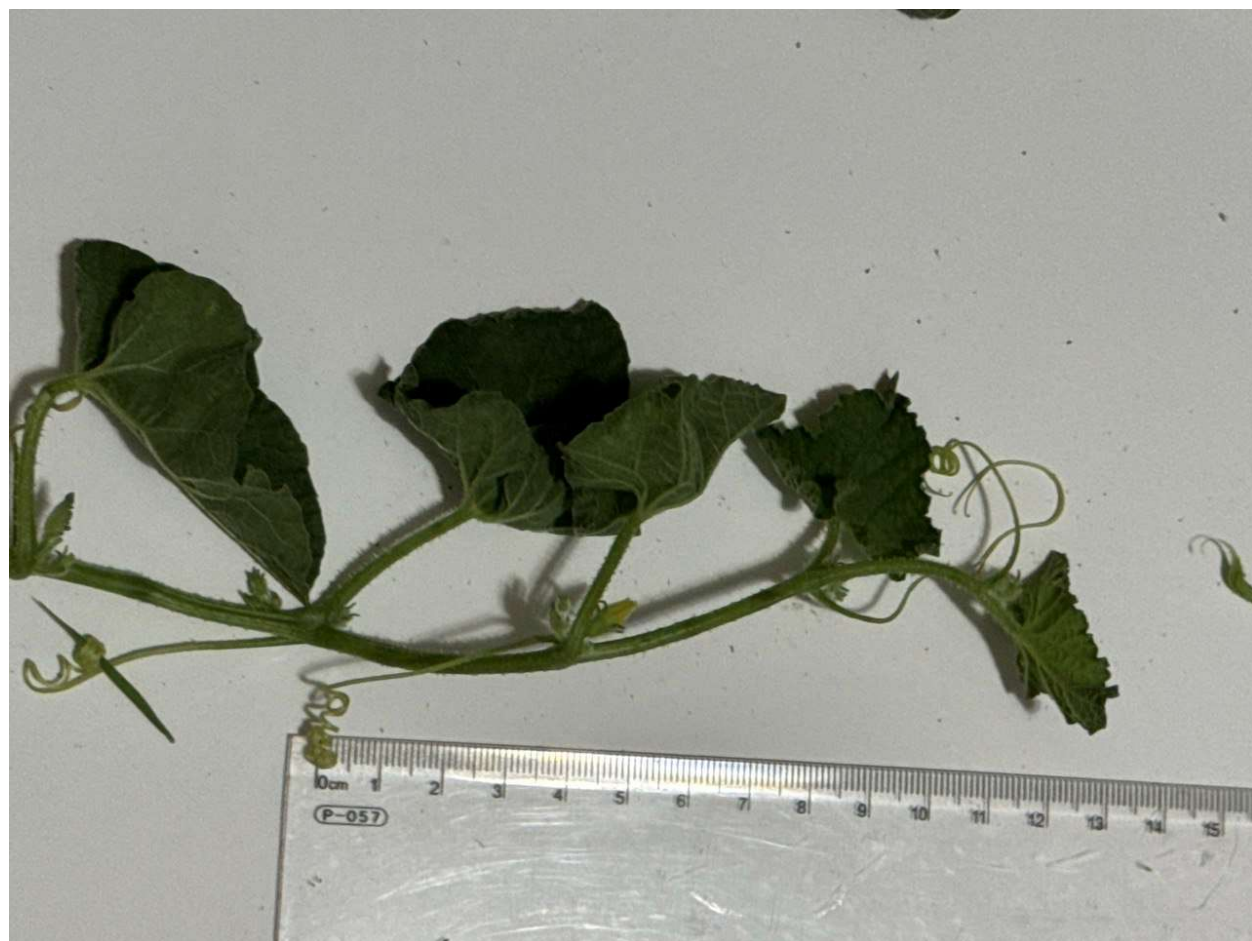

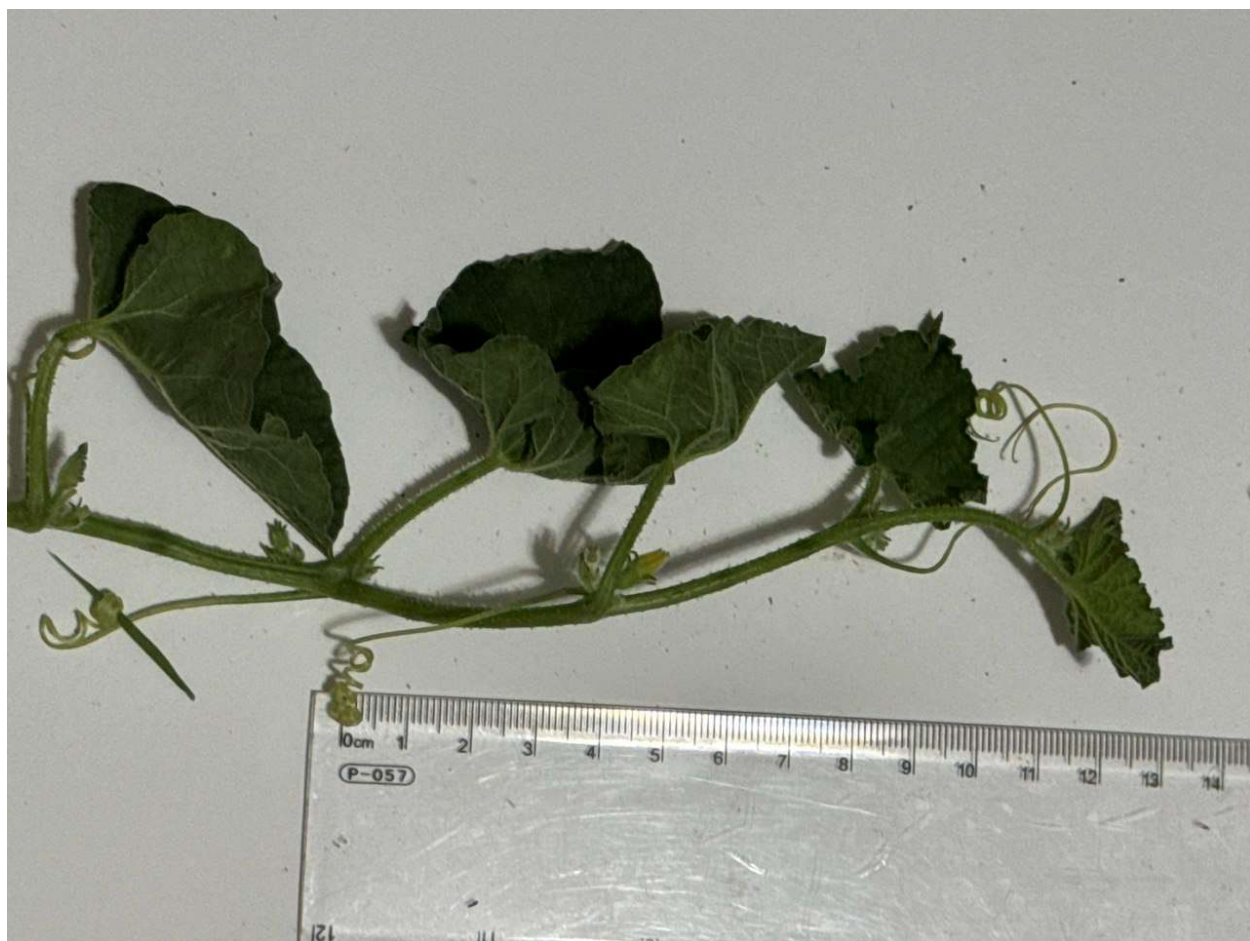

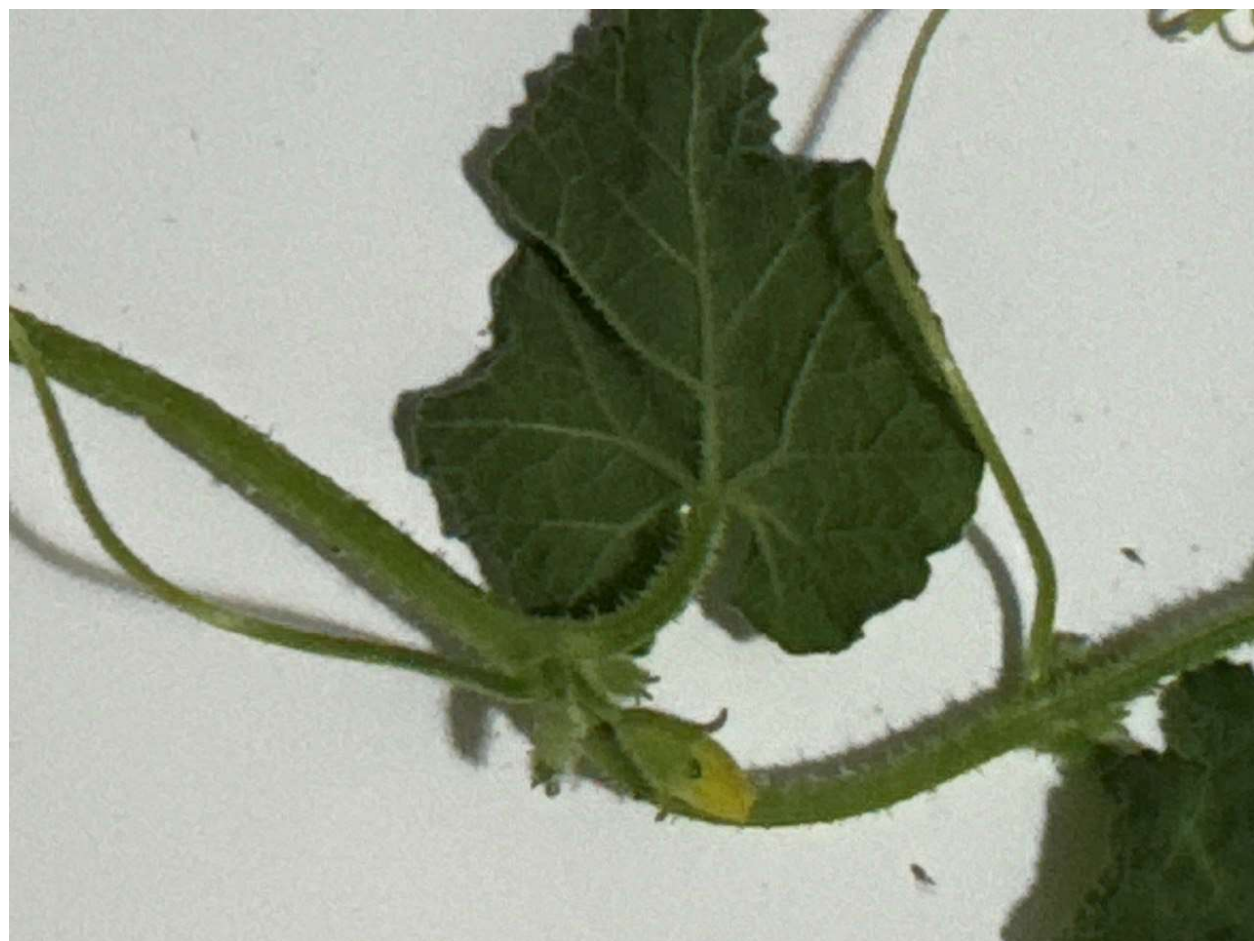

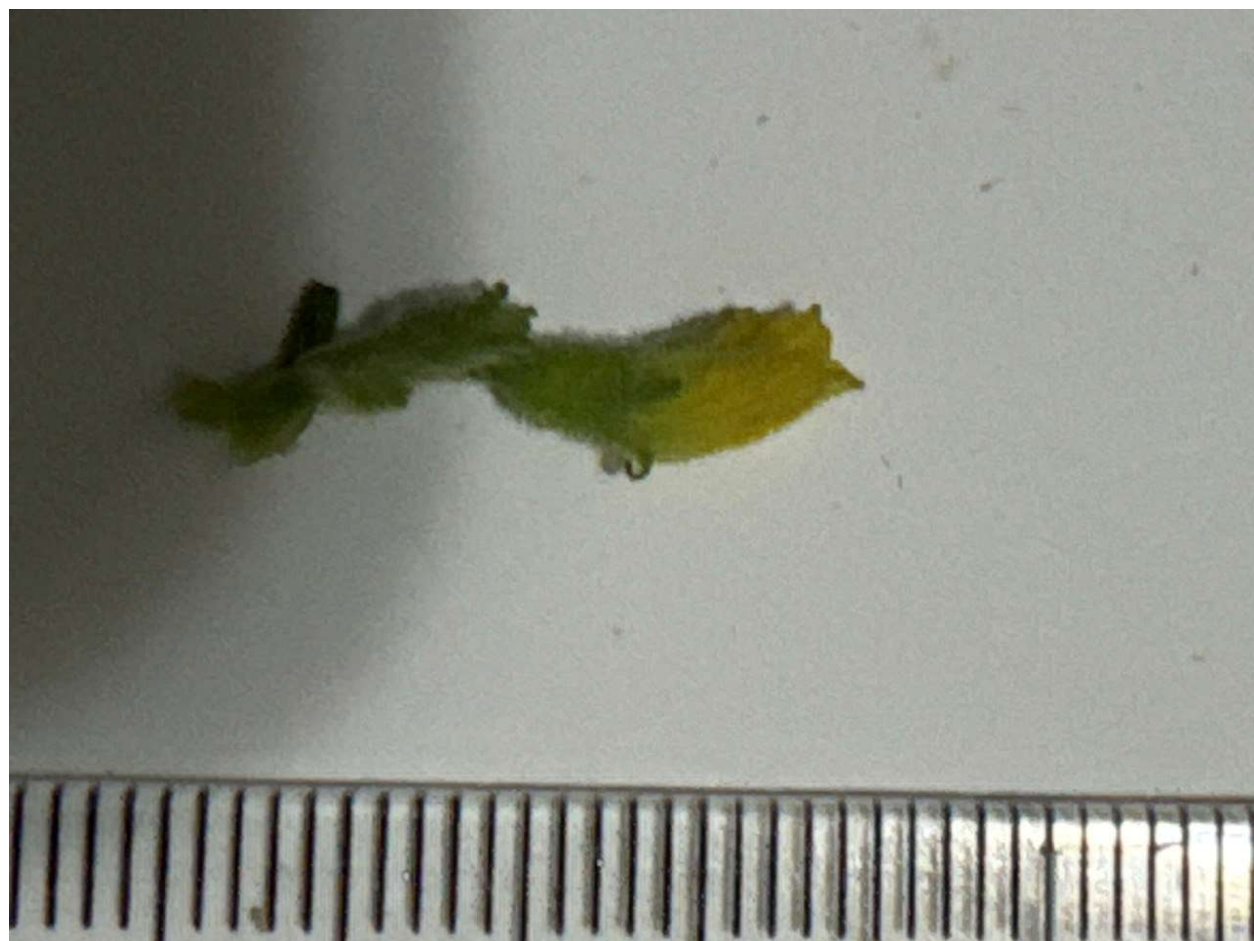

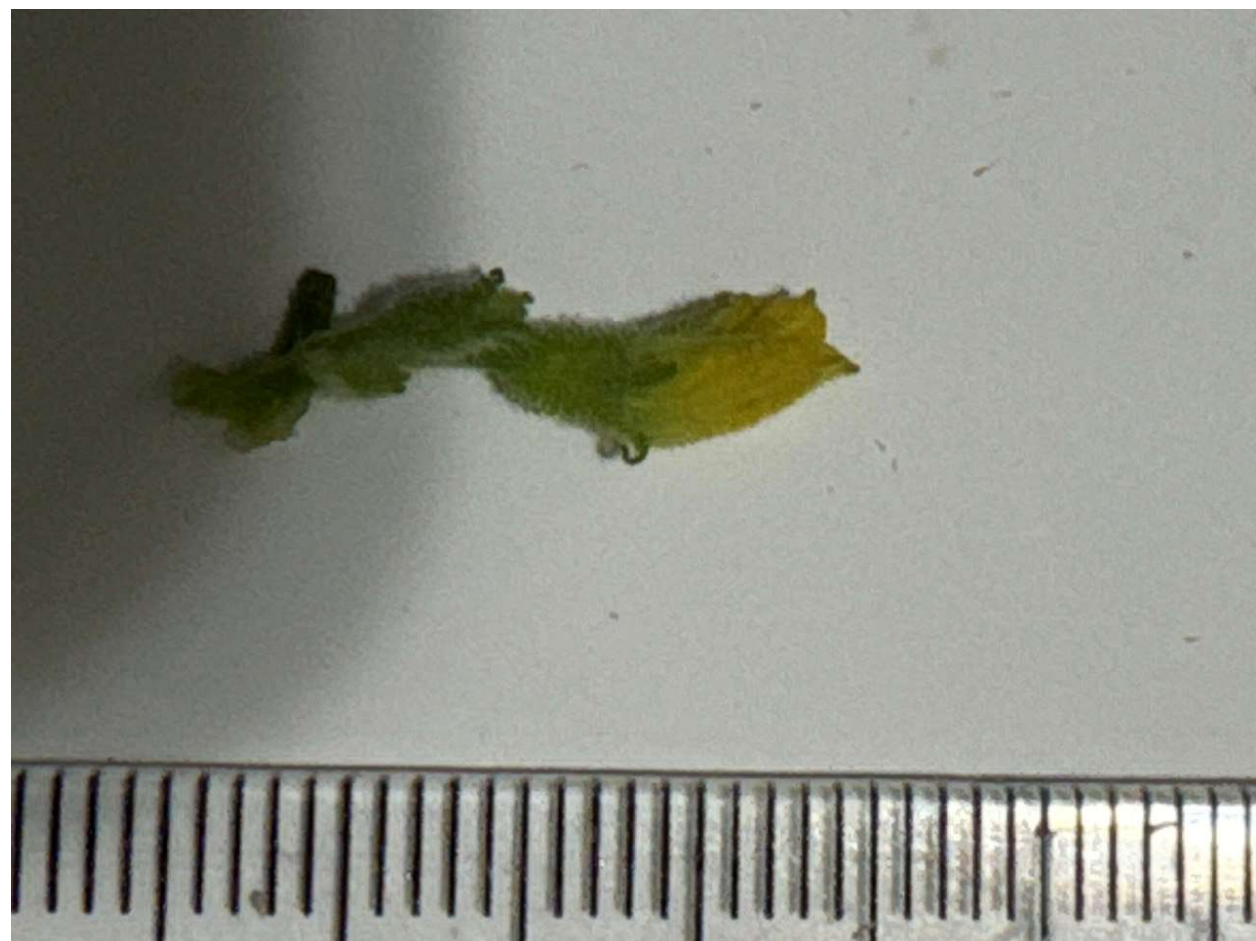

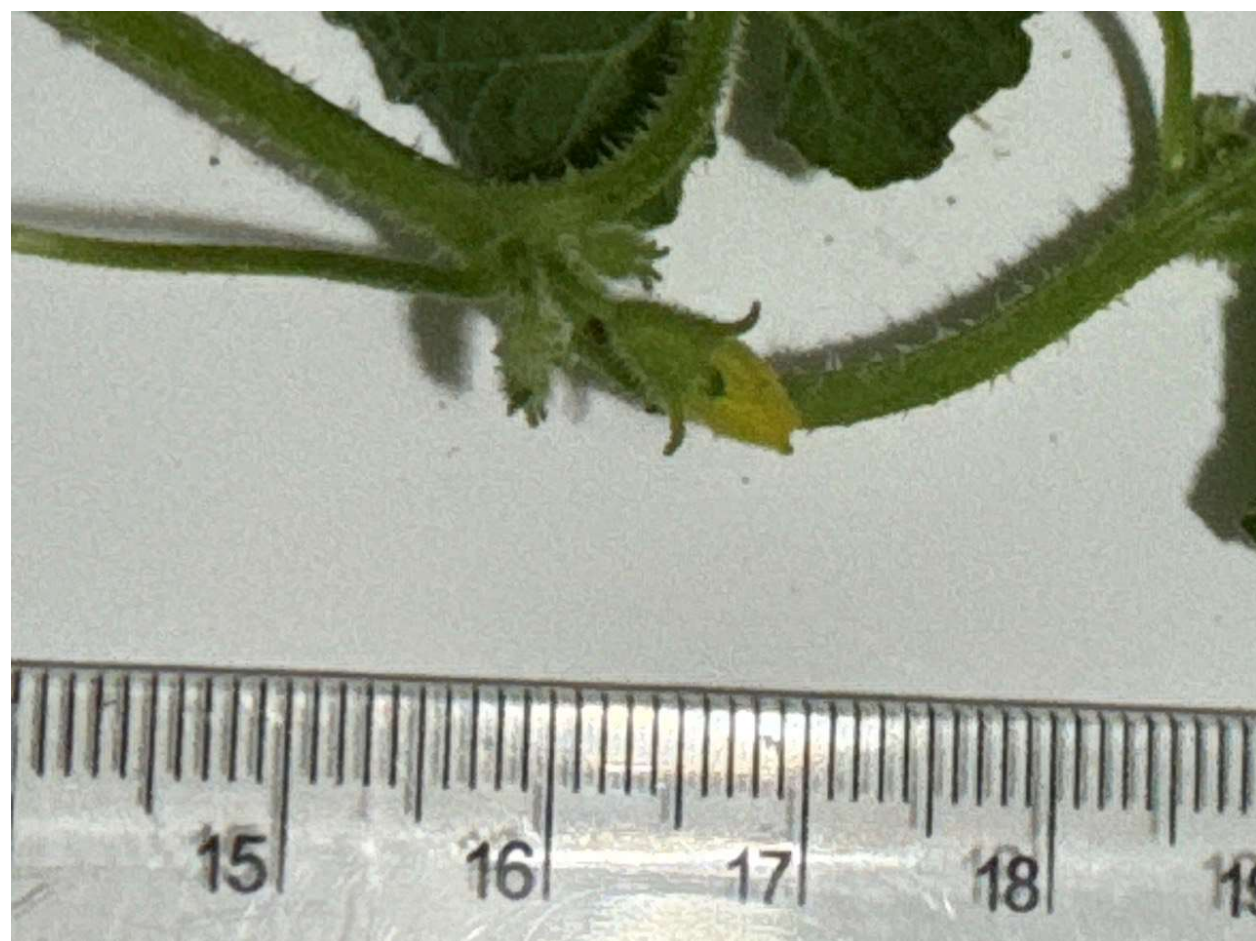

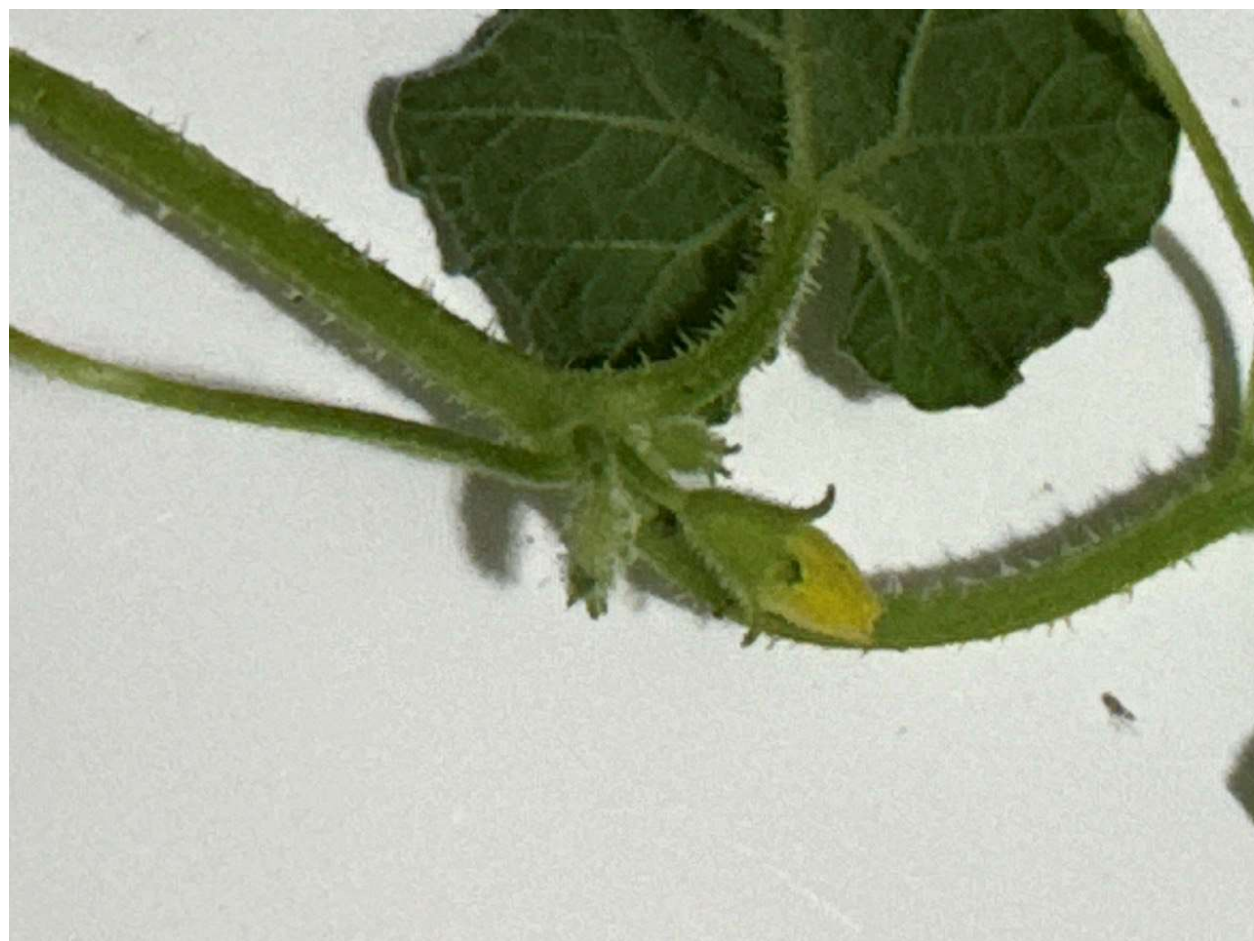

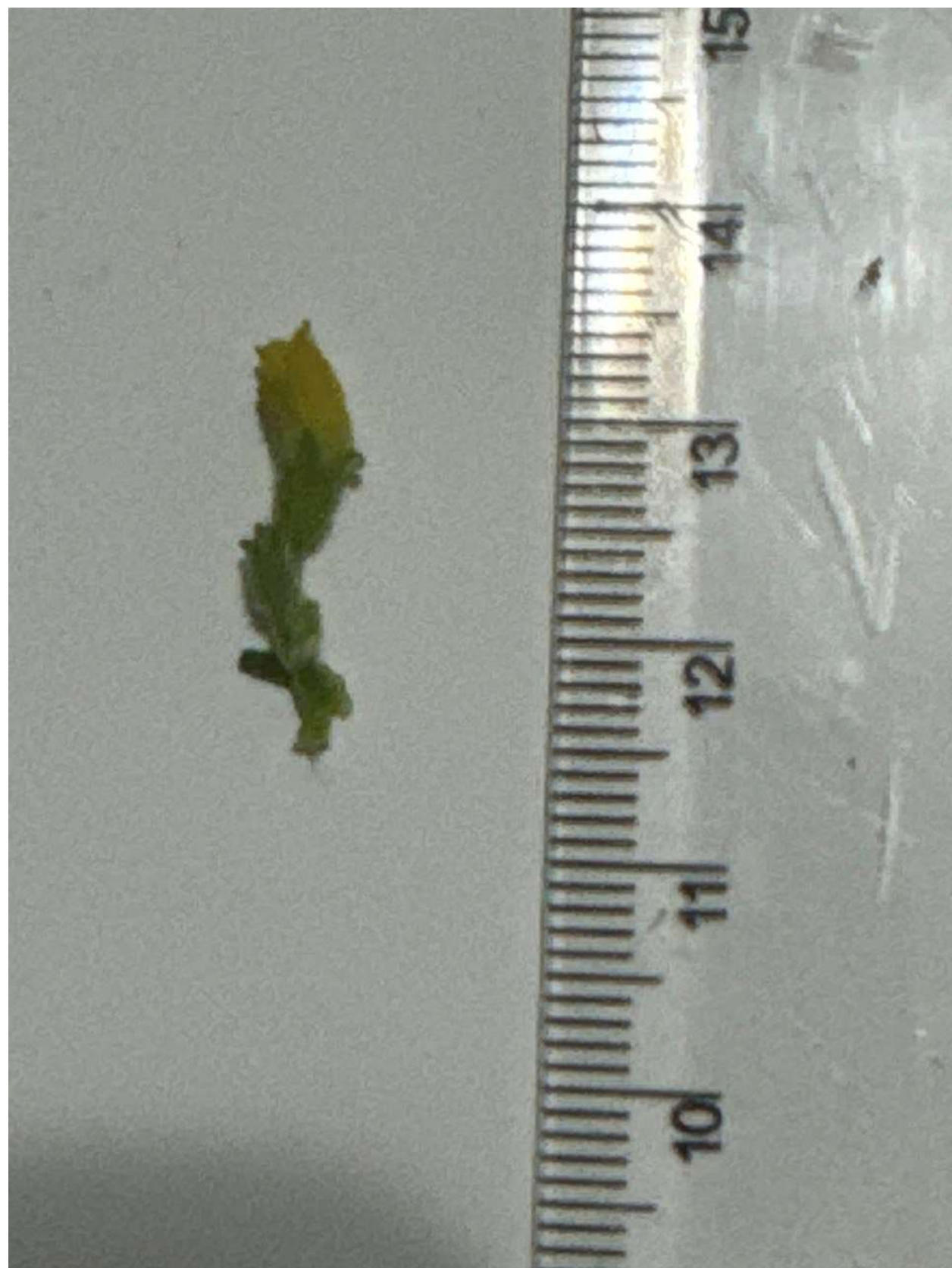

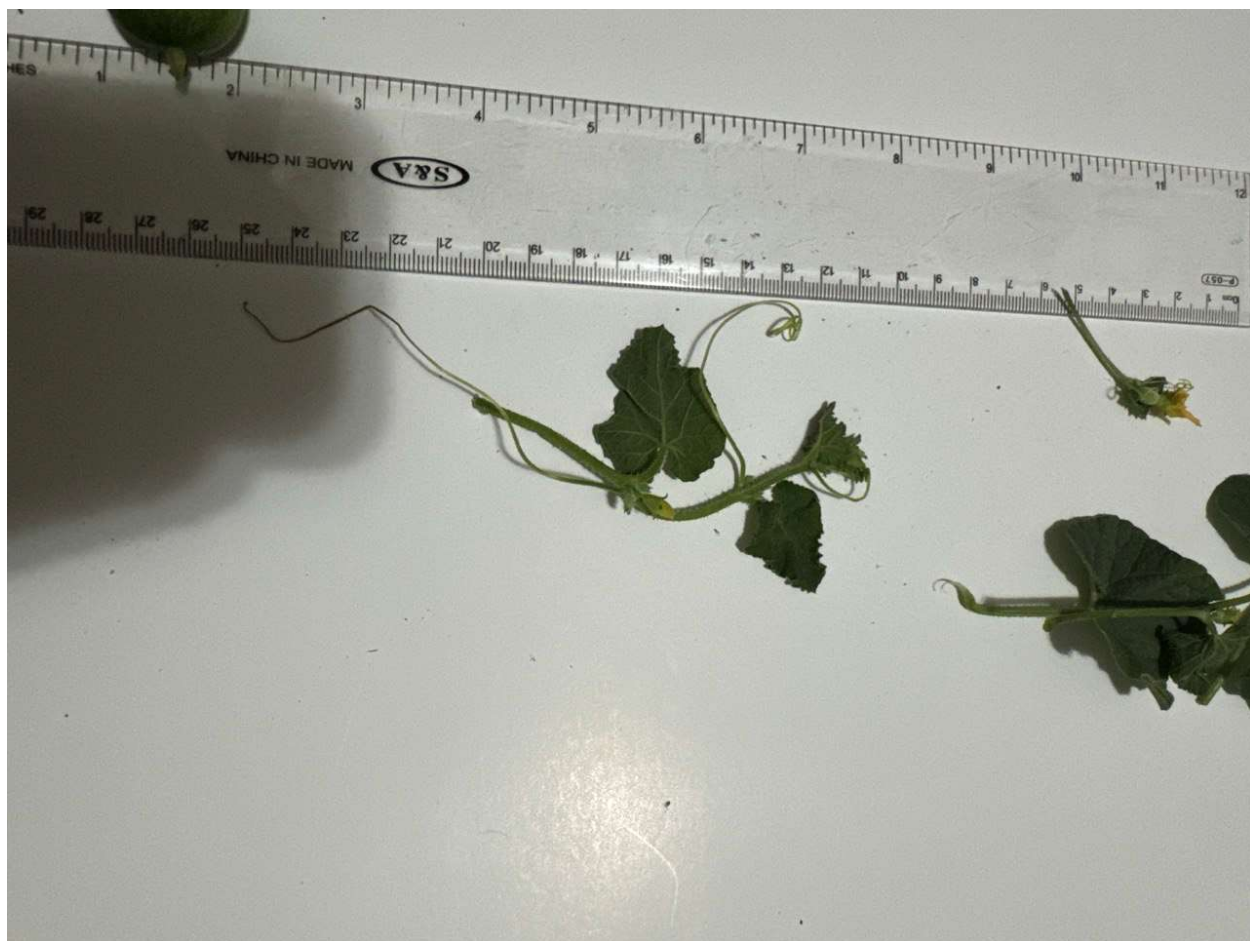

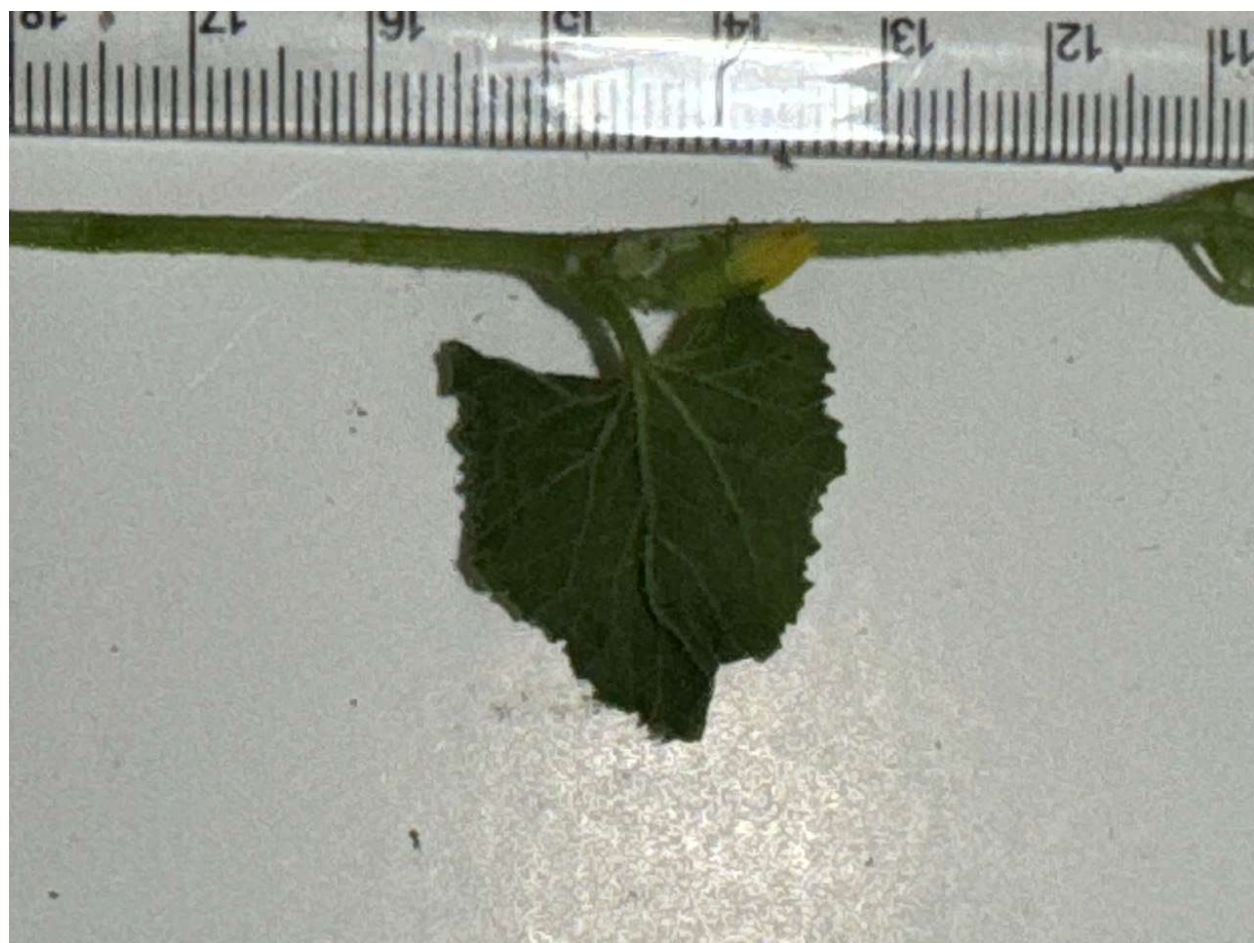

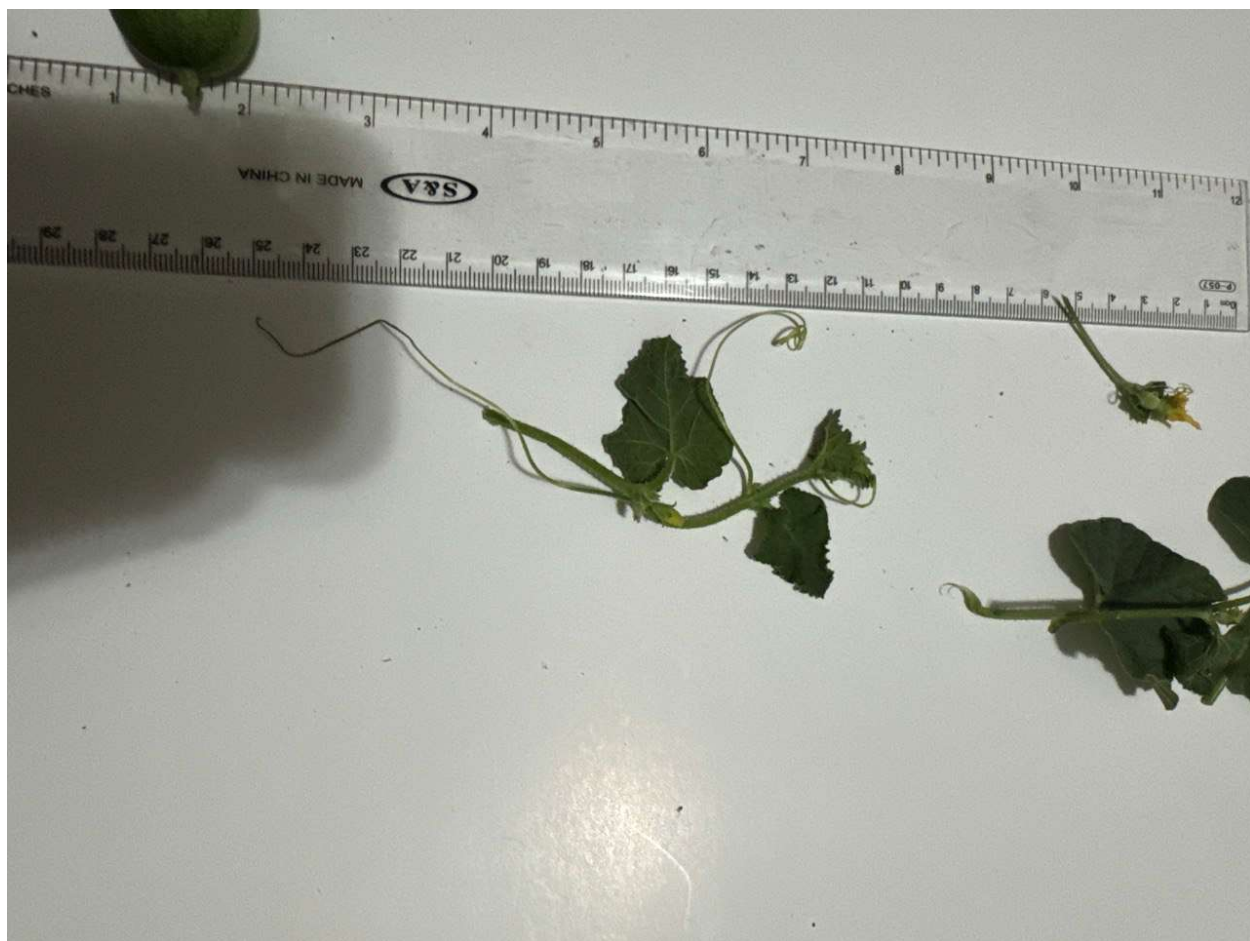

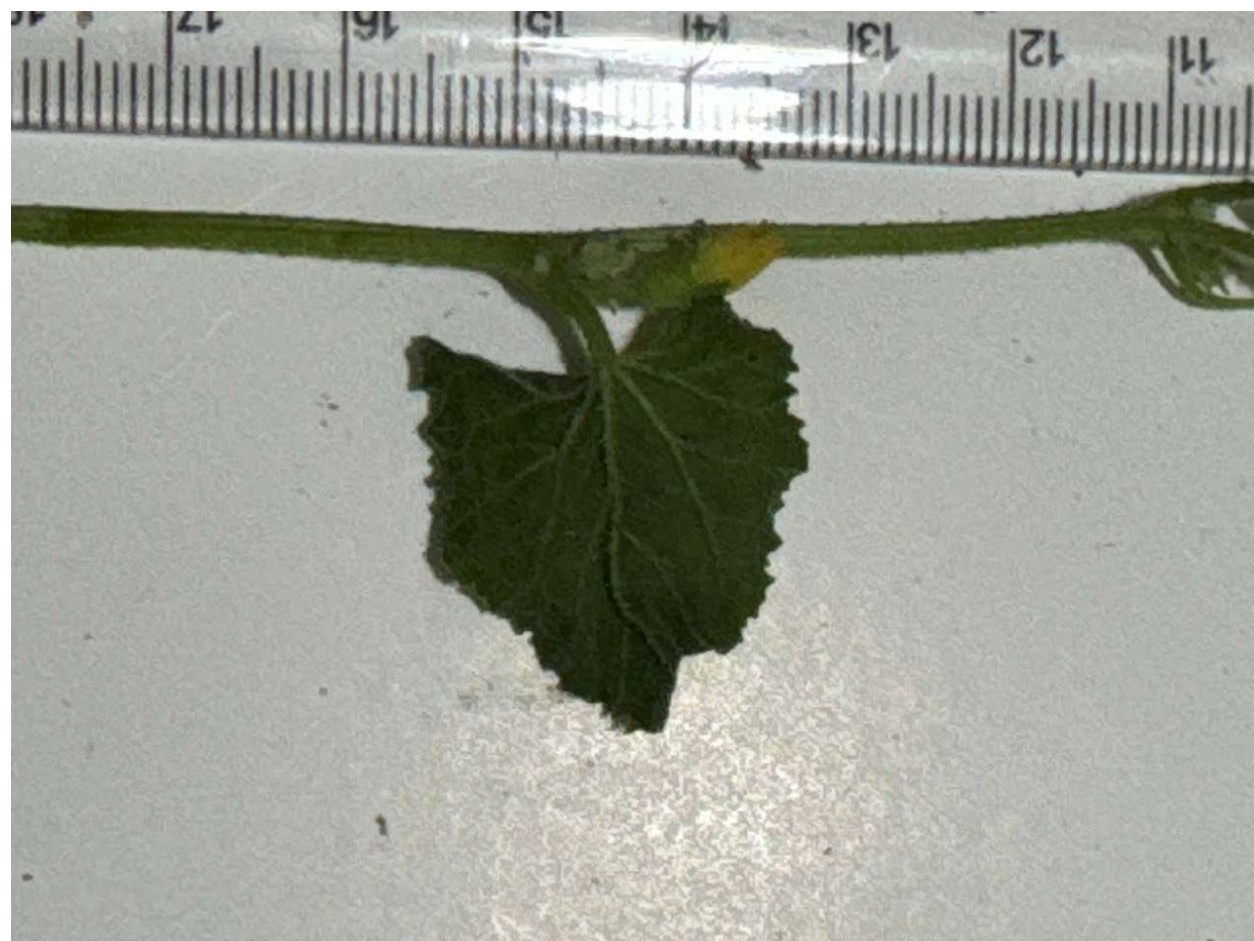

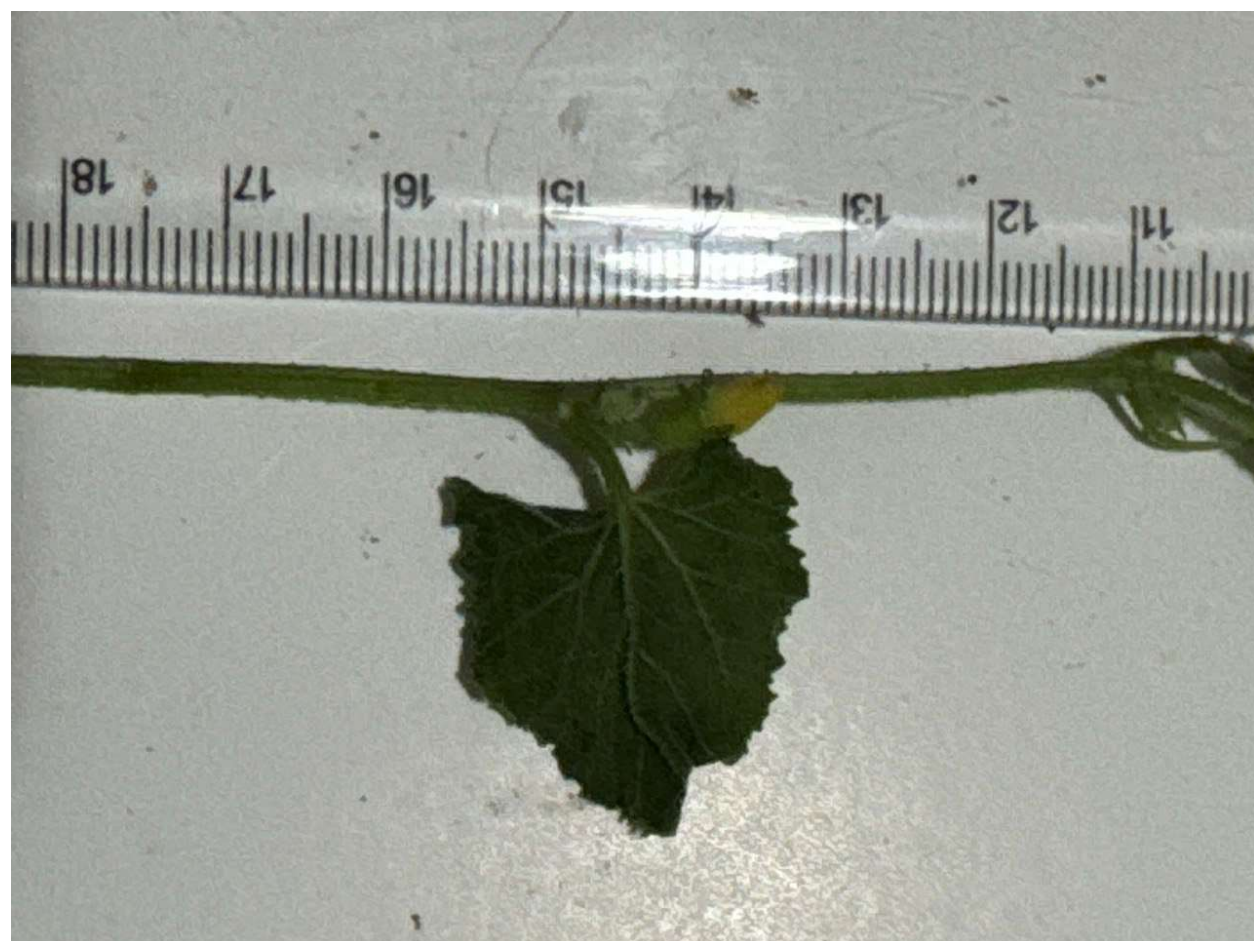

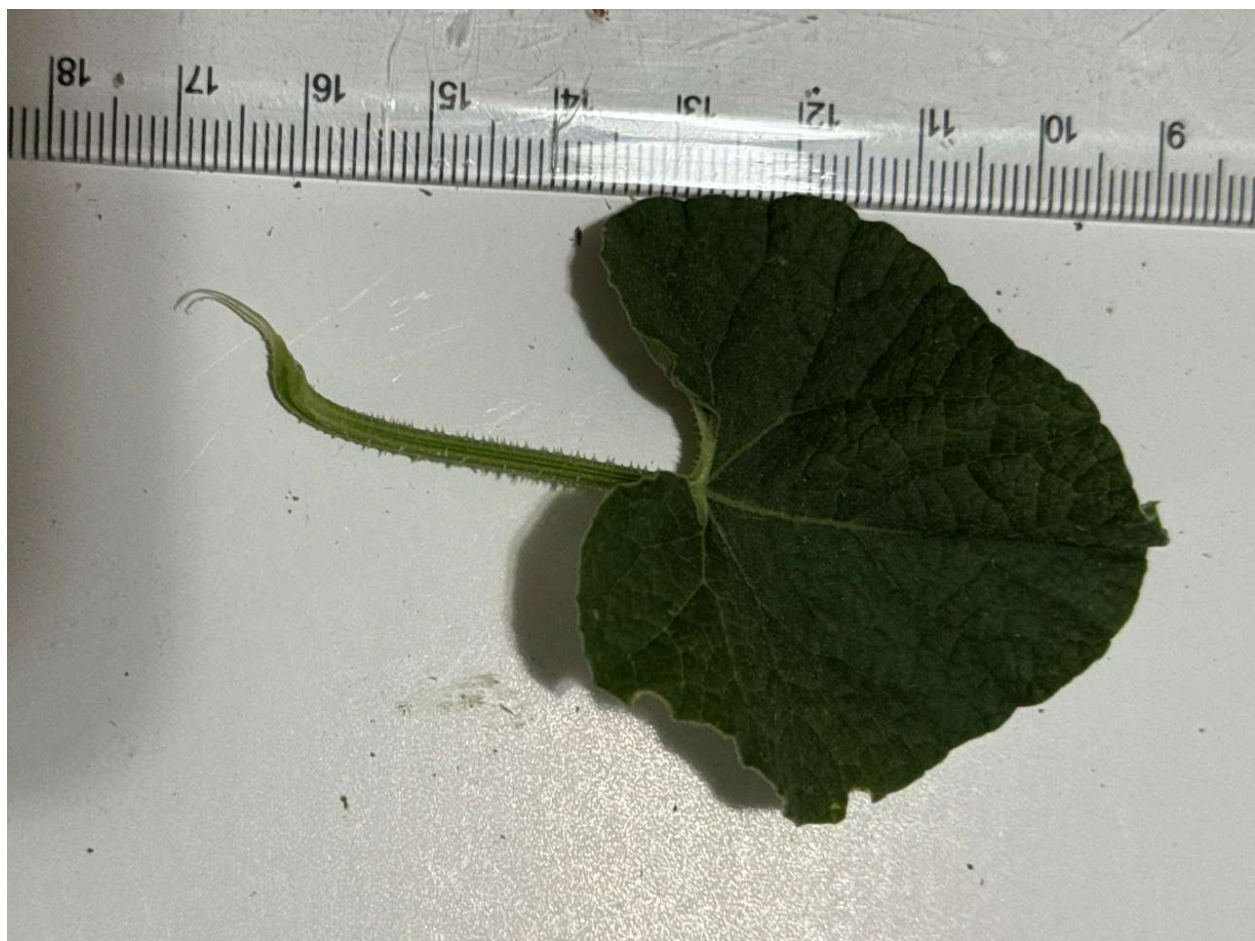

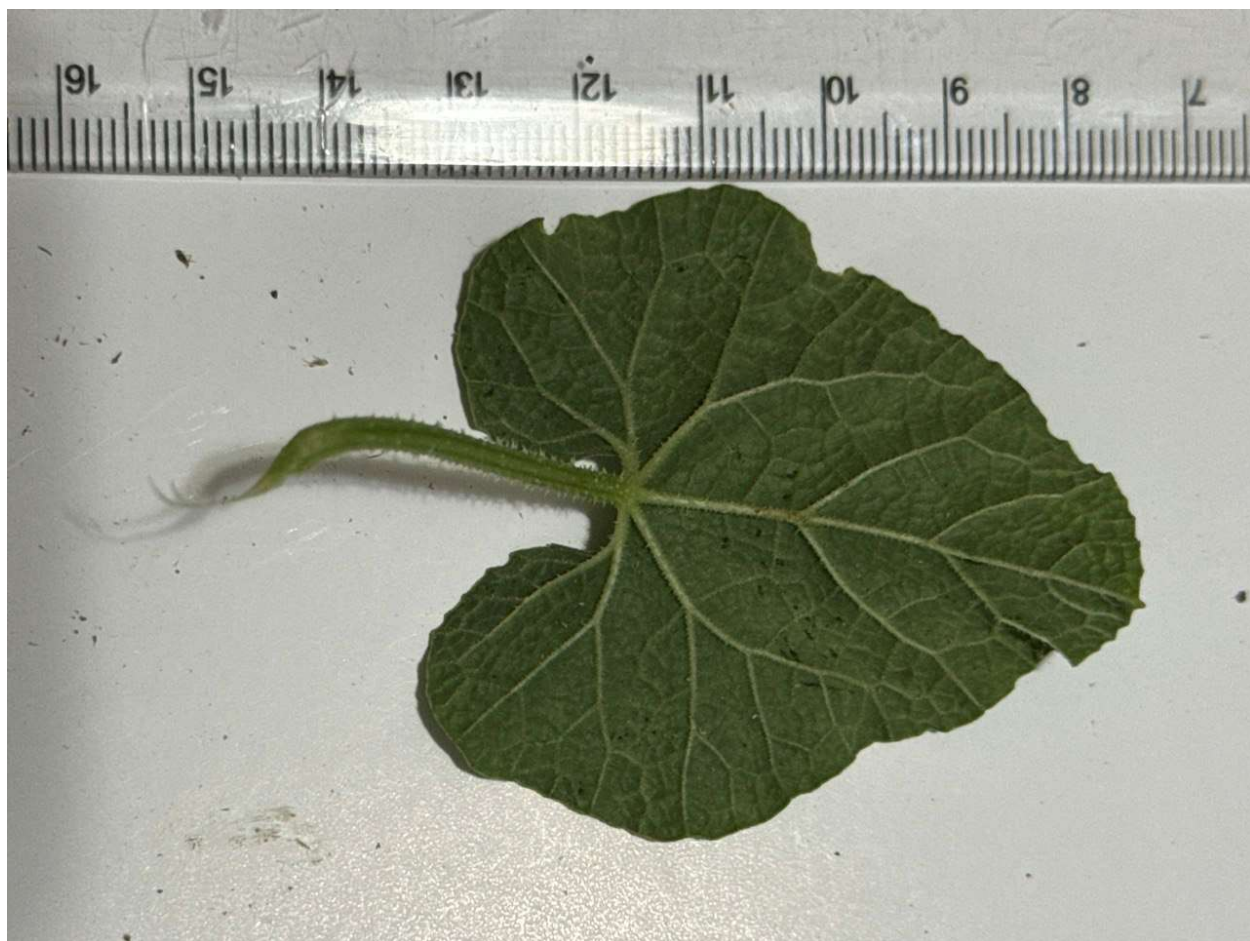

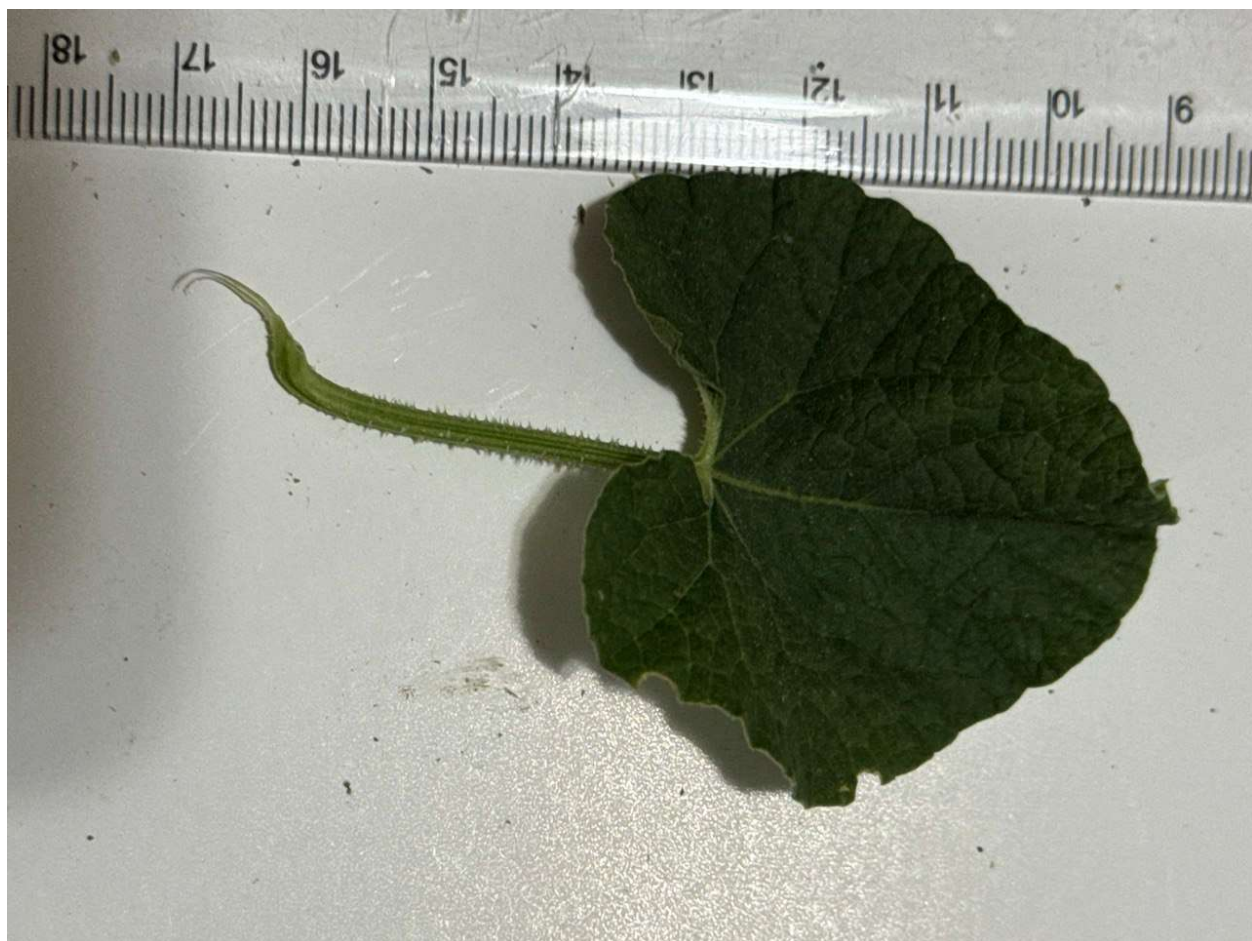

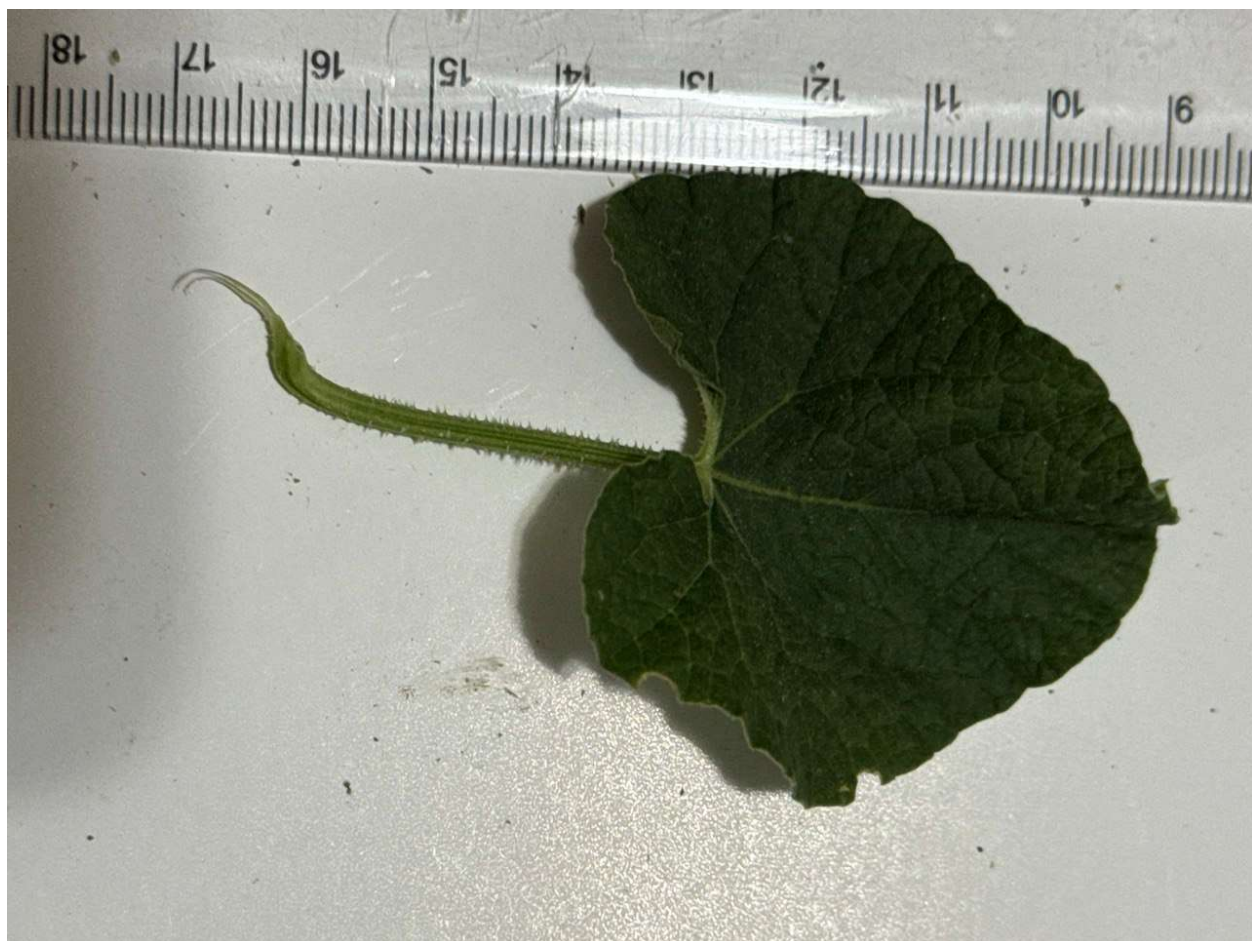

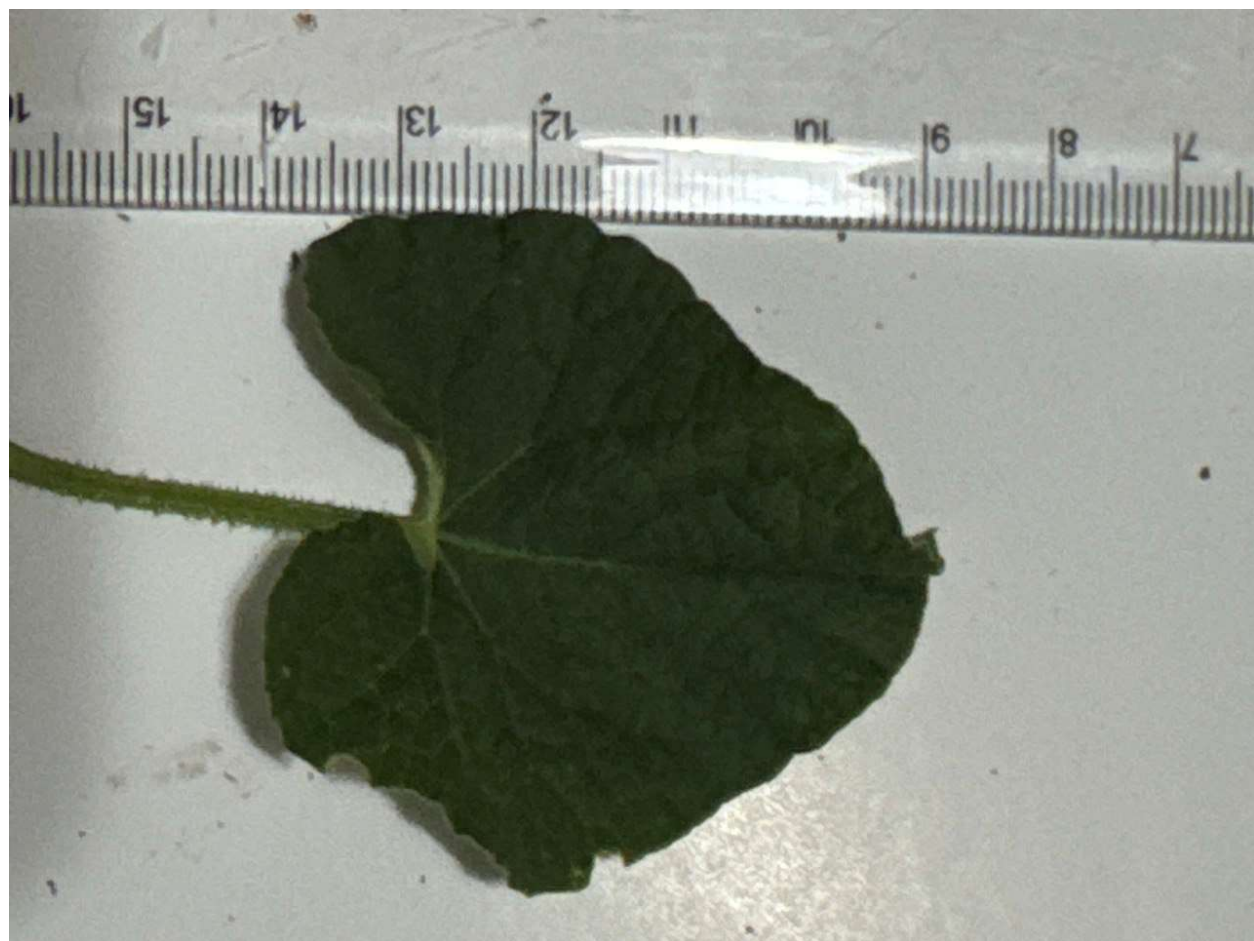

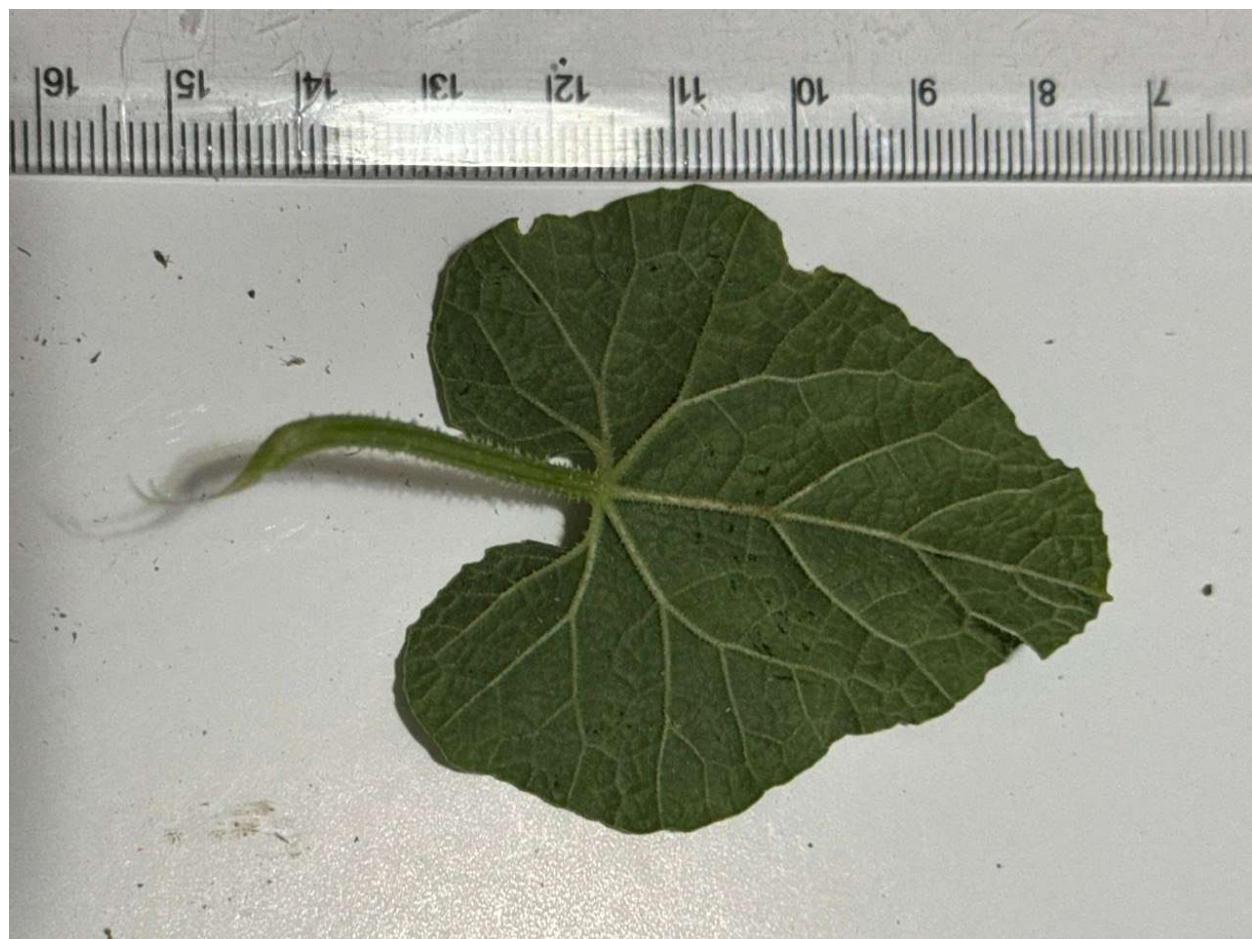

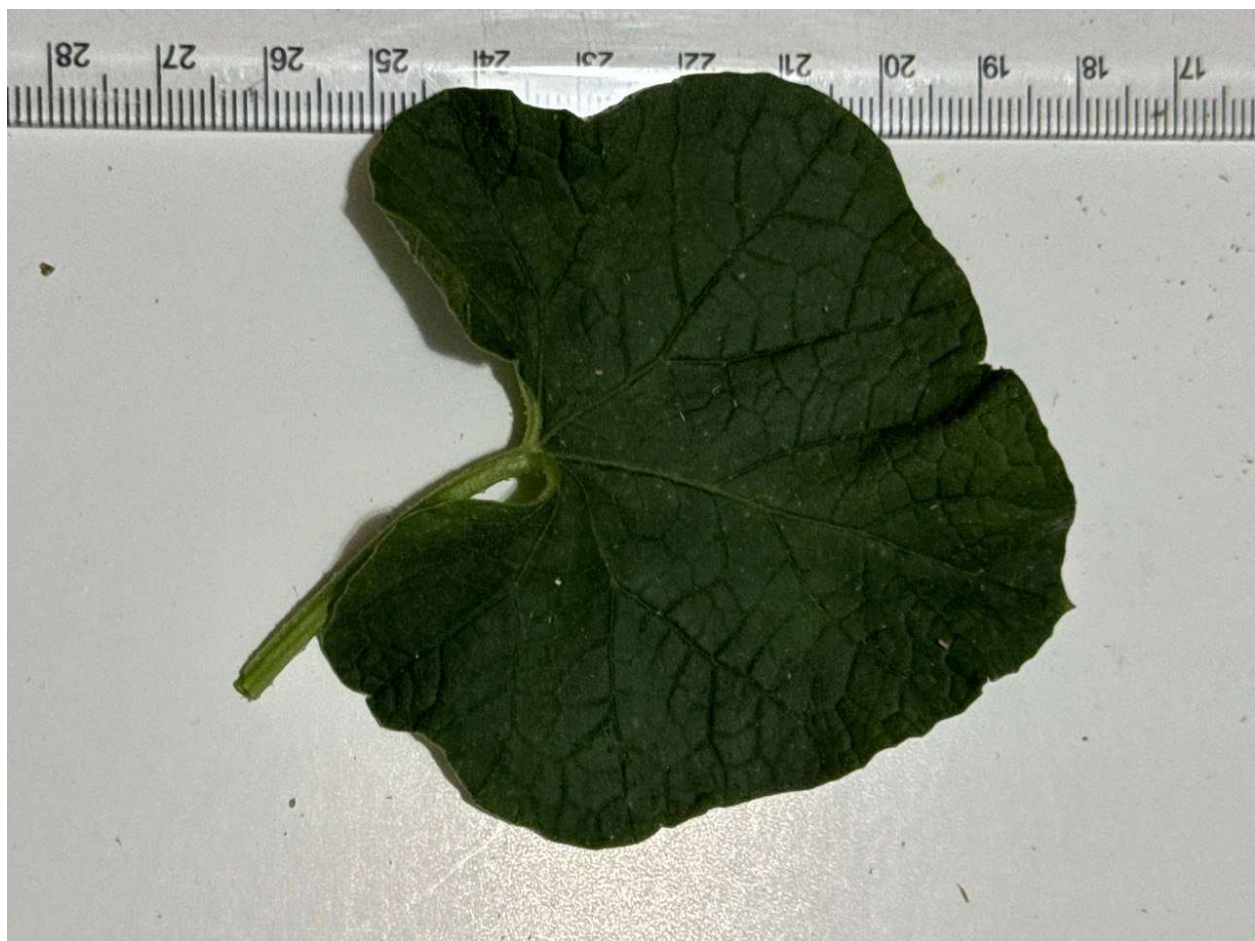

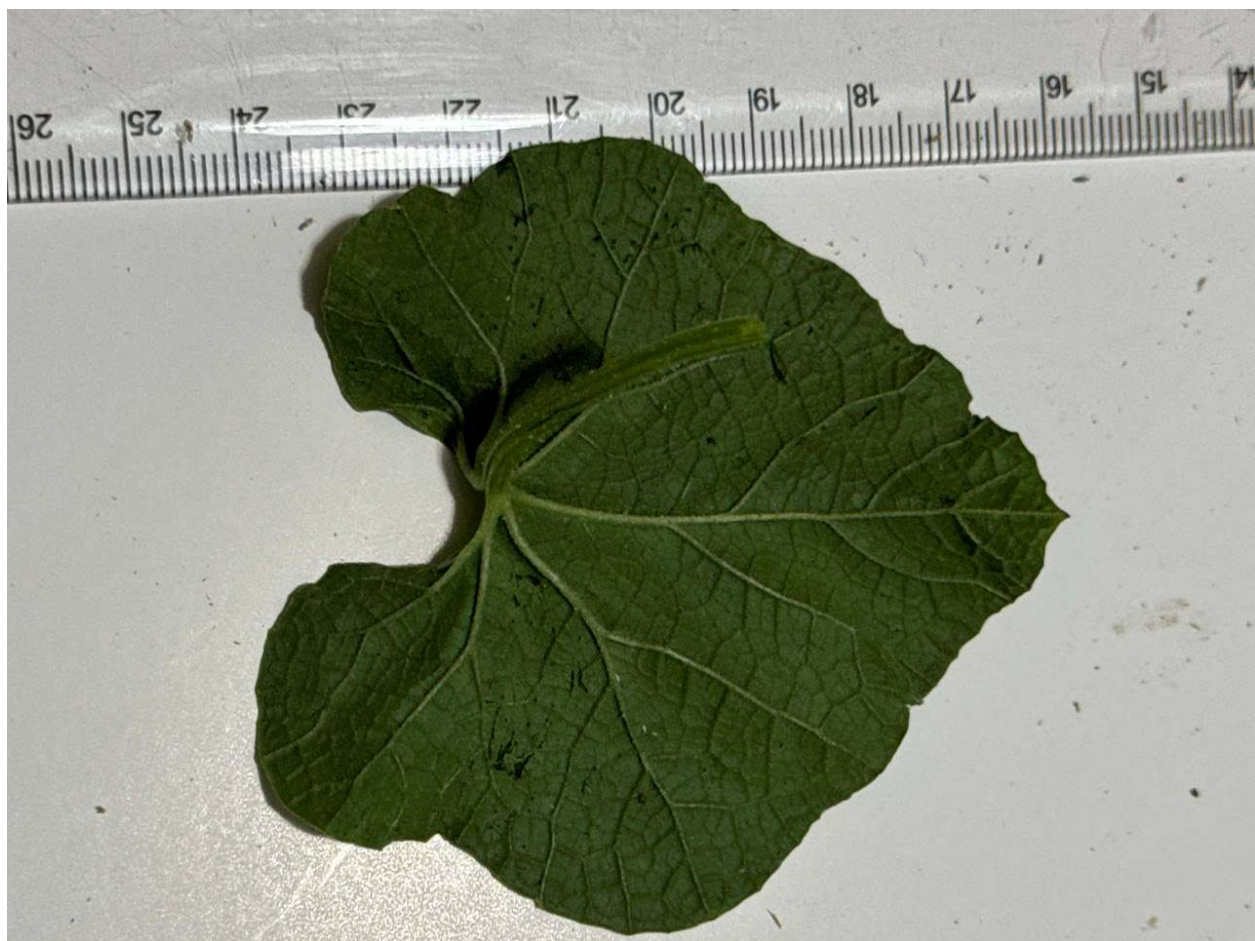

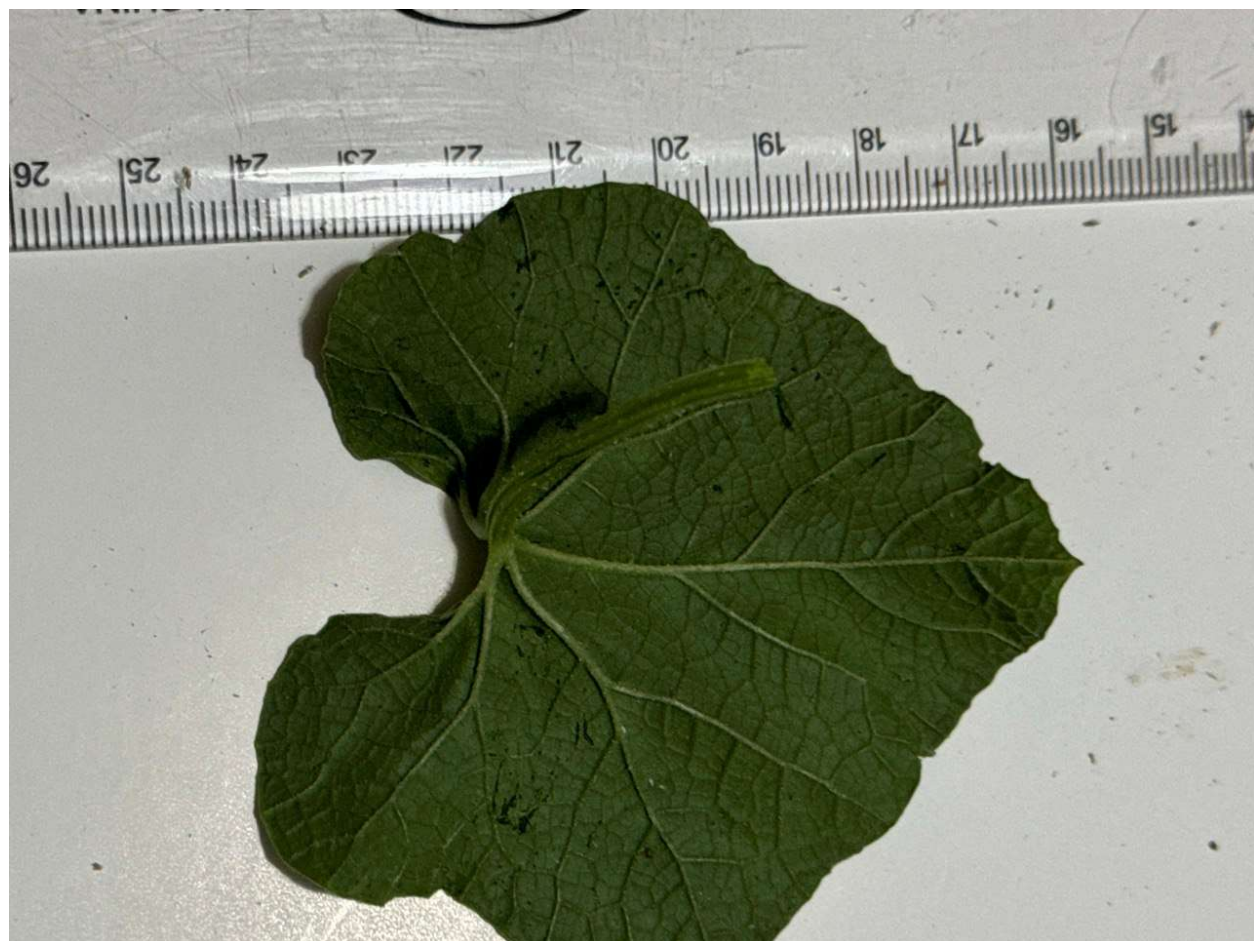

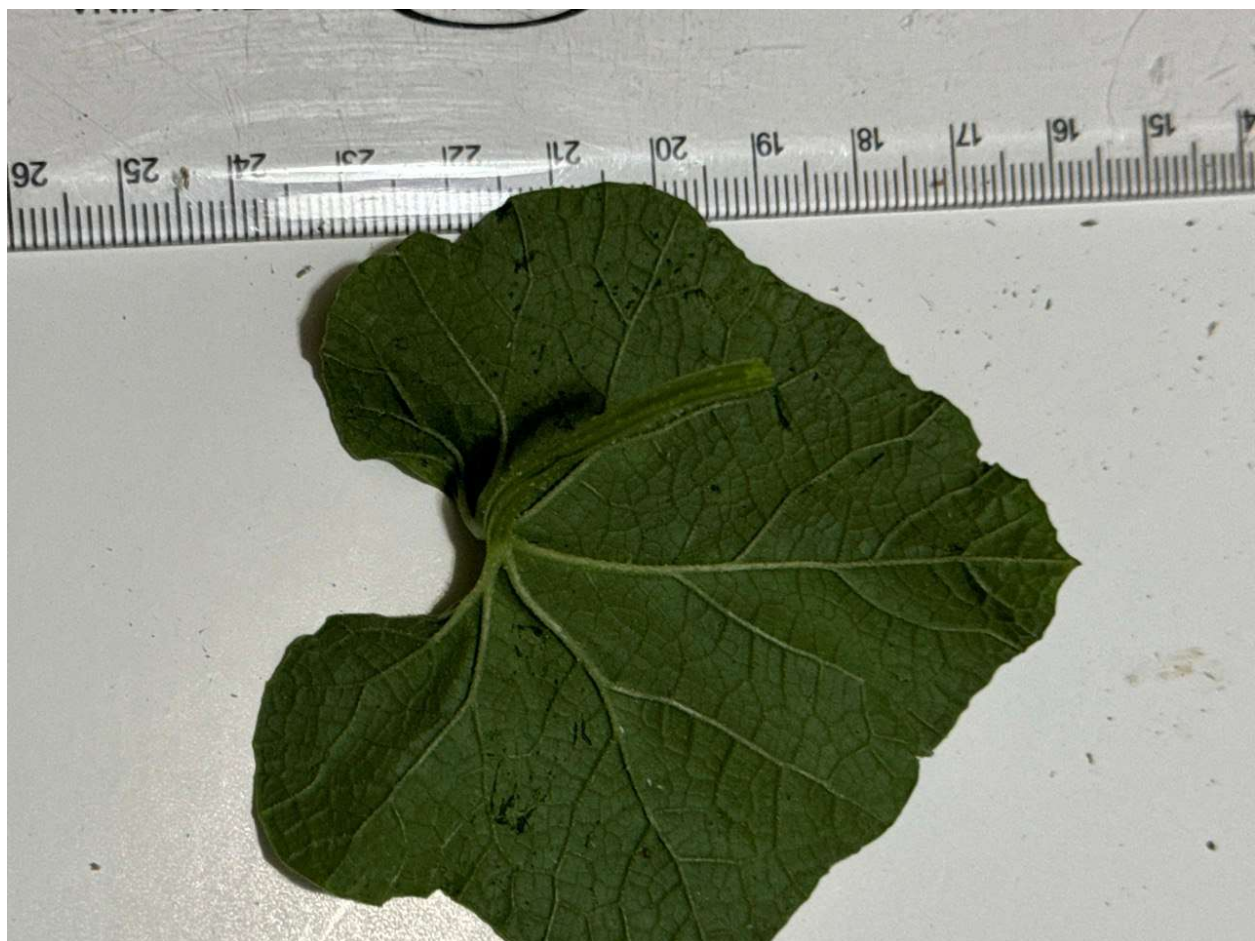

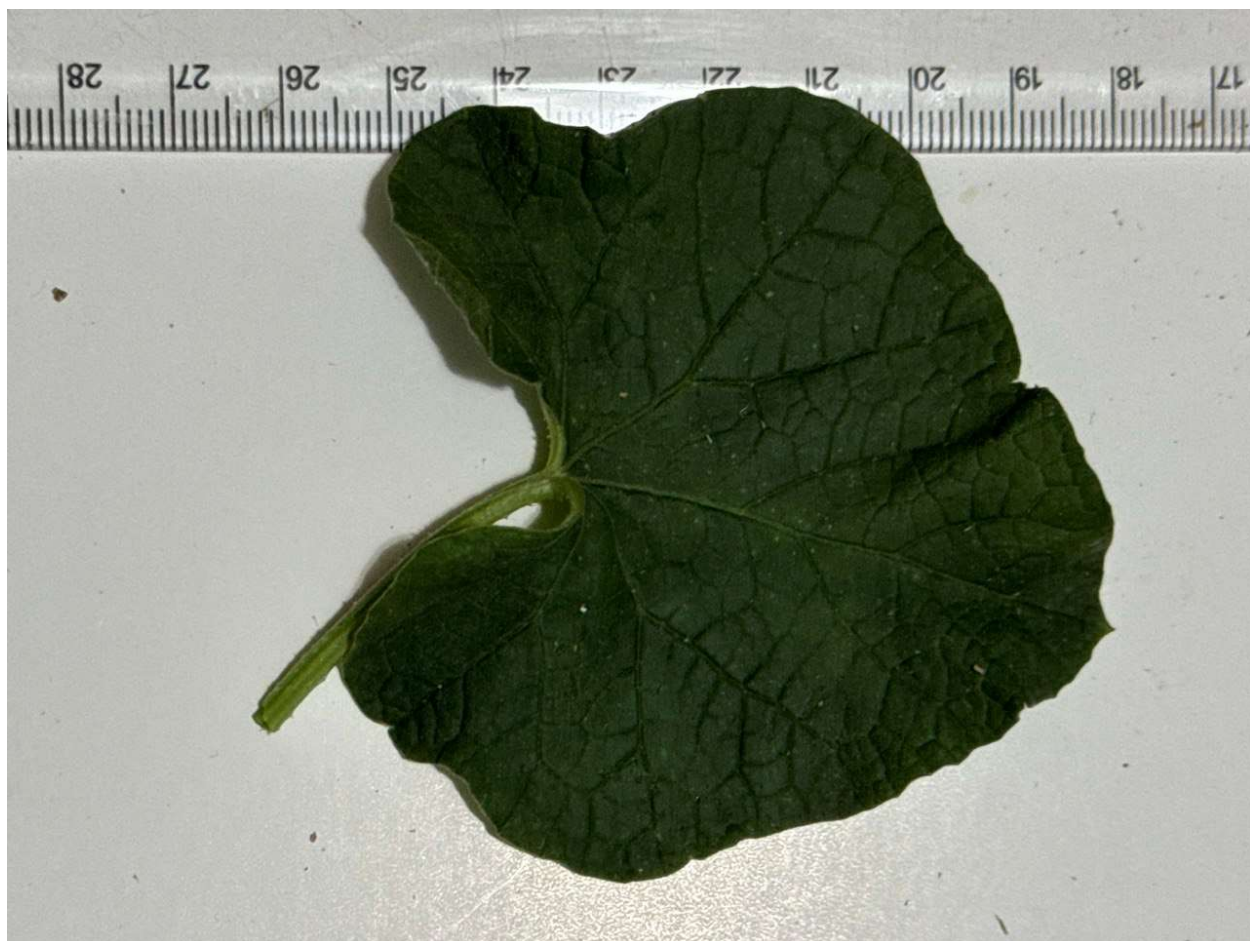

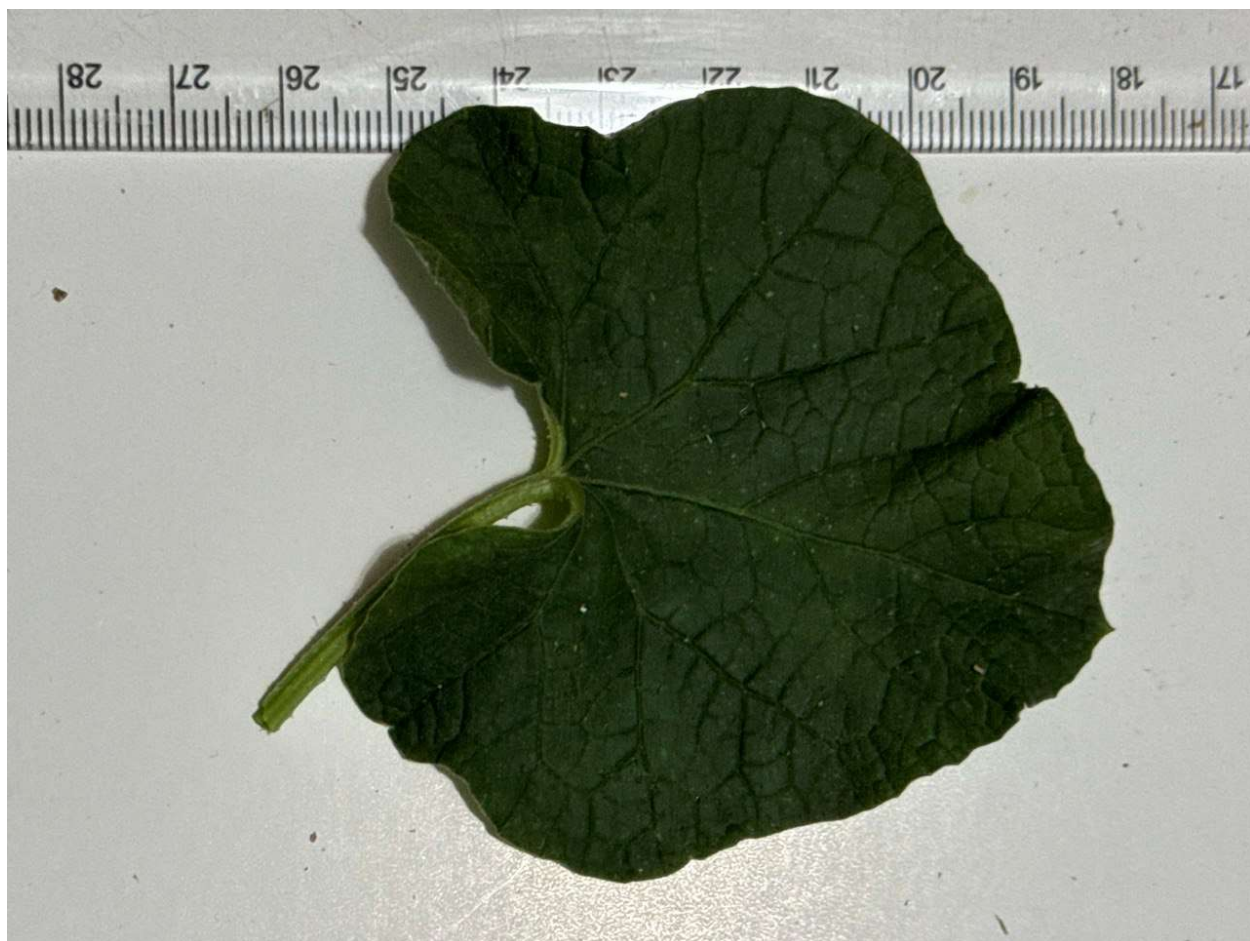

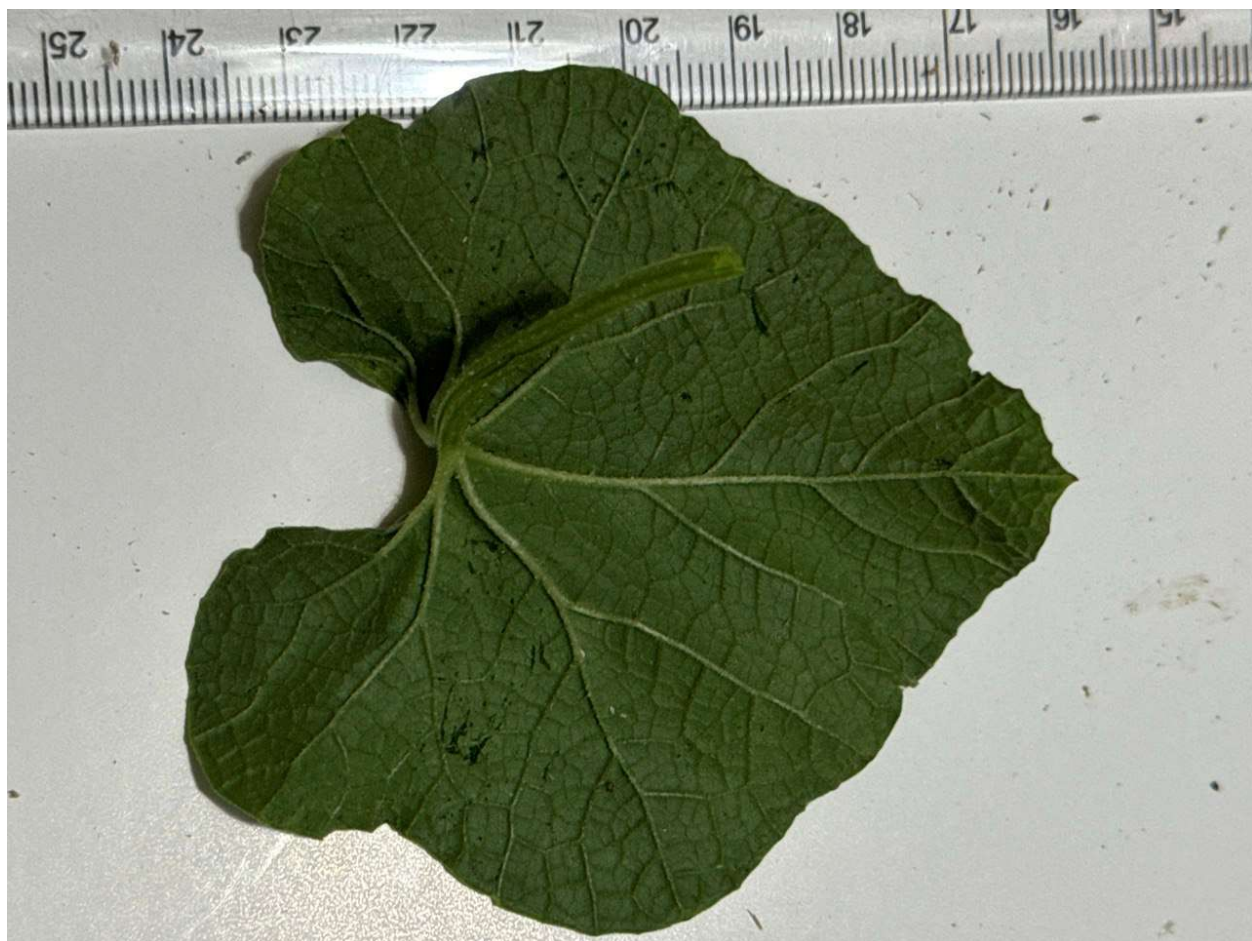

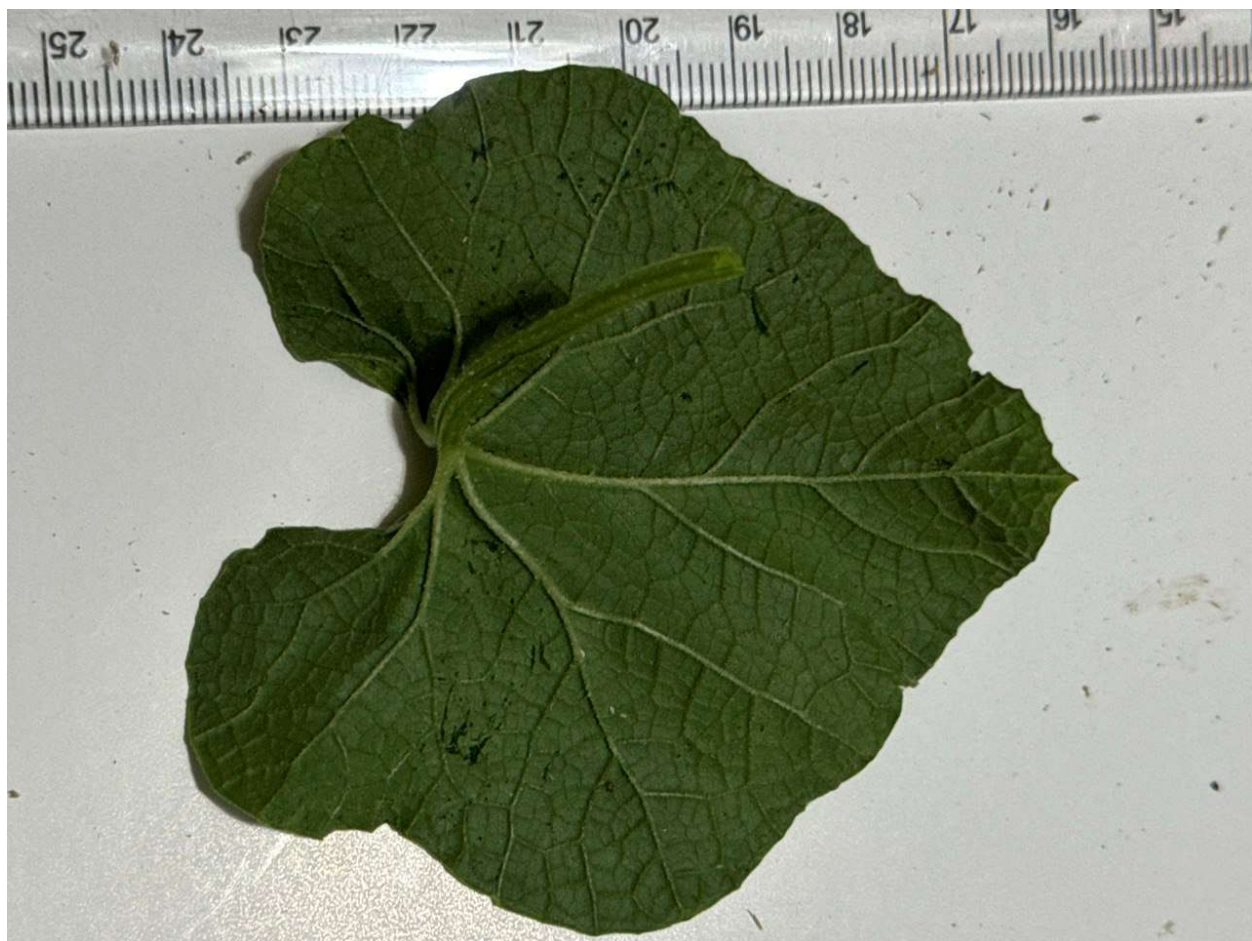

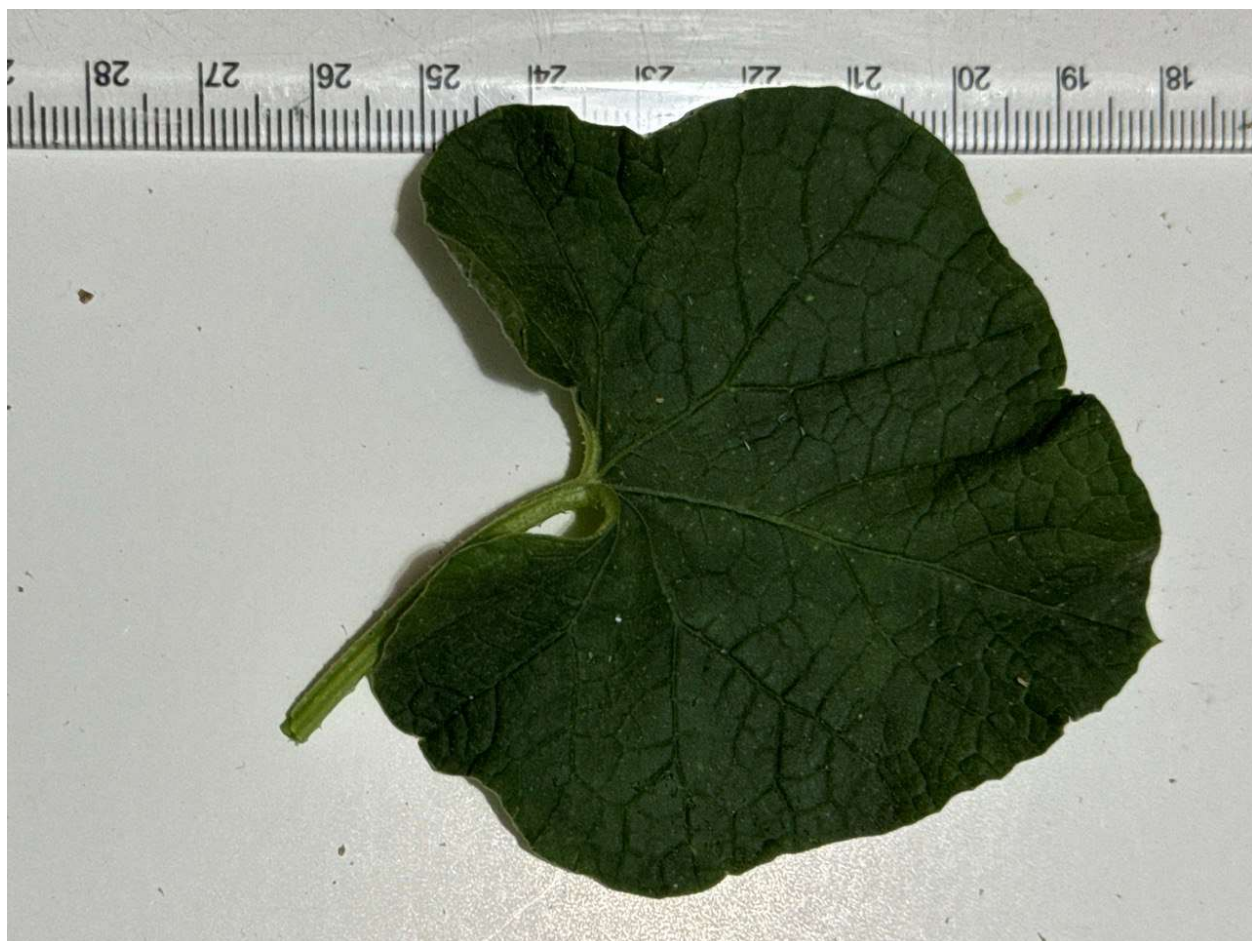

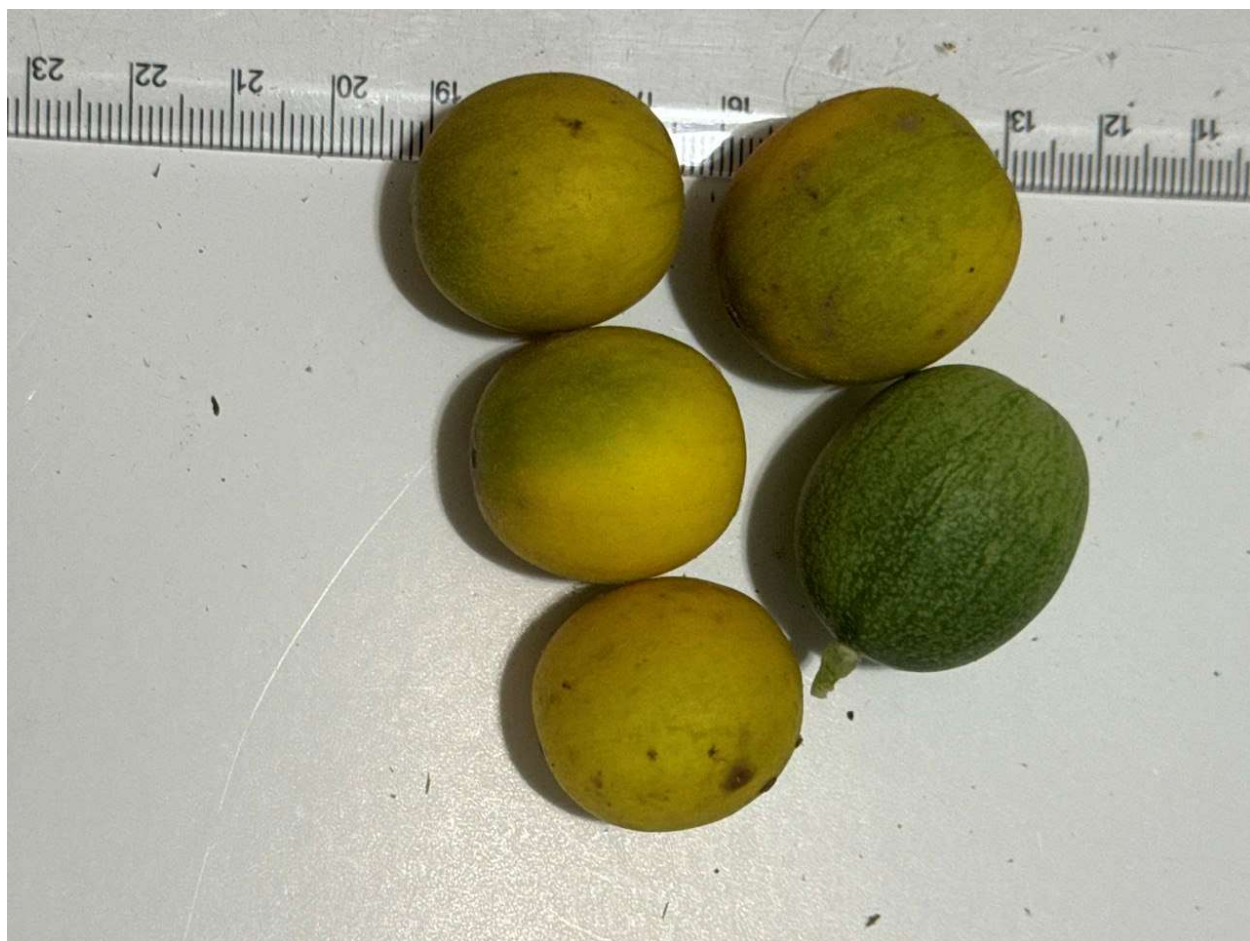

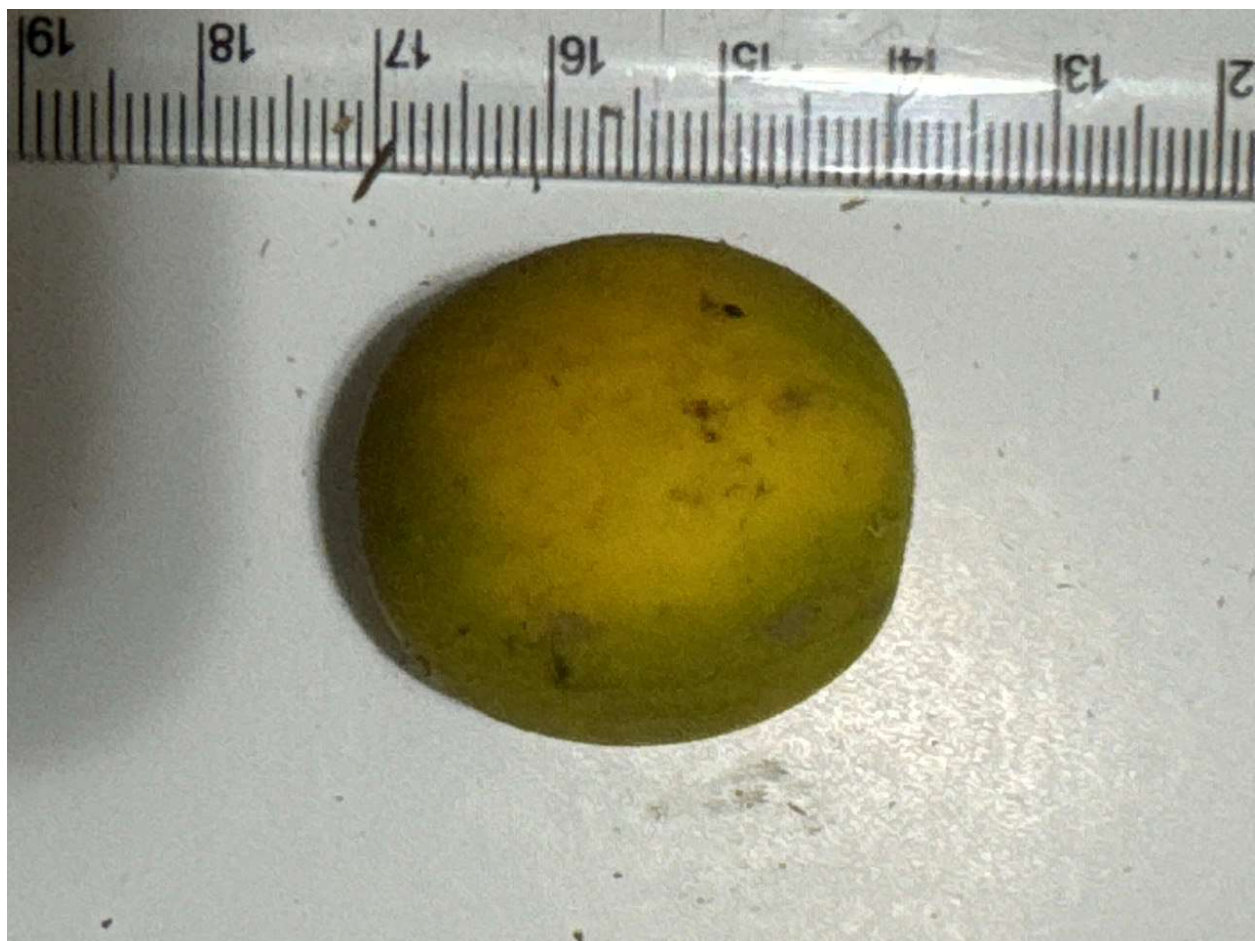

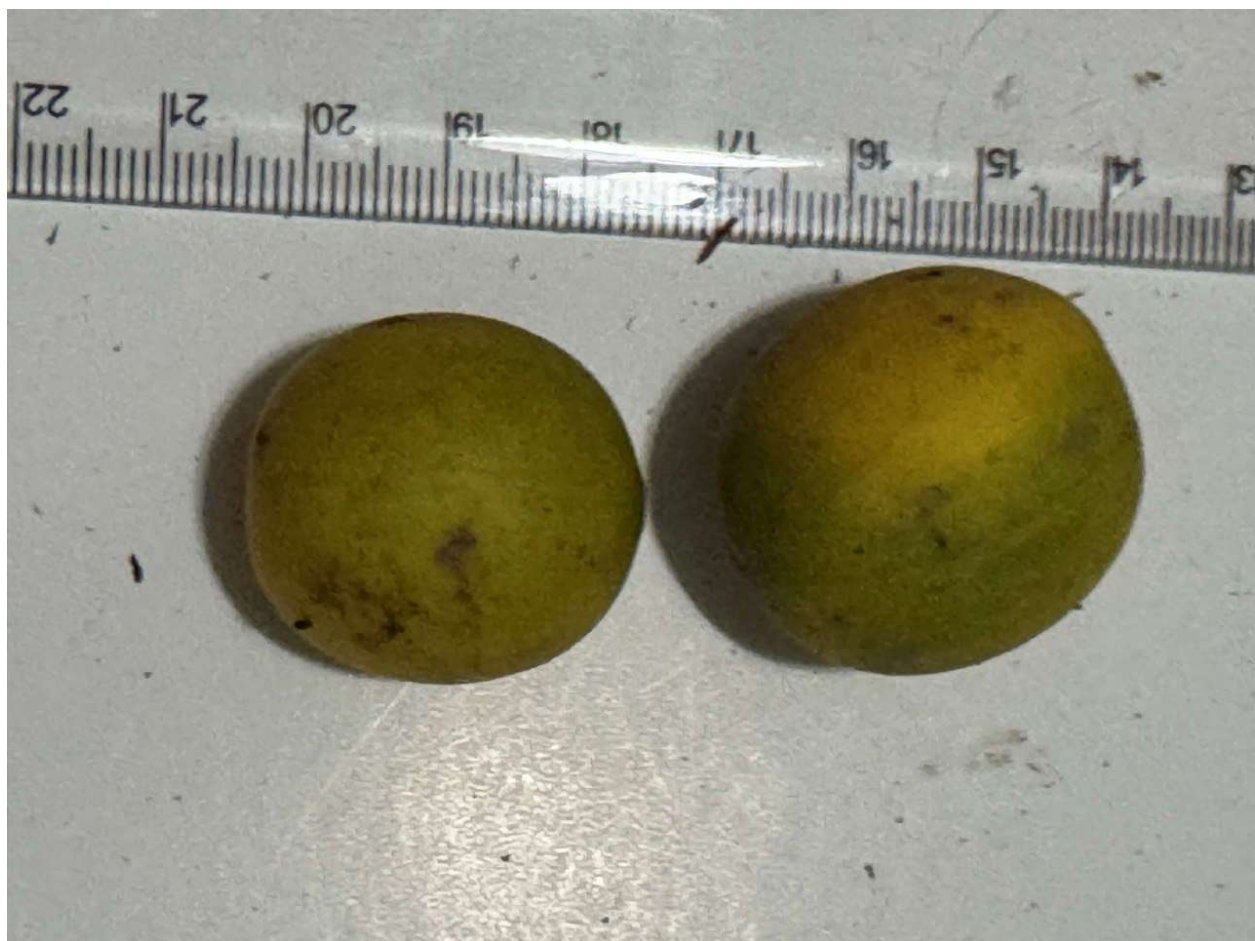

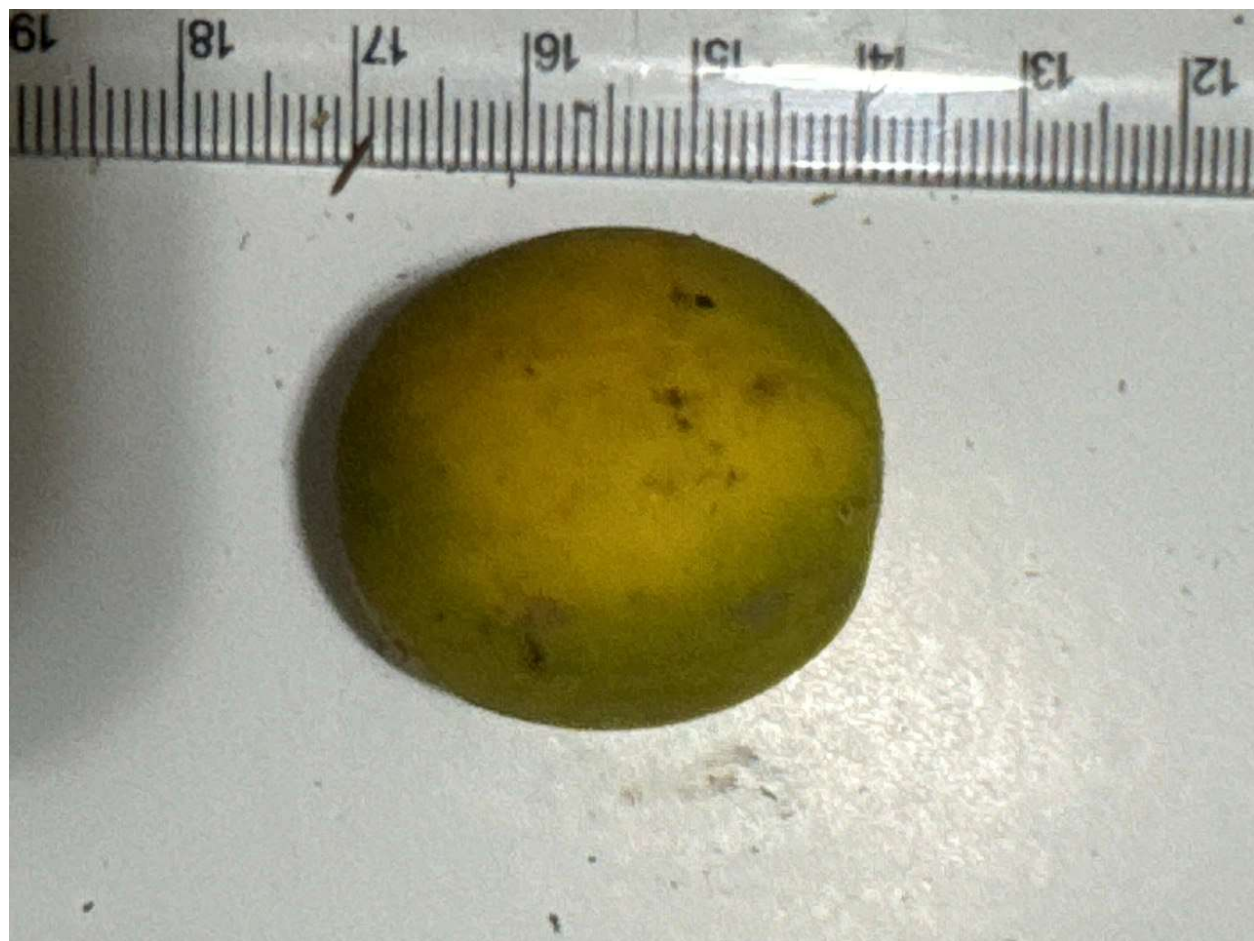

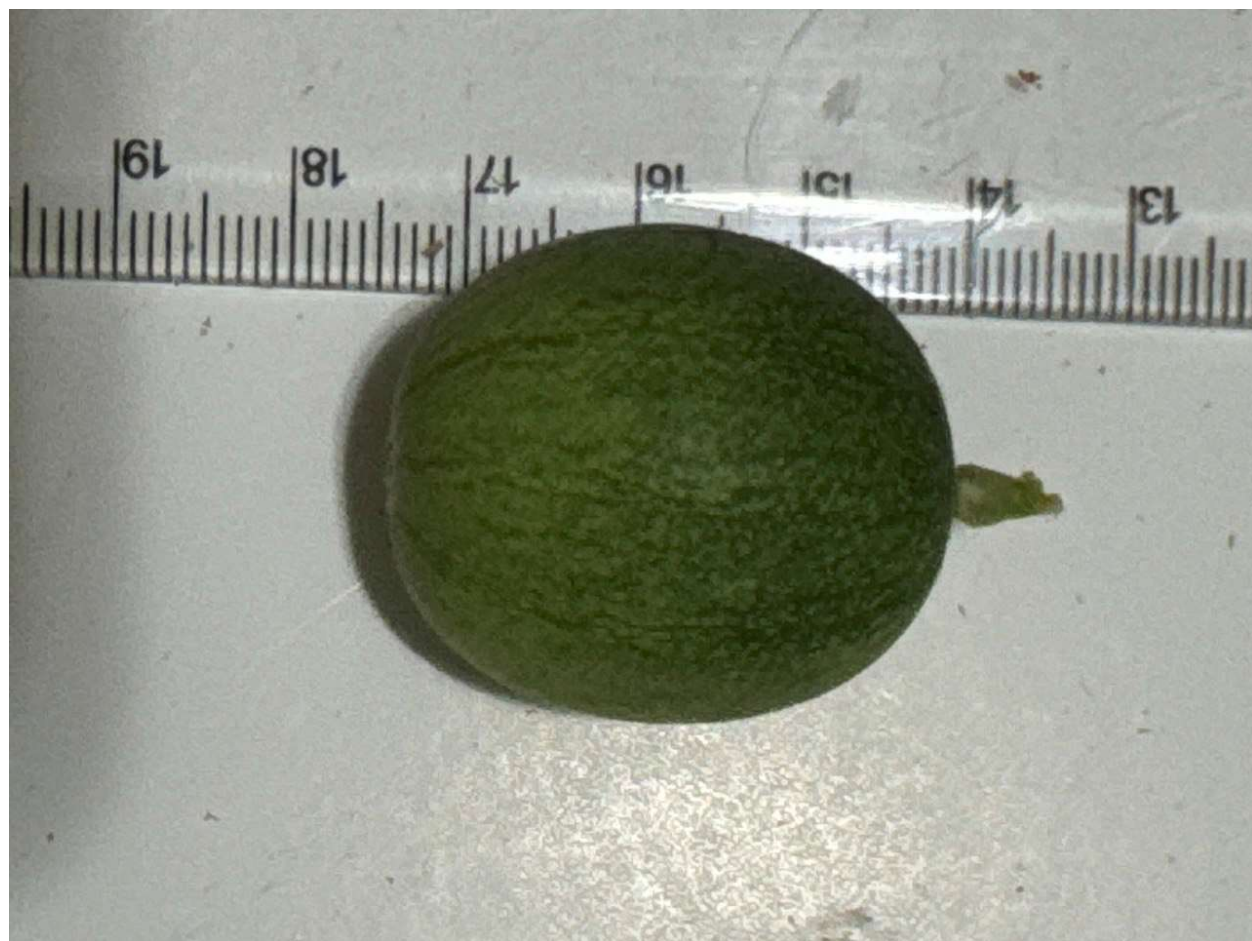

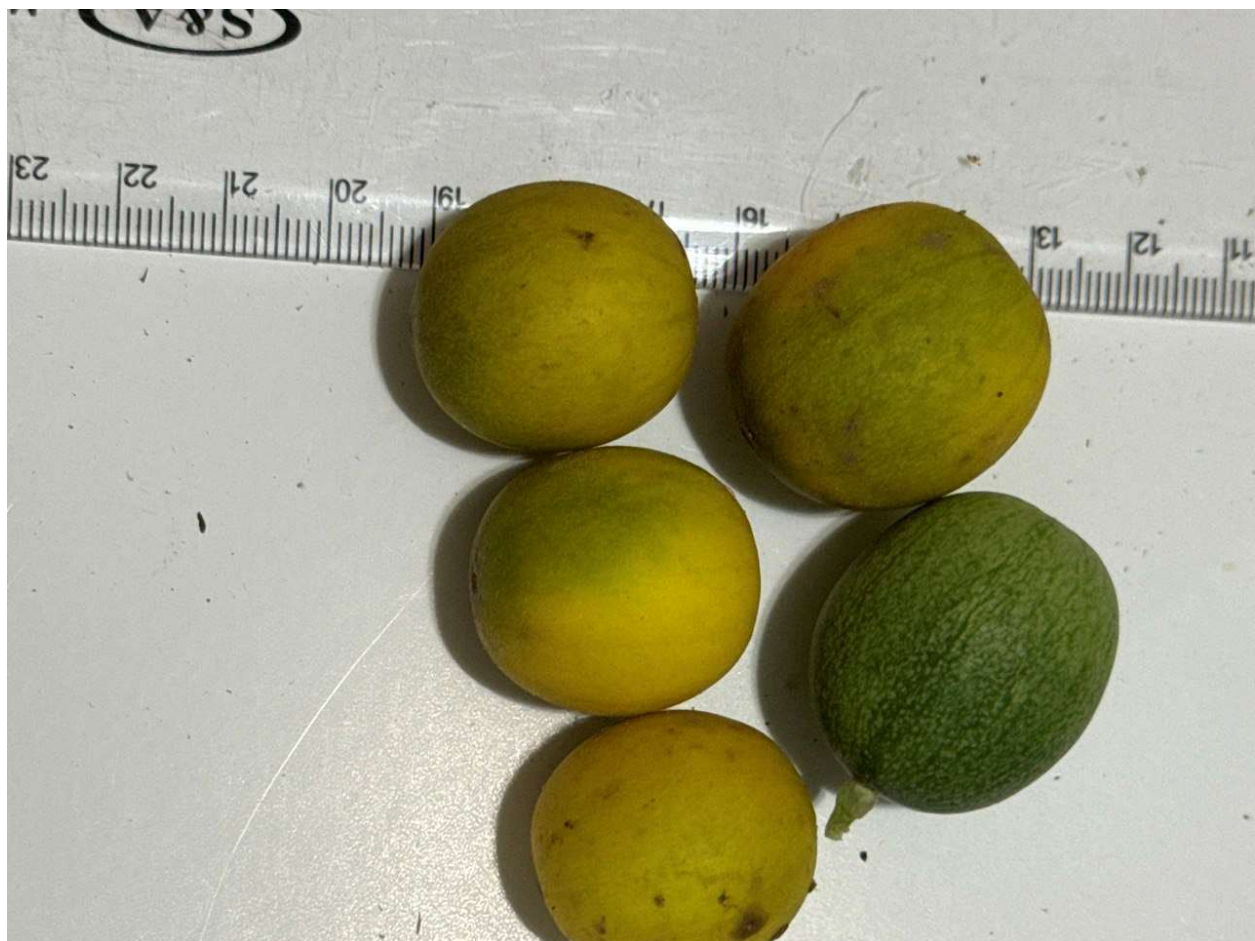

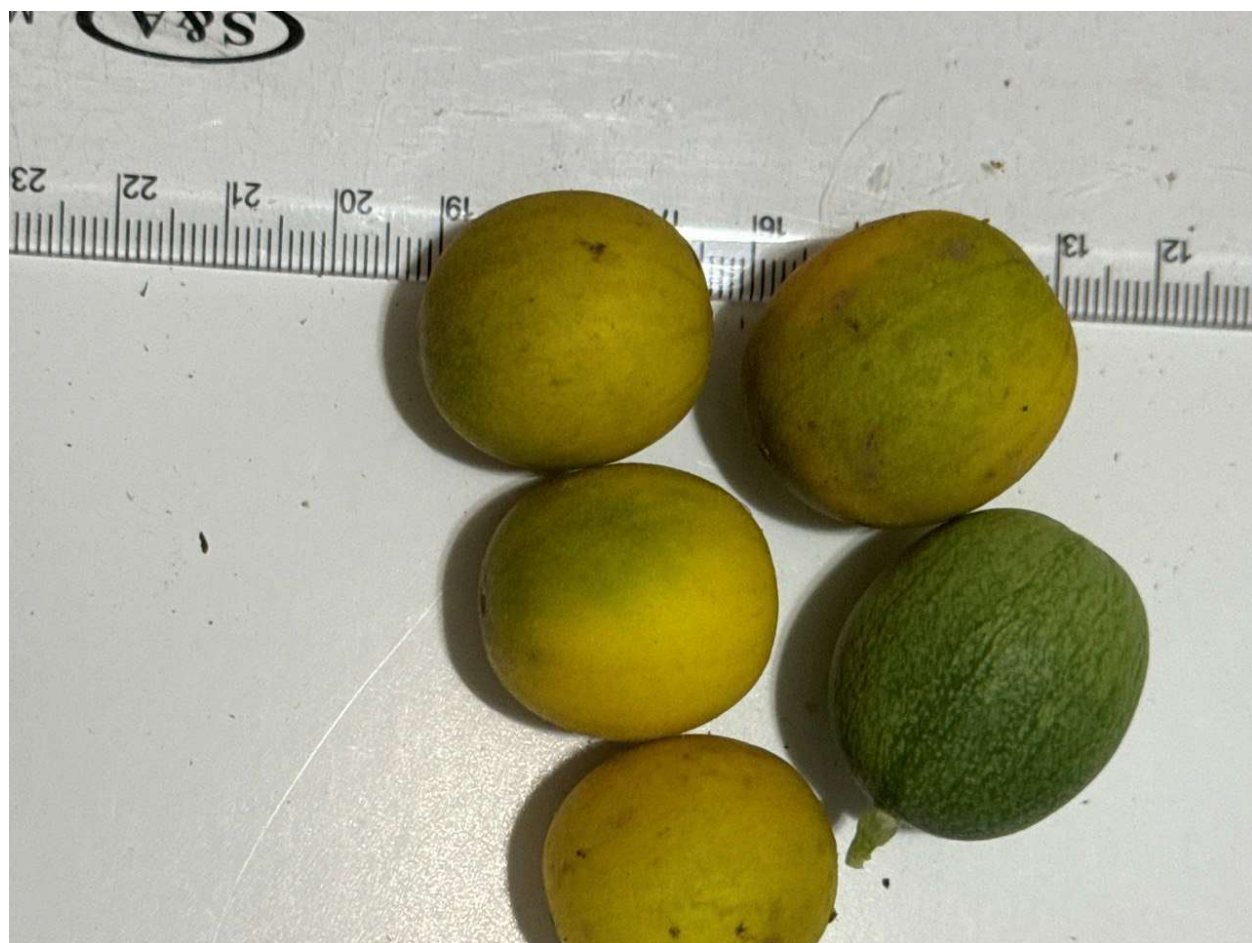

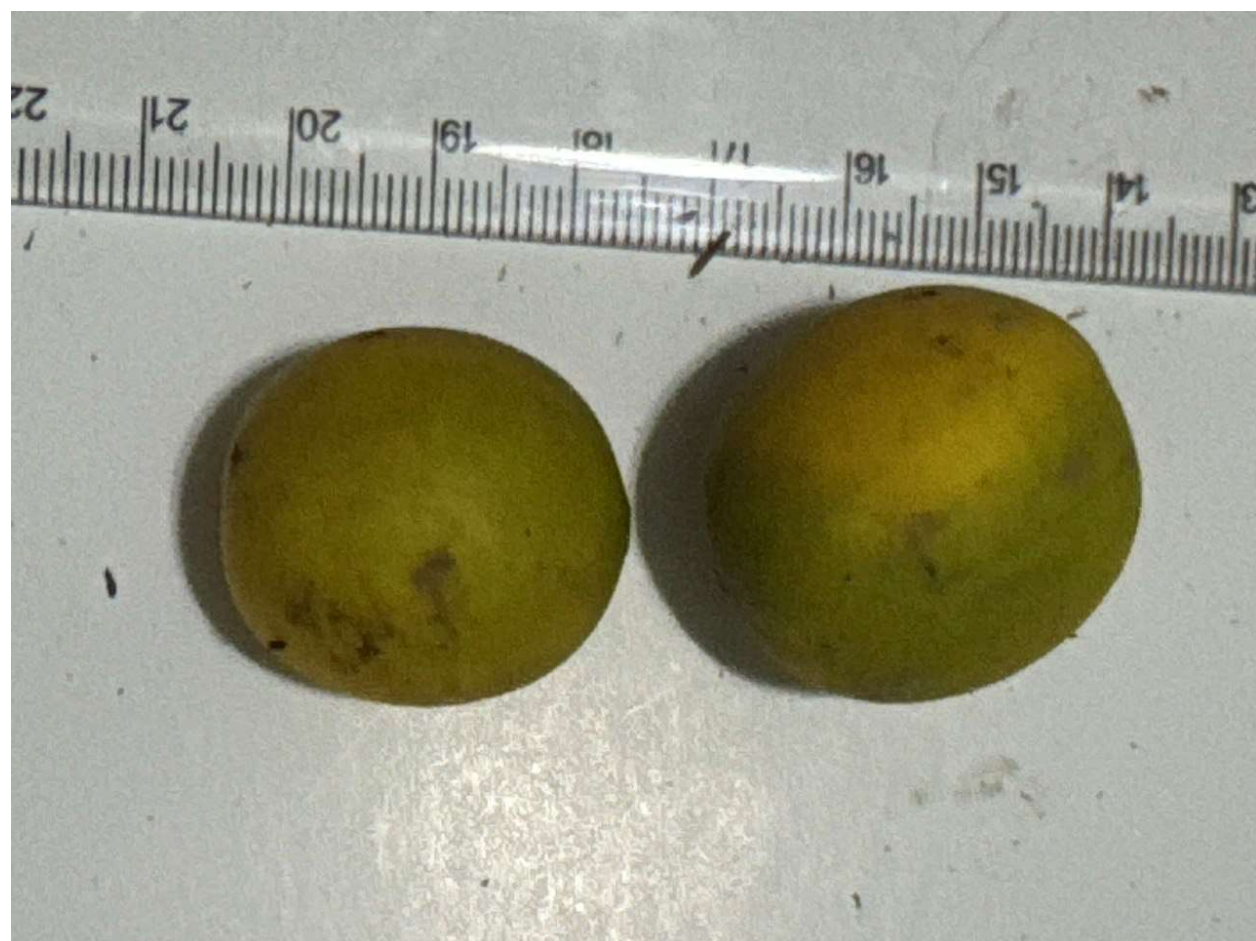

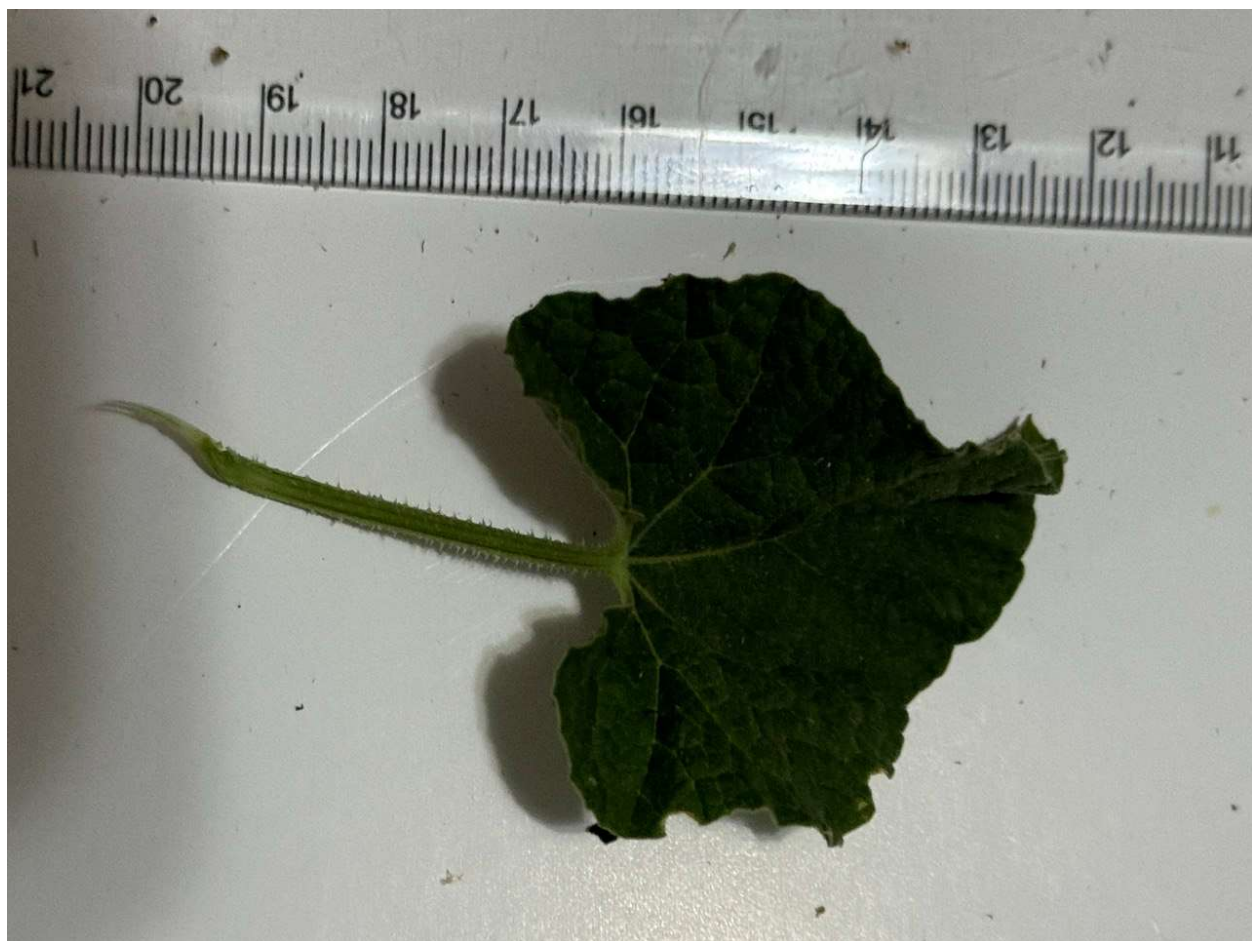

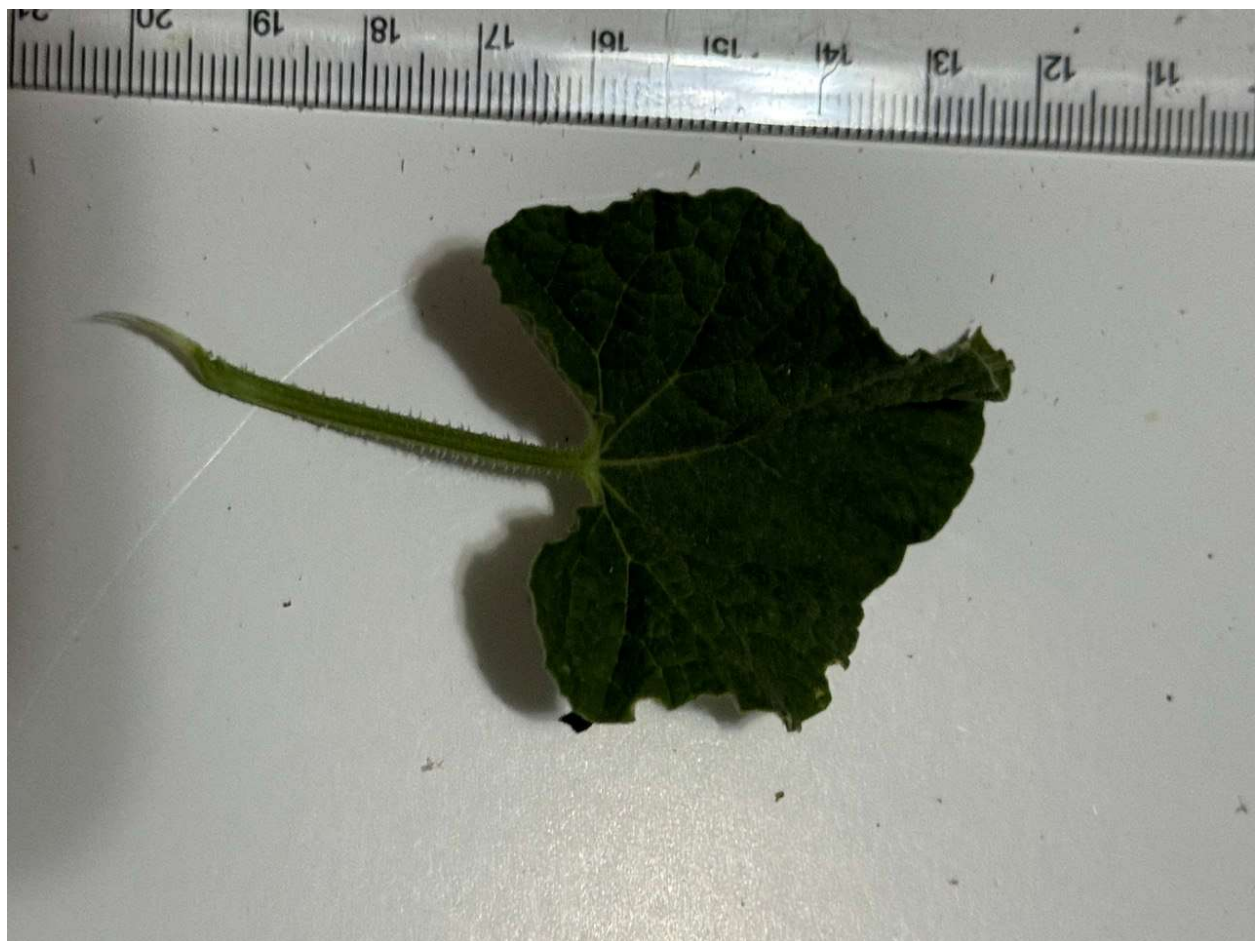

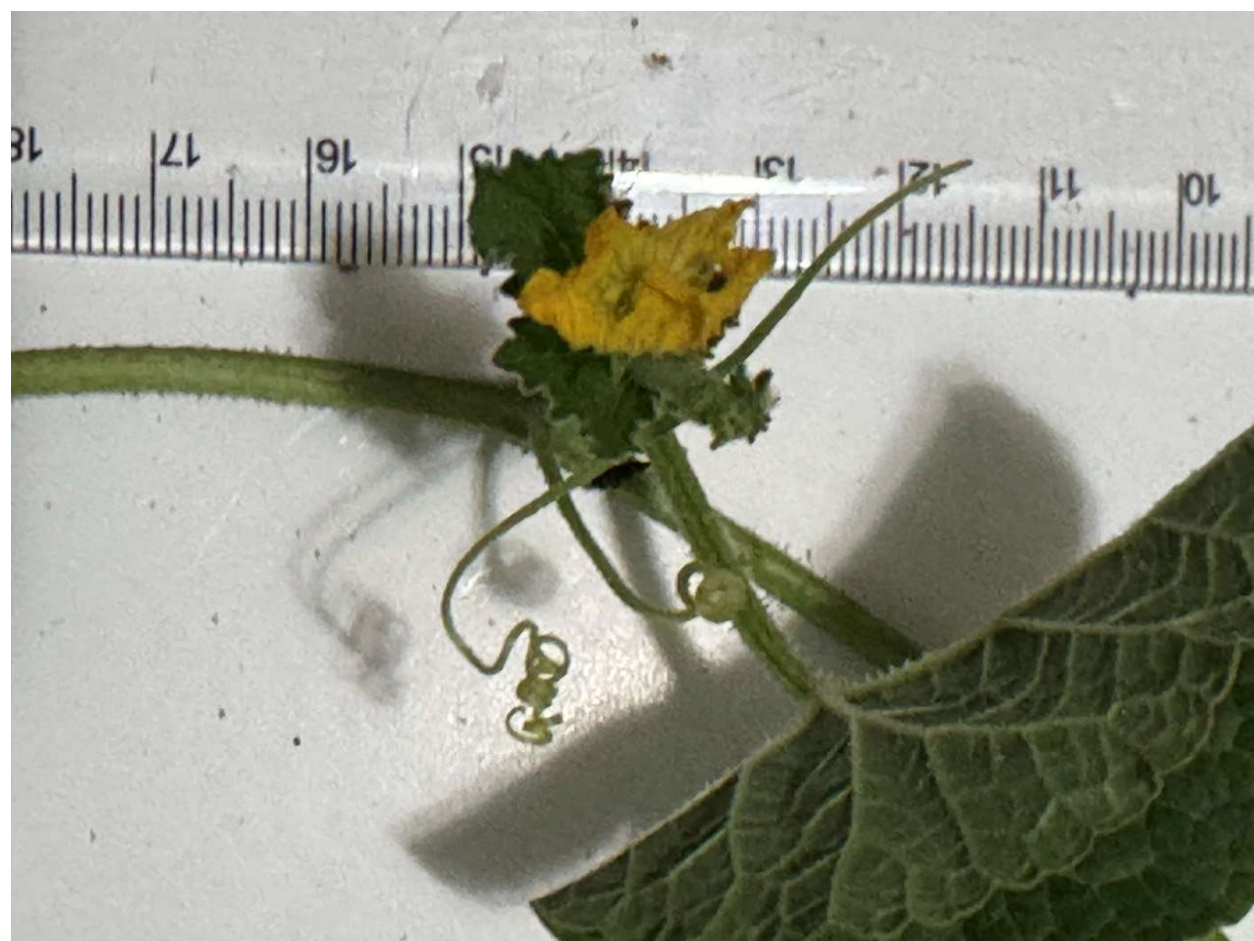

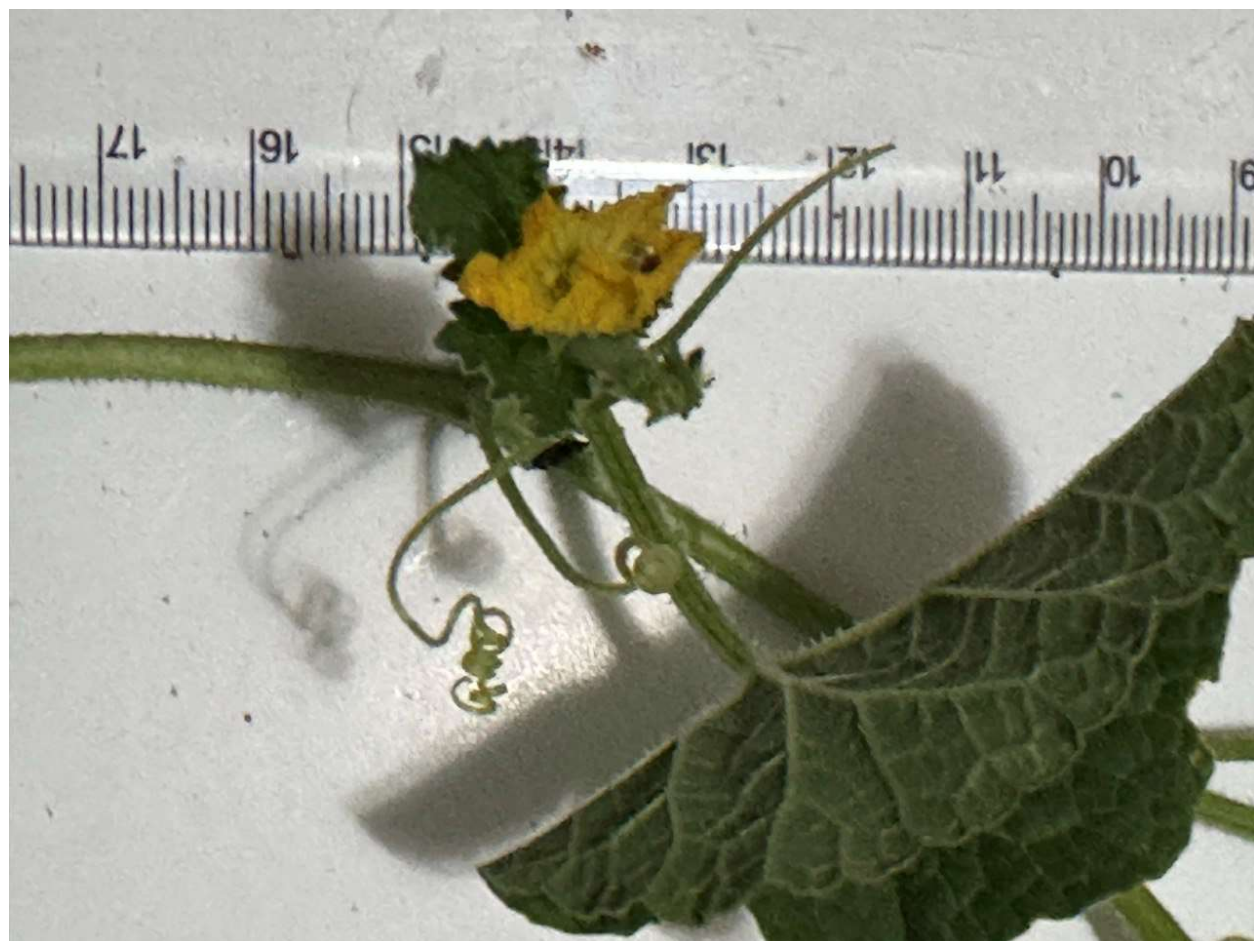

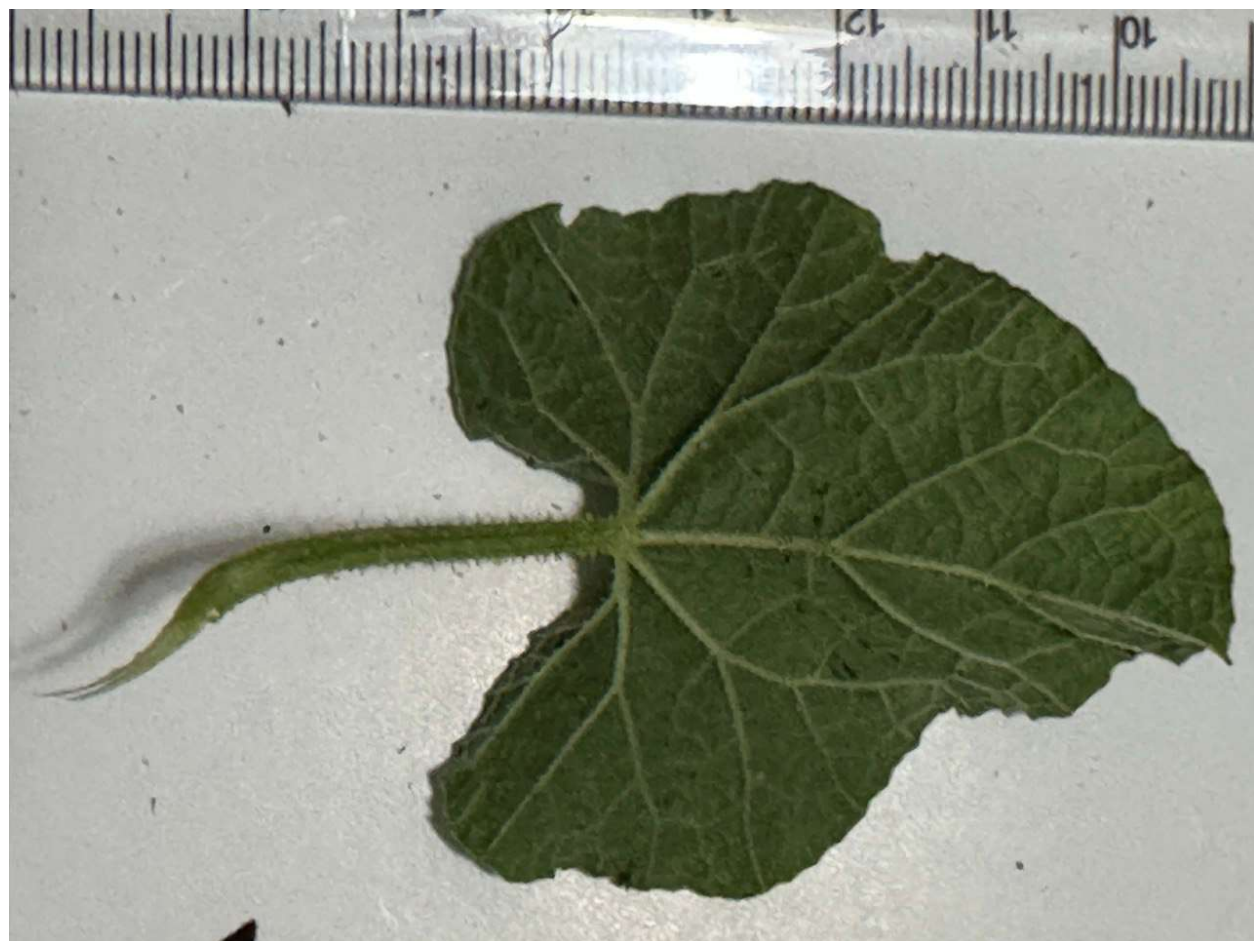

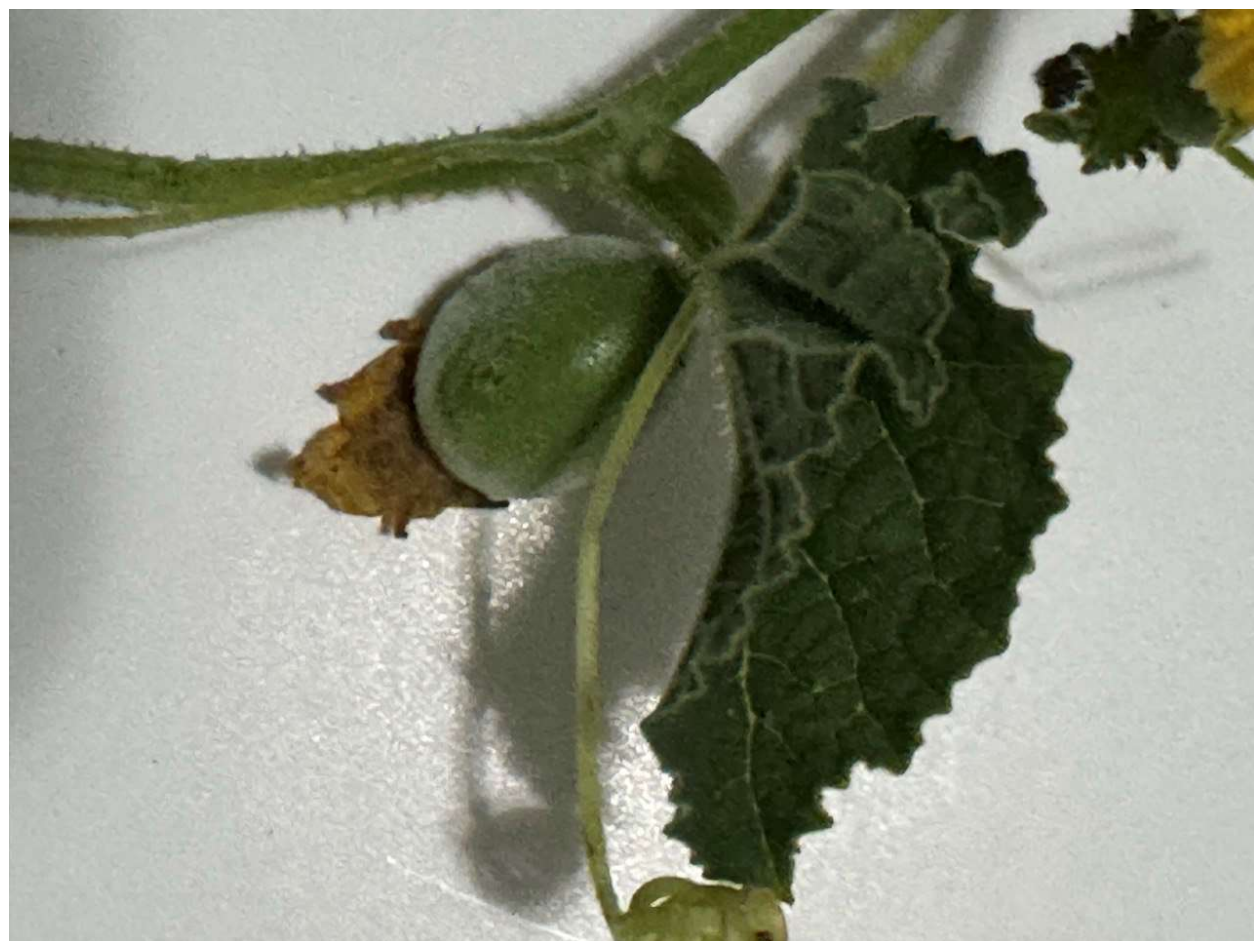

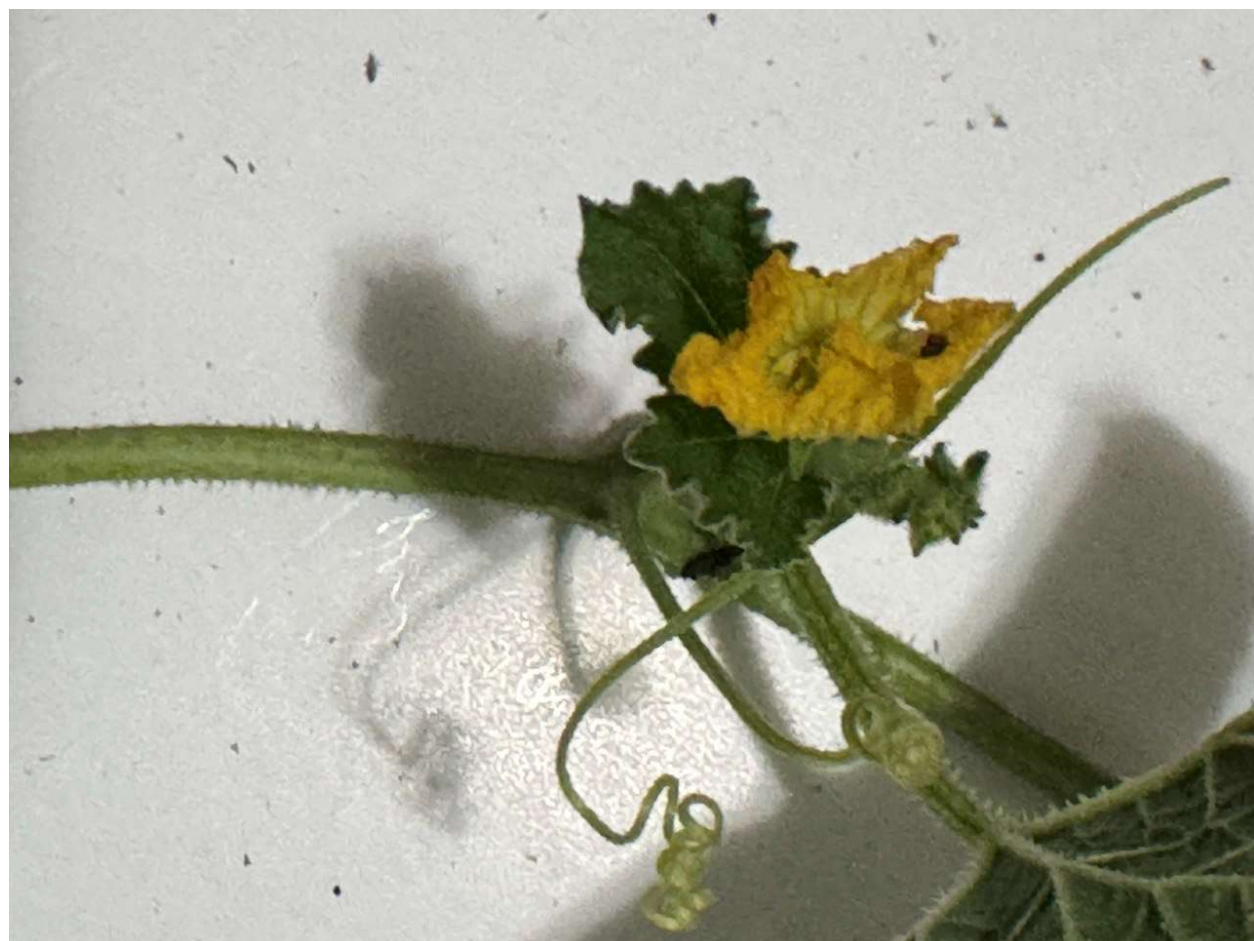

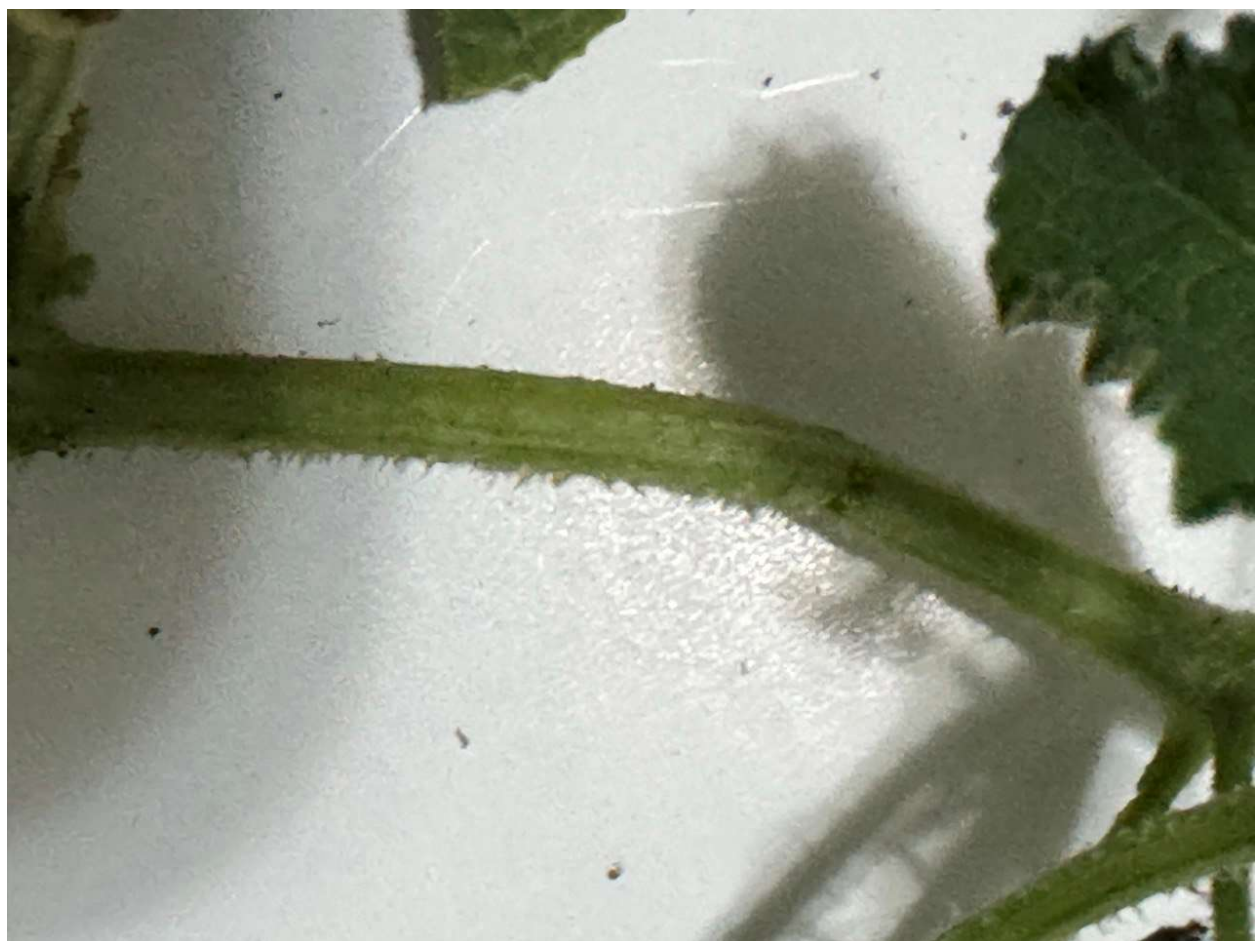

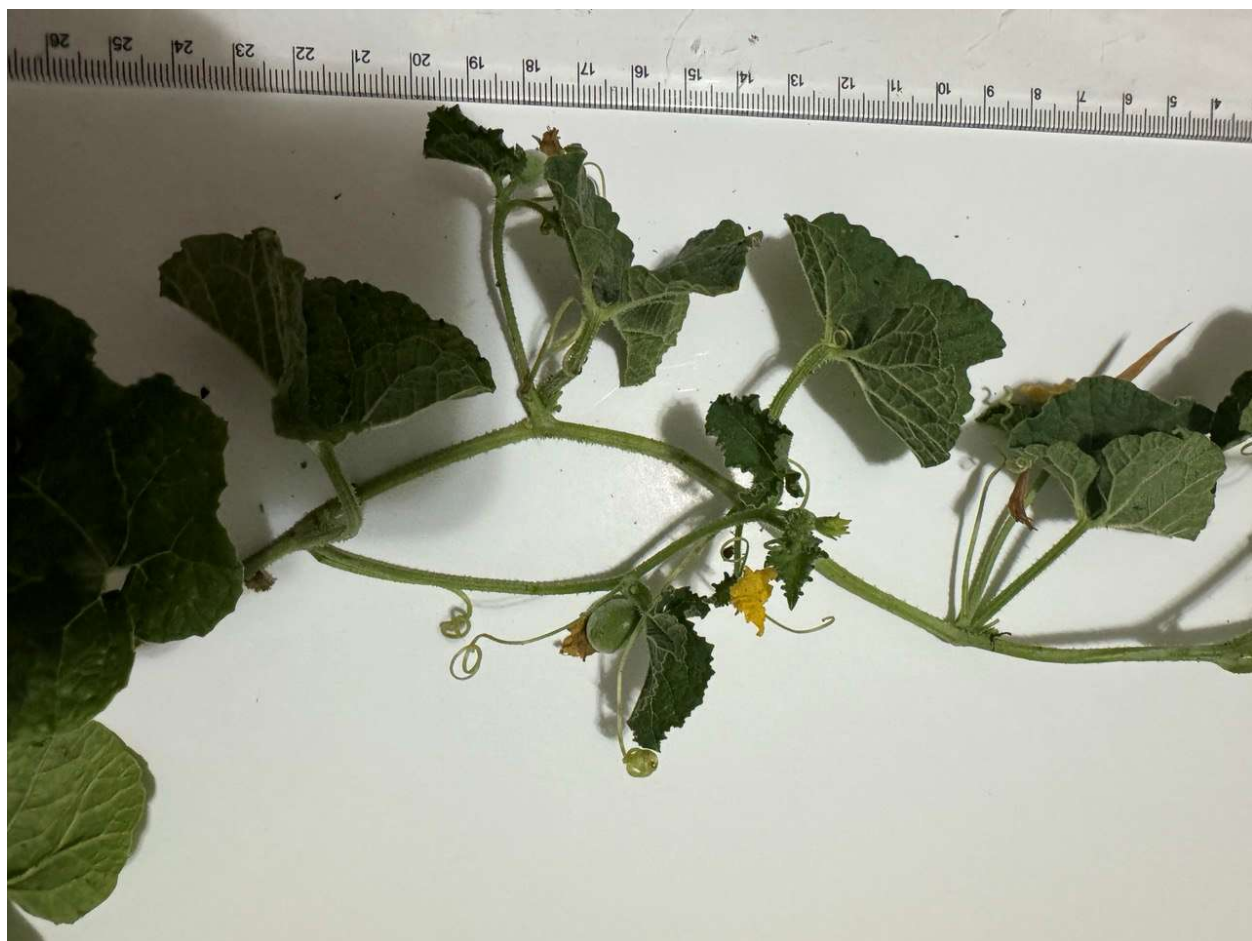

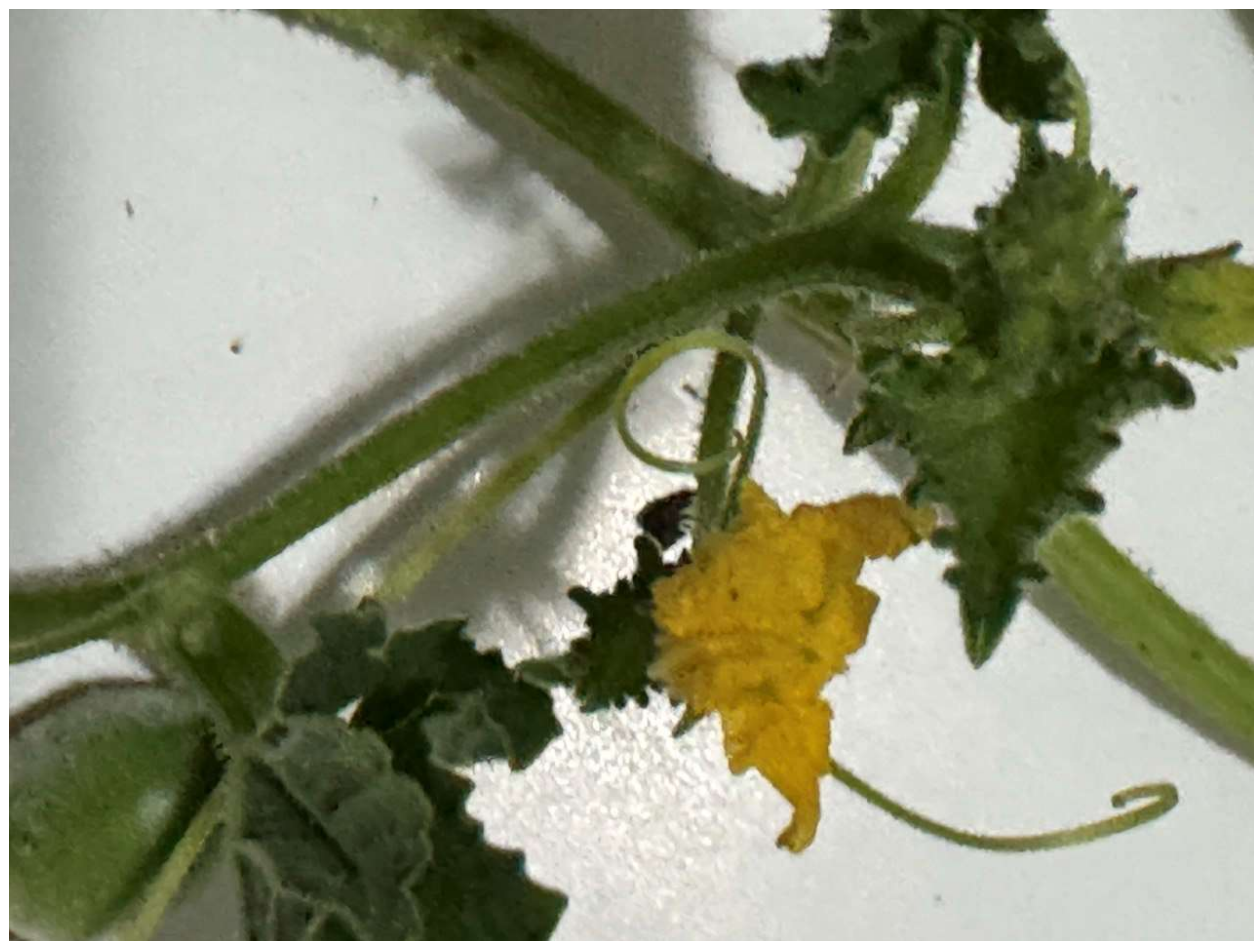

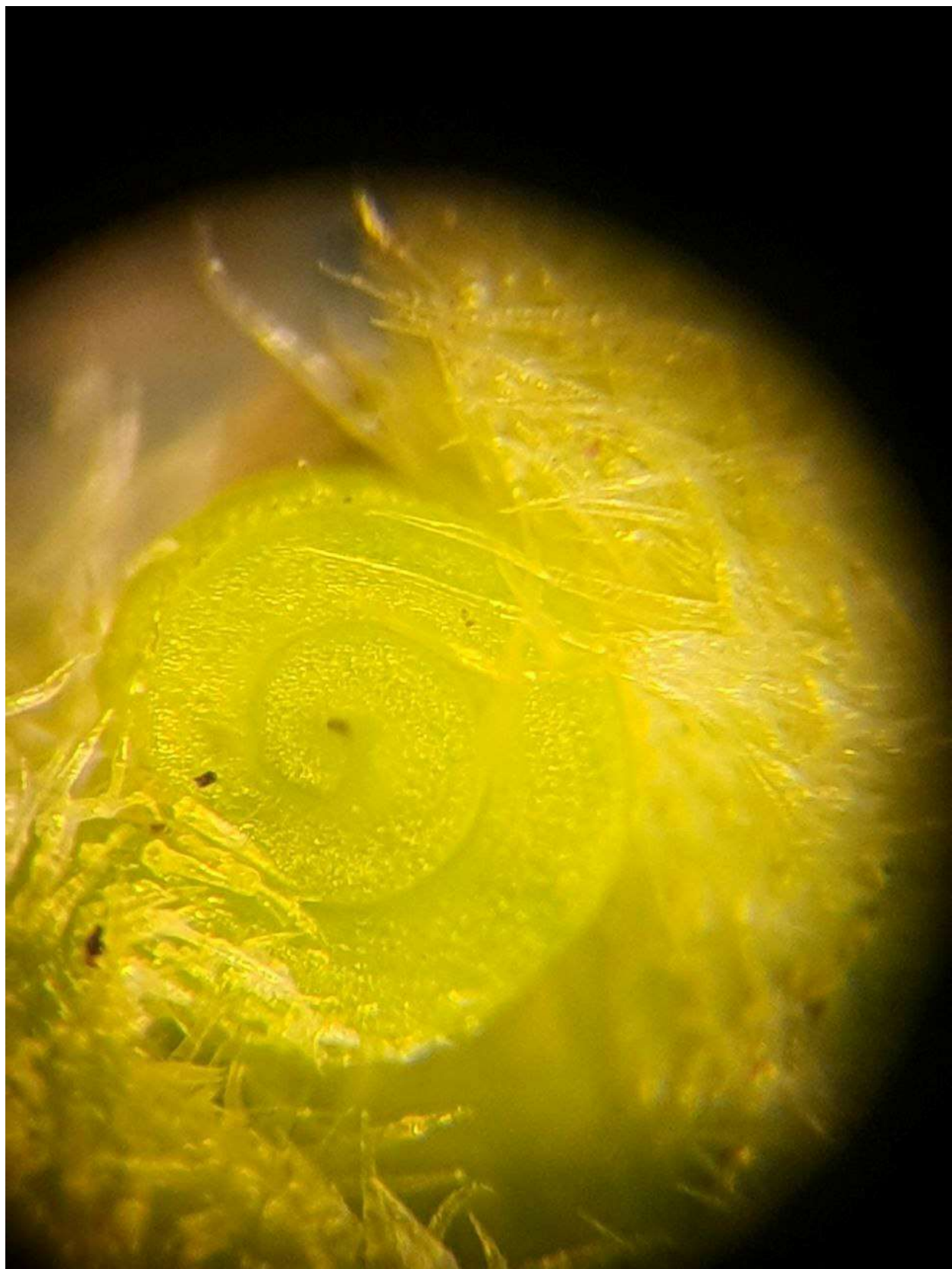

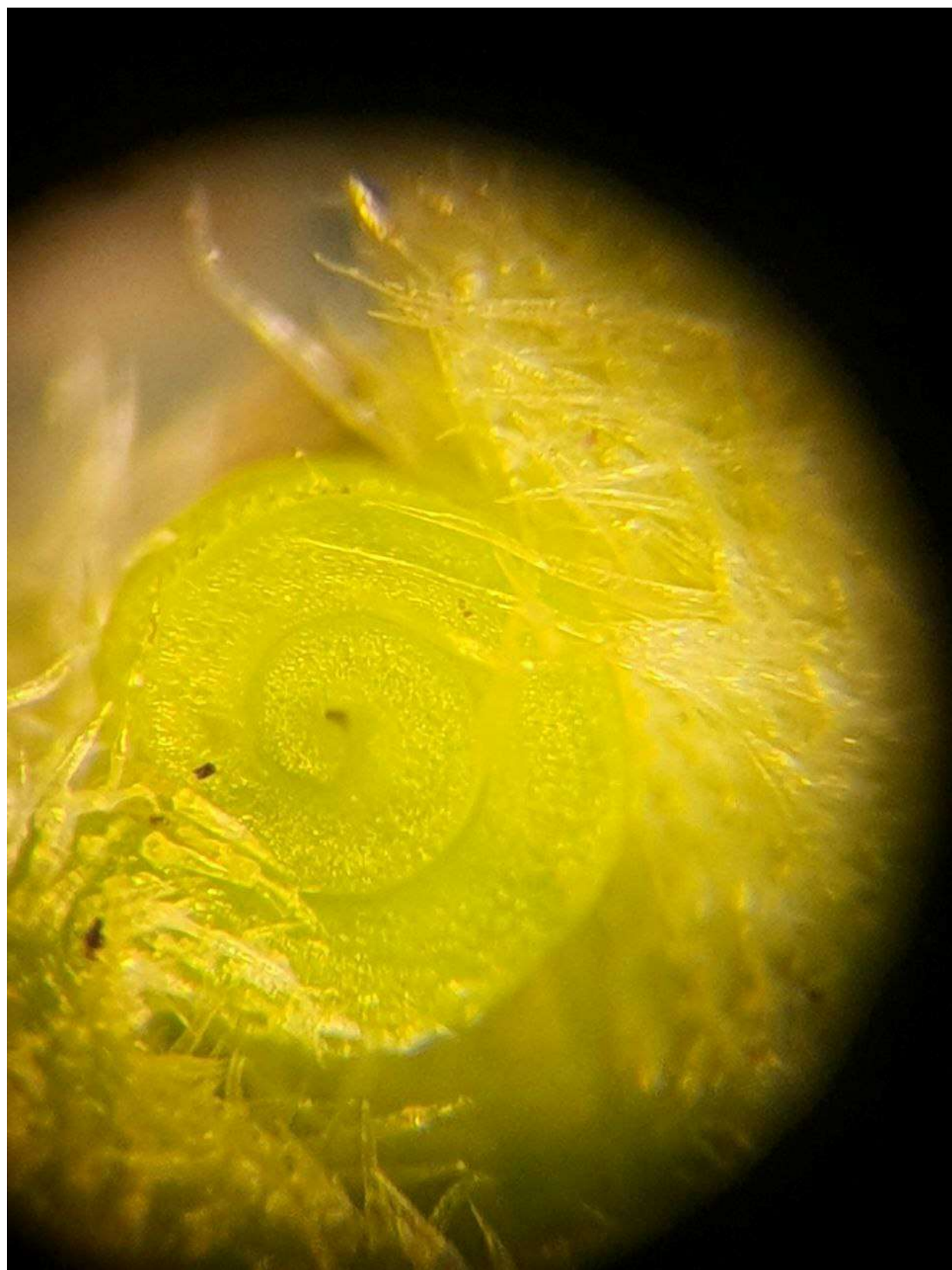

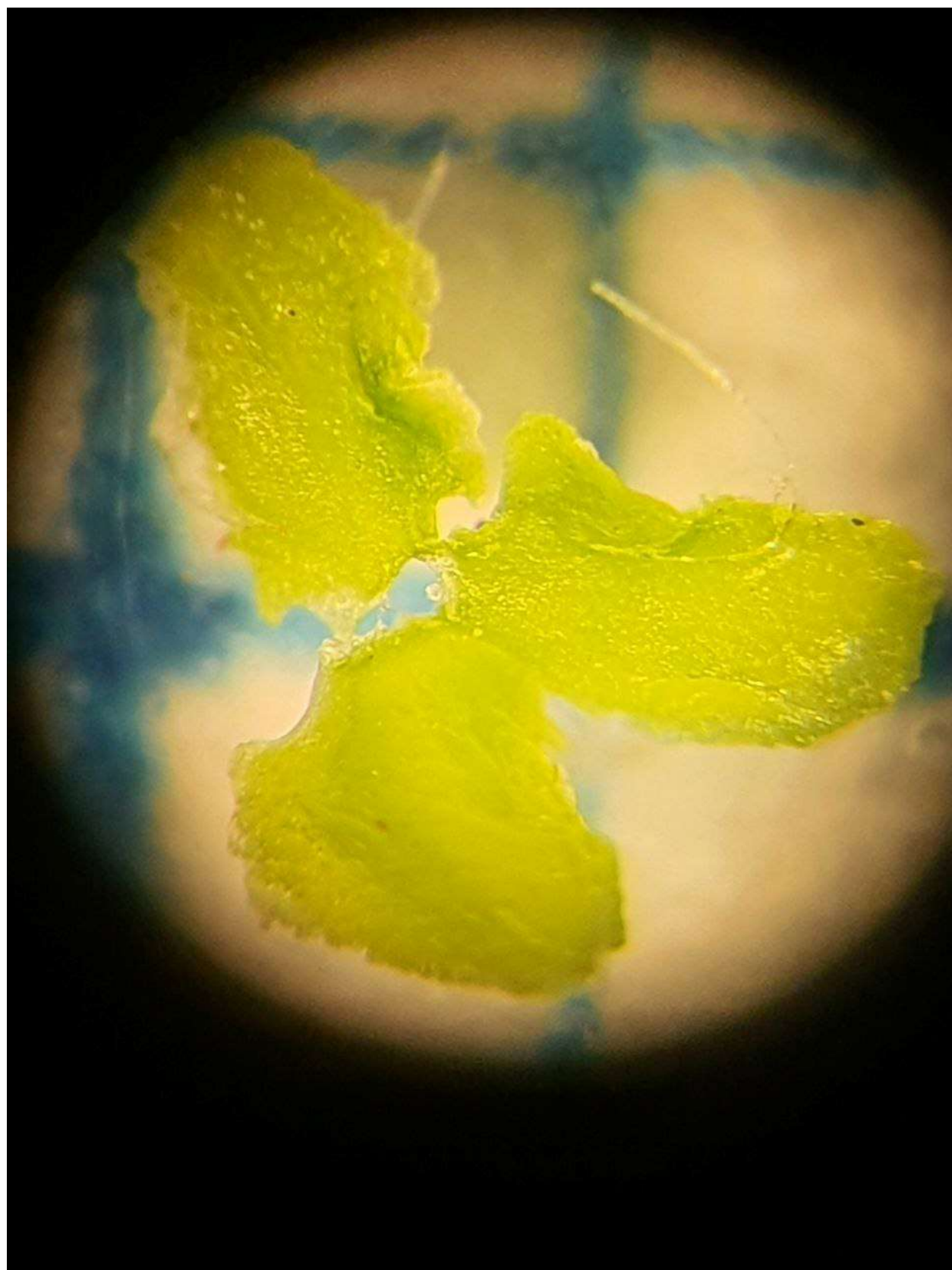

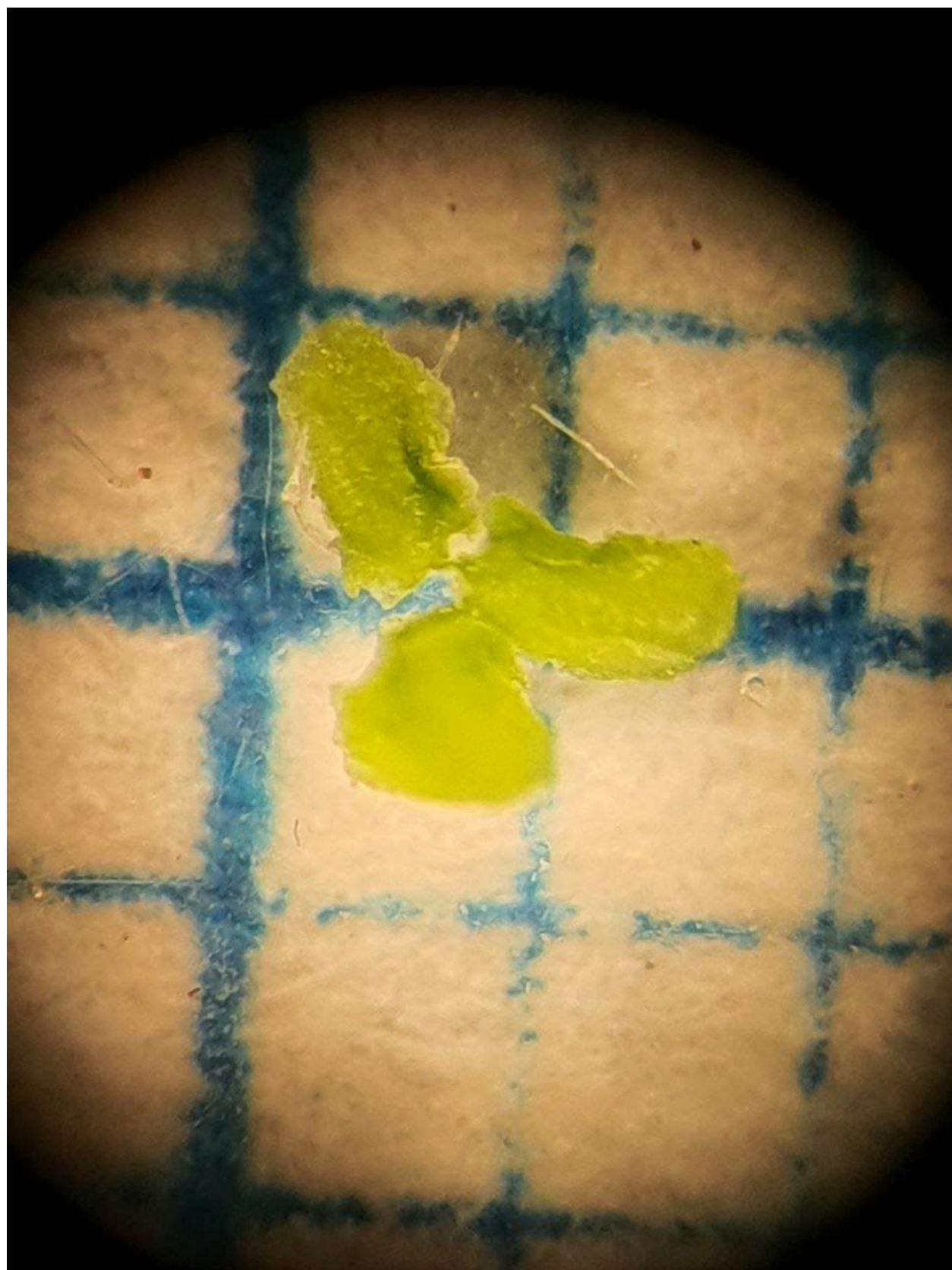

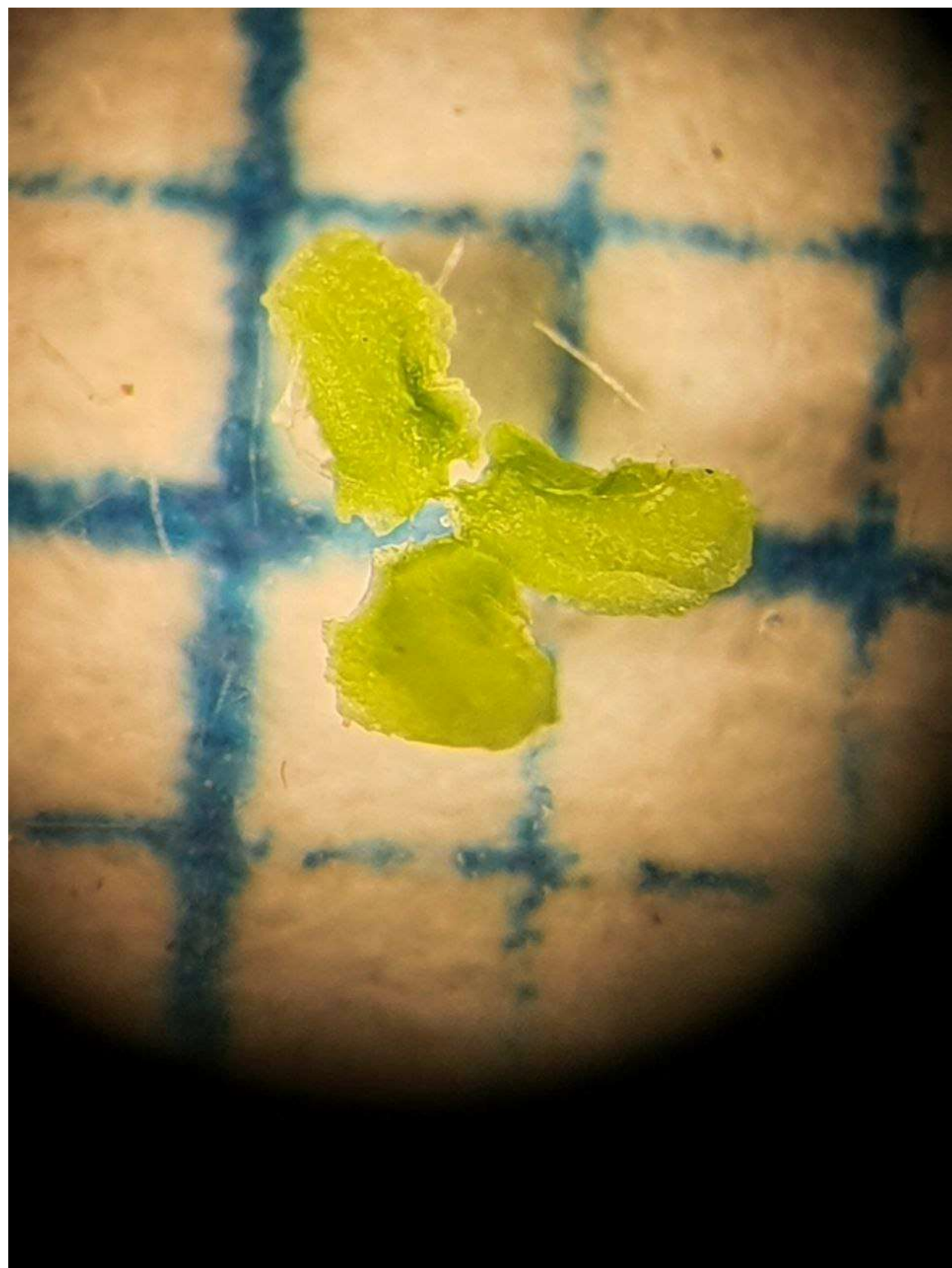

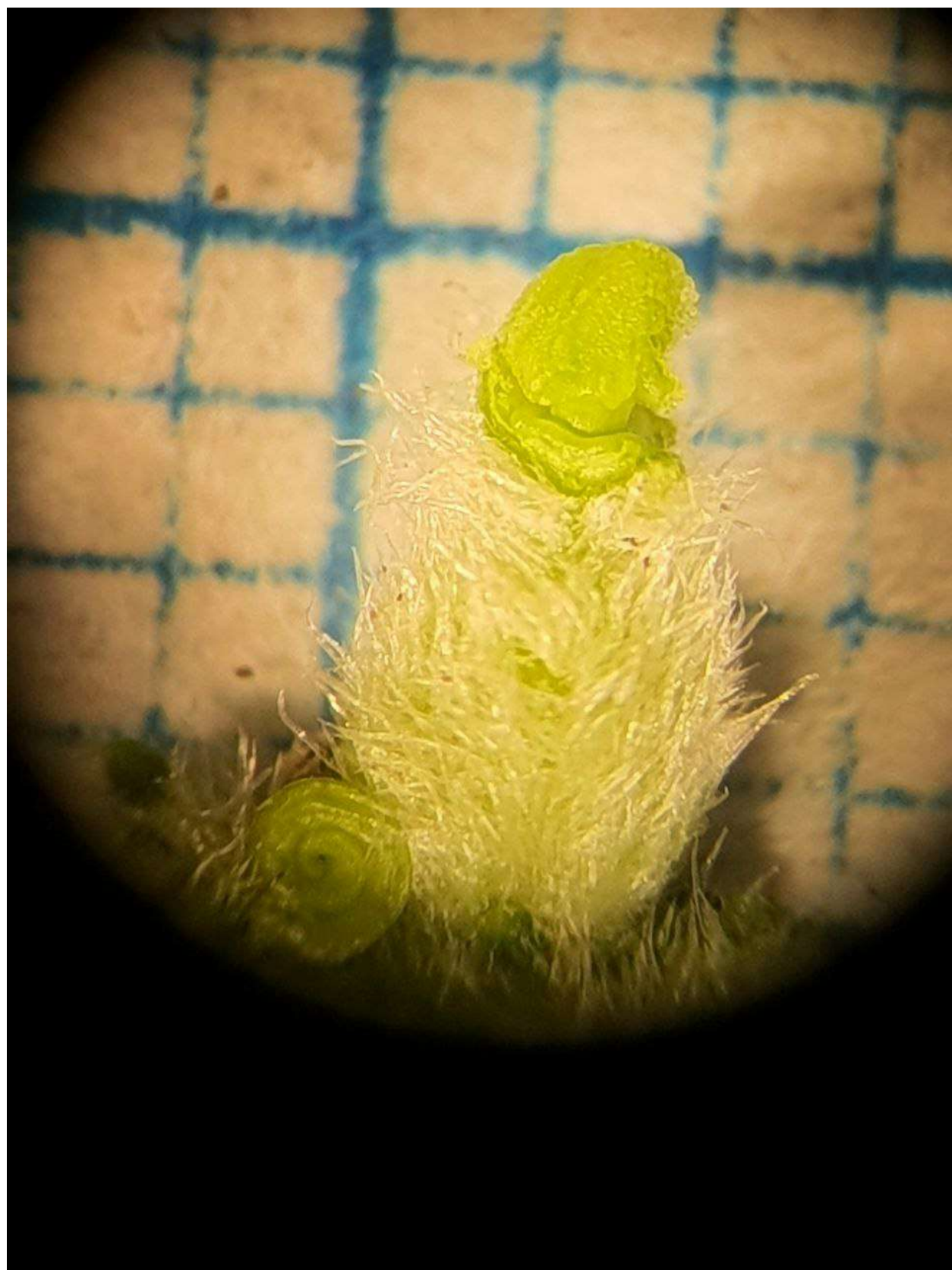

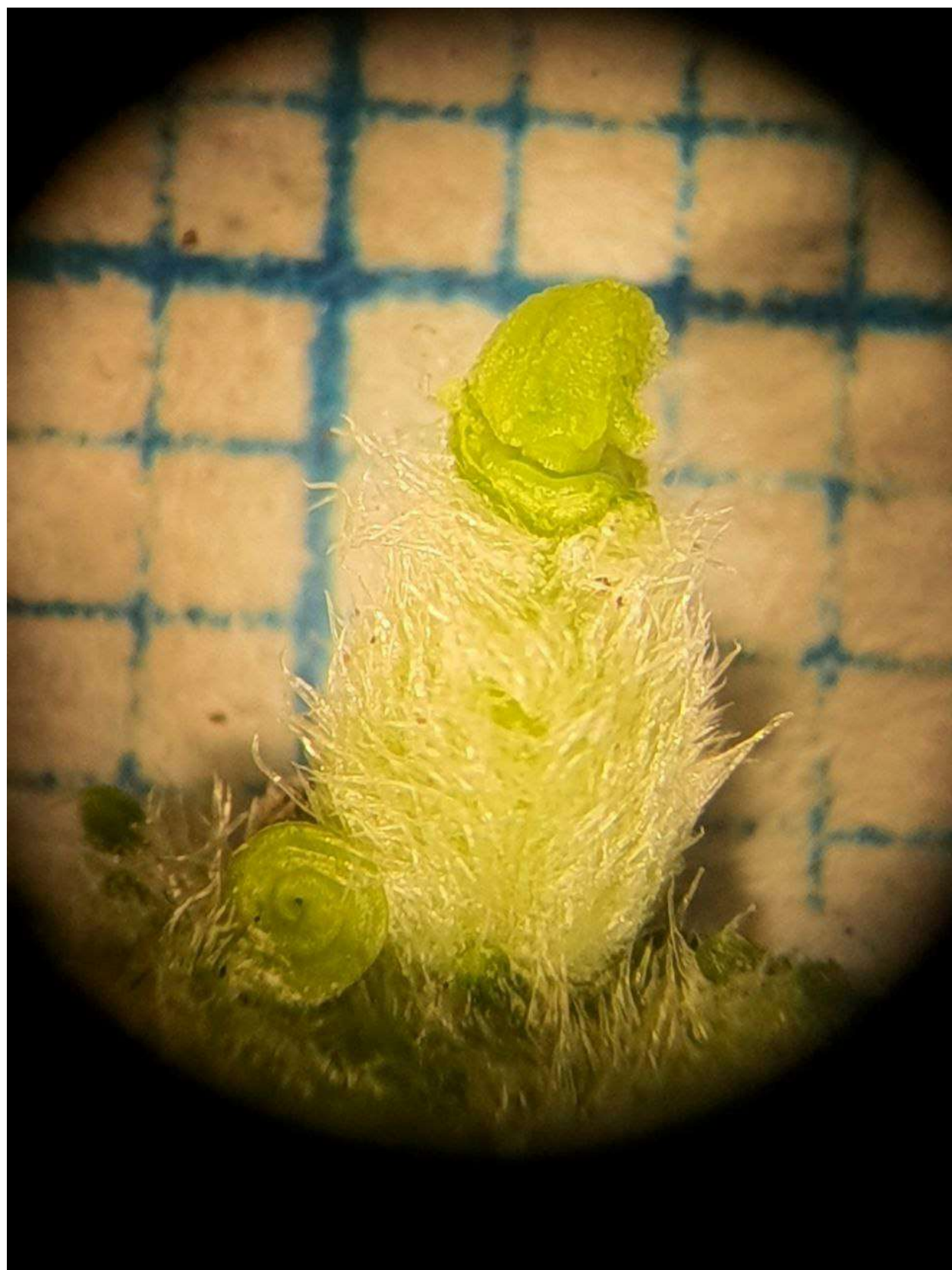

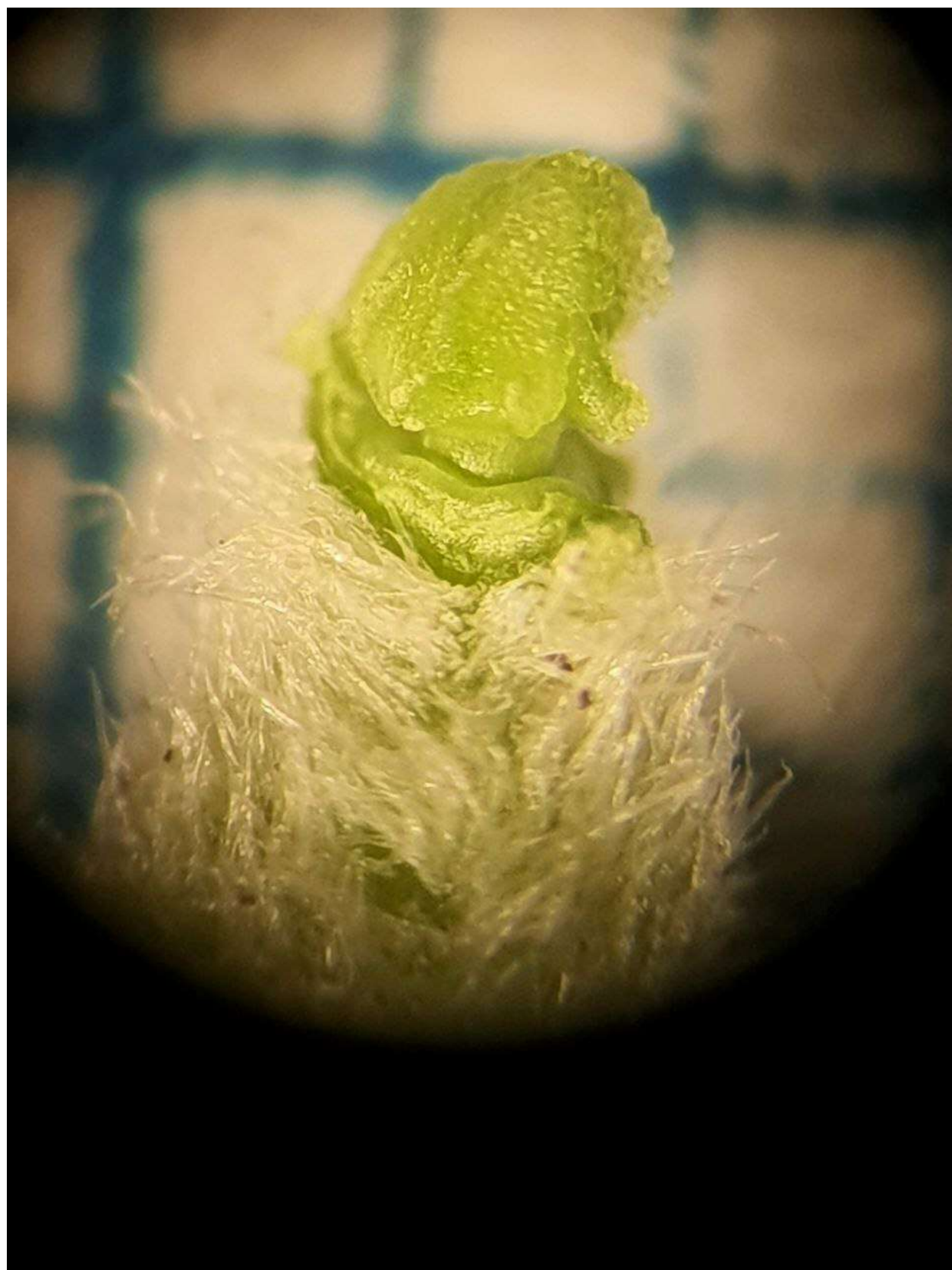

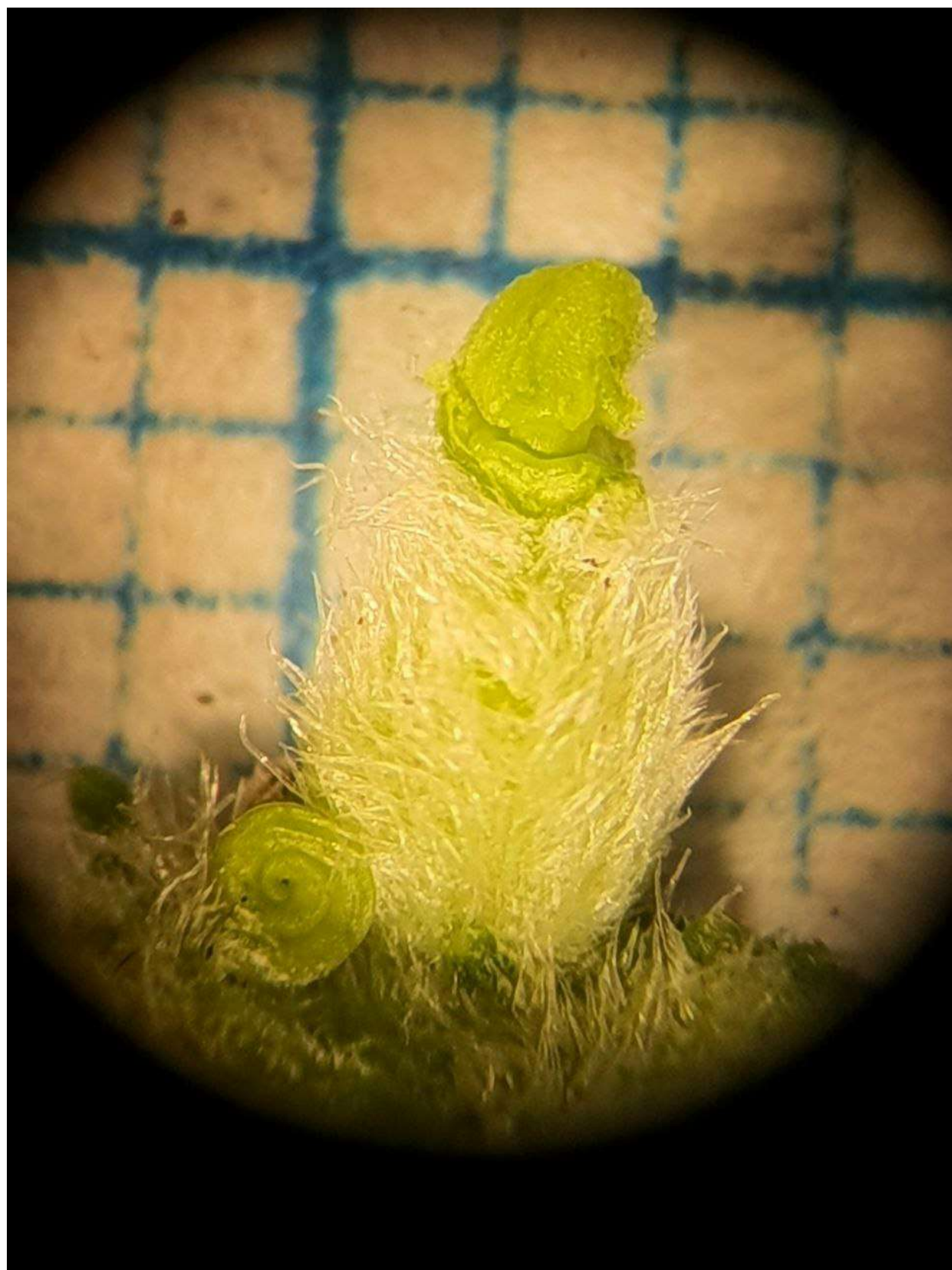

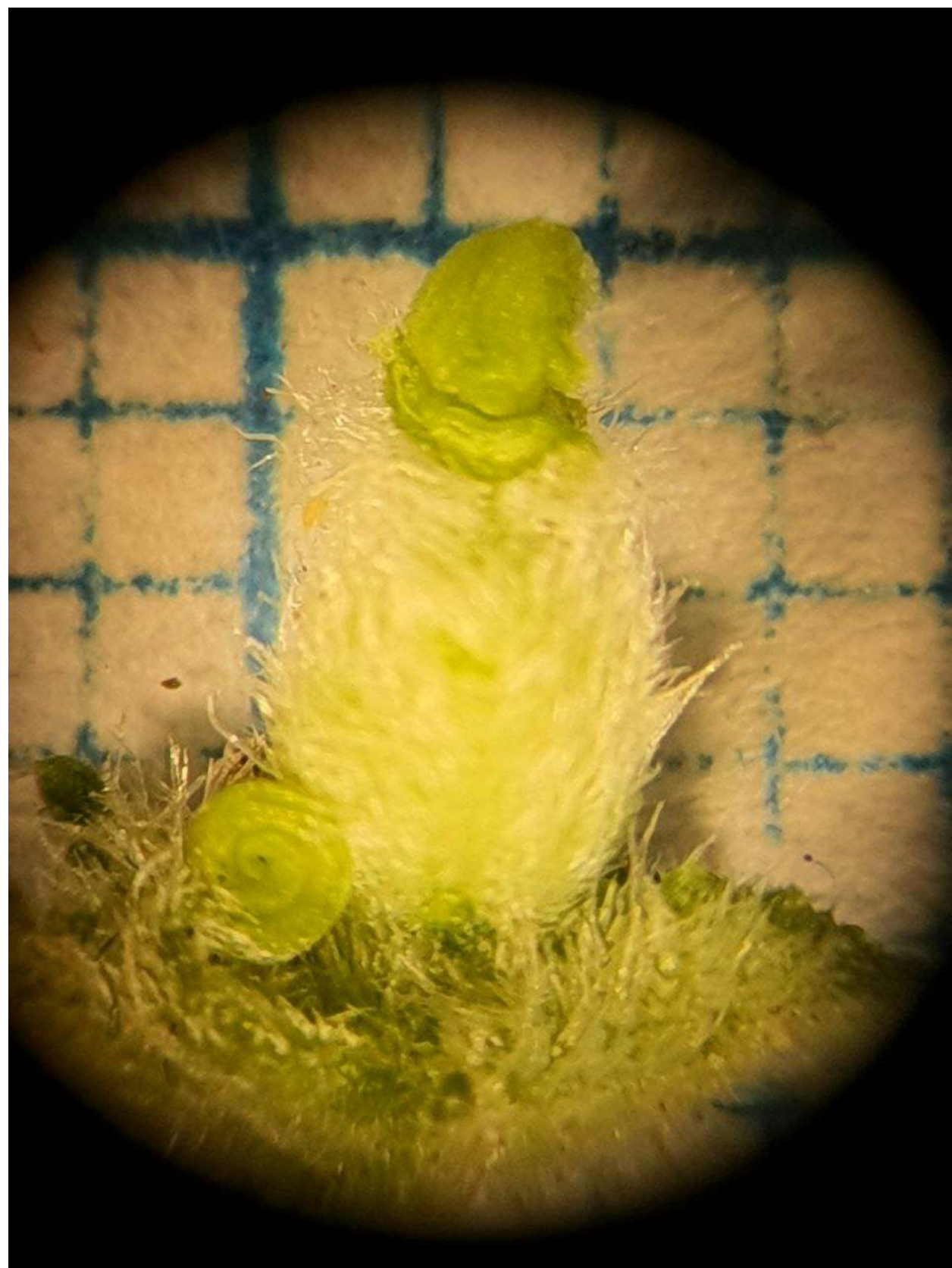

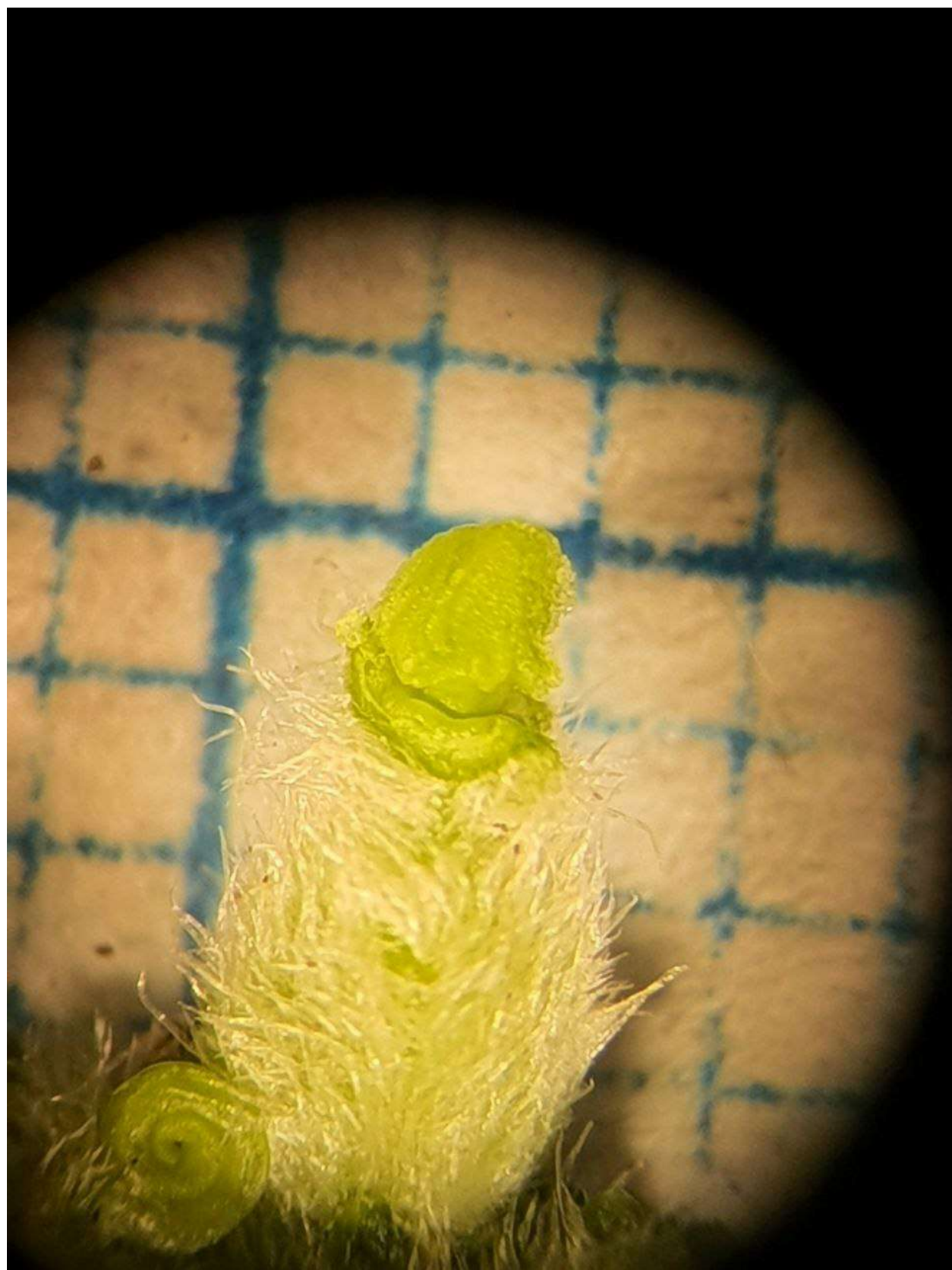

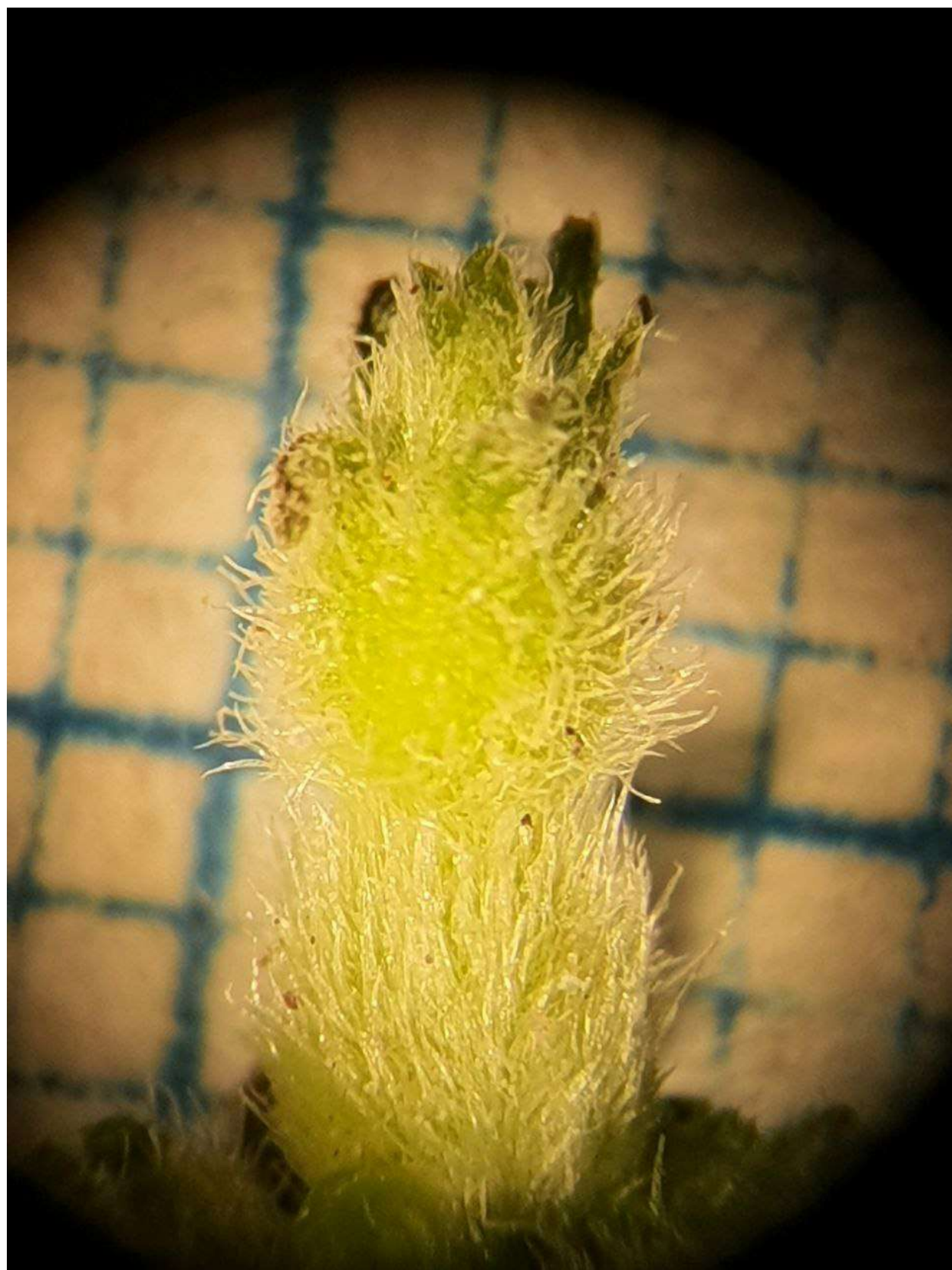

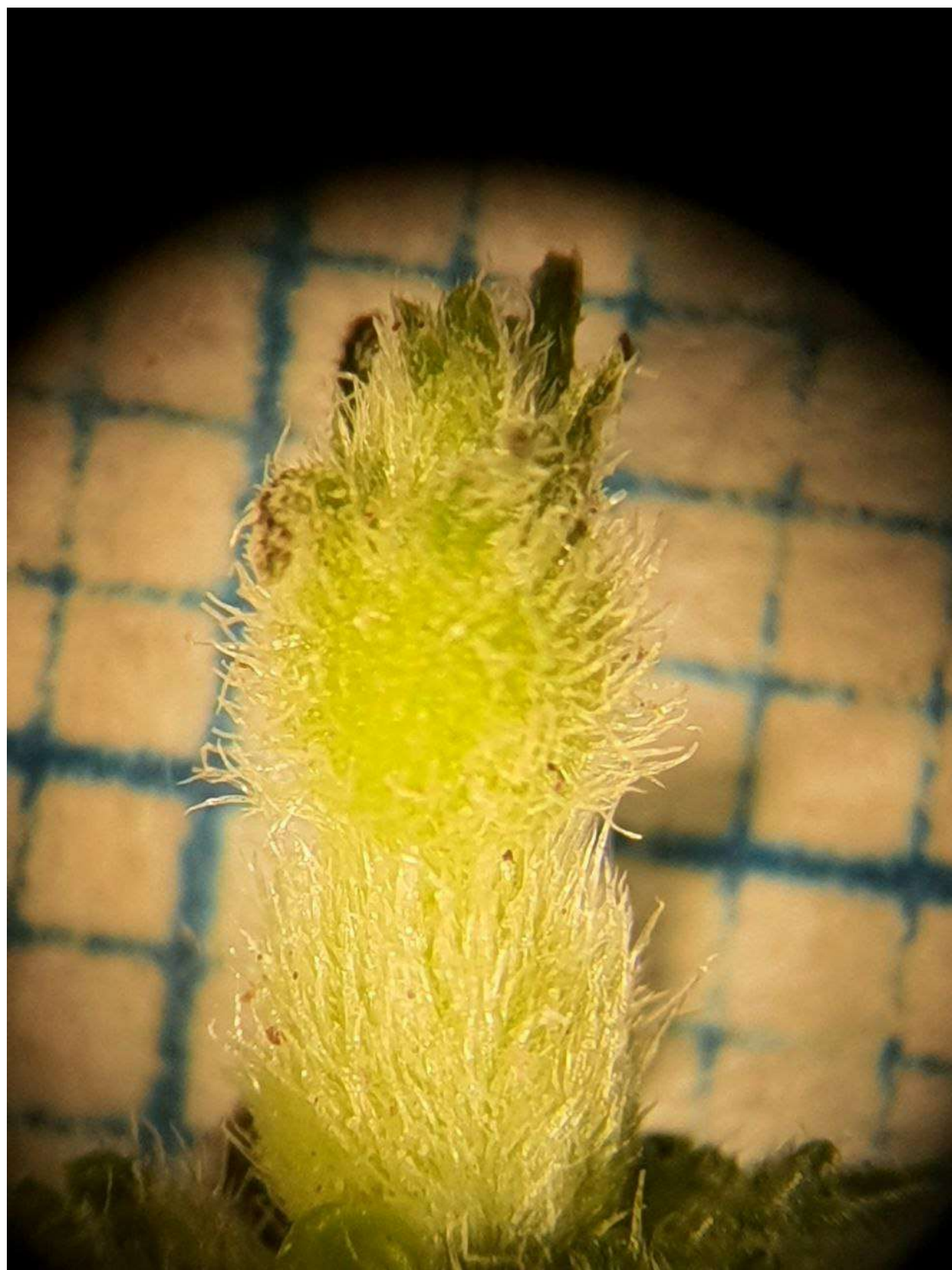

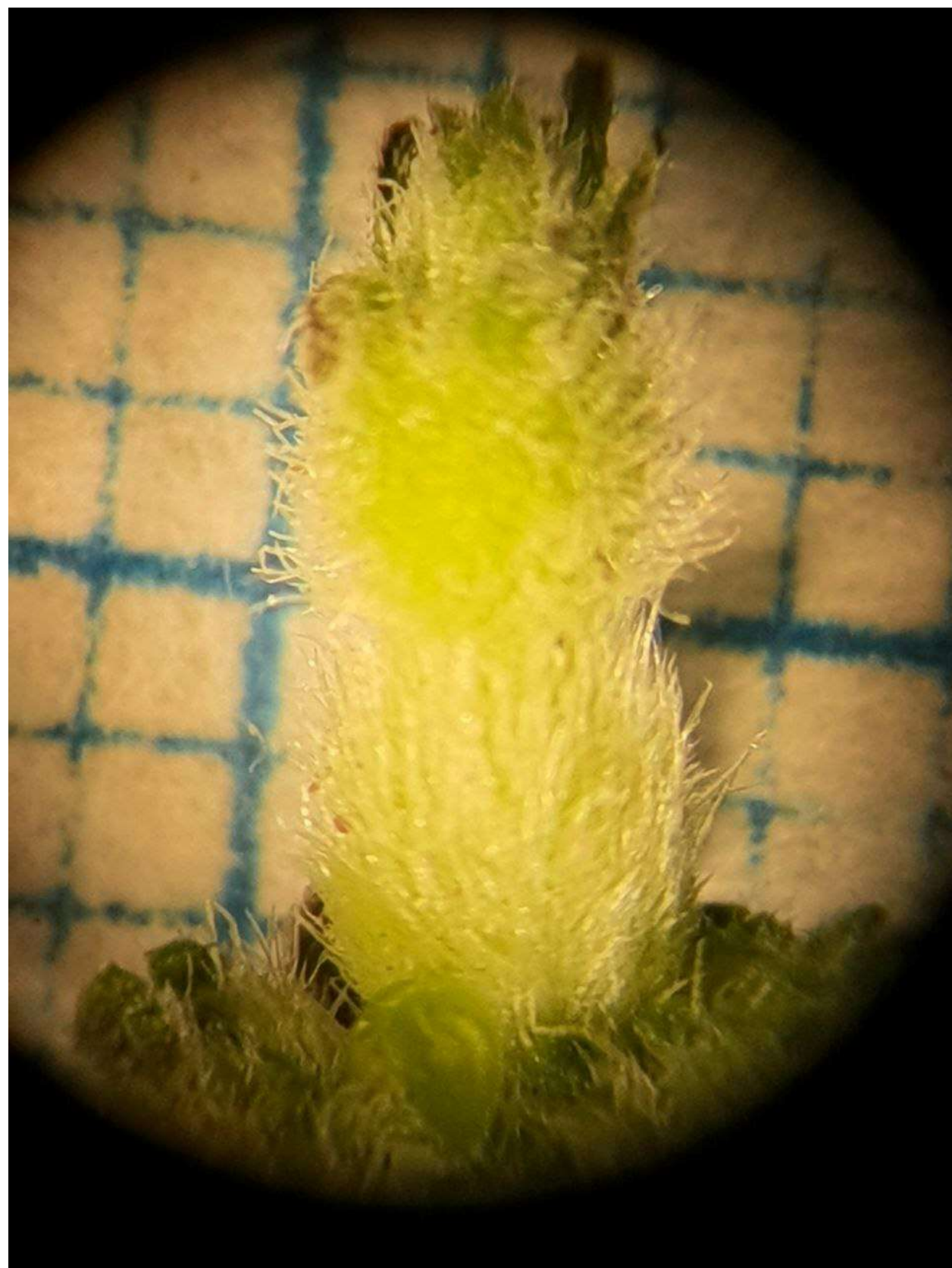

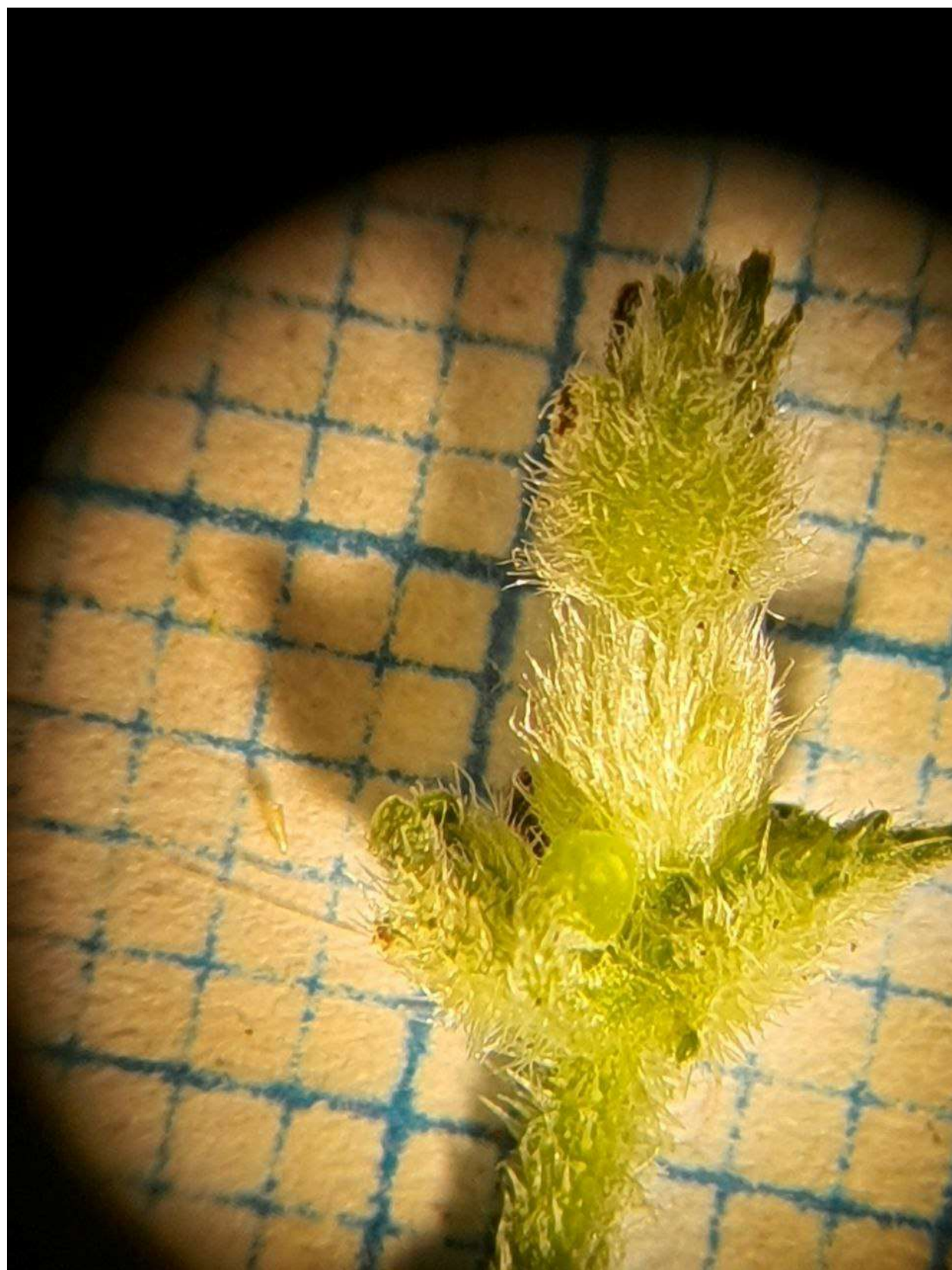

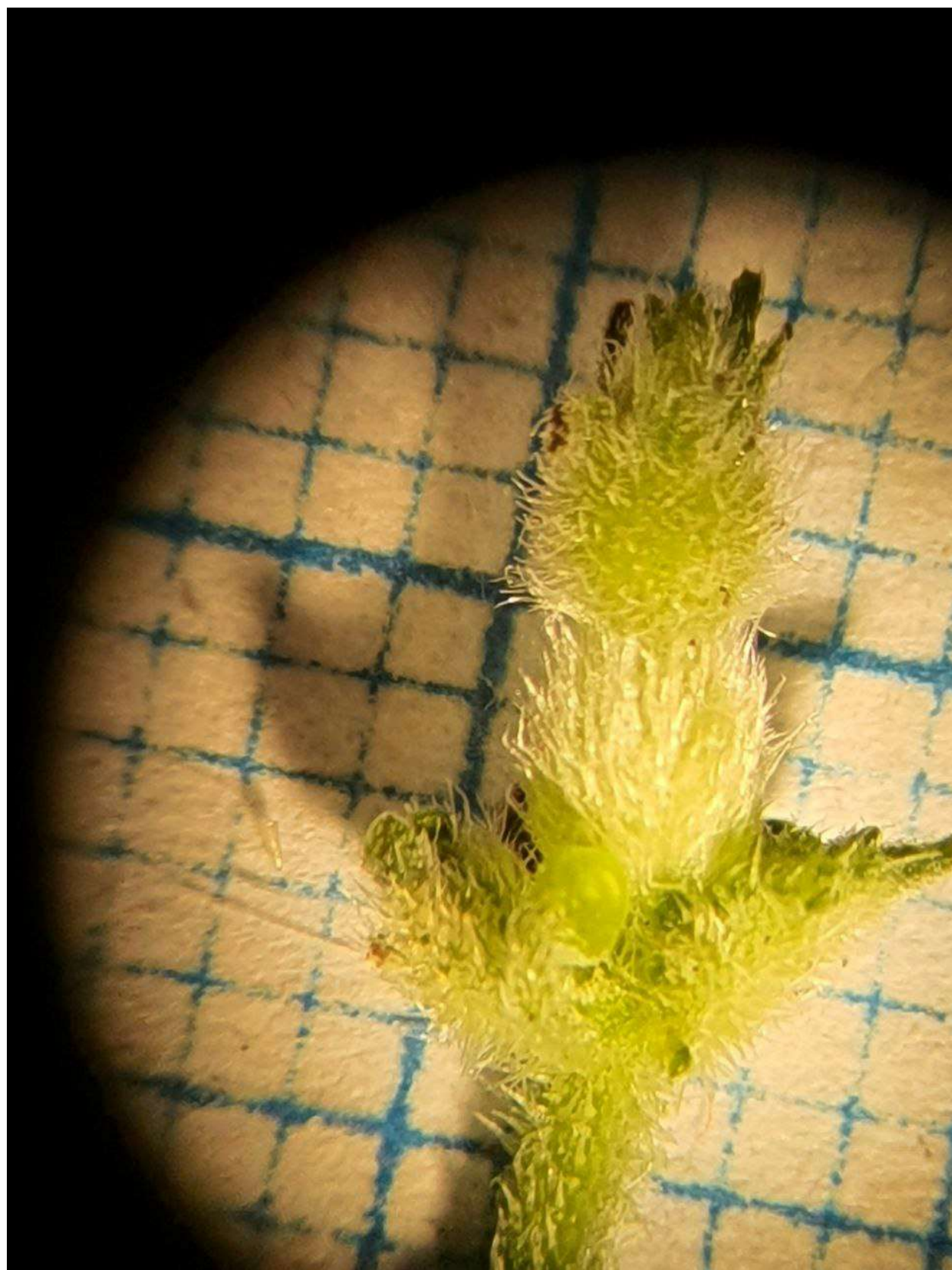

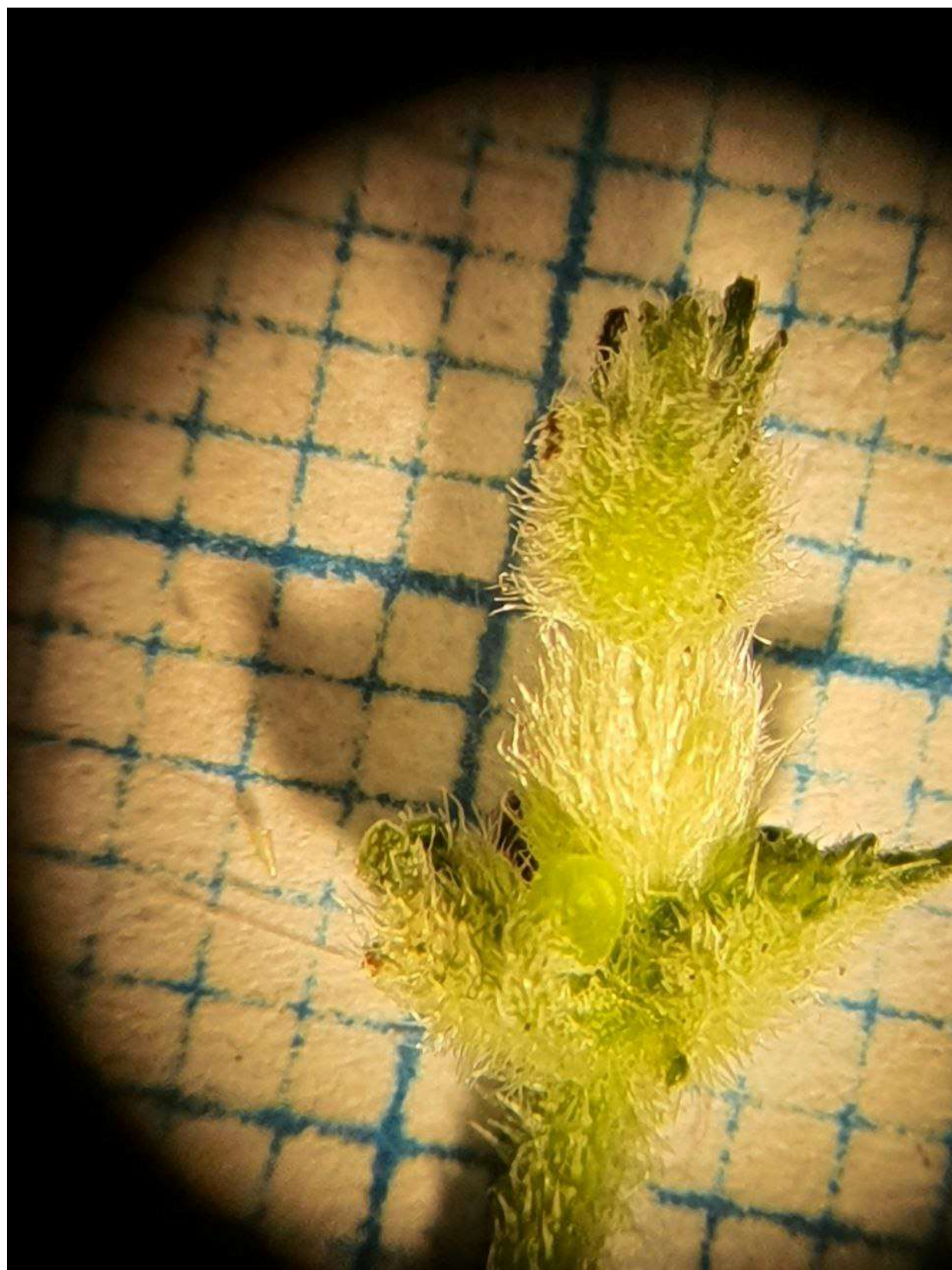

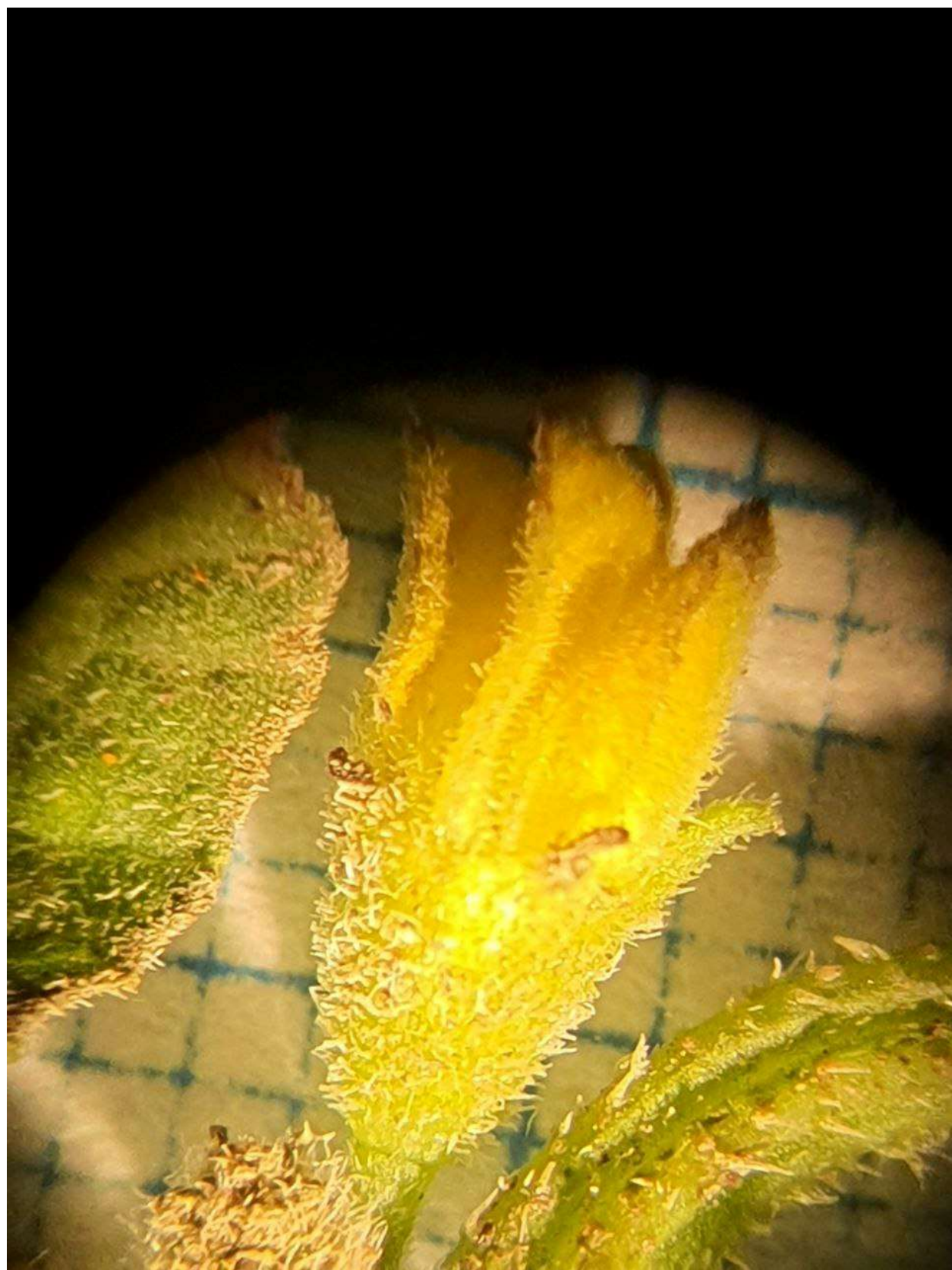

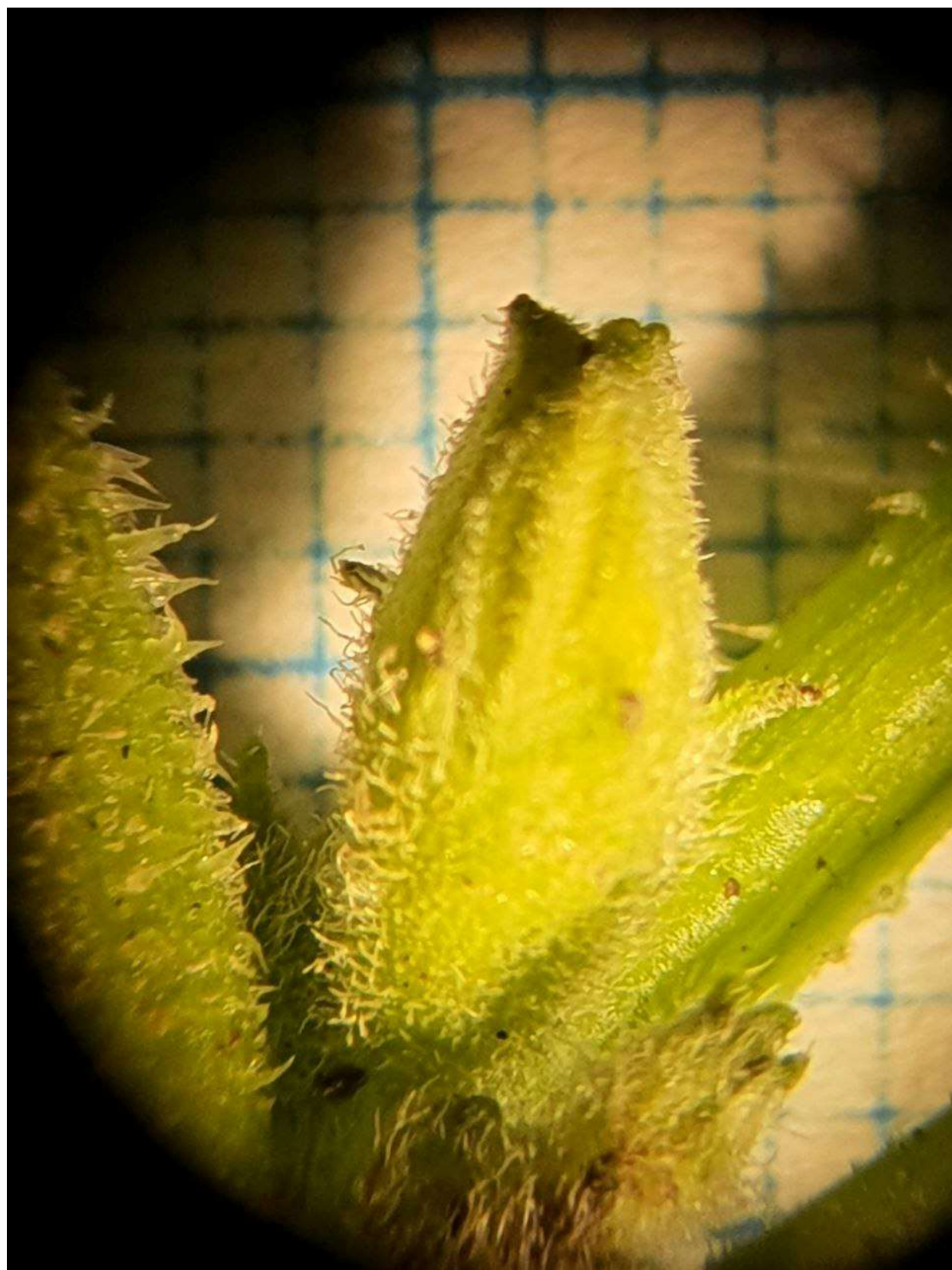

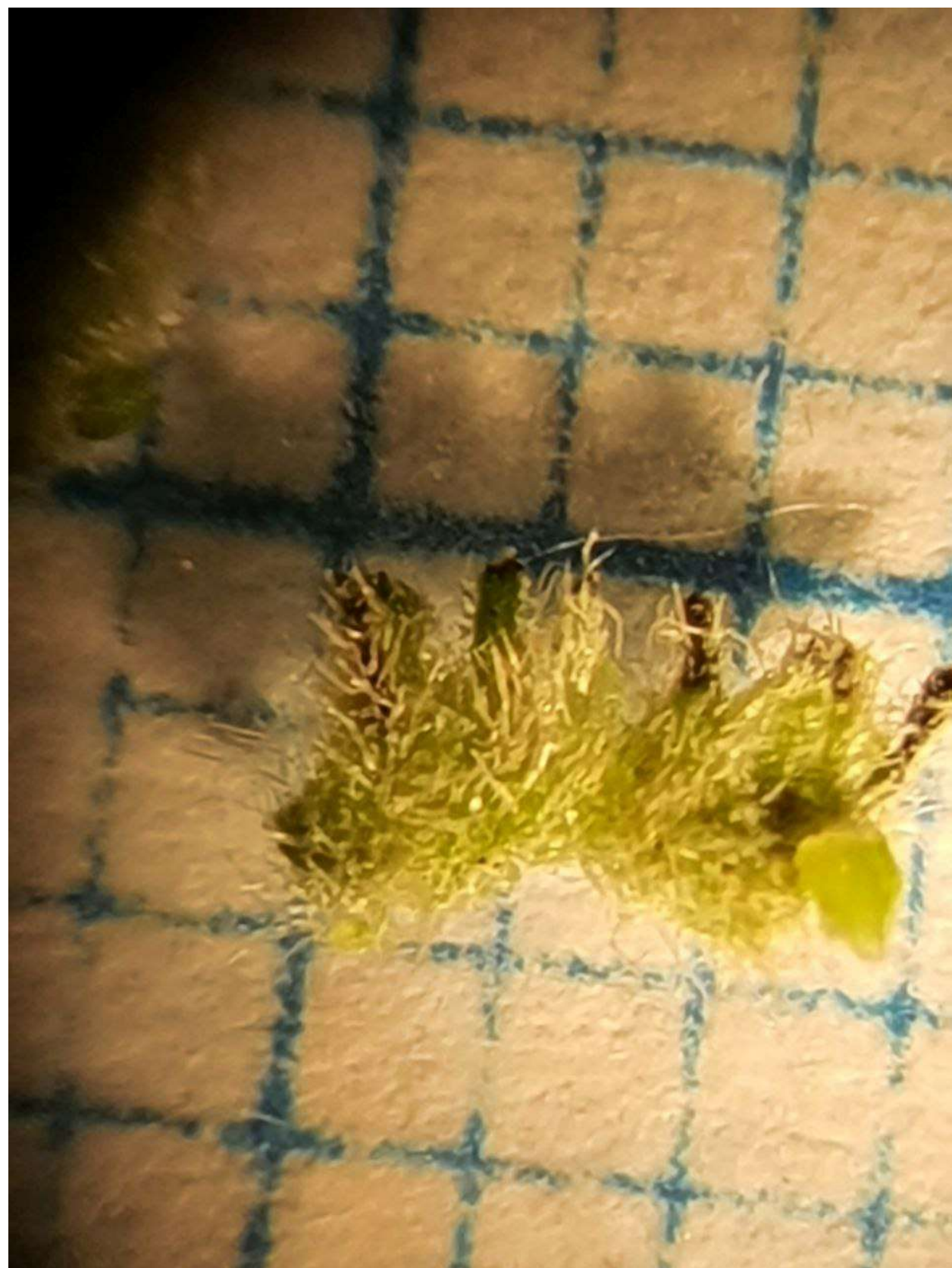

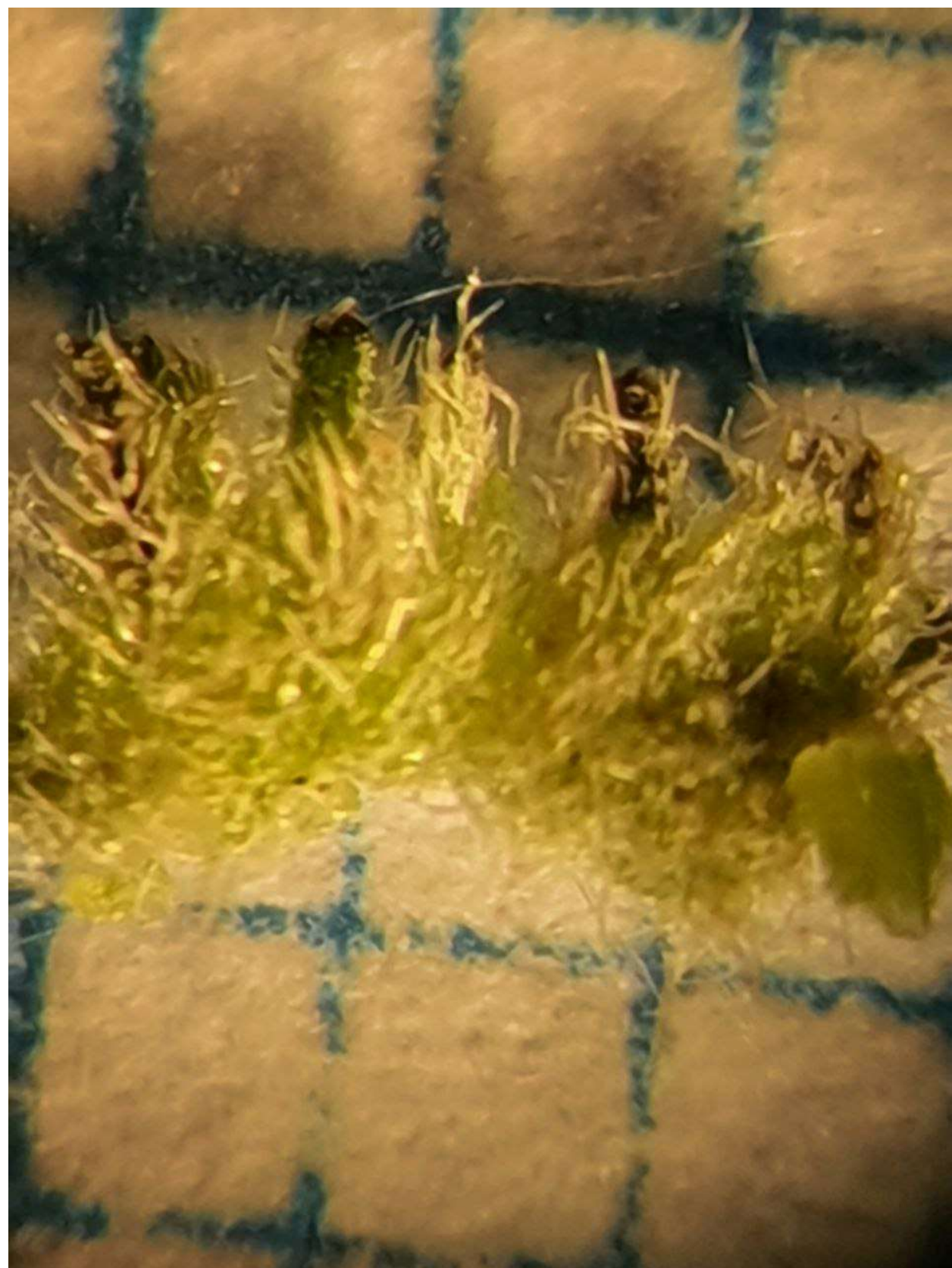

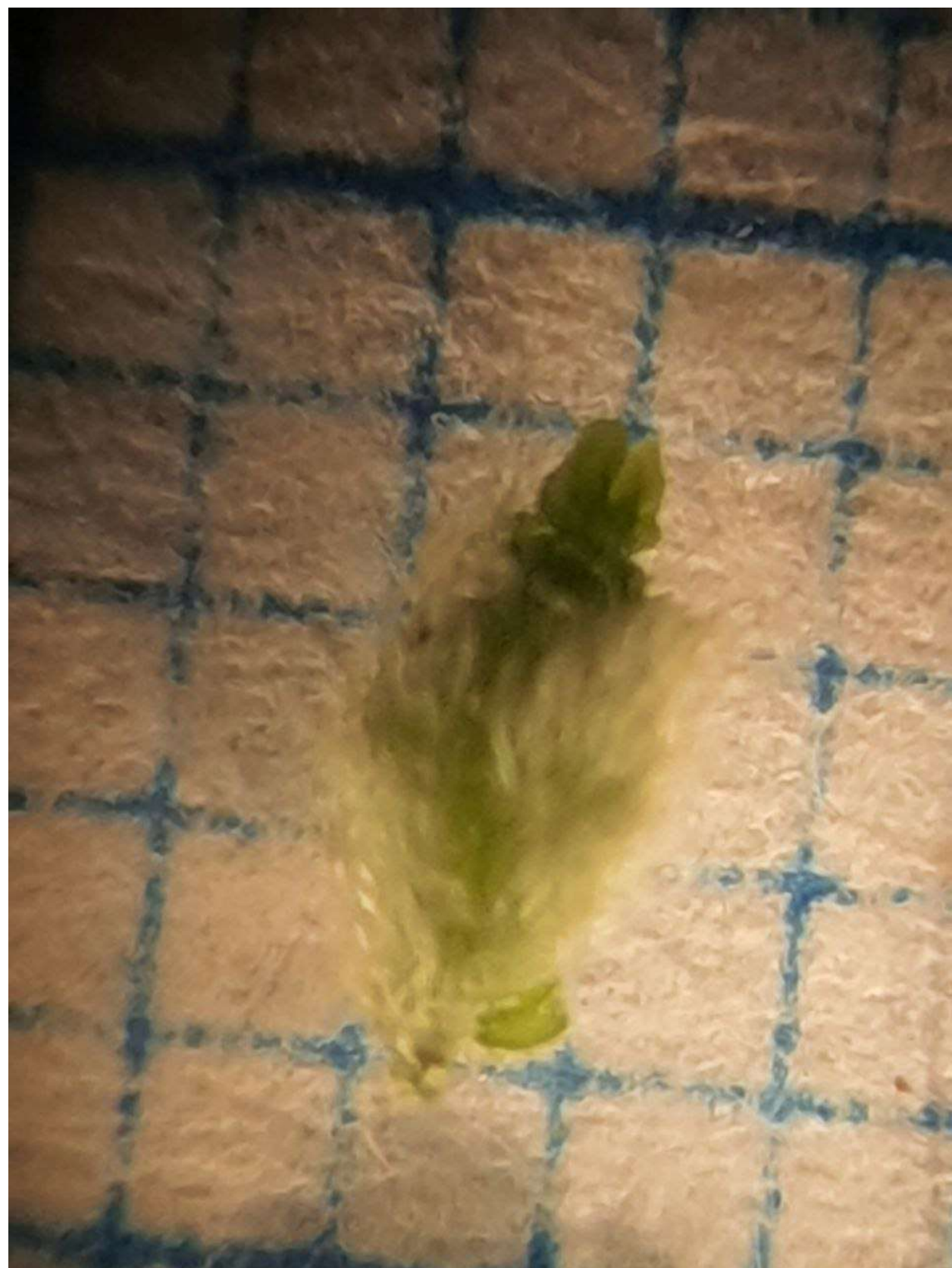

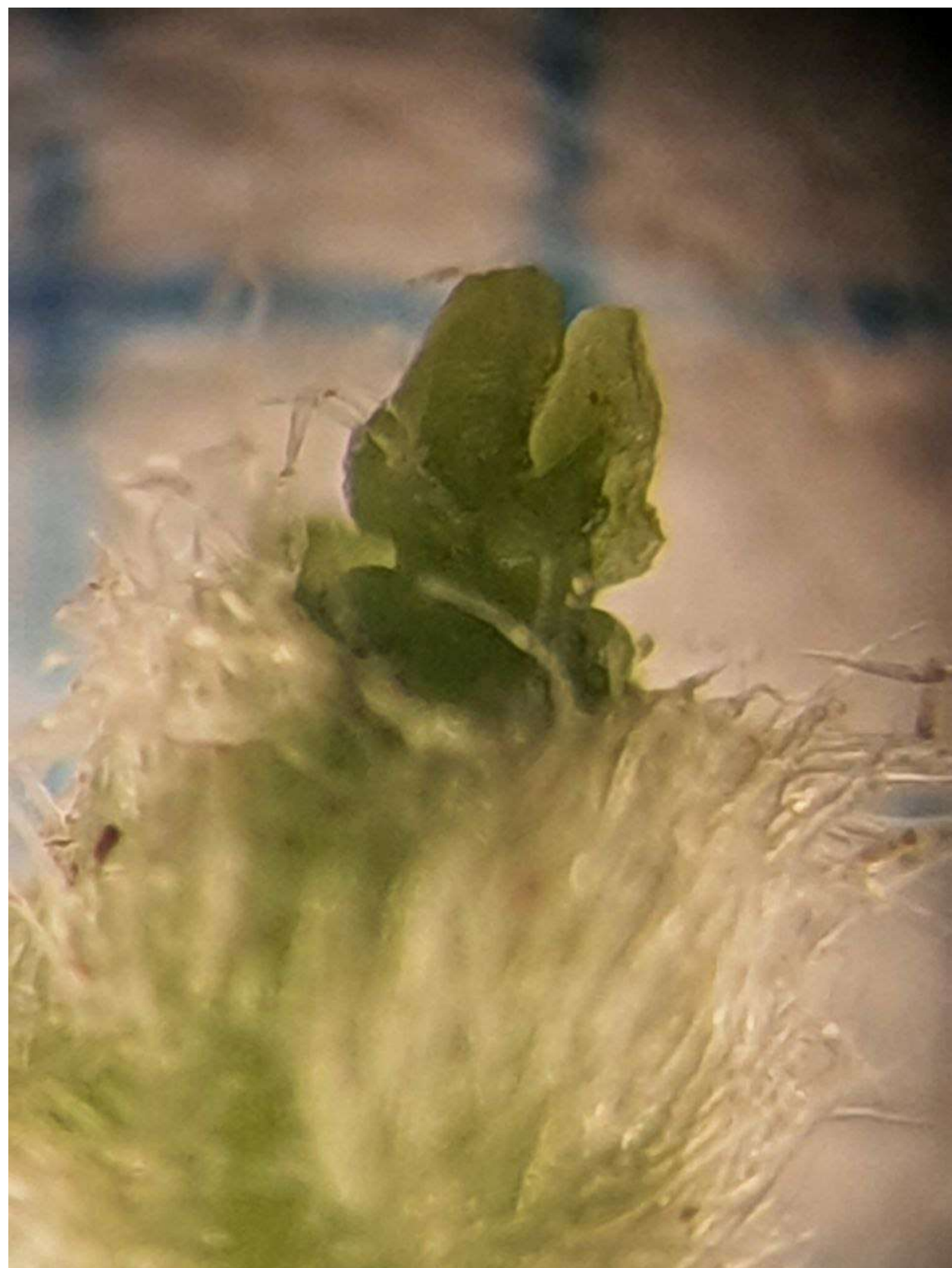

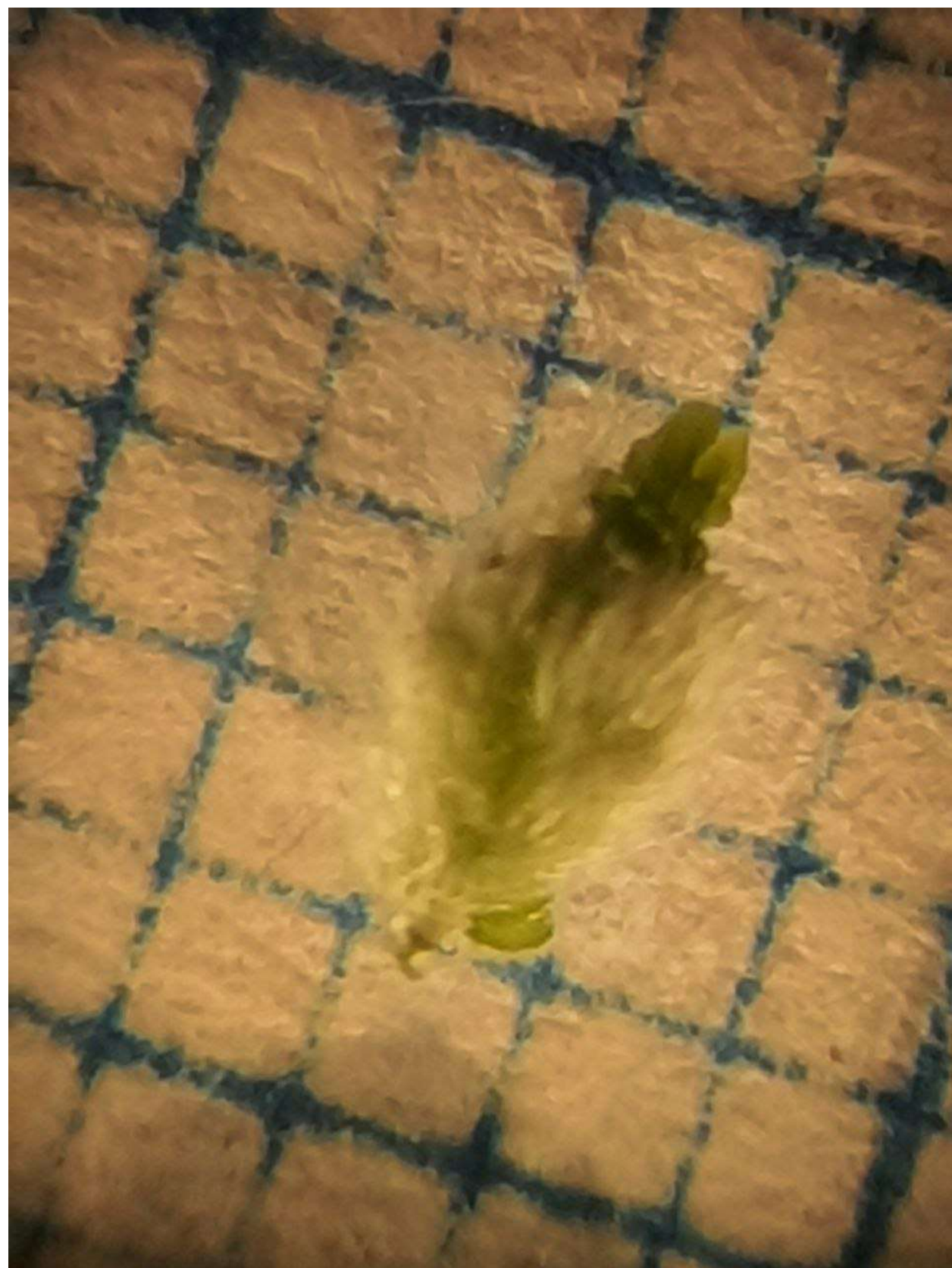

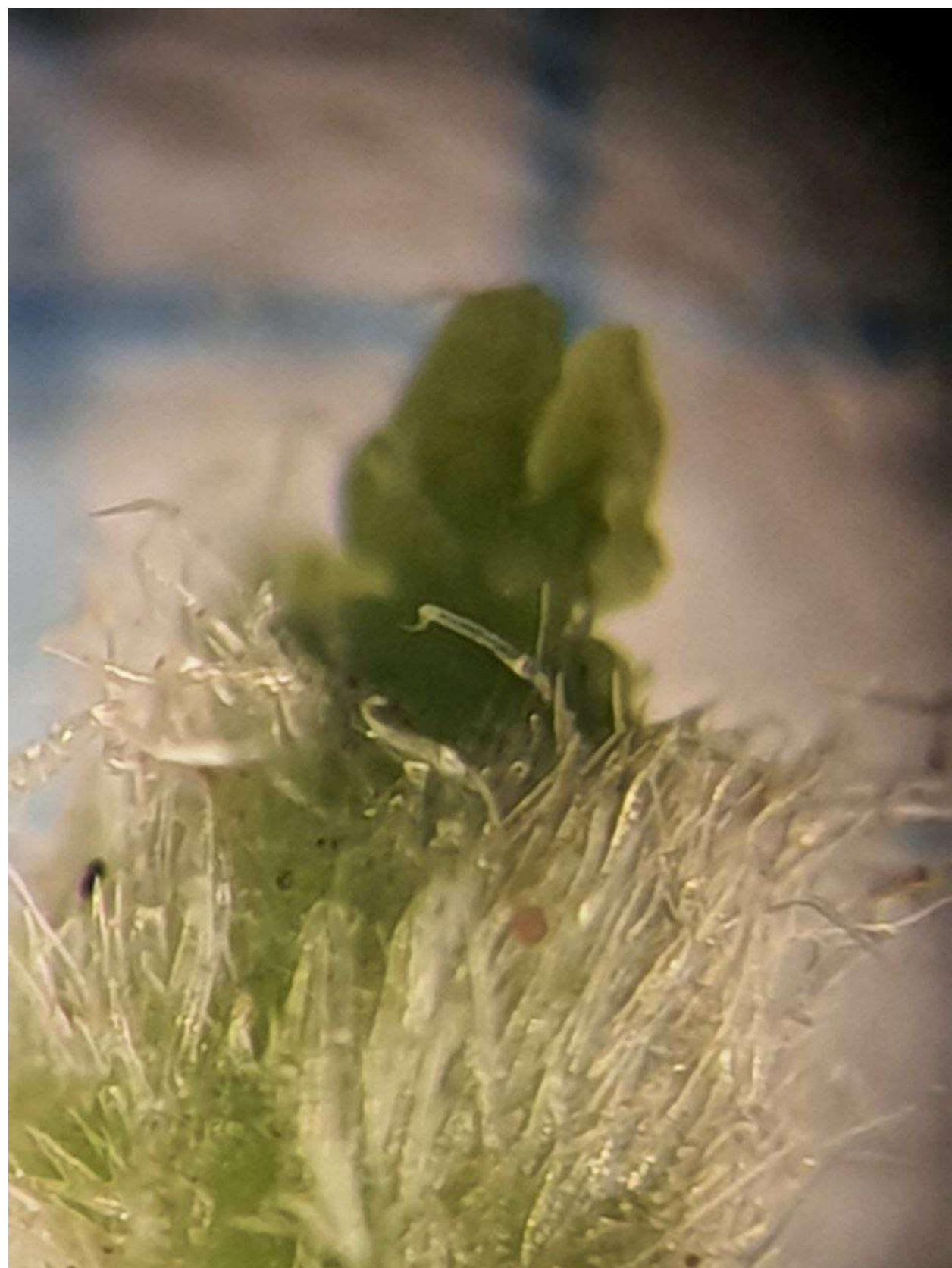

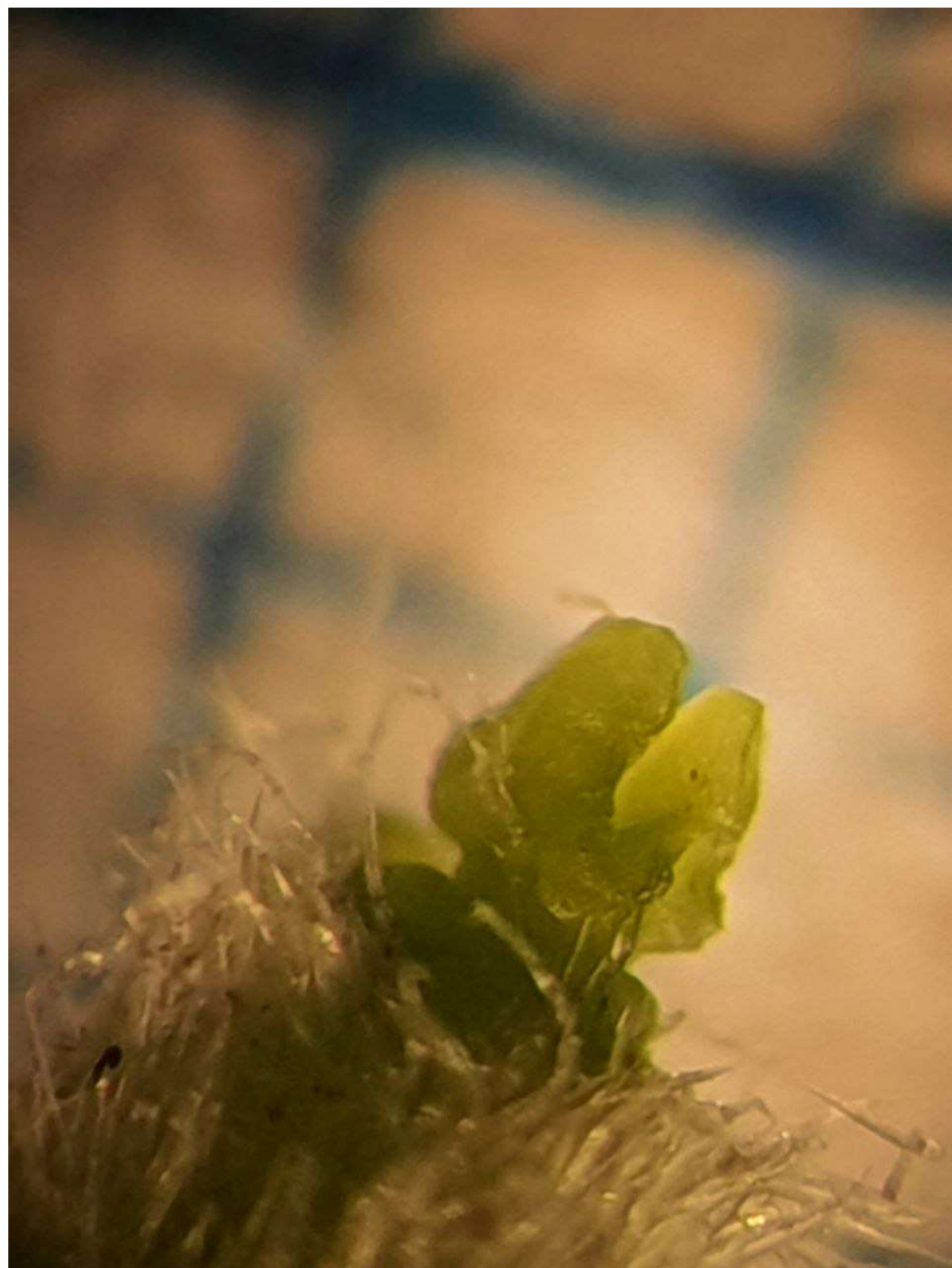

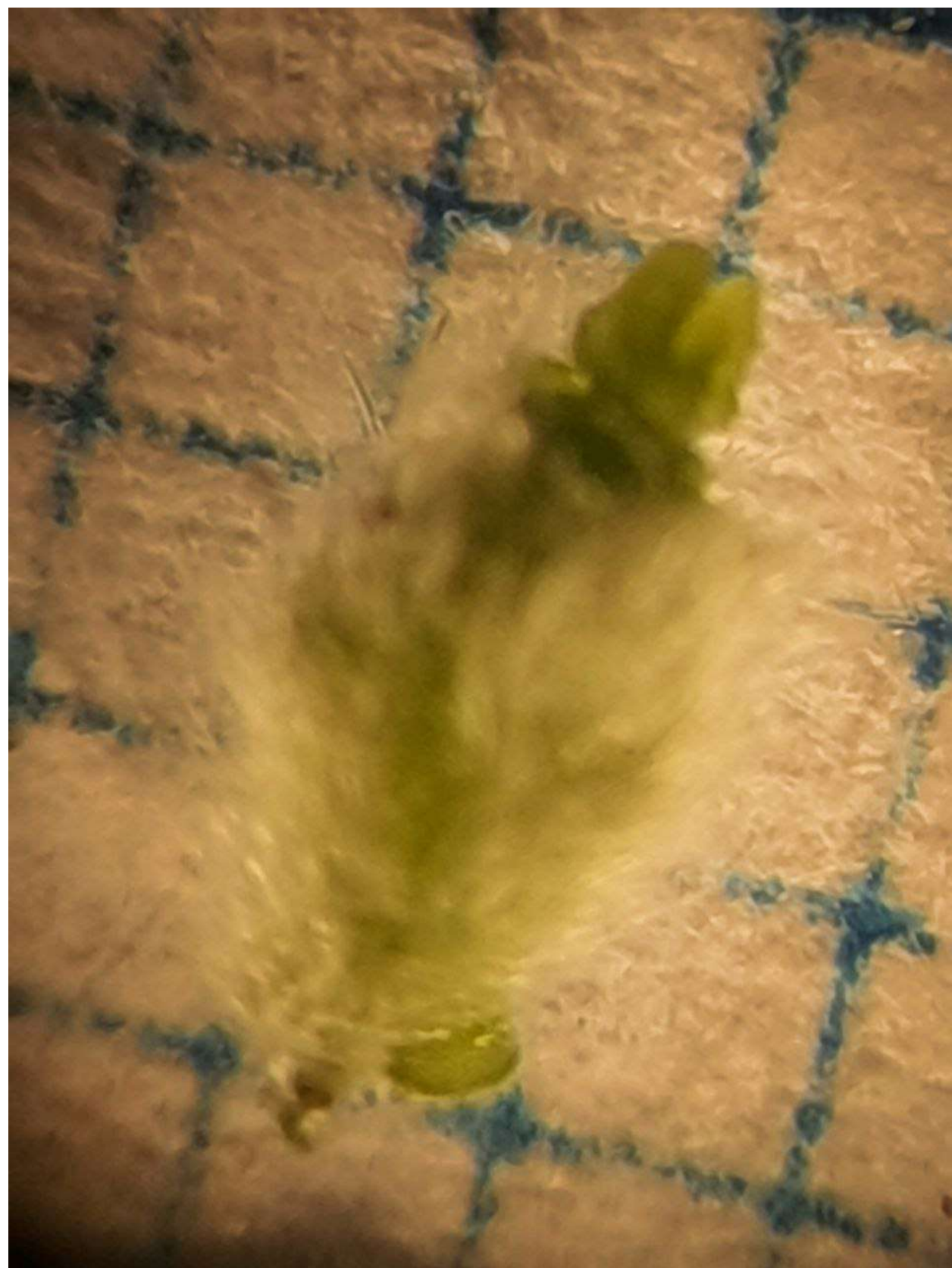

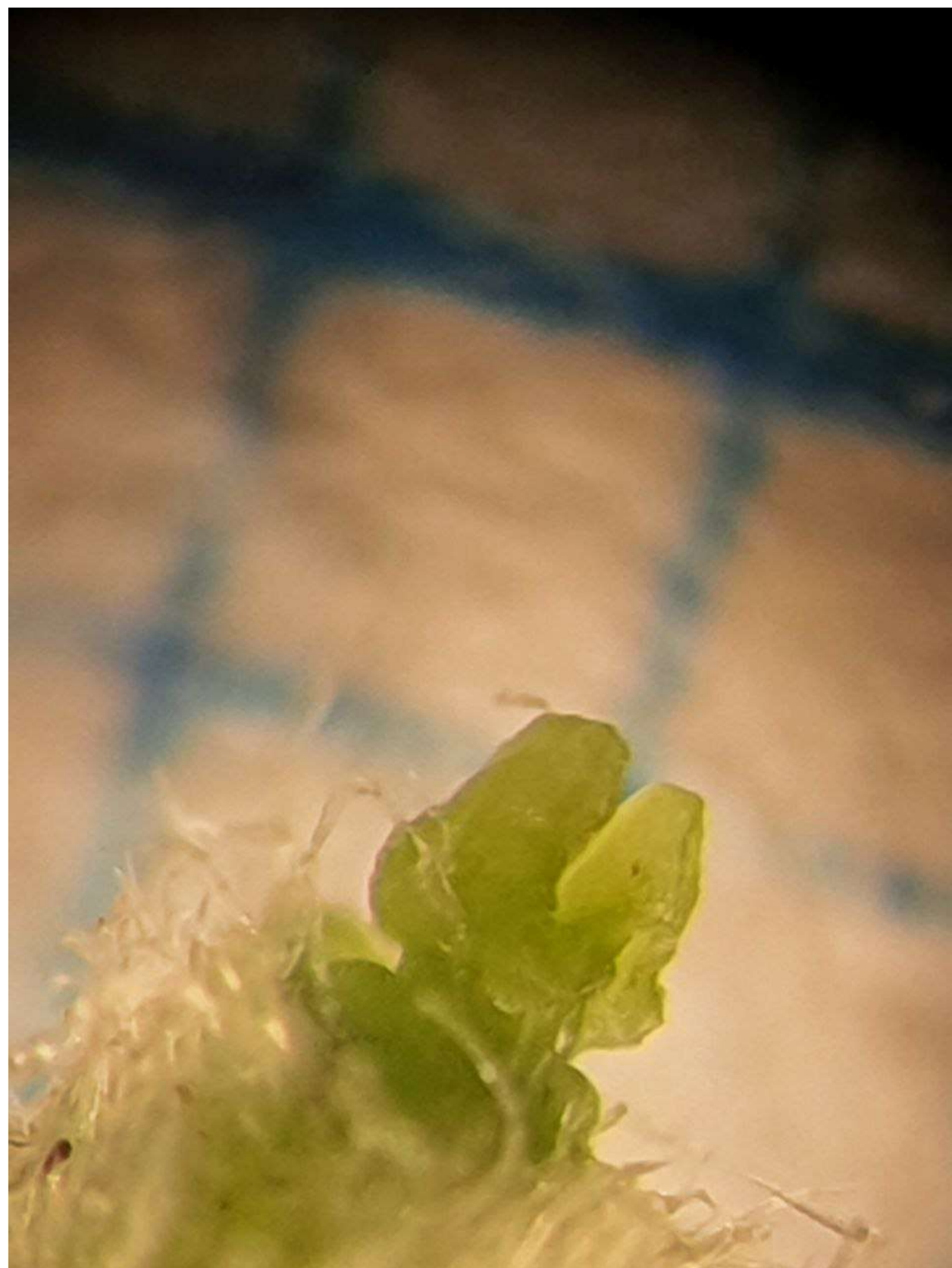

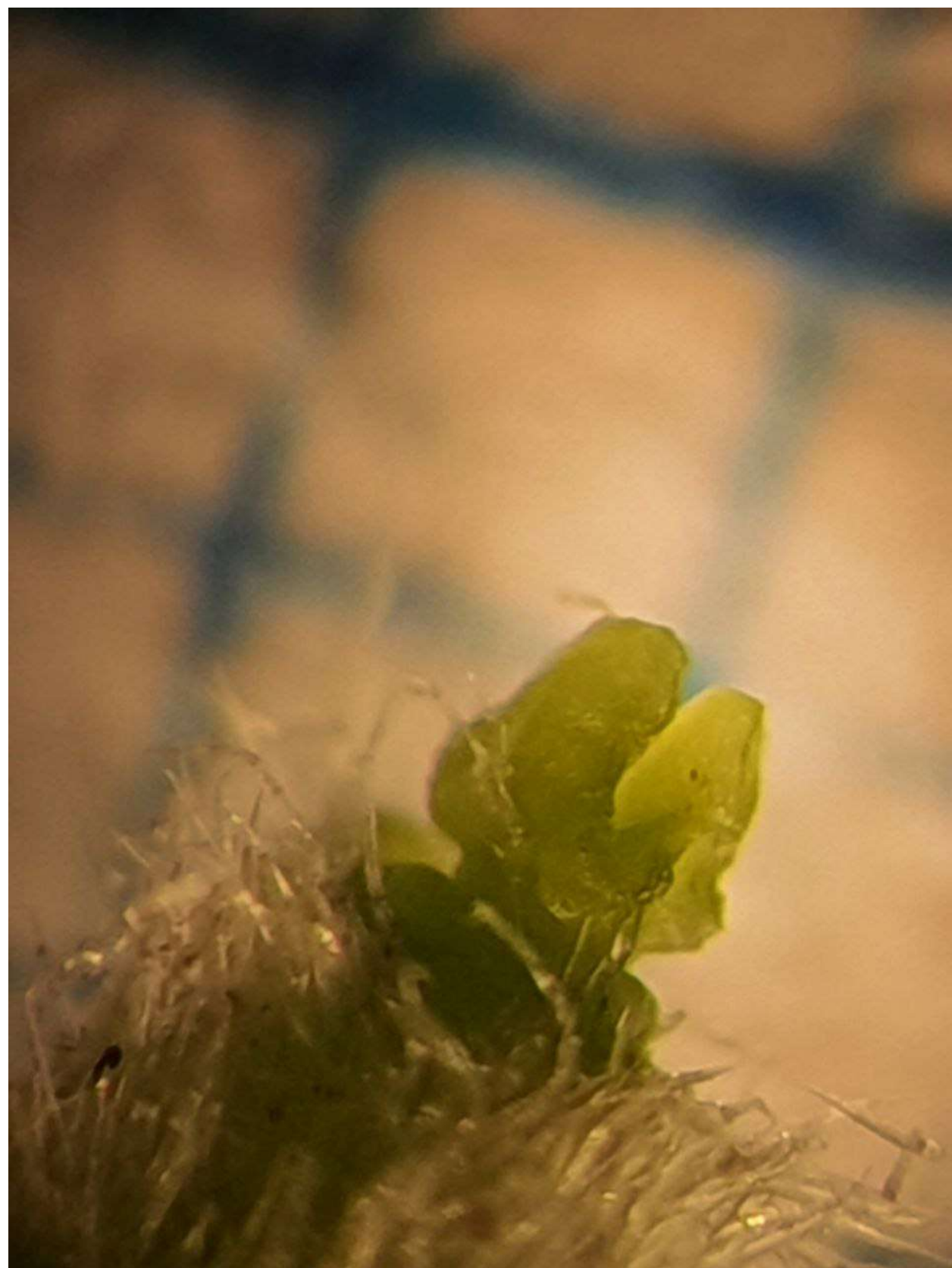

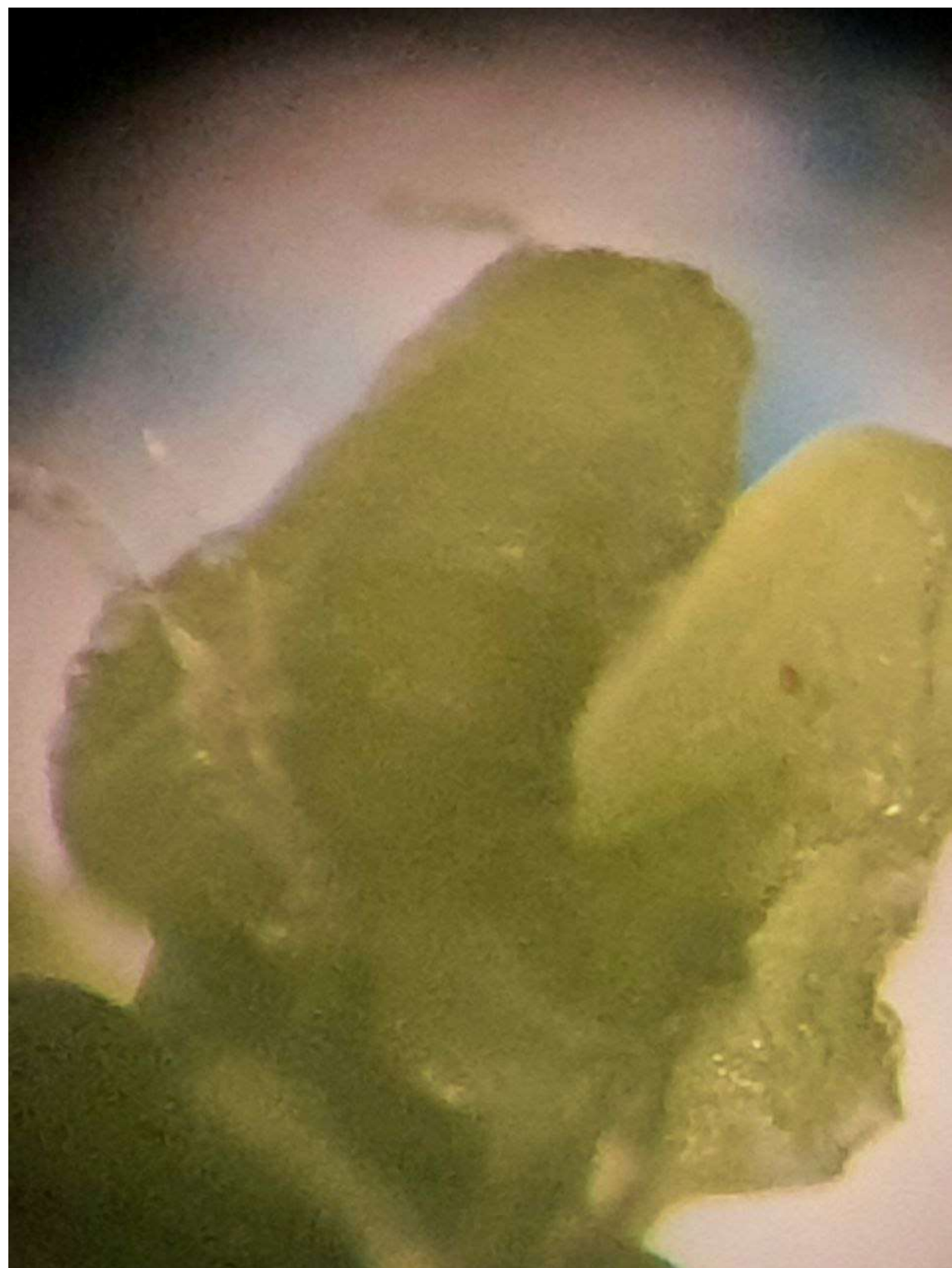

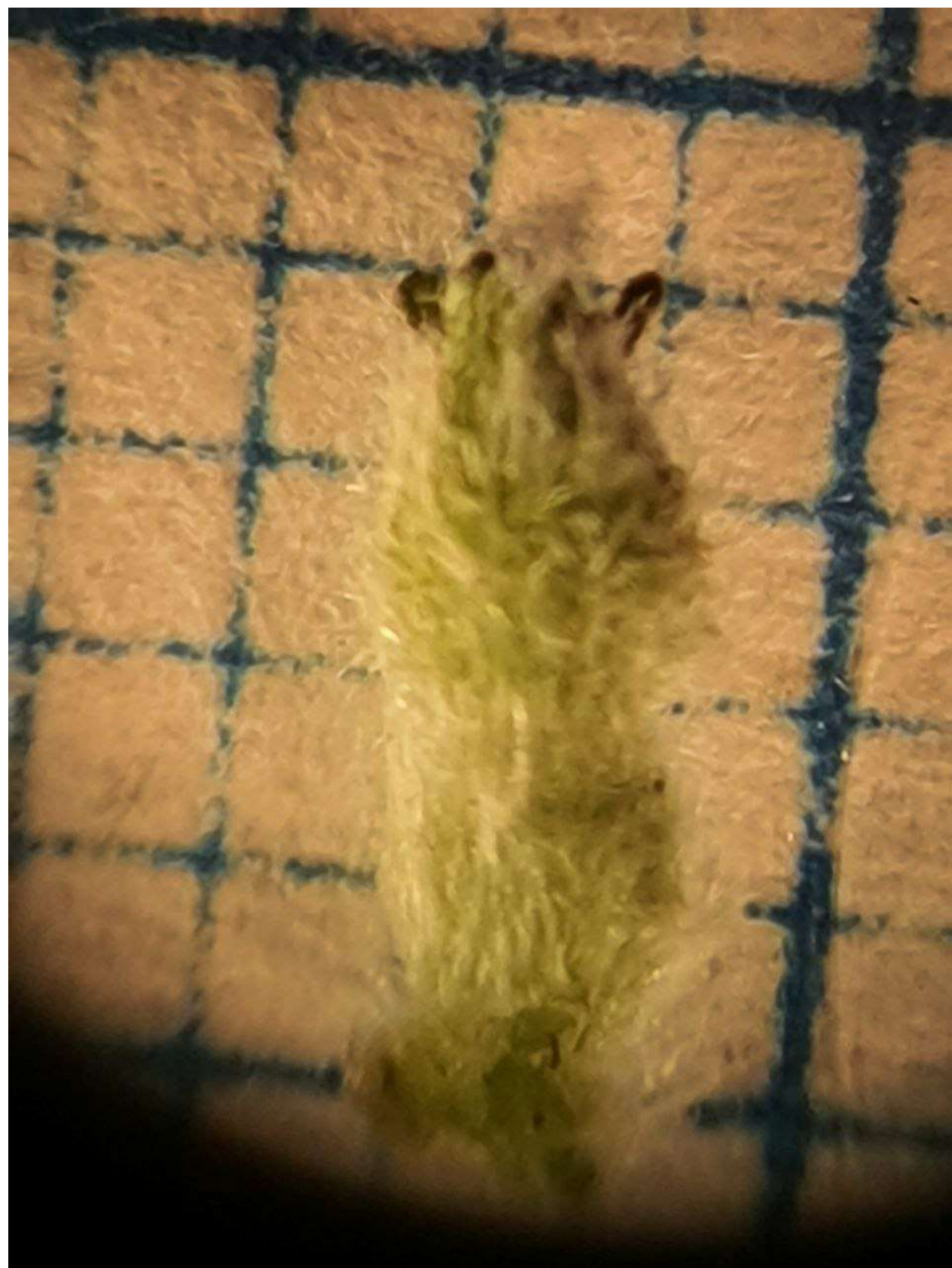

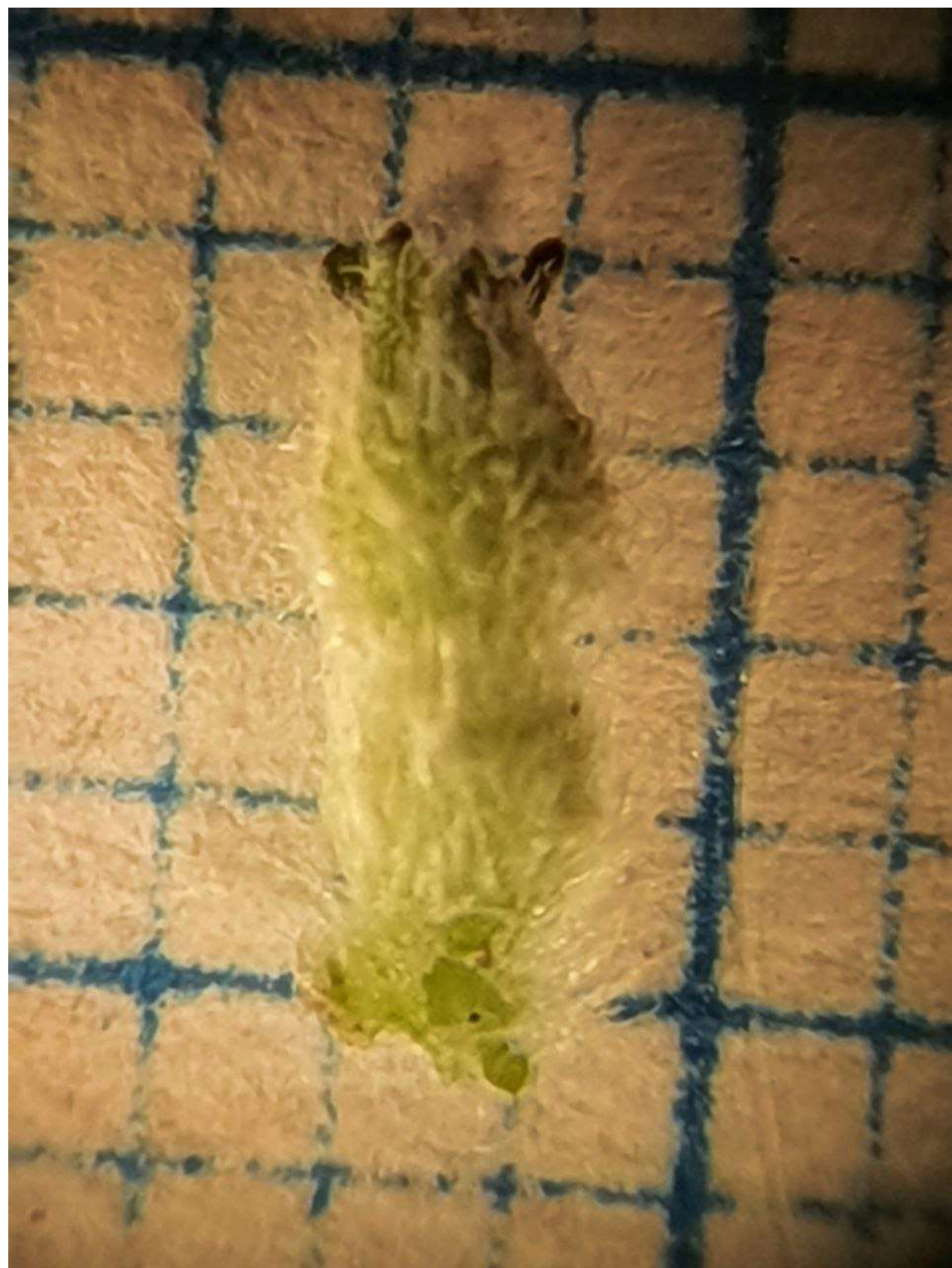

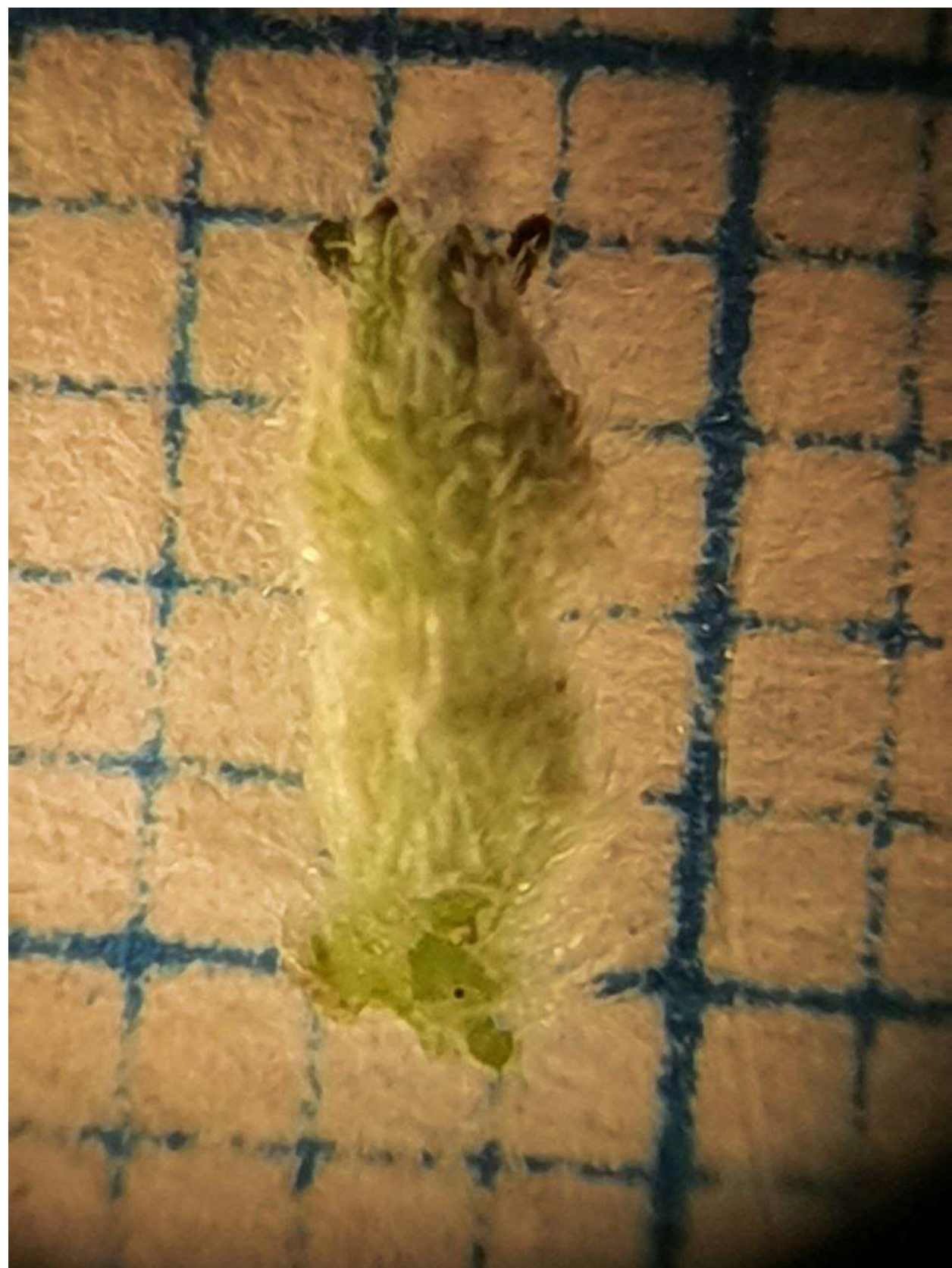

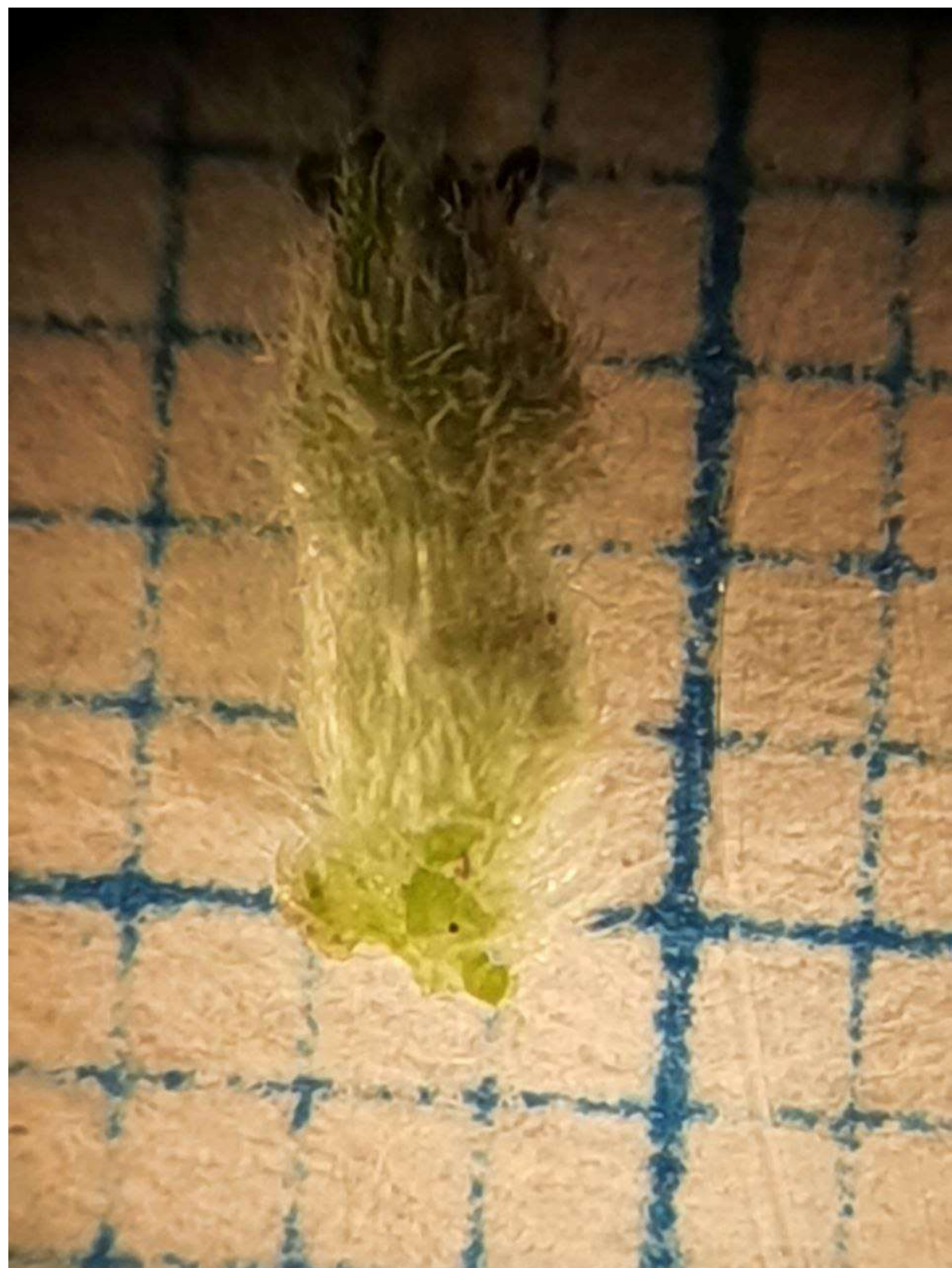

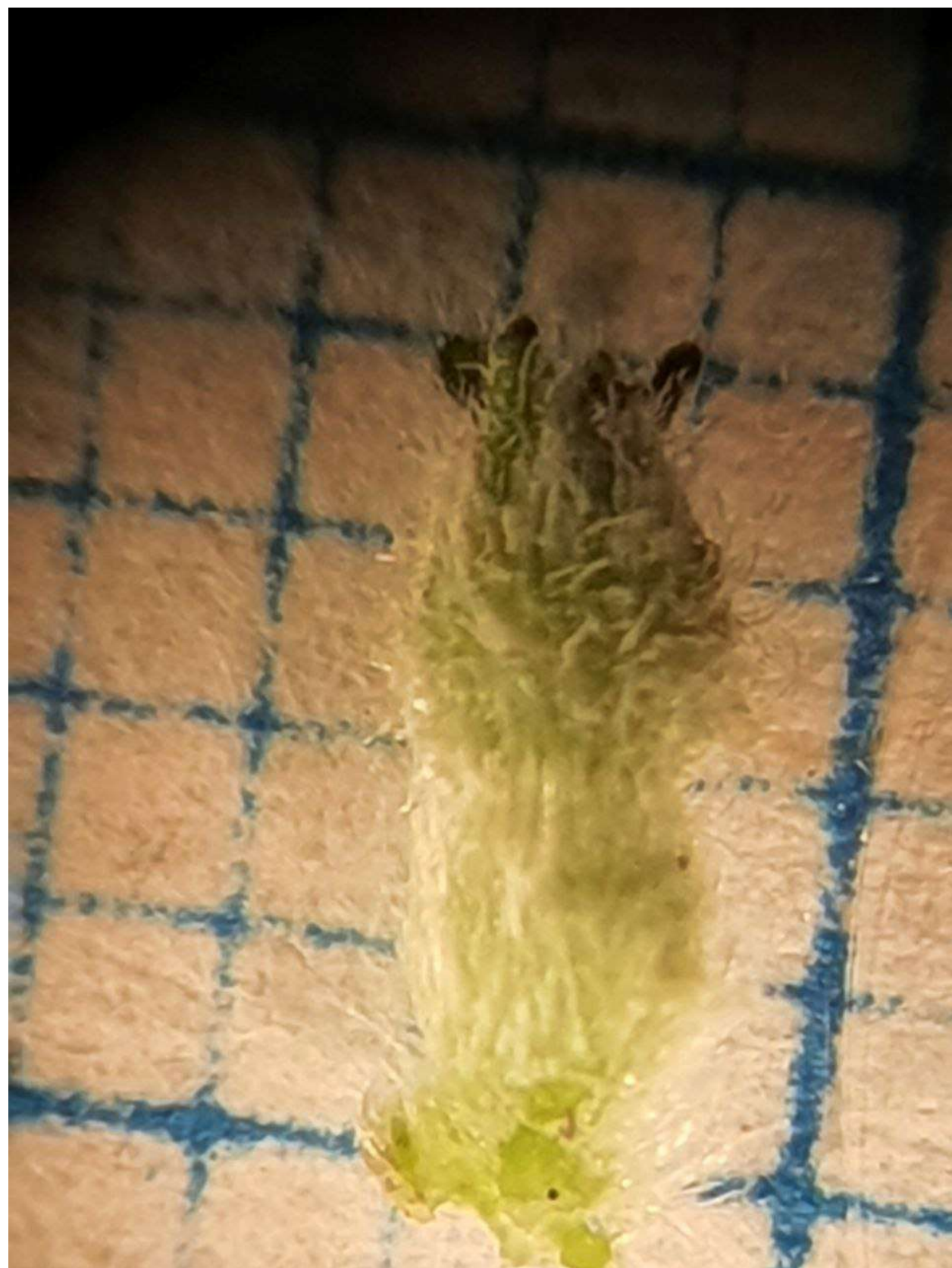

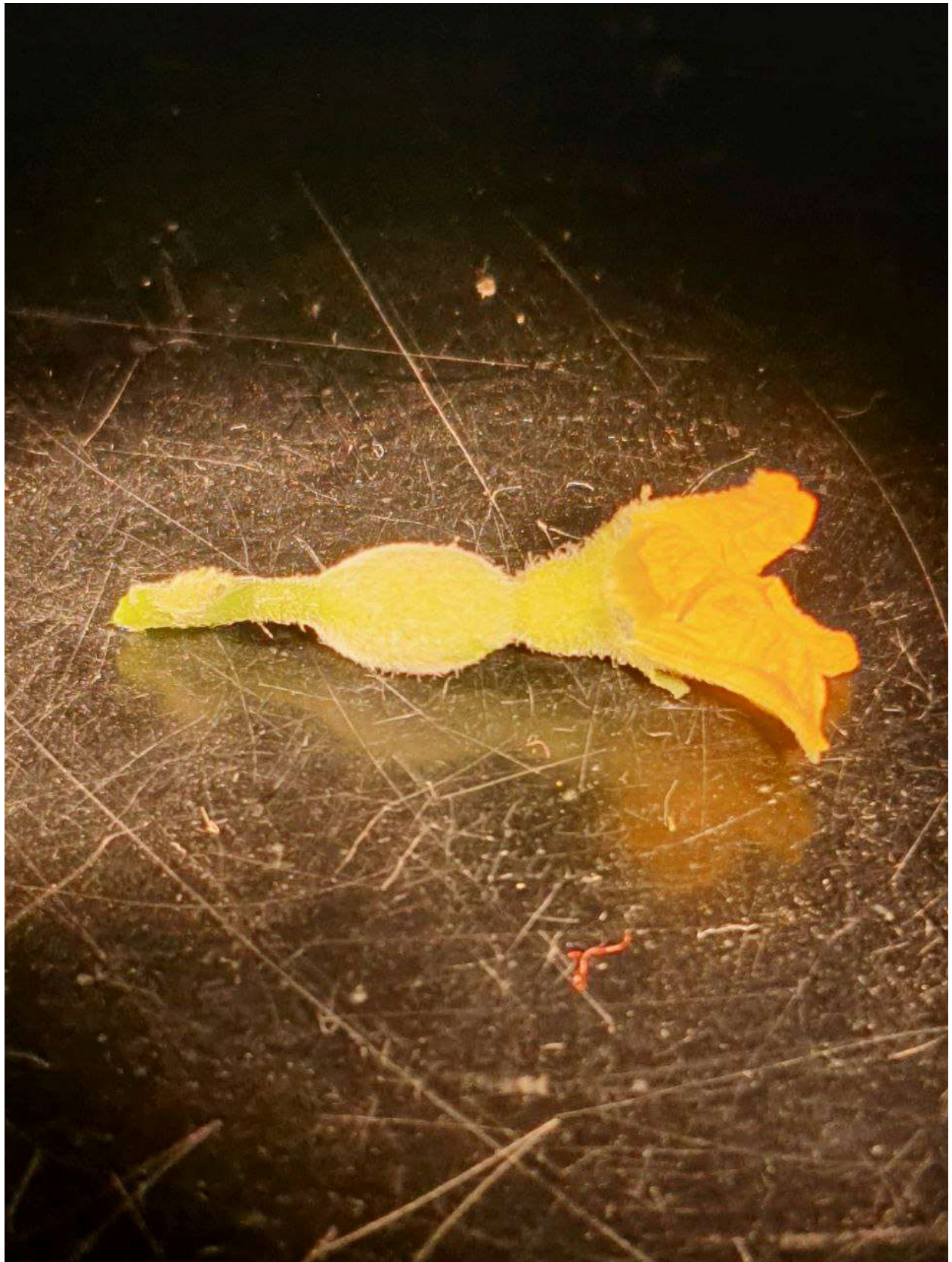

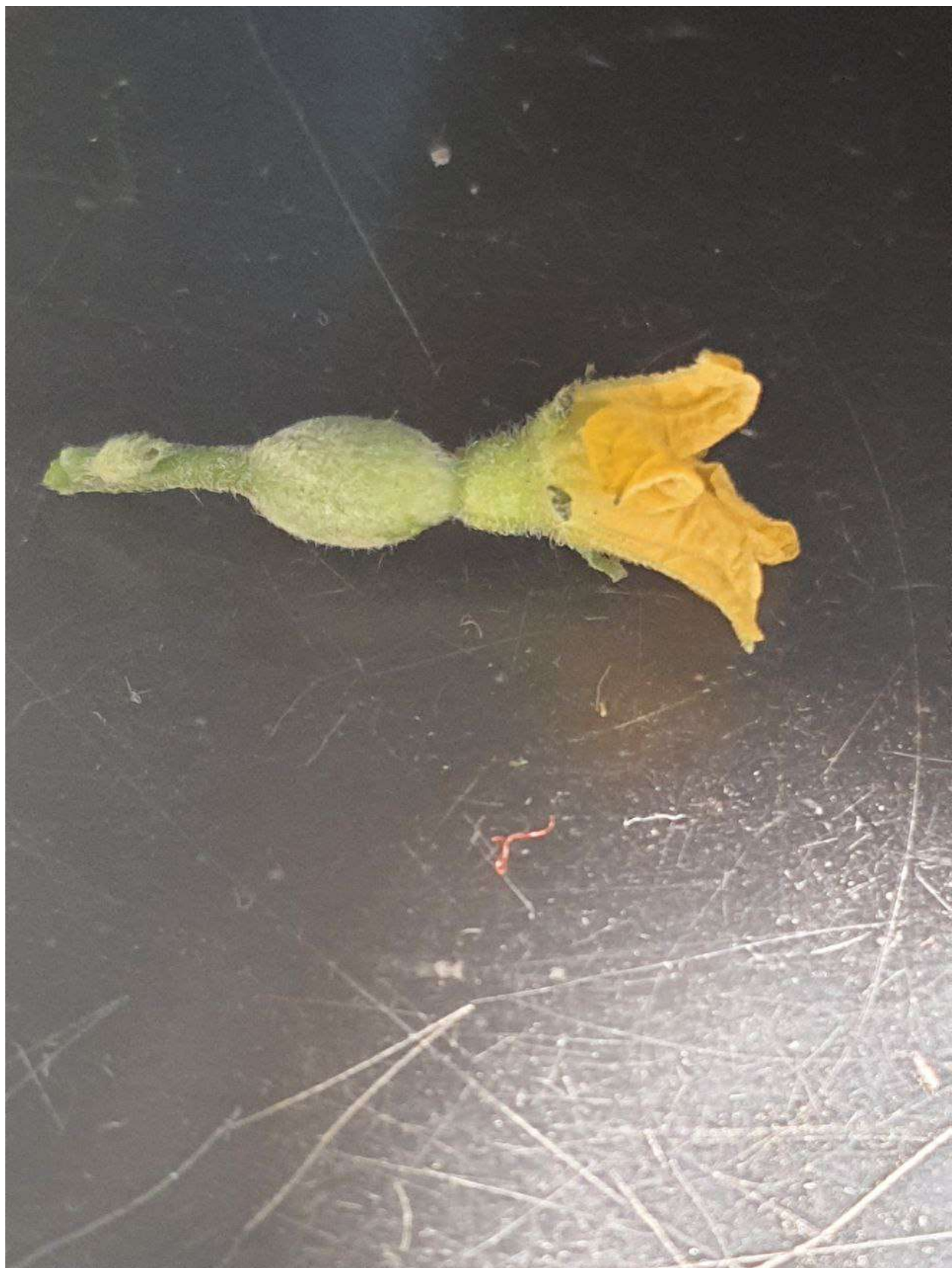

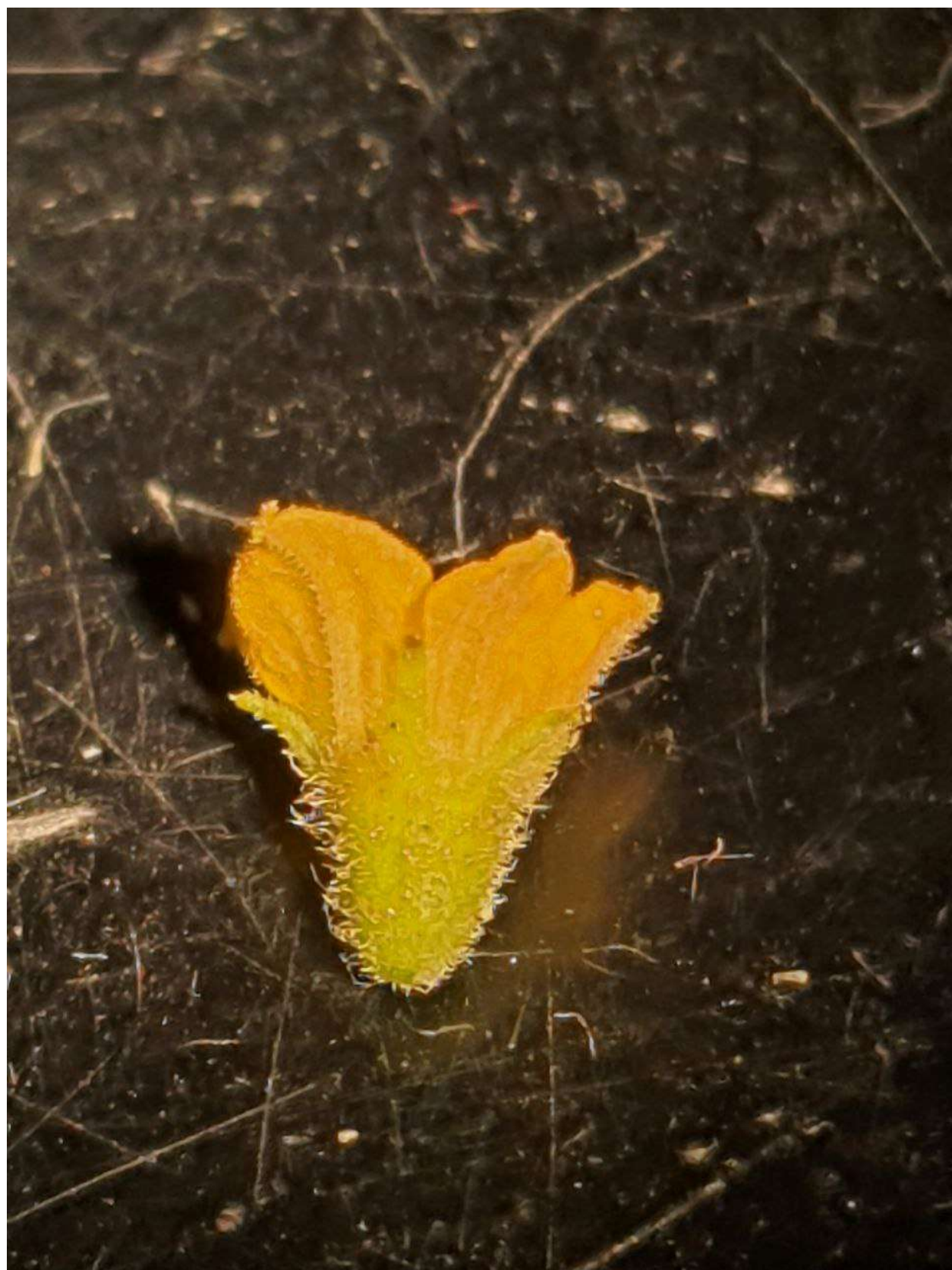

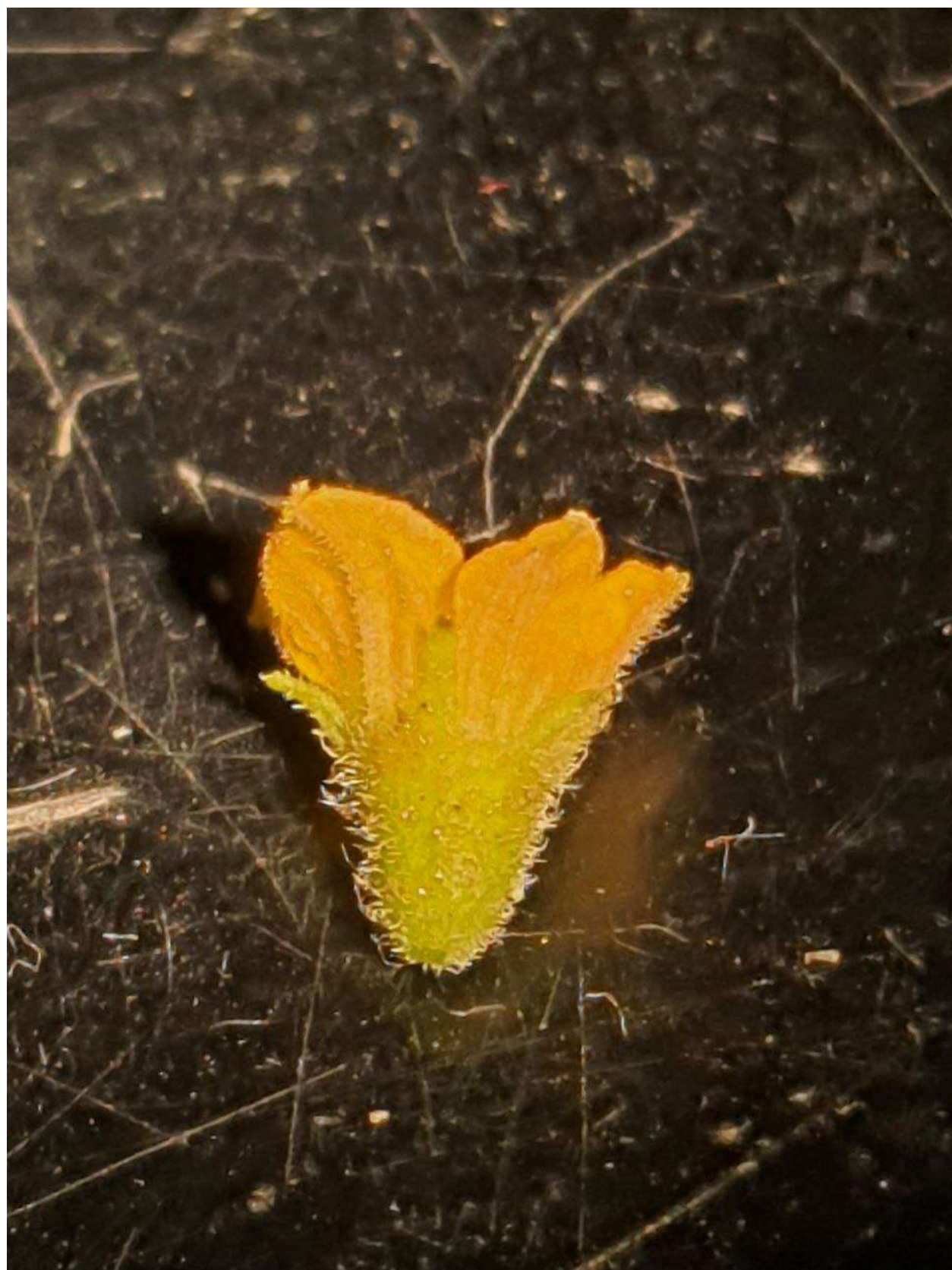

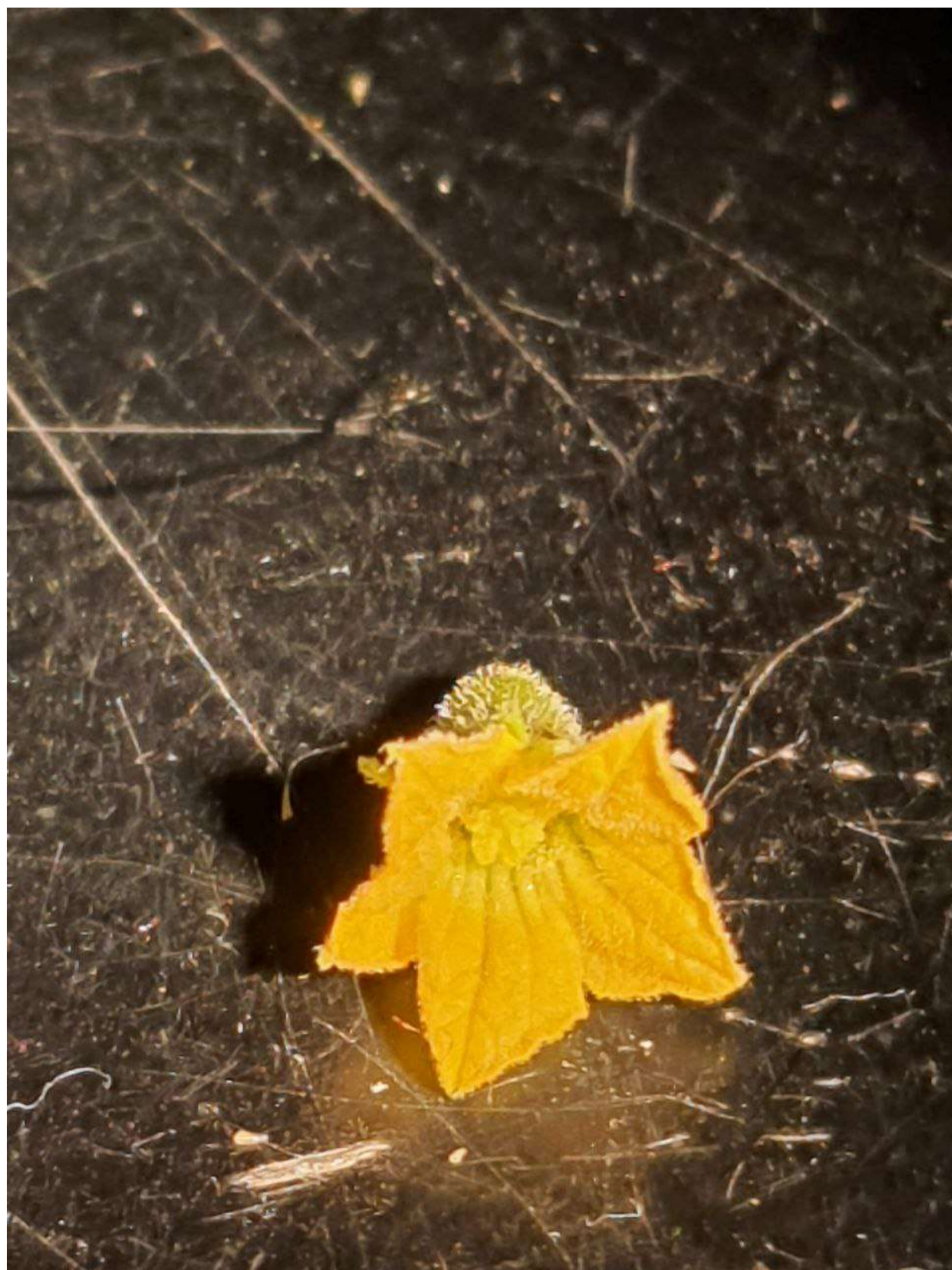

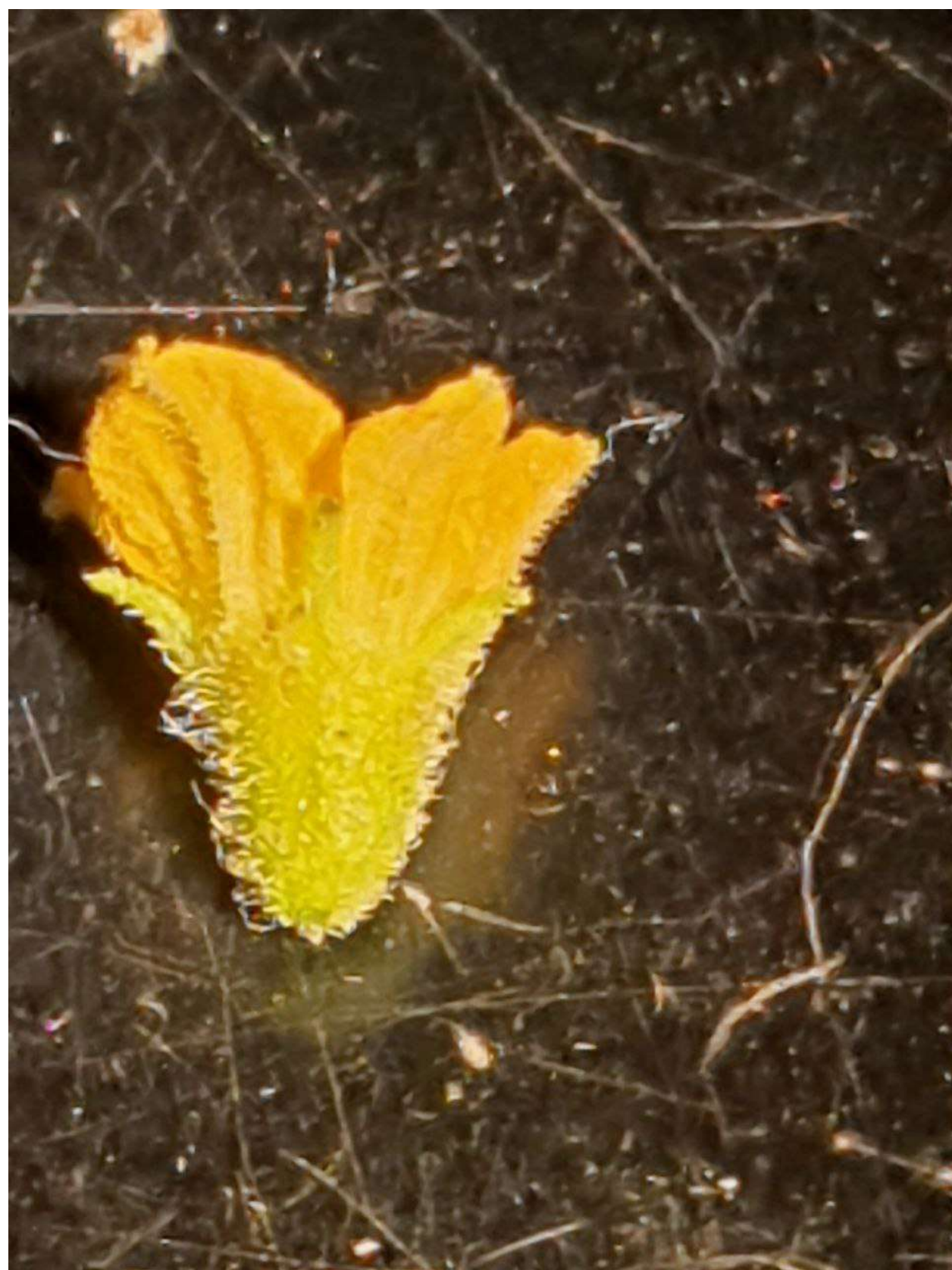

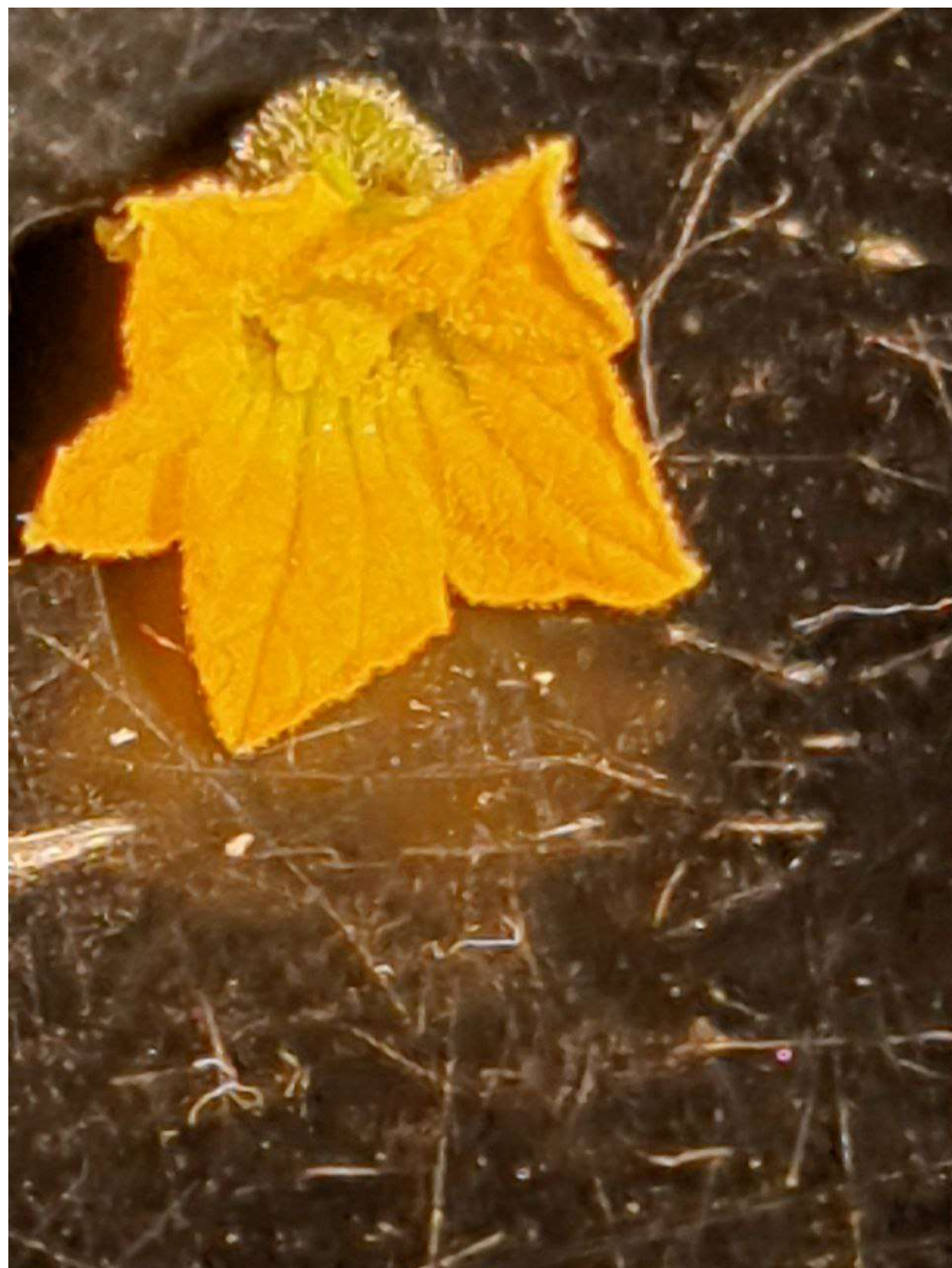

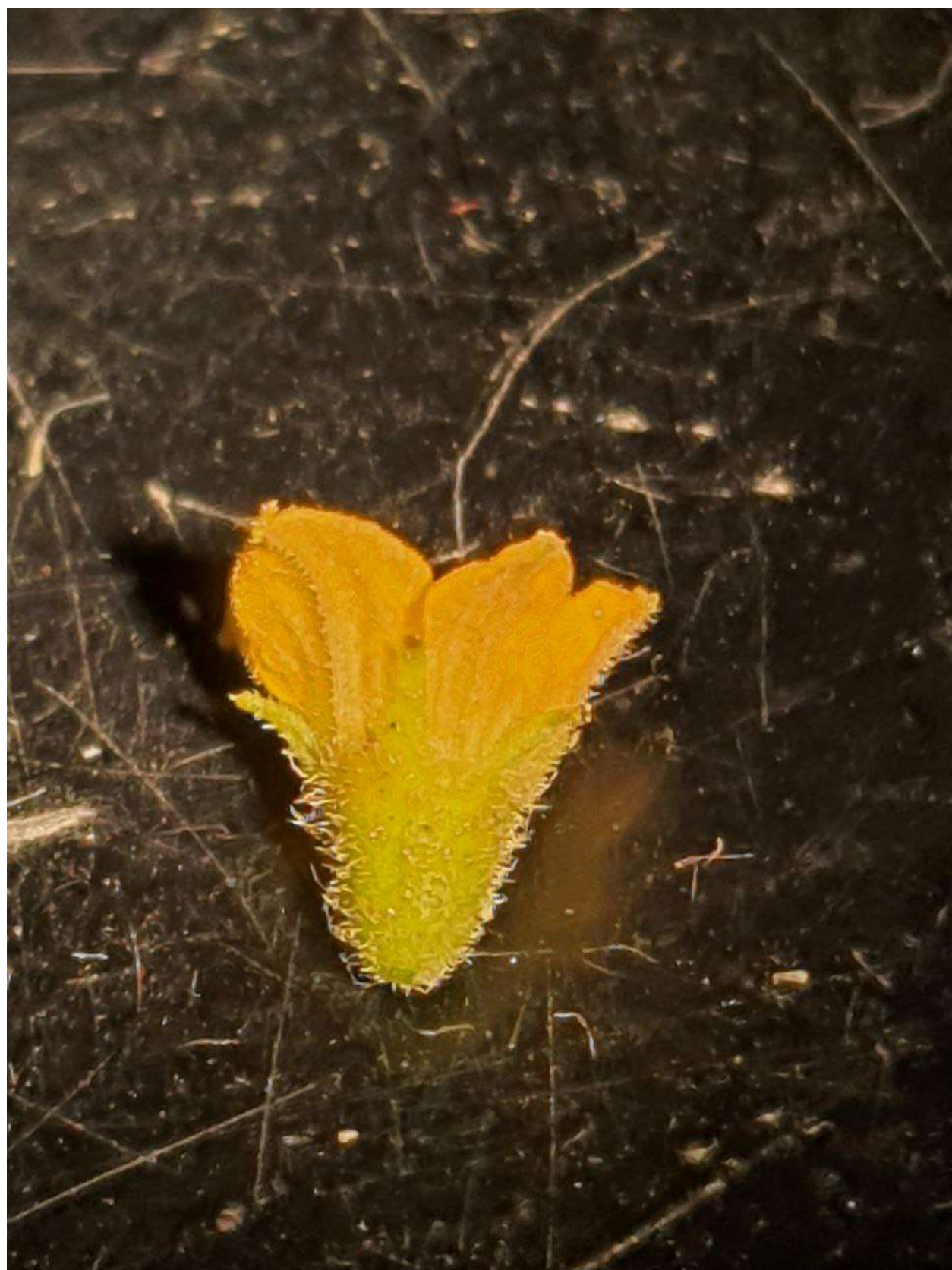

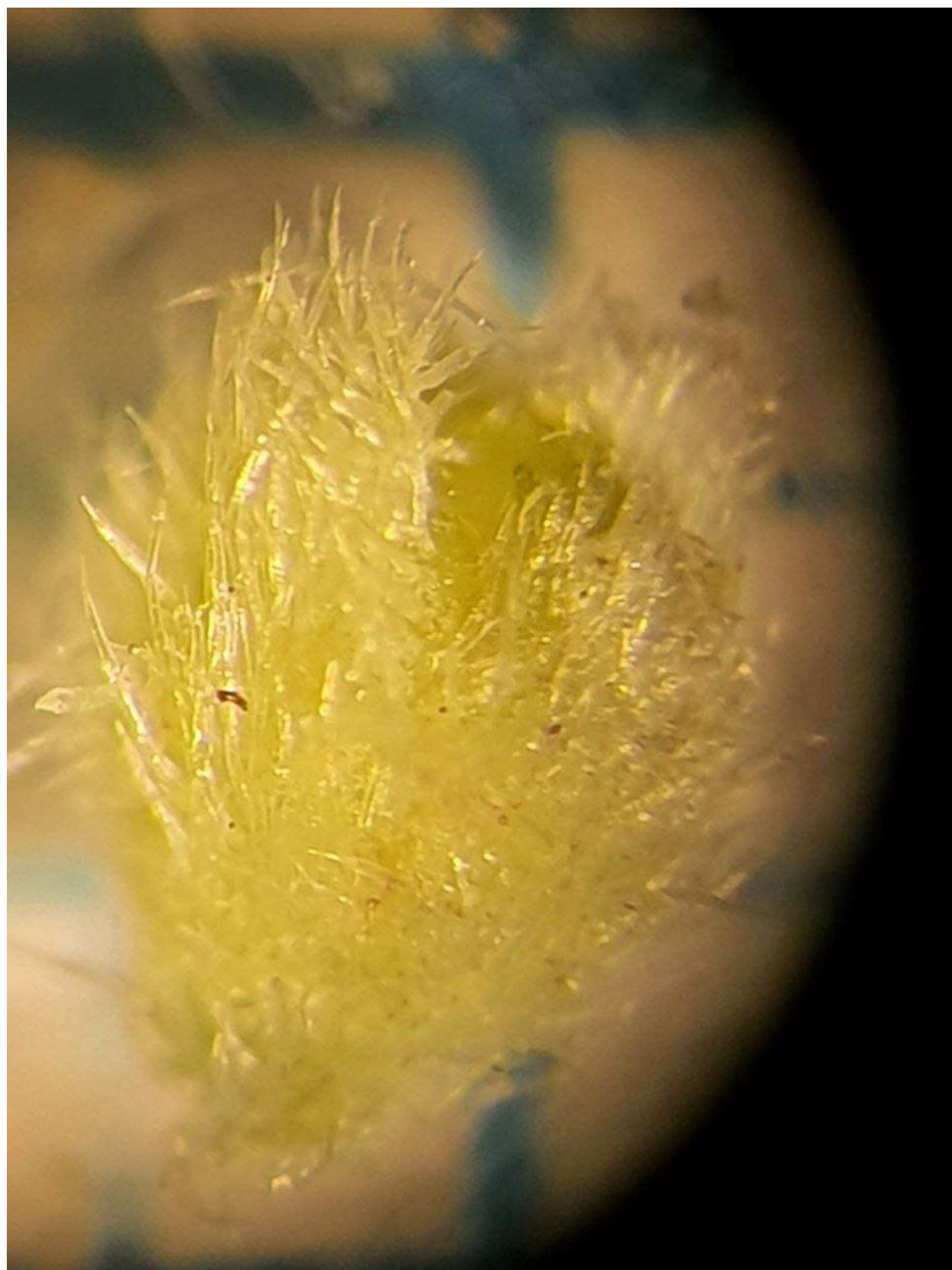

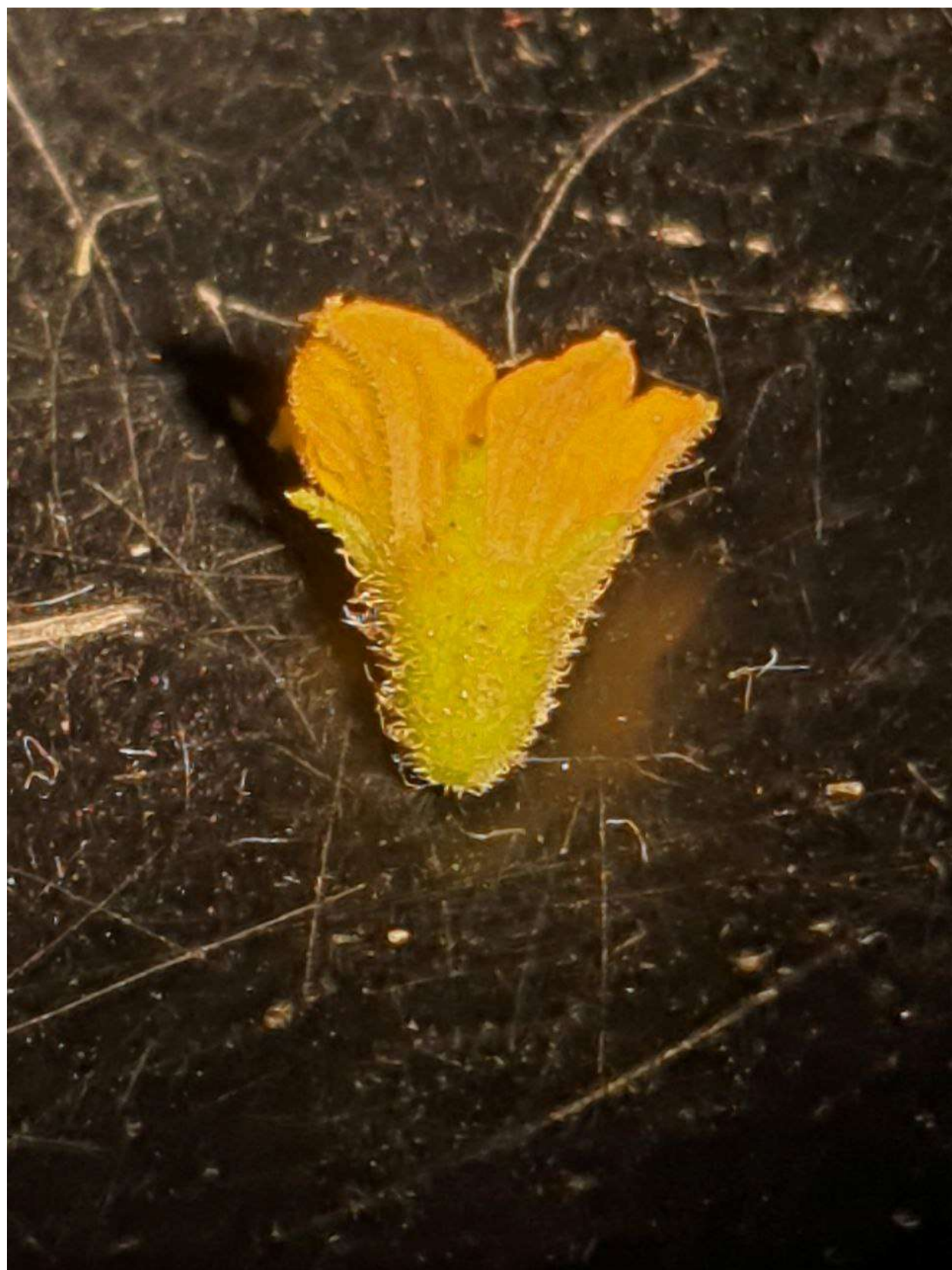

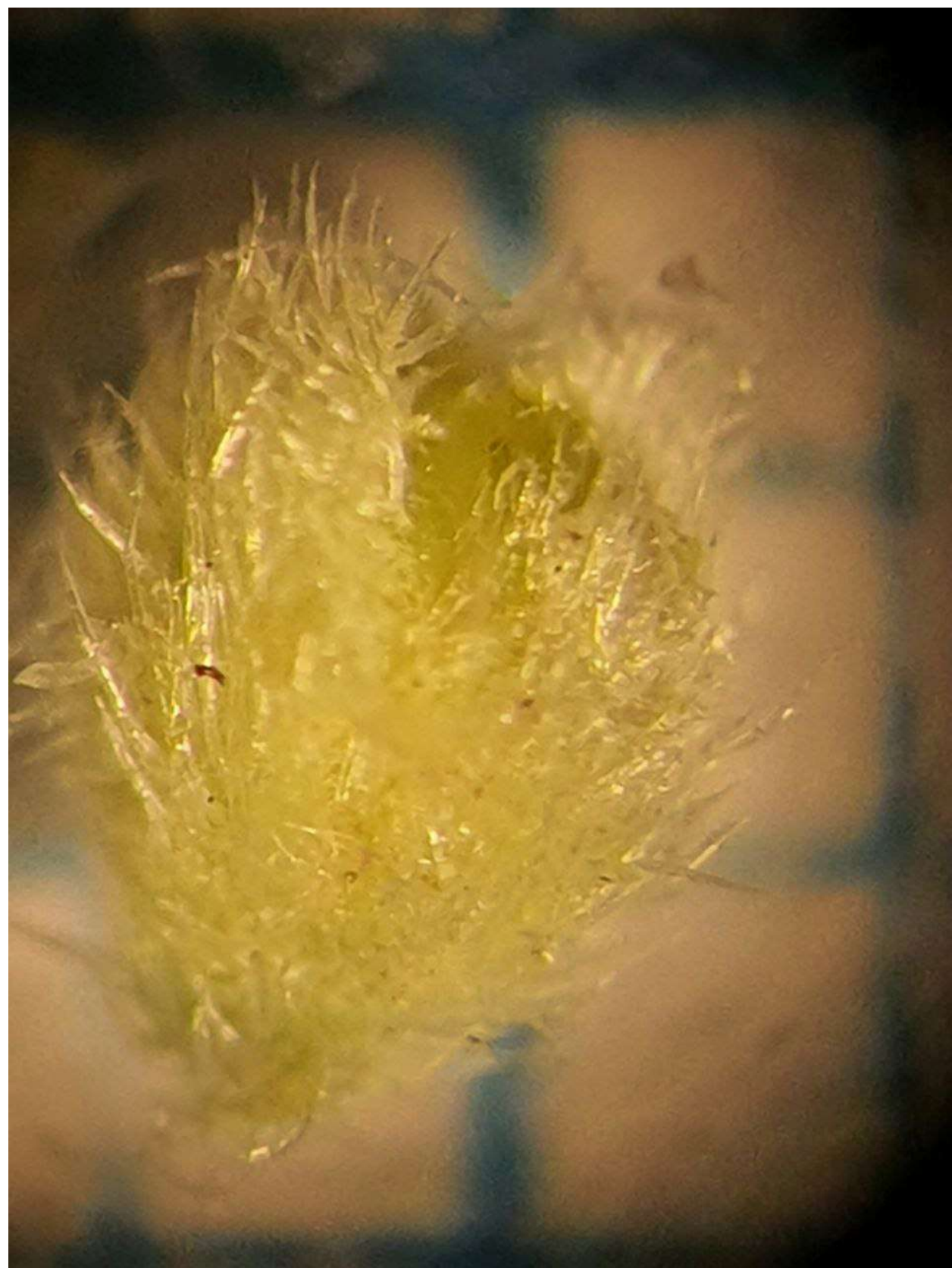

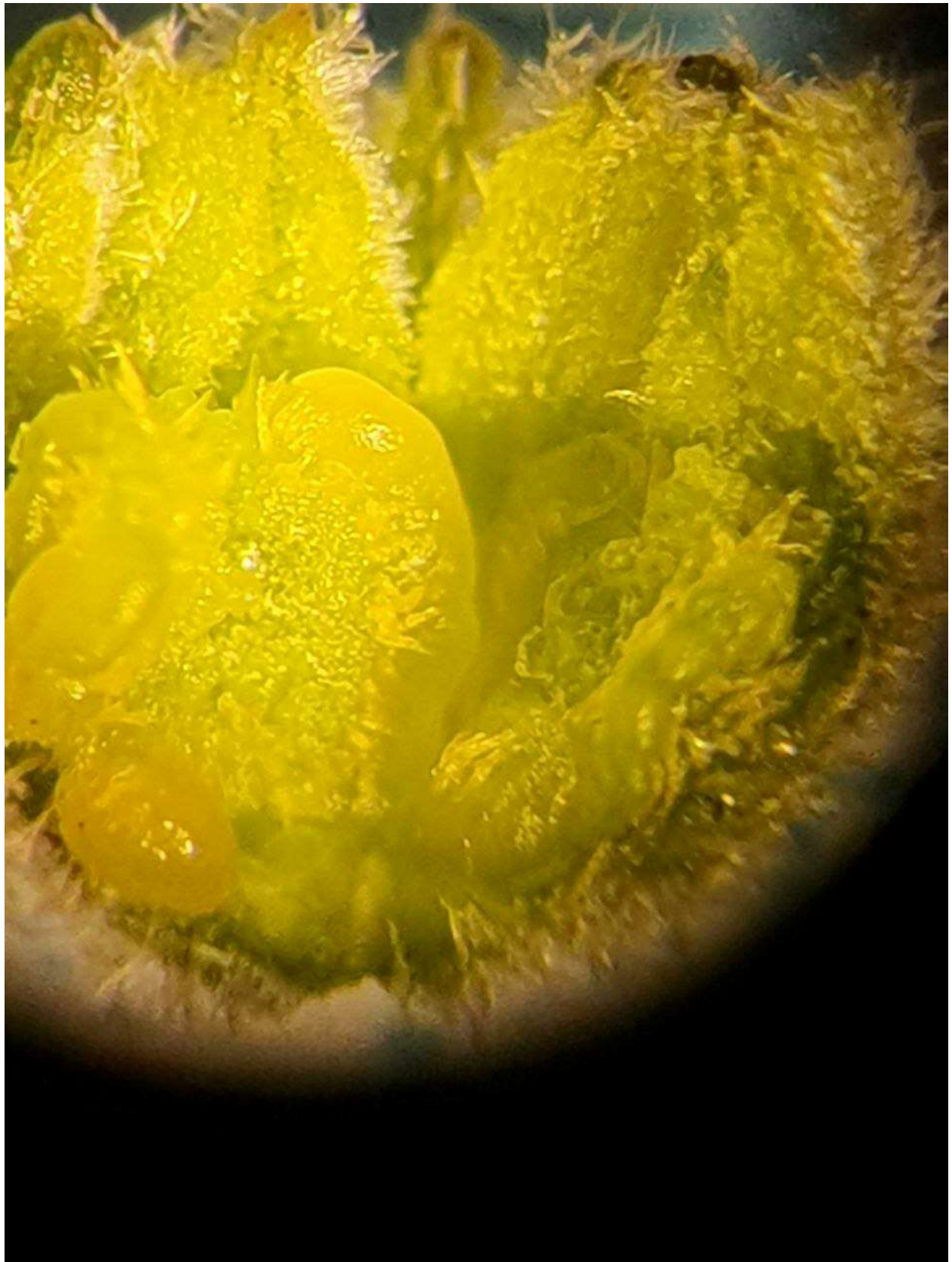

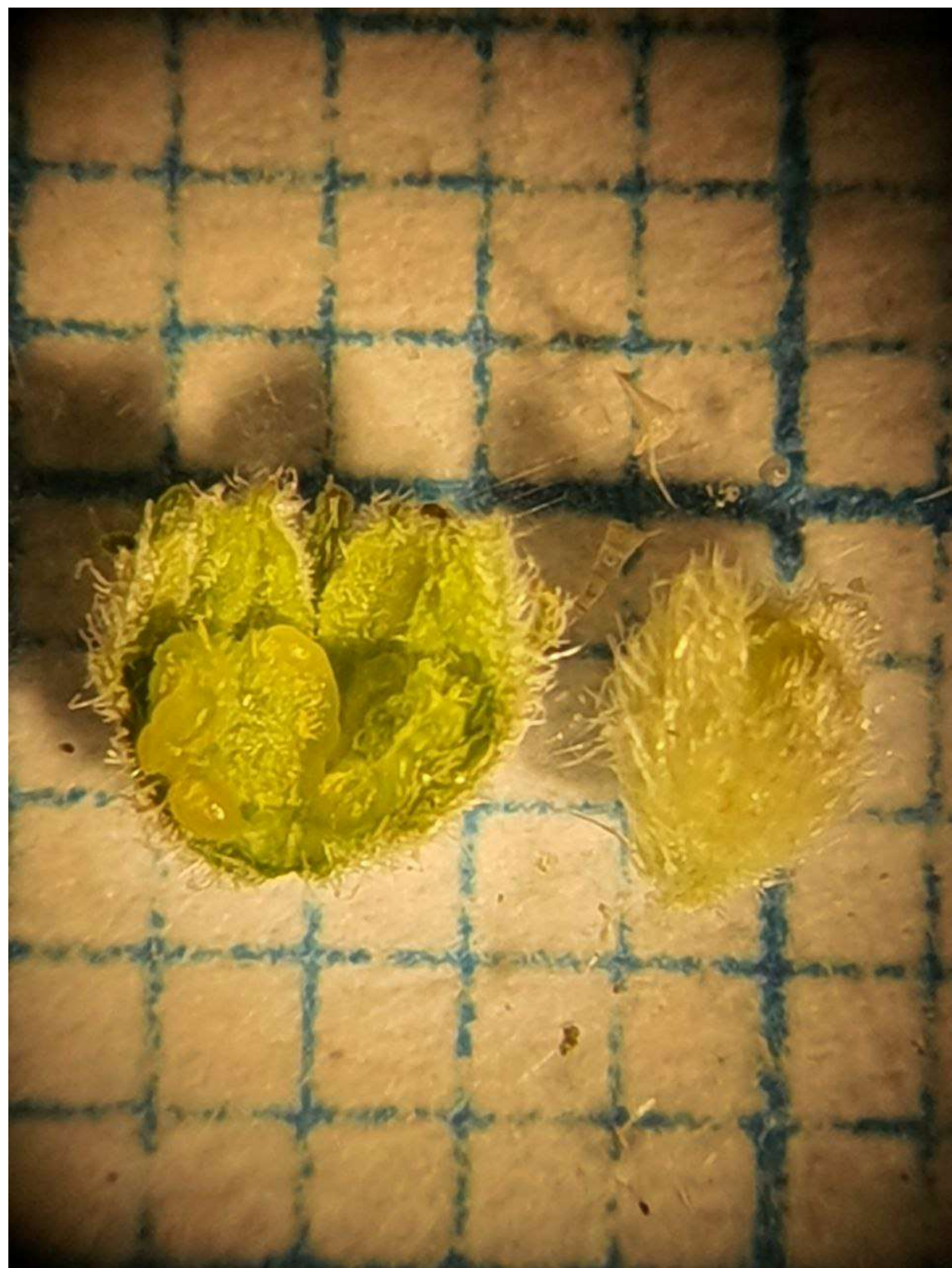

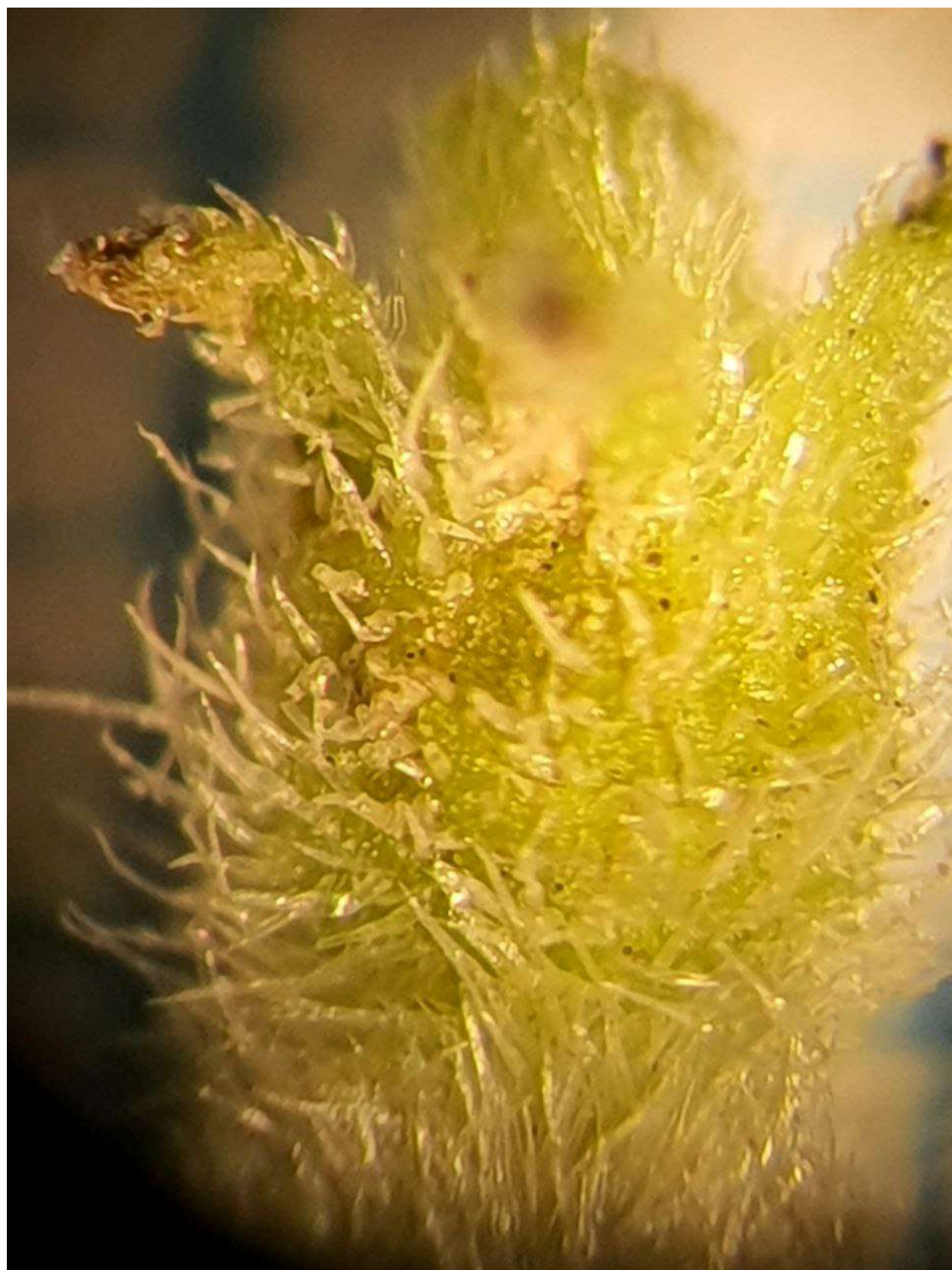

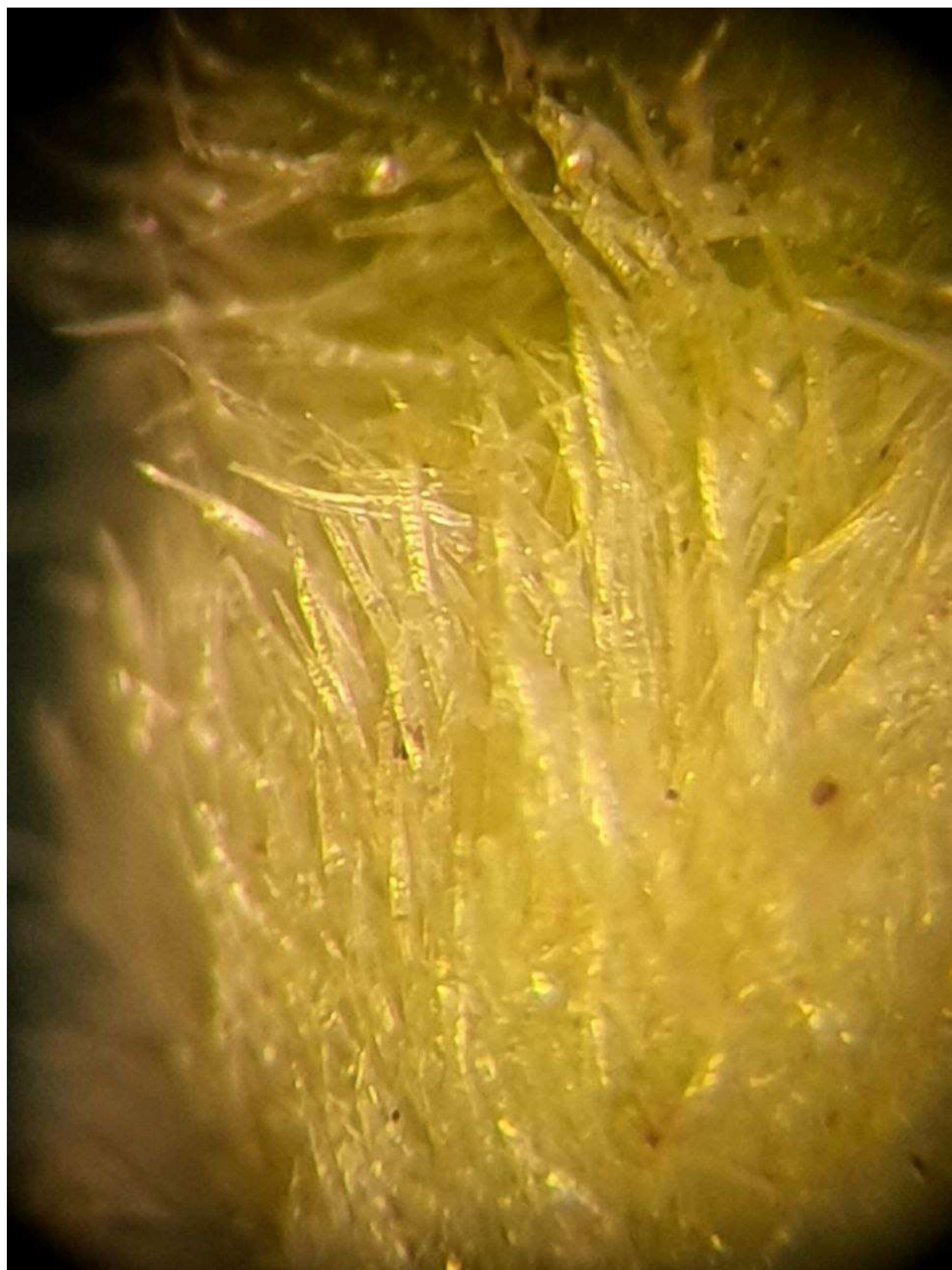

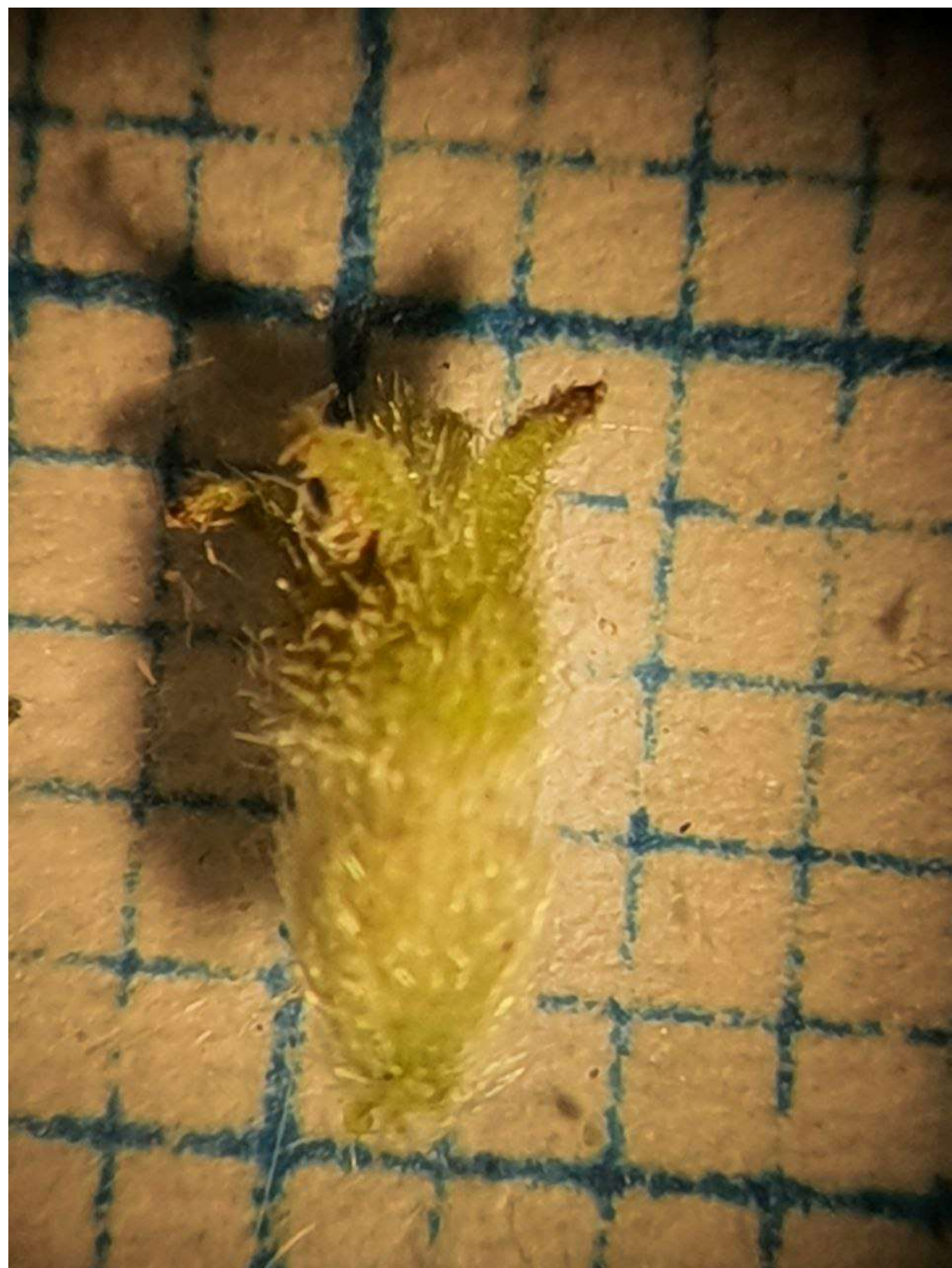

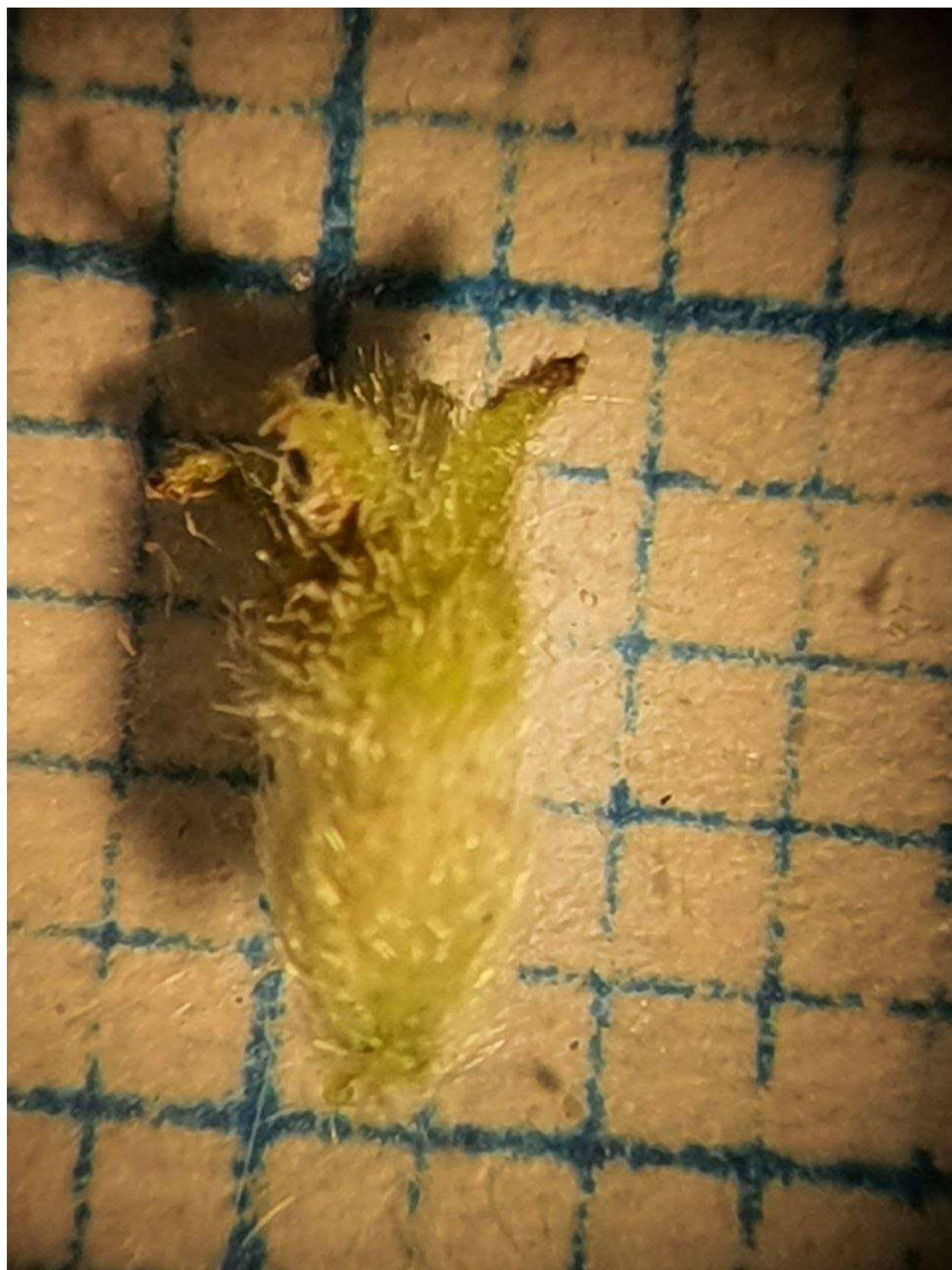

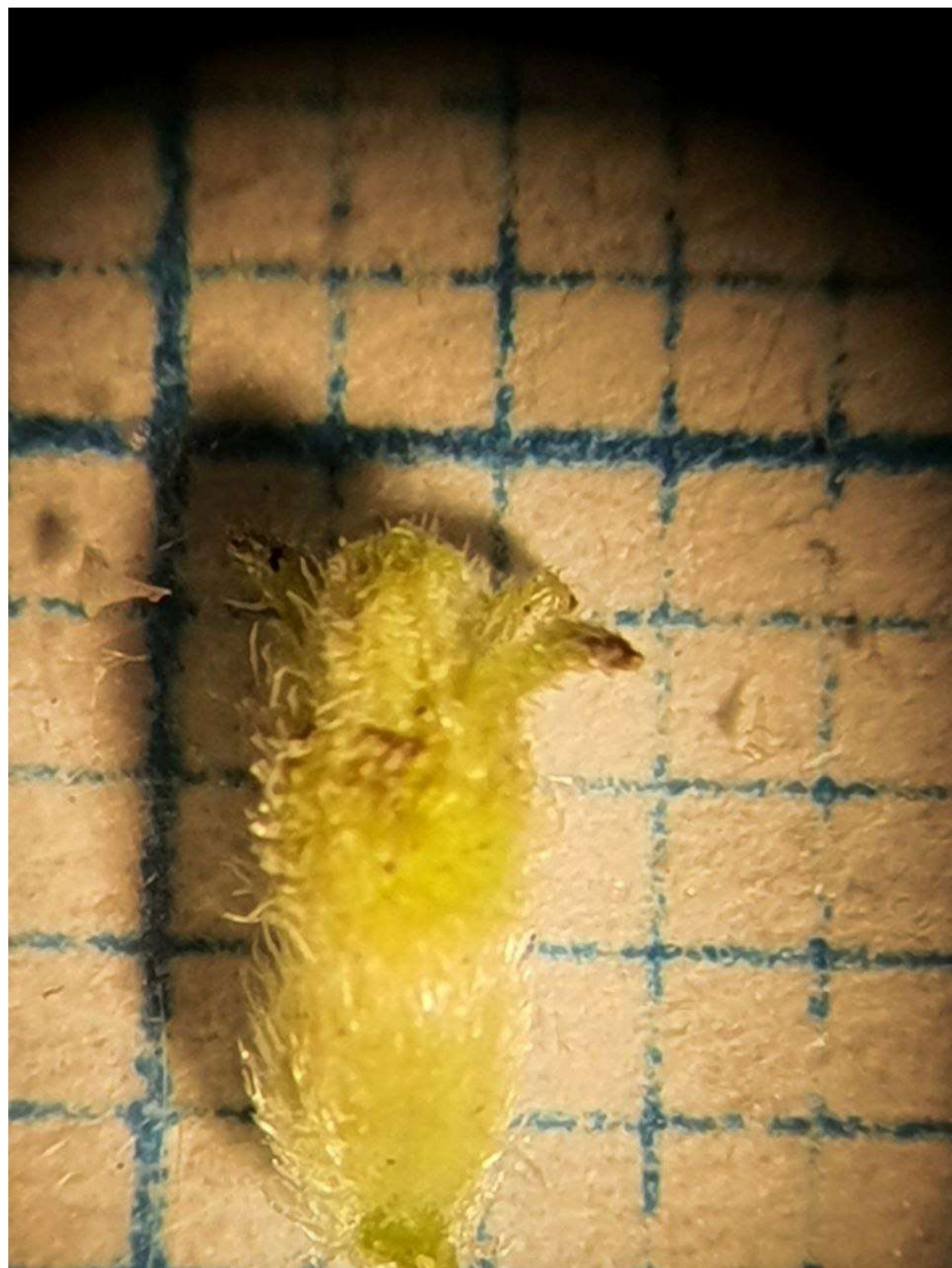

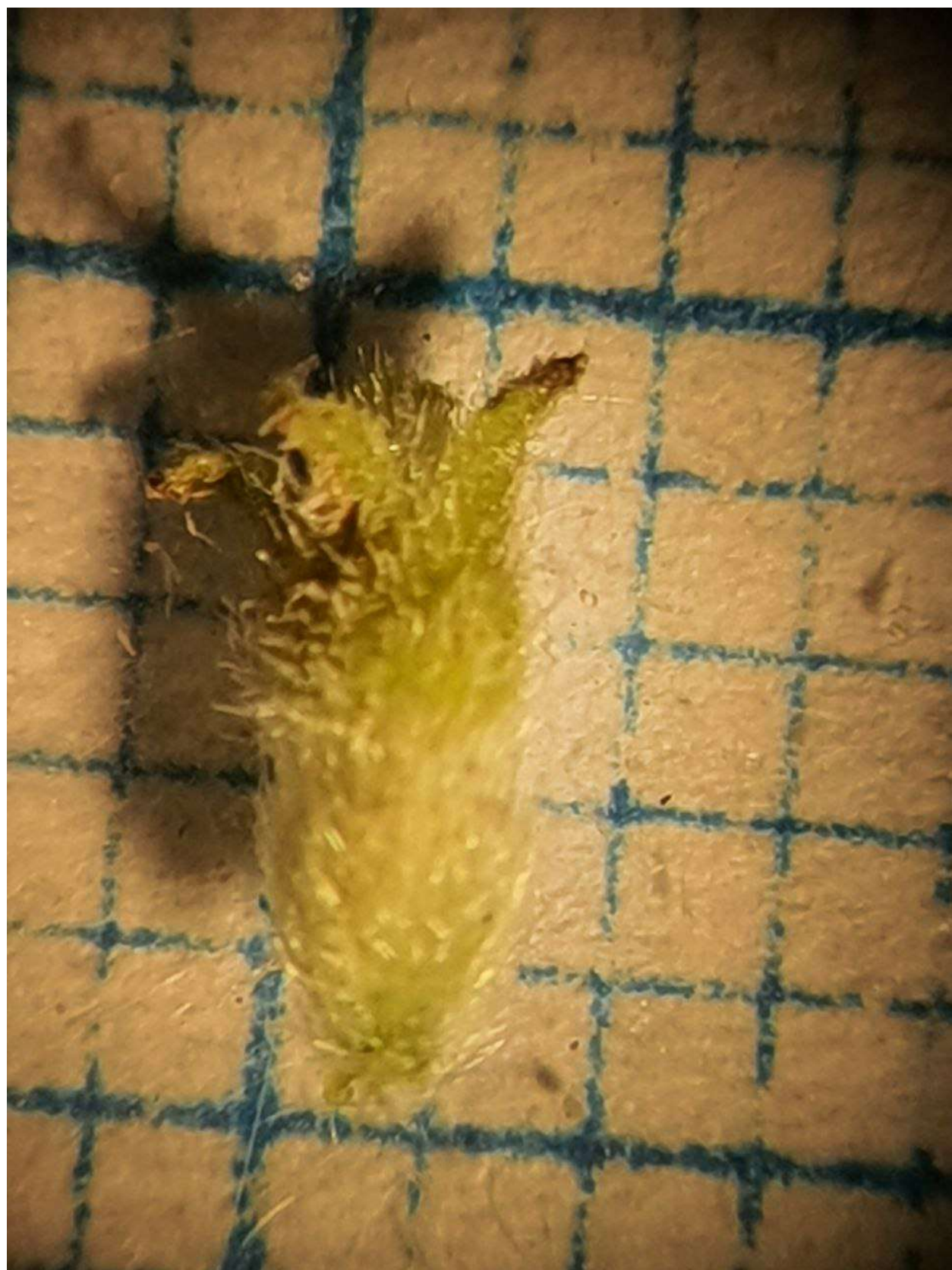

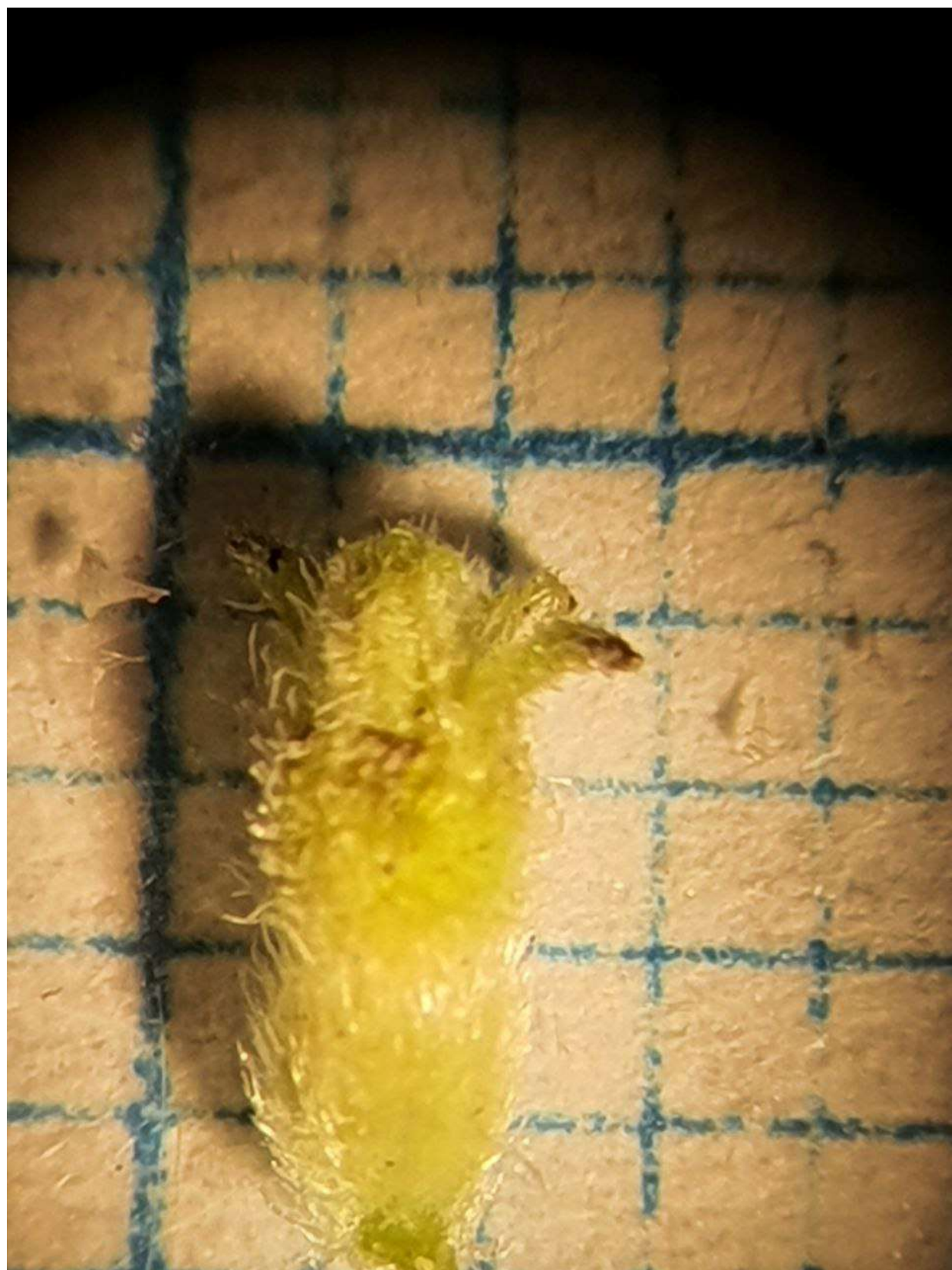

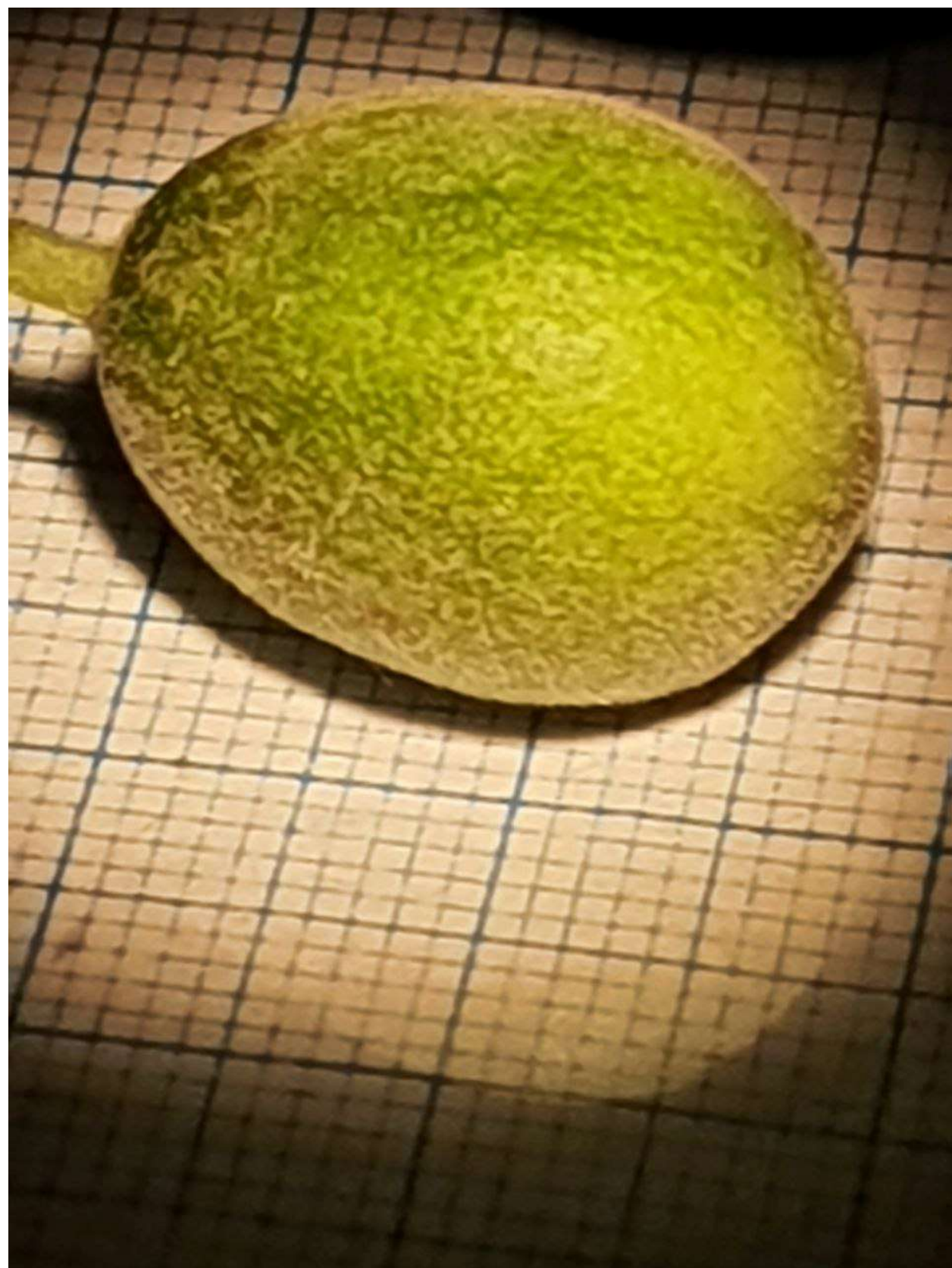

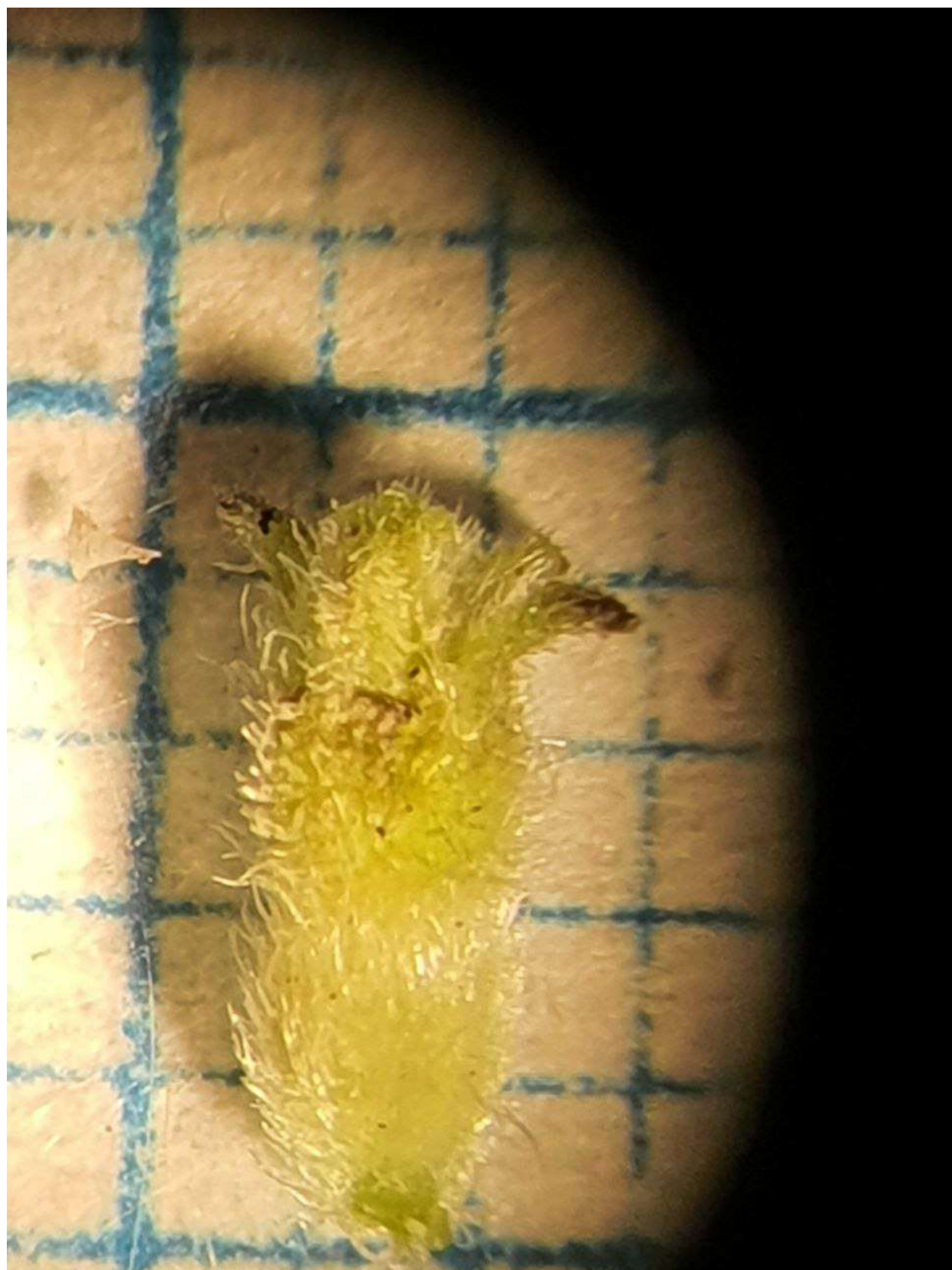

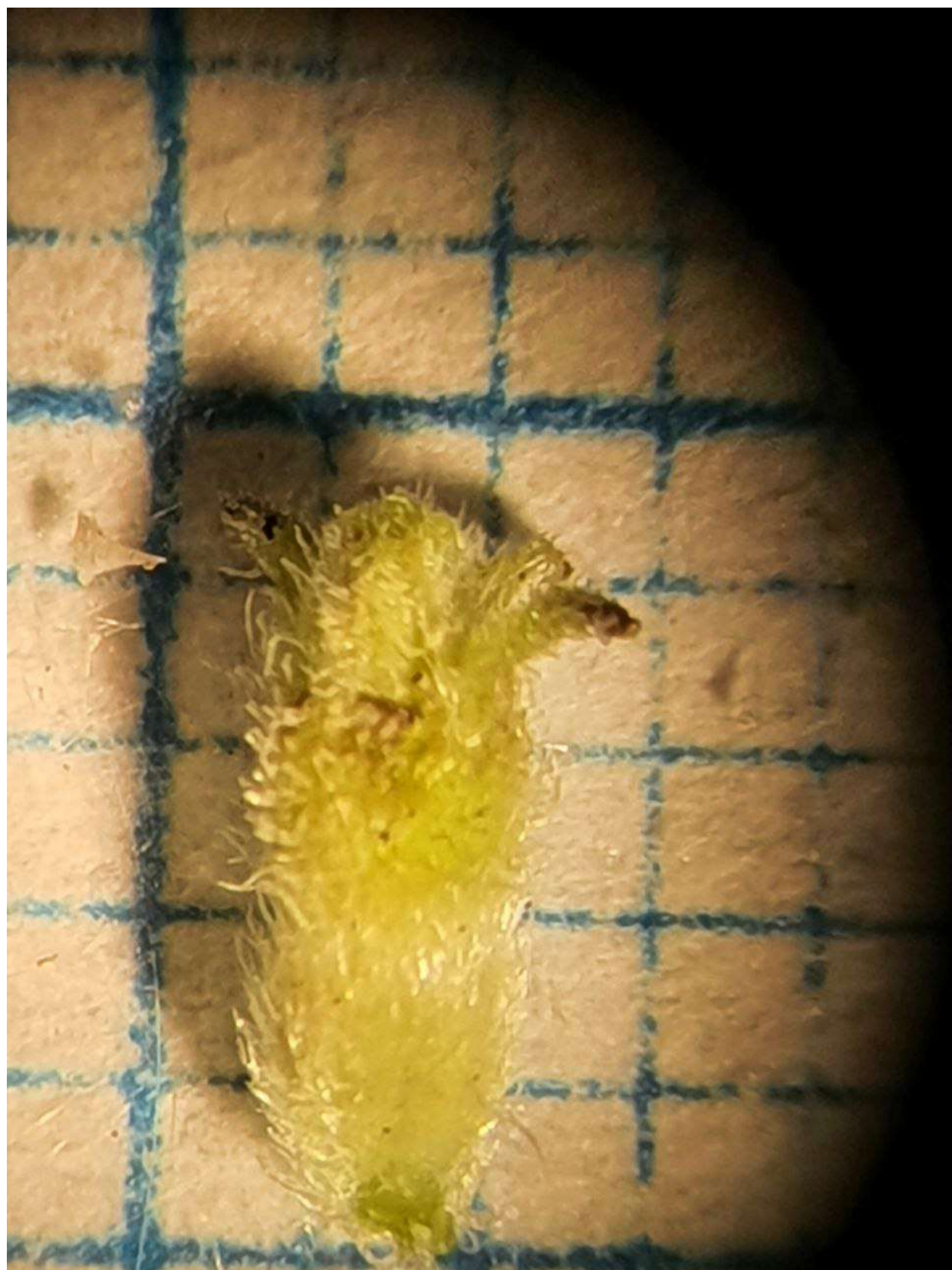

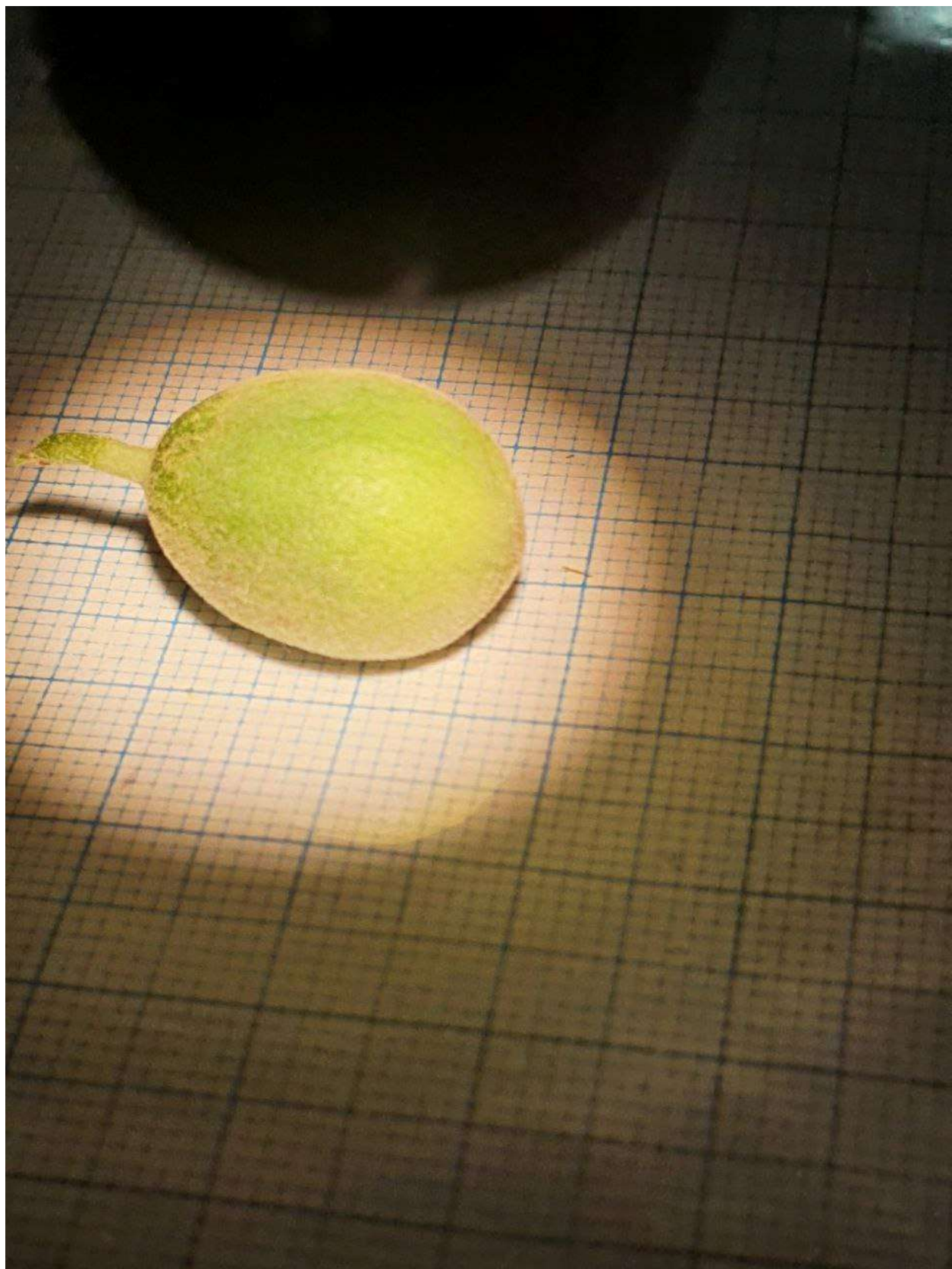

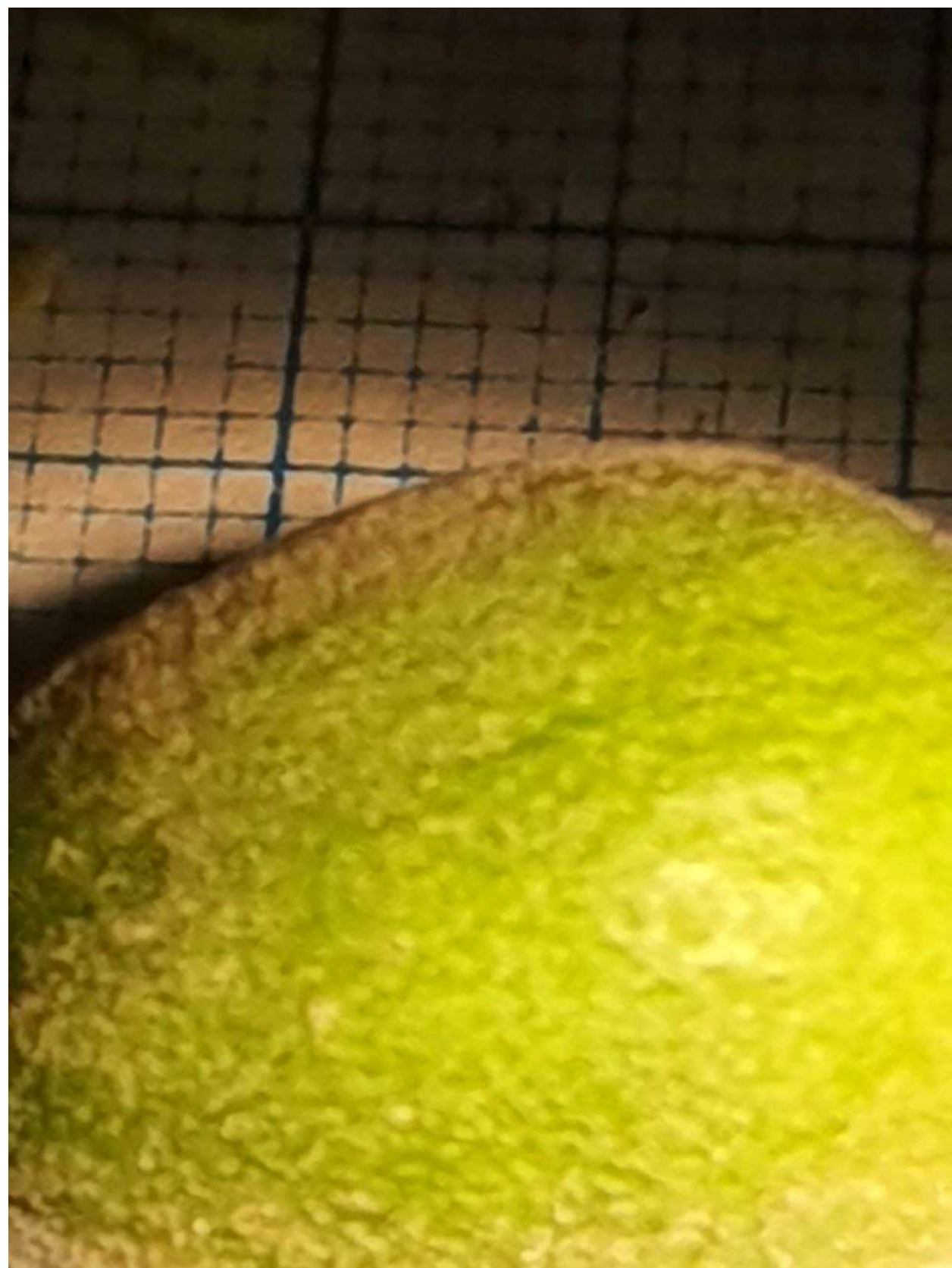

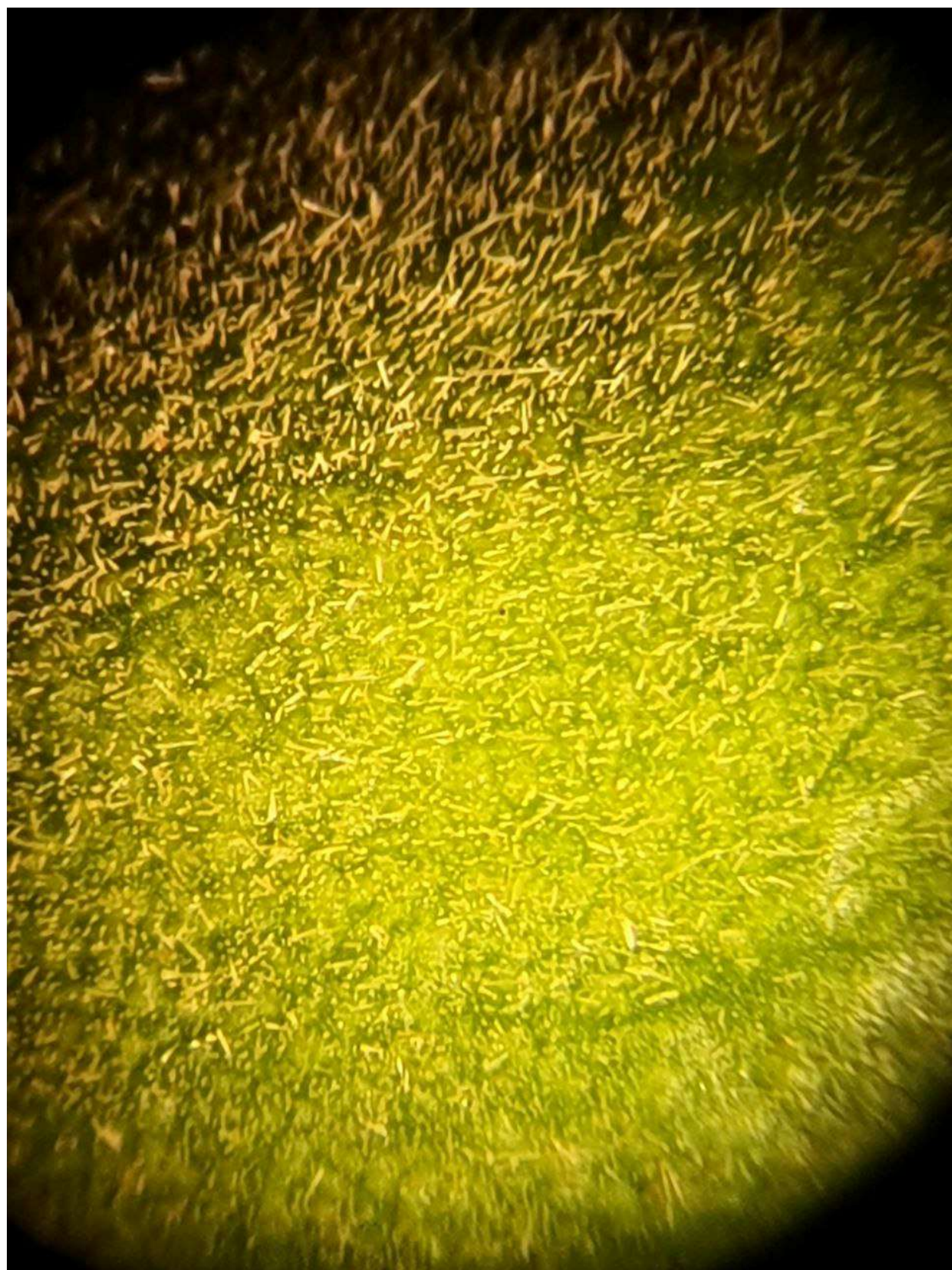

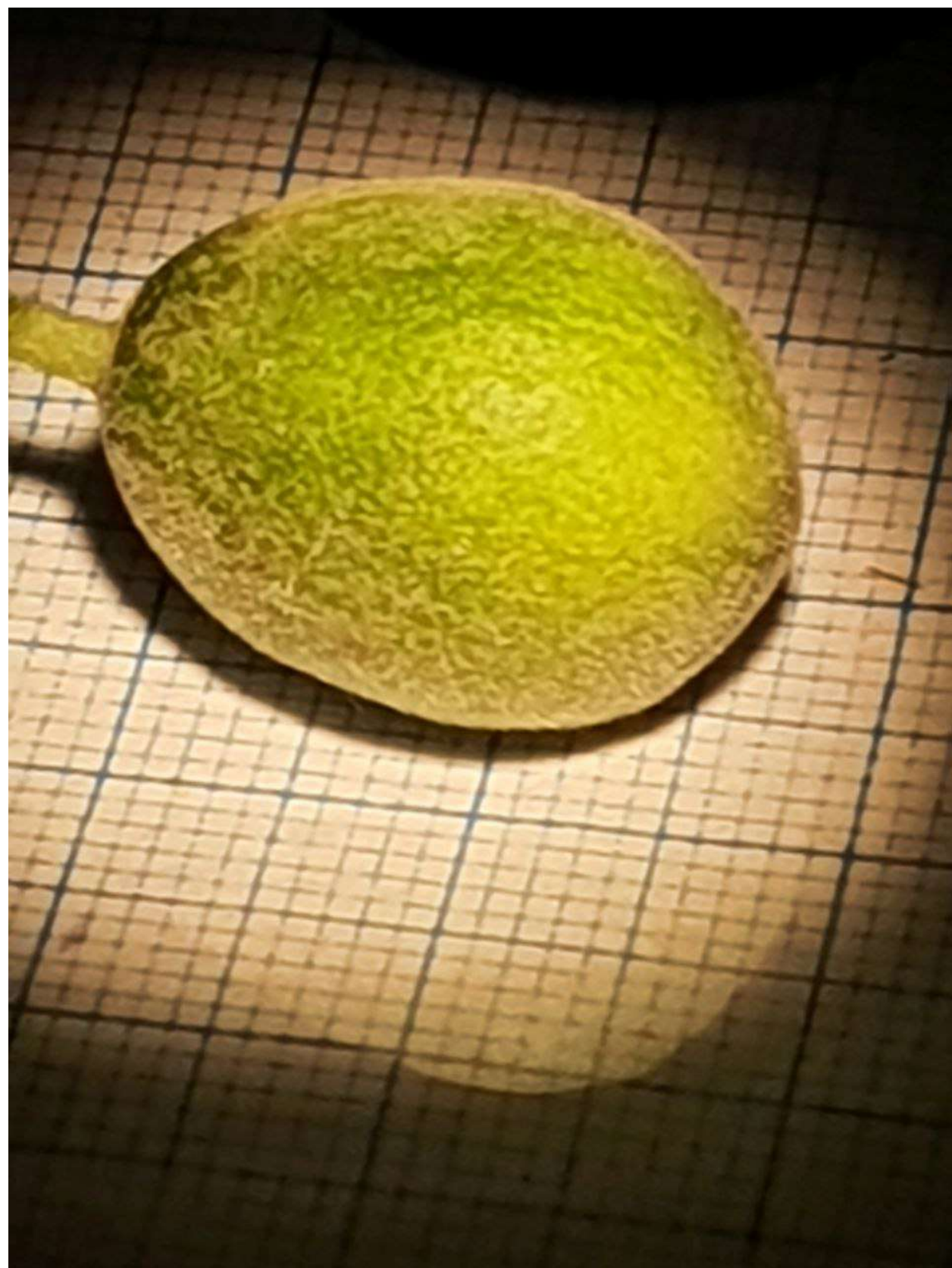

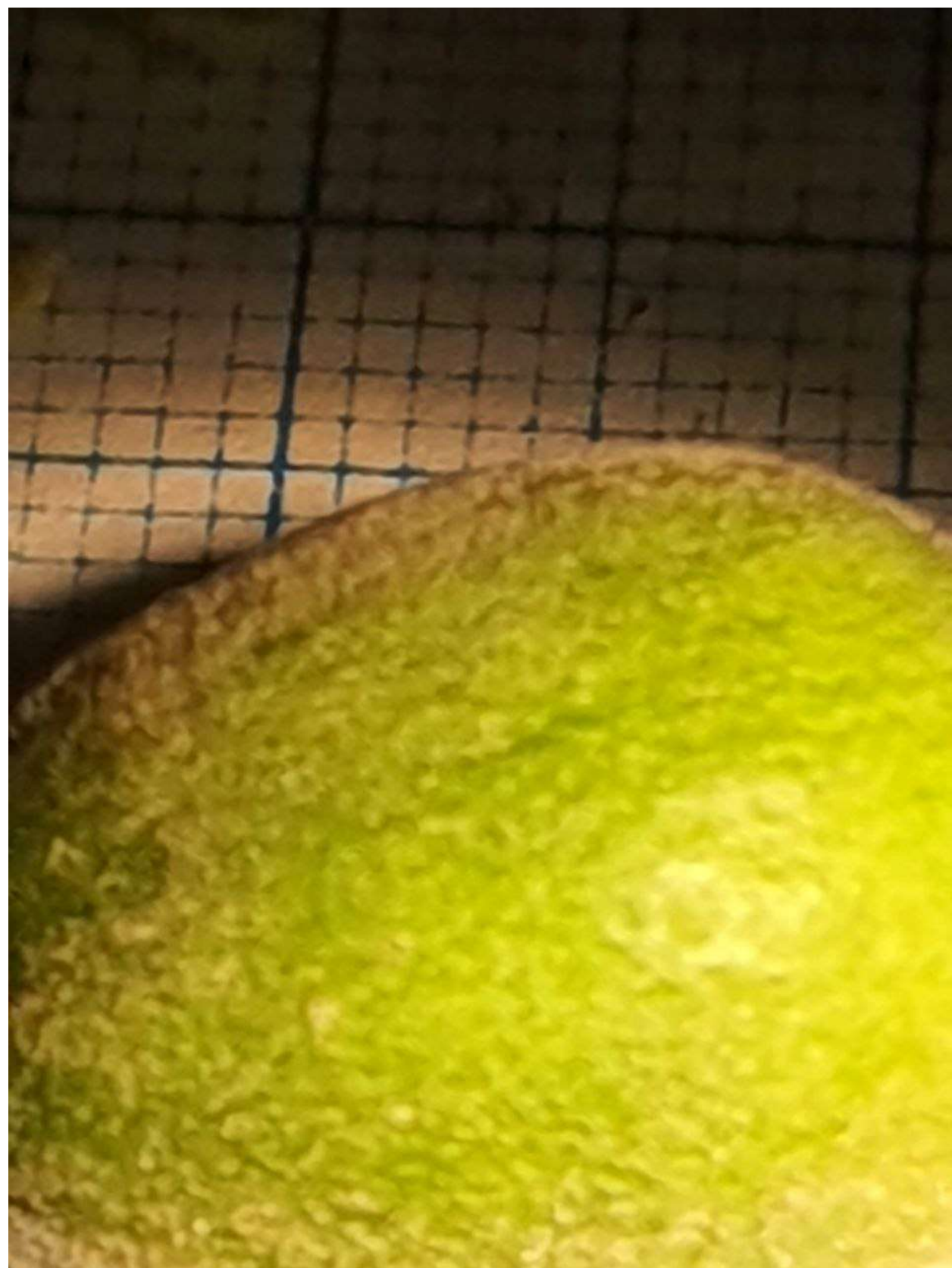

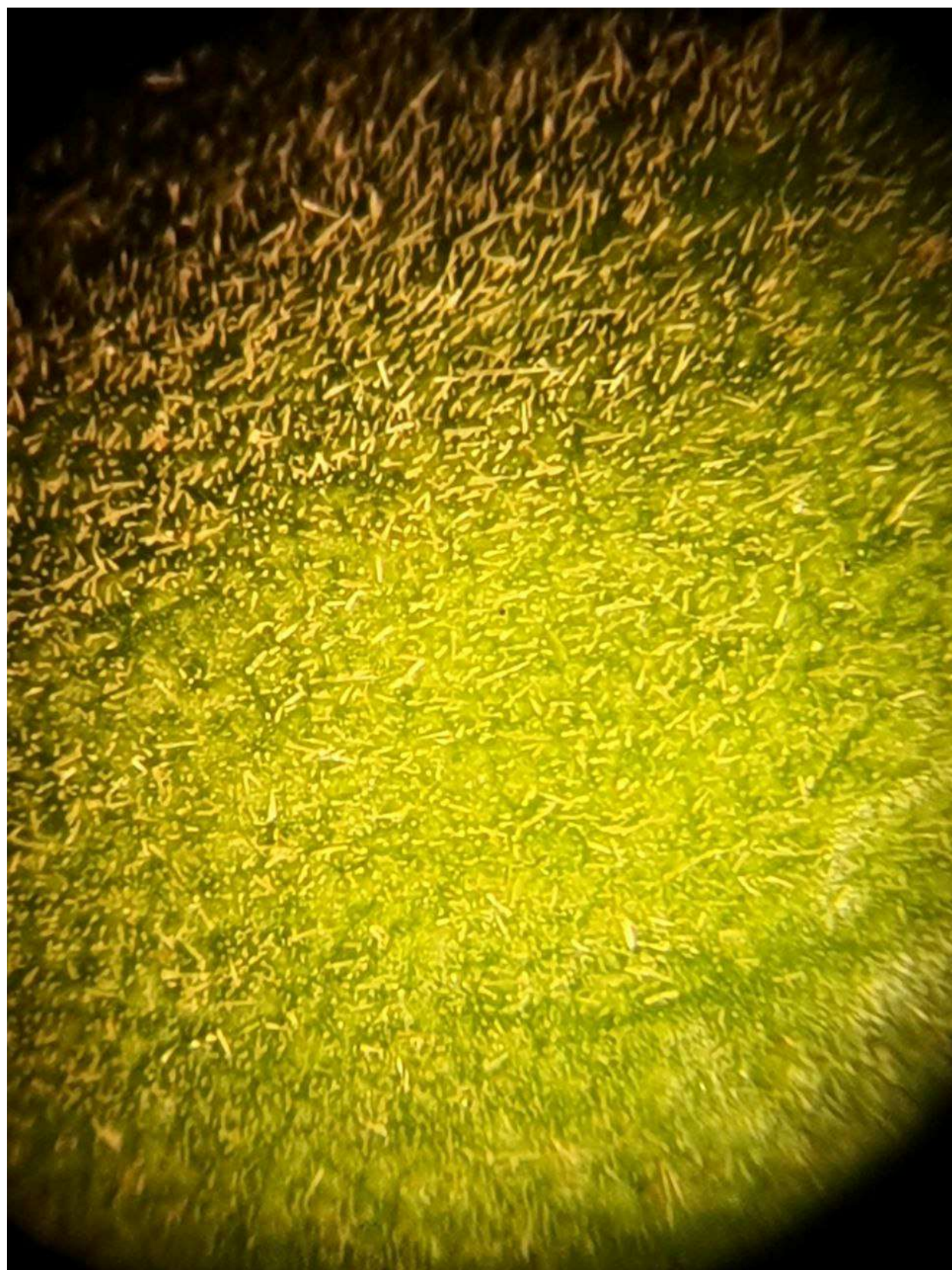

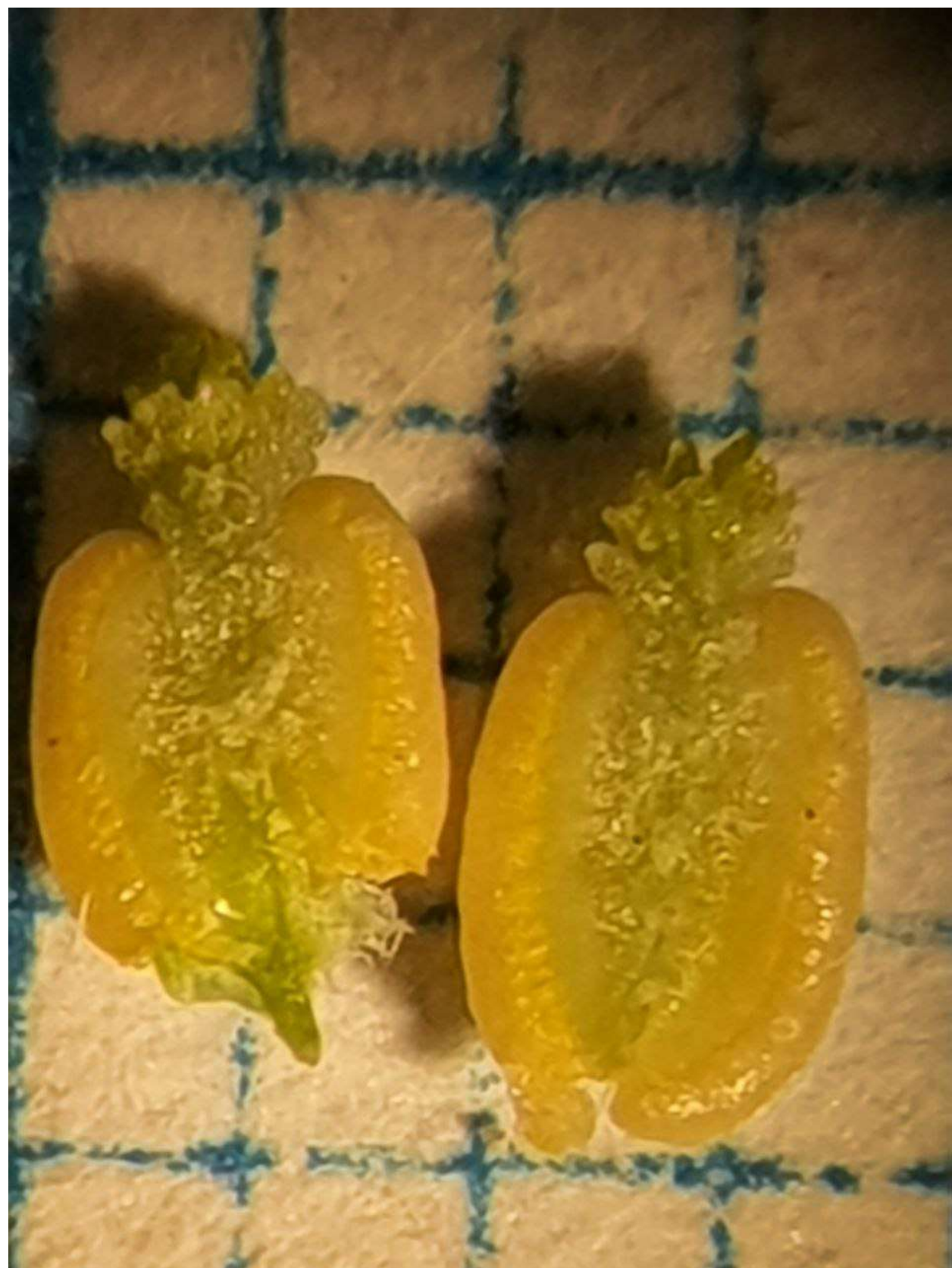

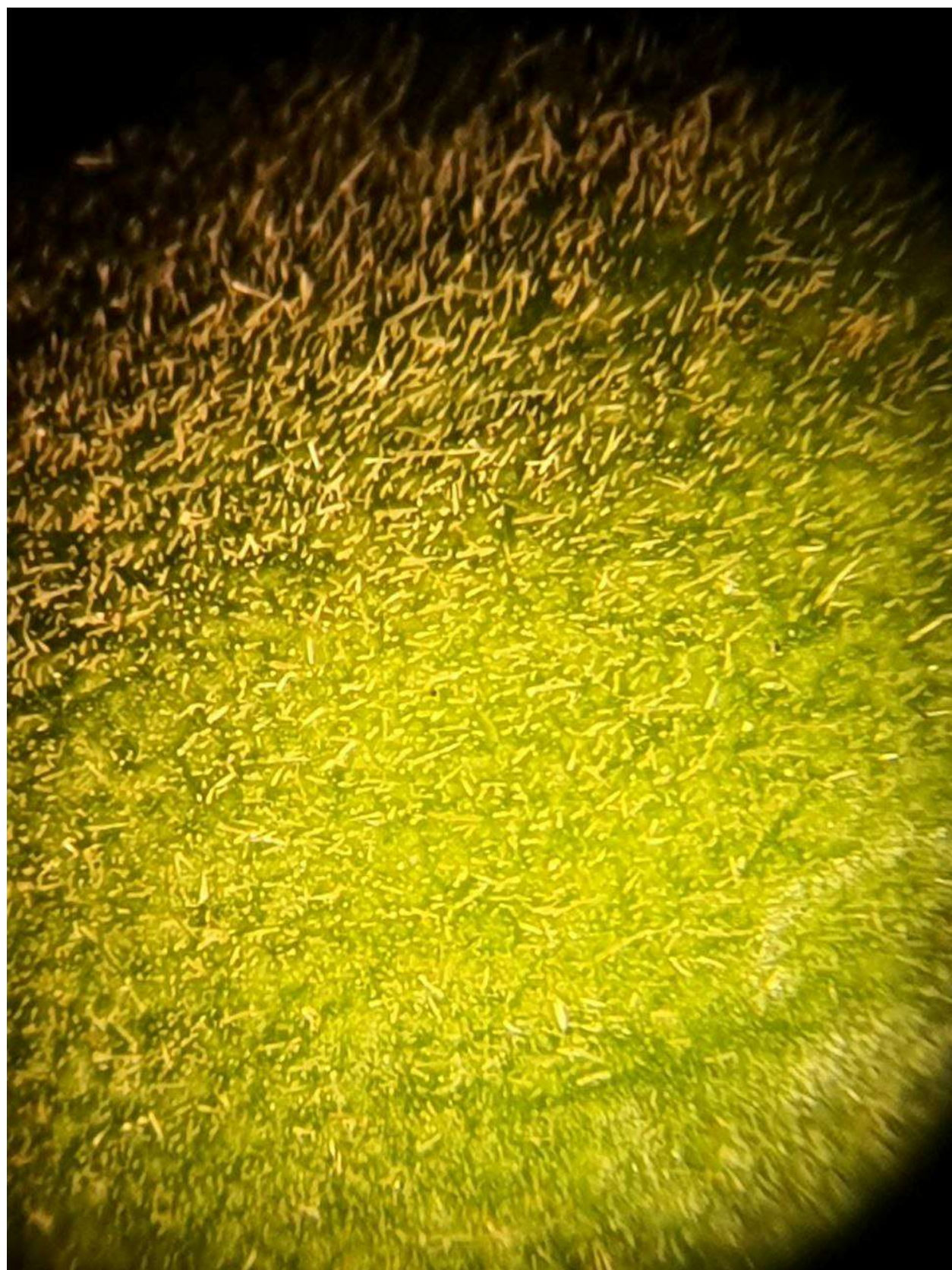

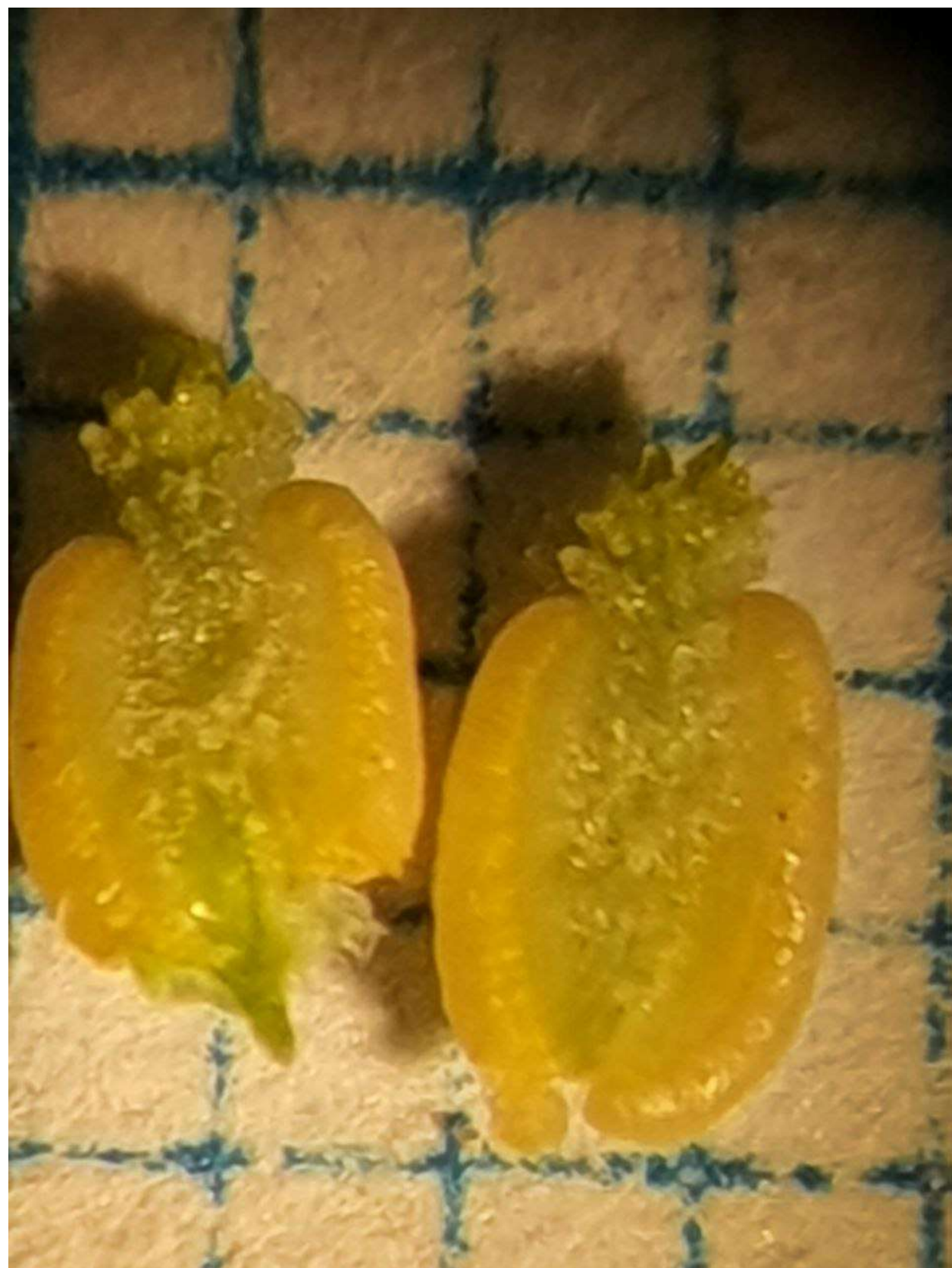

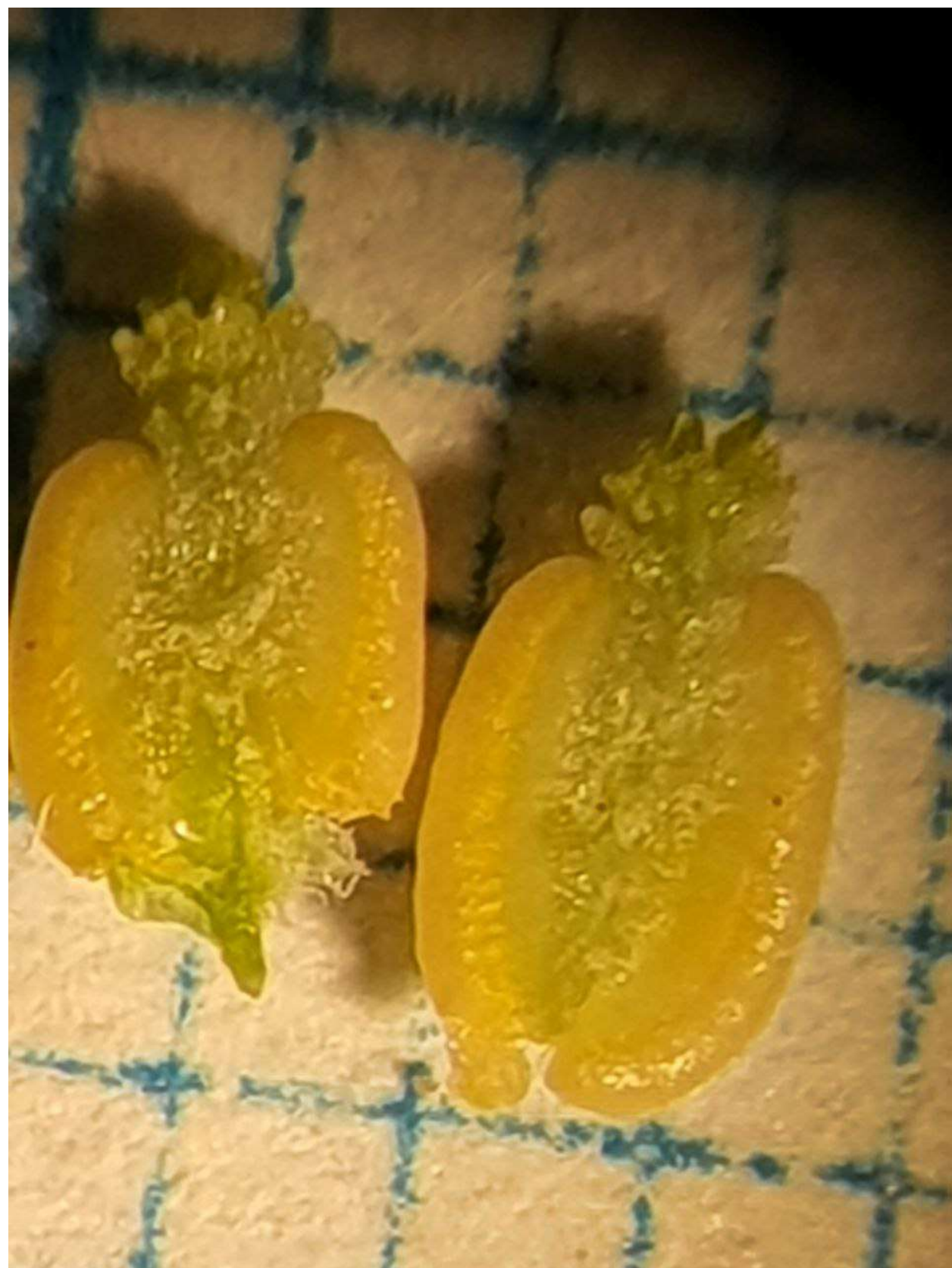

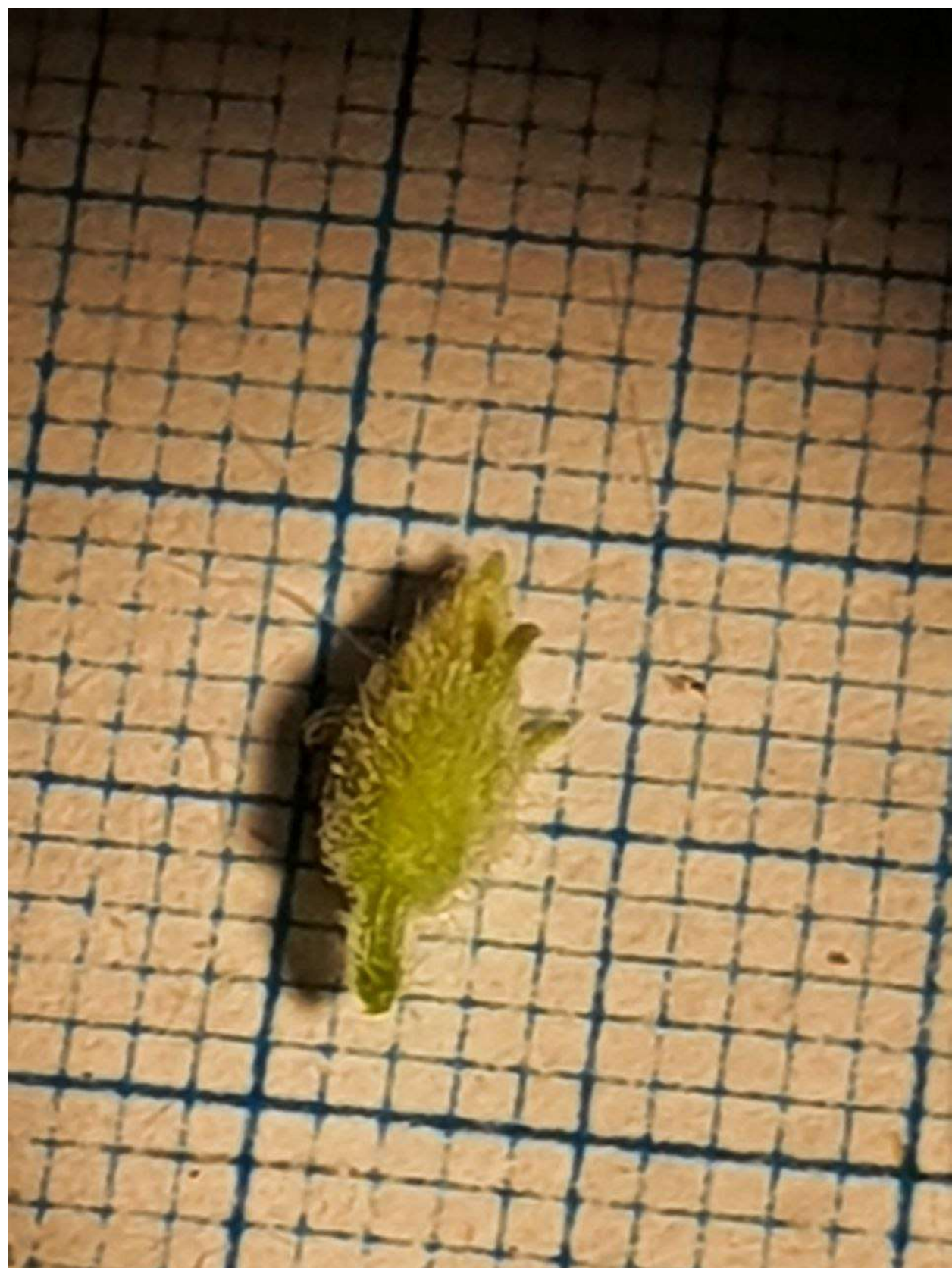

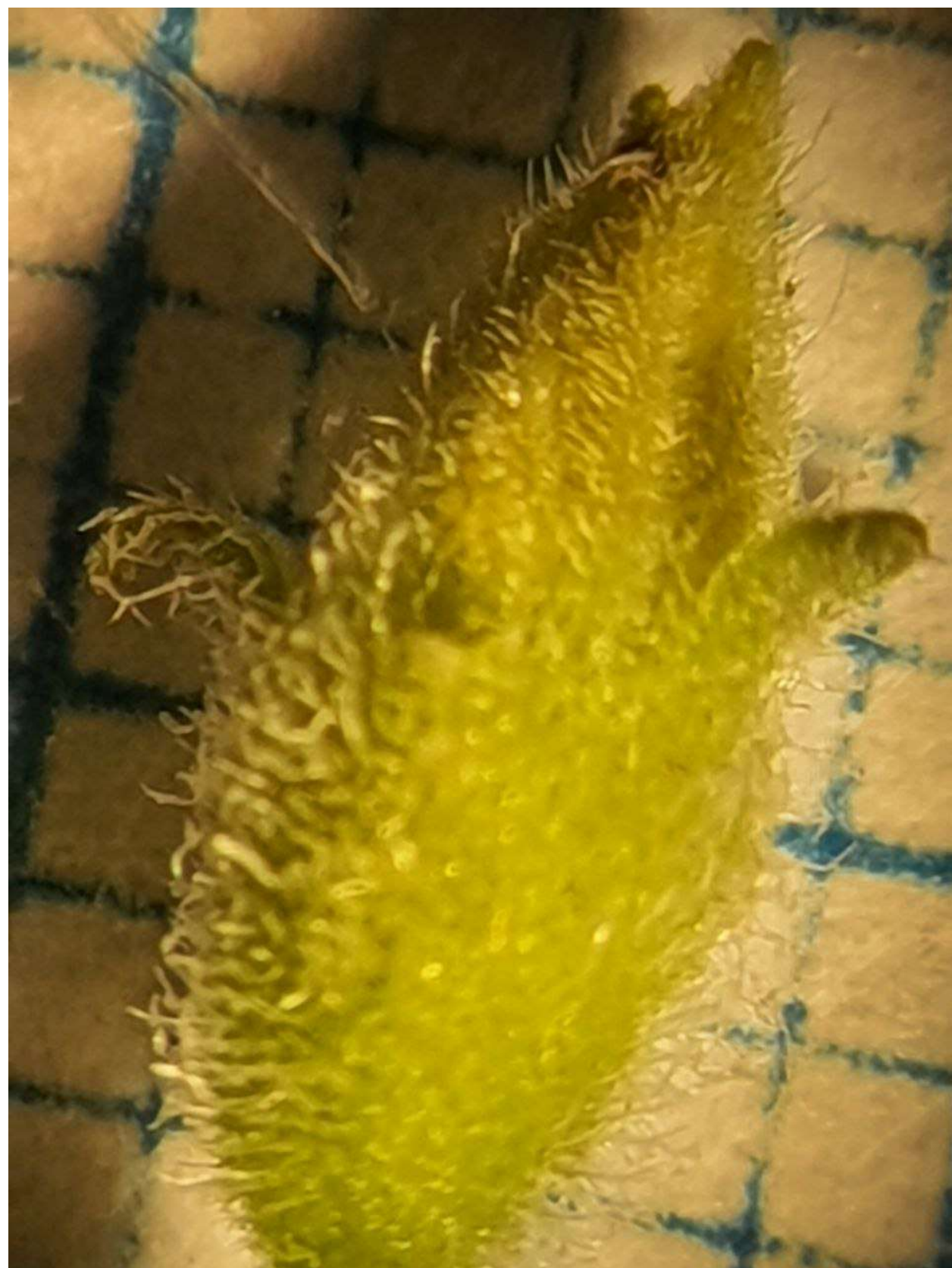

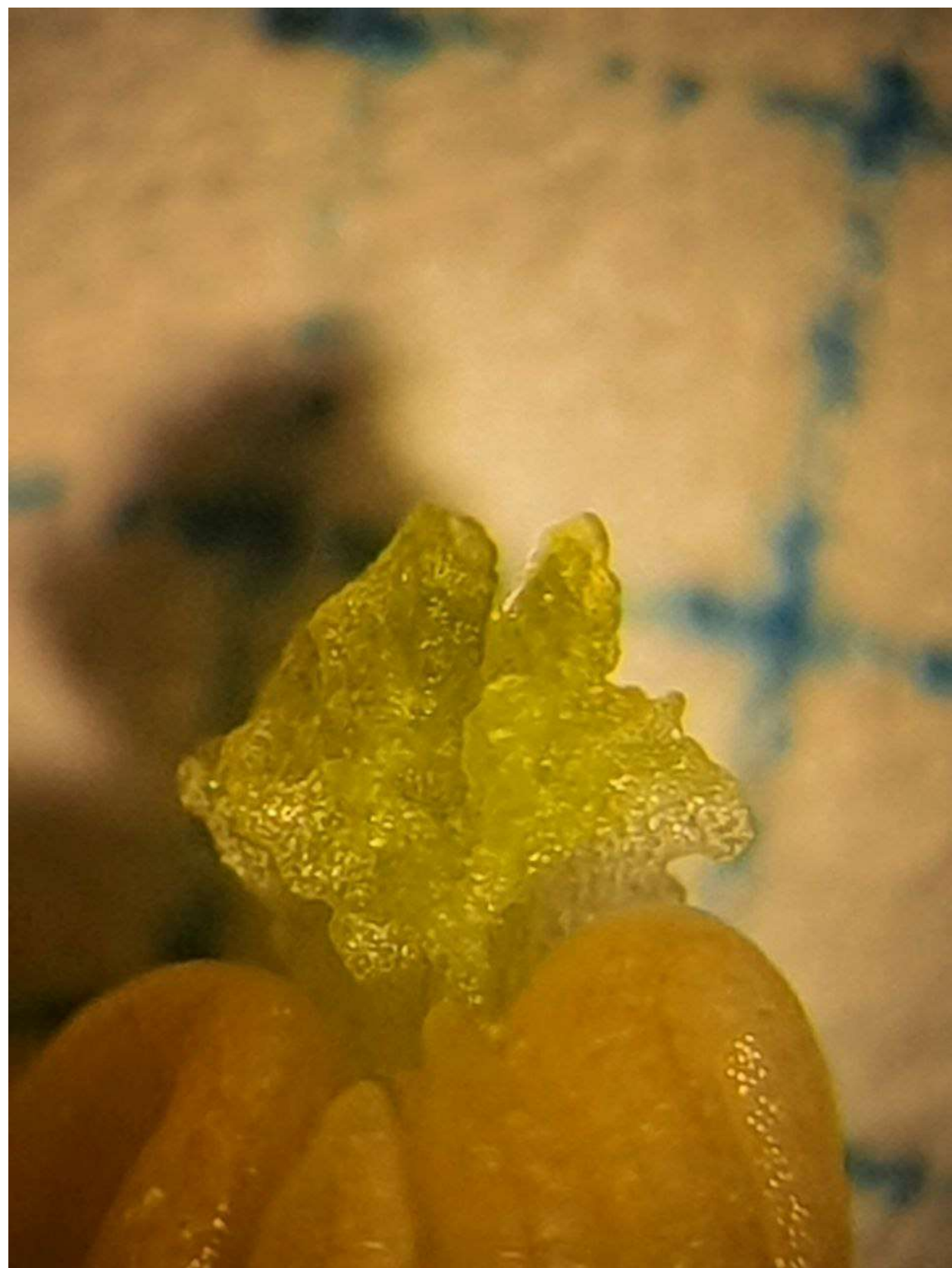

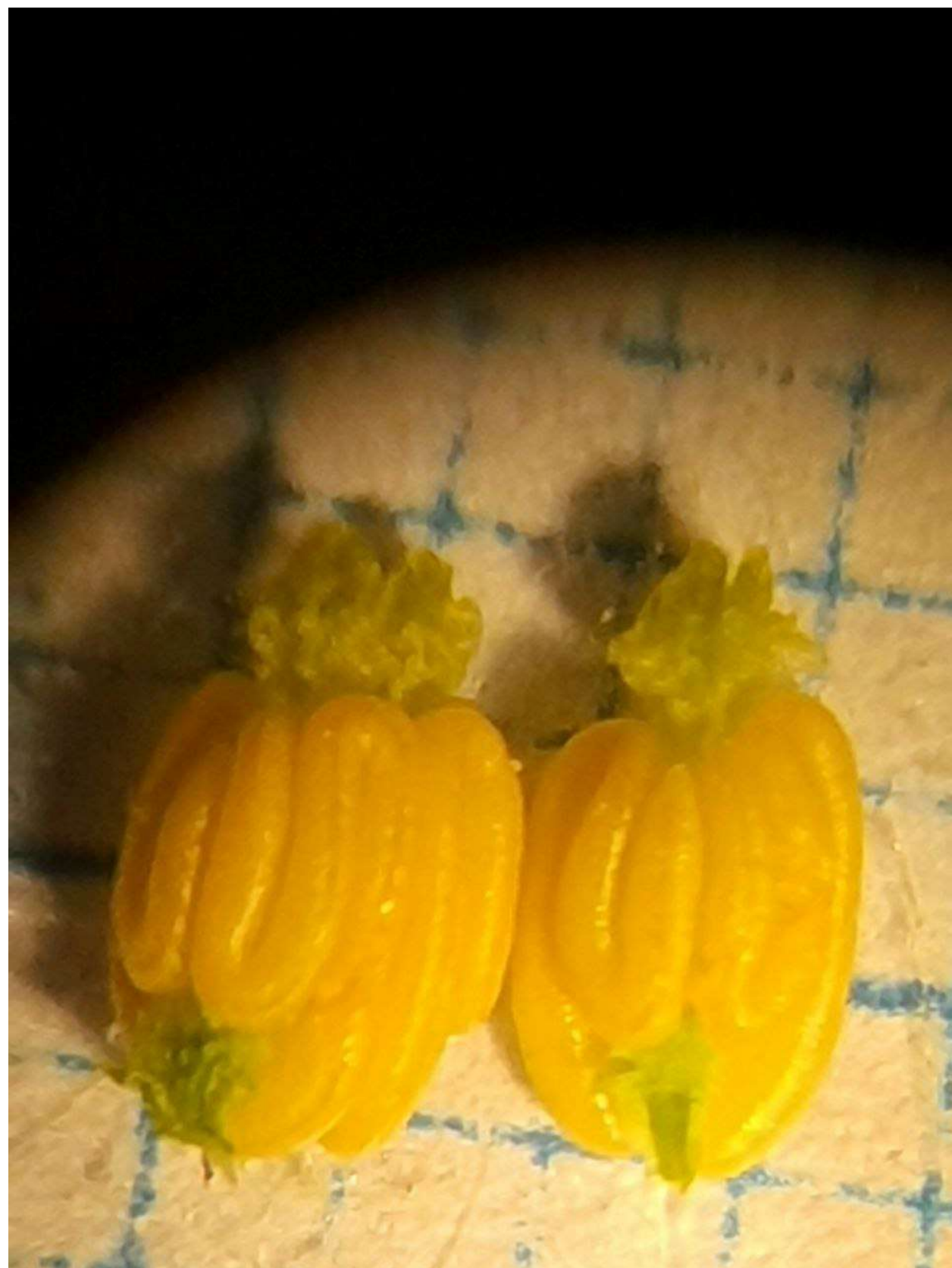

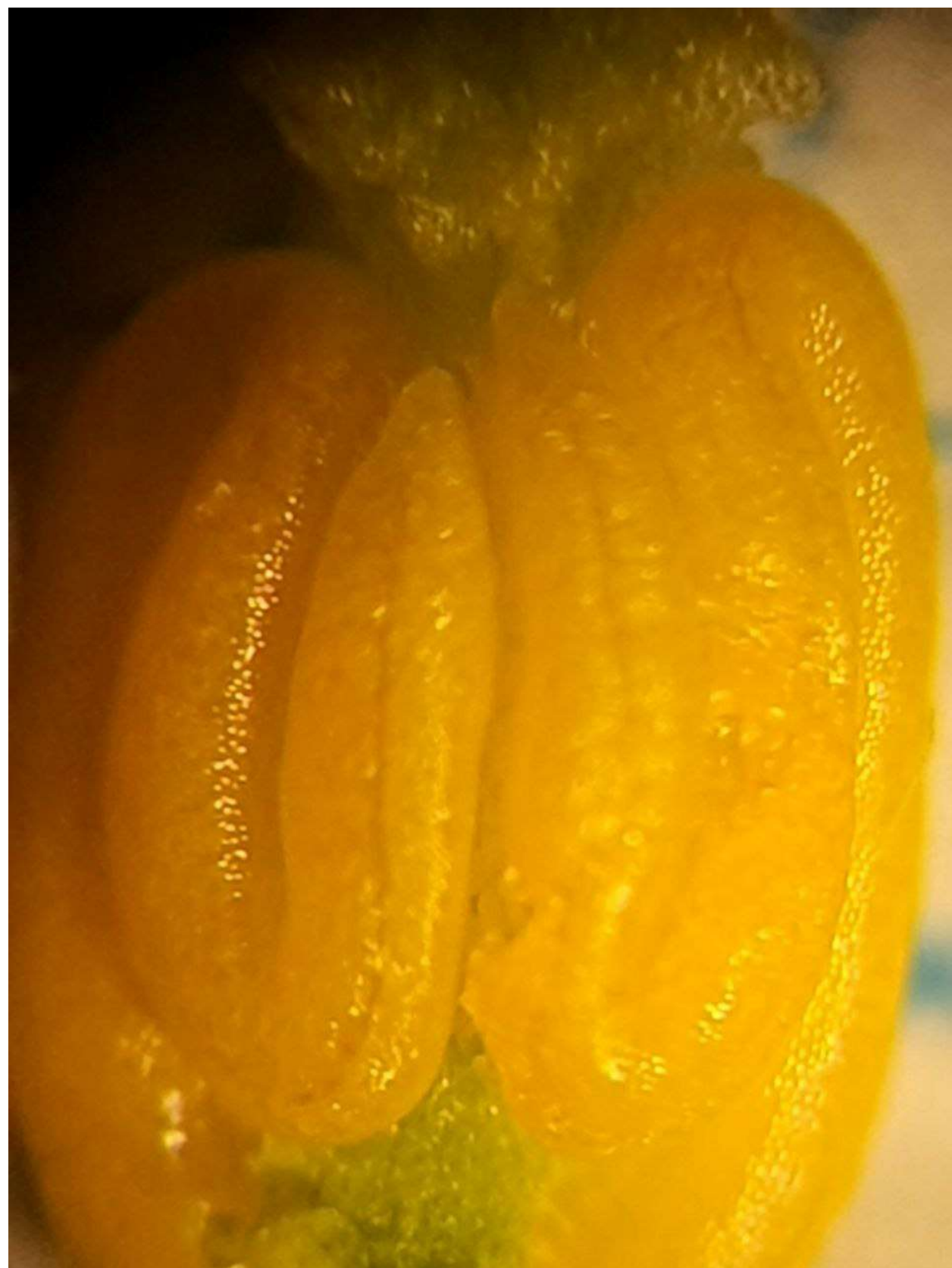

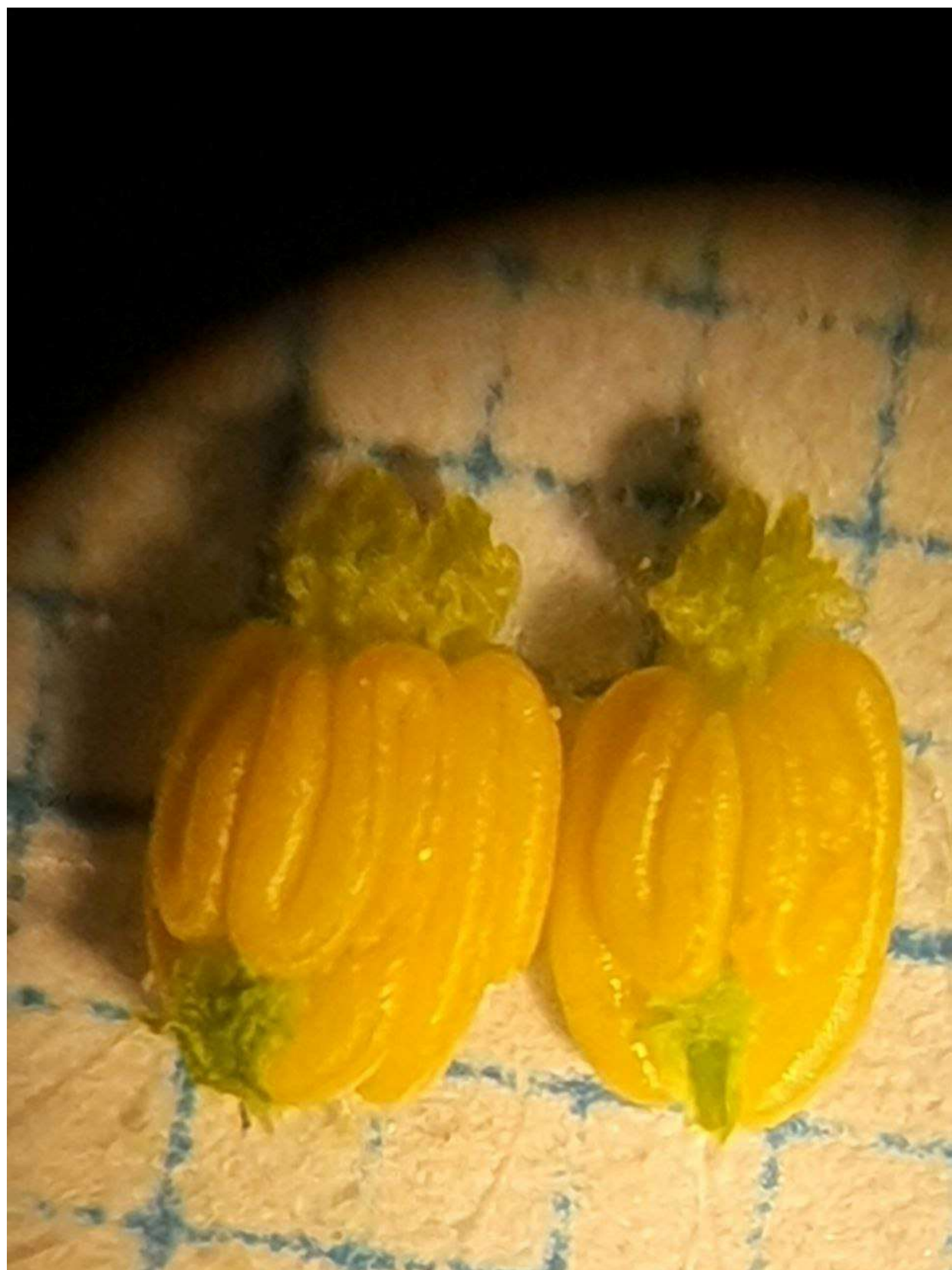

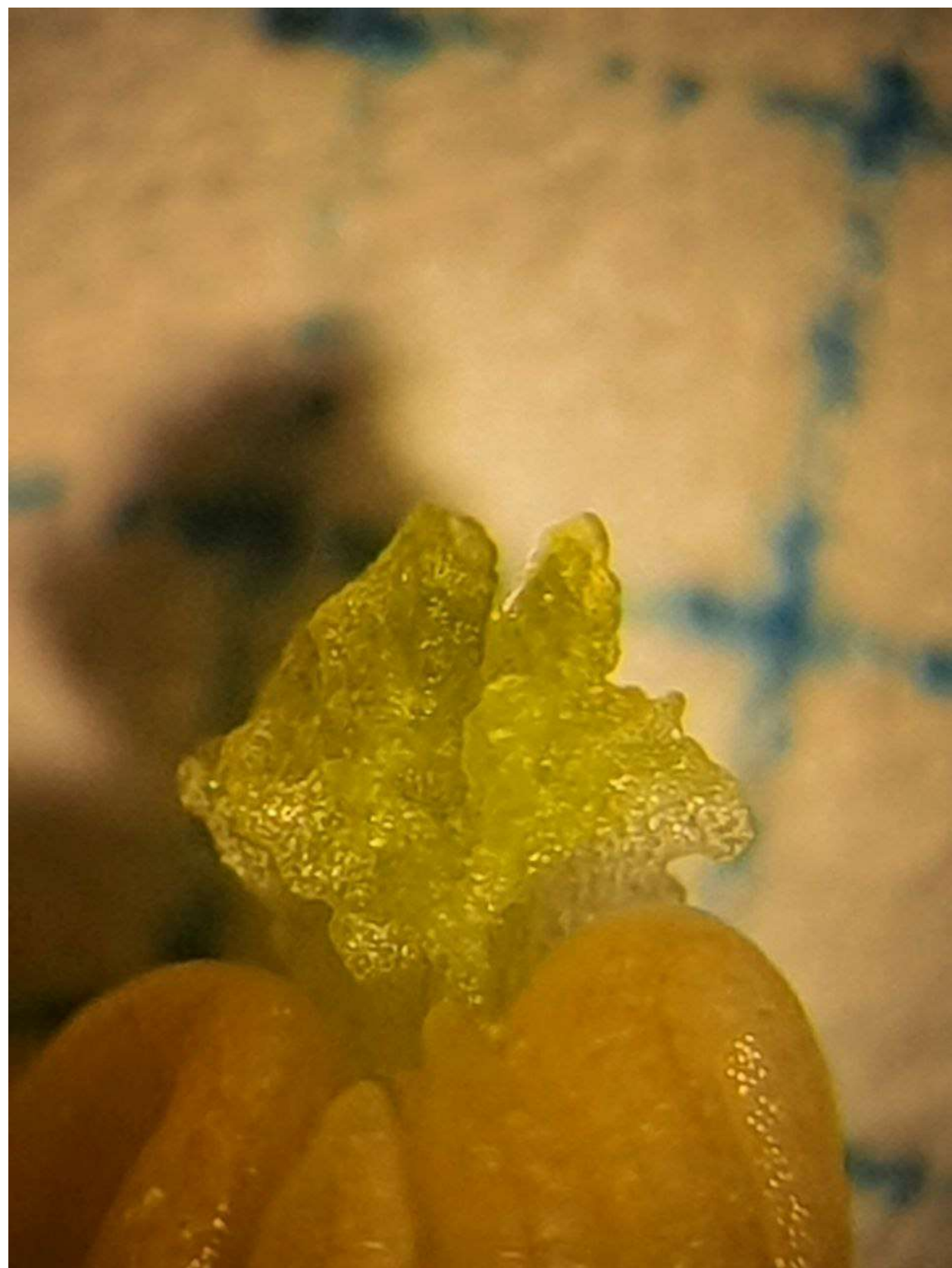

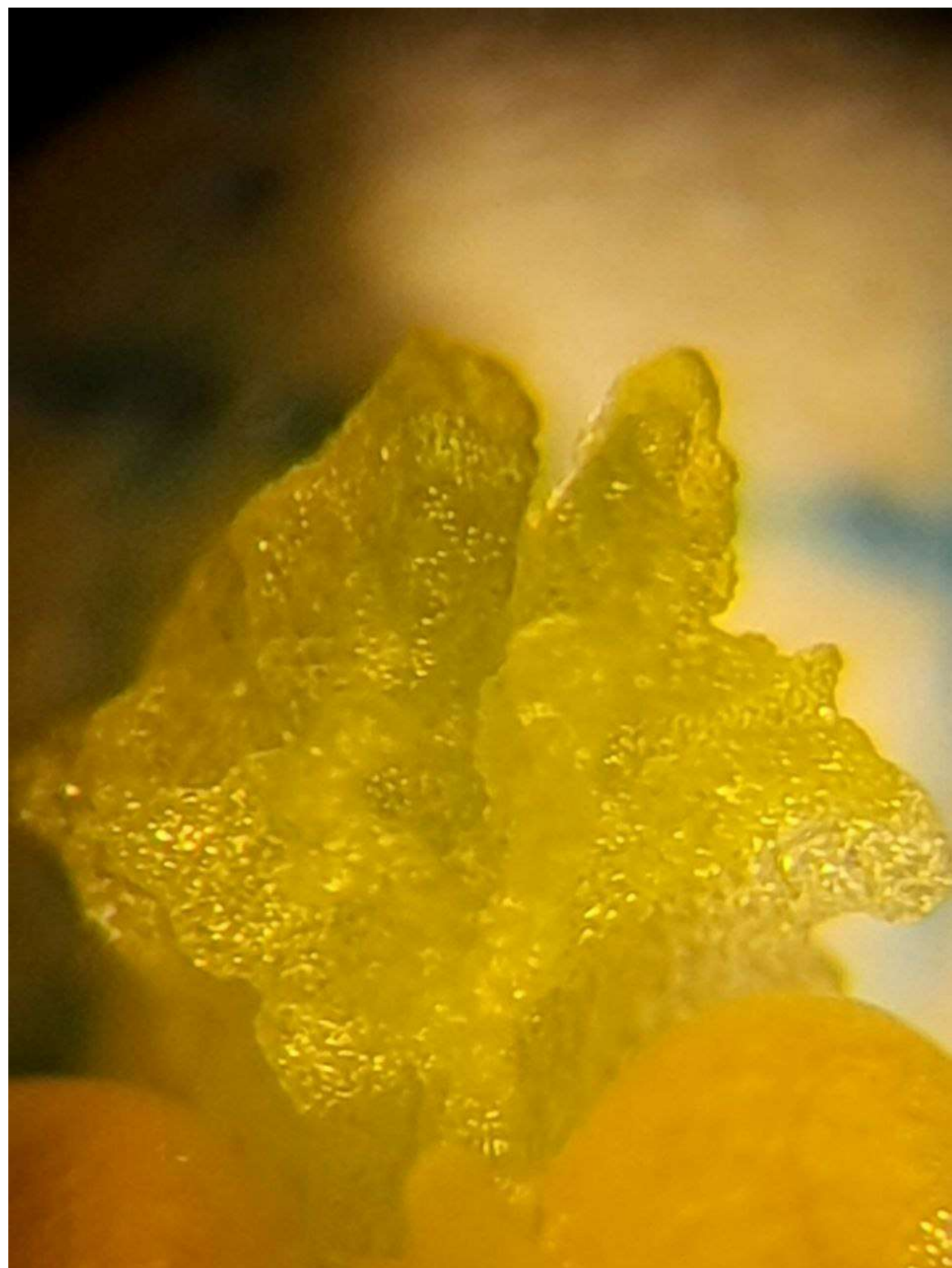

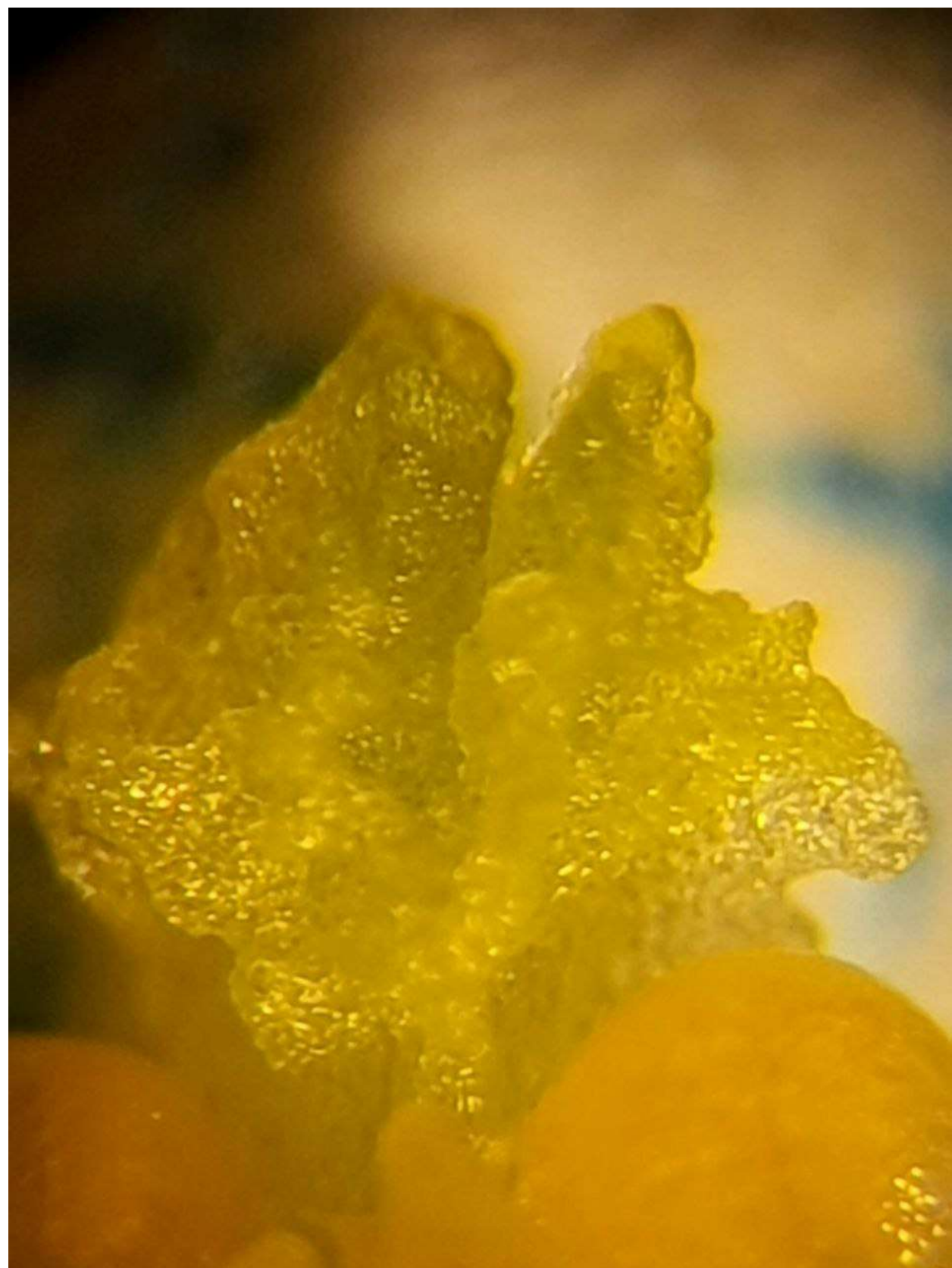

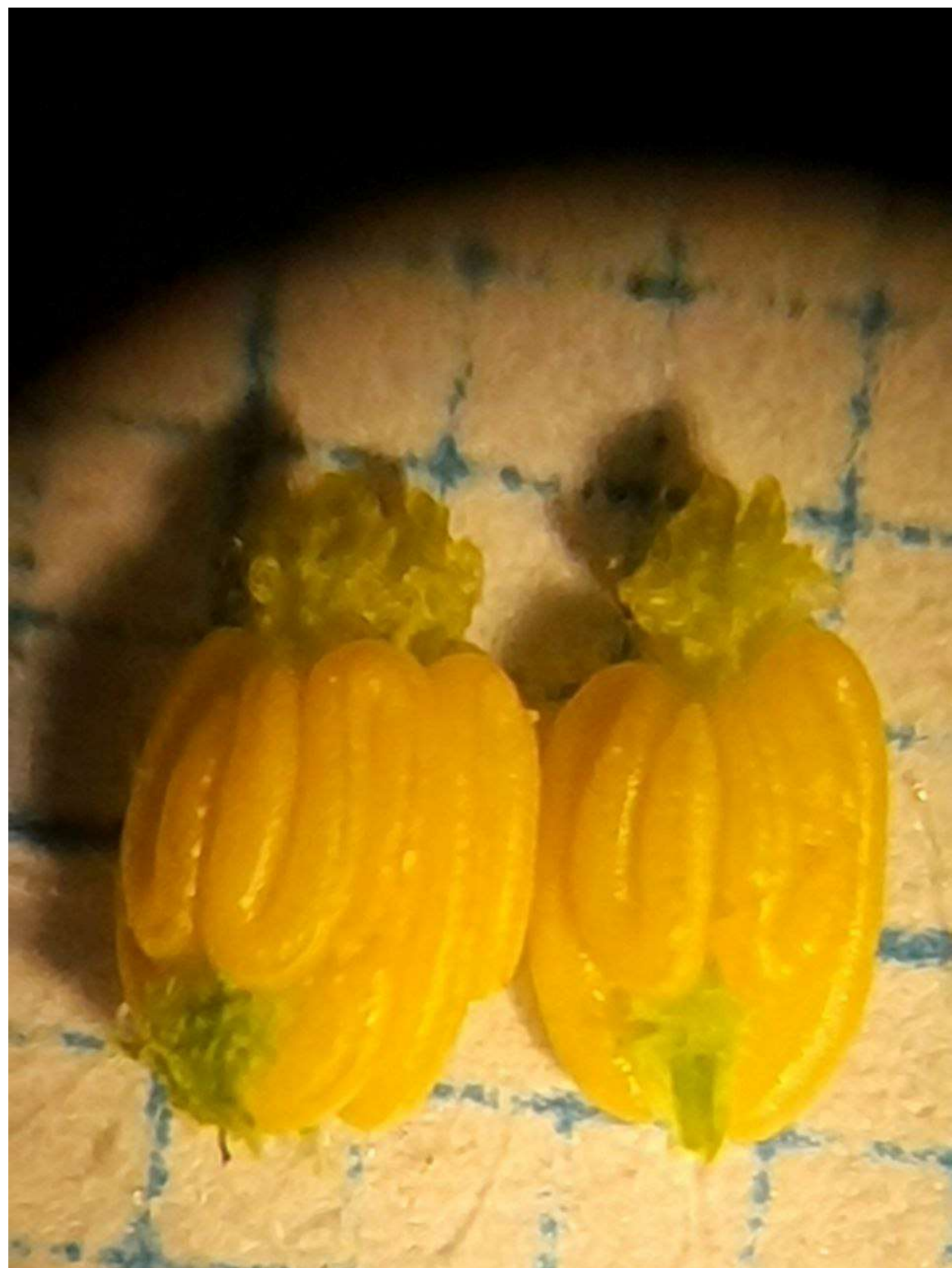

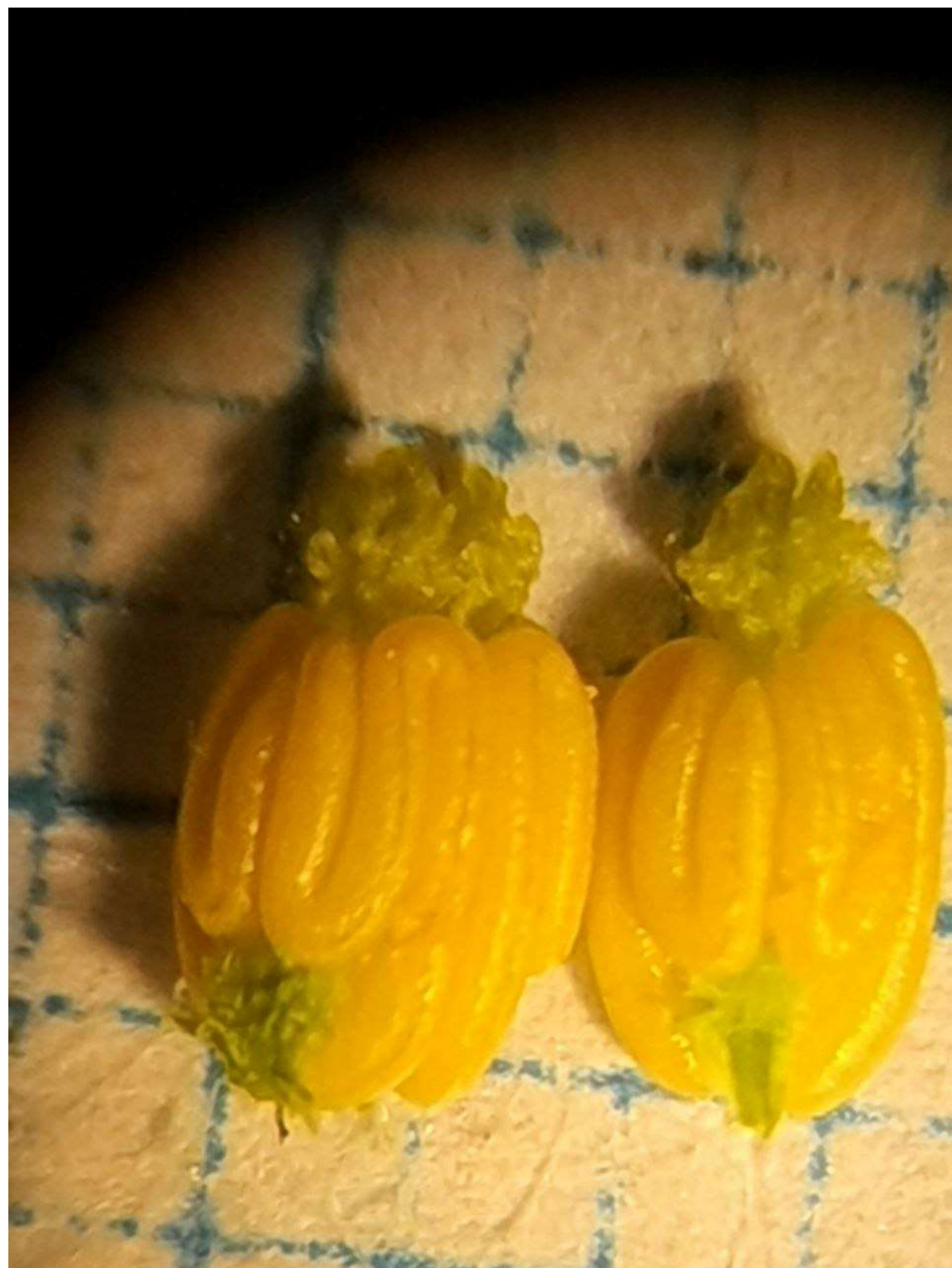

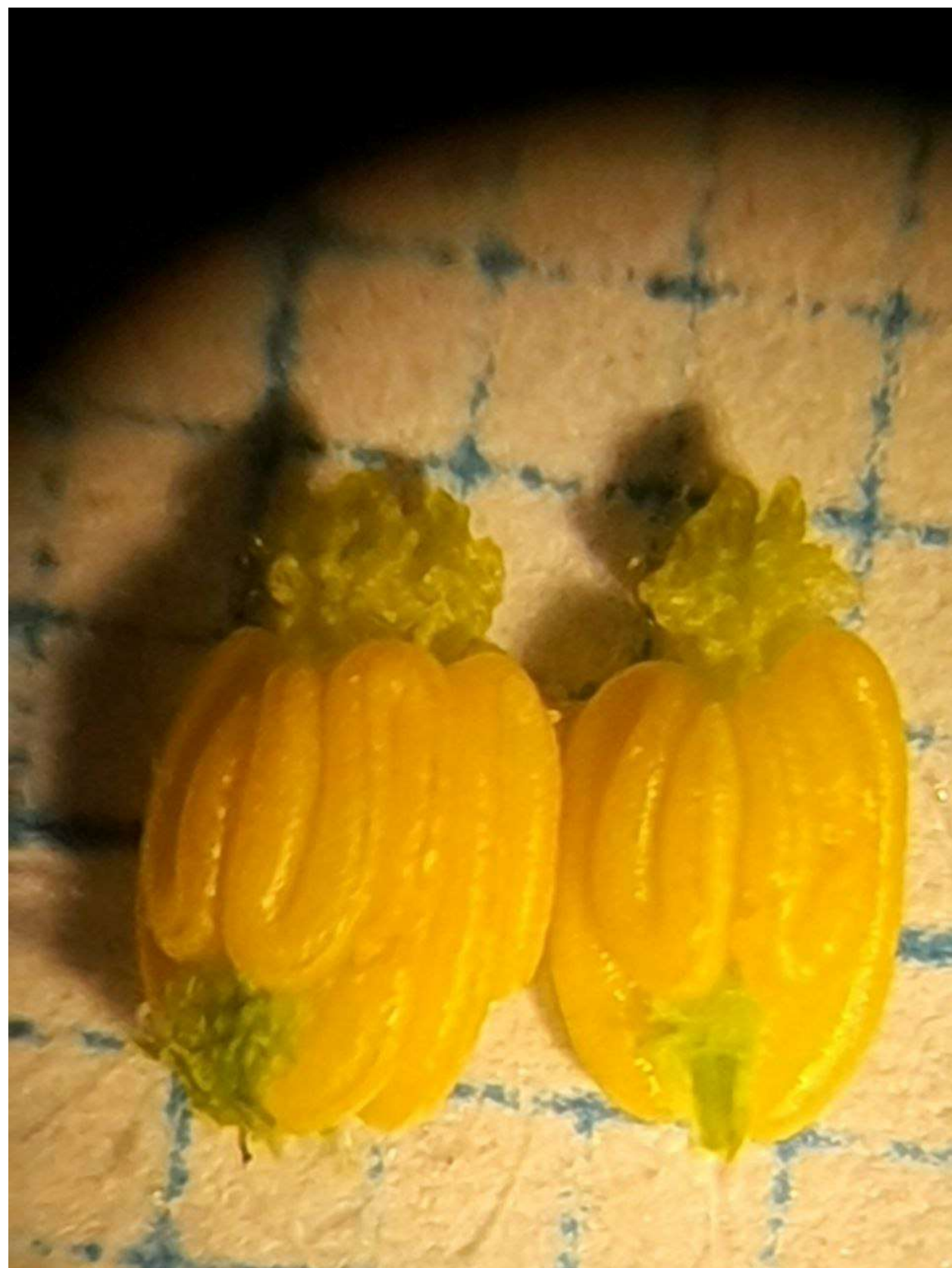

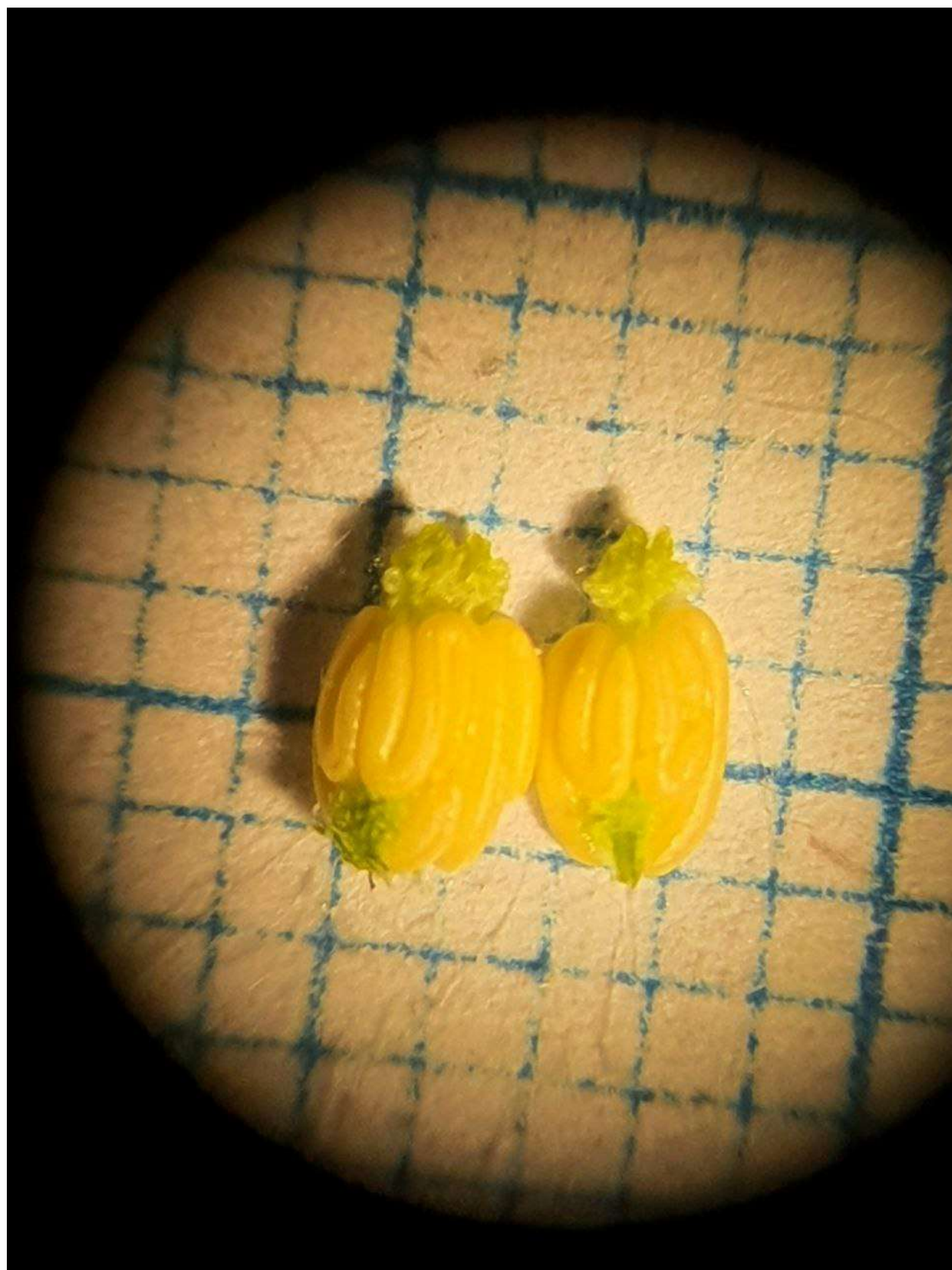

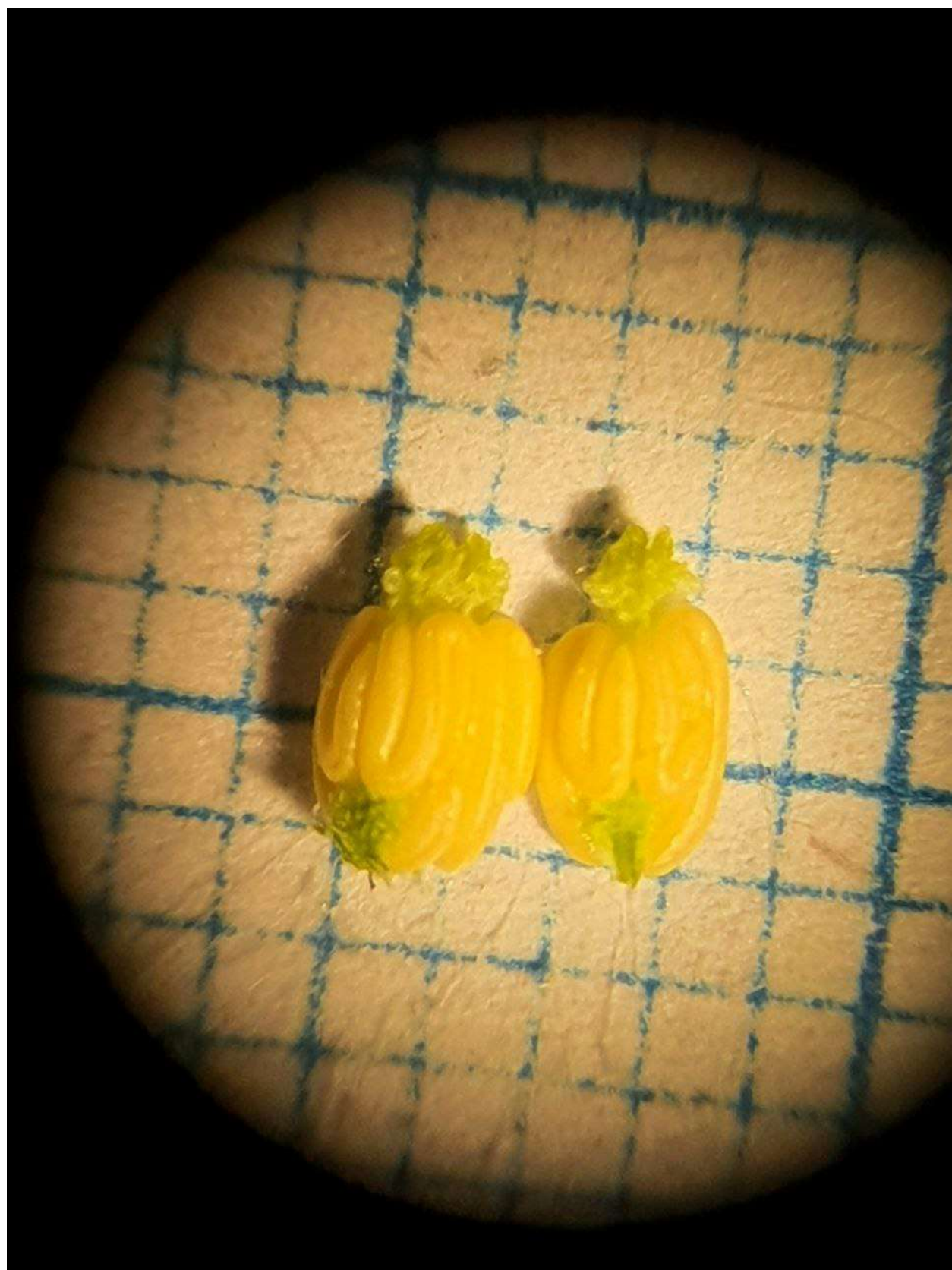

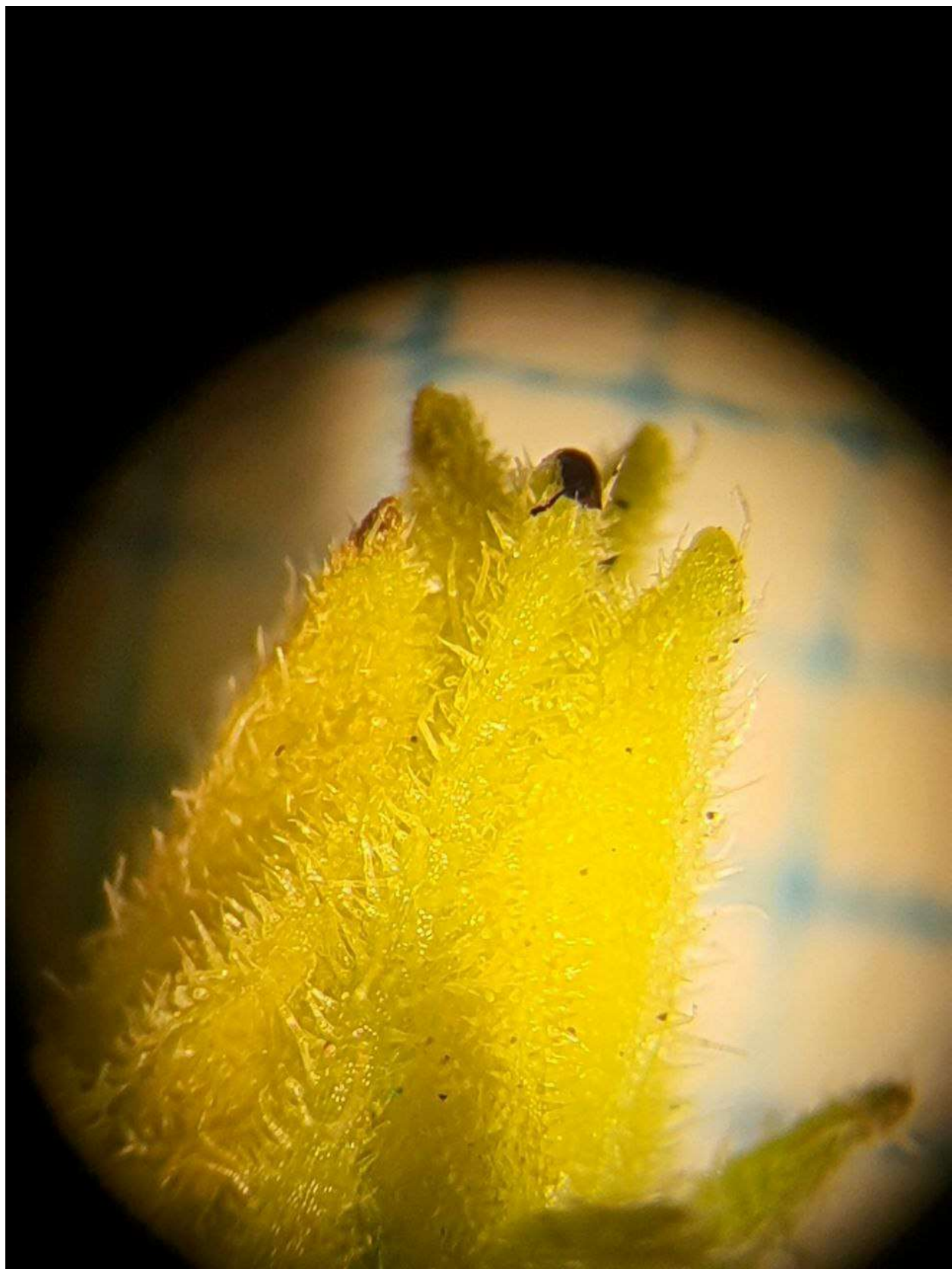

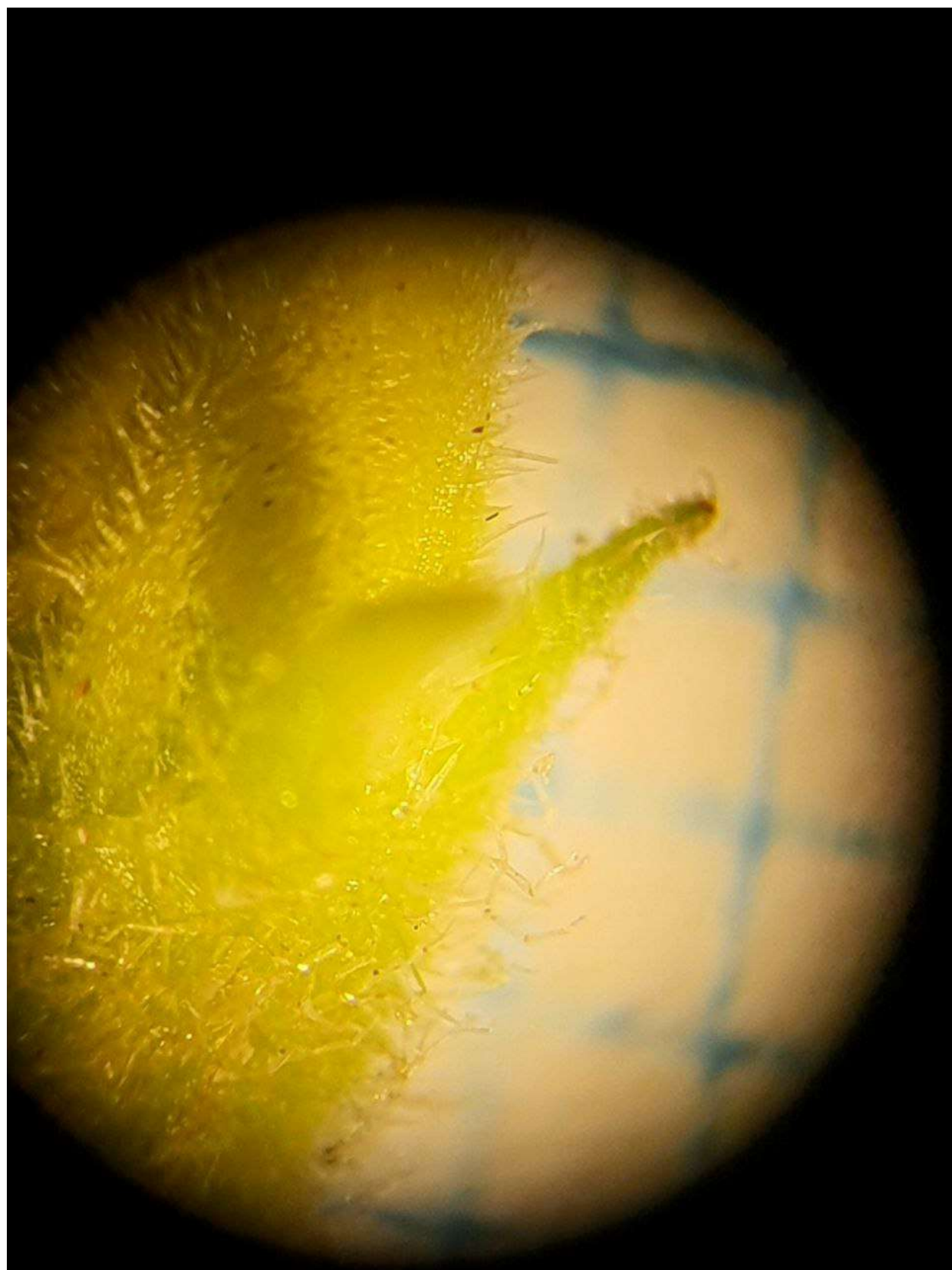

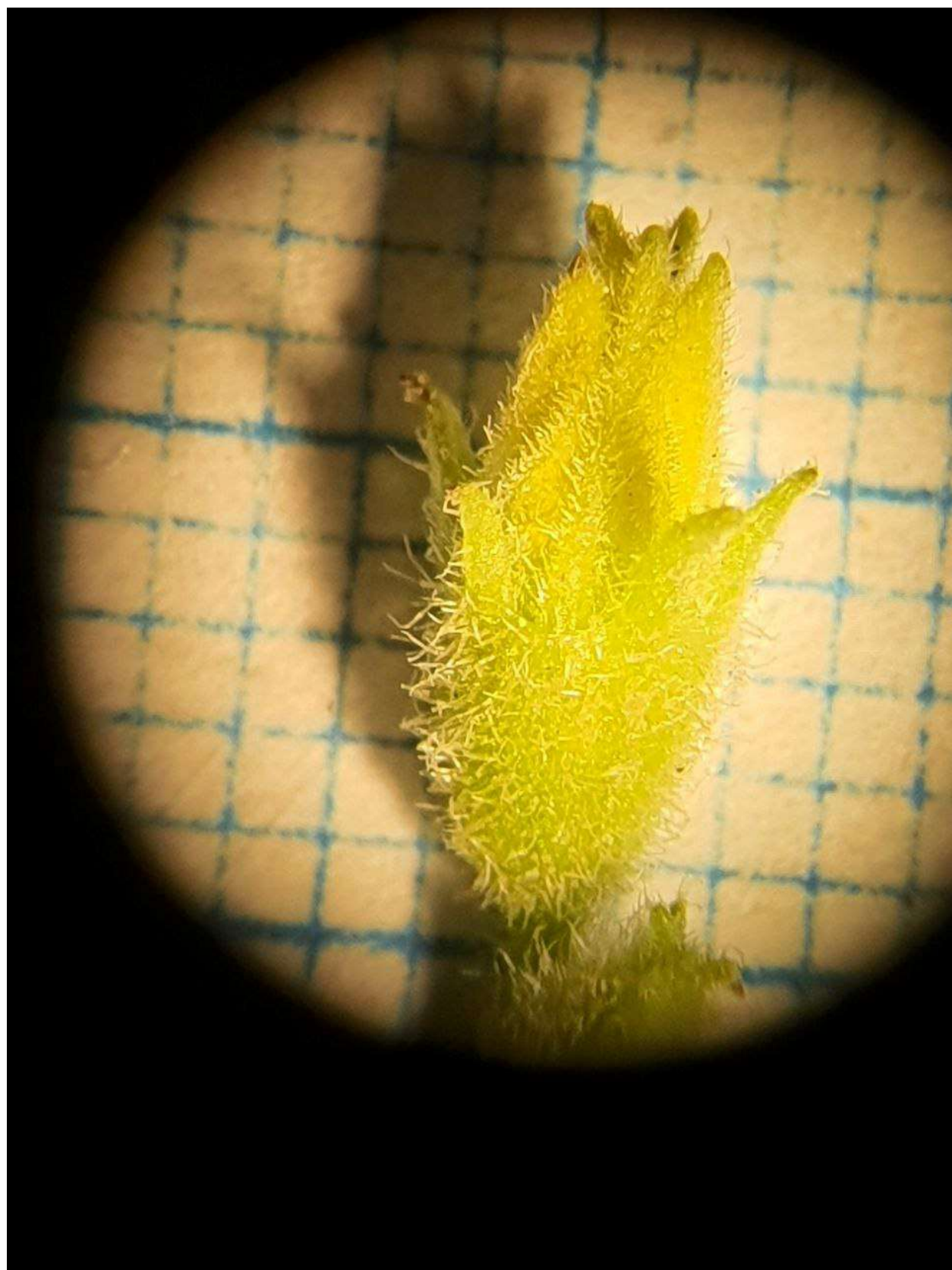

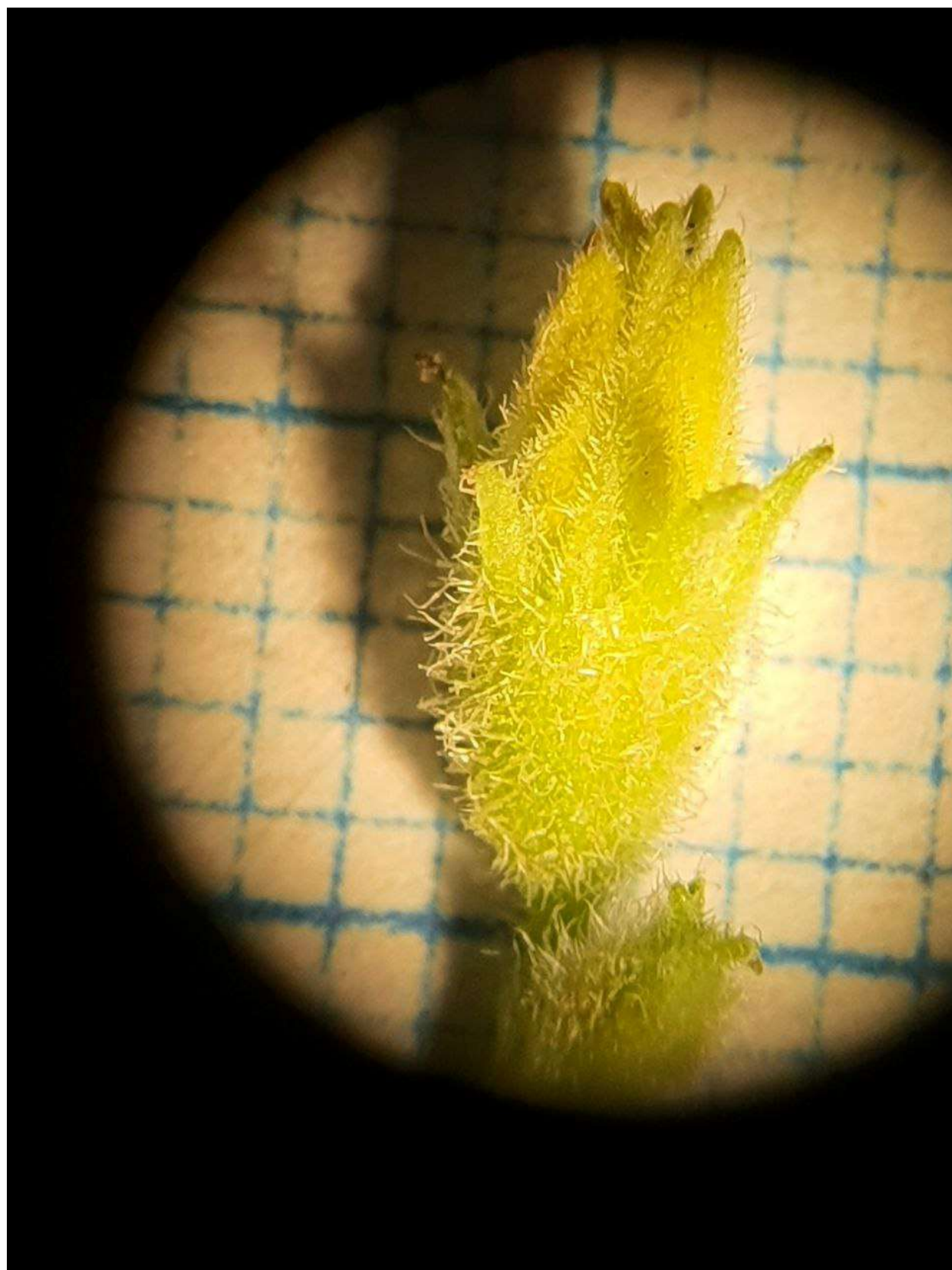

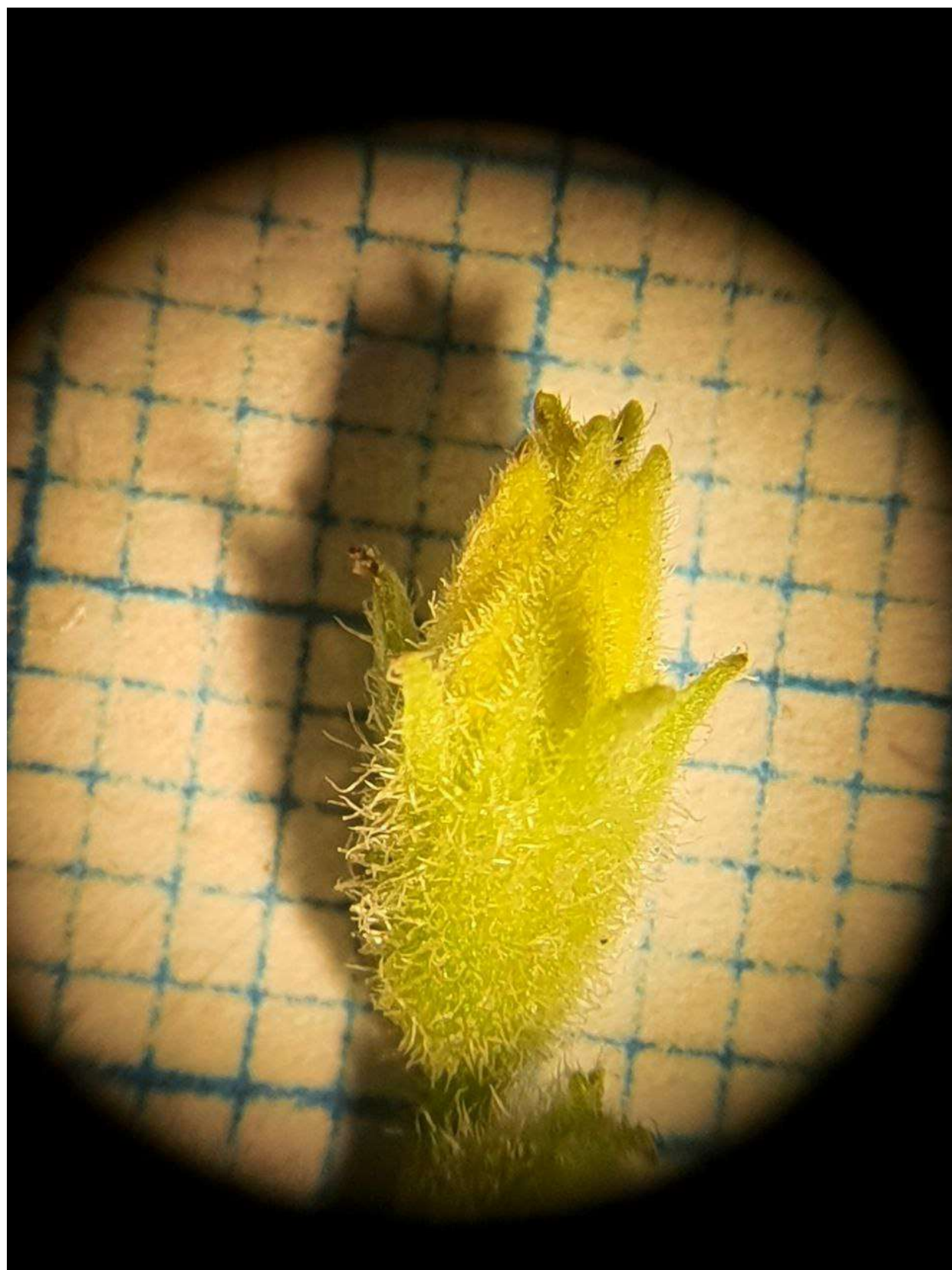

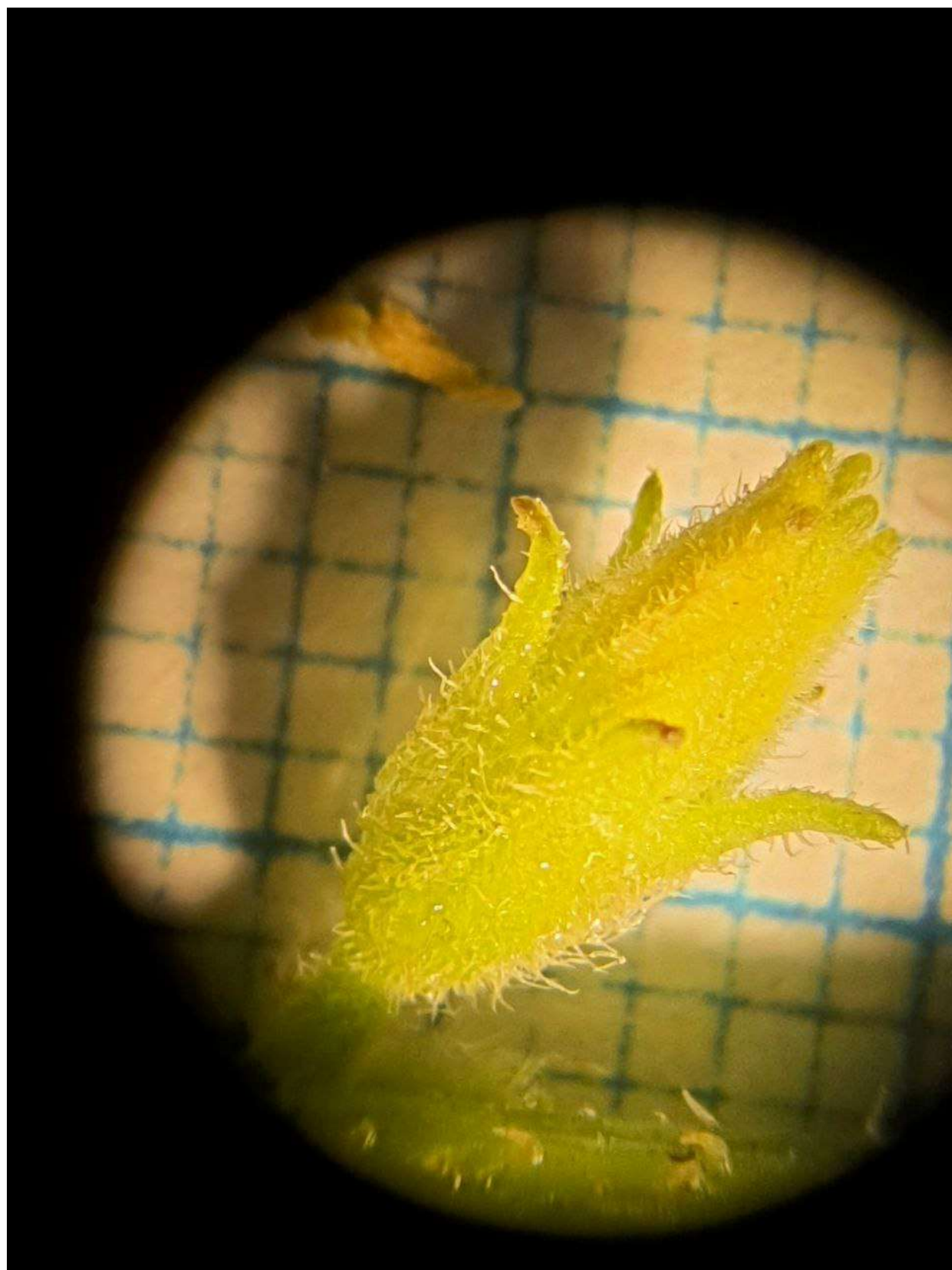

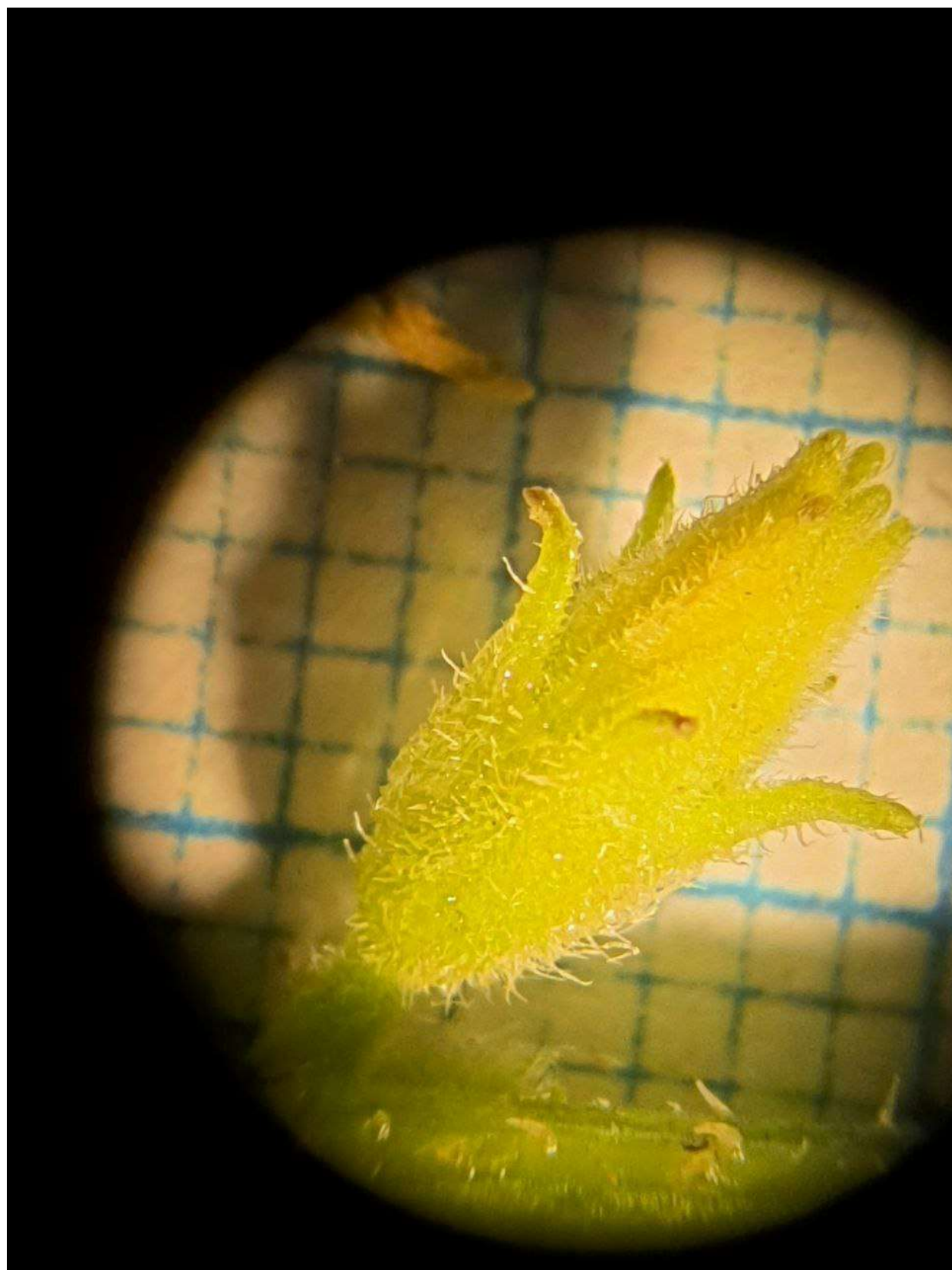

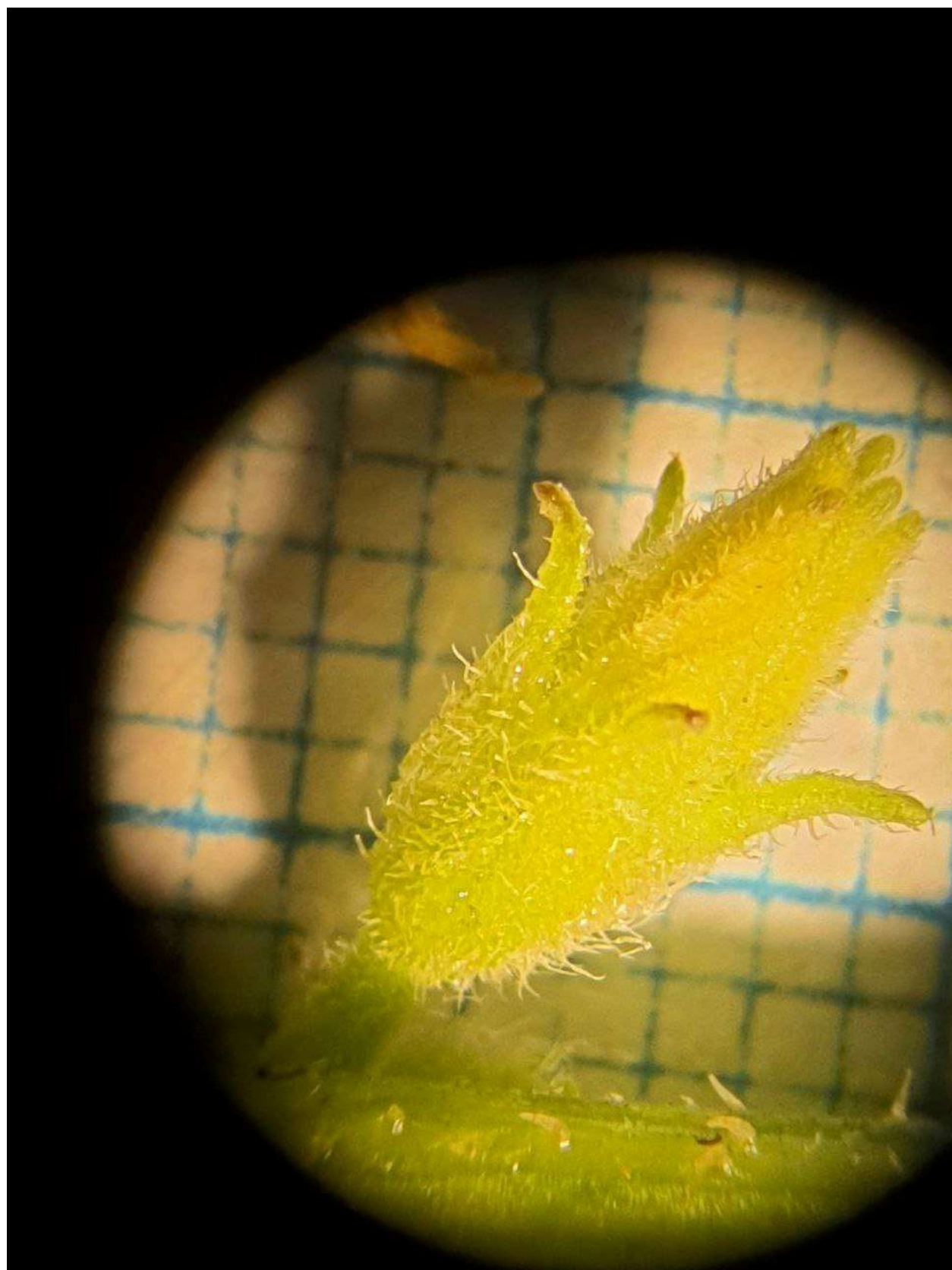

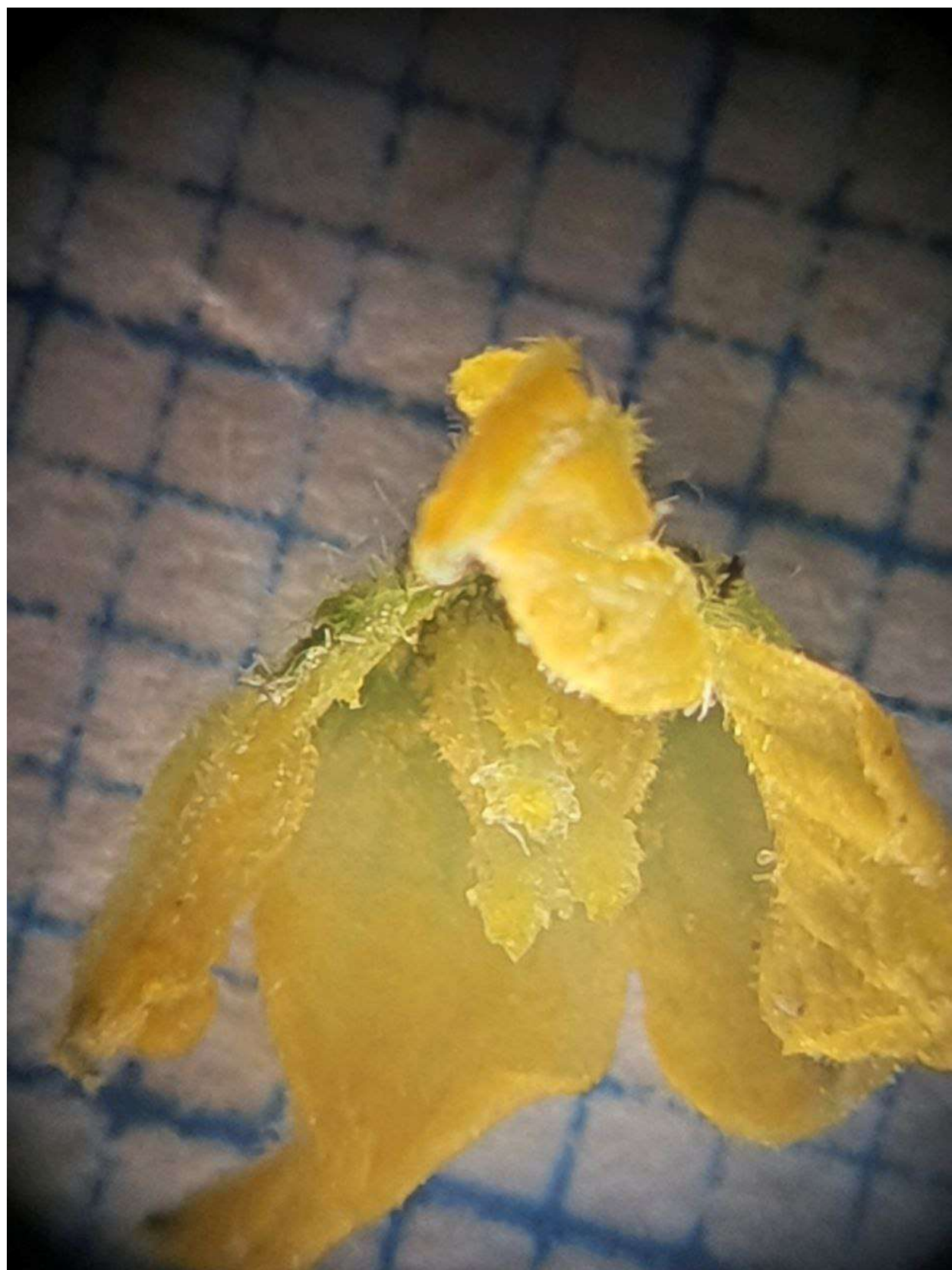

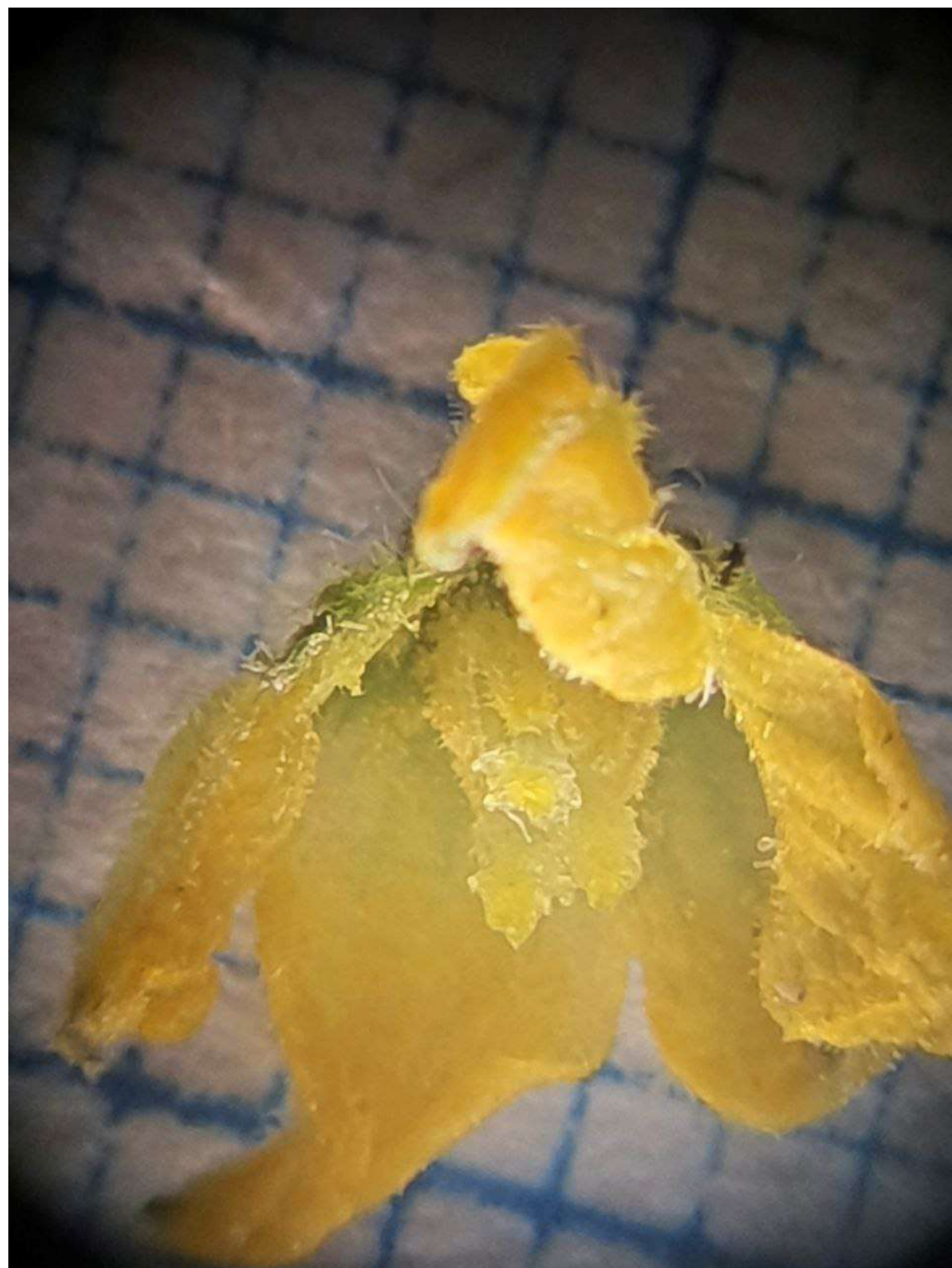

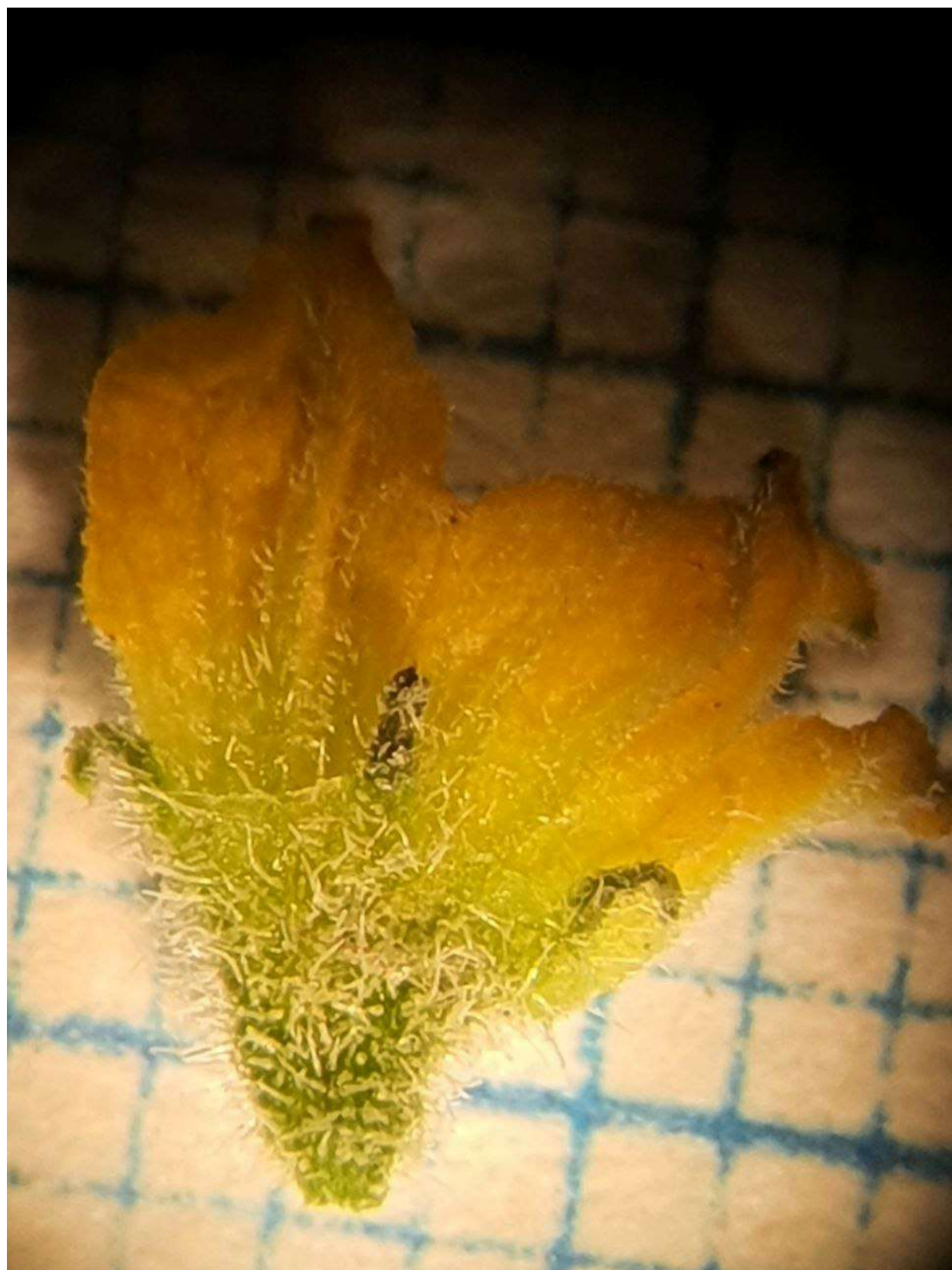

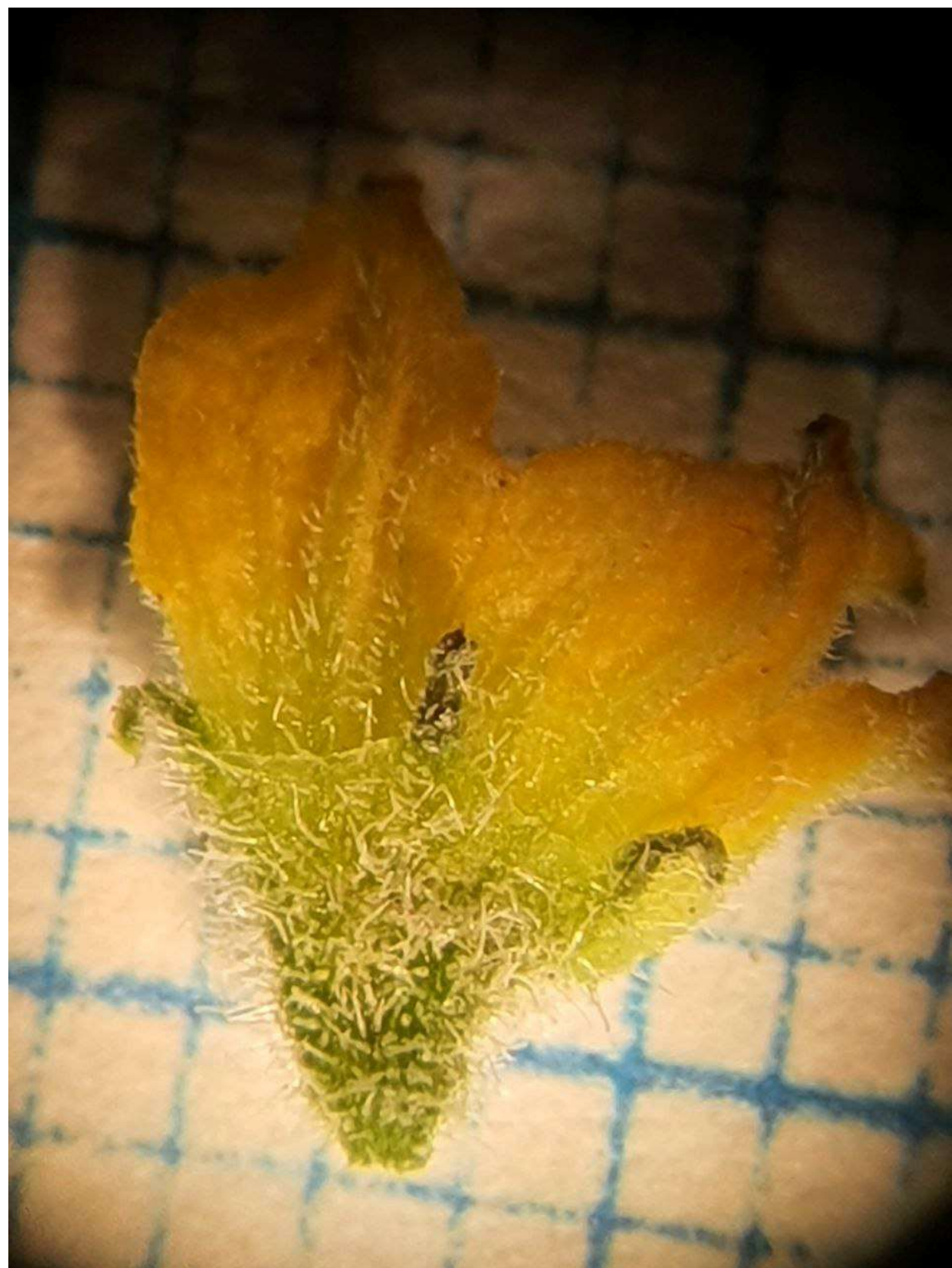

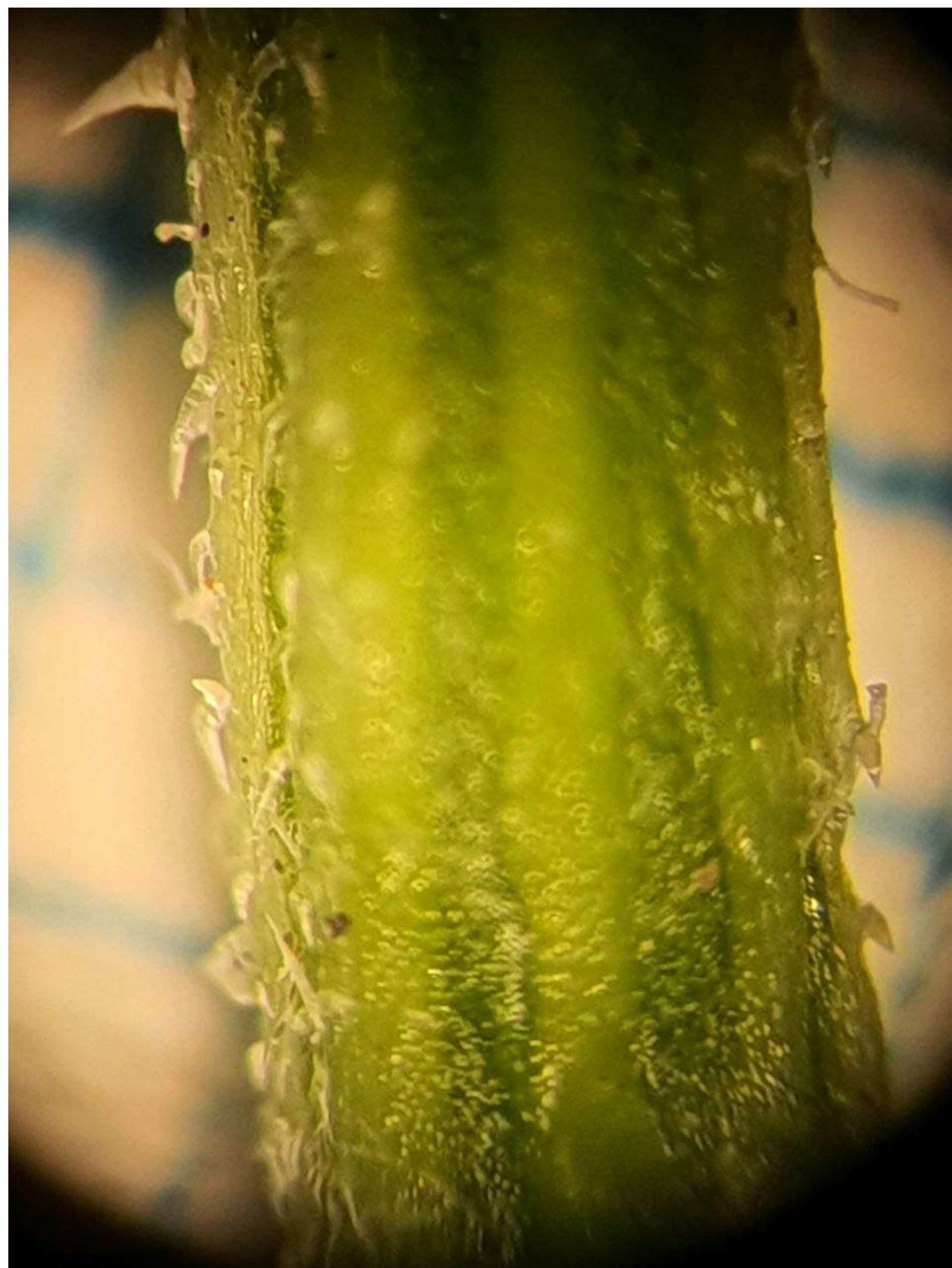

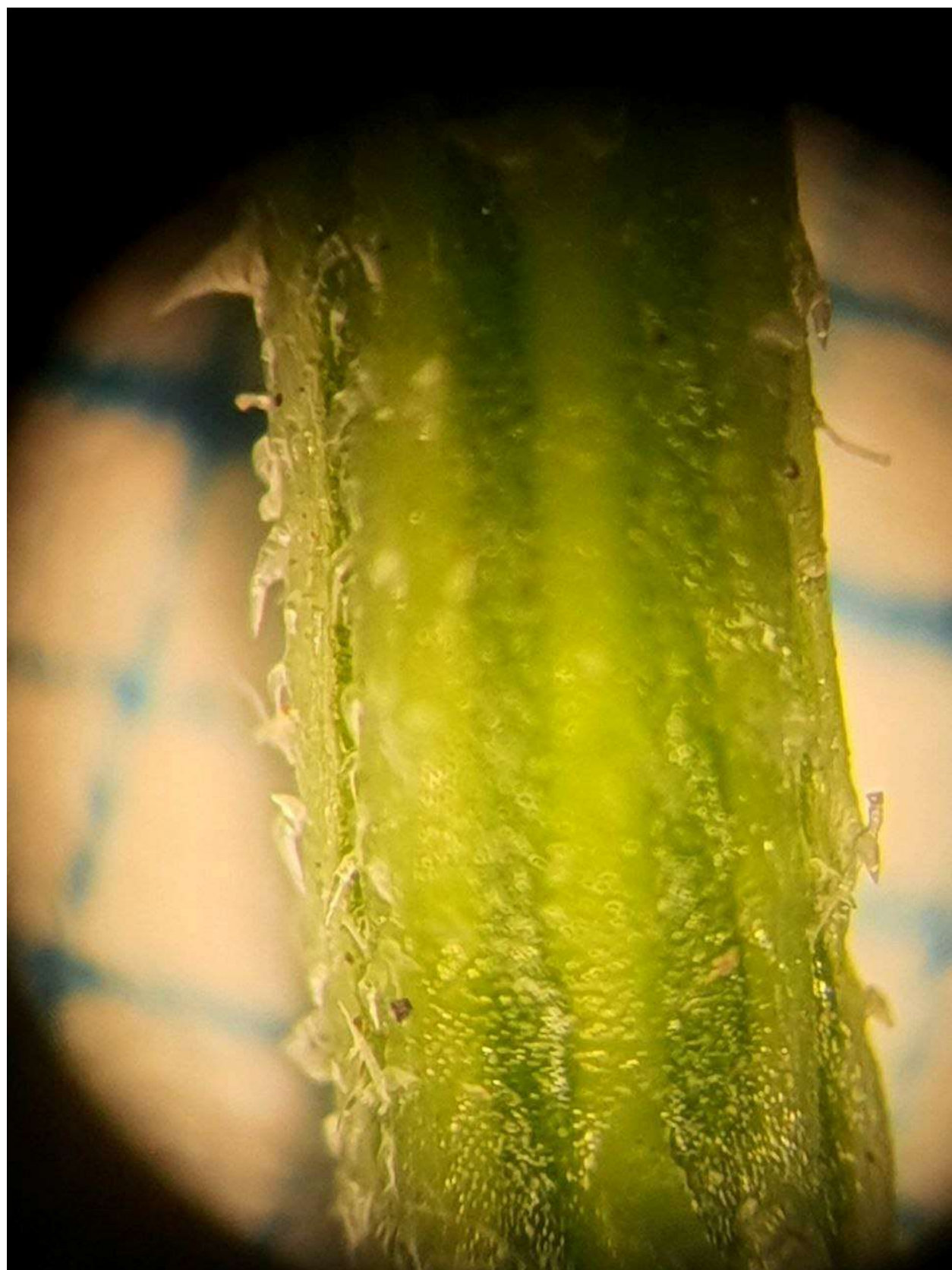

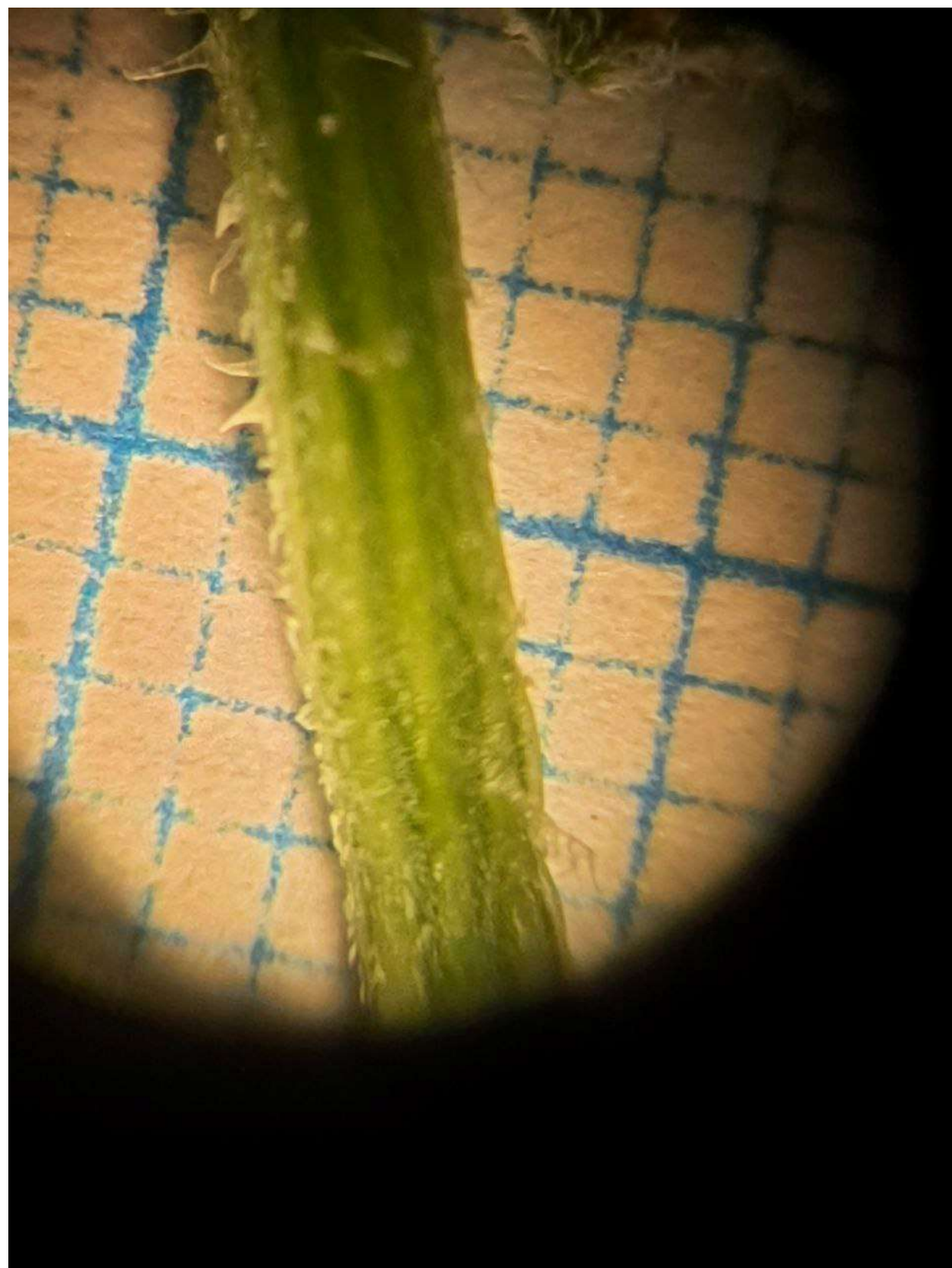

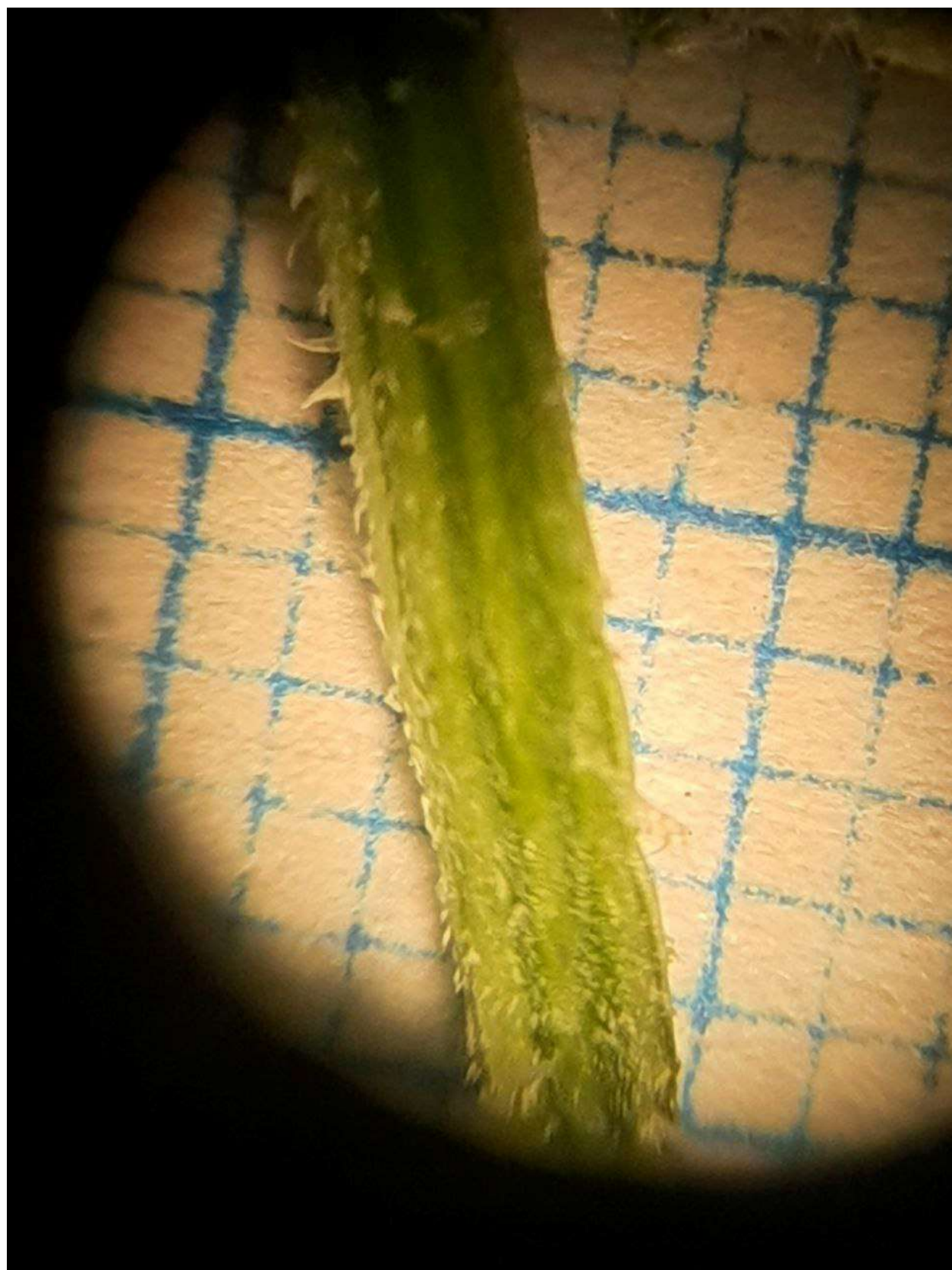

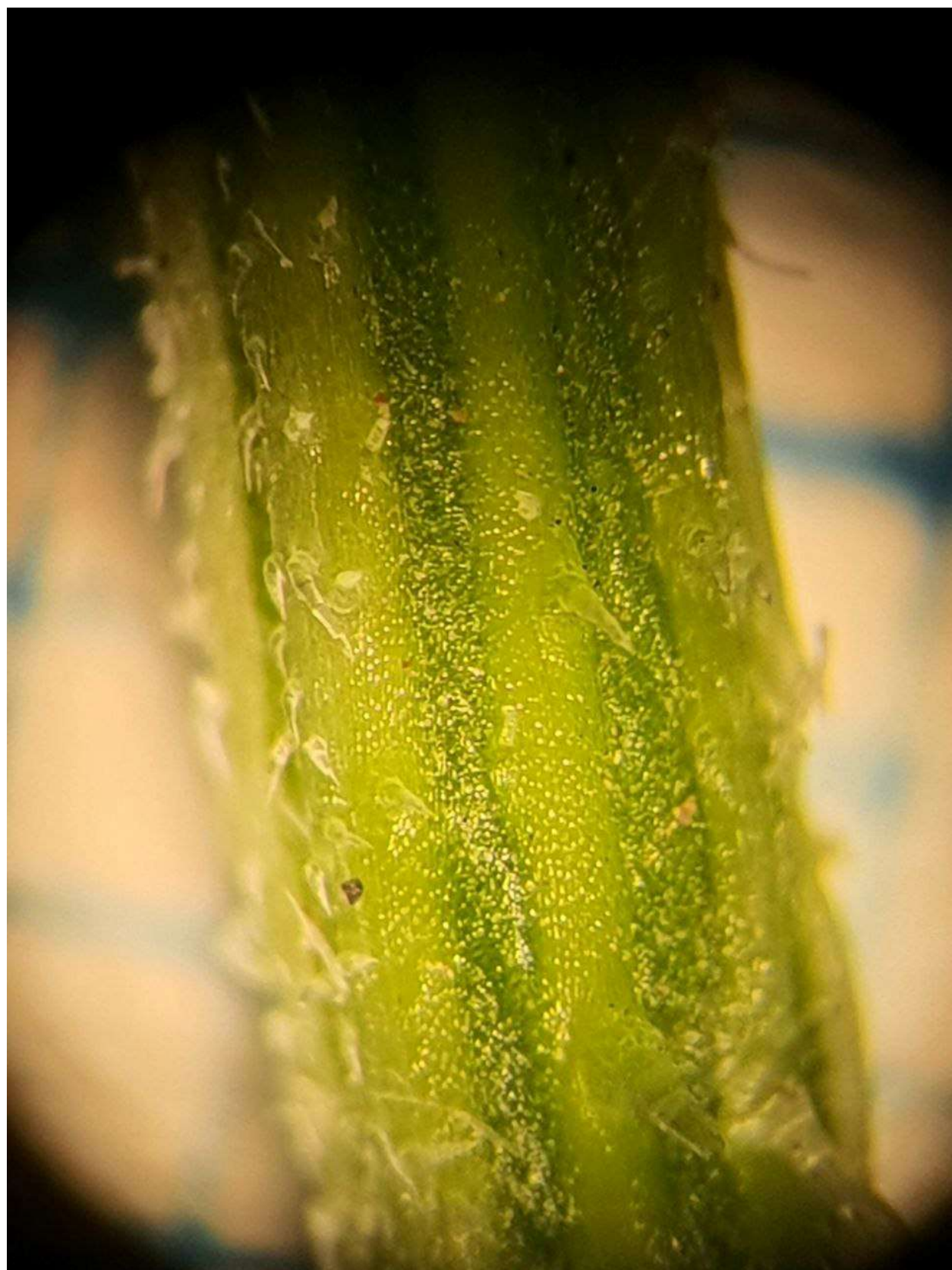

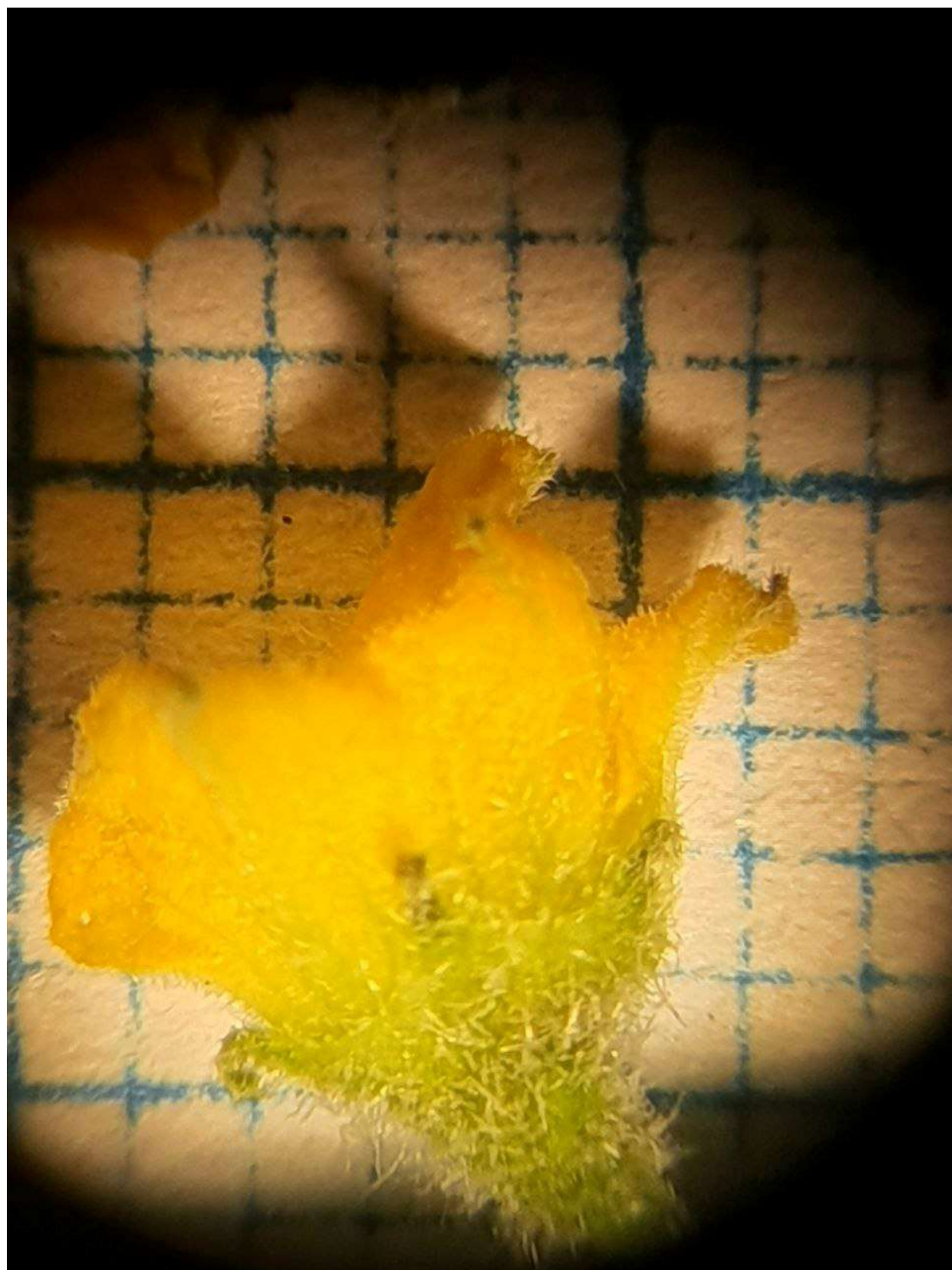

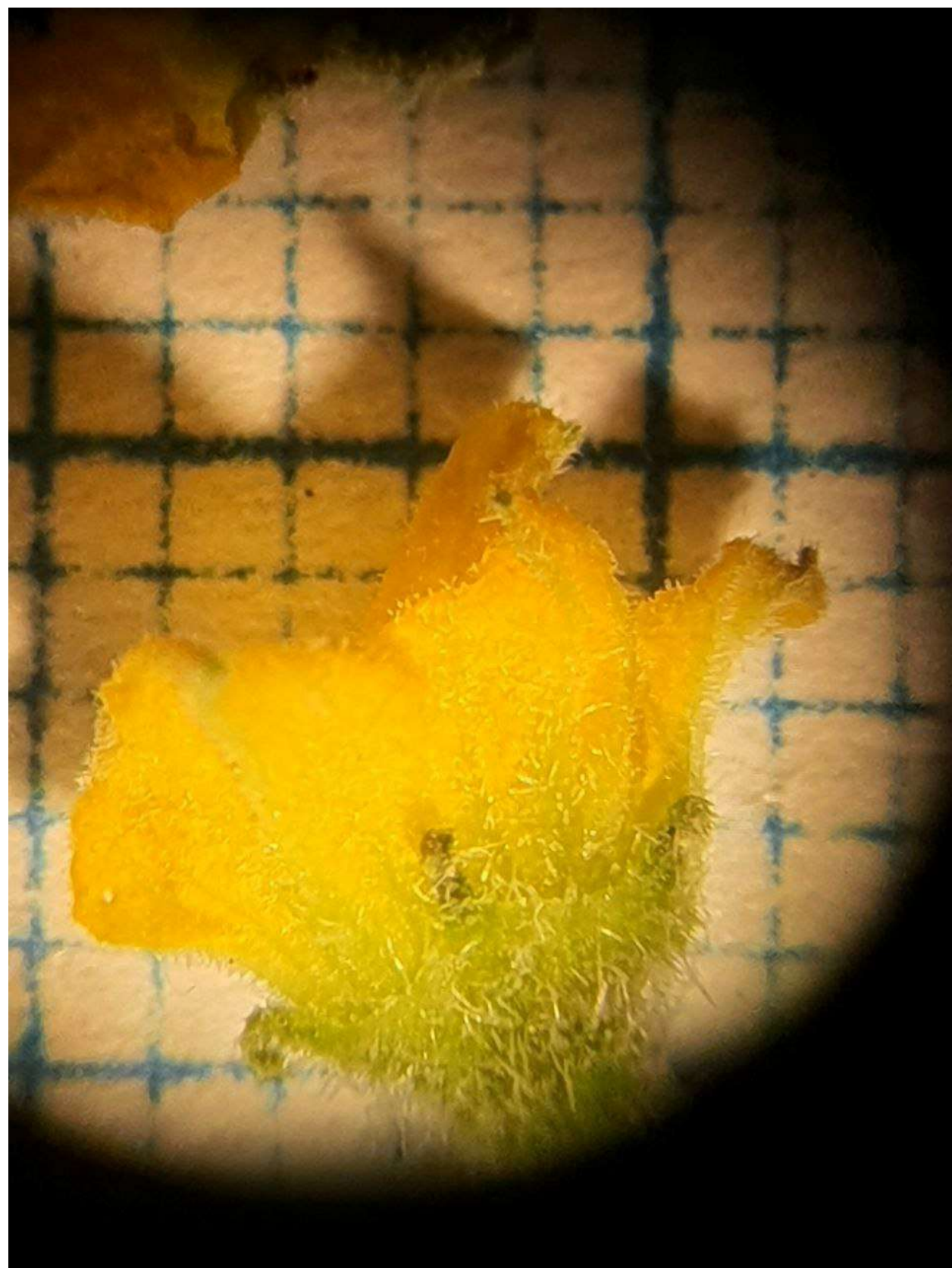

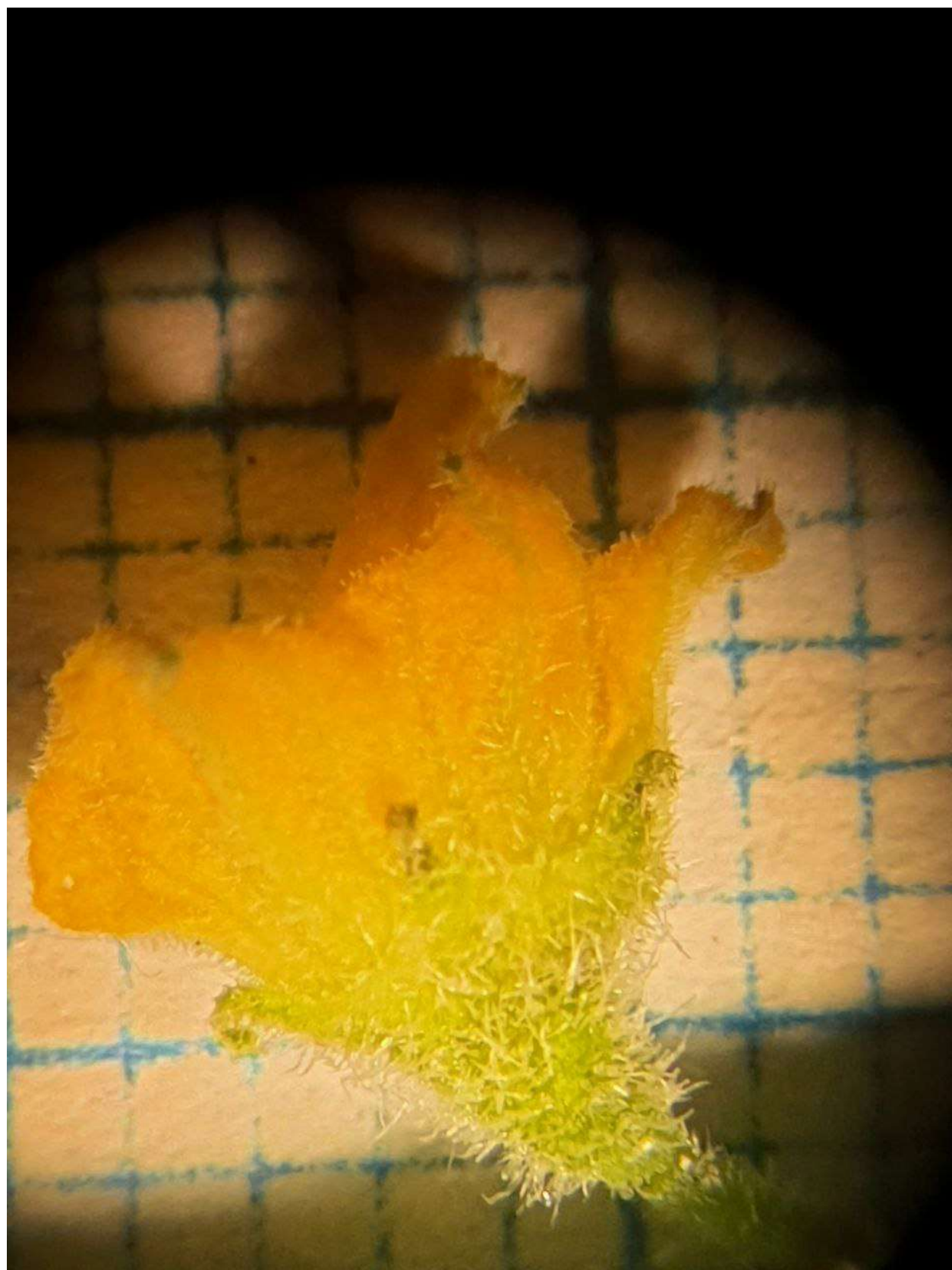

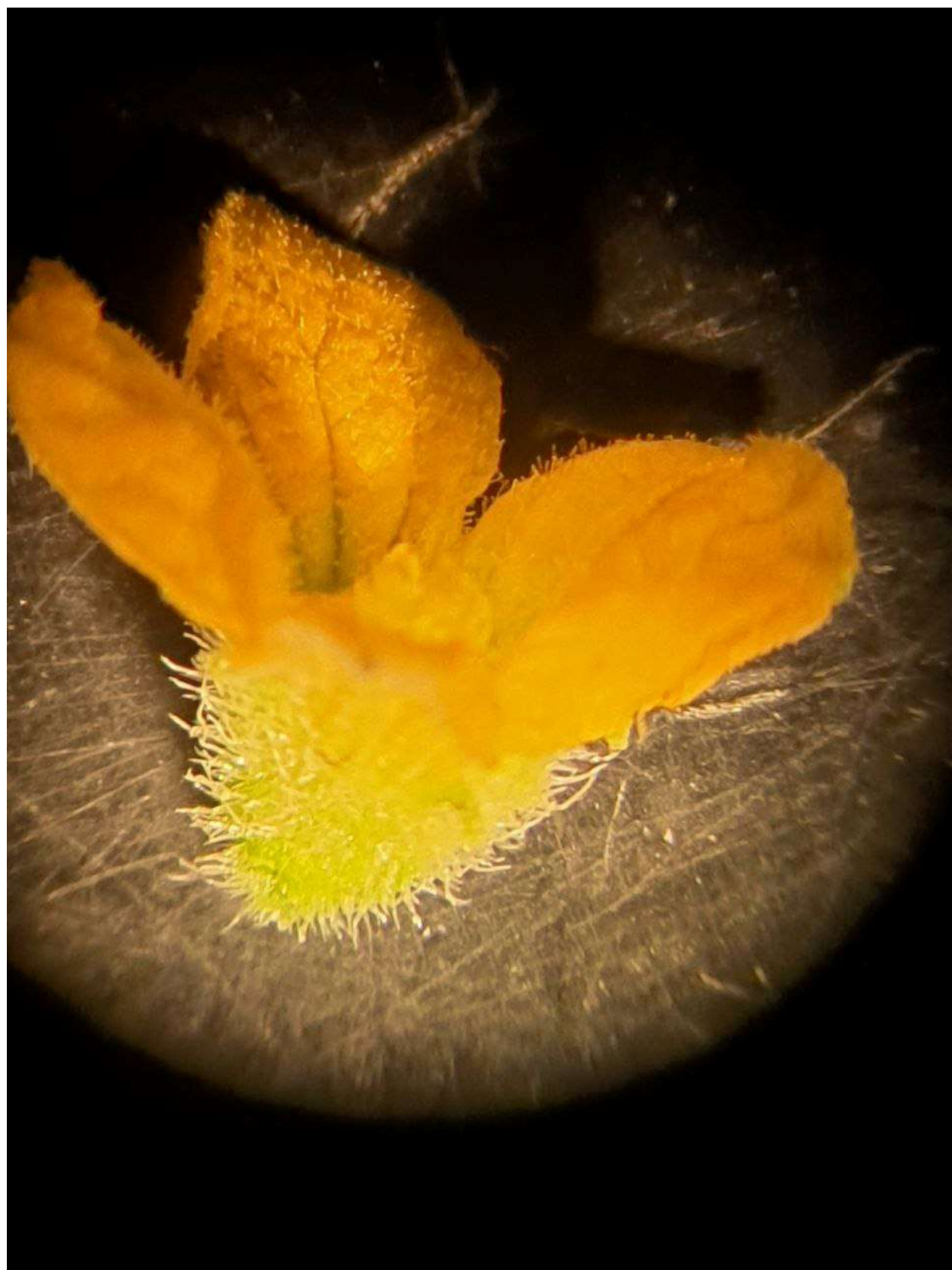

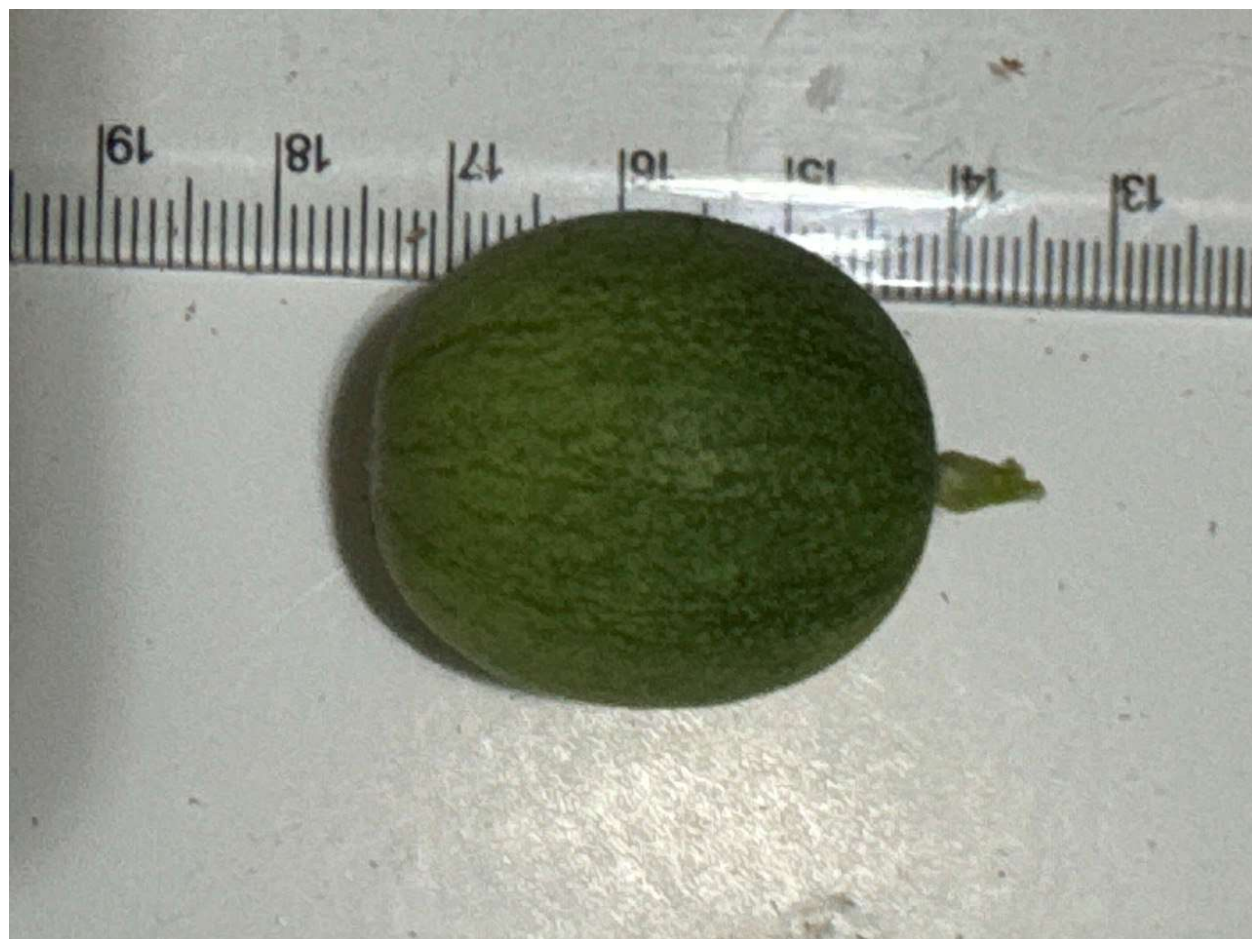

Supplement: Supplementary file 2 — Supplementary Material 2 [file 41598_2026_47246_MOESM2_ESM.pdf]
